# Comparative analysis of codon usage bias in chloroplast genomes of ten medicinal species of Rutaceae

**DOI:** 10.1186/s12870-024-04999-5

**Published:** 2024-05-20

**Authors:** Lianwen Shen, Shengqun Chen, Mei Liang, Shang Qu, Shijing Feng, Dawei Wang, Gang Wang

**Affiliations:** 1 Key Laboratory for Forest Resources Conservation and Utilization in the Southwest Mountains of China Ministry of Education, Southwest Forestry University, Kunming, 650224 China; 2 Key Laboratory for Forest Genetics and Tree Improvement and Propagation in Universities of Yunnan Province, Southwest Forestry University, Kunming, 650224 China; 3 Guizhou Academy of Forestry, Guiyang, 550005 China; 4 Guizhou Province Forestry Science and Technology Extension Station, Guiyang, 550000 China; 5 College of Forestry, Guizhou University, Guiyang, 550025 China

**Keywords:** Codon usage bias, Rutaceae family, Natural selection, Optimal codon

## Abstract

Rutaceae family comprises economically important plants due to their extensive applications in spices, food, oil, medicine, etc. The Rutaceae plants is able to better utilization through biotechnology. Modern biotechnological approaches primarily rely on the heterologous expression of functional proteins in different vectors. However, several proteins are difficult to express outside their native environment. The expression potential of functional genes in heterologous systems can be maximized by replacing the rare synonymous codons in the vector with preferred optimal codons of functional genes. Codon usage bias plays a critical role in biogenetic engineering-based research and development. In the current study, 727 coding sequences (CDSs) obtained from the chloroplast genomes of ten Rutaceae plant family members were analyzed for codon usage bias. The nucleotide composition analysis of codons showed that these codons were rich in A/T(U) bases and preferred A/T(U) endings. Analyses of neutrality plots, effective number of codons (ENC) plots, and correlations between ENC and codon adaptation index (CAI) were conducted, which revealed that natural selection is a major driving force for the Rutaceae plant family’s codon usage bias, followed by base mutation. In the ENC vs. CAI plot, codon usage bias in the Rutaceae family had a negligible relationship with gene expression level. For each sample, we screened 12 codons as preferred and high-frequency codons simultaneously, of which GCU encoding Ala, UUA encoding Leu, and AGA encoding Arg were the most preferred codons. Taken together, our study unraveled the synonymous codon usage pattern in the Rutaceae family, providing valuable information for the genetic engineering of Rutaceae plant species in the future.

**Supplementary Information:**

The online version contains supplementary material available at 10.1186/s12870-024-04999-5.

## Introduction

The Rutaceae plant family is comprised of approximately150 genera and 1600 species, which are distributed all over the world, mainly in tropical and subtropical regions, and a few are distributed in temperate regions. Many genera and species have significant economic importance as food, oil production, and medicinal sources [1]. In addition, some tree species are suitable for planting on mountains and have gradually become one of the most important tree species for countrywide conversion of farmland to forest projects [2]. *Zanthoxylum bungeanum* belongs to the *Zanthoxylum* genus of the Rutaceae family, which has a long history of cultivation in China. There are approximately 45 species and 13 varieties of *Zanthoxylum* species cultivated in China, and the main varieties are *Zanthoxylum bungeanum, Zanthoxylum armatum*, *Zanthoxylum schinifolium*, etc [3, 4]. *Zanthoxylum. bungeanum* is an important spice, oil plant and a traditional Chinese medicine tree species that has a warming effect. It can dispels cold, promote blood circulation, relieve pain, and treat cold-related ailments, bruises, blood stasis, swelling, and pain [5]. *Toddalia asiatica*, a folk medicine used in China for hundreds of years, belongs to the genus *Toddalia* of the Rutaceae family [6]. It has significant pharmacological effects such as anti-inflammatory, analgesic, hemostatic, and anti-tumor. It is also used in the treatment of cardiovascular diseases. In addition, *T. asiatica* has potential effects on rheumatism, pain, wound bleeding, and contusions; therefore, it has a wide range of clinical applications [7]. *Citrus trifoliata*, *Ruta graveolens*, and *Citrus reticulata* belong to the genus *Citrus* of the Rutaceae family. *Citrus trifoliata* is used in traditional Chinese medicine to treat digestive ulcers as well as various gastrointestinal diseases and cancers due to its antiphlogistic properties [8]. *Ruta graveolens* contains quinoline alkaloids and coumarins, such as graveoline, graveolininerutacultin, rutaretin, and suberenone. In folk medicine, it is used as a stimulant, analgesic, anti-inflammatory, antiandrogen, hypoglycemic, hypolipidemic, xanthine oxidase inhibitor, and anticancer agent [9]. *Citrus reticulata* pericarp (Chenpi, CP) has a medicinal history known for about thousands of years in China, and it is listed as a “top grade” medicine in “Shen Nong’s Herbal Classic”. In traditional Chinese medicine, CP is used to treat diseases related to the digestive, respiratory, and cardiovascular systems, as well as to regulate “Qi” and promote blood flow in the body [10]. *Tetradium ruticarpum* and *Phellodendron chinense* belongs to the genus *Tetradium* of the Rutaceae family. For thousands of years, *T. ruticarpum* has been used to treat headaches, abdominal colic, and hypertension in traditional Chinese medicine [11, 12]. *Phellodendron chinense* bark, which is used in traditional Chinese medicine, contains berberine hydrochloride, presenting anti-inflammatory and antiseptic properties [13]. Altogether, it can be concluded that the Rutaceae family plants have great potential for pharmaceutical use.

Chloroplast is a major organelle in the green plant, characterized by its genome. It participates in photosynthesis to provide energy for physiological activities in the plant [14]. The chloroplast genome consists of four parts: a large single copy (LSC), a small single copy (SSC), and two inverted repeats (IRs) [15]. Chloroplast genomes are generally conserved but have undergone intra- and inter-specific rearrangements during evolution, including IR expansion and contraction [16]. Plant chloroplast genomes are widely implicated in phylogeny, taxonomic identification, and gene expression studies [17]. Codons are crucial in transferring genetic information from mRNA to protein in an organism [18]. There are 61 codons known to encode 20 amino acids during protein translation in the organism; therefore, the codons have degeneracy [19]. Except for the tryptophan and methionine, all other amino acids are encoded by more than one codon, wherein codons encoding the same amino acid are referred to as synonymous codons. These synonymous codons are biased in encoding a few amino acids, termed codon usage bias [18, 19].

Interestingly, codon usage in most prokaryotes and eukaryotes is not random. Highly expressed genes tend to be biased toward some synonymous codons and tend to use them frequently in the organism. Thus, ‘optimal codons’ are defined as the codons that are enriched in highly expressed genes. The preference of many organisms for using a particular synonymous codon is primarily influenced by natural selection [20–22]. Altogether, codon bias is influenced by several factors, such as GC content, gene expression, gene length, and natural selection [23]. Codon bias can affect mRNA biosynthesis, translation elongation rate, protein folding, and other downstream biological functions [24]. A few studies have hypothesized that codon bias reduces the diversity of isoacceptor tRNAs and thus reduces the metabolic load, benefiting organisms under fast-growing conditions [25]. The codon bias studies clearly show that codon bias can profoundly impact the expression of heterologous proteins [26]. Identifying codon bias in genes or genomes is essential for understanding the molecular mechanism of their expression in different organisms and for revealing the long-term evolution of the species [27].

In recent years, researchers have studied codon usage bias in the chloroplast genomes of a few plants and discovered some common patterns. Natural selection and mutational bias have influenced the codon usage bias in the chloroplast genomes of these plant species, which prefer codons ending in A or T bases [28–31]. However, there are a few reports on the codon bias in the Rutaceae plant family. In the current study, the codon usage patterns of ten species belonging to the Rutaceae family (*Z. bungeanum, Z. schinifolium, Z. armatum, T. asiatica, C. trifoliata, R. graveolens, C. reticulata, T. ruticarpum, P. chinense*, and *P. amurense*)) were analyzed at the chloroplast genomes level. We explored the reasons underlying codon usage bias formation and determined the optimal codons for each species. Through the result of this study, we gained novel insights into understand how Rutaceae plants use codons, to help genetic engineering for future research on the Rutaceae.

## Materials and methods

### Source of CDS data

Ten chloroplast genome sequences of Rutaceae plant species were obtained from the NCBI database (National Center for Biotechnology Information (nih.gov)) on Jan. 10, 2023, including three *Pananthus* species, one *Toddaria* species, three *Citrus* species, one *Tetradium* species, and two *Ferrodendron* species (Table 1). Coding sequences (CDSs) were extracted from these ten chloroplast genomes according to the annotation information obtained from NCBI. MAGE software was used to process the original CDSs of the chloroplast genomes. The sequences were selected based on the following criteria: (1) Sequence begins with the start codon ATG and ends with the stop codon TAA, TAG, or TGA; (2) No stop codon occurs in the middle of the sequence; (3) the CDS is ≥ 100 base pairs in length [29, 31]. In total, 727 CDSs (Supplementary date 1) were selected from chloroplast genomes and analyzed for codon abuse.

**Table 1:** Chloroplast genomes associated information of ten medicinal Rutaceae plant species

Genus	Species	Genome Size (bp)		CDS Numbers		GeneBank ID
		Before Processing	After Processing	Before selection	After selection	
*Zanthoxylum*	*Zanthoxylum bungeanum*	158,978	64,704	87	72	MW602886.1
	*Zanthoxylum schinifolium*	158,963	64,794	87	72	KT321318.1
	*Zanthoxylum armatum*	158,610	64,710	87	72	MT990984.1
*Toddalia*	*Toddalia asiatica*	158,434	65,685	87	73	NC_056094.1
*Citrus*	*Citrus trifoliata*	160,260	65,817	91	73	NC_057088.1
	*Ruta graveolens*	157,434	66,108	87	73	MN326012.1
	*Citrus reticulata*	160,699	66,243	87	74	MW147176.1
*Tetradium*	*Tetradium ruticarpum*	158,446	65,748	86	71	MZ145060.1
*Phellodendron*	*Phellodendron chinense*	158,537	64,956	88	73	MT916287.1
	*Phellodendron amurense*	158,442	64,677	88	73	KY707335.1

### Nucleotide composition analysis

The CodonW1.4.2 and the online software CUSP (http://emboss.toulouse.inra.fr/cgi-bin/emboss/cusp) [32] were used to calculate the A3s, T3s, C3s, and G3s at the third location of chloroplast genomes in ten Rutaceae frequency of occurrence, and GC1, GC2, GC3 content, and average GC (GCall) base content.

### Index of codon usage bias

Codon usage bias is commonly measured by the relative synonymous codon usage (RSCU), the codon adaptation index (CAI), and the effective codon number (ENC) [33, 34]. Studies have shown that several factors, including GC content, mutational pressure, natural selection, expression levels, and protein length, affect codon usage. Due to strong evolutionary pressure, genetically related species share similar codon usage characteristics [35].

The ENC, CAI, and RSCU were calculated using CodonW1.4.2. ENC indicates the strength of codons encoding amino acids, and the ENC value ranges from 20 to 61. Codon bias is more substantial when the ENC value is smaller [36]. CAI measures the degree of preference for preferred codons, mainly in highly expressed genes. CAI values range from 0 to 1, with higher CAI values corresponding to stronger codon biases and higher expression levels [37]. The RSCU value is calculated to estimate the codon usage bias of genes. It is defined as the ratio of the usage frequency of synonymous codons to their expected frequency, which is the average usage frequency of all codons encoding amino acids. The formula for calculating the relative usage of synonymous codons is as follows:

$$\text{R}\text{S}\text{C}\text{U}=\frac{\text{X}\text{i}\text{j}}{{\sum }_{\text{j}}^{\text{n}\text{i}} \text{X}\text{i}\text{j}}\text{n}\text{i}$$

Xij indicates the frequency of codon j encoding the i-th amino acid, and Ni is the number of codons j encoding the i-th amino acid. An codon has an RSCU value greater than 1 for a codon indicates that this codon is used more frequently among all synonymous codons [38, 39]. Considering the first and second nucleotides of the triplet codon as the ordinate and the third nucleotide as the abscissa, R was used to build a heat map to analyze the RSCU value.

### Analysis of the source of Codon Usage Bias

A neutral plot was generated to analyze the effect of mutation pressure on codon bias. The horizontal axis in the plot is GC3, where the vertical axis is GC12. Here, GC12 is the average value of GC1 and GC2. The correlation coefficient close to 1 explains that the codon usage bias is determined by base mutation rather than natural selection pressure [40]. The effect of base composition and natural selection on codon usage bias was analyzed by ENC plots. The ENC plot was drawn with GC3 as the x-axis and ENC as the y-axis. The standard curve was drawn by ENCexp = 2 + GC3s + 29/[GC3s2 + (1 - GC3s)^2^] [41], which represents expected ENC value. GC3 is the dominant factor affecting codon usage bias when genes are on or near the curve, whereas natural selection dominates when genes are below the curve [42].

Several studies have shown that GC3 affects the principal factor of codon usage bias when genes are on or near the curve, whereas natural selection is principal when genes are below the curve [43]. To further analyze the composition characteristics of the base at the third position of a codon in the chloroplast genome CDS sequences of the Rutaceae family. In the current study, we analyzed the distribution of the third base of the synonymous codon encoding 20 amino acids in 10 samples using G3/(G3 + C3) and A3/(A3 + T3) as the abscissa and ordinate, respectively. Theoretically, if codon usage bias is only influenced by mutational pressure, the frequency of A/T and G/C bases in the third position of a codon should be equal. Otherwise, it is apparent that codon bias is determined by natural selection. To determine the effect of gene expression on codon usage, scatterplots were drawn with CAI as the x-axis and ENC as the y-axis [31, 44].

### Correspondence analysis

The multivariate analysis is can assess the changes in codon and amino acid usage. Correspondence analysis (COA) is considered the most common and appropriate method for multivariate analysis of contingency table data, such as codon usage values [45]. COA can identify significant sources of inconsistency in the data set. The COA is performed to study genome-wide changes in codon usage bias [46]. R is used to perform corresponding analysis according to the RSCU value. Within the hyperdimensional quadrant, the asymmetric variation between series is broken down into 59 axes. The correlation between axes and codon usage properties decreases with the axes’ order arrangement. The axis describes the most significant change in codon usage [45].

### Identification of optimal codons

With the changed expression level of a gene, the frequency of occurrence of the same codon was altered. A codon was identified as an optimal codon when it exhibited significantly more frequent occurrence in the high-expression gene pool than in the low-expression genes [37]. RSCU values ​​are often used to identify optimal codons [47]. Herein, codons with RSCU > 1 were selected as the optimal codons.

## Results

### Analysis of codon usage patterns

#### Distribution analysis of four bases in three positions of Codon

We recorded the number of T3s, C3s, A3s, G3s, GC1, GC2, GC3, and GCall of chloroplast coding gene codons in 10 Rutaceae plant species (Table 2). We found that all four nucleotides were asymmetrically expressed on the third position of the codon in ten chloroplast genomes. The expression frequency of four nucleotides on the third location of codons in each species was as follows: T3s (45.42% ∼ 46.06%) > A3s (41.59% ∼ 42.19%) > G3s (18.93% ∼ 19.48%) > C3s (17.73% ∼ 18.28%), and the expression levels of T and A were higher than the expression of G and C. In ten Rutaceae plant species, the GC contents of the three positions of codon (GC1, GC2, and GC3) were 46.55% ∼ 46.96%, 38.38% ∼ 38.63%, and 31.00% ∼ 31.85%, respectively. The CG contents on three codon positions were less than 50%, which indicated that codon usage showed an overall bias for codons containing A and T and ending with A/T. Overall, the chloroplast-encoded genes from ten species were highly similar in base composition of coding regions and the codon usage bias of the chloroplast genomes CDSs.

**Table 2:** Codon preference parameters of ten medicinal species of the Rutaceae family

Species	T3s (%)	C3s (%)	A3s (%)	G3s (%)	GC1 (%)	GC2 (%)	GC3 (%)	GCall (%)	GCall (%) interval of gene
*C. reticulata*	45.69	17.87	41.83	19.25	46.78	38.63	31.44	38.95	32.55 ∼ 49.16
*C. trifoliata*	45.68	17.94	41.61	19.48	46.75	38.60	31.64	39.00	32.61 ∼ 49.23
*P. amurense*	46.06	17.73	42.12	18.93	46.60	38.39	31.02	38.67	31.14 ∼ 48.68
*P. chinense*	46.05	17.74	42.16	18.93	46.55	38.38	31.00	38.64	31.14 ∼ 48.68
*R. graveolens*	45.42	18.28	41.59	19.47	46.96	38.55	31.85	39.12	32.23 ∼ 48.68
*T. asiatica*	45.84	17.94	41.99	18.95	46.74	38.53	31.21	38.82	31.61 ∼ 48.44
*T. ruticarpum*	46.01	17.89	41.96	18.94	46.63	38.45	31.15	38.75	31.14 ∼ 48.92
*Z. armatum*	45.73	17.89	42.15	19.04	46.61	38.44	31.23	38.76	31.61 ∼ 48.68
*Z. schinifolium*	45.93	17.81	42.18	18.98	46.55	38.39	31.07	38.67	31.61 ∼ 48.44
*Z.bungeanum*	45.77	17.89	42.19	18.95	46.60	38.47	31.15	38.74	31.61 ∼ 48.92

**Fig. 1:**
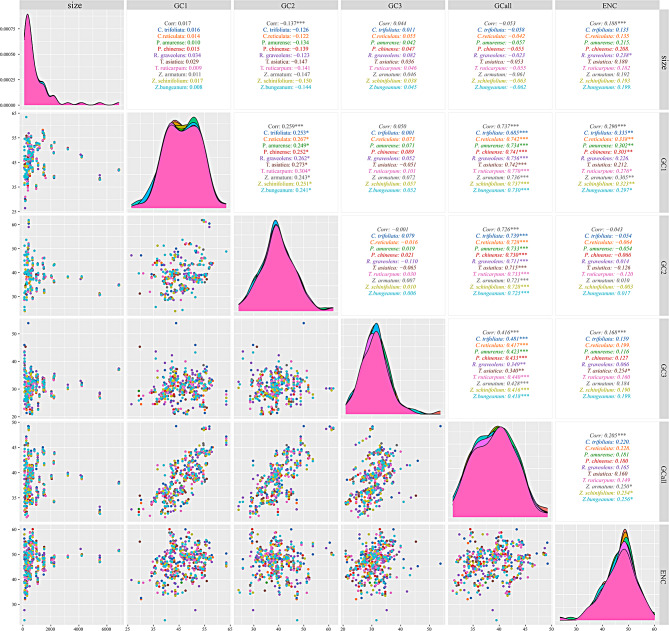
The explanation for correlation analysis of GC content at different locations. * indicates that the correlation is significant (*P* < 0.05). ** indicates that the correlation is highly significant (*P* < 0.01). *** indicates that the correlation has reached a very significant level (*P* < 0.001). The different colors of plant names in the upper right correspond to the different colored peaks in the middle and the different colored points in the lower left, respectively

The GC content is one of the essential indicators reflecting genomes characteristics. We analyzed the correlation between GC1, GC2, GC3, and the average CG content (GCall) (Fig. 1), and it was observed that GCall was significantly correlated with GC1, GC2, and GC3 (*P* < 0.001), and the correlation between GC1 and GC2 was significant (*P* < 0.02). GC3 was not correlated with GC1 and GC2. In addition, the size was not correlated with GC1, GC2, GC3, and CG. These results suggested that in ten chloroplast genomes of the Rutaceae plant family, CDSs, the GC content of codons did not correlate with the size of codons. Further, the GC composition at the first and second positions of the codon influences each other, whereas the GC composition at the third position is independent, with no relation to the GC content at the first and second positions.

#### Analysis of codon usage indicator

We analyzed CAI and ENC to evaluate the codon bias of CDSs in 10 Rutaceae chloroplast genomes. The CAI value was between 0 and 1. A larger CAI value indicates a higher gene expression level and a stronger codon preference. ENC less than 35 indicates a strong codon usage preference, whereas a value between 35 and 50 indicates a weak codon usage preference. Further, an ENC value higher than 50 indicates a weak codon usage preference. Table 3 depicts that the ENC value of the chloroplast genome codon is greater than 50. Further, the CAI value was 0.17, indicating that the chloroplast genome expression level of ten species of the Rutaceae plant family was low, and the codon preference was weak. We observed 23 genes (3.16%) with ENC values less than 35 and higher preference (Table 3), whereas the ENC values of 704 genes were greater than 35. There were 411 genes (56.53%) with ENC values between 35 and 50 and weak preference, and 293 genes (40.3%) with ENC values greater than 50 and weak preference. The average ENC value of ten Rutaceae plant species was about 48.17. Altogether, the analysis of ENC value or CAI value indicated that Rutaceae plants had weak codon usage bias.

**Table 3:** Summary table of ENC of ten Medicinal species of Rutaceae

Species	CAI	ENC	Gene number		
			ENC < 35	35 ≤ ENC ≤ 50	50 < ENC
*C. reticulata*	0.17	51.00	2	38	34
*C. trifoliata*	0.17	51.15	2	38	33
*P. amurense*	0.17	50.83	2	44	27
*P. chinense*	0.17	50.82	2	44	27
*R. graveolens*	0.17	51.46	2	41	30
*T. asiatica*	0.17	50.93	2	43	28
*T. ruticarpum*	0.17	50.89	2	41	29
*Z. armatum*	0.17	50.95	3	40	29
*Z. schinifolium*	0.17	50.86	3	41	28
*Z.bungeanum*	0.17	50.93	3	41	28

Note: CAI: codon adaptation index, ENC: effective number of codons

#### Differential analysis of synonymous codon usage

**Fig. 2:**
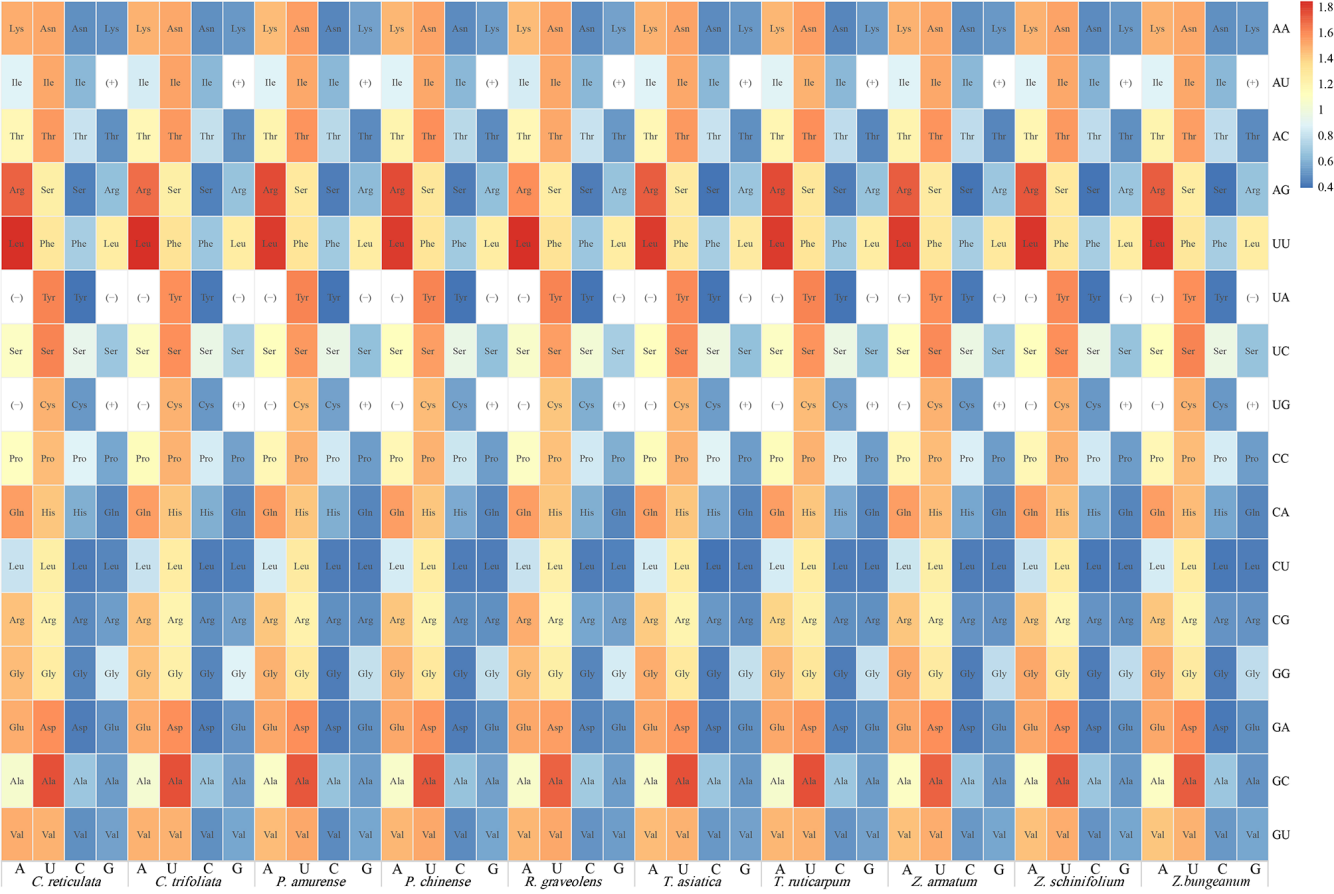
Heat map of relative synonymous codon usage (RSCU) values of ten Rutaceae species. The vertical axis represents the first two bases of the codons, and the horizontal axis represents the third base corresponding to each species. (-) indicates stop codons (UAA, UAG, and UGA). (+) indicates nonsynonymous codons (AUG and UGG)

For differential analysis, three stop codons (UAA, UAG, and UGA) and two non-synonymous codons (AUG and UGG) were removed, and a heat map was drawn. A lotal of 59 synonymous codons were mapped in the analysis (Fig. 2). The RSCU value of UUA encoding Leu was the highest, followed by GCU encoding Ala. The distribution of RSCU values in the chloroplast genome codons of ten Rutaceae plant species coincided with each other. Each species had 29 synonymous codons (RSCU > 1), most of which ended with U (16) or A (12). One of them ended with G, while no synonymous codons ended with C. Further, the A/U base had a preference for the third position. These results indicate that the codon usage patterns of the chloroplast genomes of the Rutaceae plant family members are similar.

**Fig. 3:**
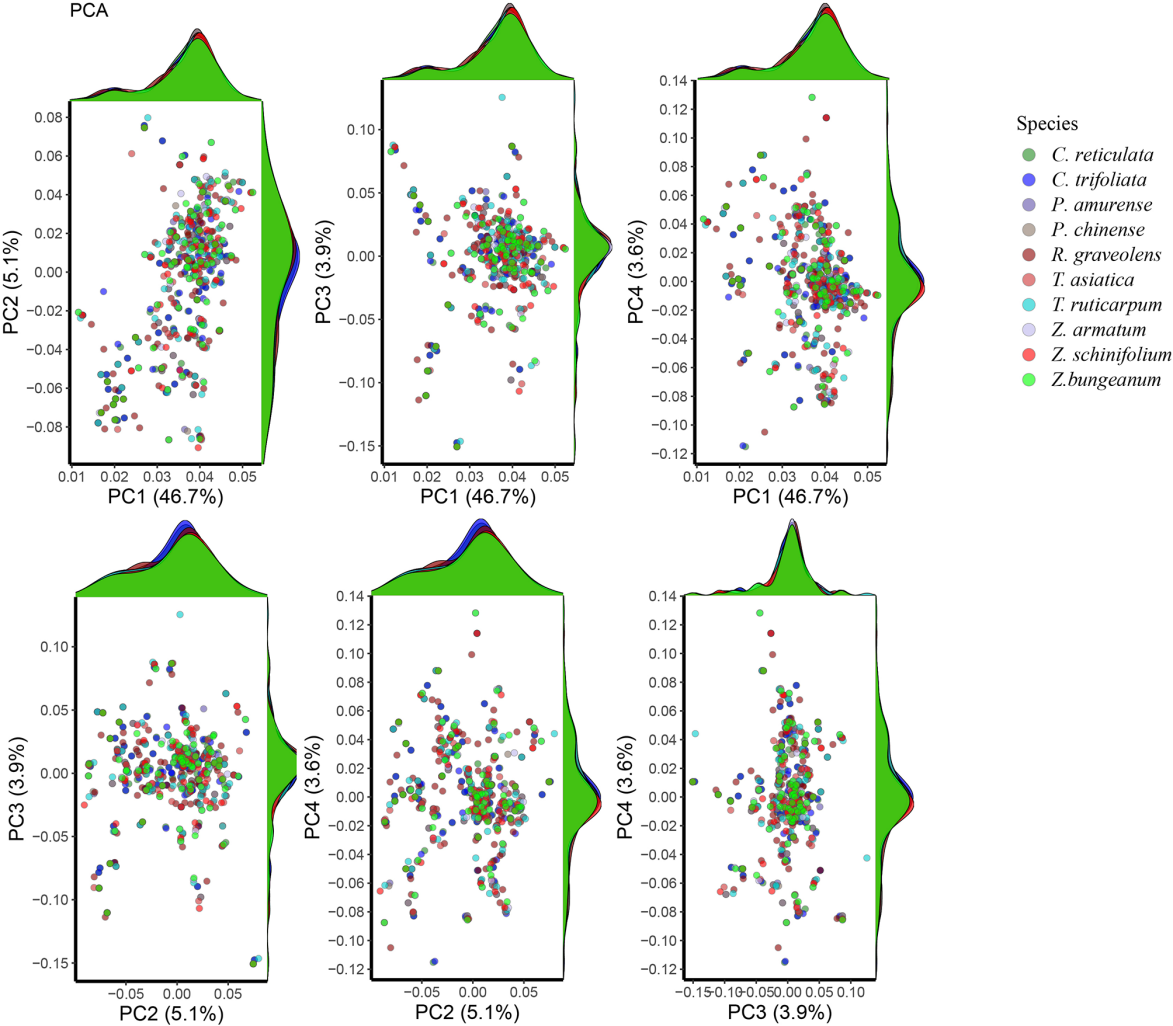
The first principal component accounts for the variability in the data, where each succeeding component accounts for the remaining variability. Three stop codons (UAA, UAG, and UGA) and two nonsynonymous codons (AUG and UGG) were removed. We showed the first four latitudes, which account for 46.7% (PC1), 5.1% (PC2), 3.9% (PC3), and 3.6% (PC4) of the codon variation information, respectively

Principal component analysis (PCA) can compress high-dimensional information into a two-dimensional map, visualizing differences in codon preferences among organisms. To further observe the codon usage differences among the ten Rutaceae species, we used the codon usage frequency calculation tool provided by the Shenzhen Kejie Industrial Development Co., Ltd platform (https://www.antiby.com/sms2/codon_usage.html) was used, which calculated the 64 codon usage frequencies for each CDS of the ten species. Based on the codon usage frequency of 59 synonymous codons, PCA was performed (Fig. 3). The results showed that the synonymous codons of ten Rutaceae species were overlapped, indicating that the codon usage frequencies were similar. Therefore, it can be proposed that the codon usage patterns of ten Rutaceae chloroplast genes were similar.

#### Identification of optimal codons

**Fig. 4:**
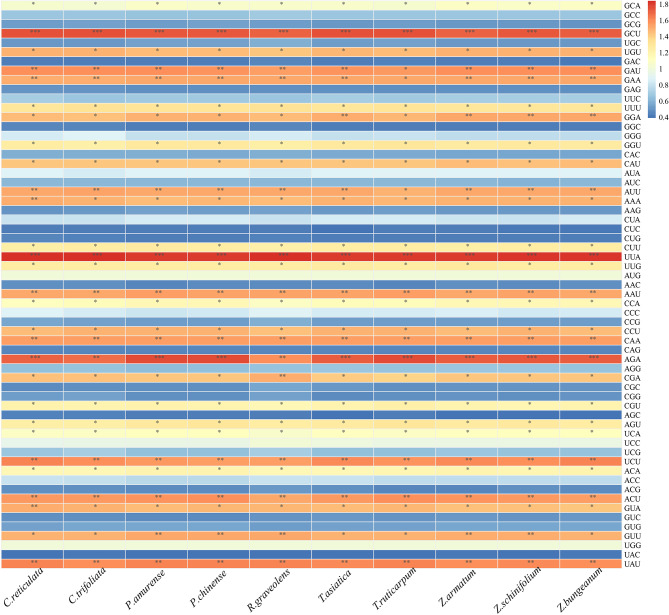
Map of optimal codon analysis. RSCU value greater than 1 refers to the optimal codon. * indicates 1 < RSCU < 1.5; ** indicates 1.5 ≤ RSCU < 1.7; *** indicates 1.7 ≤ RSCU

We observed that each sample had 30 optimal codons (RSCU > 1) (Fig. 4). The optimal codons of ten species were the same. The RSCU values ​​of GCU encoding Ala, UUA encoding Leu, and AGA encoding Arg were more than 1.7, indicating that the usage frequency of GCU, UUA, and AGA in Rutaceae plant species was higher than the other codons. Most of the 30 optimal codons contained A and U at the end position. These observations can provide helpful information for the genetic transformation of Rutaceae plants in the future.

### Analysis of factors for codon usage bias in Rutaceae plant family

#### Correspondence analysis

**Fig. 5:**
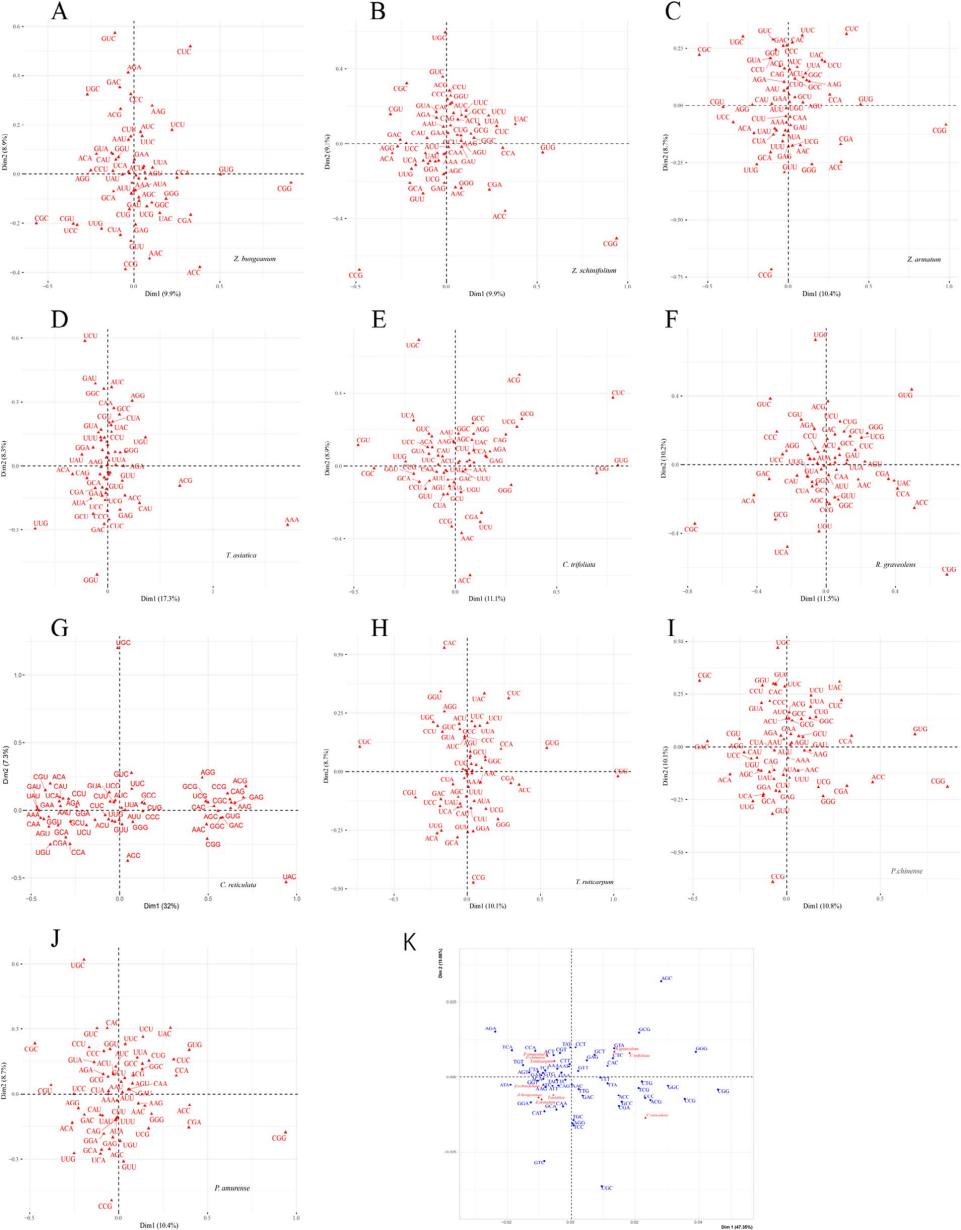
A map depicting the codon usage space. The conversion process of 59 synonymous codon usage frequencies for each species to a smaller number with correspondence analysis. Dim1 explains the variability in the data, and each subsequent Dim explains the remaining variability. To reduce the effect of amino acid composition on codon usage, each dimension corresponds to the codon usage frequency for each corresponding codon. We removed the codons ATG encoding Met, TGG encoding Trp, and the three-stop codons TAA, TAG, and TGA. (**A**). *Z.bungeanum*, (**B**). *Z.schinifolium*, (**C**). *Z.armatum*, (**D**). *T.asiatica*, (**E**). *C.trifoliata*, (**F**). *R.graveolens*, (**G**). *C.reticulata*, (**H**). *T.ruticarpum*, (**I**). *P.chinense*, (**J**). *P.amurense*, (**K**). Codon usage space map

To determine the differences in synonymous codon usage in chloroplast genomes of ten Rutaceae species, we performed Correspondence analysis (COA) based on the RSCU value, and a codon usage space map was constructed to illustrate the variation of codon usage in different genes (Fig. 5). We observed that the contribution rate of Dim1 in *C. reticulata* was 32%, and the synonymous codons were majorly distributed along Dim1 (Fig. 5G). The synonymous codons at the end of G/C and A/U were almost separated by Dim2. Interestingly, there is a unique phenomenon that most of the synonymous codons at the end of G/C are gathered at the far right of Dim2, and those at the end of A/U are almost on the left of the Dim2 side. Axis one and two of the remaining nine species (*C. trifoliata, P. amurense, P. chinense, R. graveolens, T. asiatica, T. ruticarpum, Z. armatum, Z. schinifolium, Z.bungeanum*) accounted for 9.9–17.3% and 8.3–10.2% of the synonymous codon usage variation (Fig. 5A, F, H and J), respectively. These axes were majorly distributed along Dim1 and Dim2. Codons were found to be distributed in four regions, most of which occurred near the central axis. We mapped the codon usage space (Fig. 5k) of ten species and observed that all ten species were affected by A/U-ending codons. Therefore, we speculated that the base composition at the third position of codons affects the codon usage pattern in ten Rutaceae species, wherein the codon usage of *C. reticulata* is most affected by the third base composition.

#### ENC vs. GC3 analyses

**Fig. 6:**
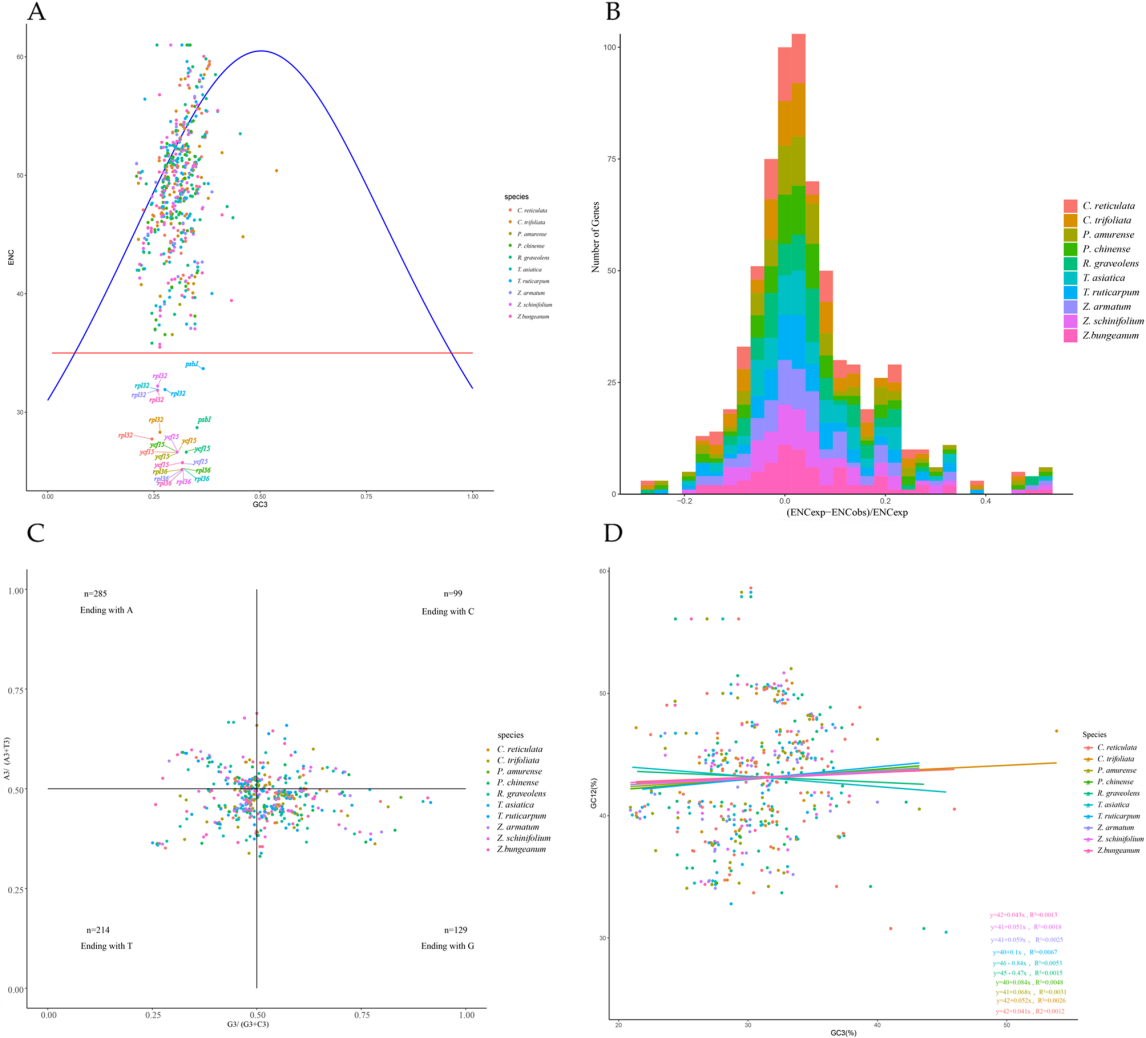
Analysis of factors for codon usage bias in Rutaceae plant family. (**A**). ENC plot. The red line represents ENC = 35, and the blue line represents the expected curve. The point below the red line indicates a strong codon bias, while the point above the red line indicates a weak codon bias. The point on or close to the curve means that GC3s is the only factor affecting codon bias, whereas the point below the curve means that natural selection is the determining factor. (**B**). Distribution of ENC ratio in ten Rutaceae species. Note: the horizontal axis is the value of the ENC ratio ((ENCexp - ENCobs)/ENCexp); the vertical axis indicates the number of genes. (**C**). RP2-plot. When mutation pressure alone influences the CUB of genes, the frequency of nucleotides A and T is expected to be equal to C and G at the third position in the codon. Conversely, natural selection would not permit A and T bases to be used equally with G and C nucleotides. (**D**). GC3 vs. GC12 plot analysis

The ENC vs. GC3s plot analysis is an efficient way to describe codon usage patterns and is feasible in detecting the effect of GC3s on codon bias. Here, we performed ENC-plot analysis on the chloroplast genomes of ten species of the Rutaceae family (Fig. 6A). Figure 6A depicts that the codon bias of the chloroplast genome is weak. However, we observed that each species has two to three genes (falling below the red line) with strong codon bias. For example, *rpl32, ycf15, rpl36* in three species of *Zanthoxylum*; and *ycf15, psnl* in *R. graveolens*, etc. Figure 6A revealed that more genes fall below the expected curve than on or above the expected curve in each species. It shows that the codon preference of chloroplast genes in ten species is affected by the base composition at the third position (mutation pressure) and natural selection, although the impact of natural selection is more significant than that of mutation pressure.

To explore a more precise relationship between expected ENC ​​(ENCexp) and observed ENC values, the ENC ratio and their distribution were plotted (Fig. 6B). The calculation of ENC ratio by “(ENCexp - ENCobs)/ENCexp” revealed that the ENC ratio of most genes was between − 0.15 and 0.15, indicating that the ENC values of most genes were close to the ENCexp value. The ENC ratios of about 63% of the genes were greater than 0, indicating that the actual ENC values ​​of most genes were lower than the expected ENC values.

#### RP2-plot and neutrality plot analyses

We analyzed the effect of mutation and selection pressure on gene codon usage bias using PR2 plots and found that genes from ten Rutaceae chloroplast genomes were unevenly distributed in the four areas and mainly distributed in the G3/(G3 + C3) < 0.5 and A3/(A3 + T3) > 0.5 regions (Fig. 6C). Overall, the third base of the codons in the chloroplast genome was biased towards A/T (U), and the base usage frequency at the third position of the codon was not equal for A, T, C, and G. Except for a few genes located near the center, other genes were scattered away from the central axis. These results suggested that natural selection and mutation pressure may affect the codon bias in the chloroplast genomes of ten Rutaceae species.

We calculated the GC content of the codons for each gene at different positions and observed the distribution of all genes on the GC3 and GC12 scatter plots (Fig. 6D). The GC12 values ​​of the chloroplast genes in the ten Rutaceae plants were between 30.47 and 58.63%. Further, the values ​​of GC3 were between 20.91% and 53.85%, indicating that the base usage frequency at the third position for G/C was lower than A/T. The correlation coefficients R2 of GC12 and GC3 were more significant than 0, and GC12 and GC3 were positively correlated. However, there was no significant correlation observed at the level of *P* > 0.05, indicating that the base composition at the 1st, 2nd, and 3rd positions of the chloroplast genes was quite different. The regression coefficient (the slope of the fitting curve between GC12 and GC3) was between − 0.84 and 0.1, suggesting that the GC composition has relatively less effect on the codon usage preferences, and the natural selection primarily affects the genomic codon bias of ten *Rutaceae plant* chloroplasts.

### Effect of gene expression level, GC content, and CDSs size on codon bias

**Fig. 7:**
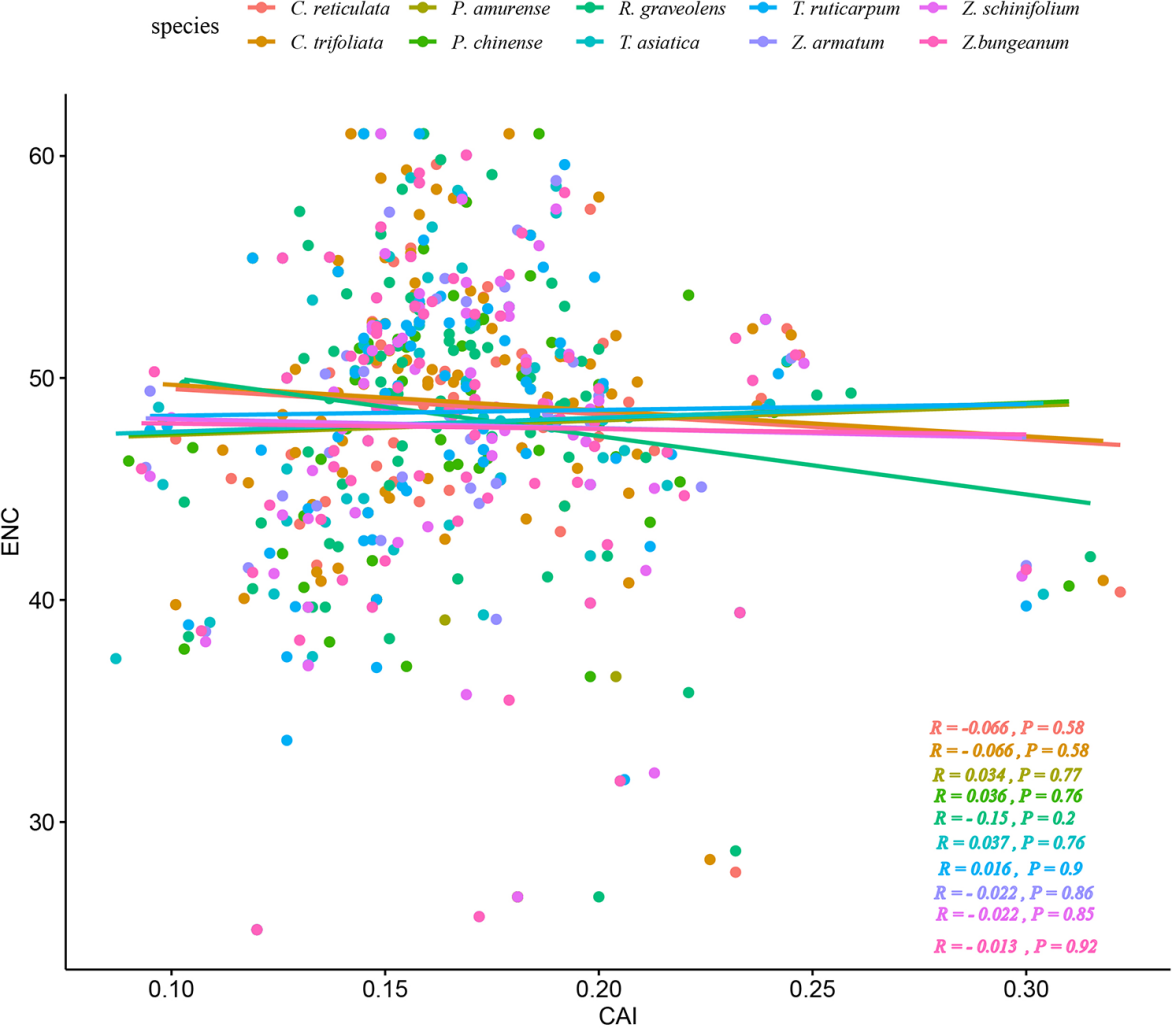
The plot of ENC vs. gene expression level in ten species of the Rutaceae family. Note: the horizontal axis represents the CAI values, while the vertical axis represents the ENC values

Codon adaptive index (CAI) was calculated to evaluate the gene expression levels. The correlation between CAI and ENC analyzed and plotted, evaluating the effect of gene expression on codon usage bias in the Rutaceae plant family (Fig. 7). Figure 7 shows that except for *R. graveolens*, the absolute value of the correlation coefficient R of all species of the Rutaceae family was less than 0.1 (*p* > 0.05). Therefore, we speculated that the codon usage bias in the Rutaceae family has an insignificant relationship with the gene expression level.

Furthermore, the correlation between GC content, size of CDSs, and ENC was analyzed (Fig. 1). The results showed that the size of CDS in *R. graveolens* was positively correlated with ENC (*P* > 0.05), and the correlation coefficient was small (*R* = 0.238). The correlation between CDS and ENC of the other nine species was not significant. Three *Zanthoxylum* GCalls were correlated with ENC at the *p* > 0.05 level, and the correlation coefficients were in the range of 0.250–0.56. Altogether, the correlations between GCalls of the other nine species and ENC were insignificant, suggesting that CDS size and GC content have negligible effects on codon usage.

## Discussion

Codon bias has a profound impact on heterologous protein expression. Studies have revealed that the codon preference in the prokaryotic cells is positively correlated with the corresponding tRNA concentration, which helps to maintain a balance between the codon content and the homologous tRNA concentration. It is known that the tRNA concentration directly affects the expression level of foreign genes [26, 48, 49]. Replacing rare codons with optimal codons can increase the expression of foreign genes in heterologous systems [48]. The analysis of codon usage patterns in the organisms, and the study of optimal codons are of great importance to facilitate the heterologous expression of exogenous genes [50]. In the current study, we analyzed the codon usage bias in the chloroplast genomes of ten Rutaceae plant species.

Codon usage is primarily measured by the evaluation of ENC in an organism. When synonymous codons encode the amino acids, the ENC value is 61, suggesting the absence of preference for the codon. When each amino acid is encoded by only one codon, the ENC value is 20 [33]. In our analysis, more than 90% of genes were found to have ENC values ​​above 35. In addition, more than 40% of genes had ENC values ​​greater than 50, suggesting that the codon bias of most genes in Rutaceae chloroplast genes is relatively weak. Correspondence analysis (COA) uses a multivariate statistical approach to identify the source of variation in the usage of a synonymous codon in genes. A study of codon usage bias in peony and honeysuckle revealed that genes with different GC content segregate along the first axis, with mutations being the determining factor [51, 52]. In the current study, similar features were observed during COA analysis, where the mutation was shown to have a more significant impact on codon usage in *C. reticulata* than in the other nine Rutaceae species. In addition, we found that *C. reticulata* codons were restricted by the fact that the base composition has a greater influence (Fig. 5G and K). Figure 5K depicts that the codon usage pattern in *C. reticulata* is different from that of the other nine Rutaceae plants. However, from additional analyses, the overall codon usage patterns of ten species were found to be similar. We speculate that the reason why *C. reticulata* is greatly affected by mutations may be that the origin of the species or *C. reticulata* apomixis [53] causes its codon usage to be gradually affected by base mutations.

Codon usage analysis has important implications in several ways. Heterologous gene expression is often used as an essential approach for biotechnological manipulation while producing antibodies, vaccines, or transgenic plants. Understanding the codon usage characteristics of the host can help in improving the expression efficiency of foreign genes and further increase the yield of desired products [54–56]. In our study, 30 optimal codons were identified from the chloroplast genome of Rutaceae family plants, which would help in the breeding of *Z. bungeanum*, citrus, etc., with beneficial traits. Of these, the following 12 codons (*R* ≥ 1.50) were selected as preferred and high-frequency codons: UAA, GCU, GAU, GAA, AUU, UUA, AAU, CAA, AGA, UCU, ACU, and UAU. These results would help in the breeding process of peppers, citrus, etc., with beneficial traits.

Regarding the origin of codon usage traits, our ENC plot, RP2 plot, and Neutrality plot analyses showed that mutational pressure and natural selection influenced the codon usage preferences in ten Rutaceae chloroplast genomes. Our results are consistent with the previous results obtained in *Euphor biaceae* [29], Theaceae plant family [31], and *Oryza* [57]. In addition, we observed that two to three genes had strong codon usage bias in the ENC plot, such as *rpl32*, *ycf15*, and *rpl36*, whereas the other genes had relatively weak codon bias.

A few researchers infer that codon bias can reduce the diversity of isotactic tRNAs. Further, the content of tRNAs with some rare codons is lower, which is conducive to saving a part of the energy of an organism under rapid growth conditions, thereby reducing its metabolic load [49]. Regardless of the underlying reason, codon bias does have a profound impact on heterologous protein expression levels. In addition, the reasons for the codon usage bias caused by natural selection have not been studied yet, which requires absolute attention in future research. In codon base composition and RP2-plot analysis, we observed that Rutaceae plant species prefer codons with rich AT(U) or A/T(U) endings. This observation is similar to the codon usage patterns of *Oryza*, algae [30, 57], and *Coffea arabica* [58]. Our results indicated that the codon usage bias in the chloroplast genome of Rutaceae species was primarily affected by natural selection, followed by base mutation.

## Conclusions

In this study, the codon usage patterns and formation factors were analyzed in ten Rutaceae plant species. Analysis of GC content and ENC values ​​of the whole chloroplast genome revealed that the codon usage bias of the chloroplast genome of the Rutaceae plants was weak. Further, analysis of single genes showed that *rpl32* and *ycf15* in *C. trifoliata* and *C. reticulata*; *rpl32*, *ycf15*, and *rpl36* in three *Zanthoxylum* species; *ycf15* and *rpl36* in *P. amurense* and *P. chinense*; *psbI* and *ycf15* in *T. asiatica; rpl32* and *rpl36* in *T. asiatica*, and *rpl32* and *psbJ* in *T. ruticarpum* have strong codon bias (ENC < 35). These species have similar codon preferences ending with A/U. GC content, CDS size, and CAI exhibited insignificant correlations with the ENC values. We speculated that the nucleotide composition, CDS size, and gene expression level might play relatively weak roles in codon usage bias in the chloroplast genomes of Rutaceae plant species. In contrast, natural selection and mutation pressure might play relatively vital roles in codon usage bias. In addition, we screened 30 optimal codons for each species, most of which end with A/U, wherein 12 are the first chosen codons. These findings might play essential roles in the genetic engineering studies of plants belonging to the Rutaceae plant family.

### Electronic supplementary material

Below is the link to the electronic supplementary material.

Supplementary Material 1

## Data Availability

Upload as a supplementary file.
