# Supplementary material for: Comparative analysis of codon usage bias in chloroplast genomes of ten medicinal species of Rutaceae
Source: BMC Plant Biol. 2024 May 20;24:424. doi: 10.1186/s12870-024-04999-5 (PMC11103831; doi:10.1186/s12870-024-04999-5)
Supplement: Supplementary file 1 — Supplementary Material 1 [file 12870_2024_4999_MOESM1_ESM.docx]

**727 Coding Sequences（CDSs）**

***C. reticulata***

>psbA

ATGACTGCAATTTTAGAGAGACGCGAAAGCGAACGCCTATGGGGTCGCTTCTGTAACTGGATAACTAGCACTGAAAACCGTCTTTACATTGGTTGGTTTGGTGTTTTGATGATCCCTACTTTATTGACCGCAACTTCTGTATTTATTATCGCCTTCATTGCTGCTCCTCCAGTAGATATTGACGGTATTCGTGAACCTGTTTCTGGATCTCTACTTTATGGAAACAATATTATTTCTGGTGCGATTATTCCTACTTCTGCAGCTATAGGTTTGCATTTTTACCCGATATGGGAAGCGGCATCCGTTGATGAATGGTTATACAATGGCGGTCCTTATGAGCTAATTGTTCTACACTTCTTACTTGGTGTAGCTTGTTACATGGGTCGTGAGTGGGAACTTAGTTTCCGTCTGGGTATGCGTCCTTGGATTGCTGTTGCATATTCAGCTCCTGTTGCAGCAGCGACTGCTGTTTTCTTGATCTACCCAATCGGTCAAGGAAGTTTTTCTGATGGTATGCCTCTAGGAATCTCTGGTACTTTCAACTTCATGATTGTATTCCAGGCTGAGCACAACATCCTTATGCACCCATTCCACATGTTAGGCGTAGCTGGTGTATTCGGCGGCTCCCTATTCAGTGCTATGCATGGTTCCTTGGTAACCTCTAGTTTGATCAGGGAAACCACAGAAAATGAATCTGCTAATGCAGGTTACAGATTCGGTCAAGAGGAAGAAACTTATAATATCGTAGCTGCTCACGGTTATTTTGGCCGATTGATCTTCCAATATGCTAGTTTCAACAATTCTCGTTCTTTACATTTCTTCCTGGCTGCTTGGCCTGTAGTAGGTATCTGGTTCACTGCTTTAGGTATTAGCACTATGGCTTTCAACTTAAATGGTTTCAATTTCAACCAATCTGTAGTTGATAGTCAAGGTCGTGTAATTAATACCTGGGCTGATATTATTAATCGTGCTAACCTTGGTATGGAAGTTATGCATGAACGTAATGCTCATAACTTCCCTCTAGACCTAGCTGCTATTGAAGCCCCATCTACAAATGGGTAA

>matK

ATGGAGGAATTTCAAGTATATTTAGAACTAGATAGATCTCAACAACACGACTTCCTATACCCACTTCTTTTTCGGGAGTATATTTATGTACTTGCTCATGATCATGGTTTAAATAGCTCGATGATGTCATTGGAAGGTGGGTTTTATGACAATAAATCTAGTTCACTAAGTGTGAAACGGTTAATTACTAGAATGTATCAACGGATTAATTTGAGTATTGTTGCTAATGATTCGAATCAAAATCCGATTTTTGGGCACAACAATAAGTTATATTCTCAAATTATATCAGAGGTATTTGCTGCTGTGGTGGAAATTCCATTTTCCCTACGGTTGGTGGCTTTTTTAGAAGGGAAAGAAATTGAAAAATCGCCTAATTTCCAATCAATTCATTCAATATTTCCTTTTTTCGAGGATAAATTGTCCCATTTAAATTATGTGTTAGATGTACGAATACCCTACCCCATTTGTCCCGAAATCTTGGTTCAAACCCTTCGCGAATGGGTAAAGGATGCCTCTTCTTTACATTTATTACGGTTCTTTCTCCACGAGTATTTTAATTCGAACAGTCTTATTACTCCAAAGAACTCTATTTCTGTTTTTTTAAAAAGTAATCCAAGATTGTTATTGTTTCTATATAATTCTCATGTATATGAATATGAATCCATCCTCTTTTTTCTCTGTAACCAATCGTCTCATTTACAATCAACATCCTTTCGAGTCCTCGTTGAGCGAACGTATTTCTATGGAAAAGTCGAACATCTTGTCGAAGTCTTTGCTAAAGATTTTCAGGACATCTTAGGGTTGGTCAAGGATCCTTTCATGCATTATGTTAGATATCAAGGAAAATCCATTTTGGCTTCAAAGGATACGCCTCTTCTGATGAATAAATGGAAATATTACCTTGTCGGTTTATGGCAATGGCATTTTCACGCGTCTTCTCAACCAGGAAGGGTTCAGCTAAACCACTTATACTTAGGCAAGTACGCTATTAACTTTCTGGGCTATCTTTCCGGTGTGCGACTAAATTCTTTGTTGGTACGGAGTCAAATGCTAGAAAATTCATTTCTAATAGATAATTCTATGAAGAAGGTCGATACGACCGTTCCAATTATTCATCTGATTGGATCATTGACTAAGGCGCGGTTTTGTAACGCATTAGGGCATCCTATCAGTAAGTCGACTTGGTCCGATTTCTCTGATTCTCATCTTATCGACCGATTTGTGCGTATATGTAGAAATCTTTCTCATTATTACAGCGGATCTTCAAAAAAAAAAAGTTTGTATCGAGTAAAATATATACTTCGGCTTTCTTGTGTTAAAAGTTTGGTTCGTAAACATAAAAGTACTGTACGCGCTTTTTTACAAAGATTAGGTTCGGAATTATTGGAAGAATTCCTTATGGAGGAAGAACACGTTCTTGCTTTACTCTTTCCAAGAGCTTCGTCTACTTCGCGTAGGTTCTATTTATATAGAGGACGGATTTGGTATTTGGATATTTTTTGTATCAACGATCTGGTTAATTGTCAATGA

>rps16

ATGGTAAAACTTCGTTTGAAACGATGTGGTAGAAAGCAACGAGTCGTTTATCGAATCGTTGCAATTGATGGTCGATCCCGAAGAGAAGGAAGAGATCTTCGGAAAGTGGGTTTTTATGATCCGATAAATAATCAAACCCATTTAAATGTTCCTGCTATTCTATATTTCCTTGCCAAGGGCGCTCAACCTACAGGAACCGTTCATGATATTTCACAGAAAGCGGGGGTTTTTACAGAAGTTAGTCTTAATCAAACGAAATTCTATTAA

>psbK

ATGCTTAATATCTTTAGTTTAATGTATATCTGTCTTAATTCTGCCCTTTATTCGAGTAGTTTTTTATTCGCTAAATTACCCGAGGCCTACGCTTTTTTGAATCCAATTGTAGATGTTATGCCAGTAATACCTGTTCTATTTTTTCTCTTAGCCTTTGTTTGGCAAGCTGCTGTAAGTTTTCGATAA

>psbI

ATGCTTACTCTCAAACTCTTTGTTTACACCGTAGTGATATTCTTTGTTTCGCTCTTCATCTTCGGATTCCTGTCTAATGATCCAGGGCGTAATCCCGGACGCGAAGAATAA

>atpA

ATGGCAACAATTAAAGCCGACGAAATTAGTAATATTATCCGCGAACGTATTGAGCAATATAATAGAGAAGTAAAGATTGTAAATATTGGGACCGTACTTCAAGTAGGCGACGGCATCGCCCGTATTTATGGTCTTGATGAAGTAATGGCAGGTGAATTAGTAGAATTTGAAGAGGGTACAATAGGCATTGCTCTGAATTTGGAATCAACTAATGTTGGTGTTGTTTTAATGGGTGACGGTTTAATGATACAAGAAGGAAGTTCTGTAAAAGCAACCGGAAAAATTGCTCAGATACCAGTAAGCGAGGCTTATTTAGGTCGTGTTATAAATGCCCTGGCTAAACCTATTGATGGTCGAGGTGAAATTTCAGCTTCTGAATCTCGATTAATTGAATCCCCTGCTCCGGGTATTATTTCGAGACGTTCCGTATATGAGCCTCTTCAAACAGGACTTATTGCTATTGATTCGATGATCCCTATCGGACGCGGTCAGCGAGAATTAATTATTGGGGACAGACAGACCGGTAAAACGGCAGTAGCCACGGATACGATTCTCAACCAACAAGGGCAAAATGTAATATGTGTTTATGTAGCTATTGGTCAAAAAGCATCTTCTGTGGCTCAGGTAGTGAATACTTTCCAGGAGAGGGGGGCAATGGACTACACTATTGTGGTAGCCGAAACGGCGGATTCCCCCGCTACGTTACAATACCTCGCTCCTTATACGGGCGCAGCTCTAGCTGAATATTTTATGTACCGTGAACGACACACTTTAATCATTTATGATGATCCCTCCAAACAAGCACAGGCTTATCGACAAATGTCTCTTCTATTACGAAGACCACCGGGTCGCGAAGCTTATCCCGGAGATGTTTTTTATTTGCATTCACGACTTTTGGAAAGAGCCGCGAAATTAGGTTCGCAGTTAGGCGAAGGAAGTATGACCGCTTTACCAATAGTTGAGACCCAGTCGGGAGATGTTTCGGCTTATATTCCTACTAATGTAATTTCCATTACAGACGGGCAAATATTCTTATCCGCGGATTTATTCAATGCTGGAATCAGACCCGCTATTAATGTGGGTATTTCCGTCTCTCGAGTAGGATCCGCAGCTCAAATTAAAGCTATGAAACAAGTAGCCGGCAAATTAAAATTGGAATTGGCACAATTCGCAGAATTAGAAGCCTTTGCACAATTTGCTTCTGATCTCGATAAAGCTACTCAGAACCAATTGGCAAGGGGTCAACGCTTACGTGAGTTGCTCAAACAGGCCCAAGCAGCCCCTCTCACGGTGGAAGAACAGATAATGACTATTTATACAGGAACGAATGGTTATCTTGATTCATTAGAAATTGGTCAAGTAAGAAAATTTCTCGTTGAGTTACGTACTTACTTAAAAACGAATAAACCTCAGTTCCAAGAAATAATATCTTCTACCAAGATATTCACCGAAGAAGCAGAAGCCCTTTTGCAAGAAGCTATTCAGGAACAAAAGGAACGTTTTCTACTTCAGGAACAATTATAA

>atpF

ATGAAAAATGTAACCGATTCTTTCGTTTCTTTGGTTAACTGGCCATTCGCCGGGAGTTTCGGGCTTAATACCGATATTTTAGCAACAAATCCAATAAATCTAAGTGTAGTGCTTGGTGTATTGATCTTTTTTGGAAAGGGAGTGTTAAGTGATTTATTAGATAATCGCAAACTGAGGATTTTGAATAGTATTCGAAATTCAGAAGAACTGCAGAGAGGGGCCATTGAACGGCTGGAAAAAGCCCGGGCCCGGTTACGAAAAATAGAAATAGAAGCAGATCAGTTTCGAGTGAATGGATACTCTGAGATAGAACGAGAAAAATTCAATTTGATTAATTCAACTTATAAGACTTTGGATCAATTAGAAAATTACAAAAATGAAACCATTCATTTTGAACAACAAAGAGCAATTAATCAAGTCCGACAACGGGTTTTCCAACAAGCTTTACAAGGAGCGCTCGGAACTCTGAATAGTTGTTTGAACAAGGAGTTACATTTACGTACCATTAGTGCCAATATTGGCATGTTTGGAGCGATGAAAGAAATAACGGATTAG

>atpH

ATGAATCCACTGATTTCTGCCGCTTCCGTTATTGCTGCTGGGTTGGCTGTTGGGCTTGCTTCTATTGGACCTGGAGTTGGTCAAGGTACTGCTGCGGGCCAGGCAGTAGAAGGGATCGCGAGACAGCCCGAGGCGGAGGGAAAAATACGAGGTACTTTATTGCTTAGTCTGGCTTTTATGGAAGCTTTAACAATTTATGGGCTGGTTGTAGCATTAGCACTTTTATTTGCGAATCCTTTTGTTTAA

>atpI

ATGAATGTTCTATCATGTTCCATGAATACACTAAGGGGGTTATACGATATATCCGGTGTGGAAGTAGGCCAACATTTCTATTGGCAAATAGGTGGGTTCCAGGTCCATGCCCAAGTACTTATTACTTCTTGGGTTGTAATTGCTATCTTATTAGGTTCAGCCTTTATAGCCGTTCGGAATCCACAAACCGTTCCGACTGCCACTCAAAATTTCTTCGAATATGTCCTTGAATTCATTCGAGACGTGAGCAAAACTCAGATTGGAGAAGAATATGGCCCATGGGTTCCCTTTATTGGAACTCTGTTTCTTTTTATTTTTGTTTCTAATTGGTCAGGTGCTCTTTTACCTTGGAAAATCATAGAGTTACCTCATGGGGAGTTAGCCGCACCCACGAATGATATAAATACTACCGTTGCTTTAGCTTTGCTCACGTCAATAGCATACTTCTATGCGGGTCTTTCCAAAAAGGGATTAGGCTATTTCAGTAAATACATTCAACCGACTCCAATTCTTTTACCCATTAACATTTTAGAAGATTTCACAAAACCTCTATCACTTAGTTTTCGACTTTTTGGGAATATATTAGCCGATGAATTAGTAGTTGTTGTTCTTGTTTCTTTAGTACCTTTAGTGGTTCCTATACCCGTCATGTTCCTTGGATTATTTACAAGCGGTATTCAAGCTCTTATTTTTGCAACTTTAGCTGCGGCTTATATAGGCGAATCTATGGAGGGACATCATTGA

>rps2

ATGGCAAGAAGGTATTGGAACATCCATTTGGAAGAGATGATGGAAGCAGGAATTCATTTTGGTCATGGTACTCGGAAATGGAATCCTAGAATGGCACCTTATATATCTGCAAAACATAAAGGTATTCATATTACAAATCTGACTCGAACTGCTCGGTTTTTATCAGAAGCTTGTGATTTAGTTTTTGATGCAGCAAGTAGGGGAAAACAATTCTTAATTGTTGGTACTAAAAATAAAGCAGCTGATTTAGTCGCGCGAGCTGCAATAAGGGCTCGGTGCCATTATGTTAATCAAAAATGGCTCGGCGGTATGTTAACCAATTGGTCCACTACAGAAACGCGACTTCACAAGTTCAGGGATTTGAGAACGGAACAAAAAGGGGGGAGACTCGACAGTCTTCCCAAAAGGGATGCCGCTATTTTGAAGAGACAATTATCGCGTCTGCAAACGTATCTGGGCGGAATTAAATATATGACGAGGGTACCCGATATTGTAATCGTCGTTGATCAGCAAGAAGAATATACGGCTCTTCGAGAATGTATCACTTTGGGAATTTCAACAATTTGTTTAATCGATACAAATTGTGACCCCGATCTCGCAGATATTTCGATTCCAGCAAACGATGACGCTATAGCCTCAATCCGATTAATCCTTAACAAATTAGTATTCGCAATTTGTGAGGGTCGCTCTAGCTATATACGAAATCCTTGA

>rpoC2

ATGGCAGAACGGGTGAGTCTGGTCTTTCACAATAAAATGATAGATGGAACTGCCATTAAACGACTTATTAGCAGGTTAATAGATCACTTCGGAATGGCATATACATCCCACATCCTGGATCAAGTAAAGACCCTGGGGTTCCAGCAAGCCACTGCTACATCTATTTCATTAGGCATTGATGATCTTTTAACGATACCTTCTAAGCGATGGCTAGTCCAAGATGCTGAACAACAAAGTTTTATTTTGGAAAAACACTATCATTATGGGAATGTACACGCGATAGAAAAACTACGACAATCCATTGAGATATGGTATGCTACAAGTGAATATTTGCGACAAGAAATGAATCCTAATTTTAGGATGACTGAGCCTTTTAATCCAGTCCATATAATGTCTTTTTCGGGGGCTAGAGGAAATGCATCTCAAGTACACCAATTGGTGGGTATGAGAGGATTAATGTCTGATCCCCAAGGGCAAATGATTGATTTACCCATTCAAAGTAATTTACGCGAAGGGCTTTCTTTAACAGAATATATCATTTCTTGCTATGGAGCCCGCAAGGGGGTTGTCGATACCGCTGTCCGAACATCAGATGCTGGATATCTTACGCGCAGACTTGTTGAAGTAGTTCAACACATTATTGTACGTAGAACAGATTGTGGCACTGTCCGAGGAATTTCTGTGAGTCCTCAAAATCAAAATAGGATGATGTCGGAAAGGGTTTTTAGCCAAACATTAATTGGTCGTGTATTAGCGGACGATATATATATGGGCCAGCGATCCATTGCCATTAGAAATCAAGATATTGGGATTGGACTTGTCAATCGACTCATAACCCTTCGAACACAAGCAATATCTATTCGAACCCCCTTTACTTGTAGGAGTACATCGTGGATCTGTCGATTATGTTATGGTCGGAGTCCGACTCATGGTGACCTGGTTGAATTGGGGGAAGCCGTAGGTATTATTGCGGGTCAATCTATTGGAGAACCAGGGACTCAACTAACATTAAGAACTTTTCATACCGGCGGCGTATTTACGGGGGGCACTGCAGAACATGTACGAGCCCCTTCTAATGGTAAAATCAAATTCAATGAGGATTTGGTTCATCCCACGCGCACACGTCACGGGCATCCGGCTTTTCTATGTTCTATAGATTTGGATGTAATTATTGAGGGTGAAGATATTATGCACAATGTGACTATTCCACCAAAAAGTTTTCTTTTAGTTCAAAATGATCAATATGTCGAATCAGAACAAGTGATTGCTGAGATTCGGGCGGGAGCATACACTTTGAATTTTAAAGAGAGGGTTCGAAAACATATTTATTCTGATTCAGAGGGAGAAATGCACTGGAGTACTGATGTGTACCATGCACCCGAATTTACATATAGTAATGTCCACCTCTTGCCAAAAACAAGCCATTTATGGATATTGTCGGGGGGTTCACGCAGATCTAGTGTAGTTTCTTTTTCACTCCACAAGGATCAAGATCAAATGAATATTCATTCTCTTTCTGTCGAACGAAGAGAGATTTCGAGCCTCTCGCTCTCAGTGAATAATGATCAAACGGGACACAAATTTTTTAGTTCTGATTTTTCTGCTAAAAAAAAAGGTCGAATTCTTGAGATGTCTGATTATTCGGGATTTAATAGAATCATAGGTACTGGTCATTGTAATCTCATCCATCCTGCAATTCTCCACGCAAATTCGGATTTATTGGCAAAAAGGCAAAGAAATGGATTTCTTATTCCATTCCACTCGATTCAAGAGCAAGAGAAAGAGCTAATGCCCCATTCAGGTATCTCGATTGAAATACCCGTAAGGGGTATTTTCCGTAGAAATAGTATTCTTGCTTATTTCGACGATCCTCGATACAGAAGAAAGAGTTCCGGAATTACTAAATGGGGGGCTCTGGGGGCGCATTCAATCGTTAAAAAAGAGGACTTGATTGAGTATCGAGGACTCAAAAAAATTAAGCCAAAATACCAAATGAAAATAGATCGCCTTTTTTTCATTCCGGAGGAAGTGCATATTTTTCCCGAATCTTCTTACCTAATGGTACGGAATAATAGTATCATTGGAGTAGACACACAAATCACTTTAAATATACGAAGCCGGGTGGGCGGATTGGTCCGAATGGAGAGAAAAAGGGGAGGGGTTGAACTAAAAATATTTTCGGGAGATATCCATTTTCCCGGAGAGATAGATAAGATATCCCGACACAGTGGCATCCTGATACCGCCAGAAAGTGAAAAAAAAAAACTTAAGGAAGCCACTAAGGAATCAAAAAAATTGAAAAAGTGGATCTATGTTCAACGGATCACACCTACAAAGAAAAAGTCTTTTGTTTTGGTTCGACCCGTAGTCACATATGAAATAGCGAACGGTATAAATTTAGCAACACTCTTTCCCCAGGATCCGCTGCGGGAAAAGGATAATATGCAATTTCGAGTTGTCAATTATGTCCTTTATGGGAAGGGCAAAGCCGCTGGGGGAATTTCTGATACAAGTCTTCAATTAGTTCGGACGTGTTTAGTGTTGAATTGGGACCAAGACAACAAAAGTTCTTCCGTCGAAGAGGTTTGTGCTTCCTTTGTTGAAGTACGTACAAATGGTCTGATTCGAGATTTCCTAAGAATCAACTTAGTGAAATCCAATATTTCGTATCTCAGAAAAAGGGATCATCCGTCGGGTTCAGGATTAATTTCTGATAATGGTTCAGCTCGCACCAATAGCAATCCGTTTTATTCCGTGTTTGGCAAGGCAGGGGTTGAACAATCGCTTAGACAAAATCAAGGAACTATTCGTACGTTGTTGAATAAAAATAAGGAATGCCAAGTTTTGATAATTTTATCATCATCTAATTATTTTCGAATGGGTCCATTGAACGATGTAAAATATCACAATGTGATAAAACAATCAATTCCAATTAAAAAAGATTCGCTAACTCCAATTAAGACTTCGTTGGGACCCTTAGGAACATTCCTTCAAATTGCGAATTTTTATTCATTTTACTATTTAATAACTCATAATCATATCTCGGTAACTAAATATTTGAAACTTGACAATTTAAAACAGCCTTTTCAAGTACTTAAATATTATTTAATGGACGAAACCGGGGAAATTTATAATCCTGATACAGATAGTAAGATCCTTTTGAATCCATTTAATTTGAATTGGTATTTTCTCCAGCCTAATTATTGTGAGGAAATGTCCCCGATAATTAGTCTTGGGCAGTTTCTTTGTGAAAATGTACGTATAACCAAAAGGGGACCATACCTAAAATCCGGTCAAGTTTTAATTGTTCAAGTTAACTCTGTAGTAATACGATCAGCTAAGCCTTATTTGGCTACTCCTGGAGCAACTGTTCATGGGCATTATGGAGCAATCCTTTCCGAAGGGGATACATTAGTTACATTTATATATGAAAAATCGAGATCTGGTGATATAACGCAGGGTCTTCCAAAAGTAGAACAGGTGTTAGAAGTGCGTTCGCTTGATTCAATATCGATGAACCTAGAAAAGAGAGTTGAGGGTTGGAACGCGAGTATAACAAGAATTCTTGGGATTCCCTGGGGATTCTTGATTGGTGCTGAGCTAACTATAGTGCAAAGTCGTATCTCTTTGGTTAATAAGATCCAAAAGGTTTATCGATCGCAGGGGGTGCAGATCCATAATAGGCATATAGAAATTATTGTACGTCAAATAACATCAAAAGTTTTAGTTTCCGAAGATGGAATGTCTAATATTTTTTTACCCGGCGAACTGATTGGATTGTTACGAGCGGAACGAATGGGGCGCGCTTTGGAAGAAGTGATCTGTTATCGAGCTAGCTTATTGGGAATAACGAGAGCGTCTCTGAATACTCAAAGTTTTATATCCGAAGCAAGTTTTCAAGAAACCACGCGAGTTTTAGCAAAAGCTGCTCTCCGAGGTCGTATCGATTGGTTGAAAGGCCTGAAAGAAAACGTTGTTCTGGGGGGGATAATACCAGTCGGTACCGGATTCAAAGGATTAGTCCACTGTTCAAGGCAGCATAACAACATTCTTTTGGAAAGACAAAAAGGGAATTTATTCGGGGGGGAAATGAGAGATATTTTCTTACACCACAGAGAATTATTTGACTCGTGCATTTCAACAACTTTCCATGATACATCAGAGCACAATTGCTTAGAGGGTTTAATGAGTCCTAGGAGCGAATTCTTTTAA

>rpoC1

ATGATTGATCGATATAAACATCAACAACTCCGAATTGGATCAGTTTCTCCTCAACAAATACGCGCTTGGGCCAATAAAATCCTACCTAATGGAGAGATAATTGGAGAAGTGACAAAACCCTATACTTTTCATTACAAAACCAATAAACCGGAAAAAGATGGATTATTTTGTGAAAGAATTTTTGGGCCTATTAAAAGTGGAATTTGTGCTTGTGGAAATTATCGAATAATCGGAGATGAAAAAGAAGACCCGCAATTTTGTGAACAATGTGGGGTTGAATTTGTTGATTCTCGGATACGAAGATATCAAATGGGATACATAAAACTGGGATGCCCAGTAACCCACGTGTGGTATTTGAAACGTCTTCCTAGTTATATCGCGAATCTTTTAGATAAACCTCTTAAAGAATTAGAAGGCCTAGTATATTGCGATTTTTCTTTTGCTAGGCCTATAGCGAAAAAACCGACTTTTTTACGATTACGCGGTTCATTCGAATATGAAATACAATCCTGGAAATACAGCATCCCACTTTTTTTTACTACCCAAGGTTTCGATAAATTTCGTAACCGAGAAATTTCTACTGGAGCAGTTGCTATCCGGGAACAATTAGCCGATCTAGATTTGCGAATTATTCTAGATAATTCCTTGTTAGAATGGAAAGAATTAGTGGAAGAAGGGCCCGCCGGTAATGACTGGGAAGATCGAAAAATTGGACGAAGAAGGGATTTTTTGGTTAGACGCATGGAATTAGCTAAGCATTTTCTTCGAACAAATATAGAACCGGAGTGGATGGTTTTATGTCTATTACCTGTTCTTCCTCCCGAGTTGAGGCCTATCATTCAAATAGATGGAGGTAAACTAATGAGTTCAGATATTAATGAACTCTATAGAAGAGTTATCTATCGGAACAATACTCTTATCGACCTATTAACAACAAGTAGATCTACGCCAGGGGAATTAGTAATGTGTCAGGAGAAATTGGTACAAGAAGCCGTGGATACGCTTCTTGATAATGGAATCCGTGGACAGCCAATGAGGGATGGTCATAATAAGATTTATAAGTCGTTTTCAGATGTAATTGAAGGAAAAGAGGGAAGATTTCGTGAGACTCTGCTTGGCAAACGGGTCGATTATTCGGGGCGGTCTGTCATTGTTGTGGGCCCCTCACTTTCATTACATCAATGTGGATTGCCTCGCGAAATCGCAATAGAGCTTTTCCAGACTTTTGTAATTTGTGGGCTAATTAGACAACACCTTGCTTCGAACATAGGAGTTGCTAAGAGTAAAATTCGGGAAAAAGGGCCGATTATCTGGGAAATACTGCAGGAAGTTATGCAGGGACATCCAGTATTGCTGAATAGAGCGCCTACTCTGCATAGATTGGGCGTACAGGCATTCCAGCCCATTTTAGTGGAAGGGCGCGCTATTTGTTTACATCCATTAGTTTGTAAGGGATTCAACGCAGACTTTGATGGGGATCAAATGGCTGTTCATGTACCCTTATCTTTAGAGGCTCAAGCAGAGGCTCGTTTACTTATGTTTTCTCATATGAATCTCTTGTCTCCTACTATTGGAGATCCCATTTCCATACCGACTCAAGATATGCTTATTGGGCTCTATGTATTAACGAGCGGGAATCGTCGAGGTATTTGTGCAAATAGGTATAATACATGGAATCGAAGAAATTATCCAGATGAAAGAATTGACGATAATAGCTATAAGTATACGAAAGAACCCCTTTTTTGTAATTCCTATGATGCAATTGGAGCTTATCGGCAGAAAAGAATCAATTTAGATAGTCCGTTGTGGCTTCGGTGGCGATTAGATCAACGCCTTATTGCTTCAAGGGAAGCTCCCATCGAAGTTCACTATGAATCTTTGGGTACCTCTCATGAGATTTATGGGCATTATCTAATAGTACGAAGTGTAAAAAAAGAAATTCTTTCTATATACATTCGAACCACCGTGGGCCATATTTCTCTTTATCGAGAAATCGAAGAAGCTATACAAGGGTTTTGCCGGGCCTGCTCATATGGTACCTAA

>rpoB

ATGCTCGGAGATGGAAATGCAGGAATGTCTACAATACCTGGGTTGAATCAGATACAATTTGAAGGATTTTGTAGGTTCATTGATCAGGGCTTAACAGAAGAGCTTTATAAGTTTCCAAAAATTGAAGATACAGATCAAGAAATTGAATTTCAATTATTTGTGGAAACATATCAATTGGTAGAACCCTTGCTAAAAGAAAGAGATGCTGTATATGAATCATTCACGTATTCTTCTGAATTATATGTATCCGCAGGATTAATTTGGAAAAGCCGAGGGGACATGCAGGAACAAACTATTTTTATTGGAAACATTCCTCTAATGAATTCTTTGGGAACTTCTATAGTAAACGGAATATACAGAATTGTCATCAATCAAATATTGCAAAGTCCCGGTATCTATTATCGGTCAGAATTGGGCCATAACGGAATTTCGGTCTATACAGGCACCATAATATCTGATTGGGGGGGAAGATTCGAATTAGAGATTGATAGAAAAGCAAGGATATGGGCTCGTGTGAGTAGGAAACAGAAAGTATCTATTCTAGTTCTATCAGCAGCTATGGGTTCGAATCTACGAGAAATTCTAGAGAATATTTGCTACCCTGAAATTTTCTTGTCTTTCCTGACGAATAAGGAGAAAAAAAAAATTGGATCAAAAGAAAATGCCATTTTGGAGTTTTATCAACAATTTGCTTGTGTAGGCGGAGATCCGGTATTTTCGGAATCCTTGTGTAAGGAATTACAAAAGAAATTTTTTCACCAAAGATGTGAATTAGGAAAGATTGGTAGACGAAATATGAACCAGAGACTGAATCTGAATATACCTCAGAACAATACATTTTTGTTACCACGAGATGTATTGGCAGCTGTCGATCATTTGATTGGACTGAAATTTGGAATGGGTACACTTGACGATATGAATCATTTGAAAAATAAACGTATTCGGTCTGTCGCGAATCTTTTACAAGATCAATTCGGATTGGCCCTGGTTCGGTTAGAAAATGTGATTAGAGGAACTATATGTGGAGCAATTCGGCATAAATTGATGCCAACTCCTCAAAATTTGGTAACTTCAACTCCCTTAACAACCACTTATGATTCTTTTTTCGGATTACATCCATTATCTCAAGTTTTGGATCGAACTAATCCATTGACACAAATAGTTCATGGGAGAAAATTGAGTTATTTGGGCCCCGGAGGATTGACAGGGCGAACTGCGAGTTTTCGGGTACGAGATATCCATCCTAGTCACTATGGCCGCATTTGTCCAATTGACACGTCCGAAGGAATCAATGTTGGACTTATTGGATCCTTAGCAATTCATGCGAGAATTGGTTATTGGGGGTCTCTAGAAAGCCCGTTTTATGAAATCTTTGAAAAATCAAAAAAAGTACGGATGCTTTATTTATCACCAAGTAGAGATGAATACTATATGGTAGCGGCAGGAAATTCTTTGGCACTGAATCAGGGTAGTCCGGAAGAACAGGTTGTTCCGACTCGATACCGTCAAGAGTTCCTGACTATTGCGTGGGAACAGGTTCATCTTCGAAGTATTTTTCCCTTCCAATATTTTTCTATTGGGGCTTCCCTCATTCCTTTTATCGAGCATAATGATGCGAATCGGGCTTTAATGAGTTCTAATATGCAACGCCAAGCAGTTCCGCTCGTTCGGTCCGAGAAGTGCATTGTTGGAACTGGGTTGGAACCTCAAGTGGCTCTAGATTCAGGGGTTCCCGCTATAGCCGAACACGAGGGAAAGATCATTTATACCGATATTGACAAGATTGTTTTATCGGGAAACGGGAATACATATTGTATTCCATTAATTATGTATCAACGTTCAAACAAAAATACTTGTATGCATCAAAAACCCCAGGTTGGGCGGGGTAAATGCATTAAAAAGGGCCAAGTTTTAGCGGATGGTGCCGCTACAGTTGGCGGCGAACTCGCTTTAGGGAAAAACATATTAGTAGCTTATATGCCATGGGAAGGCTACAATTTTGAGGATGCGGTACTTATTAGCGAACGTCTGATATATAGAGATATTTATACTTCTTTTCACATACGGAAATACGAAATTCAGACTCATGTGACAAGTCAAGGTCCCGAAAGGATCACTAATGAAATACCACATCTAGAAGCCCGGTTACTCCGCAATTTAGACAAAAATGGAATTGTGATGCTGGGATCTTGGGTAGAGACCGGCGATATTTTAGTAGGTAAATTAACGCCTCAGGCGGCGAAAGAATCATCGTATGCTCCGGAAGATAGATTATTACGGGCCATACTTGGCATTCAGGTATCCACTTCAAAGGAAACTTGCCTAAAACTACCTATAGGTGGTAGGGGTCGCGTTATTGATGTGAGATGGGTCCAGAAAAAGGGGGGTTCTAGTTATAATCCAGAAACGATTTGTGTATATATTTCACAGAAACGTGAAATCAAAGTAGGTGATAAAGTAGCTGGAAGACATGGAAATAAGGGTATCGTTTCAAAAATTTTGCCTAGACAGGATATGCCTTATTTGCAAGATGGAAGGCCTGTTGATATGGTTTTCAACCCACTAGGAGTACCCTCGCGAATGAATGTAGGACAGATATTTGAATGCTCGCTCGGGTTAGCGGGGGGTCTGCTAAATCGACATTATCGAATAGCACCTTTTGATGAGAGATATGAACAAGAGGCTTCGAGAAAACTCGTGTTTTCTGAATTATATGAAGCCAGTAAGCAAACCGCGAATCCATGGGTATTTGAGCCGGAATACCCGGGAAAAAGCAGAATATTTGATGGACGAACGGGAGATCCTTTTGAAGAACCTGTTCTAATAGGAAAGCCTTATATCTTGAAATTAATTCATCAAGTTGATGATAAAGTACACGGACGTTCCAGTGGGCATTATGCACTTGTTACCCAACAACCCCTTAGAGGAAGGTCCAAACAAGGGGGACAACGGGTAGGAGAAATGGAGGTTTGGGCTCTAGAGGGCTTTGGTGTTGCTCATATTTTACAAGAGATGCTTACTTATAAATCTGATCATATTAGAGCGCGCCAAGAAGTACTTGGTACTACAATCATTGGAGAAACAATACCTAACCCGGAAGATGCTCCAGAATCTTTTCGATTGCTCGTTCGAGAACTACGATCTTTGGCTTTGGAACTGAATCATTTTCTTGTATCTGAGAAAAACTTCCAGATTAATAAGAAGGAAGCTTAA

>psbM

ATGGAAGTAAATATTCTCGCATTTATTGCTACTACACTGTTCGTTCTAGTTCCTACTGCGTTTTTGCTTATAATATACGTAAAAACGGTCAGTCAAAGTGATTAA

>psbD

ATGACTATAGCCCTTGGTAAATTTACCAAAGAGGAAAAGGATTTATTTGATATTATGGATGACTGGTTACGGAGGGACCGATTCGTTTTTGTAGGTTGGTCTGGCCTATTGCTCTTTCCTTGTGCCTATTTCGCTTTAGGGGGTTGGTTCACAGGTACAACCTTTGTAACTTCATGGTATACCCATGGATTAGCCAGTTCCTATTTGGAAGGCTGCAACTTCTTAACCGCCGCAGTTTCGACTCCTGCTAATAGTTTAGCGCATTCTTTGTTGTTACTATGGGGTCCTGAAGCACAAGGAGATTTTACTCGCTGGTGTCAATTAGGCGGTCTTTGGACTTTTGTTGCTCTCCACGGTGCTTTCGGACTAATAGGTTTCATGTTACGTCAATTTGAACTTGCTCGCTCTGTTCAATTGCGACCTTATAATGCAATAGCATTCTCTGCTCCAATTGCTGTTTTTGTTTCTGTATTCCTGATTTATCCACTAGGTCAGTCTGGTTGGTTCTTTGCGCCTAGTTTTGGTGTAGCGGCGATATTTCGATTCATCCTTTTTTTCCAAGGGTTTCATAATTGGACATTGAACCCCTTTCATATGATGGGAGTTGCCGGTGTATTGGGCGCTGCTCTGCTATGCGCCATTCATGGCGCTACCGTAGAAAATACTTTATTTGAAGATGGTGATGGTGCAAACACATTCCGTGCTTTTAACCCAACGCAAGCCGAAGAAACCTATTCGATGGTCACCGCTAACCGCTTTTGGTCTCAAATCTTTGGGGTTGCTTTTTCCAATAAACGTTGGTTACATTTCTTTATGTTATTTGTACCAGTAACCGGTTTATGGATGAGTGCTCTTGGAGTAGTCGGCCTAGCCCTGAACCTACGTGCTTATGACTTCGTTTCCCAGGAAATCCGTGCAGCGGAAGATCCTGAATTTGAGACTTTCTACACAAAAAATATTCTTTTAAACGAGGGTATTCGTGCTTGGATGGCGGCTCAAGATCAGCCTCATGAAAACCTTATATTCCCTGAGGAGGTTCTACCCCGTGGAAACGCTCTTTAA

>psbC

ATGAAAACCTTATATTCCCTGAGGAGGTTCTACCCCGTGGAAACGCTCTTTAATGGAACTTTAGCTGTAGCAGGTCGTGACCAAGAAACCACCGGTTTCGCTTGGTGGGCCGGGAATGCCCGACTTATCAATTTATCCGGTAAACTGCTGGGCGCTCATGTAGCCCATGCTGGATTAATCGTATTCTGGGCCGGAGCAATGAACCTCTTTGAAGTGGCTCATTTCGTACCAGAAAAGCCCATGTATGAACAAGGATTAATTTTACTTCCCCACCTAGCTACTCTAGGCTGGGGGGTAGGTCCTGGTGGGGAAGTTATAGACACCTTTCCATACTTTGTATCTGGAGTACTTCACTTAATTTCCTCTGCAGTATTGGGCTTTGGCGGTATTTATCATGCACTTCTGGGACCTGAGACTCTTGAAGAATCTTTTCCATTCTTCGGTTATGTATGGAAAGATAGAAATAAAATGACCACAATTTTGGGTATTCACCTAATCTTGTTAGGTATAGGTGCTTTTCTTCTAGTATTCAAGGCTCTTTATTTTGGGGGCGTGTATGATACCTGGGCTCCGGGGGGGGGAGATGTAAGAAAAATTACCAACTTGACCCTTAGCCCAAGTGTTATTTTTGGTTATTTACTAAAATCCTTCTTTGGAGGAGAGGGGTGGATTGTTAGTGTGGATGATTTGGAAGATATAATTGGAGGTCATGTATGGTTAGGTTCCATTTGTATATTTGGTGGAATCTGGCATATCTTAACCAAGCCTTTTGCATGGGCTCGCCGTGCACTTGTATGGTCTGGGGAGGCTTACTTGTCTTATAGTTTAGGTGCTTTAGCTGTTTTTGGTTTCATTGCTTGTTGCTTTGTCTGGTTCAATAATACCGCTTATCCTAGTGAGTTTTATGGTCCCACTGGGCCAGAAGCTTCTCAAGCTCAAGCATTTACTTTTCTGGTTAGAGACCAACGTCTTGGGGCTAATGTGGGATCCGCTCAAGGGCCTACTGGTTTAGGTAAATATCTAATGCGTTCCCCTACTGGAGAAGTCATTTTTGGGGGAGAAACTATGCGTTTTTGGGATCTGCGTGCTCCATGGTTAGAACCTCTAAGGGGTCCCAACGGTTTAGACTTGAGTAGGTTGAAAAAGGACATACAACCTTGGCAAGAACGACGTTCTGCGGAATATATGACTCATGCTCCTTTAGGTTCTTTAAACTCTGTAGGTGGAGTAGCTACCGAGATCAATGCAGTTAATTATGTCTCTCCGAGAACTTGGTTAGCTACTTCTCATTTTGTTCTAGGCTTCTTCTTCTTCGTAGGTCACTTATGGCACGCGGGAAGAGCTCGTGCAGCTGCAGCAGGGTTTGAAAAAGGAATTGATCGTGATTTTGAACCTGTTCTTTCGATGACTCCTCTTAACTGA

>psbZ

ATGACTATTGCTTTCCAATTGGCTGTTTTTGCATTAATTGCTACTTCATCAATCTTACTGATTAGTGTACCCGTTGTATTTGCTTCTCCGGATGGTTGGTCGAGTAACAAAAATGTCGTATTTTCTGGTACATCATTGTGGATTGGATTAGTCTTTCTGGTGGGTATCCTTAACTCTCTCATTTCTTAA

>rps14

ATGGCAAGGCAAAGTTTGATTCAGAGGGAAAAGAAAAGGCACAAATTGGAACAAAAATATCATTTGATTCGTCGATCCTCAAAAAAAGAAATAAACAAAGCTCCATCGTTGAGCGATAAATGGAAAATTCATGGAAAGTTACAATCCTCACCGCGTAATAGTGCACCTACCCGTCTTCATCGACGTTGTTTTTTGACCGGAAGGCCGAGAGCTAACTATCGAGACTTTGGACTATCCGGACACATACTTCGTGAAATGGTTCATGCGTGTTTGTTGCCGGGGGCAACAAGATCAAGTTGGTAA

>psaB

ATGGCATTAAGATTTCCAAGGTTTAGCCAAGGCTTAGCTCAGGACCCCACTACTCGTCGTATTTGGTTTGGTATTGCTACCGCACATGACTTCGAGAGTCATGATGATATTACTGAGGAACGTCTTTATCAGAATATTTTTGCTTCTCACTTCGGGCAATTAGCAATAATTTTTCTGTGGACTTCCGGAAATCTCTTTCATGTAGCTTGGCAAGGAAATTTTGAGGCATGGGTCCAGGACCCTTTACATGTAAGACCTATTGCTCATGCAATTTGGGATCCTCATTTTGGTCAACCGGCCGTGGAAGCTTTTTCTCGGGGAGGTGCTCTTGGCCCGGTGAATATCGCTTATTCTGGTGTTTATCAGTGGTGGTATACAATCGGTTTACGCACTAATGAGGATCTTTATACTGGAGCTCTTTTTCTATTATTTCTTTCCGCCATATCTTTAATAGCGGGTTGGTTACACCTACAACCGAAGTGGAAACCGAGCGTTTCGTGGTTCAAAAATGCCGAATCTCGTCTGAATCATCATTTGTCAGGACTGTTCGGAGTAAGTTCCTTGGCTTGGACAGGGCATTTAGTACATGTCGCTATTCCTGGATCCAGGGGGGAGTATGTTCGATGGAATAATTTCTTAGATGTATTGCCGCATCCCCAAGGGTTAGGCCCACTTTTTACGGGTCAGTGGAATCTTTATGCGCAAAACCCCGATTCAAGTAGTCATTTATTTGGTACCTCCCAAGGATCAGGAACTGCCATTCTAACCCTTCTCGGGGGATTCCATCCACAAACGCAAAGCTTATGGCTGACCGATATTGCTCATCATCATTTAGCTATTGCATTTATTTTTCTCGTTGCTGGTCATATGTATAGAACGAATTTCGGGATTGGGCACAGTATAAAAGATCTTTTAGAAGCACATATTCCTCCGGGAGGACGCTTGGGGCGCGGGCATAAGGGTCTTTATGACACAATCAATAATTCGCTTCATTTTCAATTAGGCCTTGCTCTAGCCTCTTTAGGGGTTATTACTTCCTTGGTAGCTCAACACATGTACTCTTTACCTGCTTATGCATTCATAGCGCAAGATTTTACTACTCAAGCTGCGTTATATACTCATCACCAATACATCGCGGGATTCATCATGACAGGAGCTTTTGCTCACGGAGCTATCTTTTTTATTAGAGATTACAATCCGGAACAGAATGAGGATAATGTATTGGCAAGAATGTTAGACCATAAGGAAGCTATTATATCCCATTTAAGTTGGGCCAGCTTGTTTCTGGGGTTCCATACTTTGGGACTTTATGTTCATAATGATGTCATGCTTGCTTTTGGTACTCCGGAGAAACAAATCTTGATCGAACCGATATTTGCCCAATGGATACAATCTGCTCATGGTAAAACTTCATATGGATTCGATGTACTTTTATCTTCAACGGATGGCCCTGCATTCAATGCGGGTCGAAGCATATGGTTGCCTGGCTGGTTAAGTGCTGTTAATGAGAATAGTAATTCTCTATTCTTAACAATAGGCCCTGGAGACTTTTTGGTTCATCATGCTATTGCTCTAGGTTTACATACAACTACATTGATCTTGGTAAAGGGGGCTTTAGATGCACGGGGTTCTAAGTTAATGCCAGATAAAAAGGATTTCGGTTATAGTTTTCCTTGCGACGGTCCGGGACGAGGCGGTACTTGTGATATTTCGGCTTGGGACGCATTTTATTTGGCAGTTTTCTGGATGTTAAATACCATTGGGTGGGTTACTTTTTATTGGCATTGGAAACACATCACGTTATGGCAGGGTAACGTTTCACAGTTTAATGAATCTTCCACTTATTTGATGGGATGGTTAAGAGATTATCTATGGTTAAACTCTTCCCAACTTATCAATGGGTATAACCCGTTTGGTATGAATAGTTTATCAGTCTGGGCGTGGATGTTCTTATTTGGACATCTTGTTTGGGCTACTGGATTTATGTTTTTAATTTCCTGGCGCGGGTATTGGCAAGAATTGATTGAAACTTTAGCGTGGGCTCACGAACGCACACCCTTAGCTAATTTGATTCGATGGAGAGATAAACCAGTGGCCCTTTCCATTGTGCAAGCACGATTGGTTGGATTAGCCCACTTCTCTGTAGGTTATATATTCACTTATGCGGCTTTCTTGATTGCCTCTACATCGGGCAAATTTGGTTAA

>psaA

ATGATTATTCGTTCGCCGGAACCAGAAGTAAAAATTTTGGTAGATAGGGATCCCGTAAAAACTTCTTTCGAGGAATGGGCCAAACCGGGGCATTTCTCAAGAACCATAGCTAAGGGACCTGAGACTACCACTTGGATCTGGAACCTACATGCTGATGCTCACGACTTCGATAGCCATACCAGTGATTTGGAGGAGATCTCTCGAAAAGTATTTAGTGCCCATTTCGGACAACTCTCCATCATTTTTCTTTGGCTGAGCGGAATGTATTTCCACGGTGCTCGTTTTTCCAATTATGAAGCCTGGCTAAGCGATCCTACTCACATTGGACCTAGCGCACAGGTGGTTTGGCCAATAGTGGGCCAAGAAATCTTGAACGGTGATGTGGGCGGGGGTTTCCGAGGAATACAAATAACCTCCGGGTTTTTTCAGCTTTGGCGAGCATCTGGAATAACTAGTGAATTACAACTCTATTGTACCGCAATTGGCGCATTGATTTTTGCAGCCTTAATGCTTTTTGCTGGTTGGTTCCATTATCACAAAGCTGCTCCAAAATTGGCTTGGTTTCAGGATGTAGAATCTATGTTGAACCACCATTTAGCGGGGCTGCTAGGGCTCGGGTCCCTTTCTTGGGCCGGGCATCAAGTACATGTATCTTTACCGATTAACCAATTTCTAAACGCTGGAGTAGATCCTAAAGAGATCCCACTTCCTCATGAATTTATCTTGAATCGGGATCTTTTGGCTCAACTTTATCCCAGTTTTGCCGAAGGAGCAACCCCATTTTTTACCTTGAATTGGTCAAAATATGCGGAATTTCTTACTTTTCGTGGCGGATTAGATCCAGTAACTGGGGGTCTATGGCTGACCGATATTGCACACCATCATTTAGCTATTGCAATTCTTTTCCTGATCGCGGGGCACATGTATAGGACCAACTGGGGGATTGGTCATGGTCTAAAAGATATTTTAGAGGCTCATAAAGGTCCATTTACAGGTCAAGGCCATAAAGGATTATATGAGATCCTAACAACATCATGGCATGCTCAATTATCGCTTAACCTAGCTATGTTAGGATCTTTAACCATTATTGTAGCTCATCATATGTATTCCATGCCCCCTTATCCATATCTAGCTACTGACTATGGTACACAACTGTCATTGTTCACACACCACATGTGGATTGGTGGATTTCTCATTGTTGGCGCTGCTGCGCATGCAGCCATTTTTATGGTAAGAGACTATGATCCAACTACTCGATACAACGATCTCTTAGATCGTGTCCTTCGGCATCGCGATGCAATCATATCACATCTCAACTGGGTATGTATATTTCTAGGATTTCACAGTTTTGGTTTGTATATTCATAATGATACCATGAGTGCTTTAGGGCGTCCACAAGATATGTTTTCAGATACCGCGATACAATTACAACCTGTCTTTGCTCAATGGATACAAAACACCCACGCCTTAGCACCCGGCGGAACGGCCCCTGGTGCAACAGCAAGCACCAGTTTGACTTGGGGGGGTGTTGATTTAGTGGCAGTGGGCGGAAAAGTTGCTTTGTTACCTATTCCATTAGGAACCGCGGATTTTTTGGTACATCACATTCATGCATTTACGATTCATGTGACGGTATTGATACTCCTGAAAGGAGTTCTCTTTGCTCGTAGCTCGCGTTTGATACCGGATAAAGCAAATCTTGGGTTTCGTTTCCCTTGTGATGGTCCTGGAAGAGGGGGGACATGTCAAGTATCCGCTTGGGATCATGTCTTCTTAGGACTATTCTGGATGTACAATGCAATTTCGGTAGTAATATTCCATTTCAGTTGGAAAATGCAGTCAGATGTTTGGGGTAGTATAAGTGATCAGGGGGTGGTAACTCATATTACAGGAGGAAACTTTGCGCAGAGTTCCATTACGATTAATGGGTGGCTCCGCGATTTTTTATGGGCACAGGCATCCCAGGTAATTCAGTCTTATGGTTCCTCATTATCTGCATATGGCCTTTTTTTCCTAGGTGCTCATTTTGTATGGGCTTTTAGTTTAATGTTTCTATTCAGCGGGCGTGGTTATTGGCAAGAACTTATTGAATCCATCGTTTGGGCTCATAATAAATTAAAAGTTGCTCCTGCTACTCAGCCTAGAGCCTTGAGCATTGTACAAGGACGCGCTGTAGGAGTAACCCATTACCTTCTAGGTGGAATTGCCACAACATGGGCGTTCTTCTTAGCAAGAATTATTGCAGTAGGATAA

>ycf3

ATGCCTAGATCGCGGATAAACGGAAATTTTATTGATAAGACCTTTTCGATTGTAGCCAATATATTATTACGAATAATTCCGACAACTGTAGGAGAAAAAGAGGCATTTACCTATTACAGAGATGGTATGTCAGCTCAATCTGAAGGCAATTATGCGGAAGCTTTACAGAATTATTATGAAGCTATGCGACTAGAAATTGATCCTTACGATCGAAGCTATATACTCTATAACATAGGCCTTATCCACACAAGTAACGGAGAACATACAAAAGCTTTAGAATATTATTTTCGGGCACTAGAACGAAATCCGTTCTTACCACAAGCTTTTAATAATATGGCTGTGATCTGTCATTACCGGGGGGAACAGGCCGTTCGACAGGGAGATTCTGAAATTGCGGAGGCTTGGTTCAATCAAGCTGCCGAGTATTGGAAACAAGCTATTGCGCTTACTCCTGGTAATTATATTCAAGCGCAGAATTGGTTGAAGATCACGGGACGTTTCGAATAA

>rps4

ATGTCACGTTACCGAGGGCCTCGTTTCAAAAAAATACGCCGTCTGGGGGCTTTGCCGGGACTAACGAGTAAAAGGCCTAGAGCCGGAAGCGATTTTAGAAACCAATCGCGCTCCGTAAAAAAATCTCAATATCGAATTCGTTTAGAAGAAAAACAAAAATTGCGTTTTCATTATGGTCTTACAGAACGACAATTACTTAAATATGTTCGTATCGCCAGAAAAGCCAAAGGGTCAACCGGTCTGGTTTTACTACAATTACTTGAAATGCGTTTGGATAACATTCTTTTTCGATTGGGTATGGCTTCAACTATTCCTCAAGCCCGCCAATTGGTTAACCATCGACATATTTTAGTTAATGGTCGTATAGTCGATATACCAAGTTATCGTTGCAAACCCCGAGATATTATTACAGTAAGGGATGACCCAAAATCTAGATCTCTGGTTCAAAATTATCTTGATTCACCCCACCATGAGGAATTGCCAAAGCATTTGACTCTTCGCGCATTCCAATATAAAGGATTAGTCAATCAAATAATAGATAGTCAATGGGTCGGTTTGAAAATAAATGAATTGCTTGTCGTAGAATATTATTCTCGTCAAACTTAA

>ndhJ

ATGCAGGGTCGTTTGTCTGCTTGGCTGGTCAAGCATGGGCTAGTTCATAGATCTTTGGGCTTTGATTACCAAGGAATAGAAACTTTACAAATAAAGCCCGAGGATTGGCACTCCGTTGCTGTCATTTTGTATATATATGGCTACAATTATCTACGGTCCCAATGTGCCTATGATGTAGCACCGGGCGGACTGTTAGCCAGTGTGTATCATCTTACGCGAATAGAGTATGGTGTAGATCAACCAGAAGAGGTATGCATAAAAGTATTTGCTCCAAGGAGTAATCCCAAAATTCCATCGGTTTTCTGGGTTTGGAAAAGTGCGAATTTTCCAGAACGGGAATCTTATGATATGCTGGGAATCCTTTATGATAATCATCCACGACTGAAACGTATCTTAATGCCTGAAAGTTGGATAGGGTGGCCCTTACGTAAGGATTATATTGCCCCCAATTTTTATGAAATACAAGATGCTTATTGA

>ndhK

ATGAATTCTATTGAATTTTCCTTACTTGATCGAACAACCCCAAATTCATTTATTTCAACTACATCAAACGATCTTTCAAATTGGTCAAGACTCTCCAGCTTATGGCCGCTTCTTTATGGTACCAGTTGTTGTTTCATTGAATTTGCTTCATTAATCGGCTCGCGGTTCGATTTTGACCGTTATGGGCTGGTACCAAGATCGAGCCCTAGACAGGCGGACCTAATTTTAACAGCTGGTACAGTAACAATGAAAATGGCTCCTTCTTTAGTGAGATTATATGAACAAATGCCTGAACCAAAATATGTTATTGCTATGGGAGCATGTACAATTACAGGGGGGATGTTCAGTACCGATTCGTATAGTACTGTTCGAGGAGTTGATAAGCTAATTCCCGTGGATGTTTATTTGCCGGGTTGCCCGCCTAAACCAGAGGCCGTTATAGATGCCATAACAAAACTTCGTAAGAAAATATCTCGAGAAATCTATGAAGATCGAATTCGATTGCAACGGGAGAATCGCTCGTTTACTTTTACTACCAATCACAAGTTTCGTGTTGTATGCAGTACTAATACTGGAAATTATGATCAAGGATTACTTTATCAACCACCATCTACGTCAGAAATCCCGCCTGAAACCTTTTTCAAATACAAAAGTTCAGTATCTTCCCCCGAATTCATTAATTAG

>ndhC

ATGTTTCTGCTTTACAAATATGATATTTTCTGGGCATTTCTAATAATATCAAGCGTTATTCCTATTTTGGCATTTCTAATTTCCGCAGTTTTAGCCCCGATTAACAAAGGGCCAGAGAAACTTTCTAGTTATGAATCGGGTATCGAACCAATGGGCGATGCTTGGTTACAATTTCGAATCCGGTATTATATGTTTGCTCTAGTTTTTGTTGTTTTTGATGTTGAAACCGTTTTTCTTTATCCATGGGCAATGAGTTTTGATGTATTGGGGGTACCCGTATTTATAGAAGCTTTCATTTTCATGCTTATCCTAATTGTTGGTTTAGTTTATGCGTGGCGAAAAGGAGCATTAGAGTGGTCTTAG

>atpE

ATGACCTTAAATCTTTGTGTACTGACCCCTAATCGAATTGTTTGGGATTCAGAAGTGAAAGAAATCATTTTATCTACTAATAGTGGACAAATCGGCGTATTACCAAATCACGCGCCTATTGCCACAGCTGTAGATATAGGTATTTTAAGAATCCGCTTTAACGACCAATGGTTAACGATGGCTCTGATGGGTGGTTTTGCTAGAATAGGGAATAATGAGATCACTATTTTAGTAAATGATGCGGAGAAGAGTAGTGACATTGATCCCCAAGAAGCCCAGCAAACTCTTGAAATAGCAGAAGCTAATTTGAGGAAAGCTGAAAGCAAGAGACAAACAATTGAGGCAAATCTAGCTCTCAGACGAGCTAGGACACGAGTAGAGGTTATCAATGCGATTTCATAA

>atpB

ATGAGAATAAATCCTACTACTTCCGGTCCTGGGGTTTCCGCGTTTGCAAACAAAAACCTAGGACATATCGCTCAAATCATTGGTCCGGTACTGGATGTAGCCTTTCCCCCCGGCAAGATGCCTAATATTTACAATGCTCTGGTAGTTAAGGGTCGAGATACTGTCGATCAACCAATTAATGTGACTTGCGAGGTACAGCAATTATTAGGGAATAATCGAGTTAGAGCTGTAGCCATGAGTGCTACAGATGGTCTAACGAGGGGTATGGAAGTAATTGACACGGGAGCTCCTCTAAGTGTTCCAGTCGGCGGAGTGACTCTAGGACGAATTTTCAACGTGCTTGGAGAGCCCGTTGATAATTTAGGTCCTGTAGATACTCGCACAACATCCCCTATTCATAAATCCGCGCCTGCTTTTATACAGTTAGATACAAGATTATCTATTTTTGAAACAGGAATTAAAGTAGTAGATCTTTTAGCTCCTTATCGTCGGGGAGGAAAAATCGGACTATTTGGGGGAGCTGGGGTGGGTAAAACAGTACTCATTATGGAATTGATCAACAACATTGCCAAAGCCCATGGGGGTGTATCCGTATTTGGCGGAGTCGGTGAACGTACTCGTGAAGGAAATGATCTTTACATGGAAATGAAAGAGTCTGGAGTAATTAATGACCAAAATCTTTCGGAATCAAAAGTGGCTCTAGTCTACGGTCAGATGAATGAACCACCAGGAGCTCGTATGAGAGTTGGTTTGACGGCCCTAACTATGGCGGAATATTTCCGAGATGTTAATGAACAAGACGTACTTCTATTTATCGACAATATCTTCCGTTTCGTCCAAGCGGGATCCGAGGTATCCGCCTTATTGGGTAGAATGCCTTCCGCTGTGGGTTATCAACCTACCCTTAGTACCGAAATGGGTTCTTTACAAGAAAGAATTACTTCTACCAAAGAGGGGTCCATAACTTCTATTCAAGCAGTTTATGTACCCGCGGATGATTTGACTGATCCCGCTCCTGCCACGACATTTGCCCATTTAGATGCTACTACCGTACTATCAAGAGGATTAGCTGCTAAAGGTATCTATCCAGCAGTTGATCCTTTAGACTCAACGTCAACTATGCTGCAACCTCGGATCGTTGGCGAGGAACATTATGAAACGGCGCAAAGAGTTAAGCAAACTTTACAACGTTACAAAGAACTTCAGGACATTATAGCTATCCTTGGGTTGGACGAACTGTCCGAAGAGGATCGTTTAACTGTAGCAAGGGCGCGAAAAATTGAGCGTTTCTTATCACAACCCTTTTTCGTAGCCGAAGTATTTACCGGTTCCCCGGGGAAATATGTTGGTCTAGCCGAAACTATTAGAGGGTTTAAATTGATCCTGTCCGGAGAATTAGATGGTCTTCCTGAGCAGGCCTTTTATTTGGTAGGTAACATAGATGAAGTTACTGCGAAGGCTACAAACTTAGAAATGGAGAGTAATTTGAAGAAATGA

>rbcL

ATGTCACCACAAACAGAGACTAAAGCGAGTGTTGGATTCAAGGCCGGTGTTAAAGATTATAAATTGACTTATTATACTCCTGACTATGTAACCAAAGATACTGATATCTTGGCAGCATTCCGAGTAACTCCTCAGCCCGGAGTTCCACCCGAGGAAGCGGGGGCTGCGGTAGCTGCGGAATCTTCTACTGGTACCTGGACAGCTGTGTGGACCGATGGGCTTACCAGCCTTGATCGTTACAAAGGGCGATGCTACAACATTGAGCCCGTTGCTGGAGAAGAGAATCAATATATATGTTATGTAGCTTACCCGTTAGACCTTTTTGAAGAAGGTTCTGTTACTAACATGTTTACTTCCATTGTGGGTAATGTATTTGGTTTCAAAGCACTGCGCGCTCTACGTCTAGAGGATCTACGAATCCCTCCTGCGTATACTAAAACTTTCCAAGGCCCGCCTCACGGCATCCAAGTTGAGAGAGATAAATTGAACAAGTATGGCCGTCCCCTGTTGGGATGTACTATTAAACCTAAACTGGGGTTATCCGCGAAGAATTATGGTAGGGCGGTTTATGAATGTCTACGTGGTGGACTTGACTTTACCAAAGATGATGAGAACGTGAACTCCCAACCATTTATGCGTTGGAGGGACCGTTTCTTATTTTGTGCGGAAGCTCTTTATAAAGCTCAAGCTGAAACAGGTGAAATCAAAGGTCATTACTTGAATGCTACTGCAGGGACATGCGAAGAAATGCTAAAAAGGGCTGTCTTTGCCAGAGAGTTGGGAGTTCCTATCGTAATGCATGACTACTTAACAGGGGGATTCACCGCAAATACTACCTTGGCTCATTATTGCCGAGATAATGGTCTACTTCTTCACATCCACCGTGCAATGCATGCAGTTATTGATAGACAGAAGAATCATGGTATGCACTTTCGTGTACTAGCTAAAGCTTTGCGTCTGTCTGGTGGAGATCATGTTCACGCCGGTACAGTAGTAGGTAAACTTGAGGGGGAAAGAGACATAACCTTGGGATTTGTTGATTTACTACGTGATGATTTTGTTGAAAAAGATCGAAGCCGCGGTATTTATTTCACTCAAGATTGGGTCTCTATACCAGGTGTTATACCTGTGGCTTCCGGGGGTATTCACGTTTGGCATATGCCTGCGTTGACAGAGATCTTTGGAGATGATTCCGTACTACAATTTGGTGGAGGAACTTTAGGACACCCTTGGGGAAATGCACCCGGCGCTGTAGCTAATCGAGTATCTCTAGAAGCATGTGTACAAGCTCGTAATGAAGGACGCGATCTTGCTCGTGAAGGTAATGAAATTATCCGGGAGGCTAGCAAATGGAGTCCTGAACTGGCTGCTGCTTGTGAAGTCTGGAAGTCGATCAAATTCGAATTTGCCGCAATGGATACTTTGTAA

>accD

ATGAAAAAATGGTGGTTCAATTCGATGTTATCTAAGGGTAAGGGGGAATTAGAATACAGGTGTTGGTTAAGTAAATCAATGGAGAGCCCTGGTCCTATTAAAAATCCCAGTGTAAGCGAGGAACTGATTCGAAATGATAAGAATAAAAACATTCATAATTCGAGCGATAGTGACAGTTCAAGTTACAGCAAATTAGCTGGTGTCAGGGACATTCATAATTTCCTCTCGGATGACACTTTTTTTGTTAAGGATAGTAATAGAGACAGTTATTCCATCTATTTTGATATTGAAAATCAAATTTTGGAACTAGACAATGCTCATTCTTTTCTGAGTGAACTAGAAAGTTCTTTTTATAGCTTTCGTAATTATAGTTCTAGGAATAATGGATCTAAAAGCGCTGATCCGGATTCCGATCGTTACATGTATGATACTAAATCGAGTTGGAATAATCACATTCATAATTGCCTTGACTCTTATCTTCATTCTCAAATCTGTATTGATAGTCACGTTTTAAGTAGTAGTGACAATTATAGTGCCAGTTACATTTATAATTTCATTTGTAGTGAAAGTGAGAGTTCCAATATACAAAGTAGCACGAATGGTAGTGATTTAACTATAAGCGAAAGTTCTAATGAAAGCGAAAGTTCTAATGAAAGCGATGTAACTCAAAAATACAGGCATTTATGGGTTCAATGCGAAAATTGTTATGGATTAAATTATAAGAAATTTCTTAAATCAAAAATGTATCTTTGTGAACAATGCGGATATCATTTGAAAATGATTAGCTCAGATAGAATCGACCTTTTGGTTGATCCAGGTACTTGGGATCCGATGGATGACGACATGGTCTCTATAGATCCCATTGAATTTGATTCAGAAGAGGAACCTTATAAAAATCGTATTGATTCTTATCAAAGCAAGACAGGATTAACGGAGGCTGTTCAAACAGGTACAGGGCAACTAAACGGGATTCCCATCGCAATTGGGGTTATGGATTTTCAGTTTATGGGGGGTAGTATGGGATCCGTAGTAGGCGAGAAAATAACCCGTTTGATCGAGTATGCTGCCAATAAATTTTTACCTCTTCTTCTAGTGTGTGCTTCTGGGGGAGCACGCATGCAAGAAGGAAGTTTGAGCTTGATGCAAATGGCTAAAATATCTTCTGCTTTATATGATTATCAATCAAATAAAAAGTTATTCTATGTATCAATTCTTACATCCCCTACTACGGGTGGAGTGACAGCTAGTTTTGGTATGTTGGGGGATATCATTATTGCTGAACCTAATGCCTATATTGCATTTGCAGGTAAAAGAGTAATTGAACAAACATTGAATAAGACAGTACCTGAAGGTTCACAAGAGGCTGAATATTTATTCGATAAGGGCTTATTCGATCCAATCGTACCACGTAATCCTTTAAAAGGTGTTCTGAGCGAGTTATTTCTGTTCCACGGCCGTTTTCCTTTGAATCAAAATTAA

>psaI

ATGACAATTCTCAACAGCTTTCCCTCTATTTTTGTGCCTTTAGTGGGCCTAGTATTTCCGGCAATGGCAATGGCTTCGTTATTTCTTTATCTTGAAAAAAATAAGATTTTGTAA

>ycf4

ATGAGTTGGCGATCAGAATATATATGGGTAGAATTTATAGCGGGCTCTCGCAAACCAGGCAATTTCTTCTGGGCCTTTATCCTTTTTTTAGGCTCATTAGGATTCTTAGTGGTTGGAATTTCTAGTTATCTTGATAGGAATTTGCTATCTTTATTGCCGTCGCAGCAAATAAATTTTTTTCCACAAGGGATCGTGATGTCTTTCTACGGGATCGCGGGTCTCTTTATTAGTTCCTATTTGTGGTGCACAATTATATGGAATGTAGGTAGCGGTTATGATCGATTTGATACAAAAGAGGGAATAGTGTGTATTTTTCGTTGGGGATTTCCTGGAAAAAATCGCCGCATCTTTCTACGATTCCTTATGAAAGATATTCAGTCCATCAGAATAGAAGTTAAAGAGGGTATTTATGCTCGTCGTGTCCTTTATATAGAAAGCAGAGGCTTGGGGGCCATTCCCTTGAATCGTACTGATGAGAATTTGACTCCGCGAGAAATTGAGCAAAAGGCTGCGGAATTGGCCTATTTCTTGCGTGTACCAATTGAAGGATTTTGA

>cemA

ATGACAAAAAAGAACGCATCCATTCCCCTTAGATATCTTTCATCTATAGTATTTGTAGTATTTTTGCCCTGGTGGATCCCTCTCTCATTTAATAAAAGTCTGGAATCTTGGGTTACTAATTGGTGGAATACTAGTCAATCCGAAACCTTTTTGAATGATATTCAAGAAAAGGCTATTCTAGAAAAATTCATAGAATTAGAGGAATTATTTCTCTTGGACGAAATGATAAAGGAATTTCCGGAAAGACGTCTAGAAAAGCTTCGTATAGGGCTCCAGAAAGAAACAATCCAATTAATCAAGATGCACGATGAGGATCATATCCATACGATTTTTCACTTCTCGACAAATACAATCTGCTTCGTTATTCTAAGTGGTTATTCTATTTTGTGTAATGAAGAACTTTTTATTCTTAACTCTTGGGTTCAAGAATTCCTATATAATTTAAGCGACACAATAAAAGCCTTTTCGATTCTTTTCGTAACTGATTTATGTATCGGATTCCATTCACCCCGCGGTTGGGAACTACTGATTGGCTATGTCTACAACGACTTTGGGCTTGCTCATAATGATAATGATATTATTCTATCTGTTCTTGTTTCCACTTTTCCAGTCGTTCTAGATACATTTTTTAAATATTGGCTTTTTTCTTATTTAAATCGTGTATCTCCGTCACTTGTAGTGATTTATCATTCAATGACTGAGTGA

>petA

ATGCAAATTAGAAATACCTTTTCTTCGTTAAAGGGAGAGATTACTCGATTCATTTCCGTATCCCTCATGATATATATAATAACTCGGGCATCAATTTCAAATGCATATCCCATTTTTGCGCAGCAGGGTTTTGAAAATCCACGAGAAGCAACTGGTCGTATTGTATGCGCCAATTGCCATTTAGCTAATAAGCCCGTGGATATTGAGGTTCCACAGGCGGTACTCCCCGATACTGTATTTGAAGCAGTTGTTAGAATTCCTTATGATATGCAACTAAAACAAGTTCTTGCTAATGGTAAAAAGGGGGCTTTGAATGTGGGGGCCGTTCTTATTTTACCAGAGGGGTTTGAATTAGCCCCCCCCGATCGTATTTCGCCCGAGATGAAAGAAAAGATAGGCAAGCTGTCTTTTCAGACCTACCGACCCACTAAAAAAAACATTCTTGTGATAGGGCCAGTTCCTGGTCAGAAATATAGTGAAATAACTTTTCCTATTCTTTCCCCGAACCCCGCGACTAATAAAGATGCTTACTTCTTAAAATATCCAATATACGTAGGTGGGAACAGGGGAAGGGGTCAGATTTATCCCGACGGGAACAAAAGTAACAATACGGTTTATAATGCTACAGCTGCGGGTATAGTAAGCAAAATCATACGAAAAGAAAAAGGGGGATACGAAATAACCATAACGGATGCATCGAATGGACGTGAAGTGGTTGATATTATCCCCCCAGGACCAGAACTTCGTGTTTCAGAGGGCCAATCTATCAAACTTGATCAACCATTAACAAGTAATCCTAATGTAGGCGGGTTTGGTCAGGCAGATGCAGAAATAGTACTTCAAGATCCATTACGTGTCCAAGGCCTTTTGTTCTTTTTGGCATCTGTTGTTTTGGCACAAATATTTTTGGTTCTTAAAAAGAAACAGTTTGAGAAGGTTCAATTGTCCGAAATGAATTTCTAG

>psbJ

ATGGCCGATACTACTGGAAGGATTCCCCTTTGGATAATAGGAACTGTAACTGGTATTCCTGTGATCGGTTTAATAGGCATTTTCTTTTATGGTTCATATTCCGGATTAGGTTCGTCCTTGTAG

>psbL

ATGACACAATCAAACCCAAACGAACAAAATGTTGAATTGAACCGTACCAGTCTCTACTGGGGGTTATTGCTCATTTTTGTACTTGCTGTTTTATTTTCCAATTACTTCTTCAATTAA

>psbF

ATGACCATAGATCGAACCTATCCAATTTTTACAGTGCGATGGTTGGCTGTTCACGGACTAGCTGTACCTACCGTTTCTTTTTTGGGGTCAATATCAGCAATGCAGTTCATCCAACGCTAA

>psbE

ATGTCTGGAAGCACAGGAGAACGTTCTTTTGCTGATATTATTACCAGTATTCGATACTGGGTCATTCATAGCATTACTATACCTTCCCTATTCATTGCGGGTTGGTTATTCGTCAGCACGGGGTTAGCTTATGATGTGTTTGGAAGCCCCCGGCCAAACGAGTATTTTACAGAGAGCCGGCAAGGAATTCCATTAATAACCGGCCGTTTTGATTCTTTGGAACAACTCAATGAATTTAGTAGATCTTTTTAG

>petG

ATGATTGAAGTCTTTCTATTTGGAATCGTCTTAGGTCTAATTCCTATTACTTTGGCTGGATTATTCGTAACCGCCTATTTACAATACAGACGTGGTGATCAGTTGGACCTTTGA

>psaJ

ATGCGAGATCTAAAAACATATCTTTCCGTGGCACCGGTACTAAGTACTCTATGGTTCGGGTCTTTAGCAGGGTTATTGATAGAAATCAACCGTTTATTCCCCGACGCATTGACATTTCCTTTTTTTTCATTCTAG

>rpl33

ATGGCCAAGGGTAAAGAGGTCCGAGTAAGGGTTATTTTGGAATGTACTAGTTGTGTTCGAAACGGTGTTAATAAGGAATCAAGGGGTATTTCCAGATATATTACTCAAAAGAATCGACACAATACACCCAGTCGATTGGAATTGAGAAAATTCTGTCCCTATTGTTACAAGCATACACTTCATGGGGAGATAAAAAAATAG

>rps18

ATGGATAAAACCAAGCGACTCTTTCTTAAATCCAAGCGATCTTTTCGTAGGCGTTTGCCCCCGATCCAATCGGGGGATCGAATTGATTATAGAAACATGACTTTAATTAGTCGATTTCTTAGTGAACAAGGAAAAATATTATCTAGACGGGTGAATAGATTGACCTTAAAAGAACAACGATTAATTACTATTGCTATAAAACAAGCTCGTATTTTATCTTCGTTACCTTTTCTTAATAATGAGAAACAATTTGAAAGAAGTGGGTTGACCGCTAGACCTCCCGGTCTTAGAACCAGAAAAAAATAG

>rpl20

ATGACCAGAATTAGACGAGGATATATAGCTCGGAGACGTAGAACAAAAATGCGTTTATTTGTATCAAGCTTTCGCGGGGCTCATTCACGACTTAGCCGAACAATTACTCAACAGAAAATAAGAGCTTTGGTTTCGGCTCATCGCGATAGAGATAGGAAAAAAAGAGATTTTCGTCGTTTGTGGATCACCCGAATAAATGCAGTAATTCGCGGAAATCAGGTATCCTATAGTTATAGTAGATTAATATACAATCTGTATAAGGCGCAGTTGGTTCTTAATCGTAAGATACTTGCACAAATAGCTATATCAAATAGGACTTGTCTTTATATGATTTCCAATGAGATTATAAAATAA

>rps12

ATGCCAACTATTAAACAACTTATTAGAAACCCAAGACAGCCAATCAGAAATGTTACCAAATCCCCCGCTCTGCGGGGATGTCCTCAGCGCCGAGGAACATGTACAAGGGTGTATACTATCACCCCCAAAAAACCAAACTCTGCCTTACGTAAAGTTGCCAGAGTACGATTAACCTCTGGGTTTGAAATCACTGCTTATATACCTGGTATTGGCCATAATTCACAAGAACATTCTGTAGTCTTAGTAAGAGGGGGGAGGGTTAAGGATTTACCCGGTGTGAGATATCACATTGTTCGAGGAACCCTAGATGCTGTCGGAGTAAAGGATCGTCAACAAGGGCGTTCTAAATATGGGGTCAAAAAGCCAAAATAA

>clpP

ATGCCTATTGGTGTTCCAAAAGTACCTTATCGAAGTCCCGGGGACAAGCATCCATCTTGGGTTGACATATACAACCGACTTTATCGAGAAAGATTACTTTTTTTAGGTCAAATGGTTGAGAGCGATATCTCGAATCAACTTATTGGTATTATGGTATATCTCAGTATAGAGAACGAGACCAAGGATTTGTATTTATTTATCAACTCTCCTGGCGGATGGGTAATACCCGGGATAGCAATTTATGATACTATGCAATTTGTGCGACCCGATGTACAGACAATATGCATGGGATTGGCCGCCTCCATGGGGTCTTTTCTCCTGGCCGCCGGAGCAAGTACCAAACGTCTAGCATTCCCTCACGCTAGGGTCATGATCCATCAACCTATTGGCGCTTTTTATGGGGCACAAACGGGAGAATTTATCCTGGATACGGAAGAACTACTGAGACTGCGCGAAATCCTTACAATGGTTTATGTACAAAGATCGGGCAAGCCCTTATGGGTTGTATCCGAAGACATGGAAAGGGATACTTTTATGTCAGCAACAGAAGCCCAAGCTCATGGCCTTGTTGATCTTGTAGCGGTTGGATAA

>psbB

ATGGGTTTGCCTTGGTATCGTGTTCATACCGTCGTATTGAATGATCCCGGTCGTTTGATTGCTGTCCATATAATGCATACAGCCCTGGTTGCGGGTTGGGCCGGTTCAATGGCTCTATATGAATTAGCTGTTTTTGATCCCTCCGATCCAGTTCTTGATCCAATGTGGAGACAAGGCATGTTCGTTATACCCTTCATGACTCGTTTAGGAATAACCGATTCATGGGGTGGTTGGAGTATTACAGGGGGGACAGTAACGAATCCGGGTATTTGGAGTTACGAAGGTGTAGCGGGGGCACATATTGTGTTTTCCGGATTGTGCTTCTTGGCAGCTATCTGGCATTGGGTGTATTGGGATCTAGCAATATTTGTTGATGACCGTACAGGAAAACGTTCTTTGGATTTGCCTAAAATCTTTGGAATTCATTTATTTCTCTCAGGAGTGGCTTGCTTTGGTTTTGGGACATTTCATGTAACAGGATTGTATGGTCCTGGAATATGGGTGTCTGATCCGTATGGACTAACTGGAAAGGTACAATCTATAAATCCAGCATGGGGTGTGGAAGGTTTTGATCCTTTTGTTCCAGGAGGAATAGCCTCTCATCATATTGCGGCAGGGACATTGGGCATATTAGCAGGCTTATTCCATCTCAGCGTCCGCCCGCCACAACGCTTATACAAAGGATTACGTATGGGCAATATTGAAACCGTTCTTTCCAGCAGCATCGCTGCTGTCTTTTTTGCAGCGTTTGTTGTTGCTGGAACTATGTGGTATGGGTCAGCAACTACCCCCATCGAATTATTTGGTCCCACCCGTTATCAATGGGATCAGGGATACTTTCAGCAAGAAATATATCGAAGAGTCAGTGCTGGACTAGCCGAAAATCAAAGTTTATCAGAAGCTTGGTCTAAAATTCCTGAAAAATTAGCTTTTTATGATTACATCGGAAATAATCCTGCGAAAGGGGGATTATTCAGAGCGGGTTCAATGGATAACGGGGATGGAATAGCTGTCGGGTGGTTAGGACACCCTATATTTAGAGATAAAGAAGGGCGTGAACTTTTTGTACGTCGTATGCCTACTTTTTTTGAAACATTTCCAGTTGTTTTGGTAGACGGAGATGGAATTGTTAGAGCCGACGTTCCTTTTCGAAGGGCAGAATCGAAGTATAGTGTCGAACAAGTAGGTGTAACCGTTGAGTTCTATGGTGGCGAGCTGAATGGCGTGAGTTATAGTGATCCTGCTACTGTGAAAAAATATGCTAGACGTGCTCAATTGGGTGAAATTTTTGAATTAGATCGTGCTACTTTGAAATCCGATGGTGTTTTTCGTAGCAGCCCAAGGGGCTGGTTTACGTTTGGACACGCTTCATTTGCTCTGCTTTTCTTCTTCGGACACATTTGGCATGGTGCTAGAACCTTGTTCAGAGATGTTTTTGCTGGTATTGACCCGGATTTGGACGCTCAAGTGGAATTTGGAGTATTCCAAAAACTTGGAGATCCAACTACAAGAAGACAAGTAGTCTGA

>pbf1

ATGGAAACAGCAACCCTAGTCGCCATCTCTATATCTGGGTTACTTGTAAGTTTTACTGGGTACGCCTTATATACTGCTTTTGGGCAACCCTCTCAACAACTAAGAGATCCATTCGAGGAACACGGGGACTAG

>psbH

ATGGCTACACAAACAGTTGAGGGTAGTTCTAGAGCTCGTCCAAAAAGAACTTCTGCAGGGGGCTTGTTGAAACCCTTGAATTCGGAATATGGTAAAGTAGCTCCTGGATGGGGAACTACTCCTTTGATGGGAGTCGCAATGGCTTTATTTGCGGTATTCCTATCTATTATTTTGGAGATTTATAATTCGTCCGTTTTACTGGACGGAATTTCACTGAATTAG

>petB

ATGAGTATAAAATTCTCATATACGGTTCTCAGAGGGGAGTTCTCTTGGTTTACCTATCTCAATAAAGTCTACGATTGGTTCGAAGAACGTCTCGAGATTCAGGCGATTGCAGATGATATAACTAGTAAATACGTTCCTCCTCATGTCAACATATTTTATTGTCTGGGAGGAATTACGCTCACTTGTTTTTTAGTACAAGTAGCTACAGGGTTTGCTATGACTTTTTACTACCGCCCGACCGTTACTGAGGCTTTTGCCTCTGTTCAATACATAATGACGGAAGTTAACTTCGGGTGGTTAATTCGATCGGTTCATCGATGGTCGGCAAGTATGATGGTCCTAATGACAATCCTGCACGTATTTCGCGTGTATCTCACCGGGGGTTTTAAAAAACCCCGCGAATTGACTTGGGTTACAGGTGTGGTTCTGGCTGTATTGACCGCATCTTTTGGTGTAACAGGTTATTCTTTACCTTGGGACCAAATTGGATATTGGGCAGTAAAAATTGTAACAGGCGTGCCCGAAGCAATTCCGGTAATAGGATCGCCTTTGGTAGAGTTATTACGCGGAAGTGCTAGTGTGGGACAGTCCACTTTGACTCGTTTTTATAGTTTACACACTTTTGTATTACCTCTTCTTACTGCCGTATTTATGTTAATGCATTTCCTAATGATACGTAAACAAGGTATTTCTGGCCCTTTATAA

>petD

ATGGGAGTAACAAAAAAACCTGACTTGAATGATCCTGTATTAAGGGCTAAGTTGGCTAAGGGTATGGGTCATAATTATTATGGAGAACCCGCATGGCCCAACGATCTTTTATATATTTTTCCAGTAGTAATTCTAGGTACTATTGCATGTACCGTAGGCTTAGCGGTTCTAGAACCATCAATGATTGGTGAACCCGCGGATCCATTTGCAACTCCTTTGGAAATCTTACCCGAATGGTATTTCTTTCCCGTATTTCAAATACTTCGTACAGTACCCAATAAGCTGTTGGGTGTTCTTTTAATGGTTTCAGTACCTACGGGATTATTAACAGTACCTTTTTTGGAAAATGTTAATAAATTCCAAAATCCATTTCGCCGTCCCGTAGCGACAACCGTCTTTTTGATTGGTACCGCAGTGGCCTTGTGCTTGGGTATTGGAGCAACATTACCTATTGAAAAATCCCTAACTTTAGGTCTTTTTTAA

>rpoA

ATGGTTCGAGAGAAAGTAAAAGTATCTACTCGGACACTGCAGTGGAAGTGTGTTGAATCAAGAGCAGACAGTAAGCGTCTTTATTATGGGCGCTTTATTTTGTCTCCACTTATGAAAGGTCAAGCCGACACAATAGGCATTGCGATGCGAAGAGTTTTGCTTGGAGAAATAGAAGGAACATGTATTACACGCGCAAAATCTGAGAAAATCCCACACGAATATTCTACCATAGTGGGTATTCAAGAATCGGTACATGAAATTTTAATGAATTTGAAAGATATTGTATTGAGAAGTAATCTTTATGGAACTTGTGACGCGCTTATTTGTGTCAAAGGCCCGGGATATGTAACTGCTCAAGACATCCTCTTGCCGCCTTCTGTGGAAATCGTTGATAATACGCAGCACATAGCTAGCCTAACAGAACCAATTGATTTGTCTATTGGATTACAAATCGAGAGGAGTCGAGGATATAATATAAAAACGCCAAATACCTTTCAAGACGGAAATTGTTATCCTATCGATGCTGTATTCATGCCTGTTCGAAATGCGAATCATAGTATTCAGTCTTATGGGAATGGCAATGAAAAACAAGAGATCCTTTTTCTAGAAATATGGACAAACGGAAGTTTAACTCCTAAAGAAGCACTTCGTGAAGCCTCCCGGAGTTTGATTGATTTATTTATTCCCTTTCTCCAGGCAGAAGACGAAAACTTACCTTTAGAGAACAATCAATACAAGGTTACTTTACCCTTTTTTACTTTTCATGATAGATTGGCTAAACTAACGAAAAAGAAAAAAGAAATCGCATTGAAATCGATTTTTATTGACCAATCAGAAATGTCTCCCAGGATCTATAATTGTCTCAAAAAGTCCAATATACATACATTATTCGACCTTTTGAATACGCGTCAAGAAGACCTTATGAAAATTGAACACTTTCGCATAGAGGATGTAAAGCAGATAATGAGTATTCTAGAAAAGAAATAG

>rps11

ATGGCAAAATCTCCACCACGAAGTGGTTCACGTAGGCCGGGACGGATCGGTTCACGTAAAAGTGGACGTCGAATACCAAAGGGCGTTATTCATGTTCAAGCAAGTTTCAACAACACCATTGTGACTGTTACAGATGTCCGGGGTCGGGTAATTTCTTGGTCCTCGGCCGGTACTTGTGGATTCAGGGGTACAAGAAGAGGTACGCCTTTTGCTGCTCAAACCGCAGCAGGAAATGCTATTCGAGCAGTAGCGGATCAAGGTATGCAACGAGCAGAAGTCATGATAAAGGGTCCTGGTCTCGGAAGAGATGCGGCATTACGAGCTATTCGTAGAAGCGGTATCCTTTTAAATTTCGTACGGGATGTAACCCCTATGCCACACAATGGTTGCAGACCCCCTAAAAAAAGACGGGTGTAG

>rpl36

ATGAAAATAAGGGCTTCAGTTCGTAAAATTTGTGAAAAATGTCGACTGATCCGCAGGAGGGGACGGATTATAGTAATTTGTTCCAACCCGAGACATAAACAAAGACAAGGATAA

>rps8

ATGGGCAAGGACACTATTGCTGACATAATAACTTCTATACGAAATGCTGACATGAATAGAAAGGGAACGGTTCGAATAGCATCTACTAACATCACCGAAAACGTTGTTAAAATCCTTTTGCGAGAGGGTTTTATCGAAAACGCAAGGAAACTCGTGGAAAACAAAAAAGAGTTTTTGGTTTTAACCCTACGACATCGAAGGAATAGGAAAGGGCCGTATAGACCCATTTTAAATTTAAAACGAATCAGTCGACCCGGTCTACGAATCTATTTTAACTATCGACGAATTCCTAGAATTTTAGATGGGATGGGGATTGTAATTCTCTCTACTTCTCGGGGTATAATGACAGACCGAGCGGCTCGACTGGAAAGAATCGGTGGAGAAATTTTGTGTTATATATGGTAA

>rpl14

ATGATTCAACCTCAAACCCATTTGAATGTAGCAGACAATAGCGGTGCCCGAGAATTGATGTGTATTCGAATCATAGGAGCCAGTAATCGTAGATATGCTCATATTGGTGACGTTATTGTTGCTGTGATCAAGGAAGCAGTACCCAATACGCCTCTAGAAAGATCAGAAGTGATCAGAGCTGTAATTGTACGTACTTGTAAAGAACTCAGACGTGATAACGGTATGATAATACGGTACGATGACAATGCTGCAGTTGTCATTGATCACGAAGGAAATCCAAAGGGAACTCGAGTTTTTGGTGCGATCGCCCGGGAATTGAGACAGTTGAATTTTACTAAAATAGTTTCATTAGCACCTGAAGTATTATAA

>rpl16

ATGCTTAGTAACCCCAAAAGAACAAGATTCCGTAAACAACATAGAGGAAGAATGAAAGGGATATCTTATCGAGGTAATCATATTTGTTTCGGCAGATATGCTCTTCAAGCACTTGAACCCGCTTGGATCACATCTAGACAAATCGAAGCGGGGCGCCGCGCAATGACACGAAATGTACGGCGCGGTGGAAAAATATGGGTACGTATCTTTCCAGACAAACCAGTTACACTAAGACCCACGGAAACCCGTATGGGGTCCGGTAAAGGATCCCCCGAATATTGGGTAGCCGTCGTTAAACCGGGTAGAATACTTTATGAAATGAGTGGAGTAGCTGAAAATATAGCTCGAAAGGCTATTTCAATAGCGGCGTCCAAAATGCCTATAAGAACTCAATTCATTATTTCGGGATAG

>rps3

ATGGGACAAAAAATAAATCCACTAGGTTTCCGGCTTGGTACAACACAAAGTCATCATTCTCTTTGGTTTGCAAAACCAAAAAACTATTGCGAGGGTCTACAAGAAGATCAAAAAATACGAAACTTTATTAAGAATTATATAAAAAAAAATAGCAGAATATCTTCCGGTGTTGAGGGAATTGCACGGATAGAGATTCAAAAAAGAATTGATCTAATTCAAGTTATAATCTATATAGGGTTCCCAAAATTATTACTAGAAAATAGACCGCGAAGAATTGAAGAATTACAGATGAATGTACAAAAAGAACTTAATTGTGTGAATCGAAAAATAAACATTGCTATTACACGAATTACAAATCCTTATGGACACCCCAATATTCTTGCCGAATTTATAGCCGGACAATTAAAAAATCGAGTTTCTTTTCGAAAAGCAATGAAAAAAGCTATTGAATTAACGGAGCAGGCCGATACAAAAGGAATTCAAGTACAAATTGCAGGGCGTCTTGACGGAAAAGAAATTGCGCGCGCCGAATGGATCAGAGAAGGTAGAGTTCCTCTACAAACCATTGGAGCTAAAATTGATTATTGTTCCTATACGGTTCGAACTATATACGGGGTATTGGGAATCAAAATTTGGATATTTGTAGACGAAAAAAAATAA

>rpl22

ATGATAAGGATAATAAAGAAGAGGGTAGAAGTATCTGCTTTAGGTCAACATATATGTATGTCTGCTCACAAAGCCCGAAGGGTAATTGATCAGATTCGTGGACGTTCTTACGAGGAAACCCTTATGATACTCGAACTCATGCCTTATCGAGCATGTTATCCCATTTTAAAATTGGTTTATTCTGCAGCAGCAAATGGTATTCACAATCTGGGTTTCAACGAAGGAAGTTTATTCATTATTAAAGCCGAAGTAAACGAGGGTCCTACTGCGAAAAGATTAAAACCTCGAGCCCGAGGGCGGAGTTATCCGATCAAAAAACCCACTTGTCATATAACTATTGTTTTAAAAGATATATCCTTAGATGAATATGAATATAGAGACTATCTCGATCTCGAGTGCTCAAAAAACACTAGATTAATAAATAAAAAAAAAAAAATTAATAAATAA

>ndhF

ATGGAACATACATATCAATATTCCTGGATCATACCTTTAGTTCCACTTCCAGTCCCTATGTTAATAGGGGTGGGACTTCTATTTTTTCCGACCGCAACAAAAAATCTTCGCCGTATGTGGGCTTTTATTAGTATTTTATTGTTAAGTATAGTTATGATTTTTTCGATCGATCTATCTATTGAGCAAATAGATAGAACTTCGATCTATCAATCCCTAAGGAGTTGGACCATCACTAGTGATTTGTCTTTCGAGTTCGGATACTTTATTGATCCACTTACTTCTATTATGTCAATATTAATCACTACAGTTGGAATTCTGGTTCTTATTTATAGTGACAATTATATGTCTCATGATCAAGGATATTTGAGATTTTTTGCTTATATGAGTTTTTTCAATGCTTCAATGTTAGGATTAGTTACAAGTTCGAATTTCATACAAATTTATATTTTTTGGGAATTGGTTGGAATGTGCTCTTATCTATTAATCGGGTTTTGGTTCACACGACCTATTGCGGCAGGCGCCTGTCAAAAAGCATTTGTAACTAATCGTGTAGGGGATTTTGGATTATTATTAGGGATCTTAGGTCTTTATTGGCTAACAGGCAGTTTCGAATTTCGGGATTTGTTCGAAATATTGAAAAACTTGATTTATAATAATGAGGTTAACCTTTTATTTGTTACTTTGTGTGCATTTCTATTATTTGCCGGCCCGGTTGCTAAATCCGCGCAATTCCCCCTTCATGTATGGTTACCCGATGCCATGGAAGGGCCTACTCCTATTTCGGCTCTTATCCATGCTGCTACTATGGTAGCGGCGGGAATTTTTCTTGTAGCTCGGCTTCTTCCACTTTTCATAGTCATACCATACGCAATGAATCTAATATCTTTGATAGGGATAATAACAGTATTTTTAGGAGCTACTTTAGCTCTTGCTCAACAAGATATTAAGAGAGGTTTAGCTTATTCTACAATGTCTCAATTGGGTTATATGATGTTAGCTCTAGGTATGGGGTCTTATCGAGCCGCTTTATTTCATTTGATTACTCATGCCTATTCCAAAGCCTTGTTGTTTTTAGGATCCGGATCAATTATTCATTCAATGGAAGCTATTGTTGGATATTTTCCAGATAAAAGCCAGAATATGGTTCTTATGGGTGGGTTAAGAAAGCATGTGCCGATTACAAAAACCGCTTTTTTAGTAGGTACTCTTTCTCTTTGTGGTATTCCACCTCTCGCCTGTTTTTGGTCCAAGGATGAAATTCTTAATGATACTTGGTTGTATTCGCCGATTTTCGCAACAATAGCTTTTTTCACAGCTGGATTAACCGCATTTTATATGTTTCGAATTTATTTACTTACTTTTGAGGGGCCTTTCAACTTTTGCTTGCAAAATTACAGTGGCAAAAAAAGAAATTCCTTATATTCAATATCTCTATGGGGTAAAGAAGAACCAAAACCGATTAAAAACAAATTTCATTTAGTTGTTTTATTAACAATGAATAATAATAAAAGGGCTTCTTTTTTTGCGAAGAAGACTCATCGAATTGCTAGTACTGTAACAAATATGCCCTTTATTACTATTTTTCCTTTTGGCGCTGCCAAGACTTTTTGTTATCCTCACGAATCAGACAATACTATATTATTTGTTATGCTTGTATTAGTCCTATTTCCTTTGTTTGTTGGAGCGATAGGAATTCCTTTGAATCAAGAAGTAATCGAGTCGGATATTTTATCAAAATTGTTAACTCCGTCTATAAATCTGTTACATCAAAATTCAACTCATTTTGTTGATTGGTATGAAGGTGTAAAAAATCCAACCCTTTCCGTCAGTATAACGTATTTCGGAATACTTCTAGCCTACTTTTTATATAAACCCTTTTATTCATCTTTACACAATTGGAACATATTTAATTTTTTTGCTAAAAGAGGACCTAAGAGAATTCTTTGGGACAAAATACTCAATTTTCTATATGATTGGTCATATAATCGTGCTTATATAGATGCTTTTTACACAAGATCCTTAACAGAAGGGATAAGAGGATTAGCGGAACTAACTCATTTGTTCGACAGACGAGTAATTGATGGAATTACGAATGGGGTTGGTATTACAAGTTTTTTTGTAGGGGAAGGTATAAAATATTTAGGGGGAAGTCGCATCTCTTTTTATCTCTTATTATATTTATTTTCGTTATTACTCTTTTTAATATTTCTTTAA

>rpl32

ATGGCAGTTCCAAAAAAACGTACTTCTATATTAAAAAAACGTATTCGTAAAAATCTTTGGAAAAAAGGGGGGTACTGGGCAGCGTTGAAGGCTTTTTCGTTAGCGAAATCCCTTGCTACTGGGAATTCAAAAAGTTTTTTTTGTACAACAAATAAATAA

>ccsA

ATGATATTTTCAACTTTGGAGCGTATATTAACGCATATATCCTTTTCGGTCGTTTCAATTGGAATTACAATTTATTTAATAACCTTCTTAGTCGATGAAATCATAGGGCTATATGCTTCATCAGAAAGGGGGATGACAGCTACCGCTTTCTGTCTAACAGGATTATTAATCACCCGTTGGGTTTACTCGAGACATTTCCCATTAAGTGATTTATATGAATCATTAATCTTTCTTTCATGGAGTTTCTCTATTATTCATAAGATTTTTGATTTTAAAAATAATCAAAATCATTTAAGCGCTATAACGGCACCAAGTGCTTTTTTTACCCAAGGCTTTGCGACTTCGGGTTTTTTAACCAAAATGCATCAATCCAGAATATTAGTACCCGCTCTCCAAGTCCAGTGGTTAATGATGCACGTAAGTATGATGGTATTGGGCTATGCAGCTCTTTTATGTGGATCATTATTATCCACGGCTCTTCTAGTCATTACATTTCGAAAAGTGATAGGGCTTTTTTTGAAAAGAAAAAATTTTGTAAATGTAAATGGGTCATTTTGTTTCAGTGAAATCCAATACATGAACGAAAAAAAGAATGTTTTTCTAAATAGTTTTTCCGCTAGAAATTATTACAGGTCTCAAGTGATTCAACAATTGGATCGTTGGAGTTATCGTATTATTAGTTTAGGATTTATCTTTTTAACCACAGGTATTCTTTCGGGAGCAGTATGGGCTAATGAGGCGTGGGGGTCTTATTGGAATTGGGATCCAAAAGAAACTTGGGCATTTATTACTTGGACGATATTCGGGATTTATTTACATACTCGAACAAATACAAAATGGGAAGGTGTAAATTCCGCAATTGTGGCTTCTATGGGCTTTTTTATAATTTGGATATGCTATTTCGGAGTCAATCTATTAGGAATAGGGTTACATAGTTATGGTTCATTTAATTAA

>psaC

ATGTCACATTCAGTAAAGATTTATGATACATGTATAGGGTGTACTCAATGTGTACGAGCTTGCCCCACGGATGTATTAGAAATGATACCTTGGGACGGATGTAAAGCAAAGCAAATTGCTTCTGCTCCAAGAACAGAGGACTGTGTTGGTTGTAAGCGATGTGAATCCGCCTGTCCAACGGATTTCTTGAGTGTTCGAGTTTATTTATGGCATGAAACAACCCGAAGCATGGGTCTAGCTTATTGA

>ndhE

ATGATGCTCGAGCATGTACTTGTTTTGAGTGCCTATTTATTTTCGATTGGCATCTATGGCTTGATTACGAGCCGAAATATGGTTCGGGCCTTGATGTGCCTTGAACTTATACTAAATGCAGTTAATATCAATTTTGTAACATTCTCTGATTTTTTTGATAGTCGACAATTAAAAGGAGATATTTTTTCAATTTTTGTTATAGCTATTGCAGCCGCTGAAGCAGCTATCGGATCAGCTATTGTTTCGTCAATTTATCGTAACAGAAAATCGACGCGTATCAATCAATCGACTTTGTTGAATAAGTAG

>ndhG

ATGGATTTGCCTGGACCAATACACGATTTTCTTTTAGTTTTTCTGGGATCGGGTCTTATATTAGGAGGTCTGGGAGTGGTATTATTTACCAACCCAATTTATTCTGCCTTTTCCTTGGGATTGGTTCTTGTTTGTATATCATTATTCTATATTCTATCAAATTCCCATTTTGTAGCTGCCGCGCAACTCCTTATTTACGTGGGAGCTGTAAATGTTTTAATCATATTTGCTGTAATGTTCATGAACGGCTCAGACTATTCCAAAGATTTTCAGTTGAATCTTTGGACTATTGGCGATGGTCTTACTTCTCTGGTTTGTACAAGTATTTTTTTTTCGCTAATCACTACTATTCTCGATACATCGTGGTACGGGATTATTTGGACTACACGAGCCAACCAGATTATTGAACAAGATTTGATAAGTAATAGTCAACAAATTGGAATTCATTTATCAACAGACTTTTTTCTTCCATTTGAACTCGTTTCAATAATTCTTTTAGTTGCTTTAATAGGTGCAATTGCCGTGGCGCGTCAATAA

>ndhI

ATGTTCCCTATGGTAACCGGTTTCATGAATTATGGTCAACAAACAATACGAGCTGCAAGGTACATTGGTCAAAGTTTCATGATTACTTTATCCCAAGCAAATCGTTTACCTGTAACTATTCAATATCCTTATGAAAAATTAATCCCATCAGAGCGTTTTCGTGGTCGAATCCATTTTGAATTTGATAAATGTATTGCTTGTGAAGTATGCGTTCGGGTATGTCCTATAGATCTGCCTGTTGTTGATTGGAAATTTGAAACAGATATTCGAAAGAAACGATTGCTTAATTACAGTATTGATTTTGGAATTTGTATTTTTTGTGGTAACTGCGTTGAGTATTGTCCAACAAATTGTTTATCAATGACTGAAGAATATGAACTTGCGACTTACGACCGTCACGAATTGAATTATAATCAAATTGCTTTAGGTCGTTTACCAATGTCAGTAATTGACGATTTTACAATTCGAACAGTCTTGAATTCGCCTCAACGAAAAAACGTCTAA

>ndhA

ATGATAATTGCTACACCCGAAGTACAAGATATCAATTCTTTTTCCCGATTGGAATCCCTACAAGAGGTCTATGGGATCCTATGGGTGCTTGCCCCTATTTCGATTTATGTATTGGCAATCACAATAGGTGTCCTAGTAATTGTGTGGTTAGAAAGAGAAATATCTGCAGGAATACAACAGCGTATTGGGCCTGAATACGCCAGCCCTTTGGGAATTCTTCAAGCTTTAGCCGATGGGACAAAACTCCTTTTCAAAGAAAACCTTCTTCCATCTAGAGGAAATAGTAGTTTATTCAGTATTGGTCCATCTATAGCAGTCATAGCAATTCTACTAAGTTATTCAGTAATTCCTTTTAGTTATAACCTTGTTTTAGCTGACCTCAATATCGGTATTTTTTTATGGATTGCCATTTCAAGTATTGCCCCGATTGGACTTCTTATGTCAGGATATGGATCAAATAATAAATATTCCTTTTTAGGTGGTCTGCGAGCTGCTGCTCAATCGATTAGTTATGAAATACCATTAACTTTATGTGTTTTATCAATATCTCTATTATCTAACAGTTTAAGTACAGTTGATATAGTTGTGGCGCAATCAAAATATGGTTTTTGGGGGTGGAATTTGTGGCGTCAACCTATAGGGTTTATCGTTTTTCTAATTTCTTCCCTAGCGGAATGCGAGAGATTACCTTTTGATTTACCAGAAGCGGAAGAAGAATTAGTAGCCGGTTATCAAACCGAATATTCAGGAATCAAATTTGGTTTATTTTACGTTGCTTCCTATCTAAATCTACTAGTTTCCTCATTATTTGTAACAGTTCTTTACTTGGGAGGTTCGAATCTTTCCATTCCATACATATTTGTTCCTGGGCTGGTTGAAATAAATAAAGCGGATGGAATCTTTGGAACGACAATTGGTATCTTTATTACATTAGCTAAAACTTATTTGTTCTTGTTCATTCCTATTGCAACAAGGTGGACTTTACCGAGACTAAGAATGGACCAACTATTAAATCTTGGCTGGAAATTTCTTTTACCTATTTCTCTCGGTAATCTATTATTAACAACTTCTTCCCAACTCCTTTCGCTATAA

>ndhH

ATGAGTATACTAGCTACAGAAAAAGAAAAAGAATTTATGATAGTCAATATGGGACCTCACCACCCGTCAATGCATGGGGTTCTTCGTCTCATCGTTACTCTAGACGGTGAAGATGTTATTGACTGTGAACCAATATTGGGTTATTTACACAGAGGGATGGAAAAAATTGCGGAAAACCGAACAATTATACAATATCTGCCTTATGTAACCCGTTGGGATTATTTAGCTACTATGTTCACAGAAGCAATAACTGTAAATGGACCAGAACTGTTGGGAAATATTCGCGTACCTAAAAGGGCCAGCTATATCAGAGTAATTATGTTGGAGTTGAGTCGTATAGCTTCCCATCTGTTATGGCTTGGCCCTTTTATGGCAGATATTGGTGCACAGACTCCTTTCTTCTATATTTTCAGAGAAAGAGAATTAGTATATGATCTGTTCGAAGCTGCCACCGGTATGAGAATGATGCATAATTATTTTCGTATCGGAGGAGTAGCAGCTGATTTACCCTATGGTTGGATAGATAAATGTTTGGATTTCTGCGATTATTTTTTAACAGGGGTTGCTGAATATCAAAAACTTATTACGCGAAACCCCATTTTTTTAGAACGAGTTGAAGGAGTAGGCATTATTGGTGGAGAAGAAGCAATAAATTGGGGTTTATCAGGACCAATGCTACGAGCGTCTGGAATAGAATGGGATCTTCGTAAAGTTGATCATTATGAGTGTTATGACGAATTTGATTGGGAAGTCCAGTGGCAAAAAGAAGGGGATTCCTTAGCTCGTTATTTAGTCCGAATCGGTGAAATGACGGAATCTGTAAAAATAATTCAACAGGCTTTAGAAGGAATTCCGGGAGGCCCCTATGAAAATTTAGAAATCCGCTGCTTTGATAGAGAAAGCGATCCAGAACGGAATGATTTTGAAAATAGATTCATTAGTAAAAAGCCTTCTCCTACCTTTGAATTGACAAAACAAGAACTTTATGCGAGAGTAGAAGCCCCAAAAGGAGAATTGGGAATTTTTCTGATAGGGGATCAAAGTGGGTTTCCTTGGAGATGGAAAATTCGCCCACCGGGTTTTATCAATTTGCAAATTCTTCCTCAGTTAGTTAAAAGAATGAAATTGGCTGATATTATGACGATATTAGGTAGTATAGATATCATTATGGGGGAAGTTGATCGTTGA

>rps15

ATGGTAAAAAATGCATTCATCTCAGTTAGGGTTCAAGAAAAAAAAGAAAAAAACAGCGGATCGGTTGAATTTCAAGTATTTCGTTTCACCAACAAGATCCGGAGACTTACTTCACATTTAGAATTGCACAGAAAAGACTATTCATCTCAAAGGGGTCTACGTAAAATTTTGGAAAAACGCCAACGTCTACTAGCTTATTTGTCAAAGAAAAATAGAGTACGTTATAAAGAATTAATTAGTAAGTTGAATATCCGGGAGTCAAAAAATCGTTAA

>ycf1

ATGATTTTTCAATCTTTTATACTAGGTAATCTAGTATCCTTATGCATGAAGATAATCAATTCGGTCGTTGTGGTCGGACTCTATTATGGATTTATGACCACATTCTCCATAGGGCCCTCTTATCTCTTCCTTCTCCGAGCTCGGGTTATGGAAGAAGGAGAAGAAGGAACCGAGAAGAAGGTATCAGCAACAACTGGTTTTATTGCGGGACAGCTCATGATGTTCATATCGATCTATTATGCGCCTCTGCATCTAGCATTGGGTAGACCGCATACAATAACTGTCCTAGCTCTACCGTATCTTTTGTTTCATTTCTTCTGGAACAATCCCAAACACTTTTTTGATTATGGATCTACTACCAGAAATTCAATGCGTAATCTTAGCATTCAATGTGTATTCCTGAATAATCTCATTTTTCAATTATTCAACCATTTCCTTTTACCAAGTTCAATGTTAGCCAGATTAGTCAACATTTATATGTTTCGATGCAACAACAAGATGTTATTTGTAACAAGTAGTTTTGTTGGTTGGTTAATTGGTCACATTTTATTCATGAAATGGGTTGGATTGGTATTAGTTTGGATGCAGCAAAAGAATTCTATTAGGTCTAATGTACTTATTCGATTTAATAAGTACCTTGTGTCAGAATTGAGAAATTCTATGGCTCGAATCTTTAGTATTCTCTTATTTATTACCTGTATCTACTATTTAGGCAGAATACCCTCACCCATTTTTACTAAGAAACTGAAAGTGAAAGAAACCTCAGAAACGGAAGAAAGAGATGTAGAAATAGAAAAAACTTTCGAAAGGGGGGGGACTAAACAGGGACAAGAGGTATCCGCCGAAGAAGATCCTTCTCCTTCCCTTTTTTCGGAAGAAAAGGAGGATCCGGACAAAATCGAGGAAACGGAAGAGATCCGAGTGAATGGAAAGGAAAAAAAAAAAACAAAGCATGAATTCCACTTGCGCTTTAAAGAGACATGCGATAAAAATAGCCCTGTTTATGAAACTTCTTATCTGGATGGAAATCAAGAAAATTCGAAATTAGAAATATTTCAATTATTTAAAGAAAAAAAAGAAGAGAAATATTTATTATGGTTTGAAAAACCTCTTGTGACCCTTCTTTTCGACTATAAACGTTGGACTCGGCCACTTCGATATAAAAAAAATAATAGATTTGAAAATGCTGTACGAAACGAAATGTCACATTATTTTTTTTATGCATGTCGAAGTGATGGAAAAGAAAGGATCTCTTTTACATATCCAGCTAGTTTGTCAACTTTTTTGGACATGATAAAAAAAAATAATTATTTTTTCGCAACCGAAAAACTATCCTCTGATGAATTTTCTACACATTGGAATTACACTAATAACCAAAAAATTAAGAACTTAAGCAAGGAGTTTAGAAATCGACTTGAGGGTTTAGATAAAGGATCTCTTATTCGGGATATACTCGAAAAAAGGACTCAATTGTGTAATGATAAGACGAAAAAAAAATACTTACCTAAAACATATGATCCTTTATTAAACGGACCTTATCGTGGAAGAATCAACAAATTCTTTTCACCCCCAATCCTAAATACAACTTATATCAAAAATAAGATAAGAACGCCTTGGATAAATAAAATTCACAATCTAATTTTGATTAATGATTATCACGAATTTGAACAGACAATAGACCGATTTAATCCAAAATCATTTTCAAGCGAAGAAGTGCGGTCTTTATTGACAGAACGGGAACGAGAACACATCGATTCCGAAGACCGAATAAAAAGTTTCAATTTTTTATTCGATGCAGTTATAACGGATCCCAATGATCAAAAAATTAGAAAAAAAGCGATAAAAGAAATTAGTAAAAGAGTTCCCCGGTGGTCATACAAATTAATCGATAATGTATACCAAGAACTGGGAGAATACGACGAAAATGTAAGAAGGAAACATGCATTTCGTTCACGAAAAGCCAAACGTTTAGTGGTTGCTGTTGATCACCAGACAAAAGAGGATGTGACTTTGCCCCATTATTTGGAACAATCGGATTTTCGTCGATATATAATAAAAGGTTCCATGCGCGCACAAAGACGTAAAACCGTTATTTGGAAACCACTTCAAGCAAATGCCCATTCCCCTCTTTTTTTGGACAGAATAGACAAAACCCTTTATTTGTCTTTTGATATTTCCCAGCTGATGAAAGTAATTCTTCGAAATTGGATGGTAAAAAATAAAAACCAAAAACTTTCTGATTATACAAACGCAAAGACAAGAAAATTGGACAAAAAAGAAAAAAAGAAGCTCAAAGGCGAAAGATACCAAAGACAAGCAATGGTACGTATAAAACAAGCAGAAAGCTGGGATAAGCGTTTACTTACTCGAATACTAAGAAGTTCTATGTTAGTAATCCAATCGATTCTTAGAAAATATATTGTATTACCGTTATTGATAATAGCTAAAAATGTGGTTCGCATACTATTATTCCAAGATCCCGAGTGGTCCGAGGATTTTAAGGATTGGAATCGTGAAATTTATGTTAAATGCACTTATAGTGGTGTTAATTTATCTGAAACAGAATTTCCGAAAAACTGGTTAATAGAAGGTATTCAGCTAAAGATCCTATTCCCCTTTCACCTGAAACCCTGGCACGGATCTACGATACAATCCTCTCATAAAGATCCAAAAAGTGAACACGAAACTGATTTTTGTTTTTTAACAATTTTGGGGCTGGAAACTGACATGCCGTTCGGTTCTCCCCAAAAACGACGTTCCTTTTTTGAACCCATTTTTAAAGAACTAAAAAAAAAAATTCGAAAATTGAAAACTAAGTCTTTTATAGTTTTAAGGGATTTTAAAGAAGGAAAAATAAAAGAACTTTCAAAAATAAACTTGAGAGAAATCGAGAAATTGAGGGAAACTCAAAAAAATTCGATAATCAGTAAGCAGATGATTCACGAACCGTCTATTGAAATTTCATCTATGGATTGGACGAAATTATCCCGGACTGAAAAAAAAATGAAAGATCTGACTAATAGAACAAGCAGAATCCGAAATAAAATATATAAAATTACAAAAGAAAAGAAAAAAGGATCGCTAACTCAAGAAACAAATATTAGTTCGAACAAACCAACTTATTCTGTTAAAAGATTAGACCCATCAAAAAGGATTTGGCGGATATTAAAAAAAAGAAACGCTCGATTAATCCGTAAATCCTATTTGTTTCTAAAATTTGGCATTGAAAAGATATACAGAAATATTTTTATATCTACCCTTACTATTCCAAGAATCAATACAAAACCTTTTCGTGAATCAACAAGAAAACAAATGAAAATTATTGAGAAAAACATCCACAATAATGAAGCAAATCCCGAAAGAATTAATAAAACAAATAAAAATAGAATTATTTCGACTATCCAAAAATCGATTTCTAAGACTAGTAATAAGAATTCAAAGATTTCTTGTAATTTATTGTCTTTTTCACAATCACAAGCATATGTATTTTACAAATTATTACAAGTCCCAATTTTGAACTTTTATAATTTAAGACCCGTTCTTCAATATCACGGAACATCTCTATTTCTTAAGAATGAAATAAAAGATTTTTTTGAAAAACACGGAATCTTTAATTACCAATTAAGACATAAACCCCTTTGGAATTTTGGAAGGAATACACGAAAACACTGTTTAAGCGGGCATTATCAATATGATTTATCTCGGATTAAATGGGCTAGATTAGTACCACAAGAATGGCGAAATAGAGCCAATCAACACTGTATGGCTCAAAATAAAGATTTAATTAAAAGAGATTCGTATGAAAAAAATGGATTAACTCATTACGAAAAACAACATTTTTTTGAAGCAGACCTATTACGTAATCAAAAATCGAATTTTAAAAAACACTATAGATATGATCTTTTATCATATAAATCGCTTAATTATGAAGATAAGAAAGACTCGTATATTGATAGATCACTAGTCCAAGTAAATAATAAAGAAGAGTATTATTCTAATTACAATAGAAAGAAAGTAAAATTGTTGGCTATGCTGGGAAGTATCTCTATCAATAATTATATAGGGGAAGATTATATTATGGATATGGAAAAATTCTTGTATAGAAAATATTTTGATTGGAGAATTCTTAATTTTTGTCTTAGAAATAAGGCCAATATGGAAGCCTGGGTTGATATGGATACTGGTACCAGCAGTAATCAAAATACTAGGATTGGGTCCAATAATTATCAAAAAATTAATGCAATTAATAACAGAGTCCCCTTTTATCTTAGAATTCATCAAGATGAAGAAATTAACCCATCCACTCAAAAGGGGTTCTTTTGTGATTGGATGGGAATGAATGAAGAAATACTAAGTTGTCCTATATCAAACCCAGAATCTTGGTTCTTCCCAGAATTTGTACTACTTTTTAATGCATATAGAACGAAACCCTGGATTATACCAATCAAATTACTTCTTTTCAATTTTAATGGAAATAGTAAGAAAAATAGAACCGGAAAGAAAGAGGCGGATCTTTTTATATCACCTACTCAAAAAGAATATTTGGAATTATCGAATCAAAGTAAAGAAGAAAACGAACTCGCAGACCAAGGAACTCCGAGATCGGATGCACAAAAGCAAGTAATTCTTGGATCAGTTCTCTCAAACCAAGAAAAAGATGGTGAAGAAAATTATACGGGATCGGACATGAAAACCCGTATAAAGAAAAAGCAATCCAAAAGAGAAACCGAAGTACAGCTCGATTTATTCCTAAAAAGATATTTGTGTTTGCAGTTGCGATGGAGGGGTGCTGTTTCTTTCAGAGAAAAAATACTCAATGATATGAAAGTATATTGTCAGCTGGTTCGACTAATAAATCCTAGCGACGTTACTATAGCCTCTATTCAAGGGGGAGAAATGAGTCTGCCTATTTTGATCACTAAGAAGAATTTCGATCTTAAAGAATTGACGAAAGGGGGAATGCTTATTATCGAACCCCGTCGTTTGTCTGTAAAAAATGATGGACAATTTTTTCTATATCAAATCGTAGGTATTGCATTGGTTCATAAGAATAAGCGCAAAATTACTAAAAGATACCAAGAAAAGGGCTATGTTGATAAAAAAGATTTTGATGAATTCATTGCAAAACATCAAAAAATGACTGGAAATATAAACAAAAATCATTATGATTTGCTTGTTCCTGAAACTATTTTACTCCCTAAACGTCGTAGAGAATTAAGAACCCTAATTTGTTTCAATTCGAAGAATCAAAATGGTATGCAGAAAAATCCAGTATTTTTTAATAACGGAAAGGGCGGCGGCCGCGTTTTGGATAAAAACAAAAATCTTGCTCGAGAGAAAAATCAACTAATTCAATTAAAGTTCTTTCTTTGGGCCAATTCTCGATTAGAAGATTTAATTTGTATGAATCGGTATTGGTTTAATACCAATAATGGGAGTCGTTTCAGTATGGTAAGGGTCCATATGTATCCACGATTGAAAATTCGTTAA

>rps7

ATGTCACGTCGAGGTACTACAGAAGAAAAAACTGCAAAATCCGATCCAATTTATCGTAATCGATTAGTTAACATGTTGGTTAACCGTATTCTGAAACACGGAAAAAAATCATTGGCTTATCAAATTATCTATCGAGCCTTGAAAAAGATTCAACAAAAGACAGAAAAAAATCCACTATCTGTTTTACGTCAAGCAATACGTGGAGTAACTCCCGATATAGCAGTAAAAGCAAGACGTGTAGGCGGATCGACTCATCAAGTTCCCATTGAAATAGGATCCGCACAAGGAAAAGCACTTGCCGTTCGTTGGTTATTAGGGGCATCCCGAAAACGTCCGGGTCGAAATATGGCTTTCAAATTAAGTTCCGAATTAGTGGATGCTGCCAAAGGGAGTGGCGATGCCATACGCAAAAAGGAAGAGACTCATAGAATGGCAGAGGCAAATAGAGCTTTTGCACATTTTCGTTAA

>ndhB

ATGATCTGGCATGTACAGAATGAAAACTTCATTCTCGATTCTACGAGAATTTTTATGAAAGCCTTTCATTTGCTTCTCTTCGATGGAAGTTTTATTTTCCCAGAATGTATCCTAATTTTTGGCCTAATTCTTCTTCTGATGATCGATTCAACCTCTGATCAAAAAGATATACCTTGGTTATATTTCATCTCTTCAACAAGTTTAGTAATGAGCATAACGGCCCTATTGTTCCGCTGGAGAGAAGAACCTATGATTAGCTTTTCGGGAAATTTCCAAACGAACAATTTCAACGAAATCTTTCAATTTCTTATTTTACTATGTTCAACTCTATGTATTCCTCTATCCGTGGAGTACATTGAATGTACAGAAATGGCTATAACAGAGTTTCTGTTATTCGTATTAACAGCTACTCTAGGAGGAATGTTTTTATGCGGTGCTAACGATTTAATAACTATCTTTGTAGCTCCAGAATGTTTCAGTTTATGCTCCTACCTATTATCTGGATATACCAAGAAAGACGTACGGTCTAATGAGGCTACTATGAAATATTTACTCATGGGTGGGGCAAGCTCTTCTATTCTGGTTCATGGTTTCTCTTGGCTATATGGTTCATCCGGGGGCGAGATCGAGCTTCAAGAAATAGTGAATGGTCTTATCAATACACAAATGTATAACTCCCCGGGAATTTCAATTGCGCTTATATTCATCACTGTAGGAATTGGGTTCAAGCTTTCCCTAGCCCCTTCTCATCAATGGACTCCTGACGTATACGAAGGATCTCCCACTCCAGTCGTTGCTTTTCTTTCTGTTACTTCGAAAGTAGCTGCTTCAGCTTCAGCCACTCGAATTTTCGATATTCCTTTTTATTTCTCATCAAACGAATGGCATCTTCTTCTGGAAATCCTAGCTATTCTTAGCATGATATTGGGGAATCTCATTGCTATTACTCAAACAAGCATGAAACGTATGCTTGCATATTCGTCCATAGGTCAAATCGGATATGTAATTATTGGAATAATTGTTGGAGACTCAAATGGTGGATATGCGAGCATGATAACTTATATGCTGTTCTATATCTCCATGAATCTAGGAACTTTTGCTTGCATTGTATTATTTGGTCTACGTACCGGAACTGATAACATTCGAGATTATGCAGGATTATACACAAAAGATCCTTTTTTGGCTCTCTCTTTAGCTCTATGTCTCTTATCCCTAGGAGGTCTTCCTCCACTAGCAGGTTTTTTCGGAAAACTCCATTTATTCTGGTGTGGATGGCAGGCAGGCCTATATTTCTTGGTTTCAATAGGACTCCTTACGAGCGTTGTTTCTATCTACTATTATCTAAAAATAATCAAGTTATTAATGACTGGACGAAAGCAAGAAATAACCCCTCACGTGCGAAATTATAGAGGATCCCCTTTAAGATCAAACAATTCCATCGAATTGAGTATGATTGTATGTGTGATAGCATCTACTATACTAGGAATATCAATGAACCCGATTATTGCAATTGCTCAGGATACCCTTTTTTAG

>ycf15

ATGCTACTGCTGAAACATAGAAGAATTGAAATCTTAGATCAAAACACTATGTATGGATGGTATGAACTGCTTAAACAAGAATTCTTGAACAGCGAACCACCAGAGCTATTACTAACTACATCAAAAAATTTCCATTAA

>ycf2

ATGAAAGGACATCAATTCAAATCCTGGATTTTCGAATTGAGAGAGATATTGAGAGAGATCAAGAATTCTCACTATTTCTTAGATTCATGGACCCAATTCAATTCAGTGGGATCTTTCATTCACATTTTTTTCCACCAAGAACGTTTTATAAAACTCTTGGATCCACGAATTTGGAGTATCCTACTTTCACGCAATTCACAGGGTTCAACAAGCAATCGATATTTCACGATCAAGTGTGTAGTACTGTTTGTAGTAGCGATCCTTATATATCGTATTAACAATCGAAATATGGTCGAAAGAAAAAATCTCTATTTGACAGGGCTTCTTCCTATACCTATGAATTCCATTGGACCCAGAACTGATACATTGGAAGAATCTTTTGGGTCTTCCAATATCAATAGGTTGATTGTTTCGCTCCTGTATCTTCCAAAAGGAAAAAAGATCTCTGAGAGCTTTTTCCTGGATCCGAAAGAGAGTACTTGGGTTCTCCCAATAACTAAAAGGTGTATCATGCCTGAATCTAACTGGGGTTCGCGGTGGTGGAGGAACTGGCTCGGAAAAAAGAGGGATTCTAGTTGTAAGATATCTAATGAAACCGTCGCTGGAATTGAGATCTCATTCAAAGAGAAAGATCTCAAATATCTGGAGTTTCTTTTTGTATATTATATGGATGATCCGATCCGCAAGGACCATGATTGGGAATTGTTTGATCGTCTTTCTCCGAGTAAGAGGCGAAACATAATCAACTTGAATTCGGGACATCTATTCGAAATCTTAGTGAAAGACTGGATTTGTTATCTCATGTTTGCTTTTCGTGAAAAAATACCAATTGAAGTGGGGGGTTTCCTCAAACAACAAGGGGCTGGGTCAACTATTCAATCAAATGATATTGAGCGTTTTTCCCATCTCTTCTTGAGAAACAAGTGGGCTATTTCTTTGCAAAATTGTGCTCAATTTCATATGTGGCAATTCCGCCAAGATCTCTTCGTTAGTTGGGGGAAGAATCCGCACGAATCGGATTTTTTGAGGAACATAGCGAGAGAGAATTGGATTTGGTTAGACAATGTGTGGTTGGTAAACAAGGATCGATTTTTTACCAAGGTACGGAATGTATCGTCAAATATTCAATATGATTCCACAAGATCTAGTTTCGTTCAAGTAACGGATTCTAGCCAATTGAAAGGATCTTCTGATCAATCCAGAGATCATTTCGATTCCATTAGTAATGAGGATTCGGAATATCACACATTGATCAATCAAAGAGAGATTCAACAACTAAAAGAAAGATCGATTCTTTGGGATCCTTCTTTTCTTCAAGCGGAACGAAGAGAGATAGAATCAGACCGATTCCCTAAATGTCTTTCTGGATATTCCTCAATGTCCCGGCTATTCACGGAACGTGAAAGGCAGATGAATAAGCATCTGCTTCCGGAAGAAATCGAAGAATTTCTTGGGAATCCTGCAAGATCCATTCGTTCTTTTTTCTCTGACAGATGGTCAGAACTTCATCTGGGTTCGAATCCTACTGAGAGGTCCACTAGAGATCAGAAATTGTTGAAGAAAGAAGAGGATGTTTCTTTTGTCCCTTCCAGGCGATCGGAAAATAAAGAAATAGTTAATATATTCAAGATAATTACGTATTTACAAAATACCGTCTCAATTCATCCTATTTCATCAGATCCGGGATGTGATATGGTTCCGAAGGATGAACTGGATATGGACAGTTCCAATAAGATTTCATTCTTAAACAAAAATCCATTTTTTGATTTATTTCATCTATTCCATGACCGGAACAGGGGGGGATCCACGTTACACCACGATTTTGAATCAGAAGAGAGATTTCAAGAAATGGCAGATCTATTCACTCTATCAATAACCGAGCCGGATCTGGTGTATCATAAGGGATTTGCCTTTTCTATTTTTTCCTACGGATTGGAGCAAAAACAATTCTTGAATGAGGTATTCAACTCCAGGAATGAATCGAAAAAGAAATCTTTATTGGTTCTACCTCCTATTTTTTATGAAGAGAATGAATCTTTTTATCGAAGGATCAGAAAAAAATGGGTCCGGATCTCCTGCGGGAATGATTTGGAAGATCCAAAACCAAAAATAGTGGTATTTGCTAGCAACAACATAATGGAGGCAGTCAATCAATCTAGATTGATCCGAAATCGGATTCAAATCCAATATAGCACCTATGGGTACATAAGAAATGTATTGAATCGATTCTTTTTAATGAATAGATCCGATCGCAACTTCGAATATGGAATTCACAGGGATCAAATAGGAAATGATACTCTGAATCATAGAACTATAATGAAATATACGATCAACCAACATTTATCGAATTTGAAAAAGAGTCAGAAGAAATGGTTCGCTCCTCTGATTTTGATTTCTCGAACCGAGAGATTCATGAATCGGGATCCTAATGCATATAGATACAAATGGTCCAATGGGAGCAAGAATTTCCAGGAGCATTTGAAACATTTCGTTTCTGAGCAGAAGAGCCGTTTTCAAGTAGTGTTCGATCGATTACGTATTAATCAATATTCGATTGATTGGTCTGAAGTTATCGACAAAAAAGATTTGTCTAAGCCACTTCCTTTCTTTTTGTCCAAGTTTCTTTTCTTTTTGTCTAACTCACTTCCTTTTTTCTTTGTGAGTTTCGGGAATATCCCCATTCATAGGTCTGAGATCCACATCTATGAATTGAAAGGTCCGAATGATCAACTCTGCAATCAGTTGTTAGAATCAATAGGTCTTCAAATCGTTCATTTGAAAAAATTGAAACCCTTCTTATTGGATGATCATAATACTTCCCAAAAATCGAAATTCTTGATCAATGGAGGAAGAATATCACCATTTTTGTTCAATAAGATACCGAAGTGGATGATTGACTCATTCCATACTAGAAAAAATCGCAGGAAATCTTTTGATAACACGGATTCCTATTTCTCAATGATATCCCACGATCAAGACAATTGGCTGAATCCTGTGAAACCATTTCATAGAAGTTCATTGATATCTTCTTTTTATAAAGCAAATCGACTTCGATTCTTGAATAATCCACATCGCTTCCGCTTCTATTGTAACAAAAGATTCACTTTTTATGTGGAAAAGGTCCGTATCAATAATTATGATTTTACGTATGGACAATTCCTCAATATCTTGTTCATTCGCAACAAAATATTTTCTTTGTGCGGCGGTAAAAAAAAACATGCTTTTTTGGAGAGAGATACTATTTCACCAATCGAGTCACAGGTATCTAACATATTCATACCTAACGATTTTCCACAAAGCGGTGACGAAAGGTATAACTTGTACAAATTTTTCCCTTTTCCAATTCGATCCGATCTATTAGTTCGTAGAGCTATTTACTCGATCGCAGCCATTTCTGGAACACCTCTAACAGAGGGACAAATAGTCAATTTTGAAAGAACTTATTGTCAACCTCTTTCAGATATGAATCGATCTGATTCAGACGAGAAGAACTTGCATCAGTATCTCAATTTCAATTCAAACATGGGTTTGATTCACACTCCATGTTCTGAGAAATATTTACCATCCGAAAAGAGAAAGAGGAAAAAACGGAGTCTTTGTCTAAAGAAATGCGTTGAGAAAGGGCAGATGTCTAGAACCTTTCAACGAGACATTTCAACTCTCTCAAAATGGAATCGATTCCAAACATATATGCCATGGTTCCTTACTTCGACAGGGTACAAATATCTAAATTTGATATTTTTAGATACTTTTTCAGACCTATTGCCGGTACTAAGTAGCAGTCAAAAATTTGTATCCATTTTTCATGATATTATGCATGGATCAGATAGAGCATGGCGAATTCTTCAGAAAAAATGGTGTCTTCCACAATGGAATCTGATAAGTGAGATTTCGAGTAAGTGTTTCCATAATCTTCTTCTGTCCGAAGAAATGATTCATCGAAATAATGAGTCACCATTGATATCGACACATCTGAGATCGCCAAATGCTCGGGAGTTCCTCTATTCAATCCTTTTCCTTCTTCTTGTTGCTGGATATCTCGTTCATACACATCTTCTCTTTGTTTCCCGAGCCTATAGTGAGTTAGAGACAGAGTTCGAAAGGGTCAAATCTTTGATGATTCCACCATACATGATTGAGTTGCGAAAACTTCTGGATAGGTATCCCACATCTGAACTGAATTCTTTCTGGTTAAAGAATCTCTTTCGAGTTGCTCTGGAACAATTAGGAGATTCTCTAGAAGAAATACGGGGTTCTGCTTTTGGCGGCAACATGCTATGGGGTGGTGGTCCCGCGGATGGGGTTAAATCAATACGTTCTAAGACGAAAGATTTGAATATCAATCTCGTCGATATCACCGATCTCATAAGTATCATACCAAATCCCATCAACCGAATCACTTTTTCGAGAAATACGAGACATCTAAGTCATACAAGTAAAGAGATCTATTCATTGATAAGAAAAAGAAAAAAGGGGAACGGTGATTGGATTGATGATAAAATAGAATCCTGGGTCGCGAACAGTGATTCGATTGATGATAAAGAAAGAGAATTCTTGGTTCAGTTCTCCACCTTAACCTTAACGACAGAAAAAAGGATTGATCAAATTCTATCGAGTCTGACTCATAGTGATCATTTATCAAAGAATGACTCTGGTTATCAAATGATTGAACAACCGGGAACAATTTACTTACGATACTTAGTTGACATTCATAAAAAGCATTTCATGAATTATGAGTTCAATACATACTGTTTAGCAGAAAGACGGATATTCCTTGCTCATTATCAGACAATCACTTATTCACAAACTTCGTGTGGGGCTAATAGTTTTCATTTCCCATCTCATGGAAAACCCTTTTCGCTCCGCCTAGCCCTATCCCCCTCTAGGGGTATTTTAGTGATAGGTTCTATAGGAACCGGACGCTCCTATTTGGTCAAATACCTAGCGGCAAACTCCTATGTTCCTTTCATTACAGTATTTCTGAACAAGTTCCTGGATAACAAGCCTAAAGGTTTTCTTATTGATGATATCGATGATGATAGTGACGATATTGATGCTAGTGACGATATTGATGCTAGTGACGATATCGATCGTGACCTTGATACGGAGCTGGAGCTTCTAACTATGATGAATGCGCTAACTATCGATATGATGTCGGAAATAGGCCGATTTTATATCACCCTTCAATTCGAATTAGCAAAAGCAATGTCTCCTTGCATAATATGGATTCCAAACATTCATGATCTGGATGTGAATGAGTCGAATTACTTATCCCTCGGTCTATTAGTGAACTATCTCTCCAGGGATTGTGAAAGATGTTCCACTAGAAATATTCTTGTTATTGCTTCGACTCATATTCCCCAAAAAGTGGATCCCGCTCTAATAGCCCCGAATAAATTAAATACATGCATTAAGATACGAAGGCTTCTTATTCCACAACAACGAAAGCACTTTTTCACTCTTTCATATACTAGGGGATTTCACTTGGAAAAGAAAATGTTCCATACTAATGGATTCGGGTCCATAACCATGGGTTCCAATGCACGAGATCTTGTAGCACTTACCAATGAGGCCCTATCGATTAGTATTACACAGAAGAAATCAATTATAGACACTAATACAATTAGATCTGCTCTTCATAGACAAACTTGGGATTTGCGATCCCAGGTAAGATCGGTTCAAGATCATGGGATCCTTTTCTATCAGATAGGGAGGGCTGTTGCACAAAATGTACTTCTAAGTAATTGCCCCATAGATCCTATATCTATCTATATGAAGAAGAAATCGTGTAACGAAGGGGATTCTTATTTGTACAAATGGTACTTCGAACTTGGAACGAGCATGAAGAAATTAACGATACTTCTTTATCTTTTGAGTTGTTCTGCCGGATCGGTCGCTCAAGACCTTTGGTCTCTACCCGGACCCGATGAAAAAAACGGGATCACTTCTTCTGGACTCGTTGAGAATGATTCTGATCTAGTCCATGGCCTATTAGAAGTAGAAGGCGCTCTGGTGGGATCCTCGCGGACAGAAAAAGATTGCAGTCGGTTTGATAATGATCGAGTGACATTGCTTCTTCGGCCCGAACCAAGGAATCCCTTAGATATGATGCAAAAAGGATCTTGTTCTATCGTTGATCAGAGATTTCTCTATGAAAAATACGAATCGGAGTTTGAAGAAGGGGAAGGAGAAGGAGTCCTCGACCCGCAACAGATAGAGGAGGATTTATTCAATCACATAGTCTGGGCTCCTAGAATATGGCGCCCCTGGGGCTTTCTATTTGATTGTATCGAAAGGCCCAATGAATTGGGATTTCCCTATCGGGCCGGGTCATTTCGGGGCAAGCGGATCATTTATGATGAAAAGGATGAGCTTCAAGAGAATGATTCGGAGTTCTTGCAGAGTAGAACCATGCAGTACCAGACACGAGATAGATCTTCCAACGAACAAGGCTTTTTTCGAATAAGCCAATTCATTTGGGAACCTGCGGATCCACTCTTTTTCCTATTCAAAGATCAGCCCCTTGTCTCTGTGTTTTCACACCGAGAATTCTTTGCAGATGAAGAGATGTCAAAGGGGCTTCTTACTTCCCAACCAGATCCTCCTACATCTATATATAAACGCTGGTTTATCAAGAATACGCAAGAAAAGCACTTCGAATTGTTGATTCATCGCCAGAGATGGCTTAGAACCAATAGTTCATTATCTAATGGATTTTTCCGTTCTAATACTCCATCCGAGAGTTATCAGTATTTATCAAATCTGTTCCTATCTAACGGAACGCTATTGGATCAAATGACAAAGACATTGTTGAGAAAAAGATGGCTTTTTCCGGATGAAATGAAAATTGGATTCATGTAA

>rpl23

ATGGATGGAATCAAATATGCAGTATTTACAGACAAAAGTATTCGGTTATTGGGAAAAAATCAATATACTTCTAATGTCGAATCAGGATCAACTAGGACAGAAATAAAGCATTGGGTCGAACTCTTCTTTGGTGTCAAGGTAATAGCTATGAATAGTCATCAACTCCCCCGAAAGGGTAGAAGAATGGGACCTATTATGGCACATACAATGCATTACAGACGTATGATCATTACGCTTCAACCGGGTTATTCTATTCCACCTCTTAGAAAGAAAAGAACTTAA

>rpl2

ATGGCGATACATTTATACAAAACTTCTACCCCGAGCACACGCAATGGAGCCGTAGACAGTCAAGTGAAATCCAATCCACGAAATAATTTGATCTATGGACAGCATCGTTGTGGTAAAGGTCGTAATGCCAGAGGAATCATTACCGCAGGGCATAGAGGGGGAGGTCATAAGCGTCTATACCGTAAAATCGATTTTCGACGGAATGAAAAAGACATATATGGTAGAATCGTAACCATAGAATACGACCCTAATCGAAATGCATACATTTGTCTCATACACTATGGGGATGGTGAGAAGAGATATATTTTACATCCCAGAGGGGCTATAATTGGAGATACCATTGTTTCTGGTACAGAAGTTCCTATAAAAATGGGAAATGCCCTACCTTTGACCGATATGCCCTTAGGCACGGCCATACATAACATAGAAATCACACTTGGAAAGGGTGGACAATTAGCTAGAGCAGCGGGTGCTGTAGCGAAACTGATTGCAAAAGAGGGGAAATCGGCCACATTAAAATTACCTTCTGGGGAGGTCCGTTTGATATCCAAAAACTGCTCAGCAACAGTCGGACAAGTGGGGAATGTTGGGGCGAACCAGAAAAGTTTGGGTAGAGCCGGATCTAAATGTTGGCTAGGGAAGCGTCCTGTAGTAAGAGGAGTAGTTATGAACCCTGTAGACCACCCCCATGGGGGTGGTGAAGGAAGGGCTCCGATTGGTAGAAAAAGACCCGCAACCCCTTGGGGTTATCCTGCACTTGGAAGACGAAGTAGAAAAAGGAATAAATATAGTGATAATTTGATTCTTCGCCGCCGTACTAAATAG

***C. trifoliata***

>psbA

ATGACTGCAATTTTAGAGAGACGCGAAAGCGAACGCCTATGGGGTCGCTTCTGTAACTGGATAACTAGCACTGAAAACCGCCTTTACATTGGTTGGTTTGGTGTTTTGATGATCCCTACTTTATTGACCGCAACTTCTGTATTTATTATCGCCTTCATTGCTGCTCCTCCAGTAGATATTGACGGTATTCGTGAACCTGTTTCTGGATCTCTACTTTATGGAAACAATATTATTTCTGGTGCGATTATTCCTACTTCTGCAGCTATAGGTTTGCATTTTTACCCGATATGGGAAGCGGCATCCGTTGATGAATGGTTATACAATGGCGGTCCTTATGAGCTAATTGTTCTACACTTCTTACTTGGTGTAGCTTGTTACATGGGTCGTGAGTGGGAACTTAGTTTCCGTCTGGGTATGCGTCCTTGGATTGCTGTTGCATATTCAGCTCCTGTTGCAGCAGCGACTGCTGTTTTCTTGATCTACCCAATCGGTCAAGGAAGTTTTTCTGATGGTATGCCTCTAGGAATCTCTGGTACTTTCAACTTCATGATTGTATTCCAGGCTGAGCACAATATCCTTATGCACCCATTCCACATGTTAGGCGTAGCTGGTGTATTCGGCGGCTCCCTATTCAGTGCTATGCATGGTTCCTTGGTAACCTCTAGTTTGATCAGGGAAACCACAGAAAATGAATCTGCTAATGCAGGTTACAGATTCGGTCAAGAGGAAGAAACTTATAATATCGTAGCTGCTCACGGTTATTTTGGCCGATTGATCTTCCAATATGCTAGTTTCAACAATTCTCGTTCTTTACATTTCTTCCTGGCTGCTTGGCCTGTAGTAGGTATCTGGTTCACTGCTTTAGGTATTAGCACTATGGCTTTCAACTTAAATGGTTTCAATTTCAACCAATCTGTAGTTGATAGTCAAGGTCGTGTAATTAATACCTGGGCTGATATTATTAATCGTGCTAACCTTGGTATGGAAGTTATGCATGAACGTAATGCTCATAACTTCCCTCTAGACCTAGCTGCTATTGAAGCCCCATCTACAAATGGGTAA

>matK

ATGGAGGAATTTCAAGTATATTTAGAACTAGATAGATCTCAACAACACGACTTCCTATACCCACTTCTTTTTCGGGAGTATATTTATGTACTTGCTCATGATCATGGTTTAAATAGCTCGATGATGTCATTGGAAGGTGGGTTTTATGACAATAAATCTAGTTCACTAAGTGTGAAACGGTTAATTACTAGAATGTATCAACGGATTAATTTGAGTATTGCTGCTAATGATTCGAATCAAAATCCGATTTTTGGGCACAACAATAAGTTATATTCTCAAATTATATCAGAGGTATTTGCTGCTGTGGTGGAAATTCCATTTTCCCTACGGTTGGTGGCTTTTTTAGAAGGGAACGAAATTGAAAAATCGCCTAATTTCCAATCAATTCATTCAATATTTCCTTTTTTCGAGGATAAATTGTCCCATTTAAATTATGTGTTAGATGTACGAATACCCTACCCCATTTGTCCCGAAATCTTGGTTCAAACCCTTCGCGAATGGGTAAAGGATGCCTCTTCTTTACATTTATTACGTTTCTTTCTCCACGAGTATTTTAATTCGAACAGTCTTATTACTCCAAAGAACTCTATTTCTGTTTTTTTAAAAAGTAATCCAAGATTGTTATTGTTTCTATATAATTCTCATGTATATGAATATGAATCCATCCTCTTTTTTCTCTGTAACCAATCGTCTCATTTACAATCAACATCCTTTCGAGTCCTCGTTGAGCGAACGTATTTCTATGGAAAAGTCGAACATCTTGTCGAAGTCTTTGCGAAAGATTTTCAGGACATCTTAGGGTTGGTCAAGGATCCTTTCATGCATTATGTTAGATATCAAGGAAAATCCATTTTGGCTTCAAAGGATACGCCTCTTCTGATGAATAAATGGAAATATTACCTTGTCGGTTTATGGCAATGGCATTTTCACGCGTCTTCTCAACCAGGAAGGGTTCAGCTAAACCACTTATACTTAGGCAAGTACGCTATTAACTTTCTGGGCTATCTTTCCGGTGTGCGACTAAATTCTTTGTTGGTACGGAGTCAAATGCTAGAAAATTCATTTCTAATAGATAATTCTATGAAGAAGGTCGATACGACCGTTCCAATTATTCATCTGATTGGATCATTGACTAAGGCGCGGTTTTGTAACGCATTAGGGCATCCTATCAGTAAGTCGACTTGGTCCGATTTCTCTGATTCTCATCTTATCGACCGATTTGTGCGTATATGTAGAAATCTTTCTCATTATTACAGCGGATCTTCAAAAAAAAAAAGTTTGTATCGAGTAAAATATATACTTCGGCTTTCTTGTGTTAAAAGTTTGGTTCGTAAACATAAAAGTACTGTACGCGCTTTTTTAAAAAGATTAGGTTCGGAATTATTGGAAGAATTCCTTATGGAGGAAGAACACGTTCTTGCTTTACTCTTTCCAAGAGCTTCGTCTACTTCGCGTAGGTTCTATTTATATAGAGGACGGATTTGGTATTTGGATATTTTTTGTATCAACGATCTGGTTAATTATCAATGA

>rps16

ATGGTAAAACTTCGTTTGAAACGATGTGGTAGAAAGCAACGAGCCGTTTATCGAATCGTTGCAATTGATGGTCGATCCCGAAGAGAAGGAAGAGATCTTCGGAAAGTGGGTTTTTATGATCCGATAAATAATCAAACCCATTTAAATGTTCCTGCTATTCTATATTTCCTTGCCAAGGGCGCTCAACCTACAGGAACCGTTCATGATATTTCACAGAAAGCGGGGGTTTTTACAGAAGTTAGTCTTAATCAAACGAAATTTTATTAA

>psbK

ATGCTTAATATCTTTAGTTTAATGTATATCTGTCTTAATTCTGCCCTTTATTCGAGTAGTTTTTTATTCGCTAAATTACCCGAGGCCTACGCTTTTTTGAATCCAATTGTAGATGTTATGCCAGTAATACCTCTTCTATTTTTTCTCTTAGCCTTTGTTTGGCAAGCTGCTGTAAGTTTTCGATAA

>psbI

ATGCTTACTCTCAAACTCTTTGTTTACACCGTAGTGATATTCTTTGTTTCGCTCTTCATCTTCGGATTCCTGTCTAATGATCCAGGGCGTAATCCCGGACGCGAAGAATAA

>atpA

ATGGCAACAATTAAAGCCGACGAAATTAGTAATATTATCCGCGAACGTATTGAGCAATATAATAGAGAAGTAAAGATTGTAAATATTGGGACCGTACTTCAAGTAGGCGACGGCATCGCCCGTATTTATGGTCTTGATGAAGTAATGGCAGGTGAATTAGTAGAATTTGAAGAGGGTACAATAGGCATTGCTCTGAATTTGGAATCAACTAATGTTGGTGTTGTTTTAATGGGTGACGGTTTAATGATACAAGAAGGAAGTTCTGTAAAAGCAACCGGAAAAATTGCTCAGATACCAGTAAGCGAGGCTTATTTAGGTCGTGTTATAAATGCCCTGGCTAAACCTATTGATGGTCGAGGTGAAATTCCAGCTTCTGAATCTCGATTAATTGAATCCCCTGCTCCGGGGATTATTTCGAGACGTTCCGTATATGAGCCTCTTCAAACAGGACTTATTGCTATTGATTCGATGATCCCTATCGGACGCGGTCAGCGAGAATTAATTATTGGGGACAGACAGACCGGTAAAACGGCAGTAGCCACGGATACGATTCTCAACCAACAAGGGCAAAATGTAATATGTGTTTATGTAGCTATTGGTCAAAAAGCATCTTCTGTGGCTCAGGTAGTGAATACTTTCCAGGAGAGGGGGGCAATGGACTACACTATTGTGGTAGCCGAAACGGCGGATTCCCCCGCTACGTTACAATACCTCGCTCCTTATACGGGCGCAGCTCTAGCTGAATATTTTATGTACCGTCAACGACACACTTTAATCATTTATGATGATCCCTCCAAACAAGCACAGGCTTATCGACAAATGTCTCTTCTATTACGAAGACCACCGGGTCGCGAAGCTTATCCCGGAGATGTTTTTTATTTGCATTCACGACTTTTGGAAAGAGCCGCGAAATTAGGTTCGCAGTTAGGCGAAGGAAGTATGACCGCTTTACCAATAGTTGAGACCCAGTCGGGAGATGTTTCGGCTTATATTCCTACTAATGTAATTTCCATTACAGACGGGCAAATATTCTTATCCGCGGATTTATTCAATGCTGGAATCAGACCCGCTATTAATGTGGGTATTTCCGTCTCGCGAGTAGGATCCGCAGCTCAAATTAAAGCTATGAAACAAGTAGCCGGCAAATTAAAATTGGAATTGGCACAATTCGCAGAATTAGAAGCCTTTGCACAATTTGCTTCTGATCTCGATAAAGCTACTCAGAACCAATTGGCAAGGGGTCAACGCTTACGTGAGTTGCTCAAACAGGCCCAAGCAGCCCCTCTCACGGTGGAAGAACAGATAATGACTATTTATACAGGAACGAATGGTTATCTTGATTCATTAGAAATTGGTCAAGTAAGAAAATTTCTCGTTGAGTTACGTACTTACTTAAAAACGAATAAACCTCAGTTCCAAGAAATAATATCTTCTACCAAGATATTCACCGAAGAAGCAGAAGCCCTTTTGCAAGAAGCTATTCAGGAACAAAAGGAACGTTTTCTACTTCAGGAACAATTATAA

>atpF

ATGAAAAATGTAACCGATTCTTTCGTTTCTTTGGTTAACTGGCCATTCGCCGGGAGTTTCGGGCTTAATACCGATATTTTAGCAACAAATCCAATAAATCTAAGTGTAGTGCTTGGTGTATTGATCTTTTTTGGAAAGGGAGTGTTAAGTGATTTATTAGATAATCGCAAACTGAGGATTTTGAATAGTATTCGAAATTCAGAAGAACTGCAGAGAGGGGCCATTGAACGGCTGGAAAAAGCCCGGGCCCGGTTACGAAAAATAGAAATAGAAGCAGATCAGTTTCGAGTGAATGGATACTCTGAGATAGAACGAGAAAAATTCAATTTGATTAATTCAACTTATAAGACTTTGGATCAATTAGAAAATTACAAAAATGAAACCATTCATTTTGAACAACAAAGAACAATTAATCAAGTCCGACAACGGGTTTTCCAACAAGCTTTACAAGGAGCGCTCGGAACTCTGAATAGTTGTTTGAACAAGGAGTTACATTTACGTACCATTAGTGCCAATATTGGCATGTTTGGAGCGATGAAAGAAATAACTGATTAG

>atpH

ATGAATCCACTGATTTCTGCCGCTTCCGTTATTGCTGCTGGGTTGGCTGTTGGGCTTGCTTCTATTGGACCTGGAGTTGGTCAAGGTACTGCTGCGGGCCAGGCAGTAGAAGGGATCGCGAGACAGCCCGAGGCGGAGGGAAAAATACGAGGTACTTTATTGCTTAGTCTGGCTTTTATGGAAGCTTTAACAATTTATGGGCTGGTTGTAGCATTAGCACTTTTATTTGCGAATCCTTTTGTTTAA

>atpI

ATGAATGTTCTATCATGTTCCATGAATACACTAAGGGGGTTATACGATATATCCGGTGTGGAAGTAGGCCAACATTTCTATTGGCAAATAGGGGGGTTCCAGGTCCATGCCCAAGTACTTATTACTTCTTGGGTTGTAATTGCTATCTTATTAGGTTCAGCCTTTATAGCCGTTCGGAATCCACAAACCGTTCCGACTGCCACTCAAAATTTCTTCGAATATGTCCTTGAATTCATTCGAGACGTGAGCAAAACTCAGATTGGAGAAGAATATGGCCCATGGGTTCCCTTTATTGGAACTCTGTTTCTTTTTATTTTTGTTTCTAATTGGTCAGGTGCTCTTTTACCTTGGAAAATCATAGAGTTACCTCATGGGGAGTTAGCCGCACCCACGAATGATATAAATACTACCGTTGCTTTAGCTTTGCTCACGTCAATAGCATACTTCTATGCGGGTCTTTCCAAAAAGGGATTAGGCTATTTCAGTAAATACATTCAACCGACTCCAATTCTTTTACCCATTAACATTTTAGAAGATTTCACAAAACCTCTATCACTTAGTTTTCGACTTTTTGGGAATATATTAGCCGATGAATTAGTAGTTGTTGTTCTTGTTTCTTTAGTACCTTTAGTGGTTCCTATACCCGTCATGTTCCTTGGATTATTTACAAGCGGTATTCAAGCTCTTATTTTTGCAACTTTAGCTGCGGCTTATATAGGCGAATCTATGGAGGGACATCATTGA

>rps2

ATGGCAAGAAGGTATTGGAACATCCATTTGGAAGAGATGATGGAAGCAGGAATTCATTTTGGTCATGGTACTCGGAAATGGAATCCTAGAATGGCACCTTATATATCTGCAAAACATAAAGGTATTCATATTACAAATCTGACTCGAACTGCTCGGTTTTTATCAGAAGCTTGTGATTTAGTTTTTGATGCAGCAAGTAGGGGAAAACAATTCTTAATTGTTGGTACTAAAAATAAAGCAGCTGATTTAGTCGCGCGAGCTGCAATAAGGGCTCGGTGCCATTATGTTAATCAAAAATGGCTCGGCGGTATGTTAACCAATTGGTCCACTACAGAAACGCGACTTCACAAGTTCAGGGATTTGAGAACGGAACAAAAAGGGGGGAGACTCGACAGTCTTCCCAAAAGGGATGCCGCTATTTTGAAGAGACAATTATCGCGTCTGCAAACGTATCTGGGCGGGATTAAATATATGACGAGGGTACCCGATATTGTAATCGTCGTTGATCAGCAAGAAGAATATACGGCTCTTCGAGAATGTATCACTTTGGGAATTCCAACAATTTGTTTAATCGATACAAATTGTGACCCCGATCTCGCAGATATTTCGATTCCAGCAAACGATGACGCTATAGCCTCAATCCGATTAATTCTTAACAAATTAGTATTCGCAATTTGTGAGGGTCGCTCTAGCTATATACGAAATCCTTGA

>rpoC2

ATGGCAGAACGAGGGAGTCTGGTCTTTCACAATAAAATGATAGATGGAACTGCCATTAAACGACTTATTAGCAGGTTAATAGATCACTTCGGAATGGCATATACATCACACATCCTGGATCAAGTAAAGACCCTGGGGTTCCAGCAAGCCACTGCTACATCTATTTCATTAGGCATTGATGATCTTTTAACGATACCTTCTAAGCGATGGCTAGTCCAAGATGCTGAACAACAAAGTTTTATTTTGGAAAAACACTATCATTATGGGAATGTACACGCGATAGAAAAACTACGACAATCCATTGAGATATGGTATGCTACAAGTGAATATTTGCGACAAGAAATGAATCCTAATTTTAGGATGACTGAGCCTTTTAATCCAGTCCATATAATGTCTTTTTCGGGGGCTAGAGGAAATGCATCTCAAGTACACCAATTGGTGGGTATGAGAGGATTAATGTCTGATCCCCAAGGGCAAATGATTGATTTACCCATTCAAAGTAATTTACGCGAAGGGCTTTCTTTAACAGAATATATCATTTCTTGCTATGGAGCCCGCAAGGGGGTTGTCGATACCGCTGTCCGAACATCAGATGCTGGATATCTTACGCGCAGACTTGTTGAAGTAGTTCAACACATTATTGTACGTAGAACAGATTGTGGCACTGTCCGAGGAATTTCTGTGAGTCCTCAAAATCAAAATAGGATGATGTCGGAAAGGGTTTTTAGCCAAACATTAATTGGTCGTGTATTAGCGGACGATATATATATGGGCCAGCGATCCATTGCCATTAGAAATCAAGATATTGGGATTGGACTTGTCAATCGACTCATAACCCTTCGAACACAAGCAATATCTATTCGAACCCCCTTTACTTGTAGGAGTACATCGTGGATCTGTCGATTATGTTATGGTCGGAGTCCGACTCATGGTGACCTGGTTGAATTGGGGGAAGCCGTAGGTATTATTGCGGGTCAATCTATTGGAGAACCAGGGACTCAACTAACATTAAGAACTTTTCATACCGGCGGCGTATTTACGGGGGGCACTGCAGAACATGTACGAGCCCCTTCTAATGGTAAAATCAAATTCAATGAGGATTTGGTTCATCCCACGCGCACACGTCACGGGCATCCGGCTTTTCTATGTTCTATAGATTTGGATGTAATTATTGAGGGTGAAGATATTATGCACAATGTGACTATTCCACCAAAAAGTTTTCTTTTAGTTCAAAATGATCAATATGTCGAATCAGAACAAGTGATTGCTGAGATTCGGGCGGGAGCATACACTTTGAATTTTAAAGAGAGGGTTCGAAAACATATTTATTCTGATTCAGAGGGAGAAATGCACTGGAGTACTGATGTGTACCATGCACCCGAATTTACATATAGTAATGTCCACCTCTTGCCAAAAACAAGCCATTTATGGATATTGTCGGGGGGTTCACGCAGATCTAGTGTAGTTTCTTTTTCACTCCACAAGGATCAAGATCAAATGAATATTCATTCTCTTTCTGTCGAACGAAGAGAGATTTCTAGCCTCTCGCTCTCAGTGAATAATGATCAAACGGGACACAAATTTTTTAGTTCTGATTTTTCTGCTAAAAAAAAAGGTCGAATTCTTGAGATGTCTGATTATTCGGGATTTAATAGAATCATAGGTACTGGTCATTGTAATCTCATCCATCCTGCAATTCTCCACGCAAATTCGGATTTATTGGCAAAAAGGCAAAGAAATGGATTTCTTATTCCATTCCACTCGATTCAAGAGCAAGAGAAAGAGCTAATGCCCCATTCAGGTATCTCGAGTGAAATACCCGTAAGGGGTATTTTCCGTAGAAATAGTATTCTTGCTTATTTCGACGATCCTCGATACAGAAGAAAGAGTTCCGGAATTACTAAATGGGGGACTCTGGGGGCGCATTCAATCGTTAAAAAAGAGGACTTGATTGAGTATCGAGGACTCAAAAAAATTAAGCCAAAATACCAAATGAAAATAGATCGCCTTTTTTTCATTCCGGAGGAAGTGCATATTTTTCCCGAATCTTCTTACCTAATGGTACGGAATAATAGTATCATTGGAGTAGACACACAAATCACTTTAAATATACGAAGCCGGGTGGGCGGATTGGTCCGAATGGAGAGAAAAAGGGGAGGGGTTGAACTAAAAATATTTTCGGGAGATATCCATTTTCCCGGAGAGATAGATAAGATATCCCGACACAGTGGCATCCTGATACCGCCAGAAAGTGAAAAAAAAAAACTTAAGGAAGCCACTAAGGAATCAAAAAAATTGAAAAAGTGGATCTATGTTCAACGGATCACACCTACAAAGAAAAAGTCTTTTGTTTTGGTTCGACCCGTAGTCACATATGAAATAGCGGACGGTATAAATTTAGCAACACTCTTTCCCCAGGATCCGCTGCGGGAAAAGGATAATATGCAATTTCGAGTTGTCAATTATGTCCTTTATGGGAAGGGCAAAGCCGCTGGGGGAATTTCTGATACAAGTCTTCAATTAGTTCGGACGTGTTTAGTGTTGAATTGGGACCAAGACAACAAAAGTTCTTCCGTCGAAGAGGTTTGTGCTTCCTTTGTTGAAGTACGTACAAATGGTCTGATTCGAGATTTCCTAAGAATCAACTTAGTGAAATCCAATATTTCGTATCTCAGAAAAAGGGATCATCCGTCGGGTTCAGGATTAATTTCTGATAATGGTTCAGCTCGCACCAATAGCAATCCGTTTTATTCCGTGTTTGGCAAGGCAGGGGTTGAACAATCGCTTAGACAAAATCAAGGAACTATTCGTACGTTGTTGAATAAAAATAAGGAATGCCAAGTTTTGATAATTTTATCATCATCTAATTATTTTCGAATGGGTCCATTGAACGATGTCAAATATCACAATGTGATAAAACAATCAATTCCAATTCAAAAAGATTCGCTAACTCCAATTAAGACTTCGTTGGGACCCTTAGGAACATTCCTTCAAATTGCGAATTTTTATTCATTTTACTATTTAATAACTCATAATCATATCTCGGTAACTAAATATTTGAAACTTGACAATTTAAAACAGCCTTTTCAAGTACTTAAATATTATTTAATGGACGAAACCGGGGAAATTTATAATCCTGATACAGATAGTAAGATCCTTTTGAATCCATTTAATTTGAATTGGTATTTTCTCCAGCCTAATTATTGTGAGGAAATGTCCCCGATAATTAGTCTTGGGCAGTTTCTTTGTGAAAATGTAGGTATAACCAAAAGGGGACCATACCTAAAATCCGGTCAAGTTTTAATTGTTCAAGTTAACTCTGTAGTAATACGATCAGCTAAGCCTTATTTGGCTACTCCTGGAGCAACTGTTCATGGGCATTATGGAGCAATCCTTTACGAAGGGGATACATTAGTTACATTTATATATGAAAAATCGAGATCTGGTGATATAACGCAGGGTCTTCCAAAAGTAGAACAGGTGTTAGAAGTGCGTTCGCTTGATTCAATATCGATGAACCTAGAAAAGAGAGTTGAGGGTTGGAACGCGAGTATAACAAGAATTCTTGGGATTCCCTGGGGATTCTTGATTGGTGCTGAGCTAACTATAGTGCAAAGTCGTATCTCTTTGGTTAATAAGATCCAAAAGGTTTATCGATCGCAGGGGGTGCAGATCCATAATAGGCATATAGAAATTATTGTACGTCAAATAACATCAAAAGTTTTAGTTTCCGAAGATGGAATGTCTAATATTTTTTTACCCGGCGAACTTATTGGATTGTTACGAGCGGAACGAATGGGGCGCGCTTTGGAAGAAGTGATCTGTTATCGAGCTATCTTATTGGGAATAACGAGAGCGTCTCTGAATACTCAAAGTTTTATATCCGAAGCAAGTTTTCAAGAAACCACGCGCGTTTTAGCAAAAGCTGCTCTCCGAGGTCGTATCGATTGGTTGAAAGGCCTGAAAGAAAACGTTGTTCTGGGGGGGATAATACCAGTCGGTACCGGATTCAAAGGATTAGTCCACTGTTCAAGGCAGCATAACAACATTCTTTTGGAAAGACAAAAAGGGAATTTATTCGGGGGGGAAATGAGAGATATTTTCTTACACCACAGAGAATTATTTGACTCGTGCATTTCAACGACTTTCCATGATACATCAGAGCACAATTGCTTAGAGGGTTTAATGAGTCCTAGGAGCGAATTCTTTTAA

>rpoC1

ATGATTGATCGATATAAACATCAACAACTCCGAATTGGATCAGTTTCTCCTCAACAAATACGTGCTTGGGCCAATAAAATCCTACCTAATGGAGAGATAATTGGAGAAGTGACAAAACCCTATACTTTTCATTACAAAACCAATAAACCGGAAAAAGATGGATTATTTTGTGAAAGAATTTTTGGGCCTATTAAAAGTGGAATTTGTGCTTGTGGAAATTATCGAATAATCGGAGATGAAAAAGAAGACCCGCAATTTTGTGAACAATGTGGGGTTGAATTTGTTGATTCTCGGATACGAAGATATCAAATGGGATACATAAAACTGGGATGCCCAGTAACCCACGTGTGGTATTTGAAACGTCTTCCTAGTTATATCGCGAATCTTTTAGATAAACCTCTTAAAGAATTAGAAGGCCTAGTATATTGCGATTTTTCTTTTGCTAGGCCTATAGCGAAAAAACCTACTTTTTTACGATTACGCGGTTCATTCGAATATGAAATACAATCCTGGAAATACAGCATCCCACTTTTTTTTACTACCCAAGGTTTCGATAAATTTCGTAACCGAGAAATTTCTACTGGAGCAGTTGCTATCCGGGAACAATTAGCCGATCTAGATTTGCGAATTATTCTAGATAATTCCTTGTTAGAATGGAAAGAATTAGGGGAAGAAGGGCCCGCCGGTAATGACTGGGAAGATCGAAAAATTGGACGAAGAAGGGATTTTTTGGTTAGACGCATGGAATTAGCTAAGCATTTTCTTCGAACAAATATAGAACCGGAGTGGATGGTTTTATGTCTATTACCTGTTCTTCCTCCCGAGTTGAGGCCTATCATTCAAGTAGATGGAGGTAAACTAATGAGTTCAGATATTAATGAACTCTATAGAAGAGTTATCTATCGGAACAATACTCTTATCGACCTATTAACAACAAGTAGATCTACGCCAGGGGAATTAGTAATGTGTCAGGAGAAATTGGTACAAGAAGCCGTGGATACGCTTCTTGATAATGGAATCCGTGGACAGCCAATGAGGGATGGTCATAATAAGATTTATAAGTCGTTTTCAGATGTAATTGAAGGAAAAGAGGGAAGATTTCGTGAGACTCTGCTTGGCAAACGGGTCGATTATTCGGGGCGGTCTGTCATTGTTGTGGGCCCCTCACTTTCATTACATCAATGTGGATTGCCTCGCGAAATCGCAATAGAGCTTTTCCAGACTTTTGTAATTTGTGGGCTAATTAGACAACACCTTGCTTCGAACATAGGAGTTGCTAAGAGTAAAATTCGGGAAAAAGGGCCGATTATCTGGGAAATACTGCAGGAAGTTATGCAGGGACATCCAGTATTGCTGAATAGAGCGCCTACTCTGCATAGATTGGGCGTACAGGCATTCCAGCCCATTTTAGTGGAAGGGCGCGCTATTTGTTTACATCCATTAGTTTGTAAGGGATTCAACGCAGACTTTGATGGGGATCAAATGGCTGTTCATGTACCCTTATCTTTAGAGGCTCAAGCAGAGGCTCGTTTACTTATGTTTTCTCATATGAATCTCTTGTCTCCTACTATTGGAGATCCCATTTCCATACCGACTCAAGATATGCTTATTGGGCTCTATGTATTAACGAGCGGGAATCGTCGAGGTATTTGTGCAAATAGGTATAATACATGGAATCGAATAAATTATCCAGATGAAAGAATTGACGATAATAGCTATAAGTATACGAAAGAACCCCTTTTTTGTAATTCCTATGATGCAATTGGAGCTTATCGGCAGAAAAGAATCAATTTAGATAGTCCGTTGTGGCTTCGGTGGCGATTAGATCAACGCCTTATTGCTTCAAGGGAAGCTCCCATCGAAGTTCACTATGAATCTTTGGGTACCTCGCATGAGATTTATGGGCATTATCTAATAGTACGAAGTGTAAAAAAAGAAATTCTTTCTATATACATTCGAACCACCGTGGGCCATATTTCTCTTTATCGAGAAATCGAAGAAGCTATACAAGGGTTTTGCCGGGCCTGCTCATATGGTACCTAA

>rpoB

ATGCTCGGAGATGGAAATGCAGGAATGTCTACAATACCTGGGTTGAATCAGATACAATTTGAAGGATTTTGTAGGTTCATTGATCAGGGCTTAACAGAAGAGCTTTATAAGTTTCCAAAAATTGAAGATACAGATCAAGAAATTGAATTTCAATTATTTGTGGAAACATATCAATTGGTAGAACCCTTGCTAAAAGAAAGAGATGCTGTATATGAATCATTCACGTATTCTTCTGAATTATATGTATCCGCAGGATTAATTTGGAAAAGCCGAGGGGACATGCAGGAACAAACTATTTTTATTGGAAACATTCCTCTAATGAATTCTTTGGGAACTTCTATAGTAAACGGAATATACAGAATTGTCATCAATCAAATATTGCAAAGTCCCGGTATCTATTATCGGTCAGAATTGGGCCATAACGGAATTTCGGTCTATACAGGCACCATAATATCTGATTGGGGGGGAAGATTAGAATTAGAGATTGATAGAAAAGCAAGGATATGGGCTCGTGTGAGTAGGAAACAGAAAGTATCTATTCTAGTTCTATCAGCAGCTATGGGTTCGAATCTACGAGAAATTCTAGAGAATATTTGCTACCCTGAAATTTTCTTGTCTTTCCTGACGAATAAGGAGAAAAAAAAAATTGGATCAAAAGAAAATGCCATTTTGGAGTTTTATCAACAATTTGCTTGTGTAGGCGGAGATCCGGTATTTTCGGAATCCTTATGTAAGGAATTACAAAAGAAATTTTTTCACCAAAGATGTGAATTAGGAAAGATTGGTAGACGAAATATGAACCAGAGACTGAATCTGAATATACCTCAGAACAATACATTTTTGTTACCACGAGATGTATTGGCAGCTGTCGATCATTTGATTGGACTGAAATTTGGAATGGGTACACTTGACGATATGAATCATTTGAAAAATAAACGTATTCGGTCTGTCGCGAATCTTTTACAAGATCAATTCGGATTGGCCCTGGTTCGGTTAGAAAATGTGATTAGAGGAACTATATGTGGAGCAATTAGGCATAAATTGATGCCAACTCCTCAAAATTTGGTAACTTCAACTCCCTTAACAACCACTTATGATTCTTTTTTCGGATTACATCCATTATCTCAAGTTTTGGATCGAACTAATCCATTGACACAAATAGTTCATGGGAGAAAATTGAGTTATTTGGGCCCCGGAGGATTGACAGGGCGAACTGCGAGTTTTCGGGTACGAGATATCCATCCTAGTCACTATGGCCGCATTTGTCCAATTGACACGTCCGAAGGAATCAATGTTGGACTTATTGGATCCTTAGCAATTCATGCGAGAATTGGTTATTGGGGGTCTCTAGAAAGCCCGTTTTATGAAATCTTTGAAAAATCAAAAAAAGTACGGATGCTTTATTTATCACCAAGTAGAGATGAATACTATATGGTAGCGGCAGGAAATTCTTTGGCACTGAATCAGGGTAGTCCGGAAGAACAGGTTGTTCCGACTCGATACCGTCAAGAGTTCCTGACTATTGCGTGGGAACAGGTTCATCTTCGAAGTATTTTTCCCTTCCAATATTTTTCTATTGGGGCTTCCCTCATTCCTTTTATCGAGCATAATGATGCGAATCGGGCTTTAATGAGTTCTAATATGCAACGCCAAGCAGTTCCGCTCGTTCGGTCCGAGAAGTGCATTGTTGGAACTGGGTTGGAACCTCAAGTGGCTCTAGATTCAGGGGTTCCCGCTATAGCCGAACACGAGGGAAAGATCATTTATACCGATATTGACAAGATTGTTTTATCGGGAAACGGGAATACATATAGTATCCCATTAATTATGTATCAACGTTCAAACAAAAATACTTGTATGCATCAAAAACCCCAGGTTGGGCGGGGTAAATGCATTAAAAAGGGCCAAGTTTTAGCGGATGGTGCCGCTACAGTTGGCGGCGAACTCGCTTTAGGGAAAAACATATTAGTAGCTTATATGCCATGGGAAGGCTACAATTTTGAGGATGCGGTACTTATTAGCGAACGTCTGATATATAGAGATATTTATACTTCTTTTCACATACGGAAATACGAAATTCAGACTCATGTGACAAGTCAAGGTCCCGAAAGGATCACTAATGAAATACCACATCTAGAAGCCCGGTTACTCCGCAATTTAGACAAAAATGGAATTGTGATGCTGGGATCTTGGGTAGAGACCGGCGATATTTTAGTAGGTAAATTAACGCCTCAGGCGGCGAAAGAATCATCGTATGCTCCGGAAGATAGATTATTACGGGCCATACTTGGCATTCAGGTATCCACTTCAAAGGAAACTTGCCTAAAACTACCTATAGGTGGTAGGGGTCGCGTTATTGATGTGAGATGGGTCCAGAAAAAGGGGGGTTCTAGTTATAATCCAGAAACAATTTGTGTATATATTTCACAGAAACGTGAAATCAAAGTAGGTGATAAAGTAGCTGGAAGACATGGAAATAAGGGTATCGTTTCAAAAATTTTGCCTAGACAGGATATGCCTTATTTGCAAGATGGAAGGCCTGTTGATATGGTTTTCAACCCACTAGGAGTACCCTCGCGAATGAATGTAGGACAGATATTTGAATGCTCGCTCGGGTTAGCGGGGGGTCTGCTAAATCGACATTATCGAATAGCACCTTTTGATGAGAGATATGAACAAGAGGCTTCGAGAAAACTCGTGTTTTCTGAATTATATGAAGCCAGTAAGCAAACCGCGAATCCATGGGTATTTGAGCCGGAATACCCGGGAAAAAGCAGAATATTTGATGGACGAACGGGAGATCCTTTTGAAGAACCTGTTCTAATAGGAAAGCCTTATATCTTGAAATTAATTCATCAAGTTGATGATAAAGTACACGGACGTTCCAGTGGGCATTATGCACTTGTTACCCAACAACCCCTTAGAGGAAGGTCCAAACAAGGGGGACAACGGGTAGGCGAAATGGAGGTTTGGGCTCTAGAGGGCTTTGGTGTTGCTCATATTTTACAAGAGATGCTTACTTATAAATCTGATCATATTAGAGCGCGCCAAGAAGTACTTGGTACTACAATCATTGGAGAAACAATACCTAACCCGGAAGATGCTCCAGAATCTTTTCGATTGCTCGTTCGAGAACTACGATCTTTGGCTTTGGAACTGAATCATTTTCTTGTATCTGAGAAAAACTTCCAGATTAATAAGAAGGAAGCTTAA

>psbM

ATGGAAGTAAATATTCTCGCATTTATTGCTACTACACTGTTCGTTCTAGTTCCTACTGCGTTTTTGCTTATAATATACGTAAAAACGGTCAGTCAAAGTGATTAA

>psbD

ATGACTATAGCCCTTGGTAAATTTACCAAAGAGGAAAAGGATTTATTTGATATTATGGATGACTGGTTACGGAGGGACCGATTCGTTTTTGTAGGTTGGTCTGGCCTATTGCTCTTTCCTTGTGCCTATTTCGCTTTAGGGGGTTGGTTCACAGGCACAACCTTTGTAACTTCATGGTATACCCATGGATTAGCCAGTTCCTATTTGGAAGGCTGCAACTTCTTAACCGCCGCAGTTTCGACTCCTGCTAATAGTTTAGCGCATTCTTTGTTGTTACTATGGGGTCCTGAAGCACAAGGAGATTTTACTCGCTGGTGTCAATTAGGCGGTCTTTGGACTTTTGTTGCTCTCCACGGTGCTTTCGGACTAATAGGTTTCATGTTACGTCAATTTGAACTTGCTCGCTCTGTTCAATTGCGACCTTATAATGCAATAGCATTCTCTGCTCCAATTGCTGTTTTTGTTTCTGTATTCCTGATTTATCCACTAGGTCAGTCTGGTTGGTTCTTTGCGCCTAGTTTTGGTGTAGCGGCGATATTTCGATTCATCCTTTTTTTCCAAGGGTTTCATAATTGGACATTGAACCCCTTTCATATGATGGGAGTTGCCGGTGTATTGGGCGCTGCTCTGCTATGTGCCATTCATGGCGCTACCGTAGAAAATACTTTATTTGAAGATGGTGATGGTGCAAACACATTCCGTGCTTTTAACCCAACGCAAGCCGAAGAAACCTATTCGATGGTCACCGCTAACCGCTTTTGGTCTCAAATCTTTGGGGTTGCTTTTTCCAATAAACGTTGGTTACATTTCTTTATGTTATTTGTACCAGTAACCGGTTTATGGATGAGTGCTCTTGGAGTAGTCGGCCTAGCCCTGAACCTACGTGCTTATGACTTCGTTTCCCAGGAAATCCGTGCAGCGGAAGATCCTGAATTTGAGACTTTCTACACAAAAAATATTCTTTTAAACGAGGGTATTCGTGCTTGGATGGCGGCTCAAGATCAGCCTCATGAAAACCTTATATTCCCTGAGGAGGTTCTACCCCGTGGAAACGCTCTTTAA

>psbC

ATGAAAACCTTATATTCCCTGAGGAGGTTCTACCCCGTGGAAACGCTCTTTAATGGAACTTTAGCTGTAGCAGGTCGTGACCAAGAAACCACCGGTTTCGCTTGGTGGGCCGGGAATGCCCGACTTATCAATTTATCCGGTAAACTGCTGGGCGCTCATGTAGCCCATGCTGGATTAATCGTATTCTGGGCCGGAGCAATGAACCTATTTGAAGTGGCTCATTTCGTACCAGAAAAGCCCATGTATGAACAAGGATTAATTTTACTTCCCCACCTAGCTACTCTAGGCTGGGGGGTAGGTCCTGGTGGGGAAGTTATAGACACCTTTCCATACTTTGTATCTGGAGTACTTCACTTAATTTCCTCTGCAGTATTGGGCTTTGGCGGTATTTATCATGCACTTCTGGGACCTGAGACTCTTGAAGAATCTTTTCCATTCTTCGGTTATGTATGGAAAGATAGAAATAAAATGACCACAATTTTGGGTATTCACCTAATCTTGTTAGGTATAGGTGCTTTTCTTCTAGTATTCAAGGCTCTTTATTTTGGGGGCGTGTATGATACCTGGGCTCCGGGGGGGGGAGATGTAAGAAAAATTACCAACTTGACCCTTAGCCCAAGTGTTATTTTTGGTTATTTACTAAAATCCTTCTTTGGAGGAGAGGGGTGGATTGTTAGTGTGGATGATTTGGAAGATATAATTGGAGGTCATGTATGGTTAGGTTCCATTTGTATATTTGGTGGAATCTGGCATATCTTAACCAAGCCTTTTGCATGGGCTCGCCGTGCACTTGTATGGTCTGGGGAGGCTTACTTGTCTTATAGTTTAGGTGCTTTAGCTGTTTTTGGTTTCATTGCTTGTTGCTTTGTCTGGTTCAATAATACCGCTTATCCTAGTGAGTTTTATGGTCCCACTGGGCCAGAAGCTTCTCAAGCTCAAGCATTTACTTTTCTGGTTAGAGACCAACGTCTTGGGGCTAATGTGGGATCCGCTCAAGGGCCTACTGGTTTAGGTAAATATCTAATGCGTTCCCCTACTGGAGAAGTCATTTTTGGGGGAGAAACTATGCGTTTTTGGGATCTGCGTGCTCCATGGTTAGAACCTCTAAGGGGTCCCAACGGTTTAGACTTGAGTAGGTTGAAAAAGGACATACAACCTTGGCAAGAACGACGTTCTGCGGAATATATGACTCATGCTCCTTTAGGTTCTTTAAATTCTGTGGGTGGAGTAGCTACCGAGATCAATGCAGTTAATTATGTCTCTCCGAGAACTTGGTTAGCTACTTCTCATTTTGTTCTAGGCTTCTTCTTATTCGTAGGTCACTTATGGCACGCGGGAAGAGCTCGTGCAGCTGCAGCAGGGTTTGAAAAAGGAATTGATCGTGATTTTGAACCTGTTCTTTCGATGACTCCTCTTAACTGA

>psbZ

ATGACTATTGCTTTCCAATTGGCTGTTTTTGCATTAATTGCTACTTCATCAATCTTACTGATTAGTGTCCCCGTTGTATTTGCTTCTCCGGATGGTTGGTCGAGTAACAAAAATGTCGTATTTTCTGGTACATCATTGTGGATTGGATTAGTCTTTCTGGTGGGTATCCTTAACTCTCTCATTTCTTAA

>rps14

ATGGCAAGGCAAAGTTTGATTCAGAGGGAAAAGAAAAGGCACAAATTGGAACAAAAATATCATTTGATTCGTCGATCCTCAAAAAAAGAAATAAACAAAGCTCCATCGTTGAGCGATAAATGGAAAATTCATGGAAAGTTACAATCCTCACCGCGTAATAGTGCACCTACCCGTCTTCATCGACGTTGTTTTTTGACCGGAAGGCCGAGAGCTAACTATCGAGACTTTGGACTATCCGGACACATACTTCGTGAAATGGTTCATGCGTGTTTGTTGCCGGGGGCAACAAGATCAAGTTGGTAA

>psaB

ATGGCATTAAGATTTCCAAGGTTTAGCCAAGGCTTAGCTCAGGACCCCACTACTCGTCGTATTTGGTTTGGTATTGCTACTGCACATGACTTCGAGAGTCATGATGATATTACTGAGGAACGTCTTTATCAGAATATTTTTGCTTCTCACTTCGGGCAATTAGCAATAATTTTTCTGTGGACTTCCGGAAATCTCTTTCATGTAGCTTGGCAAGGAAATTTTGAGGCATGGGTACAGGACCCTTTACATGTAAGACCTATTGCTCATGCAATTTGGGATCCTCATTTTGGTCAACCGGCCGTGGAAGCTTTTTCTCGGGGAGGTGCTCTTGGCCCGGTGAATATCGCTTATTCTGGTGTTTATCAGTGGTGGTATACAATCGGTTTACGCACTAATGAGGATCTTTATACTGGAGCTCTTTTTCTATTATTTCTTTCCGCCATATCTTTAATAGCGGGTTGGTTACACCTACAACCGAAGTGGAAACCGAGCGTTTCGTGGTTCAAAAATGCCGAATCTCGTCTGAATCATCATTTGTCAGGACTGTTCGGAGTAAGTTCCTTGGCTTGGACAGGGCATTTAGTACATGTCGCTATTCCTGGATCCAGGGGGGAGTATGTTCGATGGAATAATTTCTTAGATGTATTGCCGCATCCCCAAGGGTTAGGCCCACTTTTTACGGGTCAGTGGAATCTTTATGCGCAAAACCCCGATTCAAGTAGTCATTTATTTGGTACCTCCCAAGGATCAGGAACTGCCATTCTAACCCTTCTCGGGGGATTCCATCCACAAACGCAAAGCTTATGGCTGACCGATATTGCTCATCATCATTTAGCTATTGCATTTATTTTTCTCGTTGCTGGTCATATGTATAGAACGAATTTCGGGATTGGGCACAGTATAAAAGATCTTTTAGAAGCACATATTCCTCCGGGAGGACGCTTGGGGCGCGGGCATAAGGGTCTTTATGACACAATCAATAATTCGCTTCATTTTCAATTAGGCCTTGCTCTAGCCTCTTTAGGGGTTATTACTTCCTTGGTAGCTCAACACATGTACTCTTTACCTGCTTATGCATTCATAGCGCAAGATTTTACTACTCAAGCTGCGTTATATACTCATCACCAATACATCGCGGGATTCATCATGACAGGAGCTTTTGCTCACGGAGCTATCTTTTTTATTAGAGATTACAATCCGGAACAGAATGAGGATAATGTATTGGCAAGAATGTTAGACCATAAGGAAGCTATTATATCCCATTTAAGTTGGGCCAGCTTGTTTCTGGGGTTCCATACTTTGGGACTTTATGTTCATAATGATGTCATGCTTGCTTTTGGTACTCCGGAGAAACAAATCTTGATCGAACCGATATTTGCCCAATGGATACAATCTGCTCATGGTAAAACTTCATATGGATTCGATGTACTTTTATCTTCAACGGATGGCCCTGCATTCAATGCGGGTCGAAGCATATGGTTGCCTGGCTGGTTAAGTGCTGTTAATGAGAATAGTAATTCTCTATTCTTAACAATAGGCCCTGGAGACTTTTTGGTTCATCATGCTATTGCTCTAGGTTTACATACAACTACATTGATCTTGGTAAAGGGGGCTTTAGATGCACGGGGTTCTAAGTTAATGCCAGATAAAAAGGATTTCGGTTATAGTTTTCCTTGCGACGGTCCGGGACGAGGCGGTACTTGTGATATTTCGGCTTGGGACGCATTTTATTTGGCAGTTTTCTGGATGTTAAATACCATTGGGTGGGTTACTTTTTATTGGCATTGGAAACACATCACGTTATGGCAGGGTAACGTTTCACAGTTTAATGAATCTTCCACTTATTTGATGGGATGGTTAAGAGATTATCTATGGTTAAACTCTTCCCAACTTATCAATGGGTATAACCCGTTTGGTATGAATAGTTTATCAGTCTGGGCGTGGATGTTCTTATTTGGACATCTTGTTTGGGCTACTGGATTTATGTTTTTAATTTCCTGGCGCGGGTATTGGCAAGAATTGATTGAAACTTTAGCGTGGGCTCACGAACGCACACCCTTAGCTAATTTGATTAGATGGAGAGATAAACCAGTGGCCCTTTCCATTGTGCAAGCACGATTGGTTGGATTAGCCCACTTCTCTGTAGGTTATATATTCACTTATGCGGCTTTCTTGATTGCCTCTACATCGGGCAAATTTGGTTAA

>psaA

ATGATTATTCGTTCGCCGGAACCAGAAGTAAAAATTTTGGTAGATAGGGATCCCGTAAAAACTTCTTTCGAGGAATGGGCCAAACCGGGGCATTTCTCAAGAACCATAGCTAAGGGACCTGAGACTACCACTTGGATCTGGAACCTACATGCTGATGCTCACGACTTCGATAGCCATACCAGTGATTTGGAGGAGATCTCTCGAAAAGTATTTAGTGCCCATTTCGGACAACTCTCCATCATTTTTCTTTGGCTGAGCGGAATGTATTTCCACGGTGCTCGTTTTTCCAATTATGAAGCCTGGCTAAGCGATCCTACTCACATTGGACCTAGCGCACAGGTGGTTTGGCCAATAGTGGGCCAAGAAATATTGAACGGTGATGTGGGCGGGGGTTTCCGAGGAATACAAATAACCTCCGGGTTTTTTCAGCTTTGGCGAGCATCTGGAATAACTAGTGAATTACAACTCTATTGTACCGCAATTGGCGCATTGATTTTTGCAGCCTTAATGCTTTTTGCTGGTTGGTTCCATTATCACAAAGCTGCTCCAAAATTGGCTTGGTTTCAGGATGTAGAATCTATGTTGAACCACCATTTAGCGGGGCTGCTAGGGCTCGGGTCCCTTTCTTGGGCCGGGCATCAAGTACATGTATCTTTACCGATTAACCAATTTCTAAACGCTGGAGTAGATCCTAAAGAGATCCCACTTCCTCATGAATTTATCTTGAATCGGGATCTTTTGGCTCAACTTTATCCCAGTTTTGCCGAAGGAGCAACCCCATTTTTTACCTTGAATTGGTCAAAATATGCGGAATTTCTTACTTTTCGTGGCGGATTAGATCCAGTAACTGGGGGTCTATGGCTGACCGATATTGCACACCATCATTTAGCTATTGCAATTCTTTTCCTGATCGCGGGGCACATGTATAGGACCAACTGGGGGATTGGTCATGGTCTAAAAGATATTTTAGAGGCTCATAAAGGTCCATTTACAGGTCAAGGCCATAAAGGATTATATGAGATCCTAACAACATCATGGCATGCTCAATTATCGCTTAACCTAGCTATGTTAGGATCTTTAACCATTATTGTAGCTCATCATATGTATTCCATGCCCCCTTATCCATATCTAGCTACTGACTATGGTACACAACTGTCATTGTTCACACACCACATGTGGATTGGTGGATTTCTCATTGTTGGCGCTGCTGCGCATGCAGCCATTTTTATGGTAAGAGACTATGATCCAACTACTCGATACAACGATCTCTTAGATCGTGTCCTTCGGCATCGCGATGCAATCATATCACATCTCAACTGGGTATGTATATTTCTAGGATTTCACAGTTTTGGTTTGTATATTCATAATGATACCATGAGTGCTTTAGGGCGTCCACAAGATATGTTTTCAGATACCGCGATACAATTACAACCTGTCTTTGCTCAATGGATACAAAACACCCACGCCTTAGCACCCGGCGGAACGGCCCCTGGTGCAACAGCAAGCACCAGTTTGACTTGGGGGGGTGTTGATTTAGTGGCAGTGGGCGGAAAAGTTGCTTTGTTACCTATTCCATTAGGAACCGCGGATTTTTTGGTACATCACATTCATGCATTTACGATTCATGTGACGGTATTGATACTCCTGAAAGGAGTTCTCTTTGCTCGTAGCTCGCGTTTGATACCGGATAAAGCAAATCTTGGGTTTCGTTTCCCTTGTGATGGTCCTGGAAGAGGGGGGACATGTCAAGTATCCGCTTGGGATCATGTCTTCTTAGGACTATTCTGGATGTACAATGCAATTTCGGTAGTAATATTCCATTTCAGTTGGAAAATGCAGTCAGATGTTTGGGGTAGTATAAGTGATCAGGGGGTGGTAACTCATATTACAGGAGGAAACTTTGCGCAGAGTTCCATTACGATTAATGGGTGGCTCCGCGATTTTTTATGGGCACAGGCATCCCAGGTAATTCAGTCTTATGGTTCCTCATTATCTGCATATGGCCTTTTTTTCCTAGGTGCTCATTTTGTATGGGCTTTTAGTTTAATGTTTCTATTCAGCGGGCGTGGTTATTGGCAAGAACTTATTGAATCCATCGTTTGGGCTCATAATAAATTAAAAGTTGCTCCTGCTACTCAGCCTAGAGCCTTGAGCATTGTACAAGGACGCGCTGTAGGAGTAACCCATTACCTTCTAGGTGGAATTGCCACAACATGGGCGTTCTTCTTAGCAAGAATTATTGCAGTAGGATAA

>ycf3

ATGCCTAGATCGCGGATAAACGGAAATTTTATTGATAAGACCTTTTCGATTGTAGCCAATATATTATTACGAATAATTCCGACAACTGTAGGAGAAAAAGAGGCATTTACCTATTACAGAGATGGTATGTCAGCTCAATCTGAAGGCAATTATGCGGAAGCTTTACAGAATTATTATGAAGCTATGCGACTAGAAATTGATCCTTACGATCGAAGCTATATACTCTATAACATAGGCCTTATCCACACAAGTAACGGAGAACATACAAAAGCTTTAGAATATTATTTTCGGGCACTAGAACGAAATCCGTTCTTACCACAAGCTTTTAATAATATGGCTGTGATCTGTCATTACCGGGGGGAACAGGCCGTTCGACAGGGAGATTCTGAAATTGCGGAGGCTTGGTTCAATCAAGCTGCCGAGTATTGGAAACAAGCTATTGCGCTTACTCCTGGTAATTATATTCAAGCGCAGAATTGGTTGAAGATCACGGGACGTTTCGAATAA

>rps4

ATGTCACGTTACCGAGGGCCTCGTTTCAAAAAAATACGCCGTCTGGGGGCTTTGCCGGGACTAACGAGTAAAAGGCCTAGAGCCGGAAGCGATTTTAGAAACCAATCGCGCTCCGTAAAAAAATCTCAATATCGAATTCGTTTAGAAGAAAAACAAAAATTGCGTTTTCATTATGGTCTTACAGAACGACAATTACTTAAATATGTTCGTATCGCCAGAAAAGCCAAAGGGTCAACCGGTCTGGTTTTACTACAATTACTTGAAATGCGTTTGGATAACATTCTTTTTCGATTGGGTATGGCTTCAACTATTCCTCAAGCCCGCCAATTGGTTAACCATCGACATATTTTAGTTAATGGTCGTATAGTCGATATACCAAGTTATCGTTGCAAACCCCGAGATATTATTACAGTAAGGGATGACCCAAAATCTAGATCTATGGTTCAAAATTATCTTGATTCATCCCACCATGAGGAATTGCCAAAGCATTTGACTCTTCGCGCATTCCAATATAAAGGATTAGTCAATCAAATAATAGATAGTCAATGGGTCGGTTTGAAAATAAATGAATTGCTTGTCGTAGAATATTATTCTCGTCAAACTTAA

>ndhJ

ATGCAGGGTCGTTTGTCTGCTTGGCTGGTCAAGCATGGGCTAGTTCATAGATCTTTGGGCTTTGATTACCAAGGAATAGAAACTTTACAAATAAAGCCCGAGGATTGGCACTCCGTTGCTGTCATTTTGTATATATATGGCTACAATTATCTACGGTCCCAATGTGCCTATGATGTAGCACCGGGCGGACTGTTAGCCAGTGTGTATCATCTTACGCGAATAGAGTATGGTGTAGATCAACCAGAAGAGGTATGCATAAAAGTATTTGCTCCAAGGAGTAATCCTAAAATTCCATCGGTTTTCTGGGTTTGGAAAAGTGCGAATTTTCAAGAACGGGAATCTTATGATATGCTGGGAATCCTTTATGATAATCATCCACGACTGAAACGTATCTTAATGCCTGAAAGTTGGATAGGGTGGCCCTTACGTAAGGATTATATTGCCCCCAATTTTTATGAAATACAAGATGCTTATTGA

>ndhK

ATGAATTCTATTGAATTTTCCTTACTTGATCGAACAACCCAAAATTCATTTATTTCAACTACATCAAACGATCTTTCAAATTGGTCAAGACTCTCCAGCTTATGGCCGCTTCTTTATGGTACCAGTTGTTGTTTCATTGAATTTGCTTCATTAATCGGCTCGCGGTTCGATTTTGACCGTTATGGGCTGGTACCAAGATCGAGCCCTAGACAGGCGGACCTAATTTTAACAGCTGGTACAGTAACAATGAAAATGGCTCCTTCTTTAGTGAGATTATATGAACAAATGCCTGAACCAAAATATGTTATTGCTATGGGAGCATGTACAATTACAGGGGGGATGTTCAGTACCGATTCGTATAGTACTGTTCGAGGAGTTGATAAGCTAATTCCCGTGGATGTTTATTTGCCGGGTTGCCCGCCTAAACCAGAGGCCGTTATAGATGCCATAACAAAACTTCGTAAGAAAATATCTCGAGAAATCTATGAAGATCGAATTCGATTGCAACGGGAGAATCGCTCGTTTACTTTTACTACCAATCACAAGTTTCGTGTTGTATGCAGTACTAATACTGGAAATTATGATCAAGGATTACTTTATCAACCACCATCTACGTCAGAAATCCCGCCTGAAACCTTTTTCAAATACAAAAGTTCAGTATCTTCCCCCGAATTCATTAATTAG

>ndhC

ATGTTTCTGCTTTACAAATATGATATTTTCTGGGCATTTCTAATAATATCAAGCGTTATTCCTATTTTGGCATTTCTAATTTCCGCAGTTTTAGCCCCGATTAACAAAGGGCCAGAGAAACTTTCTAGTTATGAATCGGGTATCGAACCAATGGGCGATGCTTGGTTACAATTTCGAATCCGGTATTATATGTTTGCTCTAGTTTTTGTTGTTTTTGATGTTGAAACCGTTTTTCTTTATCCATGGGCAATGAGTTTTGATGTATTGGGGGTACCCGTATTTATAGAAGCTTTCATTTTCATGCTTATCCTAATTGTTGGTTTAGTTTATGCGTGGCGAAAAGGAGCATTAGAGTGGTCTTAG

>atpE

ATGACCTTAAATCTTTGTGTACTGACCCCTAATCGAATTGTTTGGGATTCAGAAGTGAAAGAAATCATTTTATCTACTAATAGTGGACAAATCGGCGTATTACCAAATCACGCGCCTATTGCCACAGCTGTAGATATAGGTATTTTAAGAATCCGCTTTAACGACCAATGGTTAACGATGGCTCTGATGGGTGGTTTTGCTAGAATAGGGAATAATGAGATCACTATTTTAGTAAATGATGCGGAGAAGAGTAGTGACATTGATCCCCAAGAAGCCCAGCAAACTCTTGAAATAGCAGAAGCTAATTTGAGGAAAGCTGAAAGCAAGAGACAAACAATTGAGGCAAATCTAGCTCTCAGACGAGCTAGGACACGAGTAGAGGTTATCAATGCGATTTCATAA

>atpB

ATGAGAATAAATCCTACTACTTCCGGTCCTGGGGTTTCCGCGTTTGCAAACAAAAACCTAGGACATATCGCTCAAATCATTGGTCCGGTACTGGATGTAGCCTTTCCCCCCGGCAAGATGCCTAATATTTACAATGCTCTGGTAGTTAAGGGTCGAGATACTGTCGATCAACCGATTAATGTGACTTGCGAGGTACAGCAATTATTAGGGAATAATCGAGTTAGAGCTGTAGCCATGAGTGCTACAGATGGTCTAACGAGGGGTATGGAAGTAATTGACACGGGAGCTCCTCTAAGTGTTCCAGTCGGCGGAGTGACTCTAGGACGAATTTTCAACGTGCTTGGAGAGCCCGTTGATAATTTAGGTCCTGTAGATACTCGCACAACATCCCCTATTCATAAATCCGCGCCTGCTTTTATACAGTTAGATACAAGATTATCTATTTTTGAAACAGGAATTAAAGTAGTAGATCTTTTAGCTCCTTATCGTCGGGGAGGAAAAATCGGACTATTTGGGGGAGCTGGGGTGGGTAAAACAGTACTCATTATGGAATTGATCAACAACATTGCCAAAGCCCATGGGGGTGTATCCGTATTTGGCGGAGTCGGTGAACGTACTCGTGAAGGAAATGATCTTTACATGGAAATGAAAGAGTCTGGAGTAATTAATGACCAAAATCTTTCGGAATCAAAAGTGGCTCTAGTCTACGGTCAGATGAATGAACCACCAGGAGCTCGTATGAGAGTTGGTTTGACGGCCCTAACTATGGCGGAATATTTCCGAGATGTTAATGAACAAGACGTACTTCTATTTATCGACAATATCTTCCGTTTCGTCCAAGCGGGATCCGAGGTATCCGCCTTATTGGGTAGAATGCCTTCCGCTGTGGGTTATCAACCTACCCTTAGTACCGAAATGGGTTCTTTACAAGAAAGAATTACTTCTACCAAAGAGGGGTCCATAACTTCTATTCAAGCAGTTTATGTACCCGCGGATGATTTGACTGATCCCGCTCCTGCCACGACATTTGCCCATTTAGATGCTACTACCGTACTATCAAGAGGATTAGCTGCTAAAGGTATCTATCCAGCAGTTGATCCTTTAGACTCAACGTCAACTATGCTGCAACCTCGGATCGTTGGCGAGGAACATTATGAAACGGCGCAAAGAGTTAAGCAAACTTTACAACGTTACAAAGAACTTCAGGACATTATAGCTATCCTTGGGTTGGACGAACTGTCCGAAGAGGATCGTTTAACTGTAGCAAGGGCGCGAAAAATTGAGCGTTTCTTATCACAACCCTTTTTCGTAGCCGAAGTATTTACCGGTTCCCCGGGGAAATATGTTGGTCTAGCCGAAACTATTAGAGGGTTTAAATTGATCCTGTCCGGAGAATTAGATGGTCTTCCTGAGCAGGCCTTTTATTTGGTAGGTAACATAGATGAAGTTACTGCGAAGGCTACAAACTTAGAAATGGAGAGTAATTTGAAGAAATGA

>rbcL

ATGTCACCACAAACAGAGACTAAAGCGAGTGTTGGATTCAAGGCCGGTGTTAAAGATTATAAATTGACTTATTATACTCCTGACTATGTAACCAAAGATACTGATATCTTGGCAGCATTCCGAGTAAGTCCTCAGCCCGGAGTTCCACCCGAGGAAGCGGGGGCTGCGGTAGCTGCGGAATCTTCTACTGGTACCTGGACAACTGTGTGGACCGATGGGCTTACCAGCCTTGATCGTTACAAAGGGCGATGCTACAACATTGAGCCCGTTGCTGGAGAAGAGAATCAATATATATGTTATGTAGCTTACCCGTTAGACCTTTTTGAAGAAGGTTCTGTTACTAACATGTTTACTTCCATTGTGGGTAATGTATTTGGTTTCAAAGCACTGCGCGCTCTACGTCTAGAGGATCTACGAATCCCTCCTGCGTATACTAAAACTTTCCAAGGCCCGCCTCACGGCATCCAAGTTGAGAGAGATAAATTGAACAAGTATGGCCGTCCCCTGTTGGGATGTACTATTAAACCTAAACTGGGGTTATCCGCGAAGAATTATGGTAGGGCGGTTTATGAATGTCTACGCGGTGGACTTGACTTTACCAAAGATGATGAGAACGTGAACTCCCAACCATTTATGCGTTGGAGGGACCGTTTCTTATTTTGTGCGGAAGCTCTTTATAAAGCGCAAGCTGAAACAGGTGAAATCAAAGGTCATTACTTGAATGCTACTGCAGGGACATGCGAAGAAATGCTAAAAAGGGCTGTCTTTGCCAGAGAGTTGGGAGTTCCTATCGTAATGCATGACTACTTAACAGGGGGATTCACCGCAAATACTACCTTGGCTCATTATTGCCGAGATAATGGTCTACTTCTTCACATCCACCGTGCAATGCATGCAGTTATTGATAGACAGAAGAATCATGGTATGCACTTTCGTGTACTAGCTAAAGCTTTGCGTCTGTCTGGTGGAGATCATATTCACGCCGGTACAGTAGTAGGTAAACTTGAGGGGGAAAGAGACATAACCTTGGGATTTGTTGATTTACTACGTGATGATTTTGTTGAAAAAGATCGAAGCCGCGGTATTTATTTCACTCAAGATTGGGTCTCTATACCAGGTGTTATACCTGTGGCTTCCGGGGGTATTCACGTTTGGCATATGCCTGCGTTGACAGAGATCTTTGGAGATGATTCCGTACTACAATTTGGTGGAGGAACTTTAGGACACCCTTGGGGAAATGCACCCGGCGCTGTAGCTAATCGAGTAGCTCTAGAAGCATGTGTACAAGCTCGTAATGAAGGACGCGATCTTGCTCGTGAAGGTAATGAAATTATCCGGGAGGCTAGCAAATGGAGTCCTGAACTGGCTGCTGCTTGTGAAATCTGGAAGTCGATCAAATTCGAATTTGCCGCAATGGATACTTTGTAA

>accD

ATGAAAAAATGGTGGTTCAATTCGATGTTATCTAAGGGTAAGGGGGAATTAGAATACAGGTCTTGGTTAAGTAAATCAATGGAGAGCCCTGGTCCTATTAAAAATCCCAGTGTAAGCGAGGAACTGATTAGAAATGATAAGAATAAAAATATTCATAGTTCGAGCGATAGTGACAGTTCAAGTTACAGCAAATTAGCTGGTGTCAGGGACATTCATAATTTCATCTCGGATGACACTTTTTTTGTTAAGGATAGTAATAGAGACAGTTATTCCATCTATTTTGATATTGAAAATCAAATTTTGGAACTAGACAATGCTCATTCTTTTCTGAGTGAACTAGAAAGTTCTTTTTATAGCTTTCGTAATTATAGTTCGAGGAATAATGGATCTAAAAGCGCTGATCCTGATTCCGATCGTTACATGTATGATACTAAATCGAGTTGGAATAATCACATTCATAATTGCCTTGACTCTTATCTTCATTCTCAAATCTGTATTGATAGTCACCTTTTAAGTATTTTAAGTAGTAGTGACAATTATAGTGCCAGTTACATTTATAATTTCATTTGTAGTGAAAGTGAGAGTTCCAATATACAAAGTAGCACGAATGGTAGTGATTTAACTATAAGCGAAAGTTCTAATGAAAGCGAAAGTTCTAATGAAAGCGATGTAACTCAAAAATACAGGCATTTATGGGTTCAATGCGAAAATTGTTATGGATTAAATTATAAGAAATTTCTTAAATCAAAAATGTATCTTTGTGAACAATGCGGATATCATTTGAAAATGATTAGCTCAGATAGAATCGACCTTTTGGTTGATCCAGGTACTTGGGATCCGATGGATGACGACATGGTCTCTATAGATCCCATTGAATTTGATTCAGAAGAGGAACCTTATAAAAATCGTATTGATTCTTATCAAAGCAAGACAGGATTAACGGAGGCTGTTCAAACAGGTACAGGGCAACTAAACGGGATTCCCATCGCAATTGGGGTTATGGATTTTCAGTTTATGGGGGGTAGTATGGGATCCGTAGTAGGCGAGAAAATAACCCGTTTGATCGAGTATGCTGCCAATAAATTTTTACCTCTTCTTCTAGTGTGTGCTTCTGGGGGAGCACGCATGCAAGAAGGAAGTTTGAGCTTGATGCAAATGGCTAAAATATCTTCTGCTTTATATGATTATCAATCAAATAAAAAGTTATTCTATGTATCAATTCTTACATCCCCTACTACGGGTGGAGTGACAGCTAGTTTTGGTATGTTGGGGGATATCATTATTGCTGAACCTAATGCCTATATTGCATTTGCAGGTAAAAGAGTAATTGAACAAACATTGAATAAGACAGTACCTGAAGGTTCACAAGAGGCTGAATATTTATTCGATAAGGGCTTATTCGATCCAATCGTACCACGTAATCCTTTAAAAGGTGTTCTGAGCGAGTTATTTCTGTTCCACGGCCGTTTTCCTTTGAATCAAAATTAA

>psaI

ATGACAATTCTCAACAGCTTTCCCTCTATTTTTGTGCCTTTAGTGGGCCTAGTATTTCCGGCAATGGCAATGGCTTCGTTATTTCTTTATCTTGAAAAAAATAAGATTTTGTAA

>ycf4

ATGAGTTGGCGATCAGAATATATATGGGTAGAATTTATAGCGGGCTCTCGCAAACCAGGCAATTTCTTCTGGGCCTTTATCCTTTTTTTAGGCTCATTAGGATTCTTAGTGGTTGGAATTTCTAGTTATCTTGATAGGAATTTGCTATCTTTATTTCCGTCGCAGCAAATAATTTTTTTTCCACAAGGGATCGTGATGTCTTTCTACGGGATCGCGGGTCTCTTTATTAGTTCCTATTTGTGGTGCACAATTATATGGAATGTAGGTAGCGGTTATGATCGATTTGATACAAAAGAGGGAATAGTGTGTATTTTTCGTTGGGGATTTCCTGGAAAAAATCGCCGCATCTTTCTACGATTCCTTATGAAAGATATTCAGTCCATCAGAATAGAAGTTAAAGAGGGTATTTATGCTCGTCGTGTCCTTTATATAGAAAGCAGAGGCTTGGGGGCCATTCCCTTGAATCGTACTGATGAGAATTTGACTCCGCGAGAAATTGAGCAAAAGGCTGCGGAATTGGCCTATTTCTTGCGTGTACCAATTGAAGGATTTTGA

>cemA

ATGACAAAAAAGAACGCATCCATTCCCCTTAGATATCTTTCATCTATAGTATTTGTAGTATTTTTGCCCTGGTGGATCCCTCTCTCATTTAATAAAAGTCTGGAATCCTGGGTTACTAATTGGTGGAATACTAGTCAATCCGAAACCTTTTTGAATCATATTCAAGAAAAGGCTATTCTAGAAAAATTCATAGAATTAGAGGAATTATTCCTCTTGGACGAAATGATAAAGGAATTTCCGGAAAGACGTCTAGAAAAGCTTCGTATAGGGCTCCAGAAAGAAACAATCCAATTAATCAAGATGCACGATGAGGATCATATCCATACGATTTTTCACTTCTCGACAAATACAATCTGCTTCGTTATTCTAAGTGGTTATTCTATTTTGTGTAATGAAGAACTTTTTATTCTTAACTCTTGGGTTCAAGAATTCCTATATAATTTAAGCGACACAATAAAAGCCTTTTCGATTCTTTTCGTAACTGATTTATGTATCGGATTCCATTCACCCCGCGGTTGGGAACTCCTGATTGGCTATGTCTACAACGACTTTGGGCTTGCTCATAATGATAATGATATTATTCTATCTGTTCTTGTTTCCACTTTTCCAGTCGTTCTAGATACATTTTTTAAATATTGGCTTTTTTCTTATTTAAATCGTGTATCTCCGTCACTTGTAGTGATTTATCATTCAATGACTGAGTGA

>petA

ATGCAAATTAGAAATACCTTTTCTTCGTTAAAGGGAGAGATTACTCGATTCATTTCCGTATCCCTCATGATATATATAATAACTCGGGCATCAATTTCAAATGCATATCCCATTTTTGCGCAGCAGGGTTTTGAAAATCCACGAGAAGCAACTGGTCGTATTGTATGCGCCAATTGCCATTTAGCTAATAAGCCCGTGGATATTGAGGTTCCACAGGCGGTACTCCCCGATACTGTATTTGAAGCAGTTGTTAGAATTCCTTATGATATGCAACTAAAACAAGTTCTTGCTAATGGTAAAAAGGGGGCTTTGAATGTGGGGGCCGTTCTTATTTTACCAGAGGGGTTTGAATTAGCCCCCCCCGATCGTATTTCGCCCGAGATGAAAGAAAAGATAGGCAAGCTGTCTTTTCAGACCTACCGACCCACTAAAAAAAACATTCTTGTGATAGGGCCAGTTCCTGGTCAGAAATATAGTGAAATAACTTTTCCTATTCTTTCCCCGAACCCCGCGACTAATAAAGATGCTTACTTCTTAAAATATCCAATATACGTAGGTGGGAACAGGGGAAGGGGTCAGATTTATCCCGACGGGAACAAAAGTAACAATACGGTTTATAATGCTACAGCTGCGGGTATAGTAAGCAAAATCATACGAAAAGAAAAAGGGGGATACGAAATAACCATAACGGATGCATCGAATGGACGTGAAGTGGTTGATATTATCCCCCCAGGACCAGAACTTCGTGTTTCAGAGGGCCAATCTATCAAACTTGATCAACCATTAACAAGTAATCCTAATGTAGGCGGGTTTGGTCAGGCAGATGCAGAAATAGTACTTCAAGATCCATTACGTGTCCAAGGCCTTTTGTTCTTTTTGGCATCTGTTGTTTTGGCACAAATATTTTTGGTTCTTAAAAAGAAACAGTTTGAGAAGGTTCAATTGTCCGAAATGAATTTCTAG

>psbJ

ATGGCCGATACTACTGGAAGGATTCCCCTTTGGATAATAGGAACTGTAACTGGTATTCCTGTGATCGGTTTAATAGGCATTTTCTTTTATGGTTCATATTCCGGATTAGGTTCGTCCTTGTAG

>psbL

ATGACACAATCAAACCCAAACGAACAAAATGTTGAATTGAACCGTACCAGTCTCTACTGGGGGTTATTGCTCATTTTTGTACTTGCTGTTTTATTTTCCAATTACTTCTTCAATTAA

>psbF

ATGACCATAGATCGAACCTATCCAATTTTTACAGTGCGATGGTTGGCTGTTCACGGACTAGCTGTACCTACCGTTTCTTTTTTGGGGTCAATATCAGCAATGCAGTTCATCCAACGCTAA

>psbE

ATGTCTGGAAGCACAGGAGAACGTTCTTTTGCTGATATTATTACCAGTATTCGATACTGGGTCATTCATAGCATTACTATACCTTCCCTATTCATTGCGGGTTGGTTATTCGTCAGCACGGGGTTAGCTTATGATGTGTTTGGAAGCCCCCGGCCAAACGAGTATTTTACAGAGAGCCGGCAAGGAATTCCATTAATAACCGGCCGTTTTGATTCTTTGGAACAACTCAATGAATTTAGTAGATCTTTTTAG

>petG

ATGATTGAAGTCTTTCTATTTGGAATCGTCTTAGGTCTAATTCCTATTACTTTGGCTGGATTATTCGTAACCGCCTATTTACAATACAGACGTGGTGATCAGTTGGACCTTTGA

>psaJ

ATGCGAGATCTAAAAACATATCTTTCCGTGGCACCGGTACTAAGTACTCTATGGTTCGGGTCTTTAGCAGGGTTATTGATAGAAATCAACCGTTTATTCCCCGACGCATTGACATTTCCTTTTTTTTCATTCTAG

>rpl33

ATGGCCAAGGGTAAAGAGGTCCGAGTAAGGGTTATTTTGGAATGTACTAGTTGTGTTCGAAACGGTGTTAATAAGGAATCAAGGGGTATTTCCAGATATATTACTCAAAAGAATCGACACAATACACCCAGTCGATTGGAATTGAGAAAATTCTGTCCCTATTGTTACAAGCATACACTTCATGGGGAGATAAAAAAATAG

>rps18

ATGGATAAAACCAAGCGACTCTTTCTTAAATCCAAGCGATCTTTTCGTAGGCGTTTGCCCCCGATCCAATCGGGGGATCGAATTGATTATAGAAACATGACTTTAATTAGTCGATTTCTTAGTGAACAAGGAAAAATATTATCTAGACGGGTGAATAGATTGACCTTAAAAGAACAACGATTAATTACTATTGCTATAAAACAAGCTCGTATTTTATCTTCGTTACCTTTTCTTAATAATGAGAAACAATTTGAAAGAAGTGGGTTGACCGCTAGACCTCCCGGTCTTAGAACCAGAAAAAAATAG

>rpl20

ATGACCAGAATTCGACGAGGATATATAGCTCGGAGACGTAGAACAAAAATGCGTTTATTTGTATCAAGCTTTCGCGGGGCTCATTCACGACTTAGCCGAACAATTACTCAACAGAAAATAAGAGCTTTGGTTTCGGCTCATCGCGATAGAGATAGGAAAAAAAGGGATTTTCGTCGTTTGTGGATCACCCGAATAAATGCAGTAATTCGCGGAAATCAGGTATCCTATAGTTATAGTAGATTAATATACAATCTGTATAAGGCGCAGTTGGTTCTTAATCGTAAGATACTTGCACAAATAGCTATATCAAATAGGAATTGTCTTTATATGATTTCCAATGAGATTATAAAATAA

>rps12

ATGCCAACTATTAAACAACTTATTAGAAACCCAAGACAGCCAATCAGAAATTTTACCAAATCCCCCGCTCTGCGGGGATGTCCTCAGCGCCGAGGAACATGTACAAGGGTGTATACTATCACCCCCAAAAAACCAAACTCTGCCTTACGTAAAGTTGCCAGAGTACGATTAACCTCTGGGTTTGAAATCACTGCTTATATACCTGGTATTGGCCATAATTCACAAGAACATTCTGTAGTCTTAGTAAGAGGGGGGAGGGTTAAGGATTTACCCGGTGTGAGATATCACATTGTTCGAGGAACCCTAGATGCTGTCGGAGTAAAGGATCGTCAACAAGGGCGTTCTAAATATGGGGTCAAAAAGCCAAAATAA

>clpP

ATGCCTATTGGTGTTCCAAAAGTACCTTATCGAAGTCCCGGGGACAAGCATCCATCGTGGGTTGACATATACAACCGACTTTATCGAGAAAGATTACTTTTTTTAGGTCAAATGGTTGAGAGCGATATCTCGAATCAACTTATTGGTATTATGGTATATCTCAGTATAGAGAACGAGACCAAGGATTTGTATTTATTTATCAACTCTCCTGGCGGATGGGTAATACCCGGGATAGCAATTTATGATACTATGCAATTTGTGCGACCCGATGTACAGACAATATGCATGGGATTGGCCGCCTCCATGGGGTCTTTTCTCCTGGCCGCCGGAGCAAGTACCAAACGTCTAGCATTCCCTCACGCTAGGGTCATGATCCATCAACCTATTGGCGCTTTTTATGGGGCACAAACGGGAGAATTTATCCTGGATACGGAAGAACTACTGAGACTGCGCGAAATCCTTACAATGGTTTATGTACAAAGATCGGGCAAGCCCTTATGGGTTGTATCCGAAGACATGGAAAGGGATACTTTTATGTCAGCAACAGAAGCCCAAGCTCATGGACTTGTTGATCTTGTAGCGGTTGGATAA

>psbB

ATGGGTTTGCCTTGGTATCGTGTTCATACCGTCGTATTGAATGATCCCGGTCGTTTGATTGCTGTCCATATAATGCATACAGCCCTGGTTGCGGGTTGGGCCGGTTCAATGGCTCTATATGAATTGGCTGTTTTTGATCCCTCCGATCCAGTTCTTGATCCAATGTGGAGACAAGGCATGTTCGTTATACCCTTCATGACTCGTTTAGGAATAACCGATTCATGGGGCGGTTGGAGTATTACAGGGGGGACAGTAACGAATCCGGGTATTTGGAGTTACGAAGGTGTAGCGGGGGCACATATTGTGTTTTCCGGATTGTGCTTCTTGGCAGCTATCTGGCATTGGGTGTATTGGGATCTAGCAATATTTGTTGATGACCGTACAGGAAAACGTTCTTTGGATTTGCCTAAAATCTTTGGAATTCATTTATTTCTCTCAGGAGTGGCTTGCTTTGGTTTTGGGACATTTCATGTAACAGGATTGTATGGTCCTGGAATATGGGTGTCTGATCCGTATGGACTAACTGGAAAGGTACAATCTGTAAATCCAGCATGGGGTGTGGAAGGTTTTGATCCTTTTGTTCCAGGAGGAATAGCCTCTCATCATATTGCGGCAGGGACATTGGGCATATTAGCAGGCTTATTCCATCTCAGTGTCCGCCCGCCACAACGCTTATACAAAGGATTACGTATGGGCAATATTGAAACCGTTCTTTCCAGCAGCATCGCTGCTGTCTTTTTTGCAGCGTTTGTTGTTGCTGGAACTATGTGGTATGGGTCAGCAACTACCCCCATCGAATTATTTGGTCCCACCCGTTATCAATGGGATCAGGGATACTTTCAGCAAGAAATATATCGAAGAGTCAGTGCTGGACTAGCCGAAAATCAAAGTTTATCAGAAGCTTGGTCTAAAATTCCTGAAAAATTAGCTTTTTATGATTACATCGGAAATAATCCTGCGAAAGGGGGATTATTCAGAGCGGGTTCAATGGATAACGGGGATGGAATAGCTGTCGGGTGGTTAGGACACCCTATATTTAGAGATAAAGAAGGGCGTGAACTTTTTGTACGTCGTATGCCTACTTTTTTTGAAACATTTCCAGTTGTTTTGGTAGACGGAGATGGAATTGTTAGAGCCGACGTTCCTTTTCGAAGGGCAGAATCGAAGTATAGTGTCGAACAAGTAGGTGTAACCGTTGAGTTCTGTGGTGGCGAGCTGAATGGCGTGAGTTATAGTGATCCTGCTACTGTGAAAAAATATGCTAGACGTGCTCAATTGGGTGAAATTTTTGAATTAGATCGTGCTACTTTGAAATCCGATGGTGTTTTTCGTAGCAGCCCAAGGGGCTGGTTTACGTTTGGACACGCTTCATTTGCTCTGCTTTTCTTCTTCGGACACATTTGGCATGGTGCTAGAACCTTGTTCAGAGATGTTTTTGCTGGTATTGACCCGGATTTGGACGCTCAAGTGGAATTTGGAGTATTCCAAAAACTTGGAGATCCAACTACAAGAAGACAAGTAGTCTGA

>psbN

ATGGAAACAGCAACCCTAGTCGCCATCTCTATATCTGGGTTACTTGTAAGTTTTACTGGGTACGCCTTATATACTGCTTTTGGGCAACCCTCTCAACAACTAAGAGATCCATTCGAGGAACACGGGGACTAG

>psbH

ATGGCTACACAAACAGTTGAGGGTAGTTCTAGAGCTCGTCCAAAAAGAACTTCTGCAGGGGGGTTGTTGAAACCCTTGAATTCGGAATATGGTAAAGTAGCTCCTGGCTGGGGAACTACTCCTTTGATGGGAGTCGCAATGGCTTTATTTGCGGTATTCCTATCTATTATTTTGGAGATTTATAATTCGTCCGTTTTACTGGACGGAATTTCACTGAATTAG

>petB

ATGAGTATGAAATTCTCATATACGGTTCTCAGAGGGGAGTTCTCTTGGTTTACCTATCTCAATAAAGTCTACGATTGGTTCGAAGAACGTCTCGAGATTCAGGCGATTGCAGATGATATAACTAGTAAATACGTTCCTCCTCATGTCAACATATTTTATTGTCTGGGAGGAATTACGCTCACTTGTTTTTTAGTACAAGTAGCTACAGGGTTTGCTATGACTTTTTACTACCGCCCGACCGTTACTGAGGCTTTTGCCTCTGTTCAATACATAATGACGGAAGTTAACTTCGGGTGGTTAATTCGATCGGTTCATCGATGGTCGGCAAGTATGATGGTCCTAATGACAATCCTGCACGTATTTCGCGTGTATCTCACCGGGGGTTTTAAAAAACCTCGCGAATTGACTTGGGTGACAGGTGTGGTTCTGGCTGTATTGACCGCATCTTTTGGTGTAACAGGTTATTCTTTACCTTGGGACCAAATTGGATATTGGGCAGTAAAAATTGTAACAGGCGTGCCCGAAGCAATTCCGGTAATAGGATCTCCTTTGGTAGAGTTATTACGCGGAAGTGCTAGTGTGGGACAGTCCACTTTGACTCGTTTTTATAGTTTACACACTTTTGTATTACCTCTTCTTACTGCCGTATTTATGTTAATGCATTTCCTAATGATACGTAAACAAGGTATTTCTGGCCCTTTATAA

>petD

ATGGGAGTAACAAAAAAACCTGACTTGAATGATCCTGTATTAAGGGCTAAGTTGGCTAAGGGTATGGGTCATAATTATTATGGAGAACCCGCATGGCCCAACGATCTTTTATATATTTTTCCAGTAGTAATTCTAGGTACTATTGCATGTACCGTAGGCTTAGCGGTTCTAGAACCATCAATGATTGGTGAACCCGCGGATCCATTTGCAACTCCTTTGGAAATCTTACCCGAATGGTATTTCTTTCCCGTATTTCAAATACTTCGTACAGTACCCAATAAGCTGTTGGGTGTTCTTTTAATGGTTTCAGTACCTACGGGATTATTAACAGTACCTTTTTTGGAAAATGTTAATAAATTCCAAAATCCATTTCGCCGTCCCGTAGCGACAACCGTCTTTTTGATTGGTACCGCAGTGGCCTTGTGCTTGGGTATTGGAGCAACATTACCTATTGAAAAATCCCTAACTTTAGGTCTTTTTTAA

>rpoA

ATGGTTCGAGAGAAAGTAAAAGTATCTACTCGGACACTGCAGTGGAAGTGTGTTGAATCAAGAGCAGACAGTAAGCGGCTTTATTATGGGCGCTTTATTTTGTCTCCACTTATGAAAGGTCAAGCCGACACAATAGGCATTGCGATGCGAAGAGTTTTGCTTGGAGAAATAGAAGGAACATGTATTACACGCGCAAAATCTGAGAAAATCCCACACGAATATTCTACCATAGTGGGTATTCAAGAATCGGTACATGAAATTTTAATGAATTTGAAAGATATTGTATTGAGAAGTAATCTTTATGGAACTTGTGACGCGCTTATTTGTGTCAAAGGCCCGGGATATGTAACTGCTCAAGACATCCTCTTGCCGCCTTCTGTGGAAATCGTTGATAATACGCAGCACATAGCTAGCCTAACAGAACCAATTGATTTGTCTATTGGATTACAAATCGAGAGGAGTCGAGGATATAATATAAAAACGCCAAATACCTTTCAAGACGGAAATTGTTATCCTATCGATGCTGTATTCATGCCTGTTCGAAATGCGAATCATAGTATTCAGTCTTATGGGAATGGCAATGAAAAACAAGAGATCCTTTTTCTAGAAATATGGACAAACGGAAGTTTAACTCCTAAAGAAGCACTTCATGAAGCCTCCCGGAGTTTGATTGATTTATTTATTCCCTTTCTCCAGGCAGAAGACGAAAACTTACCTTTAGAGAACAATCAATACAAGGTTACTTTACCCTTTTTTACTTTTCATGATAGATTGGCTAAACTAACGAAAAAGAAAAAAGAAATCGCATTGAAATCGATTTTTATTGACCAATCAGAAATGTCTCCCAGGACCTATAATTGTCTCAAAAAGTCCAATATACATACATTATTCGACCTTTTGAATACGCGTCAAGAAGACCTTATGAAAATTGAACACTTTCGCATAGAGGATGTAAAGCAGATAATGAGTATTCTAGAAAAGAAATAG

>rps11

ATGGCAAAATCTCCACCACGAAGTGGTTCACGTAGGCCGGGACGGATCGGTTCACGTAAAAGTGGACGTCGAATACCAAAGGGCGTTATTCATGTTCAAGCAAGTTTCAACAACACCATTGTGACTGTTACAGATGTCCGGGGTCGGGTAATTTCTTGGTCCTCGGCCGGTACTTGTGGATTCAGGGGTACAAGAAGAGGTACGCCTTTTGCTGCTCAAACCGCAGCAGGAAATGCTATTCGAGCAGTAGCGGATCAAGGTATGCAACGAGCAGAAGTCATGATAAAGGGTCCTGGTCTCGGAAGAGATGCGGCATTACGAGCTATTCGTAGAAGCGGTATCCTTTTAAATTTCGTACGGGATGTAACCCCTATGCCACACAATGGTTGCAGACCCCCTAAAAAAAGACGGGTGTAG

>rpl36

ATGAAAATAAGGGCTTCAGTTCGTAAAATTTGTGAAAAATGTCGACTGATCCGCAGGAGGGGACGGATTATAGTAATTTGTTCCAACCCGAGACATAAACAAAGACAAGGATAA

>rps8

ATGGGCAAGGACACTATTGCTGACATAATAACTTCTATACGAAATGCTGACATGAATAGAAAGGGAACGGTTCGAATAGCATCTACTAACATCACCGAAAACGTTGTTAAAATCCTTTTGCGAGAGGGTTTTATCGAAAACGCAAGGAAACTCGTGGAAAACAAAAACAAAAAAGAGTTTTTGGTTTTAACCCTACGACATCGAAGGAATAGGAAAGGGCCGTATAGACCCATTTTAAATTTAAAACGAATCAGTCGACCCGGTCTACGAATCTATTTTAACTATCGACGAATTCCTAGAATTTTAGATGGGATGGGGATTGTAATTCTCTCTACTTCTCGGGGTATAATGACAGACCGAGCGGCTCGACTGGAAAGAATCGGTGGAGAAATTTTGTGTTATATATGGTAA

>rpl14

ATGATTCAACCTCAAACCCATTTGAATGTAGCAGACAATAGCGGTGCCCGAGAATTGATGTGTATTCGAATCATAGGAGCCAGTAATCGTAGATATGCTCATATTGGTGACGTTATTGTTGCTGTGATCAAGGAAGCAGTACCCAATACGCCTCTAGAAAGATCAGAAGTGATCAGAGCTGTAATTGTACGTACTTGTAAAGAACTCAGACGTAATAACGGTATGATAATACGGTACGATGACAATGCTGCAGTTGTCATTGATCACGAAGGAAATCCAAAGGGAACTCGAGTTTTTGGTGCGATCGCCCGGGAATTGAGACAGTTGAATTTTACTAAAATAGTTTCATTAGCACCTGAAGTATTATAA

>rps3

ATGGGACAAAAAATAAATCCACTAGGTTTCCGGCTTGGTACAACACAAAGTCATCATTCTCTTTGGTTTGCAAAACCAAAAAACTATTGCGAGGGTCTACAAGAAGATCAAAAAATACGAAACTTTATTAAGAATTATATAAAAAAAAATATCAGAATATCTTCCGGTGTTGAGGGAATTGCACGGATAGAGATTCAAAAAAGAATTGATCTAATTCAAGTTATAATCTATATAGGGTTCCCAAAATTATTACTAGAAAATAGACCGCGAAGAATTGAAGAATTACAGATGAATGTACAAAAAGAACTTAATTGTGTGAATCGAAAAATAAACATTGCTATTACACGAATTACAAATCCTTATGGGCACCCCAATATTCTTGCCGAATTTATAGCCGGACAATTAAAAAATCGAGTTTCTTTTCGAAAAGCAATGAAAAAAGCTATTGAATTAACGGAACAGGCCGATACAAAAGGAATTCAAGTACAAATTGCAGGGCGTCTTGACGGAAAAGAAATTGCGCGCGCCGAATGGATCAGAGAAGGTAGAGTTCCTCTACAAACCATTGGAGCTAAAATTGATTATTGTTCCTATACGGTTCGAACTATATACGGGGTATTGGGAATCAAAATTTGGATATTTGTAGACGAAAAAAAATAA

>ndhF

ATGGAACATACATATCAATATTCCTGGATCATACCCTTAGTTCCACTTCCAGTCCCTATGTTAATAGGGGTGGGACTTCTATTTTTTCCGACCGCAACAAAAAATCTTCGCCGTATGTGGGCTTTTATTAGTATTTTATTGTTAAGTATAGTTATGATTTTTTCGATCGATCTATCTATTGAGCAAATAGATAGAACTTCGATCTATCAATCCCTAAGGACTTGGACCATCACTAGTGATTTGTCTTTCGAGTTCGGATACTTTATTGATCCACTTACTTCTATTATGTCAATATTAATCACTACAGTTGGAATTCTGGTTCTTATTTATAGTGACAATTATATGTCTCATGATCAAGGATATTTGAGATTTTTTGCTTATATGAGTTTTTTCAATGCTTCAATGTTAGGATTAGTTACAAGTTCGAATTTCATACAAATTTATATTTTTTGGGAATTGGTTGGAATGTGCTCTTATCTATTAATCGGGTTTTGGTTCACACGACCTATTGCGGCAGGCGCCTGTCAAAAAGCATTTGTAACTAATCGTGTAGGGGATTTTGGATTATTATTAGGGATCTTAGGTCTTTATTGGCTAACAGGCAGTTTCGAATTTCGGGATTTGTTCGAAATATTGAAAAACTTGATTTATAATAATGAGGTTAACCTTTTATTTGTTACTTTGTGTGCATTTCTATTATTTGCCGGCCCGGTTGCTAAATCCGCGCAATTCCCTCTTCATGTATGGTTACCCGATGCCATGGAAGGGCCTACTCCTATTTCGGCTCTTATCCATGCTGCTACTATGGTAGCGGCGGGAATTTTTCTTGTAGCTCGGCTTCTTCCACTTTTCATAGTCATACCATACGCAATGAATCTAATATCTTTGATAGGGATAATAACAGTATTTTTAGGAGCTACTTTAGCTCTTGCTCAACAAGATATTAAGAGAGGTTTAGCTTATTCTACAATGTCTCAATTGGGTTATATGATGTTAGCTCTAGGTATGGGGTCCTATCGAGCCGCTTTATTTCATTTGATTACTCATGCCTATTCCAAAGCCTTGTTGTTTTTAGGCTCCGGATCAATTATTCATTCAATGGAAGCTATTGTTGGATATTTTCCAGATAAAAGCCAGAATATGGTTCTTATGGGTGGGTTAAGAAAGCATGTGCCGATTACAAAAACCGCTTTTTTAGTAGGTACTCTTTCTCTTTGTGGTATTCCACCTCTCGCCTGTTTTTGGTCCAAGGATGAAATTCTTAATGATACTTGGTTGTATTCGCCGATTTTCGCAACAATAGCTTTTTTCACAGCCGGACTAACCGCATTTTATATGTTTCGAATTTATTTACTTACTTTTGAGGGGCCTTTCAACTTTTGCTTGCAAAATTACAGTGGCAAAAAAAGAAATTCCTTATATTCAATATCTCTATGGGGTAAAGAAGAACCAAAACCGATTAAAAACAAATTTCATTTAGTTGCTTTATTAACAATGAATAATAATAAAAGGGCTTCTTTTTTTGCGAAGAAGACTCATCGAATTGCTAGTACTGTAACAAATATGCCCTTTATTACTATTTTTCCTTTTGGCGCTGCCAAGACGTTTTGTTATCCTCACGAATCAGACAATACTATATTATTTGTTATGCTTGTATTAGTCCTATTTCCTTTGTTTGTTGGAGCGATAGGAATTTCTTTGAATCAAGAAGTAATCGAGTCGGATATTTTATCAAAATTGTTAACTCCGTCTATAAATCTGTTACATCAAAATTCAACTCATTTTGTTGATTGGTATGAAGTTGTAAAAAATCCAACTCTTTCCGTCAGTATAACGTATTTAGGAATACTTCTAGCCTACTTTTTATATAAACCCTTTTATTCATCTTTACACAATTGGAACATATTGAATTTTTTTGCTAAAAGAGGGCCTAAGAGAATTCTTTGGGACAAAATACTCAATTTTCTATATGATTGGTCATATAATCGTGCTTATATAGATGCTTTTTACACAAGATCCTTAACAGAAGGTATAAGAGGATTAGCGGAACTAACTCATTTGTTCGACAGACGAGTAATTGATGGAATTACGAATGGGGTTGGTATTACAAGTTTTTTTGTAGGGGAAGGTATAAAATATTTAGGGGGAAGTCGCATCTCTTTTTATCTCTTATTATATTTATTTTCGTTATTACTCTTTTTAATATTTCTCTTCTTTTTTTTCTTTAAATAA

>rpl32

ATGGCAGTTCCAAAAAAACGTACTTCTATATTAAAAAAACGTATTCGTAAAAATCTTTGGAAAAAGGGGGGGTACTGGGCAGCGTTGAAGGCTTTTTCGTTAGCGAAATCCCTTGCTACTGGGAATTCAAAAAGTTTTTTTTGTACAACAAATAAATAA

>ccsA

ATGATATTTTCAACTTTGGAGCGTATATTAACGCATATATCCTTTTCGGTCGTTTCAATTGGAATTACAATTTATTTAATAACCTTCTTAGTCGATGAAATCAGAGGGCTATATGCTTCATCAGAAAGGGGGATGACAGCTACCGCTTTCTGTCTAACAGGATTATTAATCACCCGTTGGGTTTACTCGAGACATTTCCCATTAAGTGATTTATATGAATCATTAATCTTTCTTTCCTGGAGTTTATCTATTATTCATAAGATTTTTGATTTTAAAAATAATCAAAATCATTTAAGCGCTATAACGGCACCAAGTGCTTTTTTTACCCAAGGCTTTGCGACTTCGGGTTTTTTAACCAAAATGCATCAATCCAGAATATTAGTACCCGCTCTCCAAGTCCAGTGGTTAATGATGCACGTAAGTATGATGGTATTGGGCTATGCAGCTCTTTTATGTGGATCATTATTATCCACGGCTCTTCTAGTCATTACATTTCGAAAAGTGATAAGGCTTTTTTTGAAAAGAAAAAATTTTGTAAATGTAAATGGGTCATTTTGTTTCAGTGAAATCCAATACATGAACGAAAAAAAGAATGTTTTTCTAAATAGTTTTTCCGCTAGAAATTATTACAGGTATCAAGTGATTCAACAATTGGATCGTTGGAGTTATCGTATTATTAGTTTAGGATTTATCTTTTTAACCACAGGTATTCTTTCGGGAGCAGTATGGGCTAATGAGGCGTGGGGGTCTTATTGGAATTGGGATCCAAAAGAAACTTGGGCATTTATTACTTGGACGATATTCGGGATTTATTTACATACTCGAACAAATACAAAATGGGAAGGTGTAAATTCCGCAATTGTGGCTTCTATGGGCTTTCTTATAATTTGGATATGCTATTTCGGAGTCAATCTATTAGGAATAGGGTTACATAGTTATGGTTCATTTAATTAA

>psaC

ATGTCACATTCAGTAAAGATTTATGATACATGTATAGGGTGTACTCAATGTGTACGAGCTTGCCCCACGGATGTATTAGAAATGATACCTTGGGACGGATGTAAAGCAAAGCAAATTGCTTCTGCTCCAAGAACAGAGGACTGTGTTGGTTGTAAGCGATGTGAATCCGCCTGTCCAACGGATTTCTTGAGTGTTCGAGTTTATTTATGGCATGAAACAACCCGAAGCATGGGTCTAGCTTATTGA

>ndhE

ATGATGCTCGAGCATGTACTTGTTTTGAGTGCCTATTTATTTTCGATTGGCATCTATGGCTTGATTACGAGCCGAAATATGGTTCGGGCCTTGATGTGCCTTGAACTTATACTAAATGCAGTTAATATCAATTTTGTAACATTCTCTGATTTTTTTGATAGTCGACAATTAAAAGGAGATATTTTTTCAATTTTTGTTATAGCTATTGCAGCCGCTGAAGCAGCTATCGGATCAGCTATTGTTTCGTCAATTTATCGTAACAGAAAATCGACGCGTATCAATCAATCGACTTTGTTGAATAAGTAG

>ndhG

ATGGATTTGCCTGGACCAATACACGATTTTCTTTTAGTTTTTCTGGGATCGGGTCTTATATTAGGAGGTCTGGGAGTGGTATTATTTACCAACCCAATTTATTCTGCCTTTTCCTTGGGATTGGTTCTTGTTTGTATATCATTATTCTATATTCTATCAAATTCCCATTTTGTAGCTGCCGCGCAACTCCTTATTTACGTGGGAGCTGTAAATGTTTTAATCATATTTGCTGTAATGTTCATGAACGGCTCAGACTATTCCAAAGATTTTCAGTTGAATCTTTGGACTATTGGCGATGGTCTTACTTCTCTGGTTTGTACAAGTATTTTTTTTTCGCTAATCACTACTATTCTCGATACATCGTGGTACGGGATTATTTGGACTACACGAGCCAACCAGATTATTGAACAAGATTTGATAAGTAATAGTCAACAAATTGGAATTCATTTATCAACAGACTTTTTTCTTCCATTTGAACTCGTTTCAATAATTCTTTTAGTTGCTTTAATAGGTGCAATTGCCGTGGCGCGTCAATAA

>ndhI

ATGTTCCCTATGGTAACCGGTTTCATGAATTATGGTCAACAAACAATACGAGCTGCAAGGTACATTGGTCAAAGTTTCATGATTACTTTATCCCAAGCAAATCGTTTACCTGTAACTATTCAATATCCTTATGAAAAATTAATCCCATCAGAGCGTTTTCGTGGTCGAATCCATTTTGAATTTGATAAATGTATTGCTTGTGAAGTATGCGTTCGGGTATGTCCTATAGATCTGCCTGTTGTTGATTGGAAATTTGAAACAGATATTCGAAAGAAACGATTGCTTAATTACAGTATTGATTTTGGAATTTGTATTTTTTGTGGTAACTGCGTTGAGTATTGTCCAACAAATTGTTTATCAATGACTGAAGAATATGAACTTGCGACTTACGACCGTCACGAATTGAATTATAATCAAATTGCTTTAGGTCGTTTACCAATGTCAGTAATTGACGATTTTACAATTCGAACAGTCTTGAATTCGCCTCAACGAAAAAACGTCTAA

>ndhA

ATGATAATTGCTACACCCGAAGTACAAGATATCAATTCTTTTTCCCGATTGGAATCCCTACAAGAGGTCTATGGGATCCTATGGGTGCTTGCCCCTATTTCGATTTATGTATTGGCAATCACAATCGGTGTCCTAGTAATTGTGTGGTTAGAAAGAGAAATATCTGCAGGAATACAACAGCGTATTGGGCCTGAATACGCCAGCCCTTTGGGAATTCTTCAAGCTTTAGCCGATGGGACAAAACTCCTTTTCAAAGAAAACCTTCTTCCATCTAGAGGAAATAGTAGTTTATTCAGTATTGGTCCATCTATAGCAGTCATAGCAATTCTACTAAGTTATTCAGTAATTCCTTTTAGTTATAACCTTGTTTTAGCTGACCTCAATATCGGTATTTTTTTATGGATTGCCATTTCAAGTATTGCCCCTATTGGACTTCTTATGTCAGGATATGGATCAAATAATAAATATTCCTTTTTAGGTGGTCTGCGAGCTGCTGCTCAATCGATTAGTTATGAAATACCATTAACTTTATGTGTTTTATCAATATCTCTATTATCTAACAGTTTAAGTACAGTTGATATAGTTGGGGCGCAATCAAAATATGGTTTTTGGGGGTGGAATTTGTGGCGTCAACCTATAGGGTTTATCGTTTTTCTAATTTCTTCCCTAGCGGAATGCGAGCGATTACCTTTTGATTTACCAGAAGCGGAAGAAGAATTAGTAGCCGGTTATCAAACCGAATATTCAGGAATCAAATTTGGTTTATTTTACGTTGCTTCCTATCTAAATCTACTAGTTTCCTCATTATTTGTAACAGTTCTTTACTTGGGAGGTTCGAATCTTTCCATTCCATACATATTTGTTCCTGGACTGGTTGAAATAAATAAAGCGGATGGAATCTTTGGAACGACAATTGGTATCTTTATTACATTAGCTAAAACTTATTTGTTCTTGTTCATTCCTATTGCAACAAGGTGGACTTTACCGAGACTAAGAATGGACCAACTATTAAATCTTGGCTGGAAATTTCTTTTACCTATTTCTCTCGGTAATTTATTATTAACAACTTCTTCCCAACTCCTTTCGCTATAA

>ndhH

ATGAGTATACTAGCTACAGAAAAAGAAAAAGAATTTATGATAGTCAATATGGGACCTCACCACCCGTCAATGCATGGGGTTCTTCGTCTCATCGTTACTCTAGACGGTGAAGATGTTATTGACTGTGAACCAATATTGGGTTATTTACACAGAGGGATGGAAAAAATTGCGGAAAACCGAACAATTATACAATATCTGCCTTATGTAACCCGTTGGGATTATTTAGCTACTATGTTCACAGAAGCAATAACTGTAAATGGACCAGAACAGTTGGGAAATATTCGCGTACCTAAAAGGGCCAGCTATATCAGAGTAATTATGTTGGAGTTGAGTCGTATAGCTTCCCATCTGTTATGGCTTGGCCCTTTTATGGCAGATATTGGTGCACAGACTCCTTTCTTCTATATTTTCAGAGAAAGAGAATTAGTATATGATCTGTTCGAAGCTGCCACCGGTATGAGAATGATGCATAATTATTTTCGTATCGGAGGAGTAGCAGCTGATTTACCCTATGGTTGGATAGATAAATGTTTGGATTTCTGCGATTATTTTTTAACAGGGGTTGCTGAATATCAAAAACTTATTACGCGAAACCCCATTTTTTTAGAACGAGTTGAAGGAGTAGGCATTATTGGTGGAGAAGAAGCAATAAATTGGGGTTTATCAGGACCAATGCTACGAGCGTCTGGAATAGAATGGGATCTTCGTAAAGTTGATCATTATGAGTGTTATGACGAATTTGATTGGGAAGTCCAGTGGCAAAAAGAAGGGGATTCCTTAGCTCGTTATTTAGTCCGAATCGGTGAAATGACGGAATCTGTAAAAATTATTCAACAGGCTTTAGAAGGAATTCCGGGAGGCCCCTATGAAAATTTAGAAATCCGCTGCTTTGATAGAGAAAGCGATCCAGAACGGAATGATTTTGAAAATCGATTCATTAGTAAAAAGCCTTCTCCTACCTTTGAATTGACAAAACAAGAACTTTATGCGAGAGTAGAAGCCCCAAAAGGAGAATTGGGAATTTTTCTGATAGGGGATCAAAGTGGGTTTCCTTGGAGATGGAAAATTCGCCCACCGGGTTTTATCAATTTGCAAATTCTTCCTCAGTTAGTTAAAAGAATGAAATTGGCTGATATTATGACGATATTAGGTAGTATAGATATCATTATGGGGGAAGTTGATCGTTGA

>rps15

ATGGTAAAAAATGCATTCATGTCAGTTAGGGTTCAAGAAAAAAAAGAAAAAAACAGCGGATCGGTTGAATTTCAAGTATTTCGTTTCACCAACAAGATCCGGAGACTTACTTCACATTTAGAATTGCACAGACAAGACTATTCATCTCAAAGGGGTCTACGTAAAATTTTGGAAAAACGCCAACGTCTACTAGCTTATTTGTCAAAGAAAAATAGAGTACGTTATAAAGAATTAATTAGTAAGTTGAATATCCGGGAGTCAAAAAATCGTTAA

>ycf1

ATGATTTTTCAATCTTTTATACTAGGTAATCTAGTATCCTTATGCATGAAGATAATCAATTCGGTCGTTGTGGTCGGACTCTATTATGGATTTATGACCACATTCTCCATAGGGCCCTCTTATCTCTTCCTTCTCCGAGCTCGGGTTATGGAAGAAGGAGAAGAAGGAACCGAGAAGAAGGTATCAGCAACAACTGGTTTTATTGCGGGACAGCTCATGATGTTCATATCGATCTATTATGCGCCTCTGCATCTAGCATTGGGTAGACCGCATACAATAACTGTCCTAGCTCTACCGTATCTTTTGTTTCATTTCTTCTGGAACAATCCCAAACACTTTTTTGATTATGGATCTACTACCAGAAATTCAATGCGTAATCTTAGCATTCAATGTGTATTCCTGAATAATCTCATTTTTCAATTATTCAACCATTTCCTTTTACCAAGTTCAATGTTAGCCAGATTAGTCAACATTTATATGTTTCGATGCAACAACAAGATGTTATTTGTAACAAGTAGTTTTGTTGGTTGGTTAATTGGTCACATTTTATTCATGAAATGGGTTGGATTGGTATTAGTTTGGATGCAGCAAAAGAATTCTATTAGGTCTAATGTACTTATTCGATTTAATAAGTACCTTGTGTCAGAATTGAGAAATTCTATGGCTCGAATCTTTAGTATTCTCTTATTTATTACCTGTATCTACTATTTAGGCAGAATACCCTCACCCATTTTTACTAAGAAACTGAAAGTGAAAGAAACCTCAGAAACGGAAGAAAGAGATGTAGAAATAGAAAAAACTTTCGAAAGGGGGGGGACTAAACAGGGGCAAGAGGTATCCGCCGAAGAAGATCCTTCTCCTTCCCTTTTTTCGGAAGAAAAGGAGGATCCGGACAAAATCGAGGAAACGGAAGAGATCCGAGTGAATGGAAAGGAAAAAAAAAAAACAAAGCATGAATTCAACTTGCGCTTTAAAGAGACATGCGATAAAAATAGCCCTGTTTATGAAACTTCTTATCTGGATGGGAATAAAGAAAATTCAAAATTAGAAATATTTCAATTATTTAAAGAAAAAAAAGAAGAGAAATATTTATTATGGTTTGAAAAACCTCTTGTGACCCTTCTTTTCGACTATAAACGTTGGACTCGGCCACTTCGATATAAAAAAAATAATAGATTTGAAAATGCTGTACGAAACGAAATGTCACATTATTTTTTTTATGCATGTCGAAGTGATGGAAAAGAAAGGATCTCTTTTACATATCCAGCTAGTTTGTCAACTTTTTTGGACATGATAAAAAAAAATAATTATTTTTTCGCAACGGAAAAACTATCCTCTGATGAATTTTATACACATTGGAATTACACCAATAACCAAAAAATTAAGAACTTAAGCAAGGAGTTTAGAAATCGACTTGAGGGTTTAGATAAAGGATCTCTTATTCGGGATATACTCGAAAAAAGGACTCAATTGTGTAATGATAAGACTAAAAAAAAATACTTACCTAAAACATACGATCCTTTATTAAACGGACCTTATCGTGGAAGAATAAAAAAATTCTTTTCACCCCCAATCCTAAATACAACTTATATCAAAAATAAGATAAGAACGCCTTGGATAAATAAAATTCACAATCTAATTTTTATTAATGATTATCACGAATTTGAACAGACAATAGACCGATTTAATCGAAACTCGTTTCCAAGCGAAGAAGTGCGGTCTTTATTGACAGAACGGGAACGAGAACACATCGATTCCGAAGACCGAATAAAAAGTTTCAATTTTTTATTCGATGCAGTTATAACGGATCCCAATGATCAAAAAATTCGAAAAAAAGCGATAAAAGAAATTAGTAAAAGAGTTCCCCGGTGGTCATACAAATTAATCGATAATGTATACCAAGAACTGGGAGAATACGACGAAAATGTAAGAGGGGAACATGCATTTCGTTCACGAAAAGCCAAACGTTTAGTGGTTTCTGTTGATCACCAGACAAAAGAGAATGTGACTTTGCCCCATTATTTGGAACAATCGGATTTTCGTCGATATATAATAAAAGGTTCCATGCGCGCACAAAGACGTAAAACCGTTATTTGGAAACCAGTTCAAGCAAATGCCCATTCCCCTCTTTTTTTGGACAGAATAGACAAAACCCTTTATTTGTATTTTGATATTTCCCAGCTGATGAAAGTAGTTCTTCGAAATTGGATGGTAAAAAATAAAAACCAAAAACTTTCTGATTATACAAACGCAAAGACAAGAAAATTGGACAAAAAAGAAAAAAAGAAGCTCAAAGGCGAAAGATACCAAAGACAAGAAATGGTACGTATAAAACAAGCAGAAGGCTGGGATAAGCGTTTACTTACTCGACTACTAAGAAGTTCTATGTTAGTAATCCAATCGATTCTTAGAAAATATATTGTATTACCGTTATTGATAATAGCTAAAAATGTGGTTCGCATACTATTATTCCAAGATCCCGAGTGGTCCGAGGATTTTAAGGATTGGAATCGTGAAATTTATGTTAAATGCACTTATAGTGGTGTTAATTTATCTGAAACAGAATTTCCGAAAAACTGGTTAATAGAAGGTATTCAGATAAAGATCCTATTCCCCTTTCACCTGAAACCCTGGCACGGATCTACGATACAATCCTCTCATAAAGATCCAAAAAGTGAACAAGAAACTGATTTTTGTTTTTTAACAATTTTGGGGCTGGAAACTGACATGCCGTTCGGTTCTCCCCAAAAACGACGTTCCTTTTTTGAACCCATTTTTAAAGAACTAAAAAAAAAAATTAGAAAATTGAAAACTAAGTCTTTTCTAGTTTTAAGGGATTTTAAAGAAGGAAAAATAAGAGAACTTTCAAAAATAAACTTGAGAGAAATCGAGAAATTGAGGGAAACTCAAAAAAATTCGATAATCAGTAAGCAGATGATTCACGAACCGTCTATTGAAATTTCATCTATGGATTGGACGAAATTATCCCGGACTGAAAAAAAAATGAAAGATCTGACTAATAGAACAAGCAGAATCAGAAATCAAATATATAAAATTACAAAAGAAAAGAAAAAAGGATCGCTAACTCAAGAAACAAATATTAGTTCGAACAAACCAACTTATTCTGTTACAATATTAGACCCATCAAAAAGGATTTGGCGGATATTAAAAAAAAGAAACGCTCGATTAATCCGTAAATCCTATTTGTTTCTAAAATTTGGCATTGAAAAGATATACAGAAATATTTTTATATCTACCCTTACTATTCCAAGAATCAATACAAAACCTTTTCGTGAATCAACAAGAAAACAAATGAAAATTATTGAGAAAAACATCCACAATAATGAAGCAAATCCCGAAAGAATTAATAAAACAAATAAAAATAGAATTATTTCGACTATCCAAAAATCGATTTCTAAGACTAGTAATAAGAATTCAAAGATTTCTTGTAATTTATTGTCTTTTTCACAATCACAAGCATATGTATTTTACAAATTATTACAAGTCCCAATTTTGAACTTTTATAATTTAAGACCCGTTCTTCAATATCACGGAACATCTCTATTTCTTAAGAATGAAATAAAAGATTTTTTTGAAAAACACGGAATCTTTAATTACCAATTAAGACATAAACCCCTTTGGAATTTTGGAAGGAATACACGAAAACACTGTTTAAGCGGGCATTATCAATATGATTTATCTCGGATTAAATGGGCTAGATTAGTACCACAAGAATGGCGAAATAGAGCCAATCAACACTGTATGGCTCAAAATAAAGATTTAATTAAAAGAGATTCGTATGAAAAAAATGGATTAACTCATTACGAAAAACAACATTTTTTTGAAGCGGACCTATTACGTAATCAAAAATCGAATTTTAAAAAACACTATAGATATGATCTTTTATCATATAAATCGCTTAATTATGAAGATAAGAAAGACTCGTATATTGATAGATCACTAGTCCAAGTAAATAATAAAGAAGAGTATTATTCTAATTACAATAGAAAGAAAGTCAAATTGTTGGCTATGCTGGGAAGTATCTCGATCAATAATTATATAGGGGAAGATGATATTATGGATATGGAAAAATTCTTGTATAGAAAATATTTTGATTGGAGAATTCTTAATTTTTGTCTTAAAAATAAGGCCAATATGGAAGCCTGGGTTGATATGGATACTGGTACCAGCAGTAATCAAAATACTAGGATTGGGTCCAATAATTATCAAAAAATTAATGCAATTAATAAGAGAGTCCCCTTTTATCTTACAATTCATCTTGATGAAGAAATTAACCCATCCACTCAAAAGGGGTTCTTTTGTGATTGGATGGGAATGAATGAAGAAATACTAAGTTGTCCTATATCAAACCCAGAATCTTGGTTCTTCCCAGAATTTGTACTACTTTTTAATGCATATAGAACGAAACCCTGGATTATACCAATCAAATTACTTCTTTTCAATTTTAATGGAAATAGTAAGAAAAATAGAACCGGAAAGAAAGAGGCGGATCTTTTTATATCACCTACTCAAAAAGAATATTTTGAATTATCGAATCAAAGTAAAGAAGAAAACGAACTCGCAGACCAAGGAACTCCGCGATCGGATGCACAAAAGCAAGTAATTCTTGGATCAGTTCTCTCAAACCAAGAAAAAGATGGTGAAGAAAATTATACGGGATCGGACATGAAAACCCGTATAAAGAAAAAGCAATACAAAAGAGAAACCGAAGTACAGCTCGATTTCTTCCTAAAAAGATATTTGTGTTTGCAGTTGCGATGGAGGGGTGCTGTTTCTTTCAGAGAAAAAATACTCAATGATATGAAAGTATATTGTCACCTGGTTCGACTAATAAATCCTAGCGACGTTACTATAGCCTCTATTCAAGGGGGAGAAATTAGTCTGCCTATTTTGATCACTAAGAAGAATTTCGCTCTTAAAGAATTGACGAAAGGGGGAATGCTTATTATCGAACCCCGTCGTTTGTCTGTAAAAAATGATGGACAATTTTTTCTATATCAAATCGTAGGTATTGCATTGGTTCATAAGAATAAGCGCAAAATTACTAAAAGATACCAAGAAAAGGGCTATGTTGATAAAAAAGATTTTGATGAATTCATTGCAAAACATCAAAAAATGATTGGAAATAGAAACAAAAATCATTATGATTTGCTTGTTCCTGAAACTATTTTACTCCCTAAACGTCGTAGAGAATTAAGAACCCTAATTTGTTTCAATTCGAAGAATCAAAATGGTATGCAGAAACATCCAGTATTTTTTAATAACGGAAAGGGCGGCGGCCGCGTTTTGGATAACAACAAAAATATTGCTCGAGAGAAAAATCAACTAATTCAATTAAAGTTCTTTATTTGGGCCAATTCTCGATTAGAAGATTTAATTTGTATGAATCGGTATTGGTTTAATACCAATAATGGGAGTCGTTTCAGTATGGTAAGGGTCCATATGTATCCACGATTGAAAATTCGTCAATAG

>ycf68

ATGGCGTACTTCTCCTGTTCGAACCGGGGGTTTGAAAACAAACTCCTCCTCAGGAGGATAGATGGGGCGATTCAGGTGAGATCCAATGTAGATCCAACTTTCTATTCACTCGTGGGATCCGGGCGGTCCGGGGGGGACCACCACGGCTCCTCTCTTCTCGATAATCCATACATCCCTTATCAGTGTATGGACAGCTATCTCTCGAGCACAGGTTTAGGTTCGGCCTCAATGGGAAAAAAAATGGAGCACCTAACAACGTATCTTCACAGACCAAGAACTACGAGATCGCCCCTTTCATTTCATTCTGGGGTGACGGAGGGATCGTACCATTCGAGCCTTTTTTTTTTTTCATGCTTTTCCCGGAGGTCTGGAGAAAGCTGCAATCAATAG

>rps7

ATGTCACGTCGAGGTACTACAGAAGAAAAAACTGCAAAATCCGATCCAATTTATCGTAATCGATTAGTTAACATGTTGGTTAACCGTATTCTGAAACACGGAAAAAAATCATTGGCTTATCAAATTATCTATCGAGCCTTGAAAAAGATTCAACAAAAGACAGAAAAAAATCCACTATCTGTTTTACGTCAAGCAATACGTGGAGTAACTCCCGATATAGCAGTAAAAGCAAGACGTGTAGGCGGATCGACTCATCAAGTTCCCATTGAAATAGGATCCGCACAAGGAAAAGCACTTGCCGTTCGTTGGTTATTAGGGGCATCCCGAAAACGTCCGGGTCGAAATATGGCTTTCAAATTAAGTTCCGAATTAGTGGATGCTGCCAAAGGGAGTGGCGATGCCATACGCAAAAAGGAAGAGACTCATAGAATGGCAGAGGCAAATAGAGCTTTTGCACATTTTCGTTAA

>ndhB

ATGATCTGGCATGTACAGAATGAAAACTTCATTCTCGATTCTACGAGAATTTTTATGAAAGCCTTTCATTTGCTTCTCTTCGATGGAAGTTTTATTTTCCCAGAATGTATCCTAATTTTTGGCCTAATTCTTCTTCTGATGATCGATTCAACCTCTGATCAAAAAGATATACCTTGGTTATATTTCATCTCTTCAACAAGTTTAGTAATGAGCATAACGGCCCTATTGTTCCGCTGGAGAGAAGAACCTATGATTAGCTTTTCGGGAAATTTCCAAACGAACAATTTCAACGAAATCTTTCAATTTCTTATTTTACTATGTTCAACTCTATGTATTCCTCTATCCGTGGAGTACATTGAATGTACAGAAATGGCTATAACAGAGTTTCTGTTATTCGTATTAACAGCTACTCTAGGAGGAATGTTTTTATGCGGTGCTAACGATTTAATAACTATCTTTGTAGCTCCAGAATGTTTCAGTTTATGCTCCTACCTATTATCTGGATATACCAAGAAAGACGTACGGTCTAATGAGGCTACTATGAAATATTTACTCATGGGTGGGGCAAGCTCTTCTATTCTGGTTCATGGTTTCTCTTGGCTATATGGTTCATCCGGGGGCGAGATCGAGCTTCAAGAAATAGTGAATGGTCTTATCAATACACAAATGTATAACTCCCCGGGAATTTCAATTGCGCTTATATTCATCACTGTAGGAATTGGGTTCAAGCTTTCCCTAGCCCCTTCTCATCAATGGACTCCTGACGTATACGAAGGATCTCCCACTCCAGTCGTTGCTTTTCTTTCTGTTACTTCGAAAGTAGCTGCTTCAGCTTCAGCCACTCGAATTTTCGATATTCCTTTTTATTTCTCATCAAACGAATGGCATCTTCTTCTGGAAATCCTAGCTATTCTTAGCATGATATTGGGGAATCTCATTGCTATTACTCAAACAAGCATGAAACGTATGCTTGCATATTCGTCCATAGGTCAAATCGGATATGTAATTATTGGAATAATTGTTGGAGACTCAAATGGTGGATATGCGAGCATGATAACTTATATGCTGTTCTATATCTCCATGAATCTAGGAACTTTTGCTTGCATTGTATTATTTGGTCTACGTACCGGAACTGATAACATTCGAGATTATGCAGGATTATACACAAAAGATCCTTTTTTGGCTCTCTCTTTAGCTCTATGTCTCTTATCCCTAGGAGGTCTTCCTCCACTAGCAGGTTTTTTCGGAAAACTCCATTTATTCTGGTGTGGATGGCAGGCAGGCCTATATTTCTTGGTTTCAATAGGACTCCTTACGAGCGTTGTTTCTATCTACTATTATCTAAAAATAATCAAGTTATTAATGACTGGACGAAAGCAAGAAATAACCCCTCACGTGCGAAATTATAGAGGATCCCCTTTAAGATCAAACAATTCCATCGAATTGAGTATGATTGTATGTGTGATAGCATCTACTATACTAGGAATATCAATGAACCCGATTATTGCAATTGCTCAGGATACCCTTTTTTAG

>ycf15

ATGCTACTGCTGAAACATAGAAGAATTGAAATCTTAGATCAAAACACTATGTATGGATGGTATGAACTGCTTAAACAAGAATTCTTGAACAGCGAACCACCAGAGCTATTACTAACTACATCAAAAAATTTCCATTAA

>ycf2

ATGAAAGGACATCAATTCAAATCCTGGATTTTCGAATTGAGAGAGATATTGAGAGAGATCAAGAATTCTCACTATTTCTTAGATTCATGGACCCAATTCAATTCAGTGGGATCTTTCATTCACATTTTTTTCCACCAAGAACGTTTTATAAAACTCTTGGATCCACGAATTTGGAGTATCCTACTTTCACGCAATTCACAGGGTTCAACAAGCAATCGATATTTCACGATCAAGTGTGTAGTACTGTTTGTAGTAGCGATCCTTATATATCGTATTAACAATCGAAATATGGTCGAAAGAAAAAATCTCTATTTGACAGGGCTTCTTCCTATACCTATGAATTCCATTGGACCCAGAACTGATACATTGGAAGAATCTTTTGGGTCTTCCAATATCAATAGGTTGATTGTTTCGCTCCTGTATCTTCCAAAAGGAAAAAAGATCTCTGAGAGCTTTTTCCTGGATCCGAAAGAGAGTACTTGGGTTCTCCCAATAACTAAAAGGTGTATCATGCCTGAATCTAACTGGGGTTCGCGGTGGTGGAGGAACTGGCTCGGAAAAAAGAGGGATTCTAGTTGTAAGATATCTAATGAAACCGTCGCTGGAATTGAGATCTCATTCAAAGAGAAAGATCTCAAATATCTGGAGTTTCTTTTTGTATATTATATGGATGATCCGATCCGCAAGGACCATGATTGGGAATTGTTTGATCGTCTTTCTCCGAGTAAGAGGCGAAACATAATCAACTTGAATTCGGGACATCTATTCGAAATCTTAGTGAAAGACTGGATTTGTTATCTCATGTTTGCTTTTCGTGAAAAAATACCAATTGAAGTGGGGGGTTTCCTCAAACAACAAGGGGCTGGGTCAACTATTCAATCAAATGATATTGAGCGTTTTTCCCATCTCTTCTTGAGAAACAAGTGGGCTATTTCTTTGCAAAATTGTGCTCAATTTCATATGTGGCAATTCCGCCAAGATCTCTTCGTTAGTTGGGGGAAGAATCCGCACGAATCGGATTTTTTGAGGAACATAGCGAGAGAGAATTGGATTTGGTTAGACAATGTGTGGTTGGTAAACAAGGATCGATTTTTTACCAAGGTACGGAATGTATCGTCAAATATTCAATATGATTCCACAAGATCTAGTTTCGTTCAAGTAACGGATTCTAGCCAATTGAAAGGATCTTCTGATCAATCCAGAGATCATTTCGATTCCATTAGTAATGAGGATTCGGAATATCACACATTGATCAATCAAAGAGAGATTCAACAACTAAAAGAAAGATCGATTCTTTGGGATCCTTCTTTTCTTCAAGCGGAACGAAGAGAGATAGAATCAGACCGATTCCCTAAATGTCTTTCTGGATATTCCTCAATGTCCCGGCTATTCACGGAACGTGAAAGGCAGATGAATAAGCATCTGCTTCCGGAAGAAATCGAAGAATTTCTTGGGAATCCTGCAAGATCCATTCGTTCTTTTTTCTCTGACAGATGGTCAGAACTTCATCTGGGTTCGAATCCTACTGAGAGGTCCACTAGAGATCAGAAATTGTTGAAGAAAGAAGAGGATGTTTCTTTTGTCCCTTCCAGGCGATCGGAAAATAAAGAAATAGTTAATATATTCAAGATAATTACGTATTTACAAAATACCGTCTCAATTCATCCTATTTCATCAGATCCGGGATGTGATATGGTTCCGAAGGATGAACTGGATATGGACAGTTCCAATAAGATTTCATTCTTAAACAAAAATCCATTTTTTGATTTATTTCATCTATTCCATGACCGGAACAGGGGGGGATCCACGTTACACCACGATTTTGAATCAGAAGAGAGATTTCAAGAAATGGCAGATCTATTCACTCTATCAATAACCGAGCCGGATCTGGTGTATCATAAGGGATTTGCCTTTTCTATTTTTTCCTACGGATTGGAGCAAAAACAATTCTTGAATGAGGTATTCAACTCCAGGAATGAATCGAAAAAGAAATCTTTATTGGTTCTACCTCCTATTTTTTATGAAGAGAATGAATCTTTTTATCGAAGGATCAGAAAAAAATGGGTCCGGATCTCCTGCGGGAATGATTTGGAAGATCCAAAACCAAAAATAGTGGTATTTGCTAGCAACAACATAATGGAGGCAGTCAATCAATCTAGATTGATCCGAAATCGGATTCAAATCCAATATAGCACCTATGGGTACATAAGAAATGTATTGAATCGATTCTTTTTAATGAATAGATCCGATCGCAACTTCGAATATGGAATTCACAGGGATCAAATAGGAAATGATACTCTGAATCATAGAACTATAATGAAATATACGATCAACCAACATTTATCGAATTTGAAAAAGAGTCAGAAGAAATGGTTCGCTCCTCTGATTTTGATTTCTCGAACCGAGAGATTCATGAATCGGGATCCTAATGCATATAGATACAAATGGTCCAATGGGAGCAAGAATTTCCAGGAGCATTTGAAACATTTCGTTTCTGAGCAGAAGAGCCGTTTTCAAGTAGTGTTCGATCGATTACGTATTAATCAATATTCGATTGATTGGTCTGAAGTTATCGACAAAAAAGATTTGTCTAAGCCACTTCCTTTCTTTTTGTCCAAGTTTCTTTTCTTTTTGTCTAACTCACTTCCTTTTTTCTTTGTGAGTTTCGGGAATATCCCCATTCATAGGTCTGAGATCCACATCTATGAATTGAAAGGTCCGAATGATCAACTCTGCAATCAGTTGTTAGAATCAATAGGTCTTCAAATCGTTCATTTGAAAAAATTGAAACCCTTCTTATTGGATGATCATAATACTTCCCAAAAATCGAAATTCTTGATCAATGGAGGAAGAATATCACCATTTTTGTTCAATAAGATACCGAAGTGGATGATTGACTCATTCCATACTAGAAAAAATCGCAGGAAATCTTTTGATAACACGGATTCCTATTTCTCAATGATATCCCACGATCAAGACAATTGGCTGAATCCTGTGAAACCATTTCATAGAAGTTCATTGATATCTTCTTTTTATAAAGCAAATCGACTTCGATTCTTGAATAATCCACATCGCTTCCGCTTCTATTGTAACAAAAGATTCACTTTTTATGTGGAAAAGGTCCGTATCAATAATTATGATTTTACGTATGGACAATTCCTCAATATCTTGTTCATTCGCAACAAAATATTTTCTTTGTGCGGCGGTAAAAAAAAACATGCTTTTTTGGAGAGAGATACTATTTCACCAATCGAGTCACAGGTATCTAACATATTCATACCTAACGATTTTCCACAAAGCGGTGACGAAAGGTATAACTTGTACAAATTTTTCCCTTTTCCAATTCGATCCGATCTATTAGTTCGTAGAGCTATTTACTCGATCGCAGCCATTTCTGGAACACCTCTAACAGAGGGACAAATAGTCAATTTTGAAAGAACTTATTGTCAACCTCTTTCAGATATGAATCGATCTGATTCAGACGAGAAGAACTTGCATCAGTATCTCAATTTCAATTCAAACATGGGTTTGATTCACACTCCATGTTCTGAGAAATATTTACCATCCGAAAAGAGAAAGAGGAAAAAACGGAGTCTTTGTCTAAAGAAATGCGTTGAGAAAGGGCAGATGTCTAGAACCTTTCAACGAGACAGTGCTTTTTCAACTCTCTCAAAATGGAATCGATTCCAAACATATATGCCATGGTTCCTTACTTCGACAGGGTACAAATATCTAAATTTGATATTTTTAGATACTTTTTCAGACCTATTGCCGGTACTAAGTAGCAGTCAAAAATTTGTATCCATTTTTCATGATATTATGCATGGATCAGATAGAGCATGGCGAATTCTTCAGAAAAAATGGTGTCTTCCACAATGGAATCTGATAAGTGAGATTTCGAGTAAGTGTTTCCATAATCTTCTTCTGTCCGAAGAAATGATTCATCGAAATAATGAGTCACCATTGATATCGACACATCTGAGATCGCCAAATGCTCGGGAGTTCCTCTATTCAATCCTTTTCCTTCTTCTTGTTGCTGGATATCTCGTTCATACACATCTTCTCTTTGTTTCCCGAGCCTATAGTGAGTTAGAGACAGAGTTCGAAAGGGTCAAATCTTTGATGATTCCACCATACATGATTGAGTTGCGAAAACTTCTGGATAGGTATCCCACATCTGAACTGAATTCTTTCTGGTTAAAGAATCTCTTTCTAGTTGCTCTGGAACAATTAGGAGATTCTCTAGAAGAAATACGGGGTTCTGCTTTTGGCGGCAACATGCTATGGGGTGGTGGTCCCGCGGATGGGGTTAAATCAATACGTTCTAAGACGAAAGATTTGAATATCAATCTCGTCGATATCACCGATCTCATAAGTATCATACCAAATCCCATCAACCGAATCACTTTTTCGAGAAATACGAGACATCTAAGTCATACAAGTAAAGAGATCTATTCATTGATAAGAAAAAGAAAAAAGGGGAACGGTGATTGGATTGATGATAAAATAGAATCCTGGGTCGCGAACAGTGATTCGATTGATGATAAAGAAAGAGAATTCTTGGTTCAGTTCTCCACCTTAACCTTAACGACAGAAAAAAGGATTGATCAAATTCTATCGAGTCTGACTCATAGTGATCATTTATCAAAGAATGACTCTGGTTATCAAATGATTGAACAACCGGGAACAATTTACTTACGATACTTAGTTGACATTCATAAAAAGCATTTCATGAATTATGAGTTCAATACATACTGTTTAGCAGAAAGACGGATATTCCTTGCTCATTATCAGACAATCACTTATTCACAAACTTCGTGTGGGGCTAATAGTTTTCATTTCCCATCTCATGGAAAACCCTTTTCGCTCCGCCTAGCCCTATCCCCCTCTAGGGGTATTTTAGTGATAGGTTCTATAGGAACCGGACGCTCCTATTTGGTCAAATACCTAGCGGCAAACTCCTATGTTCCTTTCATTACAGTATTTCTGAACAAGTTCCTGGATAACAAGCCTAAAGGTTTTCTTATTGATGATATCGATGATGATAGTGACGATATTGATGCTAGTGACGATATTGATGCTAGTGACGATATCGATCGTGACCTTGATACGGAGCTGGAGCTTCTAACTATGATGAATGCGCTAACTATCGATATGATGTCGGAAATAGGCCGATTTTATATCACCCTTCAATTCGAATTAGCAAAAGCAATGTCTCCTTGCATAATATGGATTCCAAACATTCATGATCTGGATGTGAATGAGTCGAATTACTTATCCCTCGGTCTATTAGTGAACTATCTCTCCAGGGATTGTGAAAGATGTTCCACTAGAAATATTCTTGTTATTGCTTCGACTCATATTCCCCAAAAAGTGGATCCCGCTCTAATAGCCCCGAATAAATTAAATACATGCATTAAGATACGAAGGCTTCTTATTCCACAACAACGAAAGCACTTTTTCACTCTTTCATATACTAGGGGATTTCACTTGGAAAAGAAAATGTTCCATACTAATGGATTCGGGTCCATAACCATGGGTTCCAATGCACGAGATCTTGTAGCACTTACCAATGAGGCCCTATCGATTAGTATTACACAGAAGAAATCAATTATAGACACTAATACAATTAGATCTGCTCTTCATAGACAAACTTGGGATTTGCGATCCCAGGTAAGATCGGTTCAAGATCATGGGATCCTTTTCTATCAGATAGGGAGGGCTGTTGCACAAAATGTACTTCTAAGTAATTGCCCCATAGATCCTATATCTATCTATATGAAGAAGAAATCGTGTAACGAAGGGGATTCTTATTTGTACAAATGGTACTTCGAACTTGGAACGAGCATGAAGAAATTAACGATACTTCTTTATCTTTTGAGTTGTTCTGCCGGATCGGTCGCTCAAGACCTTTGGTCTCTACCCGGACCCGATGAAAAAAACGGGATCACTTCTTCTGGACTCGTTGAGAATGATTCTGATCTAGTCCATGGCCTATTAGAAGTAGAAGGCGCTCTGGTGGGATCCTCGCGGACAGAAAAAGATTGCAGTCGGTTTGATAATGATCGAGTGACATTGCTTCTTCGGCCCGAACCAAGGAATCCCTTAGATATGATGCAAAAAGGATCTTGTTCTATCGTTGATCAGAGATTTCTCTATGAAAAATACGAATCGGAGTTTGAAGAAGGGGAAGGAGAAGGAGTCCTCGACCCGCAACAGATAGAGGAGGATTTATTCAATCACATAGTCTGGGCTCCTAGAATATGGCGCCCCTGGGGCTTTCTATTTGATTGTATCGAAAGGCCCAATGAATTGGGATTTCCCTATCGGGCCGGGTCATTTCGGGGCAAGCGGATCATTTATGATGAAAAGGATGAGCTTCAAGAGAATGATTCGGAGTTCTTGCAGAGTAGAACCATGCAGTACCAGACACGAGATAGATCTTCCAACGAACAAGGCTTTTTTCGAATAAGCCAATTCATTTGGGAACCTGCGGATCCACTCTTTTTCCTATTCAAAGATCAGCCCCTTGTCTCTGTGTTTTCACACCGAGAATTCTTTGCAGATGAAGAGATGTCAAAGGGGCTTCTTACTTCCCAACCAGATCCTCCTACATCTATATATAAACGCTGGTTTATCAAGAATACGCAAGAAAAGCACTTCGAATTGTTGATTCATCGCCAGAGATGGCTTAGAACCAATAGTTCATTATCTAATGGATTTTTCCGTTCTAATACTCCATCCGAGAGTTATCAGTATTTATCAAATCTGTTCCTATCTAACGGAACGCTATTGGATCAAATGACAAAGACATTGTTGAGAAAAAGATGGCTTTTTCCGGATGAAATGAAAATTGGATTCATGTAA

>rpl23

ATGGATGGAATCAAATATGCAGTATTTACAGACAAAAGTATTCGGTTATTGGGAAAAAATCAATATACTTCTAATGTCGAATCAGGATCAACTAGGACAGAAATAAAGCATTGGGTCGAACTCTTCTTTGGTGTCAAGGTAATAGCTATGAATAGTCATCAACTCCCCCGAAAGGGTAGAAGAATGGGACCTATTATGGCACATACAATGCATTACAGACGTATGATCATTACGCTTCAACCGGGTTATTCTATTCCACCTCTTAGAAAGAAAAGAACTTAA

>rpl2

ATGGCGATACATTTATACAAAACTTCTACCCCGAGCACACGCAATGGAGCCGTAGACAGTCAAGTGAAATCCAATCCACGAAATAATTTGATCTATGGACAGCATCGTTGTGGTAAAGGTCGTAATGCCAGAGGAATCATTACCGCAGGGCATAGAGGGGGAGGTCATAAGCGTCTATACCGTAAAATCGATTTTCGACGGAATGAAAAAGACATATATGGTAGAATCGTAACCATAGAATACGACCCTAATCGAAATGCATACATTTGTCTCATACACTATGGGGATGGTGAGAAGAGATATATTTTACATCCCAGAGGGGCTATAATTGGAGATACCATTGTTTCTGGTACAGAAGTTCCTATAAAAATGGGAAATGCCCTACCTTTGACCGATATGCCCTTAGGCACGGCCATACATAACATAGAAATCACACTTGGAAAGGGTGGACAATTAGCTAGAGCAGCGGGTGCTGTAGCGAAACTGATTGCAAAAGAGGGGAAATCGGCCACATTAAAATTACCTTCTGGGGAGGTCCGTTTGATATCCAAAAACTGCTCAGCAACAGTCGGACAAGTGGGGAATGTTGGGGCGAACCAGAAAAGTTTGGGTAGAGCCGGATCTAAATGTTGGCTAGGGAAGCGTCCTGTAGTAAGAGGAGTAGTTATGAACCCTGTAGACCACCCCCATGGGGGTGGTGAAGGAAGGGCTCCGATTGGTAGAAAAAGACCCGCAACCCCTTGGGGTTATCCTGCACTTGGAAGACGAAGTAGAAAAAGGAATAAATATAGTGATAATTTGATTCTTCGCCGCCGTACTAAATAG

***P. amurense***

>psbA

ATGACTGCAATTTTAGAGAGACGCGAAAGCGAACGCCTATGGGGTCGCTTCTGTAACTGGATAACCAGCACCGAAAACCGCCTTTACATTGGATGGTTTGGTGTTTTGATGATCCCTACTTTATTGACTGCAACTTCTGTATTTATTATCGCCTTCATTGCTGCTCCTCCAGTAGATATTGATGGTATTCGTGAACCTGTTTCTGGATCTCTACTTTATGGAAACAATATTATTTCTGGTGCGATTATTCCTACTTCTGCAGCTATAGGTTTGCACTTTTACCCGATATGGGAAGCGGCATCTGTTGATGAATGGTTATACAATGGTGGTCCTTATGAGCTAATTGTTCTACACTTCTTACTTGGTGTAGCTTGTTACATGGGTCGTGAGTGGGAACTTAGTTTCCGTCTGGGTATGCGCCCTTGGATTGCTGTTGCATATTCAGCTCCTGTTGCAGCAGCGGCTGCTGTTTTCTTGATCTACCCAATCGGTCAAGGAAGTTTTTCTGATGGTATGCCTCTAGGAATCTCTGGTACTTTCAACTTCATGATTGTATTCCAGGCCGAGCACAACATTCTTATGCACCCATTTCACATGTTAGGCGTAGCTGGTGTATTCGGCGGCTCCCTATTCAGTGCTATGCATGGTTCCTTGGTAACCTCTAGTTTGATCAGGGAAACTACAGAAAATGAATCTGCTAATGAAGGTTACAGATTCGGTCAAGAGGAAGAAACTTATAATATCGTAGCTGCTCACGGTTATTTTGGCCGATTGATCTTCCAATATGCTAGTTTCAACAATTCTCGTTCTTTACATTTCTTCCTAGCTGCTTGGCCTGTAGTAGGTATCTGGTTTACCGCTTTAGGTATTAGCACTATGGCTTTCAACCTAAATGGTTTCAACTTCAACCAATCCGTAGTTGATAGTCAAGGTCGTGTAATTAATACCTGGGCTGATATTATTAATCGTGCTAACCTTGGTATGGAAGTTATGCATGAACGTAATGCTCATAACTTCCCTCTAGACCTAGCTGCTATTGAAGCTCCATCTACAAATGGATAA

>matK

ATGGAGGAATTTCAAGTATATTTAGAACTAGATAGATCTCAACAACACGACTTCCTATACCCACTTCTTTTTCGGGAGTATATTTATGCACTTGCTCATGATCTTGGTTTAAATAGCTCGATGATTTCATTGGAAAGTGGGGGTTATGACAATAAATCTAGTTCACTAAGTGTGAAACGGTTAATTACTCGAATGTATCAACAGATTCATTTGAGTATTGCTGCTAATGATTCTAACCAAAATCCAATTTTTGGGCACAACAAGAAGTTGTATTCTCAAATTATATCAGAGGGATTTGCTGTCGTGGTGGAAATTCAATTTTCCCCATGCTTGGTAGCTTTTTTAGAAGGTAAAGAAAATGAAAAATCTCCAAATTTCCAATCAATTCATTCAATATTTCCTTTTTTCGAGGACAAACTGTCACGTTTAAATTATGTGTTAGATGTACTAATACCCCACCCCATTTGTCCCGAAATCTTGGTTCAACGCCTTCGCTACTGGATAAAGGATGCCTCCTCTTTACATTTATTACGGTTCTTTCTCCACGAGTATTTTAATTCGAATAGTCTTATTACTCCAAAGAACTCTATTTCTGTTTTTTTAAAAAGGAATCCAAGATTGTTATTGTTTCTATATAATTCTCATGTATATGAATATGAATCCATCCTCTTTTTTCTCGGTAACCAATCGTCTCATTTACGATCAACATCCTCTCGAGTCCTCGTTGAGCGAATGTATTTCTATGGAAAAGTCGAACATCTTGTCGAAGTCTTTGCTAAAGATTTTCAGGACATCCTATGGTTGTTCAAGGATCCTTTCATGCATTATGTTAGATATCAAGGAAAATCCATTCTGGCTTCAAAGGATACGCCTCTTCTGATGAATAAATGGAAATATTACCTTGTTGGTTTATGGCAATGGCATTTTCACGTGTCGTCTCAACCAGGAAGGGTTCATCTAAACCACTTAGGCAAGTACTCTATCAACTTTCTGGGCTATCTTTCCGGTGTGCGACTCAATTCTTTGGTGGTACGGAGTCAAATGCTAGAAAATTCATTTCTAATAGGTAATTCTATGAAGAAGGTCGATACGACCGTTCCAATTATTCATCTGATTGGATCATTGATGAAGGCGCGGTTTTGTAACGCATTGGGGCATCCCATCAGTAAGGCGACCTGGGCCGATTTCTCCGATTCTCATCTTATCGACCGATTTGTGCGTATATGCAGAAATCTTTCTCATTATCACAGCGGATCCGCAAAAAAAAGTTTGTATCGAATAAAATATATACTTCGGCTTTCTTGTGTTAAAAGCTTGGTTCGTAAACATAAAAGTACTGTACGCGTTTTTTTGAAAAGATTAGGTTCGGAATTATTGGAAGAATTCCTTACGGAGGAAGAACACGTTCTTTCTTTAATCTTTCCAAGAGCTTCGTCTACTTCGCGTAGGTTTTATTTATATAGAGGGCGGATTTGGTATTTGGATATTATTTGTATCAACGATCTGGTTAATTATGAATGA

>rps16

ATGGTAAAACTTCGTTTGAAACGATGTGGTAGAAAGCAACGTGCCGTTTATCGAATCGTTGCAATTGATGGTCGATCCCGAAGAGAAGGAAGAGATCTTCAGAAAGTGGGTTTTTATGATCCGATAAATAATCAAACCCATTTAAATGTTCCTGCTATTCTATATTTCCTTGACAAGGGCGCCCAACCTACAGGAACCGTTCATGATATTTCAAAGAAAGCGGGGGTTTTTACAGAACTTAGTCTTAATCAAACGAAATTCTATTAA

>psbK

ATGCTTAATATCTTTAGTTTAATGTATATCTGTCTTAATTCTGCCCTTTATTCGAGTAGTTTTTTATTCGCCAAATTGCCCGAGGCCTACGCTTTTTTGAATCCAATTGTAGATGTTATGCCAGTAATACCTCTTCTATTTTTTCTCTTAGCCTTTGTTTGGCAAGCTGCTGTAAGTTTTCGATGA

>psbI

ATGCTTACTCTCAAACTCTTTGTTTACACCGTAGTGATATTCTTTGTTTCTCTCTTCATCTTCGGATTCCTGTCTAATGATCCAGGGCGTAATCCCGGACGCGAAGAATAA

>atpA

ATGACAACAATTAAAGCCGACGAAATTAGTAATATTATCCGCGAACGTATTGAGCAATATAATAGAGAAGTAAAGATTGTAAATATTGGTACCGTACTTCAAGTAGGCGACGGCATCGCCCGTATTTATGGTCTTGATGAAGTAATGGCAGGGGAATTAGTAGAATTTGAAGAGGGTACAATAGGCATTGCTCTTAATTTGGAATCAAATAATGTTGGTGTTGTTTTAATGGGTGACGGTTTAATGATACAAGAGGGAAGTTCTGTAAAAGCAACCGGCAAAATTGCTCAGATACCAGTAAGCGAGGCTTATTTAGGTCGTGTTATAAATGCCCTGGCTAAACCTATTGATGGTCGAGGTGAAATTTCAGCTTCTGAATCTCGATTAATCGAATCTCCCGCTCCGGGTATTATTTCGAGACGTTCCGTATATGAGCCTCTTCAAACAGGACTTATTGCTATTGATTCGATGATCCCTATAGGACGCGGGCAGCGAGAATTAATTATTGGGGACCGACAGACCGGTAAAACTGCAGTAGCCACGGATACGATTCTCAACCAACAAGGGCAAAATGTAATATGCGTTTATGTAGCTATTGGGCAAAAAGCATCTTCTGTGGCTCAGGTAGTGAATACTTTCCAGGAAAGGGGGGCAATGGAATACACTATTGTGGTAGCCGAAACGGCGGATTCCCCCGCTACGTTACAATACCTCGCTCCTTATACGGGCGCAGCTCTGGCTGAATATTTTATGTACCGTGAACGACACACTTTAATCATTTATGATGATCCCTCCAAACAAGCGCAGGCTTATCGACAAATGTCTCTTCTATTACGAAGACCACCCGGTCGCGAAGCTTATCCCGGAGATGTTTTTTATTTGCATTCACGGCTTTTGGAAAGAGCCGCGAAATTAGGTTCGCAGTTAGGTGAAGGAAGTATGACCGCTTTACCAATAGTTGAGACCCAGTCAGGAGATGTTTCGGCTTATATTCCTACTAATGTAATTTCCATTACAGATGGGCAAATATTCTTATCCGCCGATCTATTCAATGCTGGAATCAGACCCGCTATTAACGTGGGTATTTCTGTCTCTAGAGTAGGATCCGCAGCTCAAATTAAAGCTATGAAACAAGTAGCCGGCAAATTAAAATTGGAATTGGCCCAATTCGCGGAATTAGAAGCCTTTGCACAATTTGCTTCTGATCTCGATAAAGCTACTCAGAACCAATTGGCAAGGGGTCAACGCTTACGTGAGTTGCTGAAACAATCCCAATCAGCCCCTCTCACGGTCGAAGAACAGATAATGACTATTTATACAGGAACGAATGGTTATCTTGATTCATTAGAAATTGGCCAAGTAAGAAAATTTCTCGTTGAGTTACGTACTTACTTAAAAACGAATAAACCTCAGTTCCAAGAAATAATATCTTCTACCAAGATATTCACCGAGGAAGCAGAAGCCCTTTTGCAAGAAGCTATTCAGGAACAAATGGAACGCTTTCTACTTCAGGAACAATTATAA

>atpF

ATGAAAAATGTAACCGATTCTTTCGTTTCTTTGGTTCACTGGCCATTCGCCGGGAGTTTCGGGTTTAATACCGATATTTTAGCAACAAATCCAATAAATCTAAGTGTAGTGCTTGGTGTATTGATCTTTTTTGGAAAGGGAGTGTTAAGTGATTTATTAGATAATCGCAAACTGAGGATCTTGAATAGTATTCGAAATTCAGAAGAACTGCAGGGCGGGGCCGTTGAACGGCTGGAAAAAGCCCGGGCCCGGTTACGGAAAGTCGAAATAGAAGCAGATCAGTTTCGAGTGAACGGATACTCTGAGATAGAACGAGAAAAATTAAATTTGATTAATTCAACTTCTAAGACTTTGGGCCAATTAGAAAATTACAAAAATGAAACCATTCATTTTGAACAACAAAGAGCAATTAATCAAGTCCGACAACGGGTTTTCCAACAAGCTTTACAAGGAGCGCTCGGAACTCTGAATAGTTGTTTGAACAAGGAGTTACATTTACGTACCATTAGTGCCAATATTGGCATGTTTGGGGCGATGAACGAAATAACTGATTAG

>atpH

ATGAATCCACTTATTTCTGCCGCTTCCGTTATTGCCGCTGGGTTGGCTGTTGGGCTTGCTTCTATTGGACCTGGAGTTGGTCAAGGTACTGCTGCGGGCCAAGCAGTAGAGGGGATTGCGAGACAACCCGAGGCGGAGGGAAAAATACGAGGTACTTTATTGCTTAGTCTGGCTTTTATGGAAGCTTTAACAATTTATGGACTGGTTGTAGCATTAGCGCTTTTATTTGCGAATCCTTTTGTTTAA

>atpI

ATGAATGTTCTATCATGTTCCATCAACACACTAAAGGGGTTATACGATATGTCCGGTGTGGAAGTAGGCCAACATTTCTATTGGCAAATAGGCGGGTTCCAAGTCCATGCCCAAGTACTTATTACTTCTTGGGTTGTAATTGCTATCTTATTAGGTTCAGCCTTTATAGCCGTTCGGAATCCACAAACCGTTCCGACTGCCAGTCAAAATTTCTTCGAATATGTCCTTGAATTCATTCGAGACGTGAGCAAAACGCAGATTGGAGAAGAATATGGCCCATGGGTTCCCTTTATTGGAACTATGTTTCTTTTTATTTTTGTTTCGAATTGGTCAGGTGCTCTTTTACCTTGGAAAATCATAGAGTTACCTCATGGGGAGTTAGCCGCACCCACGAATGATATAAATACTACCGTTGCTTTAGCTTTGCTCACGTCAGTAGCATACTTCTATGCGGGTCTTTCCAAAAAGGGATTAGGTTATTTCAGTAAATACATTCAACCGACTCCAATTCTGTTACCCATTAACATTTTAGAAGATTTCACAAAACCCTTATCGCTTAGTTTTCGACTTTTCGGGAATATATTAGCCGATGAATTAGTAGTTGTTGTTCTTGTTTCTTTAGTCCCTTTAGTGGTTCCTATACCTGTCATGTTCCTTGGATTATTTACAAGCGGTATTCAAGCTCTTATTTTTGCAACTTTAGCTGCGGCTTATATAGGCGAATCTATGGAGGGACATCATTGA

>rps2

ATGGCAAGAAGATATTGGAACATCCATTTGGAAGAGATGATGGAAGCAGGAATTCATTTTGGTCATGGTACTCGGAAATGGAATCCTAGAATGGCACCTTATATATCTGCAAAACACAAAGGTATTCATATTACAAATCTGACTCGAACTGCTCGTTTTTTATCAGAAGCTTGTGATTTAGTTTTTGATGCAGCAAGTAGGGGAAAACAATTCTTAATTGTTGGTACTAAAAATAAAGCAGCTGATTCAGTCGCGCGAGCTGCAATAAGGGCTCGGTTTCATTATGTTAATAAAAAATGGCTCGGTGGTATGTTAACGAATTGGTCCACTACAGAAACGAGACTTCACAAGTTCAGGGATTTGAGAACGGAACAAAAAAAGGGGAGACTCGACAGTCTTCCCAAAAGGGATGCCGCTATTTTGAAGAGACAATTATCGCGCCTGCAAACGCATCTGGGCGGGATTAAATATATGACGAGGGTACCCGATATTGTAATCATCGTTGATCAGCACGAAGAATATACGGCTCTTCGAGAATGTATCACTTTGGGAATTCCAACAATTTGTTTAATCGATACAAATTGTGACCCTGATCTCGCAGATATTTCGATTCCAGCAAACGATGACGCTATAGCTTCAATCCGATTAATTCTTAACAAATTAGTATTCGCAATTTGTGAGGGTCGCTCTAGCTATATACGAAATCGTTGA

>rpoC2

ATGGCAGAACGGGCGAGTCTGGTCTTTCACAATAAAACGATAGATGGAACTGCCATTAAACGACTTATTAGCAGGTTAATAGATCACTTCGGAATGGCATATACATCACACATCCTGGATCAAGTAAAGACCCTGGGTTTCCAGCAAGCCACTGCTACATCTATTTCATTAGGCATTGATGATCTTTTAACGATACCTTCTAAGCGATGGCTAGTCCAAGATGCTGAACAACAAAGTTTTATTTTGGAAAAACACCACCATTATGGGAATGTACACGCGATAGAAAAACTACGTCAATCCATTGAGATATGGTATGCTACAAGTGAATATTTGCGACAAGAAATGAATCCTAATTTTAGGATGACTGATCCCTTTAATCCAGTCCATATAATGTCTTTTTCGGGAGCTAGAGGAAATGCATCTCAAGTACACCAATTGGTGGGTATGAGAGGATTAATGTCTGATCCCCAAGGTCAAATGATTGATTTACCCATTCAAAGCAATTTACGCGAAGGACTTTCTTTAACAGAATATATCATTTCTTGCTATGGAGCCCGTAAGGGAGTTGTAGATACCGCTGTACGAACATCAGATGCTGGATATCTTACGCGCAGACTTGTTGAAGTAGTTCAACACATTGTTGTACGTAGAACAGATTGTGGCACCATCCGAGAAATTTCTGTGAGTCCTCAAAATCAAAATAAGATGCTGTCGGAAAGGGTTTTTAGCCAAACATTAATTGGTCGTGTATTAGCAGACGATATATATATGGGTCCGCGATGCATCGCCATTAGAAATCAAGATATTGGGATTGGACTTGTCAATCGACTCATAACCTTTCGAACACAAGCAATATCTATTCGAACCCCCTTTACTTGTAGGAGTACATCTTGGATCTGTCGATTATGCTATGGTCGGAGTCCGACTCATGGTGACCTGGTTGAATTGGGGGAAGCCGTAGGTATTATTTCGGGTCAATCTATTGGGGAACCGGGGACTCAACTAACATTAAGAACTTTTCATACCGGTGGCGTATTTACAGGGGGCACTGCAGAACATGTACGAGCCCCTTCTAATGGTAAAATAAAATTCAACGAGGATTTGGTTCATCCCACGCGTACACGTCACGGGCATCCTGCTTTTCTATGTTCGATAGATTTGGATGTAATTATTGAGAGTGAAGATATTATGCATAATGTGACTATTCCACCAAAAAGTTTTCTTTTAGTTCAAAATGATCAATATGTCGAATCAGAACAAGTGATTGCTGAGATTCAGGCGGGAGCATACACTTTGAATTTTAAAGAGAGGGTTCGAAAACATATCTATTCTGATTCAGAGGGAGAAATGCACTGGAGTACTGATGTGTACCATGCACCCGAATTTACATATAGCAATGTACACCTCTTGCCAAAAACAAGTCATTTATGGATATTATCGGGGGGTTCATGCAGATCTAGTGTAGTTTCTTTTTCACTCTACAAGGATCAAGATCAAATGAATATTCATTCTCTTTCTGTCGAACGAAGAGAGATTTCTAGCCTCTCGCTCTCGGTGAATAATGATCAAGCGAGACACAAATTATTTAGTTCTGCTTTTTCTGCTAAAAAAGAAGGTGGAATTCTTGAGATGTCTGATTATTCGGGATTTAATAGAATCATAGGTACTGGTCATTGTAATCTCATACATCCTGCAATTCTCCGCGCGAATTCGAATTTATTGGCAAAAAGGCAAAGAAATCGATTTCTTATTCCATTCCACTCGATTCAAGAACAAGAGAAAGAGCTAATGCCCCATTCAGGAATCTCGATTGAAATACCCATAGGGGGTATTTTCCGTAGAAATAGTATTCTTGCTTATTTCGACGATCCTCGATACAGAAGAAAGAGTTCCGGAATTACTAAATATGGGACTCTGGGGGCGCATTCAATCGTCAAAAAAGAGGACTTGATTGAGTATCGAGGACTCAAAAAAATTAAGCCAAAATACCAAATTAAAATAGATCGCCTTTTTTTCATTCCCGAGGAAGTGCATTTTTTTCCCGAATCTTCTTACCTAATGGTACGGAATAATAGCATCATTGGAGTAGATACACGAATCACTTTAAATATAAGAAGCCGAGTGGGCGGATTGGTCCGAATAGAGAGAAAAAAAGGGGGGATTGAACTAAAAATATTTTCGGGAGATATCCATTTTCCCGGAGAGATAGATAAGATATCCCGACACAGTGGCATCTTGATACCGCCAGAAAGGGAAAAAAAAAAACTTAAGGAAGCCACTAAGGAATCAAAAAAATTGAAAAAATGGATCTATGTTCAACGGATCACACCTACCAAGAAAAAGTATTTTGTTTTGGTTCGACCCGTAGTCACATATGAAATAGCGGACGGTATAAATTTAGCAACACTCTTCCCCCAGGATCCGCTGCGGGAAAAGGATAATATGCAATTTCGAGTTGTCAATTATGTCCTTTATGGGAAGGGCAAAGCTGCTCGGGGAATTCCTGATACAAGTATTCAATTAGTTCGGACGTGTTTAGTGTTGAATTGGGACCAAGACAAAAAAAGTTCTTCCGTCGAAGAGGTTTGTGCTTCCTTTGTTGAAGTACGTACAAATGGTCTGATTCGCGATTTCTTAAGACTCAACTTAGTGAAATCCCAAATTTCGTATATCAGAAAAAGGAATCATCCGTCAGGTTCAGGATTGATCTCTGATAATGGTTCCGTTCGCACCAATAGCAATCCGTTTTATTCCGTTTTTGGCAAGGCAGGGGTTGAACAATCACTTAGCCAAAATCAAGGAACTATTCGTACGTTGTTGAATAGAAATAAGGAATGCCAATCTTTGATAATTTTGTCATCATCTAATTATTTTAGAATGGGTCCATTGACCGATGTAAAATATCACAATGTGATAAAACAATCAATTCCAATTCAAAAAGGTTCTCTAACCCCAATTAGGAATTCGTTGGGACCCTTAGGAACAGCCCTTCAAATTGAGAATTTTTATTCATTTTACTATTTAATAACTCTAATAACTCATAATCATCTCTCGGTAACTAAATATTTGAAACTTGACAATTTAAAACAGCCTTGTCAAGTACTTAAATATTATTTAATGGATGAAAACGGGGAAATTTCTAATCCTGATACAGACAGTAAGATCATTTTGAATCCATTTAATTTGAATTGGTATTTTCTCCATCATAATTATTGTGAGGAAATGTCCCCGATAATAAGTCTTGGGCAGTTTCTTTGTGAAAATGTATGTATAACCAAAAACGGACCACACCTAAAATCTGGTCAAGTTTTAATTGTTCAAGTTAACTCTGTAGTAATACGATCAGCTAAGCCTTATTTGGCTACTCCTGGAGCAACTGTTCATGGGCATTATGGAGAAATCCTTTACGAAGGGGATACATTAGTTACATTTATATATGAAAAATCGAGATCTGGTGATATAACGCAGGGCCTTCCAAAAGTAGAACAAGTGTTAGAAGTACGTTCGCTTGATTCAATATCGATGAACCTAGAAAAGAGAGTTGAGGGTTGGAACGCGCGTATAACAAGAATTCTTGGGATTCCCTGGGGATTCTTGATTGGTGCTGAGCTAACTATAGTGCAAAGTCGTATCTCTTTGGTTAATAAGATCCAAAAGGTTTATCGATCGCAGGGGGTGCAGATCCATAATAGGCATATAGAAATTATTGTACGTCAAATAACATCAAAAGTCTTGGTTTCAGAAGCTGGAATGTCTAATATTTTTTTACCCGGCGAACTTATTGGATTGTTACGAGCGGAACGAACGGGGCGCGCTTTGGAAGAAGTGATCTGTTATCGAGCTATCTTATTGGGAATAACGAGAGCATCTCTGAATACTCAAAGTTTTATATCCGAAGCAAGTTTTCAAGAAACCACGCGAGTTTTAGCAAAAGCAGCTCTCCGAGGTCGTATCGATTGGTTGAAAGGCCTGAAGGAAAACGTTGTTCTGGGGGGGATAATACCCGTTGGTACCGGATTCAAAGGATTAGTGCACTGTTCAAGGCAGCATAACACCATTCTTTTGGAAAGACAAAAAGGGAATTTATTCGGGGGGGAAATGAGAGATATTTTCTTACACCACAGAGAATTATTTGACTCTTGCATTTCAACGACTTTCCATGATACATCAGAGCAATTGCTTAGAGGGTTTAATGAGTCCTAG

>rpoC1

ATGATTGATCGATATAAACATCAACAACTCCGAATTGGATCAGTTTCTCCTCAACAAATAAGCGCTTGGGCCAATAAAATCCTACCTAATGGAGAGATTGTTGGAGAAGTGACAAAACCCTATACTTTTCATTACAAAACCAATAAGCCGGAAAAAGATGGATTATTTTGTGAAAGAATTTTTGGGCCTATTAAAAGCGGAATTTGCGCTTGTGGAAATTATCGAATAATCGGAGATGAAAAGGAAGACCCGCAATTTTGTGAACAATGTGGAGTTGAATTTGTTGATTCTCGGATACGAAGATATCAAATGGGATACATAAAACTAGGCTGCCCAGTAACCCACGTGTGGTATTTGAAACGTCTTCCTAGTTATATCGCGAATCTTTTAGATAAACCTCTTAAAGAATTAGAAGGCCTAGTATTTTCTTTTGCTAGGCCTATAGCGAAAAAACCGACTTTTTTACGATTACGAGGTTCATTCGAATATGAAATACAATCCTGGAAATACAGCATCCCGCTTTTTTTTACTACCCAAGGTTTCGATAAATTTCGTAACCGAGAAATTTCTACTGGAGCAGTTGCTATCCGGGAACAATTAGCCGATCTAGATTTGCGAATTATTCTAGATTATTCCTTGTTAGAATGGAAAGAATTAGGGGAAGAAGGACCCGCGGGTAATGAATGGGAAGATCTAAAAGTTGGACGAAGAAGGGATTTTTTGGTTAGACGCATGGAATTAGCTAAGCATTTTCTTCGAACAAATATAGAACCTGAGTGGATGGTTTTATGTCTATTACCTGTTCTTCCTCCCGAGCTGAGACCGATCATTCAGATAGATGGAGGTAAACTAATGAGTTCAGATATTAATGAACTCTATAGAAGAGTTATCTATCGGAACAATACTCTTACCGATCTATTAACAACAAGTAGATCTACGCCAGGGGAATTAGTAATGTGTCAGGAGAAATTGGTACAAGAAGCCGTGGATACGCTTCTTGATAATGGAATCCGTGGCCAGCCAATGAGGGATGGTCATAATAAGATTTATAAGTCGTTTTCAGATGTAATTGAAGGCAAAGAGGGAAGATTTCGTGAGACTCTGCTTGGCAAACGGGTTGATTATTCGGGGCGTTCTGTCATTGTTGTAGGCCCCTCACTTTCGTTACATCGATGTGGATTGCCTCGCGAAATCGCAATAGAGCTTTTCCAGAGTTTTGTAATTTGTGGGCTAATTAGACAACATCTTGCTTCGAACATAGGAGTTGCTAAGAGTAAAATTCGGGAAAAAGGGCCAATTGTATGGGAAATACTGCAGGAAGTTATGCAGGGACATCCAGTATTGCTGAATAGAGCGCCTACTCTGCATAGATTGGGCATACAGGCATTCCAGCCCATTTTAGTGGAAGGGCGCGCTATTTGTTTACATCCATTAGTTTGTAAGGGATTCAATGCAGACTTTGATGGGGATCAAATGGCTGTTCATGTACCTTTATCTTTAGAGGCTCAAGCGGAGGCTCGTTTACTTATGTTTTCTCATATGAATCTCTTGTCTCCTACTATTGGAGATCCCATTTCCGTACCGACTCAAGATATGCTTATTGGACTCTATGTATTAACGAGCGGGAATCGTCGAGGTATTTGTGCAAATAGGTATCATCCATGTAATCGAAGAAATTATCAAGATGAAAGAATTGACGATAATAGCTATAACTATAAGTATACGAAGGAACCCTTTTTTTGTAATTCCTATGATGCAATTGGGGCTTATCGGCAGAAAAGAATCAATTTAGATAGTCCTTTGTGGCTCCGGTGGCGATTAGATCAACGCGTTATTGCTTCAAGGGAAGCTCCCATCGAAGTTCACTATGAATCTTTGGGTACCTATCATGAGATTTATGGACATTATCTAATAGTACGAAGTGTAAAAAAAGAAATTCTTTCTATATACATTCGAACCACCGTTGGCCATATTTCTTTTTATCGAGAAATCGAAGAAGCTATACAAGGGTTTTGTCTGGCCTGCTCATATGGTACCTAA

>rpoB

ATGCTCGGAGATGGAAATGCGGGAATGTCTACAATACCTGGGTTGAATCAGATACAATTTGAAGGCTTTTGTAGGTTCATTGATCAGGGCTTAACAGAAGAACTTTATAAGTTTCCAAAAATTGAAGATACGGATCAAGAAATTGAATTTCAATTATTTGTGGAAACATATCAATTGGTAGAACCCTTGCTAAAAGAAAGAGATGCTGTATATGAATCATTCACGTATTCTTCTGAATTATATGTATCAGCAGGATTAATTTGGAAAAGCCGAGGGGATATGCAGGAACAAACAATTTTTATTGGAAACATTCCTCTAATGAATTCTTTGGGAACTTCTATAGTAAATGGAATATACAGAATTGTCATCAATCAAATATTGCAAAGTCCCGGTATCTATTATCGGTCAGAATTGGGCCATAATGGAATGTCGGTCTATACAGGCACCATAATATCCGATTGGGGAGGAAGATTCGAATTAGAGATTGATAGAAAAGCAAGGATATGGGCTCGTGTGAGTAGGAAACAGAAAGTCTCTATTCTAGTTCTATCAGCAGCTATGGGTTCGAATCTACGAGAAATTCTAGAGAATATTTGCTACCCTGAAATTTTCTTGTCTTTCCTGACCAATAAGGAGAAAAAAAAAATTGGATCAAAAGAAAATGCCATTTTGGAGTTTTATCAACAATTTGCTTGTGTAGGCGGAGATCCGGTATTTTCGGAATCCTTATGTAAGGAATTACAAAAGAAATTTTTTCACCAAAGATGTGAATTAGGAAAGATTGGTAGACGAAATATGAACCAGAGACTGAATCTTAATATACCTCCGAACAATACCTTTTTGTTACCACAAGATGTATTGGCAGCTGTCGATCATTTGATTGGACTGAAATTTGGAATGGGTACACTTGACGATATGAATCATTTGAAAAATAAGCGTATTCGGTCTGTAGCGAATCTTTTACAAGATCAATTCGGATTGGCCCTGGTTCGGTTAGAAAATGTGATTAGAGGAACTATATGCGGGGCAATTAGGCATAAATTGATGCCAACTCCTCAAAATTTGGTAACTTCAACTCCATTAACAACCACTTATGATTCTTTTTTCGGATTACATCCATTATCTCAAGTTTTGGATCGAACTAATCCATTGACACAAATAGTTCATGGGATAAAATTGAGTTATTTGGGCCCCGGAGGATTGACAGGACGGACTGCTAGTTTTCGGATACGAGATATCCATCCTAGTCACTATGGCCGCATTTGCCCAATTGACACGTCTGAAGGAATCAATGTTGGACTTATTGGATCCTTAGCAATTCATGCGAGAATTGGTTATTGGGGGTCTCTAGAAAGCCCATTTTATGAAATCTTTGAAAAATCAAAAAAAGTACGGATGCTTTATTTATCACCAAGTAGAGATGAATACTATATGGTAGCGGCAGGAAATTCTTTGGCACTGAATCAGGGTAGTCAGGAAGAACAGGTTGTTCCGACTCGATACCGTCAAGAATTCCTGACTATTGCGTGGGAACAGGTTCATCTTCGAAGTATTTTTCCCTCCCAATATTTTTCTATTGGGGCTTCCCTCATTCCTTTTATCGAGCATAATGATGCGAATCGGGCTTTAATGAGTTCGAATATGCAACGCCAAGCAGTTCCGCTCCTTCGGTCCGAGAAGTGCATTGTTGGAACTGGGTTGGAACGCCAAGTGGCTCTAGATTCAGGGGTTCCCGCTATAGCCGAACATGAGGGAAAGATCATTTATACCGATATTGACAAGATCGTTTTATCGGGCAACGGGAATACTTATAGTATTCCTTTAGTTCTGTATCAACGTTCAAACAAAAATACTTGTATGCATCAAAAAACCCAGGTTGGGCGGGGTAAATGCATTAAAAAGGGCCAAGTTTTAGCGGATGGTGCCGCTACAGTTGGTGGCGAACTCGCTTTAGGGAAAAACGTATTAGTAGCTTATATGCCATGGGAAGGTTACAATTTTGAGGATGCGGTACTTATTAGCGAACGTCTGATATATGGAGATATTTATACTTCTTTTCACATACGGAAATATGAAATTCAGACTCATGTGACAAGCCAAGGTCCCGAAAGGATCACTAATGAAATACCACATCTAGAAGCCCGTTTACTCCGCAATTTAGACAAAAATGGAATTGTGATGCTGGGATCTTGGGTAGAGACCGGCGATATTTTAGTAGGTAAATTAACGCCTCAGGCGGCGAAAGAATCATCCTATGCTCCGGAAGATAGATTATTACGGGCCATACTTGGCATTCAGGTCTCCACTTCAAAGGAAACTTGCCTAAAACTACCTATAGGTGGTAGGGGTCGAGTTATTGATGTGAGATGGGTCCAGAAAAAGGGGGGTTCCAGTTATAATCCCGAAACGATTTGTGTATATATTTCACAGAAACGTGAAATCAAAGTAGGTGATAAAGTAGCTGGAAGACATGGAAATAAGGGTATCATTTCAAAAATTTTGCCTAGACAGGATATGCCTTATTTGCAAGATGGAAGGCCTGTTGATATGGTTTTCAACCCATTAGGAGTACCCTCGCGAATGAATGTAGGACAGATATTTGAATGCTCGCTCGGGTTAGCGGGGGGTCTGCTAAATCGACATTATCGAATAGCACCTTTTGATGAGAGATATGAACAAGAGGCTTCGAGAAAACTCGTGTTTTCTGAATTATATGAAGCCAGTAAGCAAACATCGAATCCATGGGTATTTGAGCCGGAATACCCGGGAAAAAGCAGAATATTTGATGGACGAACGGGAGATCCTTTTGACCAACCTGTTCTAATAGGAAAGCCTTATATCTTGAAATTAATTCATCAAGTTGATGATAAAGTACACGGACGTTCCAGTGGGCATTATGCACTTGTTACCCAACAACCCCTTAGAGGAAGGTCAAAACAGGGGGGGCAGCGGGTAGGAGAAATGGAGGTTTGGGCTCTAGAGGGCTTTGGTGTTGCTCATATTTTACAAGAGATGCTTACTTATAAATCTGATCATATTAGAGCTCGCCAAGAAGTACTTGGTACTACAATCATTGGAGAAACAATACCTAGCCCCGAAGATGCTCCAGAATCTTTTCGATTGCTCGTTCGAGAACTACGATCTTTGGCTTTGGAACTGAATCATTTTCTTGTATCTGAGAAAAACTTCCAGATTAATAAGAAGGAAGCTTAA

>psbM

ATGGAAGTAAATATTCTCGCATTTATTGCTACTACACTGTTCGTTCTAGTTCCTACTGCTTTTTTGCTTATAATATACGTAAAAACGGTCAGTCAAAGTGATTAA

>psbD

ATGACTATAGCCCTTGGTAAATTTACCAAAGATGAAAAAGATTTATTTGATATTATGGATGACTGGTTACGGAGGGACCGATTCGTTTTTGTAGGTTGGTCCGGTCTATTGCTCTTTCCTTGTGCCTATTTCGCTTTAGGGGGTTGGTTCACAGGCACAACCTTTGTAACTTCATGGTATACCCATGGATTGGCCAGTTCCTATTTGGAAGGCTGCAACTTCTTAACCGCCGCAGTTTCGACCCCTGCTAATAGTTTAGCGCATTCTTTGTTGTTACTATGGGGTCCTGAAGCACAAGGGGATTTTACTCGTTGGTGTCAATTAGGCGGTCTTTGGACTTTTGTTGCTCTCCACGGTGCTTTCGGACTAATAGGTTTCATGTTACGTCAATTTGAACTTGCTCGCTCTGTGCAATTGCGACCTTATAATGCAATAGCATTCTCTGGTCCAATTGCTGTTTTTGTTTCTGTATTCCTGATTTATCCACTGGGTCAGTCTGGTTGGTTCTTTGCGCCTAGTTTTGGTGTAGCGGCGATATTTCGATTCATCCTTTTTTTCCAAGGGTTTCATAATTGGACATTGAACCCCTTTCATATGATGGGAGTTGCCGGTGTATTGGGCGCAGCTCTGCTATGTGCTATTCATGGCGCTACCGTAGAAAATACTTTATTTGAAGATGGTGATGGTGCAAATACATTCCGCGCTTTTAACCCAACGCAAGCTGAAGAAACTTATTCGATGGTCACCGCTAACCGCTTTTGGTCCCAAATCTTTGGGGTTGCTTTTTCCAATAAACGTTGGTTACATTTCTTTATGTTATTTGTACCAGTAACCGGTTTATGGATGAGTGCTCTTGGAGTAGTCGGCCTAGCCCTGAACCTACGTGCTTATGACTTCGTTTCCCAGGAAATCCGTGCAGCGGAAGATCCTGAATTTGAGACTTTTTACACAAAAAATATTCTTTTAAACGAGGGTATTCGTGCTTGGATGGCGGCTCAAGATCAGCCTCATGAAAACCTTATATTCCCTGAGGAGGTTCTACCCCGTGGAAACGCTCTTTAA

>psbZ

ATGACTATTGCTTTCCAATTGGCTGTTTTTGCATTAATTGCTACTTCATCAATCTTACTTATTAGTGTACCCGTTGTATTTGCTTCTCCGGATGGTTGGTCGAGTAACAAAAATGTCGTATTTTCTGGTACATCGTTGTGGATTGGATTAGTCTTTCTGGTGGGTATCCTTAATTCTCTCATCTCTTAA

>rps14

ATGGCAAGGAAAAGTTTGATTCATAGGGAGAAGAAAAGGCAAAAATTGGAACAAAAATATCATTTGATTCGTCGATCCTCAAAAAAAGAAATAAACAAAGTTCCGTCGTTGAGCGATAAATGGAAAATTCATGGAAAGCTACAGTCCTCACCGCGTAATAGTGCACCTACACGTCTTCATCGACGTTGTTTTTCGACCGGAAGGCCGAGAGCTAACTATCGAGACTTTGGACTATCCGGGCACATACTTCGTGAAATGGTTCATGCATGTTTGTTGCCGGGGGCAACAAGATCAAGTTGGTAA

>psaB

ATGGCATTAAGATTTCCAAGGTTTAGCCAAGGCTTAGCTCAGGACCCCACTACTCGTCGTATTTGGTTTGGTATTGCTACCGCACATGACTTCGAGAGTCATGATGATATTACTGAGGAACGTCTTTATCAGAATATTTTTGCTTCTCACTTCGGACAATTAGCAATAATTTTTCTGTGGACTTCCGGAAATCTCTTTCATGTAGCTTGGCAAGGAAATTTTGAGGCATGGGTACAGGACCCTTTACATGTAAGACCTATTGCTCATGCAATTTGGGATCCTCATTTTGGTCAACCGGCCGTGGAAGCTTTTTCTCGGGGAGGTGCTCTTGGCCCGGTGAATATCGCTTATTCTGGTGTTTATCAGTGGTGGTATACAATTGGTTTACGCACTAATGAGGATCTTTATACTGGAGCTCTTTTTCTATTATTTCTTTCTGCCATATCCTTAATAGCGGGTTGGTTACACCTACAACCGAAATGGAAACCGAGTGTTTCGTGGTTCAAAAATGCCGAATCTCGTCTCAATCATCATTTGTCAGGACTGTTCGGAGTAAGTTCCTTGGCTTGGACAGGACATTTAGTACATGTGGCTATTCCTGGATCCAGGGGGGAATATGTTCGATGGAATAATTTCTTAGATGTATTACCGCATCCCCAAGGGTTAGGCCCACTTTTTACAGGTCAGTGGAATCTTTATGCTCAAAACCCCGATTCAAGTAGTCATTTATTTGGTACCTCCCAAGGATCAGGAACTGCCATTCTAACCCTTCTCGGGGGATTCCATCCACAAACGCAAAGTTTATGGCTGAGCGATATTGCTCATCATCATTTAGCTATTGCAATTCTTTTCCTGATCGCGGGTCACATGTATAGAACAAATTTCGGGATTGGGCACAGTATAAAAGATCTTTTAGAAGCACATATTCCTCCGGGAGGACGATTGGGGCGCGGGCATAAGGGTCTTTATGACACAATCAACAATTCGCTTCATTTTCAATTAGGCCTTGCTCTAGCCTCTTTAGGGGTTATTACTTCCTTGGTAGCTCAACACATGTACTCTTTACCTGCTTATGCGTTCATAGCGCAAGATTTTACTACTCAAGCTGCGTTATATACTCATCACCAATACATCGCAGGATTCATCATGACCGGAGCTTTTGCTCATGGAGCTATCTTTTTTATTAGAGATTACAATCCGGAACAGAATGAGGATAATGTATTGGCAAGAATGTTAGACCATAAGGAAGCTATTATATCCCATTTAAGTTGGGCCAGTCTCTTTCTGGGGTTCCATACTTTGGGACTTTATGTTCATAATGATGTCATGCTTGCTTTTGGTACTCCGGAGAAACAAATCTTGATCGAACCGATATTTGCCCAATGGATACAATCTGCTCATGGTAAAACTTCATATGGATTCGATGTACTTTTATCTTCAACGAATGGCCCTGCATTTAATGCGGGTCGAAGCATATGGTTGCCTGGCTGGTTAAATGCTATTAATGAGAATAGTAATTCTCTATTCTTAACAATAGGCCCTGGAGACTTCTTGGTTCATCATGCTATTGCTCTAGGTTTACATACAACTACATTGATCTTAGTAAAGGGGGCTTTAGATGCACGGGGTTCCAAGTTAATGCCAGATAAAAAGGATTTCGGTTATAGTTTTCCTTGCGACGGTCCGGGACGAGGCGGTACTTGTGATATTTCGGCTTGGGACGCATTTTATTTGGCAGTTTTCTGGATGTTAAATACCATTGGGTGGGTTACTTTTTATTGGCATTGGAAACACATCACGTTATGGCAGGGTAACGTTTCACAGTTTAATGAATCTTCCACTTATTTGATGGGCTGGTTAAGAGATTATCTATGGTTAAACTCTTCCCAACTTATCAATGGGTATAACCCGTTTGGTATGAATAGTTTATCAGTCTGGGCGTGGATGTTCTTATTTGGACATCTTGTTTGGGCTACTGGATTTATGTTCTTAATTTCCTGGCGCGGGTATTGGCAAGAATTGATTGAAACTTTAGCATGGGCTCACGAACGCACACCCTTGGCTAATTTGATTCGATGGAGAGATAAACCAGTGGCTCTTTCCATTGTGCAAGCAAGATTGGTTGGATTAGCCCACTTCTCTGTAGGTTATATATTCACTTATGCGGCTTTCTTGATTGCCTCTACATCGGGCAAATTTGGTTAA

>psaA

ATGATTATTCGTTCGCCGGAACCAGAAGTAAAAATTTTGGTAGATAGGGATCCCATAAAGACTTCTTTCGAGGAATGGGCCAGACCGGGTCATTTCTCAAGAACCATAGCTAAGGGACCTGATACTACCACTTGGATCTGGAACCTACATGCTGATGCTCACGACTTCGATAGCCATACCAGTGATTTGGAGGAGATCTCTCGAAAAGTATTTAGTGCCCATTTCGGACAACTCTCCATCATTTTTCTTTGGCTGAGCGGCATGTATTTCCACGGTGCTCGTTTTTCCAATTATGAAGCCTGGCTAAGCGATCCTACTCACATTGGACCCAGTGCACAGGTGGTTTGGCCAATAGTGGGCCAAGAAATATTGAACGGTGATGTGGGCGGGGGTTTCCGAGGAATACAAATAACCTCCGGGTTTTTTCAGATTTGGCGAGCATCTGGAATAACTAGTGAATTACAACTCTATTGTACCGCAATTGGCGCATTGATTTTTGCAGCCTTAATGCTTTTTGCTGGTTGGTTCCATTATCACAAAGCTGCTCCAAAATTGGCTTGGTTTCAGGATGTAGAATCTATGTTGAATCACCATTTAGCGGGGCTGCTAGGGCTCGGGTCCCTTTCTTGGTCCGGGCATCAAGTACATGTATCTTTACCGATTAACCAATTTCTAAACGCTGGAGTAGATCCTAAAGAGATCCCACTTCCTCATGAATTTATCTTGAATCGGGATCTTTTGGCTCAACTTTATCCCAGTTTTGCCGAAGGAGCAACCCCATTTTTTACCTTGAATTGGTCAAAATATGCGGACTTTCTTACTTTTCGTGGCGGATTAGATCCAGTAACTGGGGGTCTATGGCTGACCGATATTGCACACCATCATTTAGCTATTGCAATTCTTTTCCTGATCGCGGGTCACATGTATAGGACCAACTGGGGAATTGGTCATGGTCTAAAAGATATTTTAGAGGCCCATAAAGGTCCATTTACGGGTCAAGGCCATAAAGGACTATATGAGATCCTAACAACATCATGGCATGCTCAATTATCGCTTAACCTAGCTATGTTAGGATCTTTAACCATTGTTGTAGCTCACCATATGTATTCCATGCCCCCTTATCCATATCTAGCTACTGACTATGGTACACAACTGTCATTGTTCACACATCACATGTGGATTGGTGGATTTCTCATCGTTGGCGCTGCTGCGCATGCAGCCATTTTTATGGTAAGAGACTATGATCCAACTACTCGATACAATGATCTCTTAGATCGTGTCCTTCGGCATCGCGATGCAATCATATCACATCTCAACTGGGTATGTATATTTCTAGGATTTCACAGTTTTGGTTTATATATTCATAATGATACAATGAGTGCTTTAGGGCGTCCACAAGATATGTTTTCAGATACTGCGATACAATTACAACCCGTCTTTGCTCAATGGATACAAAACACCCACGCCTTAGCACCTGGTGGAACGGCCCCTGGTGCAACAGCAAGCACCAGTTTGACTTGGGGGGGTGGTGATTTAGTGGCAGTGGGTGGAAAAGTTGCTTTGTTACCTATTCCATTAGGAACCGCGGATTTTTTGGTACATCACATTCACGCATTTACGATTCATGTGACGGTATTGATACTCCTGAAAGGAGTTCTATTTGCTCGTAGCTCGCGTTTGATACCGGATAAAGCAAATCTTGGGTTTCGTTTCCCTTGTGATGGGCCTGGAAGAGGGGGGACATGTCAAGTATCCGCTTGGGATCATGTCTTCTTAGGACTATTCTGGATGTACAATGCAATTTCGGTAGTAATATTCCATTTCAGTTGGAAAATGCAGTCAGATGTTTGGGGTAGTATAAGCGATCAGGGGGTGGTAACTCATATTACAGGAGGAAACTTTGCGCAGAGTTCCATTACGATTAATGGGTGGCTCCGCGATTTTTTATGGGCACAGGCATCCCAGGTAATTCAGTCTTATGGTTCTTCATTATCTGCATATGGCCTTTTTTTCCTAGGTGCTCATTTTGTCTGGGCTTTTAGTTTAATGTTTCTATTCAGCGGGCGTGGTTACTGGCAAGAACTTATTGAATCCATCGTTTGGGCTCATAATAAATTAAAAGTTGCTCCTGCTACTCAGCCTAGAGCCTTGAGCATTGTACAAGGACGTGCTGTAGGAGTAACCCATTACCTTCTGGGTGGAATTGCCACAACATGGGCGTTCTTCTTAGCAAGAATTATTGCAGTAGGATAA

>ycf3

ATGCCTAGATCGCGGATAAATGGAAATTTTATTGATAAGACCTTTTCAATTGTAGCCAATATATTATTACGAATAATTCCGACAACTTCAGGAGAAAAAGAGGCATTTAGCTATTACAGAGATGGTGCGATGTCAGCTCAATCTGAAGGCAATTATGCGGAAGCTTTACAGAATTATTATGAAGCTATGCGACTAGAAATTGATCCCTACGATCGAAGCTATATACTCTATAACATAGGCCTTATCCACACAAGTAACGGAGAACATACAAAAGCTTTAGAATATTATTTTCGGGCACTCGAACGAAATCCATTCTTACCACAAGCTTTGAATAATATGGCTGTGATCTGTCATTACCGGGGAGAACTGGCCGTTCGGCAGGGAGATTCTGAAATTGCGGAGGCTTGGTTCAACCAAGCCGCCGAGTATTGGAAACAAGCTATTGCGCTTACTCCTGGTAATTATATTCAAGCGCAGAATTGGTTGAAGATCACGGGGCGTTTCGAATAA

>rps4

ATGTCACGTTACCGAGGGCCTCGTTTCAAAAAAATACGCCGTCTGGGGGCTTTGCCGGGACTAACGAGTAAAGGGCCTAGAGCCGGAAGCGATTTTAGAAACCAATCGCGCTCCGGAAAAAAATCTCAATATCGAATTCGTTTAGAAGAAAAACAAAAATTGCGTTTTCATTATGGTCTTACAGAACGACAATTACTTAAATATGTTCGTATCGCCGGAAAGGCCAAAGGGTCCACCGGTCTGGTTTTACTACAATTACTTGAAATGCGTTTGGATAACATTCTTTTTCGATTGGGTATGGCTTCAACTATTCCTCAAGCCCGCCAATTAGTTAACCACCGACATATTTTAGTTAATGGGCGTATAGTCGATATACCAAGTTATCGTTGCAAACCCCGAGATATTATTACAACAAAGGATGACCAAAAATCTAGATCTCTGATTCAAAATTATCTTGATTCATCCCACCATGAGGAATTGCCAAAGCATTTGACTCTTCACGCATTCCAATATAAAGGATTAGTCAATCAAGTAATAGATAGTCAATGGGTCGGTTTGAAAATAAATGAATTGCTTGTCGTAGAATATTATTCTCGTCAGACTTAA

>ndhJ

ATGCAGGGTCGTTTGTCTGCTTGGCTAGTCAAGCATGGGCTAGTTCATAGATCTTTGGGCTTCGATTACCAAGGAATAGAAACTTTACAAATAAAGCCCGAGGATTGGCATTCCGTTGCTGTCATTTTGTATGTATATGGTTACAATTATCTACGTTCCCAATGTGCCTATGATGTAGCACCGGGTGGACTGTTAGCCAGTGTGTATCATCTTACGCGACTAGAGTATGGTGTAGATCAACCGGAAGAGGTATGCATAAAAGTATTTGCTCCAAGGAGTAATCCGAAAATTCCATCTGTTTTCTGGGTTTGGAAAAGTGCGAATTTTCAAGAACGGGAATCTTATGATATGTTGGGAATCCTTTATGATAATCATCCACGACTGAAACGTATCTTAATGCCGGAAAGTTGGATAGGGTGGCCCTTGCGTAAGGATTATATTGCCCCCAATTTTTATGAAATACAAGATGCATATTGA

>ndhK

ATGAATTCCATTGAATTTTCCTTACTTGATCGAACAACCCCAAATTCATTTATTTCAACTACATCAAATGATCTTTCAAATTGGTCAAGACTCTCCAGTTTATGGCCGCTTCTTTATGGTACCAGTTGTTGTTTCATTGAATTTGCTTCATTAATCGGCTCGCGGTTCGATTTTGACCGTTATGGACTGGTACCAAGATCGAGCCCTAGACAGGCGGACCTAATTTTAACAGCTGGTACCGTAACAATGAAAATGGCTCCTTCTTTAGTGAGATTATATGAACAAATGCCTGAACCAAAATATGTTATTGCTATGGGAGCATGTACAATTACAGGGGGGATGTTCAGTACCGATTCGTATAGTACTGTTCGGGGAGTTGATAAGCTAATTCCTGTGGATGTTTATTTGCCGGGCTGTCCGCCTAAACCAGAGGCAGTTATAGATGCCATAACAAAACTTCGTAAGAAAATATCTCGAGAAATCTACGAAGATCGAATTCGATTGCAAGGGGAGAATCGCTCGTTTACTTTTACTACCAATCACAAGTTTCGTGTTGTATGCAGTACTCATACTGGAAATTATGATCAAGGATTACTCTATCAACCACCATCTACGTCAGAAATTCCACCTGAAACCTTTTTCAAATACAAAAGTTCAGTATCTTCCCCCGAATTCATTAATTAA

>ndhC

ATGTTTCTGCTTTACAAATATGATATTTTCTGGGCATTTCTAATAATATCAAGCGTTATTCCTATTTTTGCATTTCTAATTTCCGCAGTTTTAGCCCCGATTAACAAAGGGCCAGAGAAACTTTCTAGTTATGAATCGGGTATAGAACCAATGGGCGATGCTTGGTTACAATTTCGAATCCGGTATTATATGTTTGCTCTAGTTTTTGTTGTTTTTGATGTTGAAACCGTTTTTCTTTATCCATGGGCAATGAGTTTTGATGTATTGGGGGTATCCGTATTTATAGAAGCTTTCATTTTCATGCTTATCCTAATTGTTGGTTCAGTTTATGCGTGGCGAAAAGGAGCATTAGAGTGGTCTTAG

>atpE

ATGACCTTAAATCTTTGTGTACTGACCCCGAATCGAATTGTTTGGGATTCAGAAGTGAAAGAAATCATTTTATCGACTAATAGTGGACAAATAGGCGTATTACCAAATCACGCGCCTATTGCCACAGCTGTAGATATAGGTATTTTAAGAATCCGCTTTAACGACCAATGGGTAACGATGGCTCTGATGGGTGGTTTTGCTAGAATAGGGAATAATGAGATCACTATTTTAGTAAATGATGCGGAGAAGAGTAGTGACATTGATCCCCAAGAAGCCCAGCAAACTCTTGAAATAGCGGAAGCTAATTTGAGGAAAGCTGAAAGCAAGAGACAAACAATTGAGGCAAATCTAGCTCTCAGACGAGCTAGGACACGAGTAGAGGTTATCAATGCGATTTGA

>atpB

ATGAGAATAAATCCTACTACTTCCGGCCCTGCGGTTTCCGCGCTTGCAAAAAAAAACCTGGGACATATCGCTCAAATCATTGGTCCGGTACTGGATGTAGCCTTTCCCCCCGGCAAGATGCCTAATATTTACAATGCTCTGGTAGTTAAGGGTCGAGATCCTGTCGGTCAACCAATTAATGTGACTTGCGAGGTACAGCAATTATTAGGGAATAATCGGGTTAGAGCTGTAGCCATGAGTGCTACAGATGGTCTAACGAGAGGGATGGAAGTGATTGACACGGGAGCTCCTCTAAGTGTTCCAGTCGGCGGAGCGACTCTAGGACGAATTTTCAACGTGCTTGGAGAGCCTGTTGATAATTTAGGTCCTGTAGATACTCGCACAACATCCCCTATTCATAAATCCGCGCCTGCCTTTATACAGTTAGATACAAGATTATCTATTTTTGAAACAGGAATTAAAGTAGTAGATCTTTTAGCTCCTTATCGTCGGGGAGGAAAAATCGGACTATTCGGGGGGGCTGGAGTGGGTAAAACAGTACTCATTATGGAATTGATCAACAACATTGCCAAAGCTCATGGGGGTGTATCCGTATTTGGGGGAGTCGGTGAACGTACTCGTGAAGGAAATGATCTTTACATGGAAATGAAAGAGTCTGGAGTAATTAATGAACAAAATATTGCGGAATCTAAAGTGGCTCTAGTCTACGGTCAGATGAATGAACCGCCAGGAGCTCGTATGAGAGTTGGTTTGACAGCCCTAACTATGGCAGAATATTTCCGAGATGTTAATGAACAAGACGTACTTCTATTTATCGACAATATCTTCCGTTTCGTCCAAGCGGGATCCGAGGTATCCGCCTTATTGGGTAGAATGCCTTCTGCTGTGGGTTATCAACCTACCCTTAGTACCGAAATGGGTTCTTTACAAGAAAGAATTACTTCTACCAAAGAGGGGTCCATAACTTCTATTCAAGCAGTTTATGTACCCGCAGATGATTTGACTGACCCCGCTCCTGCCACGACATTTGCCCATTTAGATGCTACTACCGTACTATCAAGAGGATTAGCTGCTAAAGGTATCTATCCAGCAGTTGATCCTTTAGACTCAACGTCAACTATGCTCCAACCTCGGATCGTTGGCGAGGAACATTATGAAACTGCGCAAAGAGTTAAGCAAACTTTACAACGTTACAAAGAACTTCAGGACATTATAGCTATCCTTGGGTTGGACGAATTGTCCGAAGAGGATCGTTTAACCGTAGCAAGAGCGCGAAAAATTGAGCGTTTCTTATCACAACCCTTTTTCGTAGCAGAAGTATTTACCGGTTCCCCGGGGAAATATGTTGGTCTAGCAGAAACTATTAGAGGGTTTAAATTGATCCTGTCCGGAGAATTAGATGGTCTTCCTGAACAGGCCTTTTATTTGGTAGGTAACATCGATGAAGTTACTGCGAAGGCTACAAACTTAGAAATGGAGAGTAATTTGAAGAAATGA

>rbcL

ATGTCACCACAAACAGAGACTAAAGCGAGTGTTGGATTCAAAGCCGGTGTTAAAGATTATAAATTGACTTATTATACTCCTGACTATGCAACCAAAGATACTGATATCTTGGCAGCATTCCGAGTAAGTCCTCAACCCGGAGTTCCACCCGAGGAAGCGGGGGCTGCGGTAGCTGCGGAATCTTCTACTGGTACATGGACAACTGTGTGGACCGATGGGCTTACCAGCCTTGATCGTTACAAAGGGCGATGCTACAACATTGAGCCCGTTGCTGGAGAAGAAAATCAATATATATGTTATGTAGCTTACCCGTTAGACCTTTTTGAAGAAGGTTCTGTTACTAACATGTTTACTTCCATTGTGGGTAATGTATTTGGTTTCAAAGCCCTGCGCGCTCTACGTCTAGAGGATCTACGAATCCCTACCGCGTATACTAAAACTTTCCAAGGCCCGCCTCACGGCATCCAAGTTGAGAGAGATAAATTGAATAAGTATGGACGTCCCCTGTTGGGATGTACTATTAAACCTAAATTGGGGTTATCCGCTAAGAATTACGGTAGGGCAGTTTATGAATGTCTACGCGGTGGACTTGACTTTACCAAAGATGATGAGAACGTGAACTCCCAACCATTTATGCGTTGGAGGGACCGTTTCTTATTTTGTGCGGAAGCAATTTATAAAGCGCAAGCTGAAACAGGTGAAATCAAAGGTCATTACTTGAATGCTACTGCAGGGACATGCGAAGAAATGATGAAAAGGGCTATCTTTGCCAGAGAGTTGGGAGTTCCTATCGTAATGCATGACTACTTAACAGGGGGATTCACCGCAAATACTAGCTTGGCTCATTATTGCCGAGATAATGGTCTACTTCTTCACATCCACCGTGCAATGCATGCAGTTATTGATAGACAGAAGAATCATGGTATACACTTTCGTGTACTAGCTAAAGCTTTACGTATGTCTGGTGGAGATCATATTCACGCTGGTACAGTAGTAGGTAAACTTGAAGGGGAAAGAGACATAACTTTGGGATTTGTTGATTTACTACGTGATGATTTTATTGAAAAAGATAGAAGCCGCGGTATTTATTTCACTCAAGATTGGGTCTCTCTACCAGGTGTTCTGCCCGTGGCTTCCGGAGGTATTCACGTTTGGCATATGCCTGCTTTGACCGAGATCTTTGGAGATGATTCCGTACTACAATTTGGTGGAGGAACTTTAGGACACCCTTGGGGAAATGCACCCGGCGCCGTAGCTAATCGAGTAGCTCTAGAAGCATGTGTACAAGCTCGTAATGAAGGACGCGATCTTGCTCGCGAAGGTAATGAAATTATCCGGGAGGCGAGCAAATGGAGTCCTGAACTGGCTGCTGCTTGTGAAGTATGGAAGGAGATCAAATTTGAATTCCCAGCAATGGATACTTTGTAA

>accD

ATGAAAAAAAAAAAGGGGTGGTTCAATTCGATGTTATCTAAGGGTAAGGAGGAATTAGAATACAGGTGTGGGTTAAGTAAATCAATGGATAGTCTTGGTCCTATTCAAAATACCAGTGTAAGCGAGGACCCGATTCGAAATGATAAGGATAAAAACATTCATAGTTCGAGGGATAGTGACTGTTCGAGTTACAGCAATTTAGCTGGTGTCAGGGACATTCGTAATTTCATCTCGGATGACACCTTTTTTATTAAGGATAGTAATAGGGACAGCTATTCCATATATTTTGATATTGAAAATCAAATTTTGGAAATAGACAACGATCATTCTTTTCTAAGTGAACTAGAAAGTTCTTTTTATAGCTTTCGTAATTATAGTTCTAGGAATAATGGATCCAAAAGTGATGATCCCGACTCTGATCGTTACATGTATGATACTCAATCGAGTTGGAATAATTACATTCATAATTGCCTCGACTCTTATCTTCATTCTCAAATCTGTATTGATAGTCACATTTTAAGTAGTAGTGACTATTATAGTGCCAGTTACATTTCTAATTTCATTTCTAGTGAAAGTGGAAATAGTAGTGAAAGCGAGAGTTCCAATATACAAAGTAGTAGTGACTATTATAGTGCCAGTTACATTTCTAGTGAAAGTGGAAATAGTAGTGAAAACGAGAGTTCCAATATACAAAGTAGCACGAATGGTAGTGATTTAACTATAAGCGAAAGTTCTGATGATCTCGATGTAACTCAAAAATACAGGCATTTATGGGTTCAATGCGAAAATTGTTATGGATTAAATTATAAGAAATTTCTTAAGTCAAAAATGTATATTTGTGAACAATGTGGATTTCATTTGAAAATGAGTAGCTCAGATAGAATCGAACTTTCGGTTGATCCAGGTACTTGGGATCCGATGGATGACGACATGGTCTCTATAGATCCCATTGAATTTCATTCAGAAGAGGAACCTTATAAAAATCGTATTGATTCTTATCAAACAAAGACAGGATTAACAGAGGCTGTTCAAACAGGTACAGGGCAACTAAACGGGATTCCCATCGCAATTGGGGTTATGGATTTTCAGTTTATGGGGGGTAGTATGGGATCCGTAGTAGGCGAGAAAATCACCCGTTTGATCGAGTATGCTGCCAATAAATTTTTACCTCTTCTTCTAGTGTGTGCTTCCGGGGGAGCACGCATGCAAGAAGGAAGTTTGAGCTTGATGCAAATGGCTAAAATATCTTCTGCTTTATATGATTATCAATTAAATAAAAAGTTATTCTATGTATCAATTCTTACATCTCCTACTACTGGTGGAGTGACAGCTAGTTTTGGTATGTTGGGGGATATCATTATTGCTGAACCTAATGCCTATATTGCATTTGCGGGTAAAAGAGTAATTGAACAAACATTGAATAAGACAGTACCTGAAGGTTCACAAGAGGCTGAATATTTATTCCATAAGGGCTTATTCGATCCAATCGTACCACGTAATCCTCTAAAAGGTGTTCTGAGCGAGTTATTTCAGTTCCACGCCTTTTTTCCTTTGAATCAAAATTAA

>psaI

ATGACAATTCTCAACAGCTTTCCCTCAATTTTTGTGCCTTTAGTGGGCCTAGTATTTCCGGCAATGGCAATGGCTTCTTTATTTCTTTATCTTGAAAAAAATAAGATTTTTTAA

>ycf4

ATGAGTTGGCGATCAGAATATCTATGGATAGAATTTATAGCAGGCTCTCGCAAAACAAGCAATTTCTGCTGGGCTCTTATCCTTTTTTTAGGTTCATTAGGATTCTTAGTGGTTGGAATTTCGAGTTATTTTGATAGGAATTTGCTATCTTTATTTCCGTCTCAGCAAATCAATTTTTTTCCACAAGGGATCGTGATGTCTTTCTACGGGATCGCGGGTCTCTTTATTAGTTCCTATTTGTGGTGCACAATTACATGGAATGTAGGTAGCGGTTATGATCGATTTGATACAAAAGAGGGAATAGTGTGTATTTTTCGTTGGGGATTTCCTGGAAAAAATCGCCGCATCTTTCTACGATTCCTTATGAAAGATATTCAGTCCATCAGAATAGAAGTTAAAGAGGGTATTTATGCTCGTCGTGTCCTTTATATAGAAAGCAGAGGCTTGGGGGCCATTCCCTTGAATCGTACTGATGAGAATTTGACTCCACGAGAAATTGAGCAAAAGGCTGCGGAATTGGCCTATTTCTTGCGTGTACCAATTGAAGGGTTTTGA

>cemA

ATGAAAAAAAAGAATGCATCCATTCCCCTTAGATATCTTTCATCTATAGTATTTGTAGTATTTTTGCCCTGGTGGATCCCTCTCTCATTTAATAAAAGTTTGGAATCCTGGGTTACTAATTGGTGGAATACTAGTCAACCCGAAACCTTTTTGAATGATATTCGGGAAAAGGCTATTCTAGAAAAATTCATAGAATTAGAGGAATTATTCCTCTTGGACGAAATGGAAATGATAAAGGAATTTCCGGAAAGACATCTAGAAAAGCTTCGCATAGGGCTCCAGAAAGAAACAATCCAATTAATCAAGATGCACGATGAGGATCATATCCATACGATTTTTCACTTCTCGACAAATACAATCTGCTTTGTTATTCTAAGTGGTTATTCGATTCTGTGTAATGAAGAACTTTTTATTCTTAACTCTTGGGTTCAAGAATTCCTATATAATTTAAGCGACACAATAAAAGCCTTTTCGATTCTTTTCGTAACTGATTTATGTATCGGATTCCATTCGCCCCGCGGTTGGGAACTACTGATTGGCTATGCCTACAACGATTTTGGATTTGCTCATAATGATATTATTCTATCTGTTCTTGTTTCCACTTTTCCAGTCATTCTAGATACGTTTTTTAAATATTGGCTTTTTTCTTATTTAAATCGTGTATCTCCGTCACTTGTAGTGATTTATCATTCAATGACTGAGTGA

>petA

ATGCAAATTAGAAATACCTTTTCTTCGTTAAAGGGCGAGATTACTCGATTCATTTCCGTATCCCTCATGATATATATAATAACTCGGGCATCAATTTCAAATGCATATCCCGTTTTTGCGCAGCAGGGTTTTGAAAATCCACGAGAGGCAACTGGTCGGATTGTATGCGCCAATTGTCATTTAGCTAATAAGCCCGTGGATATTGAGGTTCCACAGGCGGTACTCCCTGATACTGTATTTGAAGCAGTTGTTAGAATTCCGTATGATATGCAACTGAAACAAGTTCTTGCTAATGGTAAAAGGGGGTCTTTGAATGTGGGGGCCGTTCTTATTTTACCAGAGGGGTTTGAATTAGCCCCCTCCGACCGTATTTCGCCCGAGATGAAAGAAAAGATAGGCAAGCTGTCTTTTCAGACCTACCGACCTACTAAAAAAAATATTCTTGTGATAGGGCCAGTTCCTGGTCAGAAATATAGTGAAATCACTTTTCCTATTCTTTCCCCGAACCCTGCGACTAATAAGGATGCTCACTTCTTAAAATATCCAATATATGTAGGTGGGAACAGGGGGAGGGGTCAGATTTATCCCGACGGGAACAAAAGTAACAATACGGTTTATAATGCTACAGCTGCGGGTATAGTAAGCAAACTCTTACGAAAAGAAAAAGGGGGATACGAAATAACCATAACGGATGCATCGAATGGGCGTGAAGTGGTTGATATTATCCCTCCAGGCCCAGAACTTCGTGTTTCAGAGGGACAATCTATCAAACTTGATCAACCATTAACAAGTAATCCTAATGTAGGTGGGTTTGGTCAGGCAGATGCAGAAATAGTACTTCAAGATCCATTACGTGTCCAAGGCCTTTTGTTCTTTTTGGCATCTGTTGTTTTGGCACAAATCTTTTTGGTTCTTAAAAAGAAACAGTTTGAGAAGGTCCAATTGTCCGAAATGAATTTCTAG

>psbJ

ATGGCCGATACTACTGGAAGGATTCCCCTTTGGATAATAGGTACTGTAACTGGTATTCCTGTGATCGGTTTAATAGGCATTTTCTTTTATGGTTCATATTCCGGATTAGGTTCGTCCTTGTAG

>psbL

ATGACACAATCAAACCCGAACGAACAAAATGTTGAATTGAACCGTACCAGTCTCTACTGGGGGTTATTACTCATTTTTGTACTTGCTGTTTTATTTTCCAATTACTTCTTCAATTAA

>psbF

ATGACCATAGATCGAACCTATCCAATTTTTACAGTGCGATGGTTGGCCGTGCACGGACTAGCTGTACCTACCGTTTCTTTTTTGGGGTCAATATCAGCAATGCAGTTCATCCAACGATAA

>psbE

ATGTCTGGAAGCACAGGAGAACGTTCTTTTGCTGATATTATTACCAGTATTCGATACTGGGTCATTCATAGCATTACTATACCTTCCCTATTCATTGCGGGTTGGTTATTCGTCAGCACGGGGTTAGCTTACGATGTGTTTGGAAGCCCTCGTCCAAACGAGTATTTTACAGAGAGCCGACAAGGAATTCCATTAATAACCGGCCGTTTTGATTCTTTGGAACAACTCAACGAATTTAGTAGATCTTTTTAG

>petG

ATGATTGAAGTCTTTCTATTTGGAATCGTCTTAGGTCTAATTCCTATTACTTTGGCGGGATTATTCGTAACCGCATATTTACAATACAGACGTGGTGATCAGTTGGACCTTTGA

>psaJ

ATGCGAGATCTAAAAACATATCTTTCCGTGGCACCGGTACTAAGTACTCTATGGTTCGGGTCTTTAGCAGGGTTATTGATAGAAATCAACCGTTTATTCCCGGACGCATTGACATTTCCTTTTTTTTCATTCTAG

>rpl33

ATGGCCAAGGGTAAAGATGTCCGAGTAAGGGTTATTTTGGAATGTACTAGTTGTGTTCGAAACGGTGTTAATAAGGAATCAAGGGGTATTTCCAGATATATTACTCAAAAGAATCGACACAATACACCTAGTCGATTGGAATTGAGAAAATTCTGTCCCTATTGTTACAAACATACACTTCACGGGGAGATAAAAAAATAG

>rps18

ATGGATAAAACCAAGCGACTCTTTCTTAAATCCAAGCGATCTTTTCGTAGGCGTTTGCCCCCGATCCAATCGGGGGATCGAATTGATTATAGAAACATGACTTTAATTAGTCGATTTCTTAGTGAACAAGGAAAAATATTATCTAGACGGGTGAATAGATTGACCTTAAAAGAACAACGATTAATTACTATTGCTATAAAACAAGCTCGTATTTTATCTTCGTTACCTTTTCTTAATAATGAGAAACGATTTGAAAGAAGTGGGTCGACCACTAGAACTCCAGGTCTTCGAACCAGAAAAAAATAG

>rpl20

ATGACCAGAATTAGACGAGGATATATAGCTCGGAGACGTAGAACAAAAATGCGTTTATTTGCATCAAGCTTTCGCGGGGCTCATTCAAGACTTAGTCGAACAATTACTCAACAGAAAATAAGAGCTTTGGTTTCGGCTCATCGTGATAGAGATAGGAAAAAAAGAGATTTTCGTCGTTTGTGGATCACTCGAATAAATGCAATAATTCGCGGAAATAGGGTATCCTATATTTATAGTAGATTAATAAACGATTTGTATAAGGCGCAGTTGGTTCTTAATCGTAAGATACTTGCACAAATAGCTATATCAAATAGGAATTGTCTTTATATGATTTCCAATGAGATCATAAAATAA

>rps12

ATGCCAACTATTAAACAACTTATTAGAAACCCAAGACAGCCAATCAGAAACGTTACAAAATCCCCCGCTCTTGGGGGATGCCCTCAGCGCCGAGGAACATGTACAAGGGTGTATGTGCGACTCGTTACTATCACCCCCAAAAAACCAAACTCTGCCTTACGTAAAGTTGCCAGAGTACGATTAACCTCTGGGTTTGAAATCACTGCTTATATACCGGGTATTGGCCATAATTCACAAGAACATTCTGTAGTCTTAGTAAGAGGGGGGAGGGTTAAGGATTTACCCGGTGTGAGATATCACATTGTTCGAGGAACCCTAGATGCTGTCGGAGTAAAGGATCGTCAACAAGGGCGTTCTAGTGCGTTGTAG

>clpP

ATGCCTATTGGTGTTCCAAAAGTACCTTATCGAAGTCCCGGGGACAAGCATCCATCTTGGGTTGACATAAACCGACTTTATCGAGAAAGATTACTTTTTTTAGGTCAAATGGTTGAGAGTGATATCTCGAATCAACTTATTGGTATTATGGTATATCTCAGTATCGAGAACGAGACCAAGGATTTGTATTTATTTATCAACTCTCCTGGCGGATGGGTAATACCCGGAATAGCAATTTATGATACTATGCAATTTGTGCGACCAGATGTACAGACAATATGCATGGGATTGGCCGCCTCCATGGGGTCTTTTCTCCTGGCTGCAGGGGCAAGTACCAAACGTCTAGCATTCCCTCACGCTAGGGTCATGATCCATCAACCTATTGGCGCTTTTTATGGGGCACAAGCGGGAGAATTTATCCTGGATACGGAAGAACTACTGAGACTGCGCGAAATCCTTACAATGGTTTATGTACAAAGATCGGGCAAGCCCTTATGGGTTGTATCCGAAGACATGGAAAGGGATACTTTTATGTCAGCAACAGAAGCCCAAGCTTATGGAATTGTTGATCTTGTAGCGGTTGGATAA

>psbB

ATGGGTTTGCCTTGGTATCGTGTTCATACCGTCGTATTGAATGATCCCGGTCGTTTGATTTCTGTCCATATAATGCATACAGCCCTAGTTGCGGGTTGGGCCGGTTCAATGGCTCTATATGAATTAGCTGTTTTTGATCCCTCCGATCCAGTTCTTGATCCAATGTGGAGACAAGGCATGTTCGTTATACCCTTCATGACTCGTTTAGGAATAACCGATTCATGGGGCGGTTGGAGTATTACGGGGGGGACGGTAACGAATCCGGGTATTTGGAGTTACGAAGGTGTAGCCGGGGCACATATTGTGTTTTCGGGCTTGTGCTTCTTGGCAGCTATCTGGCATTGGGTGTATTGGGATCTAGCAATATTTGTCGATGACCGTACGGGAAAACGCTCTTTGGATTTGCCTAAAATCTTTGGAATTCATTTATTTCTCTCAGGAGTGGCTTGCTTTGGTTTTGGGACATTTCATGTAACAGGATTGTATGGTCCTGGAATATGGGTGTCCGACCCTTATGGACTAACTGGAAAGGTACAATCTGTAAATCCAGCATGGGGTGTGGAAGGTTTTGATCCTTTTGTTCCAGGAGGAATAGCCTCTCATCATATTGCAGCAGGGACATTGGGCATATTAGCAGGCTTATTCCATCTTAGTGTCCGCCCACCTCAACGCCTATACAAAGGATTACGTATGGGCAATATTGAAACCGTTCTTTCCAGCAGCATCGCTGCTGTCTTTTTTGCAGCGTTTGTTGTTGCTGGAACTATGTGGTATGGTTCAGCAACTACCCCCATCGAATTATTTGGTCCCACCCGTTATCAATGGGATCAGGGATACTTTCAGCAAGAAATATATCGAAGAGTCAGTGCTGGGCTAGCCGAAAATCAAAGTTTATCAGAAGCTTGGTCTAAAATTCCTGAAAAATTAGCTTTTTATGATTACATCGGAAATAATCCTGCGAAAGGGGGATTATTCAGAGCGGGTTCAATGGATAACGGGGATGGAATAGCTGTCGGGTGGTTAGGACACCCTATCTTTAGAGATAAAGAAGGGCGTGAACTTTTTGTACGTCGTATGCCTACCTTTTTTGAAACATTTCCAGTTGTTTTGGTAGACGGAGATGGAATTGTTAGAGCCGACGTGCCTTTTCGAAGGGCAGAATCGAAGTATAGTGTCGAACAAGTAGGTGTAACTGTTGAGTTCTATGGTGGCGAACTGAATGGAGTGAGTTATAGTGATCCTGCTACTGTGAAAAAATATGCTAGACGTGCTCAATTGGGTGAAATTTTTGAATTAGATCGTGCTACTTTGAAATCCGATGGTGTTTTTCGTAGCAGTCCAAGGGGCTGGTTTACTTTTGGACACGCTTCATTTGCTCTGCTTTTCTTCTTCGGACACATTTGGCATGGTGCTAGAACCTTGTTCAGAGATGTTTTTGCTGGTATTGACCCGGATTTGGATGCTCAAGTGGAATTTGGAGTATTCCAAAAACTTGGAGATCCAACTACAAGAAGACAAGTAGTCTGA

>psbN

ATGGAAACAGCAACCCTAGTCGCCATCTCTATATCTGGGTTACTTGTAAGTTTTACTGGGTACGCCTTATATACTGCTTTTGGGCAACCCTCTCAACAACTAAGAGATCCATTCGAGGAACACGGGGACTAG

>psbH

ATGGCTCCACAAACAGTTGAGGGTAGTTCTAGAGCTCGTCCAAAAATGACTTCTGCAGGGGGGTTATTGAAACCTTTGAATTCGGAATATGGTAAAGTAGCTCCTGGATGGGGAACTGCTCCTTTGATGGGTATCGCAATGGCTCTATTTGCGATATTTCTGTCTATTATTTTGGAGATTTATAATTCGTCCGTTTTACTGGACGGAATTTCAATGAATTAG

>petB

ATGAGTAAAGTCTACGATTGGTTCGAAGAACGTCTCGAGATTCAGGCGATTGCAGACGATATAACTAGTAAATACGTTCCTCCTCATGTCAACATATTTTATTGTCTAGGAGGAATTACGCTTACTTGTTTTTTAGTACAAGTAGCTACAGGGTTTGCTATGACTTTTTACTACCGTCCGACCGTTACTGAGGCTTTTGCTTCTGTTCAATACATAATGACGGAAGCTAACTTTGGTTGGTTAATCCGATCAGTTCATCGATGGTCGGCAAGTATGATGGTCCTAATGATAATCCTGCACGTATTTCGTGTGTATCTCACTGGCGGTTTTAAAAAACCTCGCGAATTGACTTGGGTTACAGGCGTGGTTCTGGCTGTATTGACCGCATCCTTTGGTGTAACTGGTTATTCTTTACCTTGGGACCAAATTGGGTATTGGGCAGTCAAAATTGTAACAGGCGTGCCAGAAGCAATTCCGGTAATAGGATCGCCTTTGGTAGAGTTATTACGCGGAAGTGCTAGTGTGGGACAGTCCACTTTGACTCGTTTTTATAGTTTACACACTTTTGTATTACCTCTTCTTACTGCCGTATTTATGTTAATGCATTTCCTAATGATACGTAAACAAGGTATTTCTGGCCCTTTATAA

>petD

ATGGGAGTAACAAAAAAACCTGACTTGAATGATCCTGTATTAAGGGCTAAGTTGGCTAAAGGTATGGGTCATAATTATTATGGCGAACCCGCATGGCCCAACGATCTTTTATATATTTTTCCAGTAGTAATTCTAGGTACTATTGCATGTAACGTAGGCTTAGCGGTTCTAGAGCCATCAATGATTGGTGAACCCGCGGATCCATTTGCAACTCCTTTGGAAATATTACCCGAATGGTATTTCTTTCCCGTATTTCAAATACTTCGTACAGTACCCAATAAGCTGTTGGGTGTTCTTTTAATGGTTTCAGTACCTACGGGATTATTAACAGTACCTTTTTTGGAAAATGTTAATAAATTCCAAAATCCATTTCGCCGCCCAGTAGCGACAACCGTCTTTTTGATTGGTACCGCAGTGGCCTTGAGCTTGGGTATTGGAGCAACATTACCTATTGAAAAATCCCTAACTTTAGGTCTTTTTTAA

>rpoA

ATGGTTCGAGAGAAAGTAAAAGTATCTACTCGGGCACTACAGTGGAAGTGTGTTGAATCAAGAGCAGACAGTAAGCGTCTTTATTATGGACGCTTTATTTTGTCTCCACTTATGAAAGGTCAAGCCGACACAATAGGCATTGCGATGCGAAGAGTTTTGCTTGGAGAAATAGAAGGAACATGTATTACACGCGCAAAATCTGAGAAAATCCCACATGAATATTCTACCATAGTGGGTATTCAAGAATCGGTACATGAAATTTTAATGAATTTGAAAGAAATTGTATTGAGAAGTAATCTTTATGGAACTTGTGACGCGCTTATTTGTGTCAAAGGTCCGGGAGATGTAACTGCTCAAGACATCCTCTTGCCACCTTCTGTAGAAATCGTTGATAAGACGCAGCACATAGCTAGCCTAACAGAACCAATTGATTTGTGTATTGGATTACAAATCGAGAGGAGTCGAGGATATAATATAAAAACGCCAAATAATTTTCAAGACGGAAATTGTTATCCTATAGACGCTGCATTCATGCCTGTTCGAAATGCGAATCATAGTATTCAGTCTTATGGGAATGGCAATGAAAAACAAGAGATCCTTTTTCTAGAAATATGGACAAACGGGAGTTTAACTCCTAAAGAAGCACTTCATGAAGCCTCCCGGAGTTTGATTGATTTATTTATTCCCTTTCTCCAGGCAGCAGACGAAAACTTACATTTAGAGAACAATCAATACAAGGTTACTTTACCTTTTTTTACTTTTCATGATAGATTGGCTAAACTAACGAAAAAGAAAAAAGAAATCGCATTGAAATCGATTTTTATTGACCAATCAGAATTGTCTCCCAGGATCTATAATTGTCTCAAAAAGTCCAATATACATACATTATTCGAGCTTTTGAATAAGAGTCAAGAAGACCTTATGAAAATTGAACACTTTCGCCTAGAAGATGTAAAGCAGATAATGGGTATTCTAGAAAATAAATAG

>rps11

ATGGCAAAATCTACACCAAGAAGTGGTTCACGTAGGGCTGGACGGATGGGTTCGCGTAAAAGTGGACGTCGAATACCAAAGGGCGTTATTCATGTTCAAGCAAGTTTCAACAACACCATTGTGACTGTTACGGATGTACGGGGTCGGGTAATTTCTTGGTCCTCGGCCGGTACTTGTGGATTCAGGGGTACAAGAAGAGGTACGCCCTTTGCTGCTCAAACCGCAGCAGGAAGTGCTATTCGAGCAGTAGCGGATCAAGGTATGCAACGAGCAGAAGTCATGATAAAGGGTCCTGGTCTCGGAAGAGATGCAGCATTACGAGCTATTCGTAGAAGCGGTATCCTTTTAAATTTCGTACGGGATGTAACCCCTATGCCACATAATGGTTGCAGACCCCCTAAAAAAAGACGGGTGTAG

>rpl36

ATGAAAATAAGGGCTTCCGTTCGTAAAATTTGTGAAAAATGTCGACTGATCCGCAGGAGGGGACGGATTATAGTAATTTGTTCCAACCCGAGACATAAACAAAGACAAGGATAA

>rps8

ATGGGCAAAGACACTATTGCTGACATAATAACTTCTATACGAAATGCTGACATGAATCGAAAGGGAACAGTTCGAATAGCATCTACTAGCATCACCGAAAACATTGTTAAAATACTTTTGCGAGAGGGTTTTCTAGAAAACGTAAGGAAACTCGTGGAAAACAAAAAAGAGTTTTTGGTTTTAACCCTACGACATAGAAGGAATAGGAAAGGGCCATATAGACCCATTTTAAATTTAAAACGAATCAGTCGACCCGGTCTACGAATCTATTTTAACTATCAACGAATTCCTAGAATTTTAGATGGGATGGGGATTGTAATTCTCTCTACTTCTCAGGGTATAATGACAGACCGAGCGGCTCGACTAGAAAGAATCGGCGGAGAGGTTTTGTGTTATATATGGTAA

>rpl14

ATGATTCAACCTCAAACCCATTTGAATGTAGCGGACAATAGCGGTGCCCGAGAATTGATGTGTATTCGAATCATAGGAGCCAGTAATCGTAGATATGCTCATATTGGTGACGTTATTGTTGCTGTGATCAAGGAAGCAGTACCAAATACGCCTCTAGAAAGATCAGAAGTGATCAGAGCTGTAATTGTACGTACTTGTAAAGAACTCAGACGTGATAACGGTATGATAATACGTTATGATGACAATGCTGCAGTTGTCATTGATCAAGAAGGAAATCCAAAGGGAACTCGAGTTTTTGGTGCGATCGCCCGAGAATTGAGACAGTTGAATTTTACTAAAATCGTTTCATTAGCACCTGAAGTATTATAA

>rpl16

ATGCTTAGTAACCCCAAAAGAACAAGATTCCGTAAACAACATAGAGGAAGAATGAAAGGAATATCTTATCGAGGTAATCATATTTGTTTCGGCAGATATGCTCTTCAAGCACTTGAACCCGCTTGGATCACATCTAGACAAATCGAAGCAGGGCGCCGAGCAATGACACGAAATGTACGGCGTGGCGGAAAAATATGGGTACGTATATTTCCAGACAAACCAGTTACAGTAAGACCCACGGAAACCCGTATGGGGTCCGGGAAAGGATCCCCCGAATATTGGGTAGCCATCGTTAAACCGGGTAGAATACTTTATGAAATGAGTGGAGTCGCTGAAAATATCGCTCGAAAGGCTATTTCAATAGCGGCGTCAAAAATGCCTATAAGAACTAAATTCATTATTTCTGGATAG

>rps3

ATGGGACAAAAAATAAATCCACTTGGTTTCCGACTTGGTACAACACAAAGTCATCATTCGCTTTGGTTTGCAAAACCAAAAAGTTATTGCGAAGGTCTACAAGAGGATCAAAAAATACGAAACGTTATTAAGAATTATGTACAAAAAAATATGAGAATATCCTCTGGTGTTGAGGGAATTGCCCGGATAGAGATTCAAAAAAGAATTGATCTAATTCAAGTCATAATCTATATAGGATTCCCAAAATTATTACTAGAAAATAGACCGCGAAGAATTGAAGAATTACAGATGAATGTACAAAAAGAACTTAATTGTGTGAACCGAAAAATAAACATTGCTATTACAAGAATTACAAATCCTTATGGACACCCCAATATTCTTGCCGAATTTATAGCGGGCCAATTAAAAAATAGAGTTTCTTTTCGCAAAGCAATGAAAAAAGCTATTGAATTAACTGAACAGGCAGATACAAAAGGAATTCAAGTCCAAATTGCAGGGCGTCTTGACGGAAAAGAAATTGCGCGCGCCGAATGGATCAGAGAAGGTAGAGTTCCTCTACAAACCATTGGAGCTAAAATTGATTATTGTTCCTATACAGTTCGAACTATATACGGGGTATTAGGAATCAAAATTTGGATATTTGTAGACGAAGAAAAATAA

>ndhF

ATGGAACACCCATATCAATATTCCTGGATCATACCTTTAGTTCCACTTCCAGTCCCTATGTTAATAGGGGTGGGACTTCTACTTTTTCCGATCGCAACAAAACATCTTCGCCGTATGTGGGCTTTTCTTAGTATTTTATTGTTAAGTATAGTTATGATTTTTTCGATTGATCTATCTATTGAGCAAATAGATCGAACTTATATCTATCAATCCCTAAGGTCTTGGACCATCAATAATGATTTTTCTTTCGAGTTCGGATACTTTATTGATCCACTTACTTCTATTATGTCAATATTAATCACTACAGTTGGAATTCAGGTTCTTATTTATAGTGACAATTATATGTCTTATGATCAAGGATATTTGAGATTTTTTGCTTATATGAGTTTTTTCAATGCTTCAATGTTAGGATTAGTTACAAGTTCGAATTTCATACAAATTTATATTTTTTGGGAATTGGTTGGAATGTGCTCTTATCTATTAATAGGATTTTGGTTCACACGACCTATTGCGGCAGGCGCTTGTCAAAAAGCATTTGTAACTAATCGTGTAGGGGATTTTGGATTATTATTAGGAATCCTAGGTCTTTATTGGATAACGGGTAGTTTCGAATTTCGGGATTTGTTCGAAATATTGAATAACTTGATTTATAATAATGAGGTTAACCTTTTATTTGTTACTTTGTGTGCATTTCTATTATTTGCCGGCCCGGTTGCTAAATCCGCGCAATTCCCTCTTCATGTATGGTTACCCGATGCCATGGAAGGGCCTACTCCTATTTCGGCTCTTATCCATGCTGCTACTATGGTAGCGGCGGGAATTTTTCTTGTAGCTCGCCTTCTTCCGCTTTTCATAGTCATACCGTACATAATGAATCTAATATCTTTGTTAGGTATAATAACAGTATTTTTAGGAGCTACTTTAGCTCTTGCTCAACAAGATATTAAGAGAGGTTTAGCTTATTCTACAATGTCTCAATTGGGTTATATGATGTTAGCTCTAGGGATGGGGTCTTATCGAGCCGCTTTATTTCATTTGATTACTCACGCTTATTCCAAAGCCTTGTTGTTTTTAGGATCCGGATCAGTTATTCATTCAATGGAAGCCATTGTTGGATATTTTCCAGATAAAAGCCAGAATATGGTTCTTATGGGTGGGTTAAGAAAGCACGTGCCAATTACAAAAACCGCTTTTTTATTGGGTACCCTTTCTCTTTGTGGTATTCCGCCTCTCGCTTGTTTTTGGTCCAAAGATGAAATTCTTAATGATAGTTGGTTGTATTCGCCGAATTTCGCAATAATTGCTTTTTTCACAGCCGGATTAACCGCATTTTATATGTTTCGAATTTATTTACTTACTTTTGAAGGACCTTTCAACTTTTGCTTTCAAAATTACAGTGGCAAAAAAAGCAATTCCTTCTATTCAATATCTCTATGGGGTAAAGAAGAACCAAAACCAATAAAAAAAAAATTTCATTTAGTTGCTTTATTAACAATGAATAATAATGAAAGGGCCTCTTTTTTTTCGCAGAAGGCTCATCGAATTGATAGGAATGTAACAAATACGCCTTTTCTGACTATTTTTCCTTTTGGCGCTACCAAGACTTTTTGTTATCCTCACGAATCAGACAATACTATGTTATTTGTTATGCTTGTATTAGTCCTATTTCCTTTGTTTGTTGGAGCTATAGGAATTCCTTTGACTCAAGAAGGAATCGATTCGGATATTTTATCAAAATTGTTAACTCCGTCTATAAATCTTTTACATCAAAATTCAACTCATTTTGTTGATTGGTATGAAGTTTTGAAAAATCCAACCCTTTCCGTCAGTATAACGTATTTCGGAATCCTTCTAGCCTACTTTTTCTATAAACCCTTTTATTCATCTTTACACAATTGGAACATACTCAATTTATTTGCTAAAAGAGGACCTAAGAGAATTCTTTGGGACAAAATACTCAATTTTCTATATGATTGGTCATATAATCGTGCTTATATAGATGCCTTTTACAAAAGATCTTTAATGGAAGGGATAAGAGGATTAGCGGAACTAACGCATTTGTTCGACAGACGAGTAATTGATGGAATTGCAAATGGGGTCGGTATTACAAGTTTTTTTGTGGGGGAAGGTATAAAATATTTAGGGGGAAGTCGCATCTCTTTTTATCTCTTATTATATTTATTTTCAATCTTAATCTTTTTAATAAGTTCCTCCTTTTAA

>rpl32

ATGGCAGTTCCAAAAAAACGTACTTCTATATTAAAAAAACGTATTCGTAAGAATATTTGGAAAAAAAGGGGGTATTGGGCAGCGTTGAAGGCTTTTTCGTTAGCGAAATCCCTTTCTACTGGGAATTCAAAAAGCTTTTTTGTACAACAAATAAATAAGAAAACGTTGGAATAA

>ccsA

ATGATCTTTTCAACTTTAGAGCATATATTAACGCATATATCCTTTTCGGTCGTTTCAATTGGAATTACAATTTTTTTACTAACCTTATTAGTCGATGAAATCAGAGGACTATATGATTCATCAGAAAAGGGGATGATAGCTACCGCTTTCTGTCTAACAGGATTATTAATCACTCGTTGGATTTACTCGAGACATTTCCCATTAAGTAATTTATATGAATCATTAATCTTTCTTTCATGGAGTTTCTCCATTATTCATAGGATTTTCGATTTTCAAAATAATAAAAATCTTTTAAGTGCTATAACGGCACCAAGTGCTATTTTTACCCAAGGTTTTGCTACTTCGGGTTTTTTAACCAAAATGCATCAATCCGGAATATTAGTACCCGCTCTCCAAGTCCAGTGGTTAATGATGCACGTAAGTATGATGGTATTGGGCTATGCAGCTCTTGTATGTGGATCCTTATTATCCACGGCTCTTCTAGTCATTACATTTCGAAAAGTGATAAGGTTTTTTTTGAAAAGAAACAATTTTTTAAATGTAAAAGAGTCGTTTTGCTTCGGTAAAATTCAATACATGAACGAAAAAAGGAATGTTTTACTAAATACTTTTTCCGCTAGAAATTATTACAGGTATCAAGTGATTCAACAATTGGATCGCTGGAGTTATCGTATTATTAGTTTCGGATTTATCTTTTTAACCATAGGGATTCTTTCGGGAGCAGTATGGGCTAATGAGGCGTGGGGGTCTTATTGGAATTGGGATCCAAAAGAAACTTGGGCATTTATTACTTGGACTATATTCGGGATTTATTTACATACTCGAACAAATACAAATTTGGAAGGTGTAAATTCCGCGATTGTCGCTTCTACGGGCTTTCTTATAATTTGGGTATGCTATTTTGGAGTAAATCTATTAGGAATAGGATTACATAGTTATGGTTCATTTAATTAA

>psaC

ATGTCACATTCAGTAAAGATTTATGATACATGTATAGGGTGTACTCAATGTGTCCGAGCTTGCCCCACAGATGTATTAGAAATGATACCTTGGGACGGATGTAAAGCAAAGCAAATTGCTTCTGCTCCAAGAACAGAGGACTGTGTTGGTTGTAAGCGATGCGAATCCGCCTGTCCAACGGATTTCTTGAGTGTTCGGGTTTATTTATGGCATGAAACAACTCGAAGCATGGGTCTAGCTTATTGA

>ndhE

ATGATGCTCGAGCATGTACTTGTTTTGAGTGCCTATTTATTTTCTATTGGTATCTATGGATTGATTACGAGCCGAAATATGGTTCGGGCCCTGATGTGTCTTGAACTTATACTAAATGCAGTTAATATCAATTTCGTAACATTCTCTGATTTTTTTGATAGTCGACAATTAAAAGGAGATATTTTCTCAATTTTTGTTATAGCTATTGCAGCCGCTGAAGCAGCTATCGGATCAGCTATTGTTTCGTCAATTTATCGTAACAGAAAATCGACTCGTATCAATCAATCGACTTTGTTGAATAAGTAG

>ndhG

ATGGATTTGCCTGGACCAATACATGATTTTCTTTTAGTTTTTCTGGGATCAGGTCTTATATTAGGAGGTCTGGGAGTGGTATTATTTACCAACCCAATTTATTCTGCCTTTTCCTTGGGATTGGTTCTTGTTTGTATATCCCTATTCTATATTCTATCAAATTCCCATTTTGTAGCTGCCGCGCAGCTCCTTATTTACGTGGGAGCTGTAAATGTTTTGATCATATTTGCTGTAATGTTCATGAATGGTTCAGACTATTCCAAAGATTTTCATTTGAATCTTTGGACTGTTGGTGATGGGCTTACTTCCCTGGTTTGTACAAGTATTTTTTTTTCGCTAATCGCTACTATTCTAGATACGTCGTGGTATGGGATTATTTGGACTACACGACCCAACCAGATTATCGAACAAGATTTGATAAGTAATAGTCAACAAATTGGAATTCATTTATCAACAGACTTTTTTCTTCCATTTGAACTCGTTTCAATAATTCTTTTAGTTGCTTTGATAGGTGCAATTGCCGTGGCTCGTCAGTAA

>ndhI

ATGTTCCCTATGGTAACTGGTTTCATGAATTATGGTCAACAAACAATACGAGCTGCAAGGTACATTGGTCAAAGTTTCATGATTACTTTATCCCAAGCAAATCGTTTACCTGTAACTATTCAATATCCTTATGAAAAATTAATCACATCGGAGCGTTTTCGCGGTAGAATCCATTTTGAATTTGATAAATGTATTGCTTGTGAAGTATGCGTTCGCGTATGTCCTATAGATCTGCCTGTTGTTGATTGGAAATTTGAAACAGATATTCGAAAGAAACGATTGCTTAATTACAGTATTGATTTTGGAATTTGTATTTTTTGTGGTAACTGCGTTGAGTATTGTCCAACAAATTGTTTATCAATGACTGAAGAATATGAACTTGCTACTTACGACCGTCACGAATTGAATTATAATCAAATTGCTTTAGGTCGTTTACCAATGTCAGTGATTGACGATTTTACAATTCGAACAGTCTTGAATTCGCCTCAAAGAAAAAACGGCTAA

>ndhA

ATGATAATTGATACAACAGAAGTACAAGATATCAATTCTTTTTCCAGATTGGAATTCCTACAAGAGGTCTATGGGATCGTGTGGGCTCTTGCCCCTATTTCGACTCCTGTAGTGGCAATCACAATAGGTGTCCTAGTAATTGTGTGGTTAGAAAGAGAAATATCTGCAGGAATACAACAACGTATTGGGCCTGAATACGCCAGTCCCTTGGGACTTCTTCAAGCTTTAGCAGATGGGACAAAACTACTTTTCAAAGAAAACCTTCTTCCATCTAGAGGAAATAGTAGTTTATTCAGTATTGGACCATCTATAGCAGTCATAGCAATTCTACTAAGTTATTCAGTAATTCCTTTTAGTTATAACTTTGTTTTAGCTGACCTCAATATCGGTATTTTTTTATGGATTGCCATTTCAAGTATTGCCCCTATTGGACTTCTTATGTCAGGATATGGATCAAATAATAAATATTCTTTTTTAGGTGGTTTGCGAGCTGCTGCTCAATCGATTAGTTATGAAATACCATTAACTTTATGTGTTTTATCAATATCTCTATTATCTAACAGTTCAAGTACAGTTGATATAGTTGGGGCGCAATCAAAATATGGTTTTTGGGGGTGGAATTTGTGGCGTCAACCTGTAGGGTTTATCGTTTTTCTAATTTCTTCCCTAGCGGAATGCGAGAGATTACCTTTTGATTTACCAGAAGCAGAAGAAGAACTAGTAGCAGGTTATCAAACCGAATATTCAGGAATAATTTTTGGTTTATTTTACGTTGCTTCCTATCTAAATCTATTAGTTTCCTCATTATTTGTAACAGTTCTTTACTTGGGGGGTTGGAATCTTTCCATTCCACACATATTTGTTCCTGAGCTATTTGAAATAAATAAAGCGGATGGAATCTTTGGAACGACAATTGGTATCTTTATTACATTAGCTAAAACTTATTTGTTCTTGTTCGTTCCGATTACAACAAGATGGACTTTACCGAGACTAAGAATGGACCAACTATTAAATCTTGGCTGGAAATTTCTTTTACCTATTTCTCTCGGTAATCTATTATTAACAACTTCTTCCCAACTCCTTTCGCTATAA

>ndhH

ATGAGTATACTAGCTACAGAAAAAGAATTTATGATAGTCAATATGGGACCTCACCACCCATCAATGCACGGTGTTCTTCGTCTCATCGTTACTCTAGATGGTGAAGATGTTATTGACTGTGAACCAATATTGGGTTATTTACACCGAGGGATGGAAAAAATTGCGGAAAACCGAACAATTATACAATATCTGCCTTATGTAACCCGTTGGGATTATTTAGCTACTATGTTCACTGAAGCAATAACTGTAAACGGACCCGAACTGTTGGGAAATATTCAAGTACCCAAAAGAGCCAGCTATATCAGAGTAATTATGTTGGAGTTGAGTCGTATAGCTTCCCATCTGTTATGGCTTGGCCCTTTTATGGCAGATATTGGTGCACAGACTCCTTTCTTCTATATTTTCAGAGAAAGAGAATTGGTCTATGATCTGTTCGAAGCTGCCACCGGTATGAGGATGATGCATAATTATTTTCGTATCGGAGGAATAGCGGCTGATTTACCTCATGGTTGGATAGATAAATGTTTGGATTTCTGCGATTATTTTTTAACGGGGGTTGCTGAATATCAAAAACTTATTACACGAAATCCTATTTTTTTAGAACGAGTTGAAGGAGTAGGCATTATTGGTGGAGAAGAAGCAATAAATTGGGGTTTATCCGGACCAATGCTACGAGCGTCTGGAATAGAATGGGATCTTCGTAAAGTTGATCATTATGAGTGTTATGACGAATTTGATTGGGAAGTCCAGTGGCAAAAAGAAGGAGATTCATTAGCTCGTTATTTAGTCCGAATCGGTGAAATGACGGAATCTATAAAGATTATTCAACAGGCTTTAGAAGGAATTCCGGGAGGACCCTATGAAAATTTAGAAATCCGATGTTTTGATAGAGAAAGCGATCCAGAATGGAATGATTTTGAAGATCGATTCATTAGTAAAAAGCCTTCTCCCACCTTTGAATTGACGAAACAAGAACTTTATGTGAGAGTAGAAGCCCCAAAAGGAGAATTGGGAATTTTTCTGATAGGAGATCAAAGTGGTTTTCCTTGGAGATGGAAAATTCGCCCACCGGGTTTTATCAATTTGCAAATTCTTCCTCAGTTAGTTAAAAGAATGAAATTGGCTGATATTATGACAATATTAGGTAGTATAGATATCATTATGGGGGAAGTTGATCGTTGA

>rps15

ATGATAAAAAATTCATTCGTCTCAGTTATGGTTCAAGAAAAAAAAGAAGAAAACTGTGGATCGGTTGAATTTCAAGTATTCCGTTTCACTAATAAGATACGGAGACTTACTTCACATTTAGAATTGCACAGAAAAGACTATTTATCTCAAAGGGGTCTACGAAAAATTTTGGAAAAACGCCAACGTCTACTAGCTTATTTGTCAAAGAAAAATAGAGTACGTTATAAGGAATTAATTAGTAAGTTCAATATTCGGGAGTCAAAAAATCGTTAA

>ycf1

ATGATTTTTCAATCTTTTATACTAGGTAATCTAGTATCCTTATGCATGAAGATAATCAATTCGGTCGTTGTGGTCGGACTCTATTATGGATTTATGACCACATTCTCCATAGGGCCCTCTTATCTCTTCCTTCTCCGAGCTCGGGTTATGGAAGAAGGAGAAGAAGGAACCGAGAAGAAGGTATCAGCAACAACTGGTTTTATTGCGGGACAGCTCATGATGTTCATATCGATCTATTATGCGCCTCTGCATCTAGCATTGGGTAGACCTCATACAATAACTGTCCTAGCTCTACCGTATCTTTTGTTTCATTTCTTCTGGAACAATCCCAAACACTTTTTTGATTATGGATCTACTACCAGAAATTCAATGCGTAATCTTAGCATTCAATGTGTATTCCTGAATAATCTCATTTTTCAATTATTCAACCATTTCATTTTACCAAGTTCAATGTTAGCCAGATTAGTCAACATTTATATGTTTCGATGCAACAACAAGATGTTATTTGTAACAAGTAGTTTTGTTGGTTGGTTAATTGGTCACATTTTATTCATGAAATGGGTTGGATTGGTATTAGTTTGGATACAGCAAAAGAATTCTATTAGGTCTAATGTACTTATTCGATTTAATAAGTACCTTGTGTCAGAATTGAGAAATTCTATGGCTCGAATCTTTAGTATTCTCTTATTTATTACCTGTATCTACTATTTAGGCAGAATACCCTCACCCATTTTTACTAAGAAACTGAAAGTGAAAGAAACCTCAGAAACGGAAGAAAGAGATGTAGAAATAGAAACAACTTTCGAAAGGAGGGGGACTAAACAGGAACAAGAGGTATCCGCCGAAGAAGATCCTTCTCCTTCCCTTTTTTCGGAAGAAAAGGAGGATCCGGACAAAATCGAGGAAACGGAAGAGATCCGAGTGAATGGAAAGGAAAAAAAAAAAACAAAGCATGAATTCCACTTTCGCTTTAAAGAGACATGCGATAAAAATAGCCCAGTTTATGAAACTTCTTATCTGGATGGGAATCAAGAAAATGCGAAATTCCAAATATTTAAATTATTTAAAGAAAAAGAAGAGAAATATTTATTAAGGTTTGAAAAACCTCTTGTGACTCTTCTTTTCGACTATAAACGATGGACTCGGCCAGTTCGATATAAAAAAAATAATCGATTTGAAAATGCTGTTCGAAATGAAATGTCACACTATTTTTTTTATACATATCGAAGTGATGGAAAAGAAAGGATCTCTTTTACGTATCCAGTAAGTCTGTCAACTTTTTTGGAAATGATAAAAAAAAAAATTTCTTTTTTCACAACAGAAAAACTATCCTCCGATGAATTTTATACACATTGGAATTACACTAATGACCAAAAAAGGAAGAACTTAACCAGGGAGTTTAGAAATAGAATCAAAGTTTTAGATAAAAGATCTCTTATTCTGGATATACTCGAAAAAAGGACTCAATTGTGCAATGATAAGACTAAAAAAAAATACTTACCTAAAATATATGATCCTTTATTACATGGACCTTATCGTGGAAGAATGAAAAATTTATTTTCACCCGCAATCCTAAATAAAACTTATATCAAAAATAAGATAGGAACGCTTTGGATAAATAATATTCACAATATCATTCTTATTAATGATTATGACGAATTTGAACAGACAATAGACCGAGTTAAGCCAAAACCATTTTCAAGAGAAGAAGTAGGGTCTTTATTTACAGAATACGAACGAGAACAAATCGATTCAGAAGAACGAATCCAAAATTTAAAATTTTTATTCGATGCAGTTATAACCGATCCCAATGATCAAAAAATTAGAAAAAAATCGATAAAAGAAATTAGTAAAAGAGTTCCCCGGTGGTCATACAAATTAATCGATAATTTAGACCAAGAGCTGGGAGAATACGACGAAAATGTAAGAGGGGAGCATGCATTTCGTTCACGAAAAGCCAAACGTTTGGTGGTTTCTGTTGATGACCAGACAGACGAGGATGTGACTTTGCCACGTTATTTGGAACAATCAGATTTTCGTCGATATATAATCAAAGGTTCTATGCGCGCACAAAGACGTAAAACCGTTATTTGGAAACCGGTTCAAGCAAATGCCCATTCCCCTCTTTTTTTGGACAGAATAGACAAACCCTTTTATTTGTCTTTTGATATTTCCGGGCTGATCAAAGTAATTTTTAGAAATTGGATGTGGAAAAATAAGGACCAAAAACTTTCTGATTATACAAACGAAAAGACAAGAAAATTGGATAAAAAAACAAAAAAGAAGCCCAAAAAAGAAGGATACACAAGACAAGACAAGGTACGTATAAAACAAGCAGAAGGCTGGGATAAGAGTTTCCGTACTCGAGTACTAAGAAGCTCTATGTTAGTAATCCAATCGATTCTTAGAAAATATATTATATTACCTTCATTGATAATAGTTAAAAACTTGGTTCGCATACTATTCTTCCAAGATCCCGAGTGGTCCGAGGATTTTAAGGATTGGAGTCGGGAAATTTATATTAAATGCACTTATAGTGGTGTTAGTTTATCTGAAACAGAATTTCCGGAAAACTGGTTAACAGAAGGTATTCAGATAAAGATACTATTCCCTTTTCGCCTGAAACCCTGGCACAGATCTAAGATTCAATCCCCTCATAAAGATGCAAAAAGTGAAGCTGATTTTTGTTTTTTAACAGCTTTGGGATTGGAAACTGAAATGCCCTTTGGTTCTCCCCGAAAACGACGTTCGTTTTTTGAACCCATTTTTAAGGAACTCAAAAAAAAAATTCTAAAATTGAAAACTAAGTCTTTTATAGTTTTAGGGGCTTTCAAAGAAGGAAAAATCAAAGAACTTTCAAAAATAAACTTGAGAGAAATCGATGAATCGGGTGAAACTCAAAAAAATTCGATACTCAGTAATCAGAAGATTCACGAATCGTCTATTGAAATTCCATCTATGGATTGGCCAAATTTATCCCGGACCGAAAAAAAAATGAAAGATCTGACTAATAGAACAAGCAGAATACGAAATCAAATATATAAAATTACAAAAGAAAATAAAAAGGGACCGCTAACTCAAGAAACAAATATTAGTTCGAACAAAACATTAGACTCATCAAAAAAGATTTGGCAGATATTCAAAAAAAGAAATACTCGATTAACCCGTAAATCCTATTTTTATCTAAATTTTTTTATTGAAAAGCTTTACATAAATTTTTTTCTATTTACCCTTACTATTCCAAGAATCAATGCAAAACCTTTTAGTGAATCAACAAGAAAAAAAATTAAAATGATTGAGAAAAACATCCACAATAATGAAGAAAATCCGGAAATAATTAATAAAACAAATCAAAATAGAATTCACTTTATTTCGACTGTCAAAAAATCACTTTCGAAGATTAGTAATAAGAATTTAAAGATTTCTTGTAATTTATCGTCTTTTTCACAATCCCAAGCATATGTATTTTACAAATTGTTACAAGCCCCAATTTTTAACTTTTCTAATTTAAGACCTGTTCTTCAATATCACGGAACATCTCTATTTCTTAAGAATGAAATAAAGGATTTTTTTGAAAAACACGGAATATTTAATTACCAATTAAGACATAAACCTTTTTGGAATTTTGGAAGGAATCCACGGAAAAACAGGTTAAGCGGTCATTATCAATATGATTTCTCTCGGATTAAATGGGCTAGATTAGTACCACAAGAATGGCGAAATAGGGCCAATCAACACTGTATGGCTCAAAATAAAGATTTAACTAAAAGAGATTCATATGAAAAAAACGGATTAACTCATTGCGAAAAACAACATTTTTTTGAAGCAGACTTATTACGGAATCAAAAATCTAATTTTAAAAAACACTATAGATATGATCTTTTATCATATAAATCGATTAATTATGAAGATAAGAAAGACTCATATATTGATAGATTACTAGGCCAAGTAAATAATAAAGAAGAGTATTATTATAATTACAATATAAAGAAAGCAAAATTATTTGCTATGCTGGGAGGTATCCTTATCAATAATTATCTAGGAGAAGATGATATTATGGATATGGAAAAATTCTTGTATAGAAAATATTTTGATTGGAGAATTCTTAATTTTTGTCTTAGAAATAAGATCAATATTGAAGCCTGGGTTGATATGGATACTGGTATCAGCAGTAATCAAAATACTAAGATTGGGTCCGATAATTATAAAAAAATTGATGCAATTAATAAAAGAGGCCCATTTTATCTTACAATTCATCAAGATGAAGAAATTAACCCATCCAACCAAAAAAGAACACTTTTTGATTGGATGGGAATGAATGAAGAAATACTAAGTTGTCCTATATCAAACCTGGAGCCTTGGTTCTTTCCAGAATTTGTGCTACTTTTTAATGCATATAGAACGAAACCCTGGATCATACCAATTAAATTACTTCTTTTCAATTTTCATGGAAATGGTAAGAAAATTATAACCGGAAAGAACGAAGCGGATCTTTTTATATCATCCACTCAAAAAGAATATCTTGAATTATCGAATCAAAGTAAAGAAGAAAAAGAACTCGCAGACCAAGGAAATGCGGGATCAGATGCCCAAAAGCAAGTAAGTCTTGGATCAGTTCTCTCAAACCAAGAAAAAGATGTTGAAGAAAATTATACGAGATCGGACATGAAAAAACGTATAAAGAAAAAGCAATACAAGAGAGAAACAGAAGCACAGCTTGATTTCTTCCTAAAAAAATATTTGTGTTTGCAGTTAAGATGGAGAGGTACTGTTTCTTTCAAGGAAAAAATACTCAATAATATGGAAGTCTATTGTCACCTGGTTCGACTGATAAATCCTAGCGACGTTACTATAGACTCTATTCAAGGAGGAGAAATTTGTTTGGCTATTTTGATCACTAAGAAGGATTTTGCTCTTAGAGAATTGACGAAAGGGGGAATGCTTATTATCGAACCCCGTCGTTTGTCTGTAAAAAATGATGGCCAATTTTTTATATATCAAACCGTAGGTATTTCATTGGTTCATAAGAATAAGCGAAAAATTACTAAAAGATACCACGAAAAGGGCTATGTTGATAAAAAAATTTTTGATGAATTCATTGCAAAACATCAAAAAATGACTGGAAATAGAAACAAAAATCATTATGATTTGCTTGTTCCTGAAAATATTTTATTCCCTAAACGTCGTAGAGAATTAAGAACTCGAATTTGTTTCAATTCAAAGAATCAAAACGGTATGCAGAGAAATCCAGTATTTTGTACTAACGTAAAAAGCGGGGGTCACCTTTTGGATAAAAACAAAGATCTTCCTAGAGAGAAAAATCAACTAATTAAATTAAAGTTCTTTATTTGGCCCAATTCTCGATTAGAAGATTTAATTTGTATGAATCGCTATTGGTTTAATACCAATAATGGGAGTCGTTTCAGTATGGTAAGGATACATATGTATCCACGATTGAAAATTCGTTAA

>rps7

ATGTCACGTCGAGGTACTACAGAAGAAAAAGCTGCAAAATCCGATCCAATTTATCGTAATCGCTTAGTTAACATGTTGGTTAACCGTATTCTGAAACACGGAAAAAAATCATTGGCTTATCAAATTATCTATCGAGCCTTGAAAAAGATTCAACAAAAGACAGAAAAAAATCCACTATCTGTTTTACGTCAAGCAATACGTGGAGTAACTCCCGATATAGCAGTAAAAGCAAGACGTGTAGGCGGATCGACTCATCAAGTTCCCATTGAAATAGGATCCGCACAAGGAAAAGCACTTGCCGTTCGTTGGTTATTAGGGGCATCCCGAAAACGTCCGGGTCGAAATATGGCTTTCAAATTAAGTTCCGAATTAGTGGATGCTGCCAAAGGGAGTGGCGATGCCATACGCAAAAAGGAAGAGACTCATAGAATGGCAGAGGCAAATAGAGCTTTTGCACATTTTCGTTAA

>ndhB

ATGATCTGGCATGTACAGAATGAAAACTTCATTCTCGATTCTACGAGAATTTTTATGAAAGCCTTTCATTTGCTTCTCTTCGATGGAAGTTTTATTTTCCCAGAATGTATCCTAATTTTTGGCCTAATTCTTCTTCTGATGATCGATTCAACCTCTGATCAAAAAGATATACCTTGGTTATATTTCATCTCTTCAACAAGTTTAGTAATGAGCATAGCGGCCCTATTGTTCCGATGGAGAGAAGAACCTATGATTAGCTTTTCGGGAAATTTCCAAACGAACAATTTCAACGAAATCTTTCAATTTCTTATTTTACTATGTTCAACTCTATGTATTCCTCTATCCGTGGAGTACATTGAATGTACAGAAATGGCTATAACAGAGTTTCTGTTATTCGTATTAACAGCTACTCTAGGAGGAATGTTTTTATGCGGTGCTAACGATTTAATAACTATCTTTGTAGCTCCAGAATGTTTCAGTTTATGCTCCTACCTATTATCTGGATATACCAAGAAAGACGTACGGTCTAATGAGGCTACTATGAAATATTTACTCATGGGTGGGGCAAGCTCTTCTATTCTGGTTCATGGTTTCTCTTGGCTATATGGTTCATCCGGGGGCGAGATCGAGCTTCAAGAAATAGTGAATGGTCTTATCAATACACAAATGTATAACTCCCCAGGAATTTCAATTGCGCTTATATTCATCACTGTAGGAATTGGGTTCAAGCTTTCCCTAGCCCCTTCTCATCAATGGACTCCTGACGTATACGAAGGATCTCCCACTCCAGTCGTTGCTTTTCTTTCTGTTACTTCGAAAGTAGCTGCTTCAGCTTCAGCCACTCGAATTTTCGATATTCCTTTTTATTTCTCATCAAACGAATGGCATCTTCTTCTGGAAATCCTAGCTATTCTTAGCATGATATTGGGGAATCTCATTGCTATTACTCAAACAAGCATGAAACGTATGCTTGCATATTCGTCCATAGGTCAAATCGGATATGTAATTATTGGAATAATTGTTGGAGACTCAAATGGTGGATATGCGAGCATGATAACTTATATGCTGTTCTATATCTCCATGAATCTAGGAACTTTTGCTTGCATTGTATTATTTGGTCTACGTACCGGAACTGATAACATTCGAGATTATGCAGGATTATACACAAAAGATCCTTTTTTGGCTCTCTCTTTAGCTCTATGTCTCTTATCCCTAGGAGGTCTTCCTCCACTAGCAGGTTTTTTCGGAAAACTCCATTTATTCTGGTGTGGATGGCAGGCAGGCCTATATTTCTTGGTTTCAATAGGACTCCTTACGAGCGTTGTTTCTATCTACTATTATCTAAAAATAATCAAGTTATTAATGACTGGACGAAAGCAAGAAATAACCCCTCACGTGCGAAATTATAGAGGATCCCCTTTAAGATCAAACAATTCCATCGAATTGAGTATGATTGTATGTGTGATAGCATCTACTATACCAGGAATATCAATGAACCCGATTATTGCAATTGCTCAGGATACCCTTTTTTAG

>ycf15

ATGCTACTGCTGAAACATAGAAGAATTGAAATCTTAGATCAAAACACTATGTATGGATGGTATGAACTGCTTAAACAAGAATTCTTGAACAGCGAACCACCAGAGCTATTACTAACTACATCAAAAAATTTCCATTAA

>ycf2

ATGAAAGGACATCAATTCAAATCCTGGATTTTCGAATTGAGAGAGATATTGAGAGAGATCAAGAATTCTCACTATTTCTTAGATTCATGGACCCAATTCAATTCAGTGGGATCTTTCATTCACATTTTTTTCCACCAAGAACGTTTTATAAAACTCTTGGACCCACGAATTTGGAGTATCCTACTTTCACGCAATTCACAGGGTTCAACAAGCAATCGATATTTCACGATCAAGGGTGTAGTACTATTTGTAGTAGCGATCCTTATATATCGTATTAACAATCGAAATATGGTCGAAAGAAAAAATCTCTATTTGACAGGGCTTCTTCCTATACCTATGAATTCCATTGGACCCAGAACTGATACATTGGAAGAATCTTTTGGGTCTTCCAATATCAATAGGTTGATTGTTTCGCTCCTCTATCTTCCAAAAGGAAAAAAGATCTCTGAGAGCTTTTTCCTGGATCCGAAAGAGAGTACTTGGGTTCTCCCAATAACTAAAAAGTGTATCATGCCTGAATCTAACTGGGGTTCGCGGTGGTGGAGGAACTGGCTCGGAAAAAAGAGGGATTCTAGTTGTAAGATATCTAATGAAACCGTCGCTGGAATTGAGATCTCATTCAAAGAGAAAGATATCAAATATCTGGAGTTTCTTTTTGTATATTATATGGATGATCCGATCCGCAAGGACCATGATTGGGAATTGTTTGATCGTCTTTCTCCGAGTAAGAGGCGAAACATAATCAACTTGAATTCGGGACATCTATTCGAAATCTTAGTGAAAGACTGGATTTGTTATCTCATGTTTGCTTTTCGTGAAAAAATACCAATTGAAGTGGAGGGTTTCTTCAAACAACAAGGAGCTGGGTCAACTATTCAATCAAATGATATTGAGCGTTTTTCCCATCTCTTCTTGAGAAACAAGTGGGCTATTTCTTTGCAAAATTGTGCTCAATTTCATATGTGGCAATTCCGCCAAGATCTCTTCGTTAGTTGGGGGAAGAATCCGCACGAATCGGATTTTGTGAGGAACATATCGAGAGAGAATTGGATTTGGTTAGACAATGTGTGGTTGGTAAACAAGGATCGATTTTTTAGCAAGGTACGGAATGTATCGTCAAATATTCAATATGATTCCACAAGATCTAGTTTCGTTCAAGTAACGGATTCTAGCCAATTGAAAGGATCTTCTGATCAATCCAGAGATCTTTTCGATTCCATTAGTAATGAGGATTCGGAATATCACACATTGATCAATCAAAGAGAGATTCAACAACTAAAAGAAAGATCGATTCTTTGGGATCCTTCCTTTCTTCAAACGGAACGAAGAGAGATAGAATCAGACCGATTCCCTAAATGTCTTTCTGGATATTCCTCAATGTCCCGGCTATTCACGGAACGTGAAAGGCAGATGAATAAGCATCTGCTTCCGGAAGAAATCGAAGAATTTCTTGGGAATCCTGCAAGATCCATTCGTTCTTTTTTCTCTGACAGATGGTCAGAACTTCATCTGGGTTCGAATCCTACTGAGAGGTCCACTAGAGATCAGAAATTGTTGAAGAAAGAAGAGGATGTTTCTTTTGTCCCTTCCAGGCGATCGGAAAATAAAGAAATAGTTAATATATTCAAGATAATTACGTATTTACAAAATACCGTCTCAATTCATCCTATTTCATCAGATCCGGGATGTGATATGGTTCCGAAGGATGAACTGGATATGGACAGTTCCAATAAGATTTCATTCTTAAACAAAAATCCATTTTTTGATTTATTTCATCTATTCCATGACCGGAACAGGGGGGGATACACGTTACACCATGATTTTGAATCAGAAGAGAGATTTCAAGAAATGGCAGATCTATTCACTCTATCAATAACCGAGCCGGATCTGGTGTATCATAAGGGATTTGCCTTTTCTATTTTTTCCTACGGATTGGATCAAAAACAATTCTTGAATGAGGTATTCAACTCCAGGAATGAATCGAAAAAGAAATCTTTATTGGTTCTACCTCCTATTTTTTATGAAGAGAATGAATCTTTTTATCGAAGGATCAGAAAAAAATGGGTCCGGATCTCCTGCGGGAATGATTTGGAAGATCCAAAACCAAAAAGAGTGGTATTTGCTAGCAACAACATAATGGAGGCAGTCAATCAATCTAGATTGATCCGAAATCGGATTCAAATCCAATATAGCACCTATGGGTACATAAGAAATGTATTGAATCGATTCTTTTTAATGAATAGATCCGATCGCAACTTCGAATATGGAATTCACAGGGATCAAATAGGAAATGATACTCTGAATCATAGAACTATAATGAAATATACGATCAACCAAGATTTATCGAATTTGAAAAAGAGTCAGAAGAAATGGTTCGCTCCTCTTATTTTTATTTCTCGAACCGAGAGATTCATGAATCGGGATCCTAATGCATATAGATACAAATGGTCCAATGGGAGCAAGAATTTCCAGGAGCATTTGAAACATTTCGTTTCTGAGCAGAAGAGCCGTTTTCAAGTAGTGTTCGATCGATTACGTATTAATCAATATTCGATTGATTGGTCTGAAGTTATCGACAAAAAAGATTTGTCCAAGTTGCTTTTCTTTTTGTCTAACTCACTTCCTTTTTTCTTTGTGAGTTTCGGGAATATCCCCATTCATAGGTCTGAGATCCACATCTATGAATTGAAAGGTCCGAATGATCAACTCTGCAATCAGTTGTTAGAATCAATAGGTCTTCAAATCGTTCATTTGAAAAAATTGAAACCCTTCTTATTGGATGATCATAATACTTCCCAAAAATCGAAATTCTTGATCAATGGAGGAAGAATATCACCATTTTTGTTCAATAAGATACCAAAGTGGATGATTGACTCATTCCATACTAGAAATAATCGCAGGAAATCTTTTGATAACACGGATTCCTATTTCTCAACGATATCCCACGATCAAGACAATTGGCTGAATCCTGTGAAACCATTTCATAGAAGTTCATTGATATCTTCTTTTTATAAAGCAAATCGACTTCGATTCTTGAATAATCCACATCGCTTCCGCTTCTATTGTAACAAAAGATTCACTTTTTATGTGGAAAAGGTCCGTATCAATAATTATGATTTTACGTATGGACAATTCCTCAATATCTTGTTCATTCGCAACAAAATATTTTCTTTGTGCGGCGGTAAAAAAAAACATGCTTTTTTGGAGAGAGATACTATTTCACCAATCGAGTCACAGGTATCTAACATATTCATACCTAACGATTTTCCACAAAGCGGTGACGAAAGGTATAACTTGTACAAATTTTTCCCTTTTCCAATTCGATCCGATCTATTAGTTCGTAGAGCTATTTACTCGATCGCAGCCATTTCTGGAACACCTCTAACAGAGGGACAAATAGTCAATTTTGAAAGAACTTATTGTCAACCTCTTTCAGATATGAATCTATCTGATTCAGACGAGAAGAACTTGCATCAGTATCTCAATTTCAATTCAAACATGGGTTTGATTCACACTCCATGTTCTGAGAAATATTTACCATCCGAAAAGAGGAAAAAACGGAGTCTTTGTCTAAAGAAATGCGTTGAGAAAGGGCAGATGTCTAGAACCTTTCAACGAGACAGTGCTTTTTCAACTCTCTCAAAATGGAATCGATTCCAAACATATATGCCATGGTTCCTTACTTCGACAGGGTACAAATATCTAAATTTTCTATTTTTAGATACTTTTTCAGACCTATTGCCGGTACTAAGTAGCAGTCAAAAATTTGTATCCATTTTTCATGATATTATGCATGGATCAGATATATCATGGCGAATTCTTCAGAAAAAATGGCGTCTTCCACAATGGAATCTGATAAGTGAGATTTCGAGTAAGTGTTTCCATAATCTTCTTCTGTCCGAAGAAATGATTCATCGAAATAATGAGTCACCATTGATATCGACACATCTGAGATCGCCAAATGCTCGGGAGTTCCTCTATTCAATCCTTTTCCTTCTTCTTGTTGCTGGATATCTCGTTCATACACATCTTATCTTTGTTTCCCGAGCCTATAGTGAGTTAGAGACAGAGTTCGAAAGGGTCAAATCTTTGATGATTCCATCATACATGATTGAGTTGCGAAAACTTCTGGATAGGTATCCCACATCTGAACTGAATTCTTTCTGGTTAAAGAATCTCCTTCTAGTTGCTCTGGAACAATTAGGAGATTCTCTAGAAGAAATACGGGGTTCTGCTTTTGGCGGCAACATGCTATGGGGTGGTGGTCCCGCGGATGGGGTTAAATCAATACGTTCTAAGACGAAAGATTTGAATATCAATCTCATCGATATCATCGATCTCATAAGTATCATACCAAATCCCATCAATCGAATCACTTTTTCGAGAAATACGAAACATCTAAGTCATACAAGTAAAGAGATCTATTCATTGATAAGAAAAAGAAAAAAGGGGAACGGTGATTGGATTGATGATAAAATAGAATCCTGGGTCGCGAACAGTGATTCGATTGATGATAAAGAAAGAGAATTCTTGGTTCAGTTCTCCACCTTAACGACAGAAAAAAGGATTGATCAAATTCTATTGAGTCTGACTCATAGTGATCATTTATCAAAGAATGACTCTGGTTATCAAATGATTGAACAACCGGGAACAATTTACTTACGATACTTAGTTGACATTCATAAAAAGCATTTCATGAATTATGAGTTCAATACATACTGTTTAGCAGAAAGACGGATATTCCTTGCTCATTATCAGACAATCACTTATTCACAAACTTCGTGTGGGGCTAATAGTTTTCATTTCCCATCTCATGGAAAACCCTTTTCGCTCCGCCTAGCCCTATCCCCCTCCAGGGGTATTTTAGTGATAGGTTCTATAGGAACCGGACGCTCCTATTTGGTCAAATACCTAGCGACAAACTCCTATGTTCCTTTCATTACAGTATTTCTGAACAAGTTCCTGGATAACAAACCTAAAGGTTTTCTTATTGATGATATCGATGATGATAGTGACGATATTGATGCTAGTGACGATATTGATGCTAGTGACGATATCGATCGTGACCTTGATACGGAGCTGGAGCTTCTAACTATGATGAATGCGCTAACTATCGATATGATGTCGGAAATAGGCCTATTTTATATCACCCTTCAATTCGAATTAGCAAAAGCAATGTCTCCTTGCATAATATGGATTCCAAACATTCATGATCTGGATGTGACTGAGTCGAATTACTTATCCCTCGGTCTATTAGTGAACTATCTCTCCAGGGATTGTGAAAGATGTTCCACTAGAAATATTCTTGTTATTGCTTCGACTCATATTCCCCAAAAAGTGGATCCCGCTCTAATAGCCCCGAATAAATTAAATACATGCATTAAGATACGAAGGCTTCTTATTCCACAACAACGAAAGCACTTTTTCACTCTTTCATATACTAGGGGATTTCACTTGGAAAAGAAAATGTTCCATACTAATGGATTCGGGTCCATAACCATGGGTTCCAATGCACGAGATCTTGTAGCACTTACCAATGAGGCCCTCTTGATTAGTATTACACAGAAGAAATCAATTATAGACACTAATACAATTAGATCTGCTCTTCATAGACAAACTTGGGATTTGCGATCCCAGGTAAGATCGGTTCAAGATCATGGGATCCTTTTCTATCAGATAGGAAGGGCTGTTGCACAAAATGTACTTCTAAGTAATTGCCCCATAGATCCTATATCTATCTATATGAAGAAGAAATCATGTAACGAAGGGGATTCTTATTTGTACAAATGGTACTTCGAACTTGGAACGAGCATGAAGAAATTAACGATACTTCTTTATCTTTTGAGTTGTTCTGCCGGATCGGTCGCTCAAGACCTTTGGTCTCTACCCGGACCCGATGAAAAAAACGGGATCACTTCTTCTGGACTCGTTGAGAATGATTCTGATCTAGTCCATGGCCTATTAGAAGTAGAAGGCGCTCTGGTGGGATCCTCGCGGACAGAAAAAGATTGCAGTCGGTTTGATAATGATCGAGTGACATTGCTTCTTCGGCCCGAACCAAGGAATCCCTTAGATATGATGCAAAAAGGATCTTGTTCTATCGTTAATCAGAGATTTCTCTATGAAAAATACGAATCGGAGTTTGAAGAAGGGGAAGGAGAAGGAGTCCTCGACCCGCAACAGATAGAGGAGGATTTATTCAATCACATAGTCTGGGCTCCTAGAATATGGCGCCCCTGGGGCTTTCTATTTGATTGTATCGAAAGGCCCAATGAATTGGGATTTCCCTATTGGGCCGGGTCATTTCGGGGCAAGCGGATCATTTATGATGAAAAGGATGAGCTTCAAGAGAATGATTCGGAGTTCTTGCAGAGTAGAACCATGCAGTACCAGACACGAGATAGATCTTCCAACGAACAAGGCTTTTTTCGAATAAGCCAATTTATTTGGGAACCTGCGGATCCACTCTTTTTCCTATTCAAAGATCAGCCCCTTGTCTCTGTGTTTTCACACCGAGAATTCTTTGCAGATGAAGAGATGTCAAAGGGGCTTCTTACTTCCCAACCAGATCCTCCTACATCTATATATAAACGCTGGTTTATCAAGAATACGCAAGAAAAGCACTTCGAATTGTTGATTCATCGCCAGAGATGGCTTAGAACCAATAGTTCATTATCTAATGGATTTTTCCGTTCTAATACTCCATCCGAGAGTTATCAGTATTTATCAAATCTCTTCCTATCTAACGGAACGCTATTGGATCAAATGACAAAGACATTGTTGAGAAAAAGATGGCTTTTTCCGGATGAAATGAAAATTGGATTCATGTAA

>rpl23

ATGGATGGAATCAAATATGCAGTATTTACAGACAAAAGTATTCGGTTATTGGGGAAAAATCAATATACTTCTAATGTCGAATCAGGATCAACTAGGACAGAAATAAAGCATTGGGTCGAACTCTTCTTTGGTGTCAAGGTAATAGCTATGAATAGTCATCAACTCCCCCGAAAGGGTAGAAGAATGGGACCTATTATGGCACATACAATGCATTACAGACGTATGATCATTACGCTTCAACCGGGTTATTCTATTCCACCTCTTAGAAAGAAAAGAACTTAA

>rpl2

ATGGCGATACATTTATACAAAACTTCTACCCCGAGCACACGCAATGGAGCCGTAGACAGTCAAGTGAAATCCAATCCACGAAATAATTTGATCTATGGACAGCATCGTTGTGGTAAAGGTCGTAATGCCAGAGGAATCATTACCGCAGGGCATAGAGGGGGAGGTCATAAGCGTCTATACCGTAAAATCGATTTTCGACGGAATGCAAAAGACATATATGGTAGAATCGTAACCATAGAATACGACCCTAATCGAAATGCATACATTTGTCTCATACACTATGGGGATGGTGAGAAGAGATATATTTTACATCCCAGAGGGGCTATAATTGGAGATACCATTGTTTCTGGTACAGAAGTTCCTATAAAAATGGGAAATGCCCTACCTTTGACCGATATGCCCTTAGGCACGGCCATACATAACATAGAAATCACACTTGGAAAGGGTGGACAATTAGCTAGAGCAGCGGGTGCTGTAGCGAAACTGATTGCAAAAGAGGGGAAATCGGCCACATTAAAATTACCTTCTGGGGAGGTCCGTTTGATATCCAAAAACTGCTCAGCAACAGTCGGACAAGTGGGGAATGTTGGGGTGAACCAGAAAAGTTTGGGTAGAGCCGGATCTAAATGTTGGCTAGGTAAGCGTCCTGTAGTAAGAGGAGTAGTTATGAACCCTGTAGACCACCCCCATGGGGGTGGTGAAGGAAGGGCTCCAATTGGTAGAAAAAGACCCGCAACCCCTTGGGGTTATCCTGCACTTGGAAGAAGAAGTAGAAAAAGGAATAAATATAGTGATAATTTGATTCTTCGCCGCCGTACTAAATAG

>rpl22

ATGATAAGGATAATAAAGAAGAAGGTAGAAGTATCTGCTTTAGGTCAACATATATGTATGTCTGCTCACAAAGCCCGAAGGGTAATTGATCAGATTCGTGGACGTTCTTACGAGGAAACCCTTATGATACTCGAACTCATGCCTTATCGAGCATGTTATCCCATTTTAAAATTGGTTTATTCTGCAGCAGCAAATGGTATTCACAAGATGTTAGAATGTTAG

***P. chinense***

>psbA

ATGACTGCAATTTTAGAGAGACGCGAAAGCGAACGCCTATGGGGTCGCTTCTGTAACTGGATAACCAGCACCGAAAACCGCCTTTACATTGGATGGTTTGGTGTTTTGATGATCCCTACTTTATTGACTGCAACTTCTGTATTTATTATCGCCTTCATTGCTGCTCCTCCAGTAGATATTGATGGTATTCGTGAACCTGTTTCTGGATCTCTACTTTATGGAAACAATATTATTTCTGGTGCGATTATTCCTACTTCTGCAGCTATAGGTTTGCACTTTTACCCGATATGGGAAGCGGCATCTGTTGATGAATGGTTATACAATGGTGGTCCTTATGAGCTAATTGTTCTACACTTCTTACTTGGTGTAGCTTGTTACATGGGTCGTGAGTGGGAACTTAGTTTCCGTCTGGGTATGCGCCCTTGGATTGCTGTTGCATATTCAGCTCCTGTTGCAGCAGCGGCTGCTGTTTTCTTGATCTACCCAATCGGTCAAGGAAGTTTTTCTGATGGTATGCCTCTAGGAATCTCTGGTACTTTCAACTTCATGATTGTATTCCAGGCCGAGCACAACATTCTTATGCACCCATTTCACATGTTAGGCGTAGCTGGTGTATTCGGCGGCTCCCTATTCAGTGCTATGCATGGTTCCTTGGTAACCTCTAGTTTGATCAGGGAAACTACAGAAAATGAATCTGCTAATGAAGGTTACAGATTCGGTCAAGAGGAAGAAACTTATAATATCGTAGCTGCTCACGGTTATTTTGGCCGATTGATCTTCCAATATGCTAGTTTCAACAATTCTCGTTCTTTACATTTCTTCCTAGCTGCTTGGCCTGTAGTAGGTATCTGGTTTACCGCTTTAGGTATTAGCACTATGGCTTTCAACCTAAATGGTTTCAACTTCAACCAATCCGTAGTTGATAGTCAAGGTCGTGTAATTAATACCTGGGCTGATATTATTAATCGTGCTAACCTTGGTATGGAAGTTATGCATGAACGTAATGCTCATAACTTCCCTCTAGACCTAGCTGCTATTGAAGCTCCATCTACAAATGGATAA

>matK

ATGGAGGAATTTCAAGTATATTTAGAACTAGATAGATCTCAACAACACGACTTCCTATACCCACTTCTTTTTCGGGAGTATATTTATGCACTTGCTCATGATCTTGGTTTAAATAGCTCGATGATTTCATTGGAAAGTGGGGGTTATGACAATAAATCTAGTTCACTAAGTGTGAAACGGTTAATTACTCGAATGTATCAACAGATTCATTTGAGTATTGCTGCTAATGATTCTAACCAAAATCCAATTTTTGGGCACAACAAGAAGTTGTATTCTCAAATTATATCAGAGGGATTTGCTGTCGTGGTGGAAATTCAATTTTCCCCATGCTTGGTAGCTTTTTTAGAAGGTAAAGAAAATGAAAAATCTCCAAATTTCCAATCAATTCATTCAATATTTCCTTTTTTCGAGGACAAACTGTCACGTTTAAATTATGTGTTAGATGTACTAATACCCCACCCCATTTGTCCCGAAATCTTGGTTCAACGCCTTCGCTACTGGATAAAGGATGCCTCCTCTTTACATTTATTACGGTTCTTTCTCCACGAGTATTTTAATTCGAATAGTCTTATTACTCCAAAGAACTCTATTTCTGTTTTTTTAAAAAGGAATCCAAGATTGTTATTGTTTCTATATAATTCTCATGTATATGAATATGAATCCATCCTCTTTTTTCTCGGTAACCAATCGTCTCATTTACGATCAACATCCTCTCGAGTCCTCGTTGAGCGAATGTATTTCTATGGAAAAGTCGAACATCTTGTCGAAGTCTTTGCTAAAGATTTTCAGGACATCCTATGGTTGTTCAAGGATCCTTTCATGCATTATGTTAGATATCAAGGAAAATCCATTCTGGCTTCAAAGGATACGCCTCTTCTGATGAATAAATGGAAATATTACCTTGTTGGTTTATGGCAATGGCATTTTCACGTGTCGTCTCAACCAGGAAGGGTTCATCTAAACCACTTAGGCAAGTACTCTATCAACTTTCTGGGCTATCTTTCCGGTGTGCGACTCAATTCTTTGGTGGTACGGAGTCAAATGCTAGAAAATTCATTTCTAATAGGTAATTCTATGAAGAAGGTCGATACGACCGTTCCAATTATTCATCTGATTGGATCATTGATGAAGGCGCGGTTTTGTAACGCATTGGGGCATCCCATCAGTAAGGCGACCTGGGCCGATTTCTCCGATTCTCATCTTATCGACCGATTTGTGCGTATATGCAGAAATCTTTCTCATTATCACAGCGGATCCGCAAAAAAAAGTTTGTATCGAATAAAATATATACTTCGGCTTTCTTGTGTTAAAAGCTTGGTTCGTAAACATAAAAGTACTGTACGCGTTTTTTTGAAAAGATTAGGTTCGGAATTATTGGAAGAATTCCTTACGGAGGAAGAACACGTTCTTTCTTTAATCTTTCCAAGAGCTTCGTCTACTTCGCGTAGGTTTTATTTATATAGAGGGCGGATTTGGTATTTGGATATTATTTGTATCAACGATCTGGTTAATTATGAATGA

>rps16

ATGGTAAAACTTCGTTTGAAACGATGTGGTAGAAAGCAACGTGCCGTTTATCGAATCGTTGCAATTGATGGTCGATCCCGAAGAGAAGGAAGAGATCTTCAGAAAGTGGGTTTTTATGATCCGATAAATAATCAAACCCATTTAAATGTTCCTGCTATTCTATATTTCCTTGACAAGGGCGCCCAACCTACAGGAACCGTTCATGATATTTCAAAGAAAGCGGGGGTTTTTACAGAACTTAGTCTTAATCAAACGAAATTCTATTAA

>psbK

ATGCTTAATATCTTTAGTTTAATGTATATCTGTCTTAATTCTGCCCTTTATTCGAGTAGTTTTTTATTCGCCAAATTGCCCGAGGCCTACGCTTTTTTGAATCCAATTGTAGATGTTATGCCAGTAATACCTCTTCTATTTTTTCTCTTAGCCTTTGTTTGGCAAGCTGCTGTAAGTTTTCGATGA

>psbI

ATGCTTACTCTCAAACTCTTTGTTTACACCGTAGTGATATTCTTTGTTTCTCTCTTCATCTTCGGATTCCTGTCTAATGATCCAGGGCGTAATCCCGGACGCGAAGAATAA

>atpA

ATGACAACAATTAAAGCCGACGAAATTAGTAATATTATCCGCGAACGTATTGAGCAATATAATAGAGAAGTAAAGATTGTAAATATTGGTACCGTACTTCAAGTAGGCGACGGCATCGCCCGTATTTATGGTCTTGATGAAGTAATGGCAGGGGAATTAGTAGAATTTGAAGAGGGTACAATAGGCATTGCTCTTAATTTGGAATCAAATAATGTTGGTGTTGTTTTAATGGGTGACGGTTTAATGATACAAGAGGGAAGTTCTGTAAAAGCAACCGGCAAAATTGCTCAGATACCAGTAAGCGAGGCTTATTTAGGTCGTGTTATAAATGCCCTGGCTAAACCTATTGATGGTCGAGGTGAAATTTCAGCTTCTGAATCTCGATTAATCGAATCTCCCGCTCCGGGTATTATTTCGAGACGTTCCGTATATGAGCCTCTTCAAACAGGACTTATTGCTATTGATTCGATGATCCCTATAGGACGCGGGCAGCGAGAATTAATTATTGGGGACCGACAGACCGGTAAAACTGCAGTAGCCACGGATACGATTCTCAACCAACAAGGGCAAAATGTAATATGCGTTTATGTAGCTATTGGGCAAAAAGCATCTTCTGTGGCTCAGGTAGTGAATACTTTCCAGGAAAGGGGGGCAATGGAATACACTATTGTGGTAGCCGAAACGGCGGATTCCCCCGCTACGTTACAATACCTCGCTCCTTATACGGGCGCAGCTCTGGCTGAATATTTTATGTACCGTGAACGACACACTTTAATCATTTATGATGATCCCTCCAAACAAGCGCAGGCTTATCGACAAATGTCTCTTCTATTACGAAGACCACCCGGTCGCGAAGCTTATCCCGGAGATGTTTTTTATTTGCATTCACGGCTTTTGGAAAGAGCCGCGAAATTAGGTTCGCAGTTAGGTGAAGGAAGTATGACCGCTTTACCAATAGTTGAGACCCAGTCAGGAGATGTTTCGGCTTATATTCCTACTAATGTAATTTCCATTACAGATGGGCAAATATTCTTATCCGCCGATCTATTCAATGCTGGAATCAGACCCGCTATTAACGTGGGTATTTCTGTCTCTAGAGTAGGATCCGCAGCTCAAATTAAAGCTATGAAACAAGTAGCCGGCAAATTAAAATTGGAATTGGCCCAATTCGCGGAATTAGAAGCCTTTGCACAATTTGCTTCTGATCTCGATAAAGCTACTCAGAACCAATTGGCAAGGGGTCAACGCTTACGTGAGTTGCTGAAACAATCCCAATCAGCCCCTCTCACGGTCGAAGAACAGATAATGACTATTTATACAGGAACGAATGGTTATCTTGATTCATTAGAAATTGGCCAAGTAAGAAAATTTCTCGTTGAGTTACGTACTTACTTAAAAACGAATAAACCTCAGTTCCAAGAAATAATATCTTCTACCAAGATATTCACCGAGGAAGCAGAAGCCCTTTTGCAAGAAGCTATTCAGGAACAAATGGAACGCTTTCTACTTCAGGAACAATTATAA

>atpF

ATGAAAAATGTAACCGATTCTTTCGTTTCTTTGGTTCACTGGCCATTCGCCGGGAGTTTCGGGTTTAATACCGATATTTTAGCAACAAATCCAATAAATCTAAGTGTAGTGCTTGGTGTATTGATCTTTTTTGGAAAGGGAGTGTTAAGTGATTTATTAGATAATCGCAAACTGAGGATCTTGAATAGTATTCGAAATTCAGAAGAACTGCAGGGCGGGGCCGTTGAACGGCTGGAAAAAGCCCGGGCCCGGTTACGGAAAGTCGAAATAGAAGCAGATCAGTTTCGAGTGAACGGATACTCTGAGATAGAACGAGAAAAATTAAATTTGATTAATTCAACTTCTAAGACTTTGGGCCAATTAGAAAATTACAAAAATGAAACCATTCATTTTGAACAACAAAGAGCAATTAATCAAGTCCGACAACGGGTTTTCCAACAAGCTTTACAAGGAGCGCTCGGAACTCTGAATAGTTGTTTGAACAAGGAGTTACATTTACGTACCATTAGTGCCAATATTGGCATGTTTGGGGCGATGAACGAAATAACTGATTAG

>atpH

ATGAATCCACTTATTTCTGCCGCTTCCGTTATTGCCGCTGGGTTGGCTGTTGGGCTTGCTTCTATTGGACCTGGAGTTGGTCAAGGTACTGCTGCGGGCCAAGCAGTAGAGGGGATTGCGAGACAACCCGAGGCGGAGGGAAAAATACGAGGTACTTTATTGCTTAGTCTGGCTTTTATGGAAGCTTTAACAATTTATGGACTGGTTGTAGCATTAGCGCTTTTATTTGCGAATCCTTTTGTTTAA

>atpI

ATGAATGTTCTATCATGTTCCATCAACACACTAAAGGGGTTATACGATATGTCCGGTGTGGAAGTAGGCCAACATTTCTATTGGCAAATAGGCGGGTTCCAAGTCCATGCCCAAGTACTTATTACTTCTTGGGTTGTAATTGCTATCTTATTAGGTTCAGCCTTTATAGCCGTTCGGAATCCACAAACCGTTCCGACTGCCAGTCAAAATTTCTTCGAATATGTCCTTGAATTCATTCGAGACGTGAGCAAAACGCAGATTGGAGAAGAATATGGCCCATGGGTTCCCTTTATTGGAACTATGTTTCTTTTTATTTTTGTTTCGAATTGGTCAGGTGCTCTTTTACCTTGGAAAATCATAGAGTTACCTCATGGGGAGTTAGCCGCACCCACGAATGATATAAATACTACCGTTGCTTTAGCTTTGCTCACGTCAGTAGCATACTTCTATGCGGGTCTTTCCAAAAAGGGATTAGGTTATTTCAGTAAATACATTCAACCGACTCCAATTCTGTTACCCATTAACATTTTAGAAGATTTCACAAAACCCTTATCGCTTAGTTTTCGACTTTTCGGGAATATATTAGCCGATGAATTAGTAGTTGTTGTTCTTGTTTCTTTAGTCCCTTTAGTGGTTCCTATACCTGTCATGTTCCTTGGATTATTTACAAGCGGTATTCAAGCTCTTATTTTTGCAACTTTAGCTGCGGCTTATATAGGCGAATCTATGGAGGGACATCATTGA

>rps2

ATGGCAAGAAGATATTGGAACATCCATTTGGAAGAGATGATGGAAGCAGGAATTCATTTTGGTCATGGTACTCGGAAATGGAATCCTAGAATGGCACCTTATATATCTGCAAAACACAAAGGTATTCATATTACAAATCTGACTCGAACTGCTCGTTTTTTATCAGAAGCTTGTGATTTAGTTTTTGATGCAGCAAGTAGGGGAAAACAATTCTTAATTGTTGGTACTAAAAATAAAGCAGCTGATTCAGTCGCGCGAGCTGCAATAAGGGCTCGGTTTCATTATGTTAATAAAAAATGGCTCGGTGGTATGTTAACGAATTGGTCCACTACAGAAACGAGACTTCACAAGTTCAGGGATTTGAGAACGGAACAAAAAAAGGGGAGACTCGACAGTCTTCCCAAAAGGGATGCCGCTATTTTGAAGAGACAATTATCGCGCCTGCAAACGCATCTGGGCGGGATTAAATATATGACGAGGGTACCCGATATTGTAATCATCGTTGATCAGCACGAAGAATATACGGCTCTTCGAGAATGTATCACTTTGGGAATTCCAACAATTTGTTTAATCGATACAAATTGTGACCCTGATCTCGCAGATATTTCGATTCCAGCAAACGATGACGCTATAGCTTCAATCCGATTAATTCTTAACAAATTAGTATTCGCAATTTGTGAGGGTCGCTCTAGCTATATACGAAATCGTTGA

>rpoC2

ATGGCAGAACGGGCGAGTCTGGTCTTTCACAATAAAACGATAGATGGAACTGCCATTAAACGACTTATTAGCAGGTTAATAGATCACTTCGGAATGGCATATACATCACACATCCTGGATCAAGTAAAGACCCTGGGTTTCCAGCAAGCCACTGCTACATCTATTTCATTAGGCATTGATGATCTTTTAACGATACCTTCTAAGCGATGGCTAGTCCAAGATGCTGAACAACAAAGTTTTATTTTGGAAAAACACCACCATTATGGGAATGTACACGCGATAGAAAAACTACGTCAATCCATTGAGATATGGTATGCTACAAGTGAATATTTGCGACAAGAAATGAATCCTAATTTTAGGATGACTGATCCCTTTAATCCAGTCCATATAATGTCTTTTTCGGGAGCTAGAGGAAATGCATCTCAAGTACACCAATTGGTGGGTATGAGAGGATTAATGTCTGATCCCCAAGGTCAAATGATTGATTTACCCATTCAAAGCAATTTACGCGAAGGACTTTCTTTAACAGAATATATCATTTCTTGCTATGGAGCCCGTAAGGGAGTTGTAGATACCGCTGTACGAACATCAGATGCTGGATATCTTACGCGCAGACTTGTTGAAGTAGTTCAACACATTGTTGTACGTAGAACAGATTGTGGCACCATCCGAGAAATTTCTGTGAGTCCTCAAAATCAAAATAAGATGCTGTCGGAAAGGGTTTTTAGCCAAACATTAATTGGTCGTGTATTAGCAGACGATATATATATGGGTCCGCGATGCATCGCCATTAGAAATCAAGATATTGGGATTGGACTTGTCAATCGACTCATAACCTTTCGAACACAAGCAATATCTATTCGAACCCCCTTTACTTGTAGGAGTACATCTTGGATCTGTCGATTATGCTATGGTCGGAGTCCGACTCATGGTGACCTGGTTGAATTGGGGGAAGCCGTAGGTATTATTTCGGGTCAATCTATTGGGGAACCGGGGACTCAACTAACATTAAGAACTTTTCATACCGGTGGCGTATTTACAGGGGGCACTGCAGAACATGTACGAGCCCCTTCTAATGGTAAAATAAAATTCAACGAGGATTTGGTTCATCCCACGCGTACACGTCACGGGCATCCTGCTTTTCTATGTTCGATAGATTTGGATGTAATTATTGAGAGTGAAGATATTATGCATAATGTGACTATTCCACCAAAAAGTTTTCTTTTAGTTCAAAATGATCAATATGTCGAATCAGAACAAGTGATTGCTGAGATTCAGGCGGGAGCATACACTTTGAATTTTAAAGAGAGGGTTCGAAAACATATCTATTCTGATTCAGAGGGAGAAATGCACTGGAGTACTGATGTGTACCATGCACCCGAATTTACATATAGCAATGTACACCTCTTGCCAAAAACAAGTCATTTATGGATATTATCGGGGGGTTCATGCAGATCTAGTGTAGTTTCTTTTTCACTCTACAAGGATCAAGATCAAATGAATATTCATTCTCTTTCTGTCGAACGAAGAGAGATTTCTAGCCTCTCGCTCTCGGTGAATAATGATCAAGCGAGACACAAATTATTTAGTTCTGCTTTTTCTGCTAAAAAAGAAGGTGGAATTCTTGAGATGTCTGATTATTCGGGATTTAATAGAATCATAGGTACTGGTCATTGTAATCTCATACATCCTGCAATTCTCCGCGCGAATTCGAATTTATTGGCAAAAAGGCAAAGAAATCGATTTCTTATTCCATTCCACTCGATTCAAGAACAAGAGAAAGAGCTAATGCCCCATTCAGGAATCTCGATTGAAATACCCATAGGGGGTATTTTCCGTAGAAATAGTATTCTTGCTTATTTCGACGATCCTCGATACAGAAGAAAGAGTTCCGGAATTACTAAATATGGGACTCTGGGGGCGCATTCAATCGTCAAAAAAGAGGACTTGATTGAGTATCGAGGACTCAAAAAAATTAAGCCAAAATACCAAATTAAAATAGATCGCCTTTTTTTCATTCCCGAGGAAGTGCATTTTTTTCCCGAATCTTCTTACCTAATGGTACGGAATAATAGCATCATTGGAGTAGATACACGAATCACTTTAAATATAAGAAGCCGAGTGGGCGGATTGGTCCGAATAGAGAGAAAAAAAGGGGGGATTGAACTAAAAATATTTTCGGGAGATATCCATTTTCCCGGAGAGATAGATAAGATATCCCGACACAGTGGCATCTTGATACCGCCAGAAAGGGAAAAAAAAAAACTTAAGGAAGCCACTAAGGAATCAAAAAAATTGAAAAAATGGATCTATGTTCAACGGATCACACCTACCAAGAAAAAGTATTTTGTTTTGGTTCGACCCGTAGTCACATATGAAATAGCGGACGGTATAAATTTAGCAACACTCTTCCCCCAGGATCCGCTGCGGGAAAAGGATAATATGCAATTTCGAGTTGTCAATTATGTCCTTTATGGGAAGGGCAAAGCTGCTCGGGGAATTCCTGATACAAGTATTCAATTAGTTCGGACGTGTTTAGTGTTGAATTGGGACCAAGACAAAAAAAGTTCTTCCGTCGAAGAGGTTTGTGCTTCCTTTGTTGAAGTACGTACAAATGGTCTGATTCGCGATTTCTTAAGACTCAACTTAGTGAAATCCCAAATTTCGTATATCAGAAAAAGGAATCATCCGTCAGGTTCAGGATTGATCTCTGATAATGGTTCCGTTCGCACCAATAGCAATCCGTTTTATTCCGTTTTTGGCAAGGCAGGGGTTGAACAATCACTTAGCCAAAATCAAGGAACTATTCGTACGTTGTTGAATAGAAATAAGGAATGCCAATCTTTGATAATTTTGTCATCATCTAATTATTTTAGAATGGGTCCATTGACCGATGTAAAATATCACAATGTGATAAAACAATCAATTCCAATTCAAAAAGGTTCTCTAACCCCAATTAGGAATTCGTTGGGACCCTTAGGAACAGCCCTTCAAATTGAGAATTTTTATTCATTTTACTATTTAATAACTCTAATAACTCATAATCATCTCTCGGTAACTAAATATTTGAAACTTGACAATTTAAAACAGCCTTGTCAAGTACTTAAATATTATTTAATGGATGAAAACGGGGAAATTTCTAATCCTGATACAGACAGTAAGATCATTTTGAATCCATTTAATTTGAATTGGTATTTTCTCCATCATAATTATTGTGAGGAAATGTCCCCGATAATAAGTCTTGGGCAGTTTCTTTGTGAAAATGTATGTATAACCAAAAACGGACCACACCTAAAATCTGGTCAAGTTTTAATTGTTCAAGTTAACTCTGTAGTAATACGATCAGCTAAGCCTTATTTGGCTACTCCTGGAGCAACTGTTCATGGGCATTATGGAGAAATCCTTTACGAAGGGGATACATTAGTTACATTTATATATGAAAAATCGAGATCTGGTGATATAACGCAGGGCCTTCCAAAAGTAGAACAAGTGTTAGAAGTACGTTCGCTTGATTCAATATCGATGAACCTAGAAAAGAGAGTTGAGGGTTGGAACGCGCGTATAACAAGAATTCTTGGGATTCCCTGGGGATTCTTGATTGGTGCTGAGCTAACTATAGTGCAAAGTCGTATCTCTTTGGTTAATAAGATCCAAAAGGTTTATCGATCGCAGGGGGTGCAGATCCATAATAGGCATATAGAAATTATTGTACGTCAAATAACATCAAAAGTCTTGGTTTCAGAAGCTGGAATGTCTAATATTTTTTTACCCGGCGAACTTATTGGATTGTTACGAGCGGAACGAACGGGGCGCGCTTTGGAAGAAGTGATCTGTTATCGAGCTATCTTATTGGGAATAACGAGAGCATCTCTGAATACTCAAAGTTTTATATCCGAAGCAAGTTTTCAAGAAACCACGCGAGTTTTAGCAAAAGCAGCTCTCCGAGGTCGTATCGATTGGTTGAAAGGCCTGAAGGAAAACGTTGTTCTGGGGGGGATAATACCCGTTGGTACCGGATTCAAAGGATTAGTGCACTGTTCAAGGCAGCATAACACCATTCTTTTGGAAAGACAAAAAGGGAATTTATTCGGGGGGGAAATGAGAGATATTTTCTTACACCACAGAGAATTATTTGACTCTTGCATTTCAACGACTTTCCATGATACATCAGAGCAATTGCTTAGAGGGTTTAATGAGTCCTAG

>rpoC1

ATGATTGATCGATATAAACATCAACAACTCCGAATTGGATCAGTTTCTCCTCAACAAATAAGCGCTTGGGCCAATAAAATCCTACCTAATGGAGAGATTGTTGGAGAAGTGACAAAACCCTATACTTTTCATTACAAAACCAATAAGCCGGAAAAAGATGGATTATTTTGTGAAAGAATTTTTGGGCCTATTAAAAGCGGAATTTGCGCTTGTGGAAATTATCGAATAATCGGAGATGAAAAGGAAGACCCGCAATTTTGTGAACAATGTGGAGTTGAATTTGTTGATTCTCGGATACGAAGATATCAAATGGGATACATAAAACTAGGCTGCCCAGTAACCCACGTGTGGTATTTGAAACGTCTTCCTAGTTATATCGCGAATCTTTTAGATAAACCTCTTAAAGAATTAGAAGGCCTAGTATTTTCTTTTGCTAGGCCTATAGCGAAAAAACCGACTTTTTTACGATTACGAGGTTCATTCGAATATGAAATACAATCCTGGAAATACAGCATCCCGCTTTTTTTTACTACCCAAGGTTTCGATAAATTTCGTAACCGAGAAATTTCTACTGGAGCAGTTGCTATCCGGGAACAATTAGCCGATCTAGATTTGCGAATTATTCTAGATTATTCCTTGTTAGAATGGAAAGAATTAGGGGAAGAAGGACCCGCGGGTAATGAATGGGAAGATCTAAAAGTTGGACGAAGAAGGGATTTTTTGGTTAGACGCATGGAATTAGCTAAGCATTTTCTTCGAACAAATATAGAACCTGAGTGGATGGTTTTATGTCTATTACCTGTTCTTCCTCCCGAGCTGAGACCGATCATTCAGATAGATGGAGGTAAACTAATGAGTTCAGATATTAATGAACTCTATAGAAGAGTTATCTATCGGAACAATACTCTTACCGATCTATTAACAACAAGTAGATCTACGCCAGGGGAATTAGTAATGTGTCAGGAGAAATTGGTACAAGAAGCCGTGGATACGCTTCTTGATAATGGAATCCGTGGCCAGCCAATGAGGGATGGTCATAATAAGATTTATAAGTCGTTTTCAGATGTAATTGAAGGCAAAGAGGGAAGATTTCGTGAGACTCTGCTTGGCAAACGGGTTGATTATTCGGGGCGTTCTGTCATTGTTGTAGGCCCCTCACTTTCGTTACATCGATGTGGATTGCCTCGCGAAATCGCAATAGAGCTTTTCCAGAGTTTTGTAATTTGTGGGCTAATTAGACAACATCTTGCTTCGAACATAGGAGTTGCTAAGAGTAAAATTCGGGAAAAAGGGCCAATTGTATGGGAAATACTGCAGGAAGTTATGCAGGGACATCCAGTATTGCTGAATAGAGCGCCTACTCTGCATAGATTGGGCATACAGGCATTCCAGCCCATTTTAGTGGAAGGGCGCGCTATTTGTTTACATCCATTAGTTTGTAAGGGATTCAATGCAGACTTTGATGGGGATCAAATGGCTGTTCATGTACCTTTATCTTTAGAGGCTCAAGCGGAGGCTCGTTTACTTATGTTTTCTCATATGAATCTCTTGTCTCCTACTATTGGAGATCCCATTTCCGTACCGACTCAAGATATGCTTATTGGACTCTATGTATTAACGAGCGGGAATCGTCGAGGTATTTGTGCAAATAGGTATCATCCATGTAATCGAAGAAATTATCAAGATGAAAGAATTGACGATAATAGCTATAACTATAAGTATACGAAGGAACCCTTTTTTTGTAATTCCTATGATGCAATTGGGGCTTATCGGCAGAAAAGAATCAATTTAGATAGTCCTTTGTGGCTCCGGTGGCGATTAGATCAACGCGTTATTGCTTCAAGGGAAGCTCCCATCGAAGTTCACTATGAATCTTTGGGTACCTATCATGAGATTTATGGACATTATCTAATAGTACGAAGTGTAAAAAAAGAAATTCTTTCTATATACATTCGAACCACCGTTGGCCATATTTCTTTTTATCGAGAAATCGAAGAAGCTATACAAGGGTTTTGTCTGGCCTGCTCATATGGTACCTAA

>rpoB

ATGCTCGGAGATGGAAATGCGGGAATGTCTACAATACCTGGGTTGAATCAGATACAATTTGAAGGCTTTTGTAGGTTCATTGATCAGGGCTTAACAGAAGAACTTTATAAGTTTCCAAAAATTGAAGATACGGATCAAGAAATTGAATTTCAATTATTTGTGGAAACATATCAATTGGTAGAACCCTTGCTAAAAGAAAGAGATGCTGTATATGAATCATTCACGTATTCTTCTGAATTATATGTATCAGCAGGATTAATTTGGAAAAGCCGAGGGGATATGCAGGAACAAACAATTTTTATTGGAAACATTCCTCTAATGAATTCTTTGGGAACTTCTATAGTAAATGGAATATACAGAATTGTCATCAATCAAATATTGCAAAGTCCCGGTATCTATTATCGGTCAGAATTGGGCCATAATGGAATGTCGGTCTATACAGGCACCATAATATCCGATTGGGGAGGAAGATTCGAATTAGAGATTGATAGAAAAGCAAGGATATGGGCTCGTGTGAGTAGGAAACAGAAAGTCTCTATTCTAGTTCTATCAGCAGCTATGGGTTCGAATCTACGAGAAATTCTAGAGAATATTTGCTACCCTGAAATTTTCTTGTCTTTCCTGACCAATAAGGAGAAAAAAAAAATTGGATCAAAAGAAAATGCCATTTTGGAGTTTTATCAACAATTTGCTTGTGTAGGCGGAGATCCGGTATTTTCGGAATCCTTATGTAAGGAATTACAAAAGAAATTTTTTCACCAAAGATGTGAATTAGGAAAGATTGGTAGACGAAATATGAACCAGAGACTGAATCTTAATATACCTCCGAACAATACCTTTTTGTTACCACAAGATGTATTGGCAGCTGTCGATCATTTGATTGGACTGAAATTTGGAATGGGTACACTTGACGATATGAATCATTTGAAAAATAAGCGTATTCGGTCTGTAGCGAATCTTTTACAAGATCAATTCGGATTGGCCCTGGTTCGGTTAGAAAATGTGATTAGAGGAACTATATGCGGGGCAATTAGGCATAAATTGATGCCAACTCCTCAAAATTTGGTAACTTCAACTCCATTAACAACCACTTATGATTCTTTTTTCGGATTACATCCATTATCTCAAGTTTTGGATCGAACTAATCCATTGACACAAATAGTTCATGGGATAAAATTGAGTTATTTGGGCCCCGGAGGATTGACAGGACGGACTGCTAGTTTTCGGATACGAGATATCCATCCTAGTCACTATGGCCGCATTTGCCCAATTGACACGTCTGAAGGAATCAATGTTGGACTTATTGGATCCTTAGCAATTCATGCGAGAATTGGTTATTGGGGGTCTCTAGAAAGCCCATTTTATGAAATCTTTGAAAAATCAAAAAAAGTACGGATGCTTTATTTATCACCAAGTAGAGATGAATACTATATGGTAGCGGCAGGAAATTCTTTGGCACTGAATCAGGGTAGTCAGGAAGAACAGGTTGTTCCGACTCGATACCGTCAAGAATTCCTGACTATTGCGTGGGAACAGGTTCATCTTCGAAGTATTTTTCCCTCCCAATATTTTTCTATTGGGGCTTCCCTCATTCCTTTTATCGAGCATAATGATGCGAATCGGGCTTTAATGAGTTCGAATATGCAACGCCAAGCAGTTCCGCTCCTTCGGTCCGAGAAGTGCATTGTTGGAACTGGGTTGGAACGCCAAGTGGCTCTAGATTCAGGGGTTCCCGCTATAGCCGAACATGAGGGAAAGATCATTTATACCGATATTGACAAGATCGTTTTATCGGGCAACGGGAATACTTATAGTATTCCTTTAGTTCTGTATCAACGTTCAAACAAAAATACTTGTATGCATCAAAAAACCCAGGTTGGGCGGGGTAAATGCATTAAAAAGGGCCAAGTTTTAGCGGATGGTGCCGCTACAGTTGGTGGCGAACTCGCTTTAGGGAAAAACGTATTAGTAGCTTATATGCCATGGGAAGGTTACAATTTTGAGGATGCGGTACTTATTAGCGAACGTCTGATATATGGAGATATTTATACTTCTTTTCACATACGGAAATATGAAATTCAGACTCATGTGACAAGCCAAGGTCCCGAAAGGATCACTAATGAAATACCACATCTAGAAGCCCGTTTACTCCGCAATTTAGACAAAAATGGAATTGTGATGCTGGGATCTTGGGTAGAGACCGGCGATATTTTAGTAGGTAAATTAACGCCTCAGGCGGCGAAAGAATCATCCTATGCTCCGGAAGATAGATTATTACGGGCCATACTTGGCATTCAGGTCTCCACTTCAAAGGAAACTTGCCTAAAACTACCTATAGGTGGTAGGGGTCGAGTTATTGATGTGAGATGGGTCCAGAAAAAGGGGGGTTCCAGTTATAATCCCGAAACGATTTGTGTATATATTTCACAGAAACGTGAAATCAAAGTAGGTGATAAAGTAGCTGGAAGACATGGAAATAAGGGTATCATTTCAAAAATTTTGCCTAGACAGGATATGCCTTATTTGCAAGATGGAAGGCCTGTTGATATGGTTTTCAACCCATTAGGAGTACCCTCGCGAATGAATGTAGGACAGATATTTGAATGCTCGCTCGGGTTAGCGGGGGGTCTGCTAAATCGACATTATCGAATAGCACCTTTTGATGAGAGATATGAACAAGAGGCTTCGAGAAAACTCGTGTTTTCTGAATTATATGAAGCCAGTAAGCAAACATCGAATCCATGGGTATTTGAGCCGGAATACCCGGGAAAAAGCAGAATATTTGATGGACGAACGGGAGATCCTTTTGACCAACCTGTTCTAATAGGAAAGCCTTATATCTTGAAATTAATTCATCAAGTTGATGATAAAGTACACGGACGTTCCAGTGGGCATTATGCACTTGTTACCCAACAACCCCTTAGAGGAAGGTCAAAACAGGGGGGGCAGCGGGTAGGAGAAATGGAGGTTTGGGCTCTAGAGGGCTTTGGTGTTGCTCATATTTTACAAGAGATGCTTACTTATAAATCTGATCATATTAGAGCTCGCCAAGAAGTACTTGGTACTACAATCATTGGAGAAACAATACCTAGCCCCGAAGATGCTCCAGAATCTTTTCGATTGCTCGTTCGAGAACTACGATCTTTGGCTTTGGAACTGAATCATTTTCTTGTATCTGAGAAAAACTTCCAGATTAATAAGAAGGAAGCTTAA

>psbM

ATGGAAGTAAATATTCTCGCATTTATTGCTACTACACTGTTCGTTCTAGTTCCTACTGCTTTTTTGCTTATAATATACGTAAAAACGGTCAGTCAAAGTGATTAA

>psbD

ATGACTATAGCCCTTGGTAAATTTACCAAAGATGAAAAAGATTTATTTGATATTATGGATGACTGGTTACGGAGGGACCGATTCGTTTTTGTAGGTTGGTCCGGTCTATTGCTCTTTCCTTGTGCCTATTTCGCTTTAGGGGGTTGGTTCACAGGCACAACCTTTGTAACTTCATGGTATACCCATGGATTGGCCAGTTCCTATTTGGAAGGCTGCAACTTCTTAACCGCCGCAGTTTCGACCCCTGCTAATAGTTTAGCGCATTCTTTGTTGTTACTATGGGGTCCTGAAGCACAAGGGGATTTTACTCGTTGGTGTCAATTAGGCGGTCTTTGGACTTTTGTTGCTCTCCACGGTGCTTTCGGACTAATAGGTTTCATGTTACGTCAATTTGAACTTGCTCGCTCTGTGCAATTGCGACCTTATAATGCAATAGCATTCTCTGGTCCAATTGCTGTTTTTGTTTCTGTATTCCTGATTTATCCACTGGGTCAGTCTGGTTGGTTCTTTGCGCCTAGTTTTGGTGTAGCGGCGATATTTCGATTCATCCTTTTTTTCCAAGGGTTTCATAATTGGACATTGAACCCCTTTCATATGATGGGAGTTGCCGGTGTATTGGGCGCAGCTCTGCTATGTGCTATTCATGGCGCTACCGTAGAAAATACTTTATTTGAAGATGGTGATGGTGCAAATACATTCCGCGCTTTTAACCCAACGCAAGCTGAAGAAACTTATTCGATGGTCACCGCTAACCGCTTTTGGTCCCAAATCTTTGGGGTTGCTTTTTCCAATAAACGTTGGTTACATTTCTTTATGTTATTTGTACCAGTAACCGGTTTATGGATGAGTGCTCTTGGAGTAGTCGGCCTAGCCCTGAACCTACGTGCTTATGACTTCGTTTCCCAGGAAATCCGTGCAGCGGAAGATCCTGAATTTGAGACTTTTTACACAAAAAATATTCTTTTAAACGAGGGTATTCGTGCTTGGATGGCGGCTCAAGATCAGCCTCATGAAAACCTTATATTCCCTGAGGAGGTTCTACCCCGTGGAAACGCTCTTTAA

>psbZ

ATGACTATTGCTTTCCAATTGGCTGTTTTTGCATTAATTGCTACTTCATCAATCTTACTTATTAGTGTACCCGTTGTATTTGCTTCTCCGGATGGTTGGTCGAGTAACAAAAATGTCGTATTTTCTGGTACATCGTTGTGGATTGGATTAGTCTTTCTGGTGGGTATCCTTAATTCTCTCATCTCTTAA

>rps14

ATGGCAAGGAAAAGTTTGATTCATAGGGAGAAGAAAAGGCAAAAATTGGAACAAAAATATCATTTGATTCGTCGATCCTCAAAAAAAGAAATAAACAAAGTTCCGTCGTTGAGCGATAAATGGAAAATTCATGGAAAGCTACAGTCCTCACCGCGTAATAGTGCACCTACACGTCTTCATCGACGTTGTTTTTCGACCGGAAGGCCGAGAGCTAACTATCGAGACTTTGGACTATCCGGGCACATACTTCGTGAAATGGTTCATGCATGTTTGTTGCCGGGGGCAACAAGATCAAGTTGGTAA

>psaB

ATGGCATTAAGATTTCCAAGGTTTAGCCAAGGCTTAGCTCAGGACCCCACTACTCGTCGTATTTGGTTTGGTATTGCTACCGCACATGACTTCGAGAGTCATGATGATATTACTGAGGAACGTCTTTATCAGAATATTTTTGCTTCTCACTTCGGACAATTAGCAATAATTTTTCTGTGGACTTCCGGAAATCTCTTTCATGTAGCTTGGCAAGGAAATTTTGAGGCATGGGTACAGGACCCTTTACATGTAAGACCTATTGCTCATGCAATTTGGGATCCTCATTTTGGTCAACCGGCCGTGGAAGCTTTTTCTCGGGGAGGTGCTCTTGGCCCGGTGAATATCGCTTATTCTGGTGTTTATCAGTGGTGGTATACAATTGGTTTACGCACTAATGAGGATCTTTATACTGGAGCTCTTTTTCTATTATTTCTTTCTGCCATATCCTTAATAGCGGGTTGGTTACACCTACAACCGAAATGGAAACCGAGTGTTTCGTGGTTCAAAAATGCCGAATCTCGTCTCAATCATCATTTGTCAGGACTGTTCGGAGTAAGTTCCTTGGCTTGGACAGGACATTTAGTACATGTGGCTATTCCTGGATCCAGGGGGGAATATGTTCGATGGAATAATTTCTTAGATGTATTACCGCATCCCCAAGGGTTAGGCCCACTTTTTACAGGTCAGTGGAATCTTTATGCTCAAAACCCCGATTCAAGTAGTCATTTATTTGGTACCTCCCAAGGATCAGGAACTGCCATTCTAACCCTTCTCGGGGGATTCCATCCACAAACGCAAAGTTTATGGCTGAGCGATATTGCTCATCATCATTTAGCTATTGCAATTCTTTTCCTGATCGCGGGTCACATGTATAGAACAAATTTCGGGATTGGGCACAGTATAAAAGATCTTTTAGAAGCACATATTCCTCCGGGAGGACGATTGGGGCGCGGGCATAAGGGTCTTTATGACACAATCAACAATTCGCTTCATTTTCAATTAGGCCTTGCTCTAGCCTCTTTAGGGGTTATTACTTCCTTGGTAGCTCAACACATGTACTCTTTACCTGCTTATGCGTTCATAGCGCAAGATTTTACTACTCAAGCTGCGTTATATACTCATCACCAATACATCGCAGGATTCATCATGACCGGAGCTTTTGCTCATGGAGCTATCTTTTTTATTAGAGATTACAATCCGGAACAGAATGAGGATAATGTATTGGCAAGAATGTTAGACCATAAGGAAGCTATTATATCCCATTTAAGTTGGGCCAGTCTCTTTCTGGGGTTCCATACTTTGGGACTTTATGTTCATAATGATGTCATGCTTGCTTTTGGTACTCCGGAGAAACAAATCTTGATCGAACCGATATTTGCCCAATGGATACAATCTGCTCATGGTAAAACTTCATATGGATTCGATGTACTTTTATCTTCAACGAATGGCCCTGCATTTAATGCGGGTCGAAGCATATGGTTGCCTGGCTGGTTAAATGCTATTAATGAGAATAGTAATTCTCTATTCTTAACAATAGGCCCTGGAGACTTCTTGGTTCATCATGCTATTGCTCTAGGTTTACATACAACTACATTGATCTTAGTAAAGGGGGCTTTAGATGCACGGGGTTCCAAGTTAATGCCAGATAAAAAGGATTTCGGTTATAGTTTTCCTTGCGACGGTCCGGGACGAGGCGGTACTTGTGATATTTCGGCTTGGGACGCATTTTATTTGGCAGTTTTCTGGATGTTAAATACCATTGGGTGGGTTACTTTTTATTGGCATTGGAAACACATCACGTTATGGCAGGGTAACGTTTCACAGTTTAATGAATCTTCCACTTATTTGATGGGCTGGTTAAGAGATTATCTATGGTTAAACTCTTCCCAACTTATCAATGGGTATAACCCGTTTGGTATGAATAGTTTATCAGTCTGGGCGTGGATGTTCTTATTTGGACATCTTGTTTGGGCTACTGGATTTATGTTCTTAATTTCCTGGCGCGGGTATTGGCAAGAATTGATTGAAACTTTAGCATGGGCTCACGAACGCACACCCTTGGCTAATTTGATTCGATGGAGAGATAAACCAGTGGCTCTTTCCATTGTGCAAGCAAGATTGGTTGGATTAGCCCACTTCTCTGTAGGTTATATATTCACTTATGCGGCTTTCTTGATTGCCTCTACATCGGGCAAATTTGGTTAA

>psaA

ATGATTATTCGTTCGCCGGAACCAGAAGTAAAAATTTTGGTAGATAGGGATCCCATAAAGACTTCTTTCGAGGAATGGGCCAGACCGGGTCATTTCTCAAGAACCATAGCTAAGGGACCTGATACTACCACTTGGATCTGGAACCTACATGCTGATGCTCACGACTTCGATAGCCATACCAGTGATTTGGAGGAGATCTCTCGAAAAGTATTTAGTGCCCATTTCGGACAACTCTCCATCATTTTTCTTTGGCTGAGCGGCATGTATTTCCACGGTGCTCGTTTTTCCAATTATGAAGCCTGGCTAAGCGATCCTACTCACATTGGACCCAGTGCACAGGTGGTTTGGCCAATAGTGGGCCAAGAAATATTGAACGGTGATGTGGGCGGGGGTTTCCGAGGAATACAAATAACCTCCGGGTTTTTTCAGATTTGGCGAGCATCTGGAATAACTAGTGAATTACAACTCTATTGTACCGCAATTGGCGCATTGATTTTTGCAGCCTTAATGCTTTTTGCTGGTTGGTTCCATTATCACAAAGCTGCTCCAAAATTGGCTTGGTTTCAGGATGTAGAATCTATGTTGAATCACCATTTAGCGGGGCTGCTAGGGCTCGGGTCCCTTTCTTGGTCCGGGCATCAAGTACATGTATCTTTACCGATTAACCAATTTCTAAACGCTGGAGTAGATCCTAAAGAGATCCCACTTCCTCATGAATTTATCTTGAATCGGGATCTTTTGGCTCAACTTTATCCCAGTTTTGCCGAAGGAGCAACCCCATTTTTTACCTTGAATTGGTCAAAATATGCGGACTTTCTTACTTTTCGTGGCGGATTAGATCCAGTAACTGGGGGTCTATGGCTGACCGATATTGCACACCATCATTTAGCTATTGCAATTCTTTTCCTGATCGCGGGTCACATGTATAGGACCAACTGGGGAATTGGTCATGGTCTAAAAGATATTTTAGAGGCCCATAAAGGTCCATTTACGGGTCAAGGCCATAAAGGACTATATGAGATCCTAACAACATCATGGCATGCTCAATTATCGCTTAACCTAGCTATGTTAGGATCTTTAACCATTGTTGTAGCTCACCATATGTATTCCATGCCCCCTTATCCATATCTAGCTACTGACTATGGTACACAACTGTCATTGTTCACACATCACATGTGGATTGGTGGATTTCTCATCGTTGGCGCTGCTGCGCATGCAGCCATTTTTATGGTAAGAGACTATGATCCAACTACTCGATACAATGATCTCTTAGATCGTGTCCTTCGGCATCGCGATGCAATCATATCACATCTCAACTGGGTATGTATATTTCTAGGATTTCACAGTTTTGGTTTATATATTCATAATGATACAATGAGTGCTTTAGGGCGTCCACAAGATATGTTTTCAGATACTGCGATACAATTACAACCCGTCTTTGCTCAATGGATACAAAACACCCACGCCTTAGCACCTGGTGGAACGGCCCCTGGTGCAACAGCAAGCACCAGTTTGACTTGGGGGGGTGGTGATTTAGTGGCAGTGGGTGGAAAAGTTGCTTTGTTACCTATTCCATTAGGAACCGCGGATTTTTTGGTACATCACATTCACGCATTTACGATTCATGTGACGGTATTGATACTCCTGAAAGGAGTTCTATTTGCTCGTAGCTCGCGTTTGATACCGGATAAAGCAAATCTTGGGTTTCGTTTCCCTTGTGATGGGCCTGGAAGAGGGGGGACATGTCAAGTATCCGCTTGGGATCATGTCTTCTTAGGACTATTCTGGATGTACAATGCAATTTCGGTAGTAATATTCCATTTCAGTTGGAAAATGCAGTCAGATGTTTGGGGTAGTATAAGCGATCAGGGGGTGGTAACTCATATTACAGGAGGAAACTTTGCGCAGAGTTCCATTACGATTAATGGGTGGCTCCGCGATTTTTTATGGGCACAGGCATCCCAGGTAATTCAGTCTTATGGTTCTTCATTATCTGCATATGGCCTTTTTTTCCTAGGTGCTCATTTTGTCTGGGCTTTTAGTTTAATGTTTCTATTCAGCGGGCGTGGTTACTGGCAAGAACTTATTGAATCCATCGTTTGGGCTCATAATAAATTAAAAGTTGCTCCTGCTACTCAGCCTAGAGCCTTGAGCATTGTACAAGGACGTGCTGTAGGAGTAACCCATTACCTTCTGGGTGGAATTGCCACAACATGGGCGTTCTTCTTAGCAAGAATTATTGCAGTAGGATAA

>ycf3

ATGCCTAGATCGCGGATAAATGGAAATTTTATTGATAAGACCTTTTCAATTGTAGCCAATATATTATTACGAATAATTCCGACAACTTCAGGAGAAAAAGAGGCATTTAGCTATTACAGAGATGGTGCGATGTCAGCTCAATCTGAAGGCAATTATGCGGAAGCTTTACAGAATTATTATGAAGCTATGCGACTAGAAATTGATCCCTACGATCGAAGCTATATACTCTATAACATAGGCCTTATCCACACAAGTAACGGAGAACATACAAAAGCTTTAGAATATTATTTTCGGGCACTCGAACGAAATCCATTCTTACCACAAGCTTTGAATAATATGGCTGTGATCTGTCATTACCGGGGAGAACTGGCCGTTCGGCAGGGAGATTCTGAAATTGCGGAGGCTTGGTTCAACCAAGCCGCCGAGTATTGGAAACAAGCTATTGCGCTTACTCCTGGTAATTATATTCAAGCGCAGAATTGGTTGAAGATCACGGGGCGTTTCGAATAA

>rps4

ATGTCACGTTACCGAGGGCCTCGTTTCAAAAAAATACGCCGTCTGGGGGCTTTGCCGGGACTAACGAGTAAAGGGCCTAGAGCCGGAAGCGATTTTAGAAACCAATCGCGCTCCGGAAAAAAATCTCAATATCGAATTCGTTTAGAAGAAAAACAAAAATTGCGTTTTCATTATGGTCTTACAGAACGACAATTACTTAAATATGTTCGTATCGCCGGAAAGGCCAAAGGGTCCACCGGTCTGGTTTTACTACAATTACTTGAAATGCGTTTGGATAACATTCTTTTTCGATTGGGTATGGCTTCAACTATTCCTCAAGCCCGCCAATTAGTTAACCACCGACATATTTTAGTTAATGGGCGTATAGTCGATATACCAAGTTATCGTTGCAAACCCCGAGATATTATTACAACAAAGGATGACCAAAAATCTAGATCTCTGATTCAAAATTATCTTGATTCATCCCACCATGAGGAATTGCCAAAGCATTTGACTCTTCACGCATTCCAATATAAAGGATTAGTCAATCAAGTAATAGATAGTCAATGGGTCGGTTTGAAAATAAATGAATTGCTTGTCGTAGAATATTATTCTCGTCAGACTTAA

>ndhJ

ATGCAGGGTCGTTTGTCTGCTTGGCTAGTCAAGCATGGGCTAGTTCATAGATCTTTGGGCTTCGATTACCAAGGAATAGAAACTTTACAAATAAAGCCCGAGGATTGGCATTCCGTTGCTGTCATTTTGTATGTATATGGTTACAATTATCTACGTTCCCAATGTGCCTATGATGTAGCACCGGGTGGACTGTTAGCCAGTGTGTATCATCTTACGCGACTAGAGTATGGTGTAGATCAACCGGAAGAGGTATGCATAAAAGTATTTGCTCCAAGGAGTAATCCGAAAATTCCATCTGTTTTCTGGGTTTGGAAAAGTGCGAATTTTCAAGAACGGGAATCTTATGATATGTTGGGAATCCTTTATGATAATCATCCACGACTGAAACGTATCTTAATGCCGGAAAGTTGGATAGGGTGGCCCTTGCGTAAGGATTATATTGCCCCCAATTTTTATGAAATACAAGATGCATATTGA

>ndhK

ATGAATTCCATTGAATTTTCCTTACTTGATCGAACAACCCCAAATTCATTTATTTCAACTACATCAAATGATCTTTCAAATTGGTCAAGACTCTCCAGTTTATGGCCGCTTCTTTATGGTACCAGTTGTTGTTTCATTGAATTTGCTTCATTAATCGGCTCGCGGTTCGATTTTGACCGTTATGGACTGGTACCAAGATCGAGCCCTAGACAGGCGGACCTAATTTTAACAGCTGGTACCGTAACAATGAAAATGGCTCCTTCTTTAGTGAGATTATATGAACAAATGCCTGAACCAAAATATGTTATTGCTATGGGAGCATGTACAATTACAGGGGGGATGTTCAGTACCGATTCGTATAGTACTGTTCGGGGAGTTGATAAGCTAATTCCTGTGGATGTTTATTTGCCGGGCTGTCCGCCTAAACCAGAGGCAGTTATAGATGCCATAACAAAACTTCGTAAGAAAATATCTCGAGAAATCTACGAAGATCGAATTCGATTGCAAGGGGAGAATCGCTCGTTTACTTTTACTACCAATCACAAGTTTCGTGTTGTATGCAGTACTCATACTGGAAATTATGATCAAGGATTACTCTATCAACCACCATCTACGTCAGAAATTCCACCTGAAACCTTTTTCAAATACAAAAGTTCAGTATCTTCCCCCGAATTCATTAATTAA

>ndhC

ATGTTTCTGCTTTACAAATATGATATTTTCTGGGCATTTCTAATAATATCAAGCGTTATTCCTATTTTTGCATTTCTAATTTCCGCAGTTTTAGCCCCGATTAACAAAGGGCCAGAGAAACTTTCTAGTTATGAATCGGGTATAGAACCAATGGGCGATGCTTGGTTACAATTTCGAATCCGGTATTATATGTTTGCTCTAGTTTTTGTTGTTTTTGATGTTGAAACCGTTTTTCTTTATCCATGGGCAATGAGTTTTGATGTATTGGGGGTATCCGTATTTATAGAAGCTTTCATTTTCATGCTTATCCTAATTGTTGGTTCAGTTTATGCGTGGCGAAAAGGAGCATTAGAGTGGTCTTAG

>atpE

ATGACCTTAAATCTTTGTGTACTGACCCCGAATCGAATTGTTTGGGATTCAGAAGTGAAAGAAATCATTTTATCGACTAATAGTGGACAAATAGGCGTATTACCAAATCACGCGCCTATTGCCACAGCTGTAGATATAGGTATTTTAAGAATCCGCTTTAACGACCAATGGGTAACGATGGCTCTGATGGGTGGTTTTGCTAGAATAGGGAATAATGAGATCACTATTTTAGTAAATGATGCGGAGAAGAGTAGTGACATTGATCCCCAAGAAGCCCAGCAAACTCTTGAAATAGCGGAAGCTAATTTGAGGAAAGCTGAAAGCAAGAGACAAACAATTGAGGCAAATCTAGCTCTCAGACGAGCTAGGACACGAGTAGAGGTTATCAATGCGATTTGA

>atpB

ATGAGAATAAATCCTACTACTTCCGGCCCTGCGGTTTCCGCGCTTGCAAAAAAAAACCTGGGACATATCGCTCAAATCATTGGTCCGGTACTGGATGTAGCCTTTCCCCCCGGCAAGATGCCTAATATTTACAATGCTCTGGTAGTTAAGGGTCGAGATCCTGTCGGTCAACCAATTAATGTGACTTGCGAGGTACAGCAATTATTAGGGAATAATCGGGTTAGAGCTGTAGCCATGAGTGCTACAGATGGTCTAACGAGAGGGATGGAAGTGATTGACACGGGAGCTCCTCTAAGTGTTCCAGTCGGCGGAGCGACTCTAGGACGAATTTTCAACGTGCTTGGAGAGCCTGTTGATAATTTAGGTCCTGTAGATACTCGCACAACATCCCCTATTCATAAATCCGCGCCTGCCTTTATACAGTTAGATACAAGATTATCTATTTTTGAAACAGGAATTAAAGTAGTAGATCTTTTAGCTCCTTATCGTCGGGGAGGAAAAATCGGACTATTCGGGGGGGCTGGAGTGGGTAAAACAGTACTCATTATGGAATTGATCAACAACATTGCCAAAGCTCATGGGGGTGTATCCGTATTTGGGGGAGTCGGTGAACGTACTCGTGAAGGAAATGATCTTTACATGGAAATGAAAGAGTCTGGAGTAATTAATGAACAAAATATTGCGGAATCTAAAGTGGCTCTAGTCTACGGTCAGATGAATGAACCGCCAGGAGCTCGTATGAGAGTTGGTTTGACAGCCCTAACTATGGCAGAATATTTCCGAGATGTTAATGAACAAGACGTACTTCTATTTATCGACAATATCTTCCGTTTCGTCCAAGCGGGATCCGAGGTATCCGCCTTATTGGGTAGAATGCCTTCTGCTGTGGGTTATCAACCTACCCTTAGTACCGAAATGGGTTCTTTACAAGAAAGAATTACTTCTACCAAAGAGGGGTCCATAACTTCTATTCAAGCAGTTTATGTACCCGCAGATGATTTGACTGACCCCGCTCCTGCCACGACATTTGCCCATTTAGATGCTACTACCGTACTATCAAGAGGATTAGCTGCTAAAGGTATCTATCCAGCAGTTGATCCTTTAGACTCAACGTCAACTATGCTCCAACCTCGGATCGTTGGCGAGGAACATTATGAAACTGCGCAAAGAGTTAAGCAAACTTTACAACGTTACAAAGAACTTCAGGACATTATAGCTATCCTTGGGTTGGACGAATTGTCCGAAGAGGATCGTTTAACCGTAGCAAGAGCGCGAAAAATTGAGCGTTTCTTATCACAACCCTTTTTCGTAGCAGAAGTATTTACCGGTTCCCCGGGGAAATATGTTGGTCTAGCAGAAACTATTAGAGGGTTTAAATTGATCCTGTCCGGAGAATTAGATGGTCTTCCTGAACAGGCCTTTTATTTGGTAGGTAACATCGATGAAGTTACTGCGAAGGCTACAAACTTAGAAATGGAGAGTAATTTGAAGAAATGA

>rbcL

ATGTCACCACAAACAGAGACTAAAGCGAGTGTTGGATTCAAAGCCGGTGTTAAAGATTATAAATTGACTTATTATACTCCTGACTATGCAACCAAAGATACTGATATCTTGGCAGCATTCCGAGTAAGTCCTCAACCCGGAGTTCCACCCGAGGAAGCGGGGGCTGCGGTAGCTGCGGAATCTTCTACTGGTACATGGACAACTGTGTGGACCGATGGGCTTACCAGCCTTGATCGTTACAAAGGGCGATGCTACAACATTGAGCCCGTTGCTGGAGAAGAAAATCAATATATATGTTATGTAGCTTACCCGTTAGACCTTTTTGAAGAAGGTTCTGTTACTAACATGTTTACTTCCATTGTGGGTAATGTATTTGGTTTCAAAGCCCTGCGCGCTCTACGTCTAGAGGATCTACGAATCCCTACCGCGTATACTAAAACTTTCCAAGGCCCGCCTCACGGCATCCAAGTTGAGAGAGATAAATTGAATAAGTATGGACGTCCCCTGTTGGGATGTACTATTAAACCTAAATTGGGGTTATCCGCTAAGAATTACGGTAGGGCAGTTTATGAATGTCTACGCGGTGGACTTGACTTTACCAAAGATGATGAGAACGTGAACTCCCAACCATTTATGCGTTGGAGGGACCGTTTCTTATTTTGTGCGGAAGCAATTTATAAAGCGCAAGCTGAAACAGGTGAAATCAAAGGTCATTACTTGAATGCTACTGCAGGGACATGCGAAGAAATGATGAAAAGGGCTATCTTTGCCAGAGAGTTGGGAGTTCCTATCGTAATGCATGACTACTTAACAGGGGGATTCACCGCAAATACTAGCTTGGCTCATTATTGCCGAGATAATGGTCTACTTCTTCACATCCACCGTGCAATGCATGCAGTTATTGATAGACAGAAGAATCATGGTATACACTTTCGTGTACTAGCTAAAGCTTTACGTATGTCTGGTGGAGATCATATTCACGCTGGTACAGTAGTAGGTAAACTTGAAGGGGAAAGAGACATAACTTTGGGATTTGTTGATTTACTACGTGATGATTTTATTGAAAAAGATAGAAGCCGCGGTATTTATTTCACTCAAGATTGGGTCTCTCTACCAGGTGTTCTGCCCGTGGCTTCCGGAGGTATTCACGTTTGGCATATGCCTGCTTTGACCGAGATCTTTGGAGATGATTCCGTACTACAATTTGGTGGAGGAACTTTAGGACACCCTTGGGGAAATGCACCCGGCGCCGTAGCTAATCGAGTAGCTCTAGAAGCATGTGTACAAGCTCGTAATGAAGGACGCGATCTTGCTCGCGAAGGTAATGAAATTATCCGGGAGGCGAGCAAATGGAGTCCTGAACTGGCTGCTGCTTGTGAAGTATGGAAGGAGATCAAATTTGAATTCCCAGCAATGGATACTTTGTAA

>accD

ATGAAAAAAAAAAAGGGGTGGTTCAATTCGATGTTATCTAAGGGTAAGGAGGAATTAGAATACAGGTGTGGGTTAAGTAAATCAATGGATAGTCTTGGTCCTATTCAAAATACCAGTGTAAGCGAGGACCCGATTCGAAATGATAAGGATAAAAACATTCATAGTTCGAGGGATAGTGACTGTTCGAGTTACAGCAATTTAGCTGGTGTCAGGGACATTCGTAATTTCATCTCGGATGACACCTTTTTTATTAAGGATAGTAATAGGGACAGCTATTCCATATATTTTGATATTGAAAATCAAATTTTGGAAATAGACAACGATCATTCTTTTCTAAGTGAACTAGAAAGTTCTTTTTATAGCTTTCGTAATTATAGTTCTAGGAATAATGGATCCAAAAGTGATGATCCCGACTCTGATCGTTACATGTATGATACTCAATCGAGTTGGAATAATTACATTCATAATTGCCTCGACTCTTATCTTCATTCTCAAATCTGTATTGATAGTCACATTTTAAGTAGTAGTGACTATTATAGTGCCAGTTACATTTCTAATTTCATTTCTAGTGAAAGTGGAAATAGTAGTGAAAGCGAGAGTTCCAATATACAAAGTAGTAGTGACTATTATAGTGCCAGTTACATTTCTAGTGAAAGTGGAAATAGTAGTGAAAACGAGAGTTCCAATATACAAAGTAGCACGAATGGTAGTGATTTAACTATAAGCGAAAGTTCTGATGATCTCGATGTAACTCAAAAATACAGGCATTTATGGGTTCAATGCGAAAATTGTTATGGATTAAATTATAAGAAATTTCTTAAGTCAAAAATGTATATTTGTGAACAATGTGGATTTCATTTGAAAATGAGTAGCTCAGATAGAATCGAACTTTCGGTTGATCCAGGTACTTGGGATCCGATGGATGACGACATGGTCTCTATAGATCCCATTGAATTTCATTCAGAAGAGGAACCTTATAAAAATCGTATTGATTCTTATCAAACAAAGACAGGATTAACAGAGGCTGTTCAAACAGGTACAGGGCAACTAAACGGGATTCCCATCGCAATTGGGGTTATGGATTTTCAGTTTATGGGGGGTAGTATGGGATCCGTAGTAGGCGAGAAAATCACCCGTTTGATCGAGTATGCTGCCAATAAATTTTTACCTCTTCTTCTAGTGTGTGCTTCCGGGGGAGCACGCATGCAAGAAGGAAGTTTGAGCTTGATGCAAATGGCTAAAATATCTTCTGCTTTATATGATTATCAATTAAATAAAAAGTTATTCTATGTATCAATTCTTACATCTCCTACTACTGGTGGAGTGACAGCTAGTTTTGGTATGTTGGGGGATATCATTATTGCTGAACCTAATGCCTATATTGCATTTGCGGGTAAAAGAGTAATTGAACAAACATTGAATAAGACAGTACCTGAAGGTTCACAAGAGGCTGAATATTTATTCCATAAGGGCTTATTCGATCCAATCGTACCACGTAATCCTCTAAAAGGTGTTCTGAGCGAGTTATTTCAGTTCCACGCCTTTTTTCCTTTGAATCAAAATTAA

>psaI

ATGACAATTCTCAACAGCTTTCCCTCAATTTTTGTGCCTTTAGTGGGCCTAGTATTTCCGGCAATGGCAATGGCTTCTTTATTTCTTTATCTTGAAAAAAATAAGATTTTTTAA

>ycf4

ATGAGTTGGCGATCAGAATATCTATGGATAGAATTTATAGCAGGCTCTCGCAAAACAAGCAATTTCTGCTGGGCTCTTATCCTTTTTTTAGGTTCATTAGGATTCTTAGTGGTTGGAATTTCGAGTTATTTTGATAGGAATTTGCTATCTTTATTTCCGTCTCAGCAAATCAATTTTTTTCCACAAGGGATCGTGATGTCTTTCTACGGGATCGCGGGTCTCTTTATTAGTTCCTATTTGTGGTGCACAATTACATGGAATGTAGGTAGCGGTTATGATCGATTTGATACAAAAGAGGGAATAGTGTGTATTTTTCGTTGGGGATTTCCTGGAAAAAATCGCCGCATCTTTCTACGATTCCTTATGAAAGATATTCAGTCCATCAGAATAGAAGTTAAAGAGGGTATTTATGCTCGTCGTGTCCTTTATATAGAAAGCAGAGGCTTGGGGGCCATTCCCTTGAATCGTACTGATGAGAATTTGACTCCACGAGAAATTGAGCAAAAGGCTGCGGAATTGGCCTATTTCTTGCGTGTACCAATTGAAGGGTTTTGA

>cemA

ATGAAAAAAAAGAATGCATCCATTCCCCTTAGATATCTTTCATCTATAGTATTTGTAGTATTTTTGCCCTGGTGGATCCCTCTCTCATTTAATAAAAGTTTGGAATCCTGGGTTACTAATTGGTGGAATACTAGTCAACCCGAAACCTTTTTGAATGATATTCGGGAAAAGGCTATTCTAGAAAAATTCATAGAATTAGAGGAATTATTCCTCTTGGACGAAATGGAAATGATAAAGGAATTTCCGGAAAGACATCTAGAAAAGCTTCGCATAGGGCTCCAGAAAGAAACAATCCAATTAATCAAGATGCACGATGAGGATCATATCCATACGATTTTTCACTTCTCGACAAATACAATCTGCTTTGTTATTCTAAGTGGTTATTCGATTCTGTGTAATGAAGAACTTTTTATTCTTAACTCTTGGGTTCAAGAATTCCTATATAATTTAAGCGACACAATAAAAGCCTTTTCGATTCTTTTCGTAACTGATTTATGTATCGGATTCCATTCGCCCCGCGGTTGGGAACTACTGATTGGCTATGCCTACAACGATTTTGGATTTGCTCATAATGATATTATTCTATCTGTTCTTGTTTCCACTTTTCCAGTCATTCTAGATACGTTTTTTAAATATTGGCTTTTTTCTTATTTAAATCGTGTATCTCCGTCACTTGTAGTGATTTATCATTCAATGACTGAGTGA

>petA

ATGCAAATTAGAAATACCTTTTCTTCGTTAAAGGGCGAGATTACTCGATTCATTTCCGTATCCCTCATGATATATATAATAACTCGGGCATCAATTTCAAATGCATATCCCGTTTTTGCGCAGCAGGGTTTTGAAAATCCACGAGAGGCAACTGGTCGGATTGTATGCGCCAATTGTCATTTAGCTAATAAGCCCGTGGATATTGAGGTTCCACAGGCGGTACTCCCTGATACTGTATTTGAAGCAGTTGTTAGAATTCCGTATGATATGCAACTGAAACAAGTTCTTGCTAATGGTAAAAGGGGGTCTTTGAATGTGGGGGCCGTTCTTATTTTACCAGAGGGGTTTGAATTAGCCCCCTCCGACCGTATTTCGCCCGAGATGAAAGAAAAGATAGGCAAGCTGTCTTTTCAGACCTACCGACCTACTAAAAAAAATATTCTTGTGATAGGGCCAGTTCCTGGTCAGAAATATAGTGAAATCACTTTTCCTATTCTTTCCCCGAACCCTGCGACTAATAAGGATGCTCACTTCTTAAAATATCCAATATATGTAGGTGGGAACAGGGGGAGGGGTCAGATTTATCCCGACGGGAACAAAAGTAACAATACGGTTTATAATGCTACAGCTGCGGGTATAGTAAGCAAACTCTTACGAAAAGAAAAAGGGGGATACGAAATAACCATAACGGATGCATCGAATGGGCGTGAAGTGGTTGATATTATCCCTCCAGGCCCAGAACTTCGTGTTTCAGAGGGACAATCTATCAAACTTGATCAACCATTAACAAGTAATCCTAATGTAGGTGGGTTTGGTCAGGCAGATGCAGAAATAGTACTTCAAGATCCATTACGTGTCCAAGGCCTTTTGTTCTTTTTGGCATCTGTTGTTTTGGCACAAATCTTTTTGGTTCTTAAAAAGAAACAGTTTGAGAAGGTCCAATTGTCCGAAATGAATTTCTAG

>psbJ

ATGGCCGATACTACTGGAAGGATTCCCCTTTGGATAATAGGTACTGTAACTGGTATTCCTGTGATCGGTTTAATAGGCATTTTCTTTTATGGTTCATATTCCGGATTAGGTTCGTCCTTGTAG

>psbL

ATGACACAATCAAACCCGAACGAACAAAATGTTGAATTGAACCGTACCAGTCTCTACTGGGGGTTATTACTCATTTTTGTACTTGCTGTTTTATTTTCCAATTACTTCTTCAATTAA

>psbF

ATGACCATAGATCGAACCTATCCAATTTTTACAGTGCGATGGTTGGCCGTGCACGGACTAGCTGTACCTACCGTTTCTTTTTTGGGGTCAATATCAGCAATGCAGTTCATCCAACGATAA

>psbE

ATGTCTGGAAGCACAGGAGAACGTTCTTTTGCTGATATTATTACCAGTATTCGATACTGGGTCATTCATAGCATTACTATACCTTCCCTATTCATTGCGGGTTGGTTATTCGTCAGCACGGGGTTAGCTTACGATGTGTTTGGAAGCCCTCGTCCAAACGAGTATTTTACAGAGAGCCGACAAGGAATTCCATTAATAACCGGCCGTTTTGATTCTTTGGAACAACTCAACGAATTTAGTAGATCTTTTTAG

>petG

ATGATTGAAGTCTTTCTATTTGGAATCGTCTTAGGTCTAATTCCTATTACTTTGGCGGGATTATTCGTAACCGCATATTTACAATACAGACGTGGTGATCAGTTGGACCTTTGA

>psaJ

ATGCGAGATCTAAAAACATATCTTTCCGTGGCACCGGTACTAAGTACTCTATGGTTCGGGTCTTTAGCAGGGTTATTGATAGAAATCAACCGTTTATTCCCGGACGCATTGACATTTCCTTTTTTTTCATTCTAG

>rpl33

ATGGCCAAGGGTAAAGATGTCCGAGTAAGGGTTATTTTGGAATGTACTAGTTGTGTTCGAAACGGTGTTAATAAGGAATCAAGGGGTATTTCCAGATATATTACTCAAAAGAATCGACACAATACACCTAGTCGATTGGAATTGAGAAAATTCTGTCCCTATTGTTACAAACATACACTTCACGGGGAGATAAAAAAATAG

>rps18

ATGGATAAAACCAAGCGACTCTTTCTTAAATCCAAGCGATCTTTTCGTAGGCGTTTGCCCCCGATCCAATCGGGGGATCGAATTGATTATAGAAACATGACTTTAATTAGTCGATTTCTTAGTGAACAAGGAAAAATATTATCTAGACGGGTGAATAGATTGACCTTAAAAGAACAACGATTAATTACTATTGCTATAAAACAAGCTCGTATTTTATCTTCGTTACCTTTTCTTAATAATGAGAAACGATTTGAAAGAAGTGGGTCGACCACTAGAACTCCAGGTCTTCGAACCAGAAAAAAATAG

>rpl20

ATGACCAGAATTAGACGAGGATATATAGCTCGGAGACGTAGAACAAAAATGCGTTTATTTGCATCAAGCTTTCGCGGGGCTCATTCAAGACTTAGTCGAACAATTACTCAACAGAAAATAAGAGCTTTGGTTTCGGCTCATCGTGATAGAGATAGGAAAAAAAGAGATTTTCGTCGTTTGTGGATCACTCGAATAAATGCAATAATTCGCGGAAATAGGGTATCCTATATTTATAGTAGATTAATAAACGATTTGTATAAGGCGCAGTTGGTTCTTAATCGTAAGATACTTGCACAAATAGCTATATCAAATAGGAATTGTCTTTATATGATTTCCAATGAGATCATAAAATAA

>rps12

ATGCCAACTATTAAACAACTTATTAGAAACCCAAGACAGCCAATCAGAAACGTTACAAAATCCCCCGCTCTTGGGGGATGCCCTCAGCGCCGAGGAACATGTACAAGGGTGTATGTGCGACTCGTTACTATCACCCCCAAAAAACCAAACTCTGCCTTACGTAAAGTTGCCAGAGTACGATTAACCTCTGGGTTTGAAATCACTGCTTATATACCGGGTATTGGCCATAATTCACAAGAACATTCTGTAGTCTTAGTAAGAGGGGGGAGGGTTAAGGATTTACCCGGTGTGAGATATCACATTGTTCGAGGAACCCTAGATGCTGTCGGAGTAAAGGATCGTCAACAAGGGCGTTCTAGTGCGTTGTAG

>clpP

ATGCCTATTGGTGTTCCAAAAGTACCTTATCGAAGTCCCGGGGACAAGCATCCATCTTGGGTTGACATAAACCGACTTTATCGAGAAAGATTACTTTTTTTAGGTCAAATGGTTGAGAGTGATATCTCGAATCAACTTATTGGTATTATGGTATATCTCAGTATCGAGAACGAGACCAAGGATTTGTATTTATTTATCAACTCTCCTGGCGGATGGGTAATACCCGGAATAGCAATTTATGATACTATGCAATTTGTGCGACCAGATGTACAGACAATATGCATGGGATTGGCCGCCTCCATGGGGTCTTTTCTCCTGGCTGCAGGGGCAAGTACCAAACGTCTAGCATTCCCTCACGCTAGGGTCATGATCCATCAACCTATTGGCGCTTTTTATGGGGCACAAGCGGGAGAATTTATCCTGGATACGGAAGAACTACTGAGACTGCGCGAAATCCTTACAATGGTTTATGTACAAAGATCGGGCAAGCCCTTATGGGTTGTATCCGAAGACATGGAAAGGGATACTTTTATGTCAGCAACAGAAGCCCAAGCTTATGGAATTGTTGATCTTGTAGCGGTTGGATAA

>psbB

ATGGGTTTGCCTTGGTATCGTGTTCATACCGTCGTATTGAATGATCCCGGTCGTTTGATTTCTGTCCATATAATGCATACAGCCCTAGTTGCGGGTTGGGCCGGTTCAATGGCTCTATATGAATTAGCTGTTTTTGATCCCTCCGATCCAGTTCTTGATCCAATGTGGAGACAAGGCATGTTCGTTATACCCTTCATGACTCGTTTAGGAATAACCGATTCATGGGGCGGTTGGAGTATTACGGGGGGGACGGTAACGAATCCGGGTATTTGGAGTTACGAAGGTGTAGCCGGGGCACATATTGTGTTTTCGGGCTTGTGCTTCTTGGCAGCTATCTGGCATTGGGTGTATTGGGATCTAGCAATATTTGTCGATGACCGTACGGGAAAACGCTCTTTGGATTTGCCTAAAATCTTTGGAATTCATTTATTTCTCTCAGGAGTGGCTTGCTTTGGTTTTGGGACATTTCATGTAACAGGATTGTATGGTCCTGGAATATGGGTGTCCGACCCTTATGGACTAACTGGAAAGGTACAATCTGTAAATCCAGCATGGGGTGTGGAAGGTTTTGATCCTTTTGTTCCAGGAGGAATAGCCTCTCATCATATTGCAGCAGGGACATTGGGCATATTAGCAGGCTTATTCCATCTTAGTGTCCGCCCACCTCAACGCCTATACAAAGGATTACGTATGGGCAATATTGAAACCGTTCTTTCCAGCAGCATCGCTGCTGTCTTTTTTGCAGCGTTTGTTGTTGCTGGAACTATGTGGTATGGTTCAGCAACTACCCCCATCGAATTATTTGGTCCCACCCGTTATCAATGGGATCAGGGATACTTTCAGCAAGAAATATATCGAAGAGTCAGTGCTGGGCTAGCCGAAAATCAAAGTTTATCAGAAGCTTGGTCTAAAATTCCTGAAAAATTAGCTTTTTATGATTACATCGGAAATAATCCTGCGAAAGGGGGATTATTCAGAGCGGGTTCAATGGATAACGGGGATGGAATAGCTGTCGGGTGGTTAGGACACCCTATCTTTAGAGATAAAGAAGGGCGTGAACTTTTTGTACGTCGTATGCCTACCTTTTTTGAAACATTTCCAGTTGTTTTGGTAGACGGAGATGGAATTGTTAGAGCCGACGTGCCTTTTCGAAGGGCAGAATCGAAGTATAGTGTCGAACAAGTAGGTGTAACTGTTGAGTTCTATGGTGGCGAACTGAATGGAGTGAGTTATAGTGATCCTGCTACTGTGAAAAAATATGCTAGACGTGCTCAATTGGGTGAAATTTTTGAATTAGATCGTGCTACTTTGAAATCCGATGGTGTTTTTCGTAGCAGTCCAAGGGGCTGGTTTACTTTTGGACACGCTTCATTTGCTCTGCTTTTCTTCTTCGGACACATTTGGCATGGTGCTAGAACCTTGTTCAGAGATGTTTTTGCTGGTATTGACCCGGATTTGGATGCTCAAGTGGAATTTGGAGTATTCCAAAAACTTGGAGATCCAACTACAAGAAGACAAGTAGTCTGA

>psbN

ATGGAAACAGCAACCCTAGTCGCCATCTCTATATCTGGGTTACTTGTAAGTTTTACTGGGTACGCCTTATATACTGCTTTTGGGCAACCCTCTCAACAACTAAGAGATCCATTCGAGGAACACGGGGACTAG

>psbH

ATGGCTCCACAAACAGTTGAGGGTAGTTCTAGAGCTCGTCCAAAAATGACTTCTGCAGGGGGGTTATTGAAACCTTTGAATTCGGAATATGGTAAAGTAGCTCCTGGATGGGGAACTGCTCCTTTGATGGGTATCGCAATGGCTCTATTTGCGATATTTCTGTCTATTATTTTGGAGATTTATAATTCGTCCGTTTTACTGGACGGAATTTCAATGAATTAG

>petB

ATGAGTAAAGTCTACGATTGGTTCGAAGAACGTCTCGAGATTCAGGCGATTGCAGACGATATAACTAGTAAATACGTTCCTCCTCATGTCAACATATTTTATTGTCTAGGAGGAATTACGCTTACTTGTTTTTTAGTACAAGTAGCTACAGGGTTTGCTATGACTTTTTACTACCGTCCGACCGTTACTGAGGCTTTTGCTTCTGTTCAATACATAATGACGGAAGCTAACTTTGGTTGGTTAATCCGATCAGTTCATCGATGGTCGGCAAGTATGATGGTCCTAATGATAATCCTGCACGTATTTCGTGTGTATCTCACTGGCGGTTTTAAAAAACCTCGCGAATTGACTTGGGTTACAGGCGTGGTTCTGGCTGTATTGACCGCATCCTTTGGTGTAACTGGTTATTCTTTACCTTGGGACCAAATTGGGTATTGGGCAGTCAAAATTGTAACAGGCGTGCCAGAAGCAATTCCGGTAATAGGATCGCCTTTGGTAGAGTTATTACGCGGAAGTGCTAGTGTGGGACAGTCCACTTTGACTCGTTTTTATAGTTTACACACTTTTGTATTACCTCTTCTTACTGCCGTATTTATGTTAATGCATTTCCTAATGATACGTAAACAAGGTATTTCTGGCCCTTTATAA

>petD

ATGGGAGTAACAAAAAAACCTGACTTGAATGATCCTGTATTAAGGGCTAAGTTGGCTAAAGGTATGGGTCATAATTATTATGGCGAACCCGCATGGCCCAACGATCTTTTATATATTTTTCCAGTAGTAATTCTAGGTACTATTGCATGTAACGTAGGCTTAGCGGTTCTAGAGCCATCAATGATTGGTGAACCCGCGGATCCATTTGCAACTCCTTTGGAAATATTACCCGAATGGTATTTCTTTCCCGTATTTCAAATACTTCGTACAGTACCCAATAAGCTGTTGGGTGTTCTTTTAATGGTTTCAGTACCTACGGGATTATTAACAGTACCTTTTTTGGAAAATGTTAATAAATTCCAAAATCCATTTCGCCGCCCAGTAGCGACAACCGTCTTTTTGATTGGTACCGCAGTGGCCTTGAGCTTGGGTATTGGAGCAACATTACCTATTGAAAAATCCCTAACTTTAGGTCTTTTTTAA

>rpoA

ATGGTTCGAGAGAAAGTAAAAGTATCTACTCGGGCACTACAGTGGAAGTGTGTTGAATCAAGAGCAGACAGTAAGCGTCTTTATTATGGACGCTTTATTTTGTCTCCACTTATGAAAGGTCAAGCCGACACAATAGGCATTGCGATGCGAAGAGTTTTGCTTGGAGAAATAGAAGGAACATGTATTACACGCGCAAAATCTGAGAAAATCCCACATGAATATTCTACCATAGTGGGTATTCAAGAATCGGTACATGAAATTTTAATGAATTTGAAAGAAATTGTATTGAGAAGTAATCTTTATGGAACTTGTGACGCGCTTATTTGTGTCAAAGGTCCGGGAGATGTAACTGCTCAAGACATCCTCTTGCCACCTTCTGTAGAAATCGTTGATAAGACGCAGCACATAGCTAGCCTAACAGAACCAATTGATTTGTGTATTGGATTACAAATCGAGAGGAGTCGAGGATATAATATAAAAACGCCAAATAATTTTCAAGACGGAAATTGTTATCCTATAGACGCTGCATTCATGCCTGTTCGAAATGCGAATCATAGTATTCAGTCTTATGGGAATGGCAATGAAAAACAAGAGATCCTTTTTCTAGAAATATGGACAAACGGGAGTTTAACTCCTAAAGAAGCACTTCATGAAGCCTCCCGGAGTTTGATTGATTTATTTATTCCCTTTCTCCAGGCAGCAGACGAAAACTTACATTTAGAGAACAATCAATACAAGGTTACTTTACCTTTTTTTACTTTTCATGATAGATTGGCTAAACTAACGAAAAAGAAAAAAGAAATCGCATTGAAATCGATTTTTATTGACCAATCAGAATTGTCTCCCAGGATCTATAATTGTCTCAAAAAGTCCAATATACATACATTATTCGAGCTTTTGAATAAGAGTCAAGAAGACCTTATGAAAATTGAACACTTTCGCCTAGAAGATGTAAAGCAGATAATGGGTATTCTAGAAAATAAATAG

>rps11

ATGGCAAAATCTACACCAAGAAGTGGTTCACGTAGGGCTGGACGGATGGGTTCGCGTAAAAGTGGACGTCGAATACCAAAGGGCGTTATTCATGTTCAAGCAAGTTTCAACAACACCATTGTGACTGTTACGGATGTACGGGGTCGGGTAATTTCTTGGTCCTCGGCCGGTACTTGTGGATTCAGGGGTACAAGAAGAGGTACGCCCTTTGCTGCTCAAACCGCAGCAGGAAGTGCTATTCGAGCAGTAGCGGATCAAGGTATGCAACGAGCAGAAGTCATGATAAAGGGTCCTGGTCTCGGAAGAGATGCAGCATTACGAGCTATTCGTAGAAGCGGTATCCTTTTAAATTTCGTACGGGATGTAACCCCTATGCCACATAATGGTTGCAGACCCCCTAAAAAAAGACGGGTGTAG

>rpl36

ATGAAAATAAGGGCTTCCGTTCGTAAAATTTGTGAAAAATGTCGACTGATCCGCAGGAGGGGACGGATTATAGTAATTTGTTCCAACCCGAGACATAAACAAAGACAAGGATAA

>rps8

ATGGGCAAAGACACTATTGCTGACATAATAACTTCTATACGAAATGCTGACATGAATCGAAAGGGAACAGTTCGAATAGCATCTACTAGCATCACCGAAAACATTGTTAAAATACTTTTGCGAGAGGGTTTTCTAGAAAACGTAAGGAAACTCGTGGAAAACAAAAAAGAGTTTTTGGTTTTAACCCTACGACATAGAAGGAATAGGAAAGGGCCATATAGACCCATTTTAAATTTAAAACGAATCAGTCGACCCGGTCTACGAATCTATTTTAACTATCAACGAATTCCTAGAATTTTAGATGGGATGGGGATTGTAATTCTCTCTACTTCTCAGGGTATAATGACAGACCGAGCGGCTCGACTAGAAAGAATCGGCGGAGAGGTTTTGTGTTATATATGGTAA

>rpl14

ATGATTCAACCTCAAACCCATTTGAATGTAGCGGACAATAGCGGTGCCCGAGAATTGATGTGTATTCGAATCATAGGAGCCAGTAATCGTAGATATGCTCATATTGGTGACGTTATTGTTGCTGTGATCAAGGAAGCAGTACCAAATACGCCTCTAGAAAGATCAGAAGTGATCAGAGCTGTAATTGTACGTACTTGTAAAGAACTCAGACGTGATAACGGTATGATAATACGTTATGATGACAATGCTGCAGTTGTCATTGATCAAGAAGGAAATCCAAAGGGAACTCGAGTTTTTGGTGCGATCGCCCGAGAATTGAGACAGTTGAATTTTACTAAAATCGTTTCATTAGCACCTGAAGTATTATAA

>rpl16

ATGCTTAGTAACCCCAAAAGAACAAGATTCCGTAAACAACATAGAGGAAGAATGAAAGGAATATCTTATCGAGGTAATCATATTTGTTTCGGCAGATATGCTCTTCAAGCACTTGAACCCGCTTGGATCACATCTAGACAAATCGAAGCAGGGCGCCGAGCAATGACACGAAATGTACGGCGTGGCGGAAAAATATGGGTACGTATATTTCCAGACAAACCAGTTACAGTAAGACCCACGGAAACCCGTATGGGGTCCGGGAAAGGATCCCCCGAATATTGGGTAGCCATCGTTAAACCGGGTAGAATACTTTATGAAATGAGTGGAGTCGCTGAAAATATCGCTCGAAAGGCTATTTCAATAGCGGCGTCAAAAATGCCTATAAGAACTAAATTCATTATTTCTGGATAG

>rps3

ATGGGACAAAAAATAAATCCACTTGGTTTCCGACTTGGTACAACACAAAGTCATCATTCGCTTTGGTTTGCAAAACCAAAAAGTTATTGCGAAGGTCTACAAGAGGATCAAAAAATACGAAACGTTATTAAGAATTATGTACAAAAAAATATGAGAATATCCTCTGGTGTTGAGGGAATTGCCCGGATAGAGATTCAAAAAAGAATTGATCTAATTCAAGTCATAATCTATATAGGATTCCCAAAATTATTACTAGAAAATAGACCGCGAAGAATTGAAGAATTACAGATGAATGTACAAAAAGAACTTAATTGTGTGAACCGAAAAATAAACATTGCTATTACAAGAATTACAAATCCTTATGGACACCCCAATATTCTTGCCGAATTTATAGCGGGCCAATTAAAAAATAGAGTTTCTTTTCGCAAAGCAATGAAAAAAGCTATTGAATTAACTGAACAGGCAGATACAAAAGGAATTCAAGTCCAAATTGCAGGGCGTCTTGACGGAAAAGAAATTGCGCGCGCCGAATGGATCAGAGAAGGTAGAGTTCCTCTACAAACCATTGGAGCTAAAATTGATTATTGTTCCTATACAGTTCGAACTATATACGGGGTATTAGGAATCAAAATTTGGATATTTGTAGACGAAGAAAAATAA

>rpl22

ATGATAAGGATAATAAAGAAGAAGGTAGAAGTATCTGCTTTAGGTCAACATATATGTATGTCTGCTCACAAAGCCCGAAGGGTAATTGATCAGATTCGTGGACGTTCTTACGAGGAAACCCTTATGATACTCGAACTCATGCCTTATCGAGCATGTTATCCCATTTTAAAATTGGTTTATTCTGCAGCAGCAAATGGTATTCACAATCTGGGTTTCAACGAAGCGAGTTTATTCATTATTAAAGCCGAAGTAAACGAGGGTACTGCGGCGAAAAGATTAAAACCTCGAGCTCGAGGGCGGAGTTATATGATCAAAAGACCCACTTGTCATATAACTATTGTTTTAAAAGATATCTCCTTAGATGAATATGAATATAGGGACTATCTCGACTGCTCAAAAAAAACTGGATTAATAAATAAAATAAAAAAAAAAAAGAACAAAACTATGACATGTCATGATACATATACATATAGGAGTGGGGGATTATGGGACAAAAAATAA

>ndhF

ATGGAACACCCATATCAATATTCCTGGATCATACCTTTAGTTCCACTTCCAGTCCCTATGTTAATAGGGGTGGGACTTCTACTTTTTCCGATCGCAACAAAACATCTTCGCCGTATGTGGGCTTTTCTTAGTATTTTATTGTTAAGTATAGTTATGATTTTTTCGATTGATCTATCTATTGAGCAAATAGATCGAACTTATATCTATCAATCCCTAAGGTCTTGGACCATCAATAATGATTTTTCTTTCGAGTTCGGATACTTTATTGATCCACTTACTTCTATTATGTCAATATTAATCACTACAGTTGGAATTCAGGTTCTTATTTATAGTGACAATTATATGTCTTATGATCAAGGATATTTGAGATTTTTTGCTTATATGAGTTTTTTCAATGCTTCAATGTTAGGATTAGTTACAAGTTCGAATTTCATACAAATTTATATTTTTTGGGAATTGGTTGGAATGTGCTCTTATCTATTAATAGGATTTTGGTTCACACGACCTATTGCGGCAGGCGCTTGTCAAAAAGCATTTGTAACTAATCGTGTAGGGGATTTTGGATTATTATTAGGAATCCTAGGTCTTTATTGGATAACGGGTAGTTTCGAATTTCGGGATTTGTTCGAAATATTGAATAACTTGATTTATAATAATGAGGTTAACCTTTTATTTGTTACTTTGTGTGCATTTCTATTATTTGCCGGCCCGGTTGCTAAATCCGCGCAATTCCCTCTTCATGTATGGTTACCCGATGCCATGGAAGGGCCTACTCCTATTTCGGCTCTTATCCATGCTGCTACTATGGTAGCGGCGGGAATTTTTCTTGTAGCTCGCCTTCTTCCGCTTTTCATAGTCATACCGTACATAATGAATCTAATATCTTTGTTAGGTATAATAACAGTATTTTTAGGAGCTACTTTAGCTCTTGCTCAACAAGATATTAAGAGAGGTTTAGCTTATTCTACAATGTCTCAATTGGGTTATATGATGTTAGCTCTAGGGATGGGGTCTTATCGAGCCGCTTTATTTCATTTGATTACTCACGCTTATTCCAAAGCCTTGTTGTTTTTAGGATCCGGATCAGTTATTCATTCAATGGAAGCCATTGTTGGATATTTTCCAGATAAAAGCCAGAATATGGTTCTTATGGGTGGGTTAAGAAAGCACGTGCCAATTACAAAAACCGCTTTTTTATTGGGTACCCTTTCTCTTTGTGGTATTCCGCCTCTCGCTTGTTTTTGGTCCAAAGATGAAATTCTTAATGATAGTTGGTTGTATTCGCCGAATTTCGCAATAATTGCTTTTTTCACAGCCGGATTAACCGCATTTTATATGTTTCGAATTTATTTACTTACTTTTGAAGGACCTTTCAACTTTTGCTTTCAAAATTACAGTGGCAAAAAAAGCAATTCCTTCTATTCAATATCTCTATGGGGTAAAGAAGAACCAAAACCAATAAAAAAAAAATTTCATTTAGTTGCTTTATTAACAATGAATAATAATGAAAGGGCCTCTTTTTTTTCGCAGAAGGCTCATCGAATTGATAGGAATGTAACAAATACGCCTTTTCTGACTATTTTTCCTTTTGGCGCTACCAAGACTTTTTGTTATCCTCACGAATCAGACAATACTATGTTATTTGTTATGCTTGTATTAGTCCTATTTCCTTTGTTTGTTGGAGCTATAGGAATTCCTTTGACTCAAGAAGGAATCGATTCGGATATTTTATCAAAATTGTTAACTCCGTCTATAAATCTTTTACATCAAAATTCAACTCATTTTGTTGATTGGTATGAAGTTTTGAAAAATCCAACCCTTTCCGTCAGTATAACGTATTTCGGAATCCTTCTAGCCTACTTTTTCTATAAACCCTTTTATTCATCTTTACACAATTGGAACATACTCAATTTATTTGCTAAAAGAGGACCTAAGAGAATTCTTTGGGACAAAATACTCAATTTTCTATATGATTGGTCATATAATCGTGCTTATATAGATGCCTTTTACAAAAGATCTTTAATGGAAGGGATAAGAGGATTAGCGGAACTAACGCATTTGTTCGACAGACGAGTAATTGATGGAATTGCAAATGGGGTCGGTATTACAAGTTTTTTTGTGGGGGAAGGTATAAAATATTTAGGGGGAAGTCGCATCTCTTTTTATCTCTTATTATATTTATTTTCAATCTTAATCTTTTTAATAAGTTCCTCCTTTTAA

>rpl32

ATGGCAGTTCCAAAAAAACGTACTTCTATATTAAAAAAACGTATTCGTAAGAATATTTGGAAAAAAAGGGGGTATTGGGCAGCGTTGAAGGCTTTTTCGTTAGCGAAATCCCTTTCTACTGGGAATTCAAAAAGTTTTTTTGTACAACAAATAAATAAGAAAACGTTGGAATAA

>ccsA

ATGATCTTTTCAACTTTAGAGCATATATTAACGCATATATCCTTTTCGGTCGTTTCAATTGGAATTACAATTTTTTTACTAACCTTATTAGTCGATGAAATCAGAGGACTATATGATTCATCAGAAAAGGGGATGATAGCTACCGCTTTCTGTCTAACAGGATTATTAATCACTCGTTGGATTTACTCGAGACATTTCCCATTAAGTAATTTATATGAATCATTAATCTTTCTTTCATGGAGTTTCTCCATTATTCATAGGATTTTCGATTTTCAAAATAATAAAAATCTTTTAAGTGCTATAACGGCACCAAGTGCTATTTTTACCCAAGGTTTTGCTACTTCGGGTTTTTTAACCAAAATGCATCAATCCGGAATATTAGTACCCGCTCTCCAAGTCCAGTGGTTAATGATGCACGTAAGTATGATGGTATTGGGCTATGCAGCTCTTGTATGTGGATCCTTATTATCCACGGCTCTTCTAGTCATTACATTTCGAAAAGTGATAAGGTTTTTTTTGAAAAGAAACAATTTTTTAAATGTAAAAGAGTCGTTTTGCTTCGGTAAAATTCAATACATGAACGAAAAAAGGAATGTTTTACTAAATACTTTTTCCGCTAGAAATTATTACAGGTATCAAGTGATTCAACAATTGGATCGCTGGAGTTATCGTATTATTAGTTTCGGATTTATCTTTTTAACCATAGGGATTCTTTCGGGAGCAGTATGGGCTAATGAGGCGTGGGGGTCTTATTGGAATTGGGATCCAAAAGAAACTTGGGCATTTATTACTTGGACTATATTCGGGATTTATTTACATACTCGAACAAATACAAATTTGGAAGGTGTAAATTCCGCGATTGTCGCTTCTACGGGCTTTCTTATAATTTGGGTATGCTATTTTGGAGTAAATCTATTAGGAATAGGATTACATAGTTATGGTTCATTTAATTAA

>psaC

ATGTCACATTCAGTAAAGATTTATGATACATGTATAGGGTGTACTCAATGTGTCCGAGCTTGCCCCACAGATGTATTAGAAATGATACCTTGGGACGGATGTAAAGCAAAGCAAATTGCTTCTGCTCCAAGAACAGAGGACTGTGTTGGTTGTAAGCGATGCGAATCCGCCTGTCCAACGGATTTCTTGAGTGTTCGGGTTTATTTATGGCATGAAACAACTCGAAGCATGGGTCTAGCTTATTGA

>ndhE

ATGATGCTCGAGCATGTACTTGTTTTGAGTGCCTATTTATTTTCTATTGGTATCTATGGATTGATTACGAGCCGAAATATGGTTCGGGCCCTGATGTGTCTTGAACTTATACTAAATGCAGTTAATATCAATTTCGTAACATTCTCTGATTTTTTTGATAGTCGACAATTAAAAGGAGATATTTTCTCAATTTTTGTTATAGCTATTGCAGCCGCTGAAGCAGCTATCGGATCAGCTATTGTTTCGTCAATTTATCGTAACAGAAAATCGACTCGTATCAATCAATCGACTTTGTTGAATAAGTAG

>ndhG

ATGGATTTGCCTGGACCAATACATGATTTTCTTTTAGTTTTTCTGGGATCAGGTCTTATATTAGGAGGTCTGGGAGTGGTATTATTTACCAACCCAATTTATTCTGCCTTTTCCTTGGGATTGGTTCTTGTTTGTATATCCCTATTCTATATTCTATCAAATTCCCATTTTGTAGCTGCCGCGCAGCTCCTTATTTACGTGGGAGCTGTAAATGTTTTGATCATATTTGCTGTAATGTTCATGAATGGTTCAGACTATTCCAAAGATTTTCATTTGAATCTTTGGACTGTTGGTGATGGGCTTACTTCCCTGGTTTGTACAAGTATTTTTTTTTCGCTAATCGCTACTATTCTAGATACGTCGTGGTATGGGATTATTTGGACTACACGACCCAACCAGATTATCGAACAAGATTTGATAAGTAATAGTCAACAAATTGGAATTCATTTATCAACAGACTTTTTTCTTCCATTTGAACTCGTTTCAATAATTCTTTTAGTTGCTTTGATAGGTGCAATTGCCGTGGCTCGTCAGTAA

>ndhI

ATGTTCCCTATGGTAACTGGTTTCATGAATTATGGTCAACAAACAATACGAGCTGCAAGGTACATTGGTCAAAGTTTCATGATTACTTTATCCCAAGCAAATCGTTTACCTGTAACTATTCAATATCCTTATGAAAAATTAATCACATCGGAGCGTTTTCGCGGTAGAATCCATTTTGAATTTGATAAATGTATTGCTTGTGAAGTATGCGTTCGCGTATGTCCTATAGATCTGCCTGTTGTTGATTGGAAATTTGAAACAGATATTCGAAAGAAACGATTGCTTAATTACAGTATTGATTTTGGAATTTGTATTTTTTGTGGTAACTGCGTTGAGTATTGTCCAACAAATTGTTTATCAATGACTGAAGAATATGAACTTGCTACTTACGACCGTCACGAATTGAATTATAATCAAATTGCTTTAGGTCGTTTACCAATGTCAGTGATTGACGATTTTACAATTCGAACAGTCTTGAATTCGCCTCAAAGAAAAAACGGCTAA

>ndhA

ATGATAATTGATACAACAGAAGTACAAGATATCAATTCTTTTTCCAGATTGGAATTCCTACAAGAGGTCTATGGGATCGTGTGGGCTCTTGCCCCTATTTCGACTCCTGTAGTGGCAATCACAATAGGTGTCCTAGTAATTGTGTGGTTAGAAAGAGAAATATCTGCAGGAATACAACAACGTATTGGGCCTGAATACGCCAGTCCCTTGGGACTTCTTCAAGCTTTAGCAGATGGGACAAAACTACTTTTCAAAGAAAACCTTCTTCCATCTAGAGGAAATAGTAGTTTATTCAGTATTGGACCATCTATAGCAGTCATAGCAATTCTACTAAGTTATTCAGTAATTCCTTTTAGTTATAACTTTGTTTTAGCTGACCTCAATATCGGTATTTTTTTATGGATTGCCATTTCAAGTATTGCCCCTATTGGACTTCTTATGTCAGGATATGGATCAAATAATAAATATTCTTTTTTAGGTGGTTTGCGAGCTGCTGCTCAATCGATTAGTTATGAAATACCATTAACTTTATGTGTTTTATCAATATCTCTATTATCTAACAGTTCAAGTACAGTTGATATAGTTGGGGCGCAATCAAAATATGGTTTTTGGGGGTGGAATTTGTGGCGTCAACCTGTAGGGTTTATCGTTTTTCTAATTTCTTCCCTAGCGGAATGCGAGAGATTACCTTTTGATTTACCAGAAGCAGAAGAAGAACTAGTAGCAGGTTATCAAACCGAATATTCAGGAATAATTTTTGGTTTATTTTACGTTGCTTCCTATCTAAATCTATTAGTTTCCTCATTATTTGTAACAGTTCTTTACTTGGGGGGTTGGAATCTTTCCATTCCACACATATTTGTTCCTGAGCTATTTGAAATAAATAAAGCGGATGGAATCTTTGGAACGACAATTGGTATCTTTATTACATTAGCTAAAACTTATTTGTTCTTGTTCGTTCCGATTACAACAAGATGGACTTTACCGAGACTAAGAATGGACCAACTATTAAATCTTGGCTGGAAATTTCTTTTACCTATTTCTCTCGGTAATCTATTATTAACAACTTCTTCCCAACTCCTTTCGCTATAA

>ndhH

ATGAGTATACTAGCTACAGAAAAAGAATTTATGATAGTCAATATGGGACCTCACCACCCATCAATGCACGGTGTTCTTCGTCTCATCGTTACTCTAGATGGTGAAGATGTTATTGACTGTGAACCAATATTGGGTTATTTACACCGAGGGATGGAAAAAATTGCGGAAAACCGAACAATTATACAATATCTGCCTTATGTAACCCGTTGGGATTATTTAGCTACTATGTTCACTGAAGCAATAACTGTAAACGGACCCGAACTGTTGGGAAATATTCAAGTACCCAAAAGAGCCAGCTATATCAGAGTAATTATGTTGGAGTTGAGTCGTATAGCTTCCCATCTGTTATGGCTTGGCCCTTTTATGGCAGATATTGGTGCACAGACTCCTTTCTTCTATATTTTCAGAGAAAGAGAATTGGTCTATGATCTGTTCGAAGCTGCCACCGGTATGAGGATGATGCATAATTATTTTCGTATCGGAGGAATAGCGGCTGATTTACCTCATGGTTGGATAGATAAATGTTTGGATTTCTGCGATTATTTTTTAACGGGGGTTGCTGAATATCAAAAACTTATTACACGAAATCCTATTTTTTTAGAACGAGTTGAAGGAGTAGGCATTATTGGTGGAGAAGAAGCAATAAATTGGGGTTTATCCGGACCAATGCTACGAGCGTCTGGAATAGAATGGGATCTTCGTAAAGTTGATCATTATGAGTGTTATGACGAATTTGATTGGGAAGTCCAGTGGCAAAAAGAAGGAGATTCATTAGCTCGTTATTTAGTCCGAATCGGTGAAATGACGGAATCTATAAAGATTATTCAACAGGCTTTAGAAGGAATTCCGGGAGGACCCTATGAAAATTTAGAAATCCGATGTTTTGATAGAGAAAGCGATCCAGAATGGAATGATTTTGAAGATCGATTCATTAGTAAAAAGCCTTCTCCCACCTTTGAATTGACGAAACAAGAACTTTATGTGAGAGTAGAAGCCCCAAAAGGAGAATTGGGAATTTTTCTGATAGGAGATCAAAGTGGTTTTCCTTGGAGATGGAAAATTCGCCCACCGGGTTTTATCAATTTGCAAATTCTTCCTCAGTTAGTTAAAAGAATGAAATTGGCTGATATTATGACAATATTAGGTAGTATAGATATCATTATGGGGGAAGTTGATCGTTGA

>rps15

ATGATAAAAAATTCATTCGTCTCAGTTATGGTTCAAGAAAAAAAAGAAGAAAACTGTGGATCGGTTGAATTTCAAGTATTCCGTTTCACTAATAAGATACGGAGACTTACTTCACATTTAGAATTGCACAGAAAAGACTATTTATCTCAAAGGGGTCTACGAAAAATTTTGGAAAAACGCCAACGTCTACTAGCTTATTTGTCAAAGAAAAATAGAGTACGTTATAAGGAATTAATTAGTAAGTTCAATATTCGGGAGTCAAAAAATCGTTAA

>ycf1

ATGATTTTTCAATCTTTTATACTAGGTAATCTAGTATCCTTATGCATGAAGATAATCAATTCGGTCGTTGTGGTCGGACTCTATTATGGATTTATGACCACATTCTCCATAGGGCCCTCTTATCTCTTCCTTCTCCGAGCTCGGGTTATGGAAGAAGGAGAAGAAGGAACCGAGAAGAAGGTATCAGCAACAACTGGTTTTATTGCGGGACAGCTCATGATGTTCATATCGATCTATTATGCGCCTCTGCATCTAGCATTGGGTAGACCTCATACAATAACTGTCCTAGCTCTACCGTATCTTTTGTTTCATTTCTTCTGGAACAATCCCAAACACTTTTTTGATTATGGATCTACTACCAGAAATTCAATGCGTAATCTTAGCATTCAATGTGTATTCCTGAATAATCTCATTTTTCAATTATTCAACCATTTCATTTTACCAAGTTCAATGTTAGCCAGATTAGTCAACATTTATATGTTTCGATGCAACAACAAGATGTTATTTGTAACAAGTAGTTTTGTTGGTTGGTTAATTGGTCACATTTTATTCATGAAATGGGTTGGATTGGTATTAGTTTGGATACAGCAAAAGAATTCTATTAGGTCTAATGTACTTATTCGATTTAATAAGTACCTTGTGTCAGAATTGAGAAATTCTATGGCTCGAATCTTTAGTATTCTCTTATTTATTACCTGTATCTACTATTTAGGCAGAATACCCTCACCCATTTTTACTAAGAAACTGAAAGTGAAAGAAACCTCAGAAACGGAAGAAAGAGATGTAGAAATAGAAACAACTTTCGAAAGGAGGGGGACTAAACAGGAACAAGAGGTATCCGCCGAAGAAGATCCTTCTCCTTCCCTTTTTTCGGAAGAAAAGGAGGATCCGGACAAAATCGAGGAAACGGAAGAGATCCGAGTGAATGGAAAGGAAAAAAAAAAAACAAAGCATGAATTCCACTTTCGCTTTAAAGAGACATGCGATAAAAATAGCCCAGTTTATGAAACTTCTTATCTGGATGGGAATCAAGAAAATGCGAAATTCCAAATATTTAAATTATTTAAAGAAAAAGAAGAGAAATATTTATTAAGGTTTGAAAAACCTCTTGTGACTCTTCTTTTCGACTATAAACGATGGACTCGGCCAGTTCGATATAAAAAAAATAATCGATTTGAAAATGCTGTTCGAAATGAAATGTCACACTATTTTTTTTATACATATCGAAGTGATGGAAAAGAAAGGATCTCTTTTACGTATCCAGTAAGTCTGTCAACTTTTTTGGAAATGATAAAAAAAAAAATTTCTTTTTTCACAACAGAAAAACTATCCTCCGATGAATTTTATACACATTGGAATTACACTAATGACCAAAAAAGGAAGAACTTAACCAGGGAGTTTAGAAATAGAATCAAAGTTTTAGATAAAAGATCTCTTATTCTGGATATACTCGAAAAAAGGACTCAATTGTGCAATGATAAGACTAAAAAAAAATACTTACCTAAAATATATGATCCTTTATTACATGGACCTTATCGTGGAAGAATGAAAAATTTATTTTCACCCGCAATCCTAAATAAAACTTATATCAAAAATAAGATAGGAACGCTTTGGATAAATAATATTCACAATATCATTCTTATTAATGATTATGACGAATTTGAACAGACAATAGACCGAGTTAAGCCAAAACCATTTTCAAGAGAAGAAGTAGGGTCTTTATTTACAGAATACGAACGAGAACAAATCGATTCAGAAGAACGAATCCAAAATTTAAAATTTTTATTCGATGCAGTTATAACCGATCCCAATGATCAAAAAATTAGAAAAAAATCGATAAAAGAAATTAGTAAAAGAGTTCCCCGGTGGTCATACAAATTAATCGATAATTTAGACCAAGAGCTGGGAGAATACGACGAAAATGTAAGAGGGGAGCATGCATTTCGTTCACGAAAAGCCAAACGTTTGGTGGTTTCTGTTGATGACCAGACAGACGAGGATGTGACTTTGCCACGTTATTTGGAACAATCAGATTTTCGTCGATATATAATCAAAGGTTCTATGCGCGCACAAAGACGTAAAACCGTTATTTGGAAACCGGTTCAAGCAAATGCCCATTCCCCTCTTTTTTTGGACAGAATAGACAAACCCTTTTATTTGTCTTTTGATATTTCCGGGCTGATCAAAGTAATTTTTAGAAATTGGATGTGGAAAAATAAGGACCAAAAACTTTCTGATTATACAAACGAAAAGACAAGAAAATTGGATAAAAAAACAAAAAAGAAGCCCAAAAAAGAAGGATACACAAGACAAGACAAGGTACGTATAAAACAAGCAGAAGGCTGGGATAAGAGTTTCCGTACTCGAGTACTAAGAAGCTCTATGTTAGTAATCCAATCGATTCTTAGAAAATATATTATATTACCTTCATTGATAATAGTTAAAAACTTGGTTCGCATACTATTCTTCCAAGATCCCGAGTGGTCCGAGGATTTTAAGGATTGGAGTCGGGAAATTTATATTAAATGCACTTATAGTGGTGTTAGTTTATCTGAAACAGAATTTCCGGAAAACTGGTTAACAGAAGGTATTCAGATAAAGATACTATTCCCTTTTCGCCTGAAACCCTGGCACAGATCTAAGATTCAATCCCCTCATAAAGATGCAAAAAGTGAAGCTGATTTTTGTTTTTTAACAGCTTTGGGATTGGAAACTGAAATGCCCTTTGGTTCTCCCCGAAAACGACGTTCGTTTTTTGAACCCATTTTTAAGGAACTCAAAAAAAAAATTCTAAAATTGAAAACTAAGTCTTTTATAGTTTTAGGGGCTTTCAAAGAAGGAAAAATCAAAGAACTTTCAAAAATAAACTTGAGAGAAATCGATGAATCGGGTGAAACTCAAAAAAATTCGATACTCAGTAATCAGAAGATTCACGAATCGTCTATTGAAATTCCATCTATGGATTGGCCAAATTTATCCCGGACCGAAAAAAAAATGAAAGATCTGACTAATAGAACAAGCAGAATACGAAATCAAATATATAAAATTACAAAAGAAAATAAAAAGGGACCGCTAACTCAAGAAACAAATATTAGTTCGAACAAAACATTAGACTCATCAAAAAAGATTTGGCAGATATTCAAAAAAAGAAATACTCGATTAACCCGTAAATCCTATTTTTATCTAAATTTTTTTATTGAAAAGCTTTACATAAATTTTTTTCTATTTACCCTTACTATTCCAAGAATCAATGCAAAACCTTTTAGTGAATCAACAAGAAAAAAAATTAAAATGATTGAGAAAAACATCCACAATAATGAAGAAAATCCGGAAATAATTAATAAAACAAATCAAAATAGAATTCACTTTATTTCGACTGTCAAAAAATCACTTTCGAAGATTAGTAATAAGAATTTAAAGATTTCTTGTAATTTATCGTCTTTTTCACAATCCCAAGCATATGTATTTTACAAATTGTTACAAGCCCCAATTTTTAACTTTTATAATTTAAGACCTGTTCTTCAATATCACGGAACATCTCTATTTCTTAAGAATGAAATAAAGGATTTTTTTGAAAAACACGGAATATTTAATTACCAATTAAGACATAAACCTTTTTGGAATTTTGGAAGGAATCCACGGAAAAACAGGTTAAGCGGTCATTATCAATATGATTTCTCTCGGATTAAATGGGCTAGATTAGTACCACAAGAATGGCGAAATAGGGCCAATCAACACTGTATGGCTCAAAATAAAGATTTAACTAAAAGAGATTCATATGAAAAAAACGGATTAACTCATTGCGAAAAACAACATTTTTTTGAAGCAGACTTATTACGGAATCAAAAATCTAATTTTAAAAAACACTATAGATATGATCTTTTATCATATAAATCGATTAATTATGAAGATAAGAAAGACTCATATATTGATAGATTACTAGGCCAAGTAAATAATAAAGAAGAGTATTATTATAATTACAATATAAAGAAAGCAAAATTATTTGCTATGCTGGGAGGTATCCTTATCAATAATTATCTAGGAGAAGATGATATTATGGATATGGAAAAATTCTTGTATAGAAAATATTTTGATTGGAGAATTCTTAATTTTTGTCTTAGAAATAAGATCAATATTGAAGCCTGGGTTGATATGGATACTGGTATCAGCAGTAATCAAAATACTAAGATTGGGTCCGATAATTATAAAAAAATTGATGCAATTAATAAAAGAGGCCCATTTTATCTTACAATTCATCAAGATGAAGAAATTAACCCATCCAACCAAAAAAGAACACTTTTTGATTGGATGGGAATGAATGAAGAAATACTAAGTTGTCCTATATCAAACCTGGAGCCTTGGTTCTTTCCAGAATTTGTGCTACTTTTTAATGCATATAGAACGAAACCCTGGATCATACCAATTAAATTACTTCTTTTCAATTTTCATGGAAATGGTAAGAAAATTATAACCGGAAAGAACGAAGCGGATCTTTTTATATCATCCACTCAAAAAGAATATCTTGAATTATCGAATCAAAGTAAAGAAGAAAAAGAACTCGCAGACCAAGGAAATGCGGGATCAGATGCCCAAAAGCAAGTAAGTCTTGGATCAGTTCTCTCAAACCAAGAAAAAGATGTTGAAGAAAATTATACGAGATCGGACATGAAAAAACGTATAAAGAAAAAGCAATACAAGAGAGAAACAGAAGCACAGCTTGATTTCTTCCTAAAAAAATATTTGTGTTTGCAGTTAAGATGGAGAGGTACTGTTTCTTTCAAGGAAAAAATACTCAATAATATGGAAGTCTATTGTCACCTGGTTCGACTGATAAATCCTAGCGACGTTACTATAGACTCTATTCAAGGAGGAGAAATTTGTTTGGCTATTTTGATCACTAAGAAGGATTTTGCTCTTAGAGAATTGACGAAAGGGGGAATGCTTATTATCGAACCCCGTCGTTTGTCTGTAAAAAATGATGGCCAATTTTTTATATATCAAACCGTAGGTATTTCATTGGTTCATAAGAATAAGCGAAAAATTACTAAAAGATACCACGAAAAGGGCTATGTTGATAAAAAAATTTTTGATGAATTCATTGCAAAACATCAAAAAATGACTGGAAATAGAAACAAAAATCATTATGATTTGCTTGTTCCTGAAAATATTTTATTCCCTAAACGTCGTAGAGAATTAAGAACTCGAATTTGTTTCAATTCAAAGAATCAAAACGGTATGCAGAGAAATCCAGTATTTTGTACTAACGTAAAAAGCGGGGGTCACCTTTTGGATAAAAACAAAGATCTTCCTAGAGAGAAAAATCAACTAATTAAATTAAAGTTCTTTATTTGGCCCAATTCTCGATTAGAAGATTTAATTTGTATGAATCGCTATTGGTTTAATACCAATAATGGGAGTCGTTTCAGTATGGTAAGGATACATATGTATCCACGATTGAAAATTCGTTAA

>rps7

ATGTCACGTCGAGGTACTACAGAAGAAAAAGCTGCAAAATCCGATCCAATTTATCGTAATCGCTTAGTTAACATGTTGGTTAACCGTATTCTGAAACACGGAAAAAAATCATTGGCTTATCAAATTATCTATCGAGCCTTGAAAAAGATTCAACAAAAGACAGAAAAAAATCCACTATCTGTTTTACGTCAAGCAATACGTGGAGTAACTCCCGATATAGCAGTAAAAGCAAGACGTGTAGGCGGATCGACTCATCAAGTTCCCATTGAAATAGGATCCGCACAAGGAAAAGCACTTGCCGTTCGTTGGTTATTAGGGGCATCCCGAAAACGTCCGGGTCGAAATATGGCTTTCAAATTAAGTTCCGAATTAGTGGATGCTGCCAAAGGGAGTGGCGATGCCATACGCAAAAAGGAAGAGACTCATAGAATGGCAGAGGCAAATAGAGCTTTTGCACATTTTCGTTAA

>ndhB

ATGATCTGGCATGTACAGAATGAAAACTTCATTCTCGATTCTACGAGAATTTTTATGAAAGCCTTTCATTTGCTTCTCTTCGATGGAAGTTTTATTTTCCCAGAATGTATCCTAATTTTTGGCCTAATTCTTCTTCTGATGATCGATTCAACCTCTGATCAAAAAGATATACCTTGGTTATATTTCATCTCTTCAACAAGTTTAGTAATGAGCATAGCGGCCCTATTGTTCCGATGGAGAGAAGAACCTATGATTAGCTTTTCGGGAAATTTCCAAACGAACAATTTCAACGAAATCTTTCAATTTCTTATTTTACTATGTTCAACTCTATGTATTCCTCTATCCGTGGAGTACATTGAATGTACAGAAATGGCTATAACAGAGTTTCTGTTATTCGTATTAACAGCTACTCTAGGAGGAATGTTTTTATGCGGTGCTAACGATTTAATAACTATCTTTGTAGCTCCAGAATGTTTCAGTTTATGCTCCTACCTATTATCTGGATATACCAAGAAAGACGTACGGTCTAATGAGGCTACTATGAAATATTTACTCATGGGTGGGGCAAGCTCTTCTATTCTGGTTCATGGTTTCTCTTGGCTATATGGTTCATCCGGGGGCGAGATCGAGCTTCAAGAAATAGTGAATGGTCTTATCAATACACAAATGTATAACTCCCCAGGAATTTCAATTGCGCTTATATTCATCACTGTAGGAATTGGGTTCAAGCTTTCCCTAGCCCCTTCTCATCAATGGACTCCTGACGTATACGAAGGATCTCCCACTCCAGTCGTTGCTTTTCTTTCTGTTACTTCGAAAGTAGCTGCTTCAGCTTCAGCCACTCGAATTTTCGATATTCCTTTTTATTTCTCATCAAACGAATGGCATCTTCTTCTGGAAATCCTAGCTATTCTTAGCATGATATTGGGGAATCTCATTGCTATTACTCAAACAAGCATGAAACGTATGCTTGCATATTCGTCCATAGGTCAAATCGGATATGTAATTATTGGAATAATTGTTGGAGACTCAAATGGTGGATATGCGAGCATGATAACTTATATGCTGTTCTATATCTCCATGAATCTAGGAACTTTTGCTTGCATTGTATTATTTGGTCTACGTACCGGAACTGATAACATTCGAGATTATGCAGGATTATACACAAAAGATCCTTTTTTGGCTCTCTCTTTAGCTCTATGTCTCTTATCCCTAGGAGGTCTTCCTCCACTAGCAGGTTTTTTCGGAAAACTCCATTTATTCTGGTGTGGATGGCAGGCAGGCCTATATTTCTTGGTTTCAATAGGACTCCTTACGAGCGTTGTTTCTATCTACTATTATCTAAAAATAATCAAGTTATTAATGACTGGACGAAAGCAAGAAATAACCCCTCACGTGCGAAATTATAGAGGATCCCCTTTAAGATCAAACAATTCCATCGAATTGAGTATGATTGTATGTGTGATAGCATCTACTATACCAGGAATATCAATGAACCCGATTATTGCAATTGCTCAGGATACCCTTTTTTAG

>ycf15

ATGCTACTGCTGAAACATAGAAGAATTGAAATCTTAGATCAAAACACTATGTATGGATGGTATGAACTGCTTAAACAAGAATTCTTGAACAGCGAACCACCAGAGCTATTACTAACTACATCAAAAAATTTCCATTAA

>ycf2

ATGAAAGGACATCAATTCAAATCCTGGATTTTCGAATTGAGAGAGATATTGAGAGAGATCAAGAATTCTCACTATTTCTTAGATTCATGGACCCAATTCAATTCAGTGGGATCTTTCATTCACATTTTTTTCCACCAAGAACGTTTTATAAAACTCTTGGACCCACGAATTTGGAGTATCCTACTTTCACGCAATTCACAGGGTTCAACAAGCAATCGATATTTCACGATCAAGGGTGTAGTACTATTTGTAGTAGCGATCCTTATATATCGTATTAACAATCGAAATATGGTCGAAAGAAAAAATCTCTATTTGACAGGGCTTCTTCCTATACCTATGAATTCCATTGGACCCAGAACTGATACATTGGAAGAATCTTTTGGGTCTTCCAATATCAATAGGTTGATTGTTTCGCTCCTCTATCTTCCAAAAGGAAAAAAGATCTCTGAGAGCTTTTTCCTGGATCCGAAAGAGAGTACTTGGGTTCTCCCAATAACTAAAAAGTGTATCATGCCTGAATCTAACTGGGGTTCGCGGTGGTGGAGGAACTGGCTCGGAAAAAAGAGGGATTCTAGTTGTAAGATATCTAATGAAACCGTCGCTGGAATTGAGATCTCATTCAAAGAGAAAGATATCAAATATCTGGAGTTTCTTTTTGTATATTATATGGATGATCCGATCCGCAAGGACCATGATTGGGAATTGTTTGATCGTCTTTCTCCGAGTAAGAGGCGAAACATAATCAACTTGAATTCGGGACATCTATTCGAAATCTTAGTGAAAGACTGGATTTGTTATCTCATGTTTGCTTTTCGTGAAAAAATACCAATTGAAGTGGAGGGTTTCTTCAAACAACAAGGAGCTGGGTCAACTATTCAATCAAATGATATTGAGCGTTTTTCCCATCTCTTCTTGAGAAACAAGTGGGCTATTTCTTTGCAAAATTGTGCTCAATTTCATATGTGGCAATTCCGCCAAGATCTCTTCGTTAGTTGGGGGAAGAATCCGCACGAATCGGATTTTGTGAGGAACATATCGAGAGAGAATTGGATTTGGTTAGACAATGTGTGGTTGGTAAACAAGGATCGATTTTTTAGCAAGGTACGGAATGTATCGTCAAATATTCAATATGATTCCACAAGATCTAGTTTCGTTCAAGTAACGGATTCTAGCCAATTGAAAGGATCTTCTGATCAATCCAGAGATCTTTTCGATTCCATTAGTAATGAGGATTCGGAATATCACACATTGATCAATCAAAGAGAGATTCAACAACTAAAAGAAAGATCGATTCTTTGGGATCCTTCCTTTCTTCAAACGGAACGAAGAGAGATAGAATCAGACCGATTCCCTAAATGTCTTTCTGGATATTCCTCAATGTCCCGGCTATTCACGGAACGTGAAAGGCAGATGAATAAGCATCTGCTTCCGGAAGAAATCGAAGAATTTCTTGGGAATCCTGCAAGATCCATTCGTTCTTTTTTCTCTGACAGATGGTCAGAACTTCATCTGGGTTCGAATCCTACTGAGAGGTCCACTAGAGATCAGAAATTGTTGAAGAAAGAAGAGGATGTTTCTTTTGTCCCTTCCAGGCGATCGGAAAATAAAGAAATAGTTAATATATTCAAGATAATTACGTATTTACAAAATACCGTCTCAATTCATCCTATTTCATCAGATCCGGGATGTGATATGGTTCCGAAGGATGAACTGGATATGGACAGTTCCAATAAGATTTCATTCTTAAACAAAAATCCATTTTTTGATTTATTTCATCTATTCCATGACCGGAACAGGGGGGGATACACGTTACACCATGATTTTGAATCAGAAGAGAGATTTCAAGAAATGGCAGATCTATTCACTCTATCAATAACCGAGCCGGATCTGGTGTATCATAAGGGATTTGCCTTTTCTATTTTTTCCTACGGATTGGATCAAAAACAATTCTTGAATGAGGTATTCAACTCCAGGAATGAATCGAAAAAGAAATCTTTATTGGTTCTACCTCCTATTTTTTATGAAGAGAATGAATCTTTTTATCGAAGGATCAGAAAAAAATGGGTCCGGATCTCCTGCGGGAATGATTTGGAAGATCCAAAACCAAAAAGAGTGGTATTTGCTAGCAACAACATAATGGAGGCAGTCAATCAATCTAGATTGATCCGAAATCGGATTCAAATCCAATATAGCACCTATGGGTACATAAGAAATGTATTGAATCGATTCTTTTTAATGAATAGATCCGATCGCAACTTCGAATATGGAATTCACAGGGATCAAATAGGAAATGATACTCTGAATCATAGAACTATAATGAAATATACGATCAACCAAGATTTATCGAATTTGAAAAAGAGTCAGAAGAAATGGTTCGCTCCTCTTATTTTTATTTCTCGAACCGAGAGATTCATGAATCGGGATCCTAATGCATATAGATACAAATGGTCCAATGGGAGCAAGAATTTCCAGGAGCATTTGAAACATTTCGTTTCTGAGCAGAAGAGCCGTTTTCAAGTAGTGTTCGATCGATTACGTATTAATCAATATTCGATTGATTGGTCTGAAGTTATCGACAAAAAAGATTTGTCCAAGTTGCTTTTCTTTTTGTCTAACTCACTTCCTTTTTTCTTTGTGAGTTTCGGGAATATCCCCATTCATAGGTCTGAGATCCACATCTATGAATTGAAAGGTCCGAATGATCAACTCTGCAATCAGTTGTTAGAATCAATAGGTCTTCAAATCGTTCATTTGAAAAAATTGAAACCCTTCTTATTGGATGATCATAATACTTCCCAAAAATCGAAATTCTTGATCAATGGAGGAAGAATATCACCATTTTTGTTCAATAAGATACCAAAGTGGATGATTGACTCATTCCATACTAGAAATAATCGCAGGAAATCTTTTGATAACACGGATTCCTATTTCTCAACGATATCCCACGATCAAGACAATTGGCTGAATCCTGTGAAACCATTTCATAGAAGTTCATTGATATCTTCTTTTTATAAAGCAAATCGACTTCGATTCTTGAATAATCCACATCGCTTCCGCTTCTATTGTAACAAAAGATTCACTTTTTATGTGGAAAAGGTCCGTATCAATAATTATGATTTTACGTATGGACAATTCCTCAATATCTTGTTCATTCGCAACAAAATATTTTCTTTGTGCGGCGGTAAAAAAAAACATGCTTTTTTGGAGAGAGATACTATTTCACCAATCGAGTCACAGGTATCTAACATATTCATACCTAACGATTTTCCACAAAGCGGTGACGAAAGGTATAACTTGTACAAATTTTTCCCTTTTCCAATTCGATCCGATCTATTAGTTCGTAGAGCTATTTACTCGATCGCAGCCATTTCTGGAACACCTCTAACAGAGGGACAAATAGTCAATTTTGAAAGAACTTATTGTCAACCTCTTTCAGATATGAATCTATCTGATTCAGACGAGAAGAACTTGCATCAGTATCTCAATTTCAATTCAAACATGGGTTTGATTCACACTCCATGTTCTGAGAAATATTTACCATCCGAAAAGAGGAAAAAACGGAGTCTTTGTCTAAAGAAATGCGTTGAGAAAGGGCAGATGTCTAGAACCTTTCAACGAGACAGTGCTTTTTCAACTCTCTCAAAATGGAATCGATTCCAAACATATATGCCATGGTTCCTTACTTCGACAGGGTACAAATATCTAAATTTTCTATTTTTAGATACTTTTTCAGACCTATTGCCGGTACTAAGTAGCAGTCAAAAATTTGTATCCATTTTTCATGATATTATGCATGGATCAGATATATCATGGCGAATTCTTCAGAAAAAATGGCGTCTTCCACAATGGAATCTGATAAGTGAGATTTCGAGTAAGTGTTTCCATAATCTTCTTCTGTCCGAAGAAATGATTCATCGAAATAATGAGTCACCATTGATATCGACACATCTGAGATCGCCAAATGCTCGGGAGTTCCTCTATTCAATCCTTTTCCTTCTTCTTGTTGCTGGATATCTCGTTCATACACATCTTATCTTTGTTTCCCGAGCCTATAGTGAGTTAGAGACAGAGTTCGAAAGGGTCAAATCTTTGATGATTCCATCATACATGATTGAGTTGCGAAAACTTCTGGATAGGTATCCCACATCTGAACTGAATTCTTTCTGGTTAAAGAATCTCCTTCTAGTTGCTCTGGAACAATTAGGAGATTCTCTAGAAGAAATACGGGGTTCTGCTTTTGGCGGCAACATGCTATGGGGTGGTGGTCCCGCGGATGGGGTTAAATCAATACGTTCTAAGACGAAAGATTTGAATATCAATCTCATCGATATCATCGATCTCATAAGTATCATACCAAATCCCATCAATCGAATCACTTTTTCGAGAAATACGAAACATCTAAGTCATACAAGTAAAGAGATCTATTCATTGATAAGAAAAAGAAAAAAGGGGAACGGTGATTGGATTGATGATAAAATAGAATCCTGGGTCGCGAACAGTGATTCGATTGATGATAAAGAAAGAGAATTCTTGGTTCAGTTCTCCACCTTAACGACAGAAAAAAGGATTGATCAAATTCTATTGAGTCTGACTCATAGTGATCATTTATCAAAGAATGACTCTGGTTATCAAATGATTGAACAACCGGGAACAATTTACTTACGATACTTAGTTGACATTCATAAAAAGCATTTCATGAATTATGAGTTCAATACATACTGTTTAGCAGAAAGACGGATATTCCTTGCTCATTATCAGACAATCACTTATTCACAAACTTCGTGTGGGGCTAATAGTTTTCATTTCCCATCTCATGGAAAACCCTTTTCGCTCCGCCTAGCCCTATCCCCCTCCAGGGGTATTTTAGTGATAGGTTCTATAGGAACCGGACGCTCCTATTTGGTCAAATACCTAGCGACAAACTCCTATGTTCCTTTCATTACAGTATTTCTGAACAAGTTCCTGGATAACAAACCTAAAGGTTTTCTTATTGATGATATCGATGATGATAGTGACGATATTGATGCTAGTGACGATATTGATGCTAGTGACGATATCGATCGTGACCTTGATACGGAGCTGGAGCTTCTAACTATGATGAATGCGCTAACTATCGATATGATGTCGGAAATAGGCCTATTTTATATCACCCTTCAATTCGAATTAGCAAAAGCAATGTCTCCTTGCATAATATGGATTCCAAACATTCATGATCTGGATGTGACTGAGTCGAATTACTTATCCCTCGGTCTATTAGTGAACTATCTCTCCAGGGATTGTGAAAGATGTTCCACTAGAAATATTCTTGTTATTGCTTCGACTCATATTCCCCAAAAAGTGGATCCCGCTCTAATAGCCCCGAATAAATTAAATACATGCATTAAGATACGAAGGCTTCTTATTCCACAACAACGAAAGCACTTTTTCACTCTTTCATATACTAGGGGATTTCACTTGGAAAAGAAAATGTTCCATACTAATGGATTCGGGTCCATAACCATGGGTTCCAATGCACGAGATCTTGTAGCACTTACCAATGAGGCCCTCTTGATTAGTATTACACAGAAGAAATCAATTATAGACACTAATACAATTAGATCTGCTCTTCATAGACAAACTTGGGATTTGCGATCCCAGGTAAGATCGGTTCAAGATCATGGGATCCTTTTCTATCAGATAGGAAGGGCTGTTGCACAAAATGTACTTCTAAGTAATTGCCCCATAGATCCTATATCTATCTATATGAAGAAGAAATCATGTAACGAAGGGGATTCTTATTTGTACAAATGGTACTTCGAACTTGGAACGAGCATGAAGAAATTAACGATACTTCTTTATCTTTTGAGTTGTTCTGCCGGATCGGTCGCTCAAGACCTTTGGTCTCTACCCGGACCCGATGAAAAAAACGGGATCACTTCTTCTGGACTCGTTGAGAATGATTCTGATCTAGTCCATGGCCTATTAGAAGTAGAAGGCGCTCTGGTGGGATCCTCGCGGACAGAAAAAGATTGCAGTCGGTTTGATAATGATCGAGTGACATTGCTTCTTCGGCCCGAACCAAGGAATCCCTTAGATATGATGCAAAAAGGATCTTGTTCTATCGTTAATCAGAGATTTCTCTATGAAAAATACGAATCGGAGTTTGAAGAAGGGGAAGGAGAAGGAGTCCTCGACCCGCAACAGATAGAGGAGGATTTATTCAATCACATAGTCTGGGCTCCTAGAATATGGCGCCCCTGGGGCTTTCTATTTGATTGTATCGAAAGGCCCAATGAATTGGGATTTCCCTATTGGGCCGGGTCATTTCGGGGCAAGCGGATCATTTATGATGAAAAGGATGAGCTTCAAGAGAATGATTCGGAGTTCTTGCAGAGTAGAACCATGCAGTACCAGACACGAGATAGATCTTCCAACGAACAAGGCTTTTTTCGAATAAGCCAATTTATTTGGGAACCTGCGGATCCACTCTTTTTCCTATTCAAAGATCAGCCCCTTGTCTCTGTGTTTTCACACCGAGAATTCTTTGCAGATGAAGAGATGTCAAAGGGGCTTCTTACTTCCCAACCAGATCCTCCTACATCTATATATAAACGCTGGTTTATCAAGAATACGCAAGAAAAGCACTTCGAATTGTTGATTCATCGCCAGAGATGGCTTAGAACCAATAGTTCATTATCTAATGGATTTTTCCGTTCTAATACTCCATCCGAGAGTTATCAGTATTTATCAAATCTCTTCCTATCTAACGGAACGCTATTGGATCAAATGACAAAGACATTGTTGAGAAAAAGATGGCTTTTTCCGGATGAAATGAAAATTGGATTCATGTAA

>rpl23

ATGGATGGAATCAAATATGCAGTATTTACAGACAAAAGTATTCGGTTATTGGGGAAAAATCAATATACTTCTAATGTCGAATCAGGATCAACTAGGACAGAAATAAAGCATTGGGTCGAACTCTTCTTTGGTGTCAAGGTAATAGCTATGAATAGTCATCAACTCCCCCGAAAGGGTAGAAGAATGGGACCTATTATGGCACATACAATGCATTACAGACGTATGATCATTACGCTTCAACCGGGTTATTCTATTCCACCTCTTAGAAAGAAAAGAACTTAA

>rpl2

ATGGCGATACATTTATACAAAACTTCTACCCCGAGCACACGCAATGGAGCCGTAGACAGTCAAGTGAAATCCAATCCACGAAATAATTTGATCTATGGACAGCATCGTTGTGGTAAAGGTCGTAATGCCAGAGGAATCATTACCGCAGGGCATAGAGGGGGAGGTCATAAGCGTCTATACCGTAAAATCGATTTTCGACGGAATGCAAAAGACATATATGGTAGAATCGTAACCATAGAATACGACCCTAATCGAAATGCATACATTTGTCTCATACACTATGGGGATGGTGAGAAGAGATATATTTTACATCCCAGAGGGGCTATAATTGGAGATACCATTGTTTCTGGTACAGAAGTTCCTATAAAAATGGGAAATGCCCTACCTTTGACCGATATGCCCTTAGGCACGGCCATACATAACATAGAAATCACACTTGGAAAGGGTGGACAATTAGCTAGAGCAGCGGGTGCTGTAGCGAAACTGATTGCAAAAGAGGGGAAATCGGCCACATTAAAATTACCTTCTGGGGAGGTCCGTTTGATATCCAAAAACTGCTCAGCAACAGTCGGACAAGTGGGGAATGTTGGGGTGAACCAGAAAAGTTTGGGTAGAGCCGGATCTAAATGTTGGCTAGGTAAGCGTCCTGTAGTAAGAGGAGTAGTTATGAACCCTGTAGACCACCCCCATGGGGGTGGTGAAGGAAGGGCTCCAATTGGTAGAAAAAGACCCGCAACCCCTTGGGGTTATCCTGCACTTGGAAGAAGAAGTAGAAAAAGGAATAAATATAGTGATAATTTGATTCTTCGCCGCCGTACTAAATAG

***R. graveolens***

>psbA

ATGACTGCAATTTTAGAGAGACGCGAAAGTGACCGCCTGTGGGGTCGTTTCTGTAACTGGATAACCAGCACCGAAAACCGCCTTTACATTGGATGGTTTGGTGTTTTGATGATCCCTACTTTATTGACCGCAACTTCTGTATTTATTATCGCCTTCATTGCTGCTCCTCCAGTAGATATTGATGGTATTCGTGAACCTGTTTCTGGATCTCTACTTTACGGAAACAATATTATTTCTGGTGCGATTATTCCTACTTCTGCAGCTATAGGTTTACACTTTTACCCGATATGGGAAGCGGCATCCGTTGATGAATGGTTATACAATGGCGGTCCTTATGAGCTAATTGTTCTACACTTCTTGCTTGGTGTAGCTTGTTACATGGGGCGTGAGTGGGAACTTAGTTTCCGTCTGGGTATGCGCCCGTGGATTGCTGTTGCATATTCAGCTCCTGTTGCAGCAGCGACTGCTGTTTTCTTGATCTACCCAATCGGTCAAGGAAGTTTTTCTGATGGTATGCCTCTAGGAATCTCTGGTACTTTCAACTTCATGATTGTATTCCAGGCTGAGCACAACATCCTTATGCACCCATTCCACATGTTAGGCGTAGCTGGTGTATTCGGCGGCTCCCTATTCAGTGCTATGCATGGTTCCTTGGTAACCTCGAGTTTGATCAGGGAAACCACAGAAAATGAATCTGCTAATGAAGGTTACAGATTCGGTCAAGAGGAAGAAACCTATAATATCGTAGCTGCTCACGGTTATTTTGGCCGATTGATCTTCCAATATGCTAGTTTCAATAATTCTCGTTCTTTACATTTCTTCCTAGCTGCTTGGCCTGTAGTAGGTATCTGGTTCACTGCTTTAGGTATTAGCACTATGGCTTTCAACCTAAATGGTTTCAATTTCAACCAATCTGTAGTTGACAGTCAAGGTCGTGTAATTAATACCTGGGCTGATATTATTAACCGTGCTAACCTTGGTATGGAAGTTATGCATGAACGTAATGCTCACAACTTCCCTCTAGACCTAGCTGCTATTGAAGCTCCATCTACAAATGGATAA

>matK

ATGGAGGAATTTCAAGTATATTTCGAACTAGATAGATCGCAACAACACAATTTCCTATACCCACTTCTTTTTCGGGAGTATATTTATGCGCTTGCTCATGATCATGGTTTAAATAGCTCGATGATGTCATTGGAAAGTGGGGTTTATGACAATAAATCTAGTTCACTAAGTGTCAAACGTTTAATTACTCGAATGTATCAGCGGATTCAGTTGCGTATTGCTCCTAATGATTCTAACCAAAGTCCCATTTTTGGGCACAACAATCTGTTGTATTCTCAAATTCTATCAGAAGGATTTGCTGTCGTGGTGGAAATTCCATTTTCCCTACGTTTGGTAGCTTTTTTAGAAGGGAAAGAAATTGACAAATTTCATAATTTTCAATCAATTCATTCAATATTTCCTTTTTTCGAGGACAAATTGTTACATTTAAATTTTGTGTTAGATGTACGAATACCCCACCCCATTTGTCCCGAAATTTTGGTTCAAATACTTCGCTACTGGGTAAAGGATGCCTCTTCTTTACATTTATTACGGTTCGTTCTTCACGAGTATTTTAATTCGAACAGCCTTATTATTCCAAAAAACTTGAGTTCTGTTTTTTTAAAAAGTAATCCAAGATTGTTATTGTTTCTATATAATTCTCATGTATATGAATATGAATCTATCCTCTTTTTTCTCTGTAACCAATCGTCTCATTTACGATCAACATCCTCTCGAGCCCTCGTTGAACGAATGTATTTCTATAGAAAAGTCGAACATCTTTTCGAAGTCTTTGCTAAAGCTTTTCAGGACATCTTAGGTTTGTTCAAGGATCCTTGCATGCATTATGTTAGATATCAAGGAAAATCCATTCTGGCATCAAAAGATAGGCCTCTTCTGATGACTAAATGGCAATATTACCTTTTCAGTTTATGGCAATGGCATTTTCACATATCATCTCAATCTGGAAAGGTTCATCTAAAGCACTTAGACAAGTACTCTATCAACTTTCTGGGCTATCTTTCCAGTGTGCAACTCAATTCTTTGGTGGTACGGAGTCGAATGCTAGAAAATTCTTTTCTAATAGGTAATTCTCTGAAGAAGGTCGATACGATGGTTCCAATTATTCATCTGATTGGAGCATTGACTAAGGCGCGGTTTTGTAATGCATTAGGGCATCCCATCAGTAAGTCGTCCTGGGCCGATTTCTCTGATTCTCATCTTATCGACCGGTTTGTGCGTATATGCAGAAATCTTTCTCATTATCATAGCGGATCCTCAAAAAACAAAAGTTTGTATCGAATAAAATATATACTTCGCCTTTCCTGTGTTAAAAGTTTGGTTCGTAAACATAAAAGTACTGTACGCGCTTTTTTAAAAAGATCAGGTTCGGGATTTTTGGAAGAATTCCTTATGGAGGAAGAACACGTTCTTTCTTTAATCTTTCCAAGGGCTTCGTCTACTTCGCGGGGGTTCTATAGAGGGCAGATTTGGTATTTGGATATTTTTTTTATCAACGATCTGGTTAATTATGACTGA

>rps16

ATGGTAAAACTTCGTTTGAAACGATGTGGTAGAAAGCAACGTGCCGTTTATCGAATCGTTGCAATTGATGGTCGATCCCGAAGAGAAGGAAGAGATCTTCGGAAAGTGGGTTTTTATGATCCGATAAATAATCAAACCCAGTTAAATGTTCCTGCTATTCTCTATTTTCTTGACAAGGGCGCCCAGCCTACAGGAACCGTTTATGATATTTCAAAGAAAGCGGGGGTTTTTACAGAATTGATTCTTAATCAAACGAAATTCGATTAA

>psbK

ATGCTTAATATCTTTAGTTTAATGTATATCTGTATTAATTTTGCCCTTTATTCGAGTAGTTTTTTATTCGCCAAATTGCCTGAGGCATACGCTTTTTTGAATCCAATTGTAGATGTTATGCCAGTAATACCTCTTCTATTTTTGCTCTTAGCCTTTGTTTGGCAAGCTGCTGTAAGTTTTCGATGA

>psbI

ATGCTTACTCTCAAACTCTTTGTTTACACTGTAGTGATATTCTTTGTTTCTCTCTTCATCTTTGGATTCCTGTCTAATGATCCAGGGCGTAATCCCGGACGTGAAGAATAA

>atpA

ATGGCAACAATTAAAGCCGACGAAATTAGTAATATTATCCGGGAACGTATTGAGCAATATAATAGAGAAGTAAAGATTGTAAATATTGGCACCGTACTTCAAGTAGGCGACGGCATTGCCCGTATTTATGGTCTTGATGAAGTAATGGCAGGTGAATTGGTAGAGTTTGAAGAGGGTACAATAGGCATTGCTCTGAATTTGGAATCAAATAATGTTGGTGTTGTTTTAATGGGTGACGGTTTAATGATCCAAGAGGGAAGTTCTGTAAAAGCAACCGGAAAAATTGCTCAGATACCAGTAAGCGAGGCTTATTTAGGTCGTGTTATAAACGCCCTGGCTAAACCTATTGATGGTCGAGGTGAAATTTCAGCTTCGGAATCGCGATTAATTGAATCTCCTGCTCCGGGTATTATTTCAAGACGTTCTGTCTATGAGCCTCTGCAAACAGGCCTTATTGCTATTGATTCGATGATTCCTATCGGACGCGGGCAGCGAGAATTAATTATTGGGGACAGACAGACTGGTAAAACAGCAGTAGCCACAGATACGATTCTCAACCAGCAAGGGCAAAATGTAATATGCGTTTATGTAGCTATTGGTCAAAAAGCATCTTCGGTGGCTCAGGTAGTGAATACTTTCCAGGAGAGGGGGGCAATGGAATACACCATTGTGGTAGCCGAAACGGCGGATTCCCCCGCTACGTTACAATACCTCGCTCCTTATACGGGCGCAGCTCTAGCTGAATATTTTATGTACCGTGAACGACACACTTTAATCATTTATGATGATCCATCCAAACAAGCACAGGCTTATCGACAAATGTCTCTTCTATTACGAAGACCCCCCGGTCGTGAAGCTTACCCCGGAGATGTTTTTTATTTACATTCACGACTTTTGGAAAGAGCCGCGAAATTAGGTTCGCAATTAGGCGAAGGAAGTATGACCGCTTTACCAATAGTTGAGACCCAGTCAGGAGATGTTTCGGCTTATATTCCTACTAATGTAATTTCTATTACAGATGGTCAAATATTTTTATCCGCGGATCTATTCAATGCAGGAATCAGACCCGCAATTAATGTGGGTATTTCGGTCTCGAGAGTCGGATCCGCAGCTCAGATTAAAGCTATGAAACAAGTAGCCGGAAAATTAAAATTGGAATTGGCCCAATTCGCAGAATTAGAAGCCTTTGCACAATTTGCCTCTGATCTCGATAAAGCTACTCAGAACCAATTGGCAAGGGGCCAACGCTTACGTGAGTTGCTCAAACAATCCCAATCAGCACCTCTCACGGTCGAAGAACAGATAATGACTATTTATACAGGAACTAATGGTTATCTTGATTCATTAGAAATTGGCCAAGTAAGAAAATTTCTCGTTGAGTTACGTACTTACTTAAAAACGAATAAACCCCAGTTCCAAGAAATAATATCTTCTACCAAGGTATTTACCGAGGAAGCCGAAGCCCTTTTGAAAGAAGCTATTCAGGAACAAAAGGAACGCTTTCTACTTCAGGAACAATTATAA

>atpF

ATGAAAAATGTAACCGATTCTTTCGTTTCTTTGGTTCACTGGCCATTCGCCGGGAGTTTCGGGCTTAATACCGATATTTTAGCAACAAATCCAATAAATCTAAGTGTAGTGCTTGGTGTATTGATCTTTTTTGGAAAGGGAGTGTTAAGTGATTTATTAGATAATCGAAAAATGAGGATCTTAAATAGTATTCGAAATTCAGAAGAACTGCAGCGAGGAGCCATTGAACGGCTGGAAAAAGCCCGGGACCGGTTACGGAAAATCAAAATAGAAGCCGATCAGTTTCGGGTGAATGGATACTCTGAGATAGAACGAGAAAAATTGAATTTGAAAAATTCAACTTATAAGACCTTGGATCAATTCGAAAATTACAAAAATGAAACCATTCATTTTGAACAACAAAGAGCAATTAATCAAGTTCGACAACGGGTTTTCCAACAAGCTTTACAAGGAGCGCTCGGAACTCTGAATAGTTGTTTGAATAAGGAGTTACATTTACGTACCATTAGTGACAATATTGGCATGTTTGGGGCGATGAAAGAAATAACTGATTAG

>atpH

ATGAATCCACTGATTTCTGCCGCTTCCGTTATTGCTGCCGGGTTGGCTGTTGGGCTTGCTTCTATTGGACCTGGAGTTGGTCAAGGTACTGCTGCGGGTCAAGCAGTAGAAGGGATCGCGAGACAACCCGAGGCGGAGGGAAAAATACGAGGTACTTTATTGCTTAGTCTGGCTTTTATGGAAGCTTTAACAATTTATGGACTGGTTGTAGCATTAGCACTTTTATTTGCTAATCCTTTTGTTTAA

>atpI

ATGAATGTTCTATCATGTTCCATCAACACACTAAAGGGGTTATACGATATATCCGGTGTGGAAGTAGGCCAACATTTCTATTGGCAAATAGGTGGGTTCCAAGTCCATGCCCAAGTACTTATTACTTCTTGGGTTGTAATTGCGATCTTATTAGGATCAGCCTTTATAGCCGTTCGGAATCCACAAACCGTTCCGACTGCCAGTCAAAATTTCTTCGAATATGTCCTTGAATTCATTCGAGACGTGAGCAAAACTCAGATTGGAGAAGAATATGGACCATGGGTTCCCTTTATTGGAACTCTGTTTCTTTTTATTTTTGTTTCGAATTGGTCAGGCGCTCTTTTGCCTTGGAAAATCATCGAGTTACCCCATGGGGAGTTAGCCGCACCCACGAATGATATAAATACTACCGTTGCTTTAGCTTTGCTCACGTCAGTAGCCTACTTCTATGCGGGTCTTTCCAAAAAGGGATTAGGTTATTTCCGTAAATACATTCAACCGACTCCAATTCTTTTACCAATTAACATTTTAGAAGATTTCACAAAACCCTTATCGCTTAGTTTTCGGCTTTTCGGGAATATATTAGCCGATGAATTAGTAGTTGTTGTTCTTGTTTCTTTAGTACCTTTAGTGGTTCCTATCCCTGTCATGTTCCTTGGATTATTTACAAGCGGTATTCAAGCTCTTATTTTTGCAACTTTAGCTGCGGCTTATATAGGCGAATCTATGGAGGGACATCATTGA

>rps2

ATGGCAAGAAGATATTGGAACATCCAGTTGGAAGAGATGATGGAAGCAGGAATTCATTTTGGTCATGGTACTCGGAAATGGAATCCTAGAATGGCGCCTTATATATCTGCAAAACATAAGGGTATTCATATTCTAAATCTGACTCGAACTGCTCGTTTTTTATCAGAAGCTTGTGATTTAGTTTTTGATGCAGCAAGTAGAGGAAAACAATTCTTAATTGTTGGTACGAAAAATAAAGCAGCTGATTCAGTCGCACGAGCTGCAATAAGGGCTCGGTGTCATTATGTTAATAAAAAATGGCTCGGCGGTATGTTAACGAATTGGTTCACTACAGAAACGAGACTCCACAAGTTCAGGGATTTGCGAACGGAACACAAAAGGGGGGGACTCGATAGTCTTCCCAAAAGGGATGCCGCTATTTTGAAGAGACAATTATCGCGCCTGCAAACGTATCTGGGCGGGATTAAATATATGACGAGGGTACCCGATATTGTAATCATCGTTGATCAGCAAGAAGAATATACGGCTCTTCGAGAATGTATCACTTTGGGAATTCCAACAATTTGTTTAATCGATACAAATTGTGATCCCGATCTCGCAGATATTTCGATTCCAGCAAACGATGACGCTATAGCCTCAATCCGATTAATTCTTAACAAATTAGTAGTCGCAATTTGTGAGGGTCGCTCTAGCTATATACGAAATCCTTGA

>rpoC2

ATGGCAGAACGGGTGAGTCTGGTCTTTCACAATAAAATGATAGATGGAACTGCCATTAAACGACTTATTAGCAGGTTAATAGATCACTTCGGAATGGCATATACATCACACATACTCGATCACGTAAAGACCCTGGGTTTCCAGCAAGCCACCGCTACATCTATTTCATTAGGCATTGATGATCTTTTAACGATACCTTCTAAGCGATGGCTAGTCCAAGATGCTGAACAACAAAGTTTTATTTTGGAAAAACACCATCATTATGGGAATGTACACGCAATAGAAAAACTACGTCAATCCATTGAAATATGGTATGCTACAAGTGAATATTTGCGACAAGAAATGAATCCGAATTTTAGGATGACTGATCCCTTTAATCCAGTCCATATAATGTCCTTTTCGGGAGCTAGAGGAAATGCATCTCAAGTACACCAATTGGTGGGTATGAGAGGATTAATGTCTGATCCCCAAGGGCAAATGATTGATTTACCCATTCAAAGCAATTTACGCGAAGGACTCTCTTTAACCGAATATATAATTTCTTGCTATGGAGCCCGCAAAGGAGTTGTGGATACCGCTGTCCGAACCTCCGATGCAGGATATCTTACGCGCAGACTTGTTGAAGTAGTTCAACACGTTATTGTACGTAGAACAGATTGCGGCACCATCCAAGGAATTTCTGTGAGCCCTCAAAATCAAAATAAGATGATGTCGGAAAGGGTTTTTAGCCAAACATTAATTGGGCGTGTATTAGCGGACGATATATATATGGGCCCGCGATGCATCGCCATTAGAAATCAAGACATTGGGATTGGACTTGTCAAGCGACTCATAACCTTTCGAACACAAGCAATATCTATTCGAACCCCTTTTACTTGTAGGAGTACATCGTGGATCTGTCGATTATGCTATGGTCGGAGTCCGACGCATGGTGACCTGGTTGAATTGGGGGAAGCCGTCGGTATTATTTCGGGTCAATCTATTGGGGAGCCGGGAACTCAACTAACATTAAGAACTTTTCATACCGGCGGTGTATTTACAGGGGGTACTGCAGAACATGTACGAGCCCCTTCAAATGGTCAAATCAAATTCAATGAGGATTTTGTTCATCCCACGCGCACACGCCATGGGCATCCCGCTTTTCTATGCTCTATAGATTTGGATGTAATTATTGAGAGTGAAGATATTATCCATAATGTGACTATTCCACCAAAAAGTTTTCTTTTAGTTCAAAACGATCAATATGTCGAATCCGAACAAGTGATTGCTGAGATTCAGGCGGGAGCATACACTTCGAATTTTAAAGAGAGGGTTCGAAAACATATTTATTCTGATTCTGAGGGAGAAATGCACTGGAGTACTGATGTGTACCATGCACCCGAATTTACATATAGTAATGTCCACCTCTTGCCAAAAACAAGTCATTTATGGATATTATCGGGGGGTTCGCGCAGATCTAATGTAGTTTCCTTTTCACTCCACAAGGATCAAGATCAAATGAATATTCATTCGCTTTCTGTCGAACGGCGAGAGATTTCTGGCCTCGCGCTCTCAGGGAATAATGATCAAGGGAGGCATAAATTTTTGAGTTCTGATTTTTCGGGATTTAATAGAATCATAGGTACTGGTCATTGTAATCTCATCCATCCTGCACTTCTCCACTCCAATTCGGATTTATTGGCAAAAAGGCAAAGAAATCGCTTTCTTATTCCATTCCACTCGATTCAAGAACAAGAGAAAGAGCTAATGCCTCCTTCAGGTATCTCGATTGAAATACCCCTTAGGGGTATTTTCCGTAGAAATAGTATTCTTGCTTATTTCGATGATCCTCAATACAAAAGAAAGAGTTCCGGAATTACTAAATATGGGACTCTGGGGACGCATTCAATCGTTAAAAAAGAGGACTTGATTGAGTATCGAGGACTCAAAAAAATTAAGCCAAACTACCAAATGAAAATAGACCGCCTTTTTTTCATTCCGGAGGAAGTGCATATTTTTCCCGAATCTTCTTACCTAATGGTACGGAATAATAGTATCATTGGAGTAGATACACAAATTGCTTTAAATATAAGAAGCCGAGTGGGTGGATTGGTTCGAGTGGAGAGAAAAAAAGGCGGGCTTGAACTAAAAATATTTTCGGGAGATATCCATTTTCCCGGAGAGATCGATAAGATATCCCGGCACAGTGGCATCCTGATACCGCCCGAAAGTGAAAAAAAAAAACTTAAGGAAGCCACTAAGGAATCAAAAAAATTGAAAAAATGGATCTATGTTCAACGGATCACACCTACCAAGAAAAAGTTTTTTGTTTTGGTGCGACCCGTAGTCACATATGAAATAGCAGACGGTATAAATTTAGCAACACTCTTCCCCCAGGATCCGCTGCGGGAAAAGGATAATATGAAATTTCGAGTTGTCAATTATGTCCTTTATGGGAGGGGCAAAGCTAAAACAGCTCGTGGAATTTCTGATCCAAGTATTCAATTAGTTCGGACGTGTTTAGTGTTGAATTGGGACCAAGACAAAAAAAGTGCTTCCGTCGAAGGGGTTTGTGCTTCCTTTGTTGAAGTACGGACAAATGGTCTGATTCGAGATTTCTTAAGAATCCACTTAGTGGAATCCCATATTTCGTATATCAGAAAAAGGAACCATCTGTCGGGTTCAGGATTTTTTTCTGATAATGGTTCAGCTCGCGCCAATAGCAATCCGTTTTATTCCGTTTTTGGCAAGTCAGGGGTTGAACAATCACTTAGCCAAAATCAAGGAACTATTCGTACGTTGTTGAATAAAAATAAGGAACGCCAATCTTTGATAATTTTGTCATCATCTAATTATTTTCGAATGGGTCCATTGAACGATGTAAAATATCACAATGTGATAAAACAAACAATTCCAATTCAAAAAGATTCTCTAACTCCAATTAGGAGTTCGTTGGGATCCTTAGGAACCGCCCTTCAAATTGCGAATTTTTATTCATTTTACTATTTAATAACTCATAATCATATCTCGGTAACTAAATATTTGCAACTTGACAATTTCAAACAGCCTTTTCAAGTACTTAAATATTATTTAATGGACGAAAACTGGGAAATTTATAATCCTGATACATACAGTAAGATCATTTTGAATCCATTTAATTTGAATTGGCATTTTCCCCATCATAATTATAATTATTGTGAGGAAAGGGACCCGATAATTAGTCTTGGGCAGTTTCTTTGTGAAAATGTATGTATAACCAAAAAGGGACCACACCTAAAATCCGGTCAAGTTTTAATTGTTCAAGTTAACTCTGTAGTAATAAGATCAGCTAAGCCTTATTTGGTTACTCCTGGAGCAACTGTTCATGGGCATTATGGAGAAATCCTTTACGAAGGGGATACATTAATTACATTTATATATGAAAAATCTAGATCCGGTGATATAACGCAGGGTCTTCCAAAAGTAGAACAAGTGTTAGAAGTGCGTTCCCTTGATTCAATATCGATGAACCTGGAAAAGAGAGTTGAGGGTTGGAACGCGCGTATAACAAGAATTCTTGGGATTCCCTGGGGATTCTTGATTGGTGCGCAGCTAACTATAGTGCAAAGTCGTATCTCTTTGGTTAATAAGATCCAAAAGGTTTATCGATCACAGGGGGTGCAGATCCATAATAGGCATATAGAAATTATTGTACGGCAAATAACATCAAAAGTCTTGGTTTCAGAAGATGGAATGTCTAATATTTTTTTACCCGGTGAACTGATCGGATTGGTACGAGCGGAACGAACGGGGCGTGCTTTGGAAGAAGTGATCCGTTATCGAGCTATCTTATTGGGAATAACCAGAGCATCTCTGAATACTCAAAGTTTTATATCCGAAGCGAGTTTTCAAGAAACCACGCGAGTTTTAGCAAAAGCCGCTCTTCGAGGTCGTATCGATTGGTTGAAAGGCCTGAAAGAAAACGTTGTTCTGGGGGGGATAATACCCGTAGGTACCGGATTCAAAGGATTAGTGCACTATTCAAGGCAGCATAACAACATTCTTTTGGAACGACAAAAAAAGAATTTATTCGGGGGGGGAAATGAGAGATATTTTCTTACACTACAAAGAATTAGTCGCCTCTTGCATTTCAACGACTTCCCATGA

>rpoC1

ATGATTGATCGATATAAACATCAACAACTCCGAATTGGATCAGTTTCTCCTCAACAAATAAGTGCTTGGGCCAATAAAATCCTACCTAATGGAGAGATAGTTGGAGAAGTGACAAAACCCTATACTTTTCATTACAAAACCAATAAACCGGAAAAAGATGGATTATTTTGTGAAAGAATTTTTGGGCCTATTAAAAGTGGAATTTGTGCTTGTGGAAATTATCGAATAATCGGAGATGAAAAAGAAGACCCGCAATTTTGCGAACAATGTGGAGTTGAATTTGTGGATTCTCGGATACGAAGATATCAAATGGGATACATAAAACTGGGATGCCCAGTAACCCACGTGTGGTATTTGAAACGTCTTCCTAGTTATATCGCGAATCTTTTAGATAAACCTCTTAAAGAATTAGAAGGCCTAGTATTTTCTTTTGCTAGGCCTATAGCAAAAAAACCTACTTTTTTACGATTACGAGGTTCATTCGAATATGAAATACAATCCTGGAAATACAGCATCCCACTTTTTTTTACTACCCAAGGTTTCGATAAATTTCGTAACCGAGAAATTTCTACTGGAGCAGTTGCTATCCGGGAACAATTAGCCGATCTCGATTTGCGAACTATTCTAGATTATTCCTTGTTAGAATGGAAAGAATTAGGGGAAGAAGGGCCCGCAGGTAATGAATGGGAAGATCGAAAAATTGGACGAAGAAGGGATTTTTTGGTTAGACGCATGGAATTAGCTAAGCATTTTCTTCGAACAAATATAGAACCGGAGTGGATGGTTTTATGTCTATTACCCGTTCTTCCTCCCGAGTTGAGACCGATCATTCAGATAGATGGAGGTAAACTAATGAGTTCCGATATTAATGAACTCTATAGAAGAGTTATCTATCGAAACAATACTCTTACCGATCTATTAACAACAAGTAGATCTACGCCAGGGGAATTAGTAATGTGCCAGGAGAAATTGGTACAAGAAGCCGTGGATACGCTTCTTGATAATGGAATCCGCGGCCAGCCAATGAGGGATGGTCATAATAAGGTTTATAAGTCTTTTTCAGATGTAATTGAAGGAAAAGAGGGAAGATTTCGTGAGACTCTGCTTGGCAAACGGGTCGATTATTCGGGGCGTTCTGTCATTGTTGTAGGCCCCTCACTTTCATTACATCGATGTGGATTGCCTCGCGAAATCGCAATAGAGCTTTTCCAGACTTTTGTAATTTGTGCGCTAATTAGACAACATCTTGCCTCGAACATAGGAGTTGCTAAGAGTAAAATTCGGGAAAAGGGGCCGATTGTATGGGAAATACTGGAAGAAGTTATGCAGGGACATCCAGTATTGCTGAATAGGGCGCCTACTCTGCATAGATTGGGCATACAAGCATTCCAGCCCATTTTAGTGGAAGGGCGTGCTATTTGTTTACATCCATTAGTTTGTAAGGGATTCAATGCTGACTTTGACGGGGATCAAATGGCTGTTCATGTACCTTTATCTTTAGAGGCTCAAGCAGAGGCCCGTTTACTTATGTTTTCTCATATGAATCTCTTGTCTCCGACTATTGGCGATCCCATTTCCGTACCGACTCAAGATATGCTTATTGGACTCTATGTATTAACGAGCGGGAATCGTCGAGGTATTTGTGCAAATAGGTATAATCCGTGTAATCGAAGAAATTATCAAGATGAAAGAATTGATGATAATAGCTATAAGTATACGAAAGAACCCCTTTTTTGTAATTCCTATGATGTAATTGGAGCTTATCGGAAGAAAAGAATCAATTTAGATAGTCCCTTGTGGCTCCGGTGGCGATTAGATCAACGCGTGATTGCTTCAAGGGAAGCTCCCATTGAAGTTCACTATGAATCTTTGGGTACCTATCATGAGATTTATGGGCATTATCTAATAGTACGAAGTGTAAAAAATGAAATTCTTTCGATATACATTCGAACTACCGTTGGCCATATTTCTCTTTATCGAGAAATCGAAGAAGCTATACAAGGGTTTTGTCGGGCCGGCGCATATGGTACCTAA

>rpoB

ATGATCGGAGATGGAAATACGGGAATGTCGACAATACCTGGATTGAATCAGATCCAATTTGAAGGATTTTGTAGGTTCATTGCTCAGGGCTTAACAGAAGAACTTTATAAGTTTCCAAAAATTGAAGATACAGATCAAGAAATTGAATTTCAATTATTTGTGGAAACATATCAATTGGTAGAACCCTTGCTAAAAGAAAGGGATGCTGTATCTGAATCATTCACGTATTCTTCTGAATTATATGTATCCGCAGGATTAATTTGGAAAAGCCGCGGGGATATGCAGGAACAAACTATTTTTATTGGAAACATTCCTCTAATGAATTCTTTGGGAACTTCTATAGTAAATGGAATATACAGAATTGTCATCAATCAAATATTGCAAAGTCCCGGTATCTATTATCGGTCAGAATTGGGCCATAACGGAATTTCGGTCTATACAGGCACGATAATATCCGATTGGGGAGGAAGATTAGAATTAGAGATTGATAGAAAAGCAAGGATATGGGCTCGTGTGAGTAGGAAACAGAAAGTATCTATTCTAGTTTTATTAGCAGCTATGGGTCTAAATCTACGAGAAATTCTAGAGAATATTTGCTACCCTGAAATGTTCTTGTCTTTCCTGACGAATAAGGAGAAAAACCAAATTGGATCAAAAGAAAATGCCATTTTGGAGTTTTATCAACAATTTGCTTGTGTAGGCGGAGATCCGGTATTTTCGGAATCCTTATGTAAGGAATTACAAAAGAAATTTTTTCACCAAAGGTGTGAATTAGGAAAGATTGGTCGACGAAATATGAACCGGAGATTGAATCTTAATATACCTCCGAATAATACATTTTTGTTACCACGAGACGTATTGGCAGCTGTCGATCATTTGATTGGACTGAAATTTGGCATGGGTACACTTGACGATATGAATCATTTGAAAAATAAACGTATTCGGTCTGTAGCGAATCTTTTACAAGATCAATTCGGATTGGCCCTGGTTCGGTTAGAAAATGTGATTAGAGGAACTATAGGCGGAGCAATTAGACATAAATTGATGCCAACTCCTCAAAATTTGGTAACTTCAACCCCATTAACAACCACTTATGATTCTTTTTTCGGATTACATCCATTATCTCAAGTTTTGGATCGAACTAATCCATTGACACAAATAGTTCATGGGAGAAAGTTGAGTTATTTGGGCCCCGGAGGATTGACAGGACGAACTGCTAGTTTTCGGATACGAGATATCCATCCTAGTCACTACGGCCGCATTTGCCCAATTGACACGTCTGAAGGAATCAATGTTGGGCTTATTGGATCCTTAGCAATTCATGCGAGAATTGGTTATTGGGGGTCTCTCGAAAGCCCATTTTTTGAAATCTTTAAAAAATCAAAAAAAGTACGGATGCTTTATTTATCACCAAGTAGAGATGAATACTTTATGGTAGCGGCAGGAAATTCTTTGGCACTGAATCAGGGTAGTCAGGAAGAACAGATTGTTCCGACTCGATACCGTCAAGAATTCCTGACTATTGCGTGGGAACAGGTTCATCTTCGAAGTATTTTTCCCTTCCAATATTTTTCTATTGGGGCTTCCCTCATTCCTTTTATCGAGCACAATGATGCGAATCGGGCTTTAATGAGTTCTAATATGCAACGCCAAGCAGTTCCGCTCTTTCGGTCCGAGAAGTGCATTGTTGGAACTGGGTTGGAACGCCAAGTGGCGCTAGATTCAGGGGTTCCCGCTATAGCCGAACATGAGGGAAAGATCATTTATACCGATATTGACAAGATTGTTTTATCGGGAAGCGGTAATACTTATAGTATTCCGTTAATTATGTATCAACGTTCAAACAAAAATACTTGTATGCATCAAAAAGCCCAGGTTAGGCGGGGTAAATGCATTAAAAAGGGCCAAGTTTTAGCGGACGGCGCCGCTACAGTTGGTGGCGAACTCGCTTTAGGGAAAAACATATTAGTAGCTTATATGCCATGGGAGGGCTACAATTTTGAGGATGCGGTACTTATTAGCGAACGTCTGATATATCGAGATATTTATACTTCTTTTCACATACGGAAATATGAAATTCAGACCCATGTGACAAGCCAAGGTCCCGAAAGGATCACTAATGAAATACCACATCTAGAAGCCCTTTTACTCCGCAATTTAGATAAAAACGGAATTGTGATGCTGGGATCTTGGGTAGAGACCGGGGATATTTTAGTAGGTAAATTAACGCCTCAAGCGGCGAAAGAATCATCGTATGCTCCGGAAGATAGATTATTACGGGCCATACTTGGCATTCAGGTATCCGCTTCAAAGGAAACTTGCCTAAAACTACCTATAGGTGGGCGGGGCCGCGTTATTGATGTGAGATGGGTCCAGAAAAAGGGGGGTTCTAGTTATAATCCAGAAACGATTTGTGTATATATTTTACAGAAACGTGAAATCAAAGTAGGTGATAAAGTGGCCGGAAGACATGGAAATAAGGGTATCATTTCTAAAATTTTGCCTAGACAGGATATGCCTTATTTGCAAGATGGAAGGCCTGTTGATATGGTTTTCAACCCATTAGGAGTACCCTCGCGAATGAATGTAGGACAGATATTTGAATGCTCGCTGGGGTTAGCAGGGAGTCTGCTAAATCGACATTATCGAATAGCACCTTTTGATGAGAGATATGAACAAGAGGCTTCGAGAAAACTCGTGTTTTCTGAATTATATGAAGCCGGTAAGCAAACCGCGAATCCATGGGTATTTGAGCCGGAATACCCGGGAAAAAGCAGAATATTTGATGGACGAACGGGAGATCCTTTTGAACAACCTGTTCTAATAGGAAAGGCTTATATCTTGAAATTAATTCATCAAGTTGATGATAAAGTACACGGACGTTCCAGTGGGCATTATGCACTTGTTACCCAACAACCCCTTAGAGGAAGGTCCAAACAAGGGGGACAACGGGTAGGCGAAATGGAGGTGTGGGCTCTAGAGGGCTTTGGTGTGGCTCATATTTTACAAGAGATGCTTACTTATAAATCGGATCATATTAGAGCTCGCCAAGAAGTACTTGCTACTACAATCATTGGAGAAACAATACCTAACCCCGAGGATGCTCCAGAATCTTTTCGATTGCTCGTTCGAGAACTACGATCTTTGGCTTTGGAACTGAATCATTTTCTTGTATCTGAGAAAAACTTCCAGATTAATAAGAAGGAAGCTTAA

>psbM

ATGGAAGTAAATATTCTCGCATTTATTGCTACTACATTGTTCGTTCTAGTTCCTACTGCGTTTTTGCTTATAATATACGTAAAAACGGTCAGTCAAAGTGATTAA

>psbD

ATGACTATAGCCCTTGGTAAATTTACCAAAGATGAAAAGGATTTATTTGATATTATGGATGACTGGTTACGGAGGGACCGATTCGTTTTTGTAGGTTGGTCCGGTCTATTGCTCTTTCCTTGTGCCTATTTCGCTTTAGGGGGTTGGTTTACAGGCACAACCTTTGTAACTTCATGGTATACCCATGGATTGGCCAGTTCCTATTTGGAAGGCTGCAACTTCTTAACTGCGGCAGTTTCGACTCCTGCTAATAGTTTAGCGCATTCTTTGTTGTTACTATGGGGTCCTGAAGCACAAGGAGATTTTACTCGTTGGTGTCAATTAGGCGGTCTTTGGACTTTTGTTGCTCTCCACGGTGCTTTCGGACTAATAGGTTTCATGTTACGTCAATTTGAGCTTGCTCGCTCTGTTCAATTGCGACCTTATAATGCAATATCATTCTCTGCTCCAATTGCTGTTTTTGTTTCTGTATTCCTGATTTATCCACTAGGTCAGTCTGGTTGGTTCTTTGCGCCTAGTTTTGGTGTAGCGGCAATATTTCGATTTATCCTTTTTTTCCAAGGGTTTCATAATTGGACCTTGAACCCCTTTCATATGATGGGAGTTGCCGGTGTATTGGGCGCTGCTCTGCTATGTGCTATTCATGGCGCTACCGTAGAAAATACTTTATTTGAAGATGGTGATGGTGCAAATACATTCCGTGCTTTTAACCCAACGCAAGCCGAAGAAACTTATTCGATGGTCACCGCTAACCGCTTTTGGTCCCAAATCTTCGGGGTTGCTTTTTCCAATAAACGTTGGTTACATTTCTTTATGTTATTTGTACCAGTAACCGGTTTATGGATGAGTGCTCTTGGAGTAGTCGGCCTAGCCCTGAACCTACGTGCTTATGACTTTGTTTCCCAGGAAATCCGTGCAGCGGAAGATCCTGAATTTGAGACTTTCTACACAAAAAATATTCTTTTAAACGAGGGTATTCGTGCTTGGATGGCGGCTCAAGATCAGCCTCATGAAAACCTTATATTCCCTGAGGAGGTTCTACCCCGTGGAAACGCTCTTTAA

>psbC

ATGAAAACCTTATATTCCCTGAGGAGGTTCTACCCCGTGGAAACGCTCTTTAATGGAACTTTAGCTGTAGCAGGTCGGGACCAAGAAACCACCGGTTTCGCTTGGTGGGCCGGGAATGCCCGACTTATCAATTTATCCGGTAAACTCCTGGGCGCTCATGTAGCCCATGCTGGATTAATCGTATTCTGGGCCGGAGCAATGAACCTATTTGAAGTGGCTCATTTCGTACCAGAAAAGCCCATGTATGAACAAGGATTAATTTTACTTCCCCACCTAGCTACTCTAGGCTGGGGGGTAGGTCCTGGTGGGGAAGTTATAGACACCTTTCCATACTTTGTATCTGGAGTACTTCATTTAATTTCCTCCGCAGTATTGGGCTTTGGCGGGATTTATCACGCACTTCTGGGACCTGAGACTCTTGAAGAATCTTTTCCATTCTTCGGTTATGTATGGAAAGATAGAAATAAAATGACCACAATTTTGGGTATTCACTTAATCTTGTTAGGTATCGGTGCTTTTCTTCTAGTATTCAAGGCTCTTTATTTTGGGGGCGTGTATGATACCTGGGCTCCGGGGGGTGGAGATGTACGAAAAATTACCAACTTGACCCTTAGCCCAAGTGTTATATTTGGTTATTTACTAAAATCCTTCTTTGGAGGAGAGGGGTGGATTGTTAGTGTGGATGATTTGGAAGATATAATTGGAGGGCATGTATGGTTAGGTTCCATTTGTATATTTGGTGGAATCTGGCATATCTTAACCAAACCTTTTGCATGGGCTCGCCGTGCACTTGTATGGTCTGGGGAGGCTTACTTGTCTTATAGTTTAGCTGCTTTATCTGTTTTTGGTTTCATTGCTTGTTGCTTTGTCTGGTTCAATAATACCGCTTATCCTAGTGAGTTTTATGGGCCCACTGGTCCAGAAGCTTCTCAAGCTCAAGCATTTACTTTTCTAGTTAGAGACCAACGTCTTGGGGCTAACGTGGGATCTGCGCAAGGGCCTACTGGGTTAGGTAAATATCTAATGCGGTCCCCCACTGGAGAAGTCATTTTTGGGGGAGAAACAATGCGTTTTTGGGATCTGCGAGCTCCATGGTTAGAACCTCTAAGGGGTCCCAACGGTTTAGACTTGAGTAGGTTGAAAAAAGACATACAACCTTGGCAAGAACGACGTTCTGCGGAATATATGACTCATGCCCCTTTAGGGTCTTTAAATTCTGTGGGTGGCGTAGCTACCGAGATCAATGCAGTTAATTATGTCTCTCCGAGAAGTTGGTTAGCTACTTCTCATTTTGTTCTAGGCTTCTTCCTATTCGTAGGTCATTTATGGCACGCGGGAAGGGCTCGTGCAGCTGCCGCAGGGTTTGAAAAAGGAATTGATCGTGATTTTGAACCTGTTCTTTCGATGACTCCTCTTAACTGA

>psbZ

ATGACTATTGCTTTCCAATTGGCTGTTTTTGCATTAATTGCTACTTCATCAATTTTACTGATTAGCGTACCCGTTGTATTTGCTTCTCCGGATGGTTGGTCAAGTAACAAAAATGTCGTATTTTCTGGTACATCATTGTGGATTGGATTAGTCTTTCTGGTGGGTATCCTTAATTCTATCATCTCTTAA

>rps14

ATGGCAAAGAAAAGTTTGATTCAGAGGGAGAAGAAAAGGCAAACATTGGAACAAAAATATCATTTGATTCGTCGATCCTCAAAAAACGAAATAAAAAAAGTTCCCGCGTTGAGCGATAAATGGAAAATTCATGGAAAGTTACAATCCTTACCGCGTAATAGTGCACCTACACGTCTTCATCGACGTTGTTTTTCGACCGGAAGGCCGCGAGCTAACTATCGAGACTTTGGACTATCCGGACACATACTTCGTGAAATGGTTCATGCGTGTTTGTTGCCGGGGGCAACAAGATCAAGTTGGTAA

>psaB

ATGGCATTAAGATTTCCAAGGTTTAGCCAAGGCTTAGCTCAGGACCCCACTACTCGTCGTATTTGGTTTGGTATTGCTACCGCGCATGACTTCGAGAGTCATGATGATATTACTGAGGAACGTCTTTATCAGAATATTTTTGCTTCGCACTTCGGGCAATTAGCAATAATTTTTCTGTGGACTTCCGGAAATCTCTTTCATGTAGCTTGGCAAGGAAATTTCGAGGCATGGGTACAGGACCCTTTACATGTAAGACCGATTGCTCATGCAATTTGGGATCCTCATTTTGGTCAACCGGCCGTGGAAGCTTTTTCTCGGGGAGGTGCTCTTGGTCCGGTGAATATCGCTTATTCTGGTGTTTATCAGTGGTGGTATACAATTGGTTTACGCACTAATGAGGATCTTTATACTGGAGCTCTTTTTCTATTATTTCTTTCCGCCATATCCTTAATAGCGGGTTGGTTACACCTACAACCTAAATGGAAACCGAGCGTTTCGTGGTTCAAAAATGCCGAATCTCGTCTCAATCATCATTTGTCAGGATTGTTTGGAGTAAGTTCCTTGGCTTGGACAGGGCATTTAGTACATGTCGCTATTCCTGGATCCAGGGGGGAATATGTTCGATGGAATAATTTCTTAGATGTATTACCGCATCCCCAAGGGTTAGGCCCGCTTTTTACAGGTCAGTGGAATCTTTATGCTCAAAACCCCGATTCAAGTAGTCATTTATTTGGTACCTCTCAAGGATCAGGAACTGCCATTCTAACCCTTCTTGGGGGATTCCATCCACAAACGCAAAGTTTATGGCTGACCGATATGGCTCATCATCATTTAGCTATTGCATTTATTTTTCTCGTTGCTGGTCATATGTATAGAACGAATTTCGGGATTGGGCACAGTATAAAAGATCTTTTAGAAGCACATATTCCTCCGGGAGGACGATTGGGGCGCGGGCATAAGGGTCTTTATGACACAATCAACAATTCGCTTCATTTTCAATTAGGCCTTGCTCTAGCTTCGTTAGGGGTTATTACTTCCTTGGTAGCTCAACACATGTACTCTTTACCGGCTTATGCATTCATAGCACAAGATTTTACTACTCAAGCTGCATTATATACTCATCACCAATACATCGCGGGATTCATCATGACAGGAGCTTTTGCTCATGGAGCTATCTTTTTTATTAGAGATTACAATCCGGAACAGAATGAGGATAATGTATTGGCAAGAATGTTAGACCATAAGGAAGCTATTATATCCCATTTAAGTTGGGCCAGCCTGTTTCTGGGGTTCCATACTTTGGGACTTTATGTTCATAATGATGTCATGCTTGCTTTTGGTACTCCGGAGAAACAAATCTTGATCGAACCGATATTTGCCCAATGGATACAATCTGCTCATGGTAAAACTTCATATGGATTTGATGTACTTTTATCTTCAACGAATGGCCCTGCCTTCAATGCGGGTCGAAGCATATGGTTGCCTGGCTGGTTAAATGCTATTAATGAGAATAGTAATTCTCTATTCTTAACAATAGGCCCAGGGGACTTCTTGGTTCATCATGCTATTGCTCTGGGTTTACATACAACTACATTGATCTTAGTAAAAGGGGCTTTAGATGCACGTGGTTCCAAGTTAATGCCAGATAAAAAGGATTTCGGTTATAGTTTTCCTTGCGACGGTCCGGGGCGAGGCGGTACTTGTGATATTTCGGCTTGGGACGCATTTTATTTGGCAGTTTTCTGGATGTTAAATACCATTGGGTGGGTTACGTTTTATTGGCATTGGAAACACATCACGTTATGGCAGGGTAACGTTTCACAGTTTAATGAATCTTCCACTTATTTGATGGGGTGGTTAAGAGATTATCTATGGTTAAACTCTTCCCAACTTATCAATGGGTATAACCCGTTTGGTATGAATAGTCTATCAGTCTGGGCGTGGATGTTCTTATTTGGACATCTTGTTTGGGCTACTGGATTTATGTTCTTAATTTCCTGGCGCGGGTATTGGCAAGAATTGATTGAAACTTTAGCATGGGCTCACGAACGCACACCCCTGGCTAATTTGATTCGATGGAGAGATAAACCAGTGGCTCTTTCCATTGTGCAAGCACGATTGGTTGGGTTAGCCCACTTCTCTGTAGGTTATATATTCACTTATGCGGCTTTCTTGATTGCTTCTACATCGGGGAAATTTGGTTAA

>psaA

ATGATTATTCGTTCGCCGGAACCAGAAGTAAAAATTTTGGTAGATAGGGATCCCGTAAAAACTTCTTTCGAGGAATGGGCCAAACCGGGTCATTTCTCAAGAACCATAGCTAAGGGACCTGAGACTACCACTTGGATCTGGAACCTACATGCCGATGCTCACGACTTTGATAGCCATACCAGTGATTTGGAGGAAATCTCTCGAAAAGTATTTAGTGCCCATTTCGGACAACTCTCCATCATTTTTCTTTGGCTGAGCGGCATGTATTTCCACGGTGCTCGTTTTTCCAATTATGAAGCCTGGCTAAGCGATCCGACTCACATTGGACCTAGTGCACAGGTGGTTTGGCCAATAGTGGGCCAAGAAATATTGAATGGTGATGTGGGCGGTGGTTTCCGAGGAATACAAATAACCTCCGGGTTTTTTCAGCTTTGGCGAGCATCTGGAATAACTAGTGAATTACAACTCTATTGTACCGCAATTGGCGCATTGATTTTTGCAGCCTTAATGCTTTTTGCGGGTTGGTTCCATTATCACAAAGCTGCTCCAAAATTGGCTTGGTTTCAGGATGTAGAATCCATGTTAAATCACCATTTAGCGGGGCTGCTAGGGCTCGGGTCCCTTTCTTGGGCCGGGCATCAAGTACATGTATCTTTACCGATTAACCAATTTCTAAATGCTGGAGTAGATCCTAAAGAGATCCCACTTCCTCATGAATTTATTTTGAACCGGGATCTTTTGGCTCAACTTTATCCCAGTTTTGCTGAAGGAGCAACCCCATTTTTTACCTTGAATTGGTCAAAATATGCGGACTTTCTTACTTTTCGTGGCGGATTAGATCCAGTAACTGGGGGTCTATGGCTGACCGATATTGCACACCATCATTTAGCTATTGCAATTCTTTTCCTCATCGCGGGTCACATGTATAGGACCAACTGGGGGATTGGTCATGGTCTAAAAGATATTTTAGAGGCTCATAAAGGTCCATTTACAGGTCAAGGCCATAAAGGACTGTATGAGATCCTAACAACATCGTGGCATGCTCAATTATCGCTTAACCTAGCTATGTTAGGATCTTTAACCATTGTTGTAGCGCATCATATGTATTCCATGCCCCCTTATCCATATCTAGCTACTGACTATGGTACACAACTGTCATTGTTCACGCATCACATGTGGATTGGTGGATTTCTCATCGTTGGTGCTGCTGCGCATGCAGCCATTTTTATGGTAAGAGACTATGATCCAACTACTCGCTACAACGATCTCTTAGATCGTGTCCTGCGGCACCGCGATGCAATCATATCACACCTCAATTGGGTATGTATATTTCTAGGATTTCACAGTTTTGGTTTGTATATTCATAATGATACAATGAGTGCTTTAGGGCGTCCACAAGATATGTTTTCAGATACCGCGATCCAATTACAACCTGTCTTCGCTCAATGGATACAAAACACCCACGCCTTAGCACCCGTTGGAACGGCTCCTGGTGCAACAGCAAGCACCAGTTTGACTTGGGGGGGCGCTGATTTAGTGGCAGTGGGTGGAAAAGTTGCCTTGTTGCCTATTCCATTAGGTACCGCGGATTTTTTGGTACATCACATTCATGCATTTACGATTCATGTGACGGTATTGATACTCCTGAAAGGAGTTCTCTTTGCTCGTAGCTCGCGTTTGATACCGGATAAAGCAAATCTTGGGTTTCGTTTCCCTTGTGATGGGCCTGGAAGAGGGGGGACATGTCAAGTATCCGCTTGGGATCATGTCTTCTTAGGACTATTCTGGATGTACAATGCAATTTCGGTAGTAATATTCCATTTCAGTTGGAAAATGCAGTCAGATGTTTGGGGTAGTATAAGCGATCAGGGGGTGGTAACTCATATTACAGGAGGAAACTTCGCACAGAGTTCCATTACGATTAATGGGTGGCTCCGCGATTTTTTATGGGCACAGGCATCCCAGGTAATCCAGTCTTATGGTTCTTCATTATCCGCATATGGCCTTTTTTTCCTAGGTGCTCATTTTGTATGGGCTTTTAGTTTAATGTTTCTATTCAGCGGGCGTGGTTATTGGCAAGAACTTATTGAATCCATCGTTTGGGCTCATAATAAATTAAAAGTTGCTCCTGCTACTCAGCCTAGAGCCTTGAGCATTGTACAAGGACGTGCTGTAGGAGTAACCCATTACCTTCTGGGTGGAATTGCCACAACATGGGCGTTCTTCTTAGCAAGAATTATTGCAGTAGGATAA

>ycf3

ATGCCTAGATCGCGGATCAATGGAAATTTTATTGATAAGACCTTTTCAATTGTAGCCAATATATTATTACGAATAATTCCGACAACCTCAGGAGAAAAAGAGGCATTTACCTATTACAGAGATGGTGCGATGTCAGCTCAATCTGAAGGCAATTATGCGGAAGCTTTACAGAATTATTATGAAGCTATGCGACTAGAAATTGATCCCTACGATCGAAGCTATATACTCTATAACATAGGCCTTATCCACACGAGTAACGGCGAACATACAAAAGCTTTAGAATATTATTTTCGGGCACTAGAACGAAATCCGTTCTTACCACAAGCTTTTAATAATATGGCTGTAATCTGTCATTACCGGGGAGAACAGGCCGTTCAACAGGGGGATTCTGAAATTGCGGAGGCTTGGTTCAATCAAGCCGCCGAGTATTGGAAACAAGCTATTGCGCTTACTCCTGGTAATTATATTCAAGCGCAGAATTGGTTGAAGATCACGGGGCGTTTCGAATAA

>rps4

ATGTCACGTTACCGAGGGCCTCGTTTCAAAAAAATACGCCGTCTGGGGTCTTTGCCCGGACTAACGAGTAAAAGGCCTAGAGCCGGAAGCGATTTTAGAAACCAATCGCGCTCCGGAAAAAAATCTCAATATCGAATTCGGTTAGAAGAAAAACAAAAATTGCGTTTTCATTATGGTCTTACAGAACGACAATTACTTAAATATGTTCGTATCGCCGGAAAAGCAAAAGGGTCAACCGGTTTGGTTTTACTACAATTACTTGAAATGCGTTTGGATAACATTCTTTTTCGATTGGGTATGGCTTCAACTATTCCTCAAGCCCGCCAATTAGTTAACCATCGACATATTTTAGTTAATGGGCGTATAGTCGATATACCAAGTTATCGTTGCAAACCCCAAGATATTATTACTGCAAGGGATGACCAAAAATCTAGATCTCTGATTCAAAATTATCTTGATTCATCCCACCGCGAGGAGTTGCCAAAACATTTGACTCTTCACGCATTCCAATATAAAGGATTAGTCAATCAAATAATAGATAGTCAATGGGTCGGTTTGAAAATAAACGAATTGCTTGTCGTAGAATATTATTCTCGTCAGACTTAA

>ndhJ

ATGCAGGGTCGTTTGTCTGCTTGGCTAGTCAAGCATGGGCTAGTTCATAGATCTTTGGGCTTCGATTACCAAGGAATAGAAACGTTACAAATAAAGCCCGAGGATTGGCATTCCGTTGCTGTCATTTTGTATATATATGGGTACAATTATCTACGTTCCCAATGTGCCTATGATTTAGCACCGGGTGGGCTGTTAGCCAGTGTGTATCATCTTACGCGAATAGAGTATGGGGTAGATCAACCAGAAGAGGTATGCATAAAAGTATTTGCTCCAAGGGGTAATCCTAAAATTCCATCGGTTTTCTGGGTTTGGAAAAGCGCGAATTTTCAAGAAAGGGAATCTTATGATATGTTGGGAATCTTTTATGATAATCATCCACGAATGAAACGTATCTTAATGCCAGAAAGTTGGATCGGGTGGCCCTTGCGTAAGGATTATATTGCCCCCAATTTTTATGAAATACAAGACGCTTATTGA

>ndhK

ATGAATTCCATTGAATTTTCCTTACTTGATCGAACAACTCCAAATTCATTTATTTCAACTACATCAAACGATCTTTCAAATTGGTCAAGACTCTCCAGTTTATGGCCGCTTCTGTATGGTACTAGTTGTTGTTTCATTGAATTTGCTTCATTAATAGGCTCGCGGTTCGATTTTGACCGTTATGGGCTAGTACCAAGATCGAGCCCGAGGCAGGCGGACCTAATTTTAACAGCGGGGACAGTAACAATGAAAATGGCTCCTTCTTTAGTTCGATTATATGAACAAATGCCTGAACCAAAATATGTTATTGCTATGGGAGCATGTACAATTACAGGGGGGATGTTTAGTACGGATTCGTATAGTACTGTTCGGGGAGTTGATAAGCTAATCCCGGTTGATGTTTATTTGCCGGGTTGTCCGCCTAAACCAGAGGCGGTTATAGATGCCATAACAAAACTTCGGAAGAAAGTATCTCGCGAAATCTATGAAGATCGAATTCGATTGCAACGGGAAAATCGCGCGTTTATTTTTACTACCAATCACAAGTTTCGTGTTGTAGGCAGTACTCATGCTGGAAATTTTGATCAAGGATTACTCTATCAACCACCATCTCCGTCAGAAATCACGCCTGAAACCTTTTTCAAATACAAAAGTTCAGTATCTTCCCCCCGAATTCATTAA

>ndhC

ATGTTTCTGCTTTACAAATATGATATTTTCTGGGCATTTCTAATAATATCAAGCGTTATTCCTATTTTGGCATTTCTAATTTCCGGAGTTTTAGCCCCGATTAACAAAGGGCCAGAGAAACTTTCTAGTTATGAATCGGGTATCGAACCAATTGGCGATGCTTGGTTACAATTCCGAATCCGGTATTATATGTTTGCTCTAGTTTTTGTTGTTTTTGATGTTGAAACCGTTTTTCTTTATCCATGGGCAATGAGTTTGGATGTATTGGGGGTATCCGTATTTATAGAAGCTTTCATTTTCATGCTTATCCTAATAGTTGGCTCAATTTACGCGTGGCGAAAAGGAGCATTAGAGTGGTCTTAG

>atpE

ATGACCTTAAATCTTTGTGTACTGACCCCTAATCGAATTGTTTGGGATTCAGAAGTGAAAGAAATAATTTTATCTACTAATAGTGGACAAATAGGCGTATTATCAAATCACGCGCCTATTGCCACAGCTGTAGATATAGGTATTTTAAGAATCCGCTTTAATGACCAATGGTTAACGATGGCTCTGATGGGTGGTTTTGCTAGAATAGGGAATAATGAGATTACTATTTTAGTAAATGATGCGGAGAAGAGTAGCGACATTGATCCCCACGAAGCCCGGCAAACTCTTGAAATAGCAGAAGAAAATTTGAGGAAAGCTGAAAGCAAGAGACAAACAATTGAGGCAAATCTAGCTCTCAGACGAGCTAGGACACGAGTAGAGGTTATCAATGCGATTTCTTAA

>atpB

ATGAGAATAAATCCTACTCCTTCTGGTTCCGGGGTTTCCGCGCTTGCAAAAAAAAACCTGGGACAGATCGCTCAAATCATTGGTCCGGTACTGGATGTAGCCTTTCCCCCCGGCAAGATGCCGAATATTTACAATGCTCTGGTAGTGAAGGGGCGAGATACTGGTGGTCAACCAATTAATGTGACTTGCGAGGTACAGCAATTATTAGGAAATAATCGAGTTAGAGCTGTAGCCATGAGTGCTACAGATGGTCTAACGAGAGGTATGGAAGTGATTGACACGGGAGCTCCTCTAAGTGTTCCAGTCGGCGGAGCGACTCTAGGACGAATTTTCAACGTGCTTGGGGAGCCTGTTGATAATTTAGGTCCTGTAGATACTCGCACAACATCCCCTATTCATAAATCCGCGCCTGCCTTTATACAGTTAGATACAAGATTATCGATTTTTGAAACAGGAATTAAAGTAGTAGATCTTTTAGCCCCTTATCGGCGGGGAGGAAAAATCGGACTATTCGGGGGAGCTGGGGTGGGTAAAACAGTACTCATTATGGAATTGATCAACAACATTGCCAAAGCCCATGGGGGTGTATCCGTATTTGGCGGAGTCGGTGAACGTACTCGGGAAGGAAATGATCTTTACATGGAAATGAAAGAGTCTGGAGTAATTAATGAGCAAAATATTGCGGAATCAAAAGTGGCTCTAGTCTACGGTCAGATGAATGAACCGCCAGGAGCTCGTATGAGAGTTGGTTTGACGGCCCTAACGATGGCAGAATATTTCCGAGATGTTAATGAACAAGACGTACTTCTATTTATCGACAATATCTTCCGTTTCGTCCAAGCGGGATCCGAGGTATCCGCCTTATTAGGTCGAATGCCTTCCGCTGTAGGTTATCAACCTACCCTTAGTACCGAAATGGGTTCTTTACAAGAAAGAATTACTTCTACCAAAGAGGGGTCCATAACTTCTATTCAAGCAGTTTATGTACCTGCAGATGATTTGACCGACCCCGCTCCTGCCACGACATTTGCCCATTTAGATGCTACTACCGTACTATCAAGAGGATTAGCTGCTAAAGGTATCTATCCAGCAGTTGATCCTTTAGACTCAACGTCAACTATGCTCCAACCTCGGATCGTTGGCGAGGAACATTATGACACTGCGCAAAGAGTTAAGGAAACTTTACAACGTTACAAAGAACTTCAGGACATTATAGCTATCCTTGGGTTGGACGAATTGTCCGAAGAGGATCGTTTAACCGTAGCAAGAGCACGAAAAATTGAACGTTTCTTATCACAACCCTTTTTCGTAGCAGAAGTATTTACCGGTTCCCCGGGGAAATATGTTGGTCTGGCCGAAACTATTAGAGGGTTTAAATTGATCCTGTCCGGAGAATTAGATGGTCTTCCTGAACAGGCCTTTTATTTGGTAGGTAACATCGATGAAGTTACCGCGAAGGCTACAAACTTAGAAATGGAGAGTAATTTGAAGAAATGA

>rbcL

ATGTCACCACAAACAGAGACTAAAGCGAGTGTTGGATTCAAAGCCGGTGTTAAAGATTATAAATTGACTTATTATACTCCTGAGTATGTAACCAAAGATACTGATATCTTGGCAGCATTCCGAGTAACTCCTCAACCTGGAGTTCCACCCGAGGAAGCGGGGGCTGCGGTAGCTGCGGAATCTTCTACTGGTACATGGACAACTGTGTGGACCGATGGGCTTACCAGCCTTGATCGTTACAAAGGGCGATGCTACGACATTGAGCCCGTTGCTGGAGAAGAAAATCAATATATATGTTATGTAGCTTACCCGTTAGACCTTTTTGAAGAAGGTTCTGTTACTAACATGTTTACTTCCATTGTGGGTAATGTATTTGGTTTTAAAGCCCTGCGCGCGCTACGTCTAGAGGATCTACGAATCCCTACTGCGTATGTTAAAACTTTCCAAGGCCCGCCTCACGGTATCCAAGTTGAAAGAGATAAATTGAACAAGTACGGCCGTCCCCTGTTGGGATGTACAATTAAACCTAAATTGGGGTTATCCGCTAAGAATTATGGTAGGGCGGTTTATGAATGTCTACGTGGTGGACTTGACTTTACCAAAGATGATGAGAACGTGAACTCCCAACCATTTATGCGTTGGAGGGACCGTTTCTTATTTTGTGCGGAAGCACTTTATAAAGCACAAGCTGAAACAGGTGAAATCAAAGGTCATTACTTGAATGCTACTGCAGGGACATGCGAAGAAATGATCAAAAGGGCTGTCTTTGCCAGAGAGTTGGGAGTTCCTATCGTAATGCATGACTACTTAACCGGGGGATTCACCGCAAATACTAGCTTGGCTCATTATTGCCGAGATAATGGTCTACTTCTTCACATCCACCGGGCAATGCATGCCGTTATTGATAGACAGAAGAATCATGGTATACACTTTCGTGTACTAGCTAAAGCTTTACGTATGTCTGGTGGAGATCATATTCACTCTGGTACAGTAGTAGGTAAACTTGAAGGGGAAAGAGACATTACTTTGGGATTTGTTGATTTACTACGTGATGATTTTATTGACAAAGATCGAAGCCGCGGTATTTATTTCACTCAAGATTGGGTTTCTCTACCAGGTGTTCTACCCGTTGCTTCCGGGGGTATTCACGTTTGGCATATGCCTGCTTTGACCGAGATCTTTGGAGATGATTCCGTACTACAATTTGGTGGAGGAACTTTAGGACACCCTTGGGGAAATGCACCTGGCGCTGTAGCTAATCGAGTAGCTCTAGAAGCATGTGTCCAAGCTCGTAATGAAGGCCGCGATCTTGCTCGCGAAGGCAATGAAATTATTCGAGAGGCTTGCAAATGGAGTCCTGAACTGGCTGCTGCTTGCGAAGTATGGAAGGAGATCAAATTTGAATTTGAAGCAATGGATACTTTGTAA

>accD

ATGCAAAAATGGTGGTTCAATTCGATGTTATCGAAGGGTAAGGGGCAATTCGAATCCAGGTGTGGGTTAAGTAAATCAATGGAGAGTTTTGGTCCTATTAAAAATACCAGTGTAAGCGAAGACCCGATTAGAAATGATAAGGATAAAAACATTCATAGTTCGAGCGATAGTGACAGTTCGAGTTACGGCAATTTAGCTGGTGTCAGGGACATTCATAATTTCATCTCGGATGAAACTTTTTTTGTTAAGGATAGTAATAGGGACAGTTATTCCATCCATTTTGATATTGAAAATCAAATTTTTGAAATAGCCAATGATCATTCTTTTTTGAGTGAACTAGAAAGTTCTTTTGATAACTTTCGTAATTTGAGTTCGAGGAATAATGGATCGAAAAGTGATGATCCCGGCTCAGATCGTTACATGGATGATACTAAATCGAGTTGGCATAATCACATTAATAATTGCCTTGACTCTTATCTTCATTCTCAAATCTGTATTGATAGTCACATTTTAAGTAGTAGTGACAATTATAGTGCCAGTTACATTTATAATTTAGTTTGTAGTGAAAGTGGAAATAGTAGTGAAAGTGAGAGTTCCAATATACAAAATAGCACGAATGGTAGTGGTTTAACTATAAGCGACAGTTCGAAAAACGAAAGTTCGAATGATCTCGATATAACTCAAAAATACAGGCATTTATGGGTTCAATGCGAAAGTTGTTATGGATTAAATTATAAGAAATTTCTTAAGTCAAAAATGTATATTTGTGAACAATGCGGCCATCATTTGAAAATGAGTAGCTCAGATAGAATCGAACTGTCGGTTGATCCAGGTACTTGGGATCCAATGGATGACGACATGGTCTCCATAGATCCCATTGAATTTCAGTCAGAAGAAGAAGATTATAAAAATCGTATTGATTCTTATCAAATAAAGACAGGATTAACGGAGGCTGTTCAAACAGGTACAGGGCAACTAAACGGGATTCCCATCGCAATTGGGGTTATGGATTTTCAGTTTATGGGGGGTAGTATGGGATCCGTAGTAGGTGAGAAAATCACCCGTTTGATCGAGTATGCTGCCACTCAATTTTTACCTCTTCTTATAGTGTGTGCTTCCGGGGGAGCACGCATGCAAGAAGGAAGTTTGAGCTTGATGCAAATGGCTAAAATATCCTCCGCTTTATATGATTATCAATCAATTAAAAAGTTATTCTATGTATCACTTCTTACATCTCCTACTACTGGTGGAGTGACAGCCAGTTTTGGTATGTTGGGGGATATCATTATTGCCGAACCTAATGCCTATATTGCATTTGCGGGTAAAAGAGTAATTGAACAAACATTGAATAAGACAGTACCTGAAGGTTCACAAGCAGCTGAATATTTATTCCATAAGGGCTTATTCGATCCAATCGTACCACGTAATCCTTTAAAAGGTGTTCTGAGCGAGTTATTTCAGTTCCACGACTTTGTTCCTTCCAGTAGAGTTCTTAAGTAA

>psaI

ATGACAATTCTCAACAGCTTTCCCTCTATTTTTGTGCCGTTAGTGGGCCTAGTATTTCCCGCAATGGCAATGGCTTCGTTATTTCTTTATCTTGAAAAAAATAAGATTTTGTAA

>ycf4

ATGAGTTGGCGATCAGAATATATATGGATAGACTTTATAGCGGGCTCTCGAAAAACAAGCAATTTCTGCTGGGCCTTTATCCTTTTTTTAGGTTCATTAGGATTCTTAGCGGTTGGAATTTCTAGTTATCTTGATAGGAATTTGCTATCTTTAGTCCCGTCGCAGCAAATAAATTTTTTTCCGCAGGGGATCGTTATGTCTTTCTACGGGATCGCGGGTCTCTTTATTAGTTCCTATTTGTGGTGCACAATTACATGGAATGTAGGTAGCGGTTATGATCGATTTGATACAAAAGAGGGAATAGTGTGTATTTTTCGTTGGGGATTTCCTGGACAAAATCGCCGCATCTTTCTACGATTCCTTATGAAAGATATTCAGTCCATCAGAATAGAAGTTAAAGAGGGTATTTATGCTCGTCGTGTCCTTTATCTCGAAAGCATAGGCTTGGGAGCCATTCCCTTGAATCGTACTGATGAGAATTTGACGCCACGAGAAATGGAGCAAAAGGCTGCGGAATTGGCCTATTTCTTGCGTGTACCAATTGAAGGGTTTTGA

>cemA

ATGACAAAAAAGAACGTACCCCTTCCCCTTAGATATCTTTCATCTATAGTATTTGTAGTATTTTTGCCCTGGTGGATTCATCTATCATTTAATAAAAGTCTGGAATCCTGGATTACTAATTGGTGGAATACTAGGCAATCCGAAACCCTTTTGAATGATATTCAAGAAAAGACTATTTTAGAAAAATTCATAGAATTAGAGCAATTAGTCCGCTTGGACGAAATGATAAAGGAATTTCCGGAAAGACGTCTAGAAAAGCTTGGTATAGCGCTCCAGAAAGAAACAATTCAATTAATCAAGATGCACGATGAGGATCATATCCATACGATTTTTCACTTCTCGACAAATACAATCTGCTTCGTTATTCTAAGTGGTTATTCTATTCTGTGTAATGAAGAACTTTTTATTCTTAACTCTTGGGTTCAAGAATTCCTATATAATTTAAGCGACACACTAAAAGCCTTTTCGATTCTTTTCGTAACTGATTTATGTATCGGATTCCATTCGCCCCGCGGTTGGGAACTGCTGATTGGATATGTCTACAATGATTTTGGGTTTGCTCACAATGATAATGATATTATTCTATCTGTTCTTGTTTCCACTTTTCCAGTCGTTCTAGATACATTTTTTAAATATTGGCTTTTTTCTTATTTAAATCGTGTATCCCCGTCACTTGTAGTGATTTATCATTCAATGACTGAGTGA

>petA

ATGCAAATTAGAAATACCTTTTCTTCGTTAAAGGGAGAGATTACTCGATTCATTTCCGTATCCCTCATGATATATATAATAACTCGGGCATCCATTTCAAATGCATATCCCATTTTTGCGCAACAGGGTTTTGAAAATCCACGAGAAGCAACTGGTCGTATTGTATGCGCCAATTGCCATTTAGCTAATAAGCCTGTGGATATCGAGGTTCCACAGGCGGTCCTCCCTGATACTGTATTTGAAGCAGTTGTTAGAATTCCTTATGATATGCAACTGAAACAAGTTCTTGCTAATGGTAAAAAGGGGGCTTTGAATGTGGGGGCCGTTCTGATTTTACCAGAGGGGTTTGAATTAGCCCCCCCCGACCGTATTTCGCCCGAGATGAAAGAAAAGATAGGCAAGCTGTCTTTTCAGACCTATCGACCCACTAAAAAAAATATTCTTGTGATAGGGCCGGTTCCTGGGCAGAAATATAGTGAAATCACTTTTCCTATTCTTTCCCCGAACCCCGCGACTAATAAAGATGCTCACTTCTTAAAATACCCAATATATGTAGGCGGGAACAGGGGGAGGGGTCAGATTTATCCCGACGGGAACAAAAGTAACAATACGGTTTATAATGCTACAGCTATGGGTATAGTAAGCAAAATCATCCGAAAAGAAAAAGGGGGATACGAAATAACCATAACGGATGCCTCGAATGGACGTGAAGTGGTTGATATTATCCCCCCGGGACCAGAACTTCGTGTTTCCGAGGGCCAATCTATCAAACTTGATCAACCATTAACAAGTAATCCTAATGTAGGTGGGTTTGGTCAGGCAGATGCAGAAATAGTACTTCAAGATCCATTACGTGTCCAAGGCCTTTTGTTCTTTTTGGCATCTGTTGTTTTGGCACAAATCTTTTTGGTTCTGAAAAAGAAACAGTTTGAGAAGGTTCAATTGTCCGAAATGAATTTCTAG

>psbJ

ATGGCCGATACTACTGGAAGGATTCCCCTTTGGATAATAGGTACTGTAGCTGGTATTCCTGTGATCGGTTTAATAGGCATTTTCTTTTATGGTTCATATTCCGGATTAGGTTCGTCCTTGTAG

>psbL

ATGACACAATCAAACCCGAACGAACAAAACGTTGAATTGAACCGTACCAGTCTCTACTGGGGGTTATTACTCATTTTTGTACTTGCTGTTTTATTTTCGAATTACTTCTTCAATTAA

>psbF

ATGACCATAGATCGAACCTATCCAATTTTTACAGTGCGATGGTTGGCTGTTCACGGACTAGCTGTACCTACAGTTTCTTTTTTGGGGTCAATATCCGCAATGCAGTTCATCCAACGATAA

>psbE

ATGTCTGGAAGCACAGGAGAACGTTCTTTTGCTGATATTATTACCAGTATTCGATACTGGGTCATTCATAGCATTACTATACCTTCCCTATTCATTGCGGGTTGGTTATTCGTCAGCACGGGGTTAGCTTACGATGTGTTTGGAAGCCCTCGCCCAAACGAGTATTTTACAGAGAGCCGACAAGGCATTCCATTAATAACCGGCCGTTTTGATTCTTTGGAACAACTCAATGAATTTAGTAGATCTTTTTAG

>petG

ATGATTGAAGTCTTTCTATTTGGAATCGTCTTAGGTCTAATTCCTATTACTTTGGCTGGATTATTCGTAACCGCCTATTTGCAGTACAGACGTGGTGATCAGTTGGACCTTTGA

>psaJ

ATGCGCGATCTAAAAACATATCTTTCCGTGGCACCGGTACTAAGTACTCTATGGTTCGGGTCTTTAGCAGGGTTATTGATCGAAATCAACCGTTTATTCCCCGACGCATTGACATTTCCATTTTTTTCATTCTAG

>rpl33

ATGGCCAAGGGTAAAGATGTCCGAGTAAGGGTTATTTTGGAATGTACTAGCTGTGTTCGAAACGGTGTTAATAAGGAATCAAGGGGTATTTCCAGATATATTACTCAAAAGAATCGACACAATACACCTAGTCGATTGGAATTGAGAAAATTCTGTCCTTATTGTTACAAACATACACTTCATGGGGAGATAAAAAAATAG

>rps18

ATGGATAAAACCAAGCGACTCTTTCTTAAATCCAAGCGATCTTTTCGTAGGCGTTTGCCCCCGATCCAATCGGGGGATCGAATTGATTATAGAAACATGACTTTAATTAGTCGATTTCTTAGTGAACAAGGAAAAATATTATCTAGACGAGTGAATAGATTGACTTTAAAAGAACAACGATTAATTACTATTGCTATAAAACAAGCTCGTATTTTATCTTTGTTACCTTTTCTTAATAATGAGAAACAATTTGAAAGAAGTGGGTCGACCGCTCGAACGCCAGGTCTTCGAACCAGAAAAAAATAG

>rpl20

ATGACCAGAATTAGACGAGGATATATAGCTCGGAGACGTAGAACAAAAATGCGTTTATTTGCATCAAGCTTTCGCGGGGCTCATTCCAGACTTAGTCGAACAATTACTCAACAGAAAATAAGAGCTTTAGTTTCGGCTCATCGCGATAGAGATAGGAAAAAAAGGGATTTTCGCCGTTTGTGGATCACTCGAATAAATGCAGTAATTCGCGGAAACGGGGTATCCTATATTTATAGTAGATTAATATACAATCTGTATAAGGCACAGTTGGTTCTTAATCGTAAGATACTTGCACAAATAGCTATATCAAATAGGAATTGTCTGTATATGATTTCCAATGAGATCAGAAAATAA

>rps12

ATGCCAACTATTAAACAACTTATTAGAAACCCAAGACAGCCAATCAGAAACGTTACAAAATCCCCCGCTCTGCGAGGATGCCCTCAGCGCCGAGGAACATGTACAAGGGTGTATACTATCACCCCCAAAAAACCAAACTCTGCCTTACGTAAAGTTGCCAGAGTACGATTAACCTCTGGGTTTGAAATCACTGCTTATATACCCGGTATTGGCCATAATTCACAAGAACATTCTGTAGTCTTAGTAAGAGGGGGGAGGGTTAAGGATTTACCCGGTGTGAGATATCACATTGTTCGAGGAACCCTAGATGCTGTCGGAGTAAAGGATCGTCAACAAGGGCGTTCTAAATATGGGGTCAAAAAGCCAAAATAA

>clpP

ATGCCTATTGGTGTTCCAAAAGTACCTTATCGAAGTCCCGGGGACAAGCACCCATCTTGGGTTGACATAAATCGACTTTATCGAGAAAGATTACTTTTTTTAGGTCAAATGGTTGAGAGTGATATCTCGAATCAACTTATTGGTATTATGGTCTATCTCAGTATAGAAAATGAGACCAAGGATTTGTATTTATTTATCAACTCTCCTGGCGGGTGGGTAATACCCGGGATAGCTATTTATGATACTATGCAATTTGTGCGACCGGATGTACAGACAATATGCATGGGATTGGCCGCTTCCATGGGGTCTTTTCTCTTGGCCGCAGGAGCAAGTACCAAACGTCTAGCATTCCCTCACGCTAGGGTCATGATCCATCAACCGATTGGCGCTTTTTATGGGGCACAAACGGGAGAATTTATCCTGGATACGGAAGAACTGCTGAGACTGCGCGAAATCCTTACAATGGTTTATGTACAAAGGTCGGGCAAGCCCTTATGGGTTGTATCCGAAGACATGGAAAGGGATACTTTTATGTCAGCAACAGAAGCCCAAGCTCATGGAATTGTTGATCTTGTAGCGGTTGGATAA

>psbB

ATGGGTTTGCCTTGGTATCGTGTTCATACTGTCGTATTGAATGATCCCGGTCGTTTGATTTCTGTCCATATAATGCATACAGCCCTGGTTGCGGGTTGGGCCGGTTCCATGGCTCTATATGAATTAGCTGTTTTTGATCCCTCCGACCCGGTTCTTGATCCAATGTGGAGACAAGGCATGTTCGTTATACCCTTCATGACTCGTTTAGGAATAACCGATTCTTGGGGTGGTTGGAGTATTACAGGGGGGACGGTAGCGAATCCGGGTATTTGGAGTTACGAAGGTGTAGCCGGGGCACATATTGTGTTTTCCGGATTGTGCTTCTTGGCAGCTATCTGGCATTGGGTGTATTGGGATTTAGCAATATTTGTTGATGACCGTACAGGAAAACGTTCTTTGGATTTGCCTAAAATCTTTGGAATTCATTTATTTCTCTCAGGAGTGGCTTGCTTTGGTTTTGGGACATTTCATGTAACAGGATTGTATGGTCCTGGAATATGGGTGTCTGACCCCTATGGACTAACTGGAAAGGTACAATCTGTAAATCCAGCGTGGGGTGTGGAAGGTTTTGATCCTTTTGTTCCAGGAGGAATAGCCTCTCATCATATTGCTGCAGGGACATTGGGGATATTAGCAGGTTTATTCCATCTTAGTGTCCGCCCACCTCAACGCCTATACAAAGGATTACGTATGGGCAATATTGAAACCGTGCTTTCCAGCAGCATCGCTGCTGTCTTTTTTGCAGCCTTTGTTGTTGCTGGAACTATGTGGTATGGTTCAGCAACTACCCCTATCGAATTATTTGGTCCCACCCGTTATCAATGGGATCAGGGATACTTTCAGCAAGAAATATATCGAAGAGTCAGTGCTGGACTAACCGAAAATCAAAGTTTAGCAGAAGCTTGGTCGAAAATTCCTGAAAAATTAGCTTTTTATGATTACATCGGAAATAATCCTGCGAAAGGGGGATTATTCCGAGCGGGTTCAATGGATAATGGGGATGGAATAGCTGTCGGGTGGTTAGGACACCCTATCTTTAGAGATAAAGAAGGACGTGAACTTTTTGTACGTCGTATGCCTACTTTTTTTGAAACCTTTCCAGTTGTTTTGGTAGACGGAGATGGAATTGTTAGAGCCGACGTGCCTTTTCGAAGAGCAGAATCAAAGTATAGCGTCGAACAAGTAGGTGTAACTGTTGAGTTCTATGGTGGCGAACTGAATGGCGTGAGTTATAGCGATCCTGCTACTGTGAAAAAATATGCTAGACGTGCTCAATTGGGTGAAATTTTTGAATTAGATCGTGCTACTTTGAAATCTGATGGTGTTTTTCGTAGTAGTCCAAGGGGCTGGTTTACTTTTGGACACGCTTCATTTGCTCTGCTTTTCTTCTTCGGACACATTTGGCACGGTGCTAGAACCTTGTTCAGAGATGTTTTTGCTGGTATTGACCCGGATTTGGATGCTCAAGTGGAATTTGGAGTATTCCAAAAACTTGGAGATCCAACTACAAGAAGACAAGCAGTCTGA

>psbN

ATGGAAACAGCAACCCTAGTCGCCATCTCTATATCTGGGTTACTTGTAAGTTTTACTGGGTACGCCTTATATACTGCTTTTGGGCAACCCTCTCAACAACTAAGAGATCCATTCGAGGAACACGGAGACTAG

>psbH

ATGGCTGCAAAAACGGTTGAAGGTAGTTCTAGAGCTCGTCCAAAAAGCACTTCTGCAGGAGGTTTATTGAAACCCTTGAATTCGGAATATGGTAAAGTAGCTCCGGGATGGGGAACTACTCCTTTGATGGGTGTCGCAATGGCTCTATTTGCAGTATTCTTGTCGATTATTTTGGAGATTTATAATTCGTCCGTTTTACTGGACGGAATTTCAATGAATTAG

>petB

ATGAGTATGAAATTCTCCTATACGGTTCTCAGAGGGGAGTCCCCTTGGTTTACCTATCTCAATAAAGTCTATGATTGGTTCGAAGAACGTCTCGAGATTCAGGCGATTGCAGATGATATAACTAGTAAATACGTTCCTCCTCATGTCAACATATTTTATTGTCTAGGAGGAATTACGCTCACTTGTTTTTTAGTACAAGTAGCTACAGGGTTTGCTATGACTTTTTACTATCGTCCGACCGTTACTGAGGCTTTTGCTTCTGTTCAATACATAATGACGGAAGCTAACTTCGGGTGGTTAATTCGATCAGTTCATCGATGGTCGGCAAGTATGATGGTCCTAATGATAATCCTGCACGTATTTCGTGTGTATCTCACCGGCGGTTTTAAAAAGCCTCGCGAATTGACTTGGGTTACAGGTGTGGTTCTGGCTGTATTGACCGCATCTTTTGGTGTAACAGGTTATTCTTTACCTTGGGACCAAATTGGATATTGGGCAGTCAAAATTGTAACAGGCGTGCCAGAAGCAATTCCGGTAATAGGATCGCCTTTGGTAGAGTTATTACGTGGCAGTGCTAGTGTGGGACAGTCCACTTTGACTCGTTTTTATAGTTTACACACTTTTGTATTACCTCTTCTTACTGCCGTATTTATGTTAATGCATTTCCTAATGATACGTAAACAAGGTATTTCTGGCCCTCTATAA

>petD

ATGGGAGTAACAAAAAAACCTGACTTGAATGATCCTGTATTAAGGGCTAAATTGGCTAAGGGTATGGGCCATAATTATTATGGCGAACCCGCATGGCCCAACGACCTTTTATATATTTTTCCAGTAGTAATTCTAGGTACTATTGCATGTAACGTAGGCTTAGCGGTTCTAGAACCTTCAATGGTTGGTGAACCCGCGGATCCATTTGCAACTCCTTTGGAAATCTTACCCGAATGGTATTTTTTTCCCGTATTTCAAATACTTCGCACAGTACCCAATAAGCTGTTGGGTGTTCTTTTAATGGTTTCAGTACCTGCGGGATTATTAACAGTACCCTTTTTGGAAAATGTTAATAAATTCCAAAATCCATTTCGCCGCCCAGTCGCGACAACCGTCTTTTTGATTGGTACCGTGGTGGCCTTGGGCTTGGGTATTGGAGCAACATTACCTATTGAAAAATCCCTAACTTTAGGTCTTTTTTAA

>rpoA

ATGGTTCGAGAGAAAGTAAAAGTATCGACTCGGACACTACAGTGGAAGTGTGTTGAATCAAGAGTAGACAGTAAGCGTCTTTATTATGGGCGCTTTATTTTGTCTCCACTTATGAAAGGTCAAGCCGACACAATAGGCATTGCGATGCGAAGAGTTTTGCTTGGAGAAATAGAAGGAACATGTATTACACGCGCAAAATCTGAGAAAATCCCGCATGAATATTCTACTATAGCGGGGATTCAAGAATCGGTACATGAAATTTTAATGAATTTGAAAGAAATAGTATTGAGAAGTAATCTTTATGGAACTTGTGACGCGCTTATTTGTGCCAAAGGTCCGGGGTATGTAACTGCTCAAGACATCCTCTTGCCGCCTTCTGTAGAAATCGTTGATAATACGCAGCACATAGCTATCCTAACAGAACCCGTCGATTTGCGTATTGGATTACAAATCGAGAGGAATCGAGGATATAATATAAAAACACCAAATAACTTTCAAGACGGAAATCATTATTCTATAGATGCCGCATTCATGCCTGTTCGAAATGCGAATCATAGTATTCAGTCTTATGGGAACGGCAATGAAAAACAAGAGATCCTTTTTCTCGAAATATGGACAAACGGAAGTTTAACTCCTAAAGAAGCACTTCATGAAGCCGCCCGGAGTTTGATTGATTTATTTATTCCCTTTCTACAGGCAGCAGACGCAAACTTACATTTAGAGAACAATCAATACAAGGTTACTTTACCCTTTTTTACTTTTCATGATAGATTGGCTAAACTAACGAAAAAGAAAAAAGAAATTGCATTGAAATCCATTTTTATTGACCAATCAGAAATGTCTCCCAGGATCTATAATTGTCTCAAAAAGTACAATATACATACATTATTCGACCTTTTGAATAAGAGTCAAGAAGACCTTATGAAAATTGAACACTTTCGCATAGAAGATGTAAAGCAGATAATGGGTATTCTCGAAAAAAAATAG

>rps11

ATGGCAAAATCTACACCAAGAAGTGTTTCACGTAGGCCGGGACGGATTGGTTCACGTAAAAGTGGACGCCGAATACCAAAGGGCGTTATTCATGTTCAAGCAAGTTTCAACAACACCATTGTGACTGTTACAGATGTACGAGGGCGAGTAATTTCGTGGTCCTCGGCCGGGACTTGTGGATTCAGGGGAACGAGACGAGGTACGCCTTTTGCTGCTCAAACCGCAGCAGCAAATGTTATTCGCGCAGTAGCGGATCAAGGTATGCAACGAGCAGAAGTCATGATCAAAGGTCCTGGTCTCGGAAGAGATGCAGCATTACGAGCTATTCGTCGAAGCGGGATTCTTTTAAATTTCGTACGGGATGTAACCCCTATGCCACATAATGGTTGCAGACCCCCTAAAAAAAGACGGGTGTAG

>rpl36

ATGAAAATAAGGGCTTCAGTTCGTAAAATTTGTGAAAAATGTCGACTGATCCGCAGGAGGGGCCGGATTATAGTAATTTGTTCCAACCCAAGACATAAACAAAGACAAGGATAA

>rps8

ATGAGCAACGACACTATTGCTGACATAATAACGTCTATCCGAAATGCTGACATGAATAGAAAGGGAACAGTTCGAATAGCATCTACTAACATCACCGAAAACATTGTTAAAATACTTTTGCGAGAGGGTTTTATAGAAAACGTAAGGAAACTCTCAGAAAACAAAAAAGAGTTTTTTGTTTTAACCCTACGACATAGAAGGAATAGGAAAGGACCCTATAGACCCATTTTAAATTTAAAACGAATCAGTCGACCCGGTCTCCGAATCTATTTTAACTATCAACGAATTCCTAGAATTTTAGATGGGATGGGGATTGTAATTCTCTCTACTTCTCGGGGTATAATGACAGACCGAGCGGCTCGACTAGAAAGAATCGGCGGGGAAATTTTATGTTATATATGGTAA

>rpl14

ATGATTCAACCTCAAACCCATTTGAATGTAGCAGACAATAGCGGTGCTCGAGAATTGATGTGTATTCGAATCATAGGAGCCAGTAATCGTAGATATGCTAATATTGGAGACGTTATTGTTGCTGTGATCAAGGAAGCAGTACCAAATACGCCTCTAGAAAGATCAGAAGTGATCAGAGCAGTAATTGTACGTACTTGTAAAGAACTCAGACGCGATAACGGTATGATAATAAGGTATGATGACAATGCTGCAGTTGTCATTGATCAAGAGGGAAATCCAAAGGGAACTCGAGTTTTTGGTGCGATCGCCCGGGAATTGAGACAGTTGAATTTTACTAAAATAGTTTCATTAGCGCCGGAAGTATTATAA

>rpl16

ATGCTTAGTAACCCCAAAAGAACAAGATTCCGTAAACAACATAGAGGAAGAATGAAAGGAATATCTTATCGAGGTAATCATATTTCTTTCGGTCAATATGCTCTTCAAGCACTTGAACCCGCTTGGATTACATCTCGACAAATCGAAGCGGGACGCCGCGCAATGACACGAAATGTACGGCGCGGGGGAAAAATATGGGTACGTATATTTCCAGACAAACCTGTTACGGTCAGACCCACGGAAACACGTATGGGGTCCGGGAAAGGATCCCCCGAATATTGGGTAGCCGTCGTTAAACCGGGTCGAATACTTTATGAAATGAGTGGAGTAGCCGAAAATCTAGCTCGAAAGGCTATTTCAATAGCGGCATCTAAAATGCCTATAAGAACGCAATTCATTATTTCTGGATAG

>rps3

ATGGGACAAAAAATAAATCCACTTGGTTTCCGACTTGGTACAACCCAAAGCCATCATTCTCTTTGGTTTGCAAAACCAAAAAATTATTGCGAAGGTCTACAAGAGGATCAAAAACTACGAAACTTTATTAAGAATTATATACAAAAAAATATGAGAGTATCCCCCGGTGTTGAGGGAATTGCGCGTATCGAGATTCAAAAACGAATTGATCTAATTCAAGTCATAATCTATATAGGATTCCCAAAATTATTACTCGAAAATCGACCGCGAAGAATTGAAGAATTGCAATTGAATGTACAAAAAGAACTTAATTGTGTGAACCGAAAACTAAACATTGCTATTACAAGAATTGCAAATCCTTATGGAAACCCCAATATTCTTGCCGAATTTATAGCCGGCCAATTAAAAAATCGGGTTTCTTTTCGCAAAGCAATGAAAAAAGCTATTGAATTAACTGAACAGGCGGATACAAAAGGAATTCAAGTACAAATTGCAGGGCGTCTTGACGGAAAAGAAATTGCGCGCGCCGAATGGATCAGAGAAGGTAGAGTTCCTCTACAAACCATTGGAGCTCAAATTGATTATTGTTCCTATACAGTTCGAACAATCTATGGGGTATTAGGAATCAAGATTTGGATATTTGTAGACGAAGAAAAATAA

>rpl22

ATGATAAGGATAATAAAGAAGAAGGTAAGAGTATCTGCTTTAGGTCAACATATATGTATGTCTGCTCACAAAGCCCGAAGGGTAATTGATCAGATTCGTGGACGTTCTTACGAGGAAACTCTTATGATACTCGAACTCATGCCTTATCGAGCATGTTATCCCATTTTTAAATTGGTTTATTCTGCAGCAGCAAATGGGATTCACAATTTCGGCTTCAACGAAGGAAGTTTATTCATTGTTAAAGCGGAAGTAAACGAAGGACCTGCTGCGAAAAGAGTCAAACCTCGAGCTCGAGGGCGGAGTTATCTGATCAAAAGACCCACTTGCCATATAACTATTGTTTTAAGAGATATATCCTTAGATGAATATGAATATAGAGACTATCTCGACTGCTGA

>ndhF

ATGGAACATACATATCAATATTCCTGGATCATACCCTTAGTTCCACTTCCAGTCCCTGTGTTAATAGGGGTAGGACTTCTACTTTTTCCGACCGCAACAAAACATCTTCGCCGGATGTGGGCTTTTCTTAGTATTTTATTGTTAAGTATAGTTATGGTTTTTTCGACTGATTTAGCTATTAAGCAAATAGATGGAACGTCAATCTATCAATCCCTAAGATCTTGGACCATCAATAATGATTTTTCTTTCGAGTTCGGATACTTTATTGATCCACTTACTTCTATTATGTTAATATTAATCACTACTGTTGGAATTCTTGTTCTTATTTATAGTGACAATTATATGTCTCATGATCAAGGATATTTGAGATTTTTTGCTTATATGACTTTTTTCAATGCGGCAATGTTAGGATTAGTTACAAGTTCAAATTTCATACAAATTTATATTTTTTGGGAATTGGTTGGAATGTGCTCTTATCTATTAATAGGGTTTTGGTTCACACGACCTATTGCGGCAGGCGCTTGTCAAAAAGCATTTGTAACTAATCGTGTAGGGGATTTTGGATTATTATTAGGGATTTTAGGTCTTTATTGGATAACGGGTAGTTTCGAATTTAAGGATTTGTTCGAAATATTGAATAACTTGATTTATAATAATGAGGTTAATCTTTTATTTGTTACTTTGTGTGCATTTCTATTATTTGCCGGCCCTGTTGCTAAATCCGCGCAATTCCCTCTTCATGTATGGTTACCCGATGCCATGGAAGGGCCCACTCCGATTTCGGCTCTTATCCACGCCGCAACTATGGTAGCGGCGGGAATTTTTCTTGTAGCTCGCCTTCTTCCGCTTTTTCTAGTCATACCATACATAATGAATCTAATATCTTTGATAGGTATAATAACAGTATTTTTAGGAGCTACTTTAGCTCTTGCTCAACAAGATATTAAGAGAGGGTTAGCTTATTCTACAATGTCTCAATTGGGTTATATGATGTTAGCTCTAGGTATAGGTTCTTATCGAGCCGCTTTATTTCATTTGATTACGCATGCTTATTCCAAAGCCTTGTTGTTTTTAGGATCCGGATCCATTATTCATTCAATGGAAGCTATTGTTGGCTATTTTCCAGATAAAAGCCAGAATATGGTTCTGATGGGTGGGTTAAGGAAGCACGTGCCCATTACAAAAACCGCTTTTTTATTAGGTACCCTTTCCCTTTGTGGTATTCCGCCTCTCGCTTGTTTTTGGTCTAAGGATGAAATTCTTAATGATAGCTGCTTGTATTCGCCGATTTTCTCACTAATAGCTTTTTTCACAGCCGGATTAACCGCATTTTATATGTTTCGAATTTATTTACTTACTTTTGAGGGATCTTTCAACTTTTGCTTTCAAAATTACAGTGGCAAAACAAAAAACTCGTTCTATTCAATATCTCTATGGGGTCAAGAAGAATCAAAACCGATAAAAAGAAAAATTGAGTTAGTGGCTTTATTAACACTCAAGAATAATGAAAGGGCTTCTTTTTTTTCGAAGAAGACTCATCGAAGTGCTAGTAATATAAAAGCTACACCCTTTCTTACTATTTTTCCTTTTCGCGCTACCAAGACTTTTTTGTATCCTCACGAATCAGACAATACTATGTTATTTGGTATGCTTGTATTAGTCCTATTTCCTTTATTTGTTGGAGCTATAGGAATTCCTTTGAATCAAGAAGTTATCGAGTCGGATATTTTATCAAAATTGTTAACTCCCTCTATAAACCTTTTACATCAAAATTCGAATCAGTTTGTTGATTGGTATGAAGGTTTAAAAACTCCAATGCTTTCCGTTAGTATAACCTATTTGGCAATATTTCTAGCCTACTTTTTATATAAGCCCTTTTATTCACCTTTACACAATTGGAACATACTTAATTTATTTGCTAAAAGAGGACCTAAAAGAAGTCTGTGGGACAAAATACTCCATTTTCTCTATGATTGGTCATATAATCGTGCTTATATAGATGCTTTTTACACACGCTTTTTAACGGAAGGGATAAGGGGATTAGCAGAAATAATTCATTTGTTCGACAGACGAGTAATTGATGGAATTACAAATGGGGTTGGTATTACAAGTTTTTTTGTAGGGGAAGGTCTAAAATATTTAGGGGGAAGTCGCATCTCTTTTTATCTTGTATTATATTTATTTTCTCTATTAATATTTTTAATAAGTTACTCCTTTTTCTTTTTTGTCCAGTATTTATAG

>rpl32

ATGGCAGTTCCAAAAAAACGTACTTCTAGATTAAAAAAACGTATTCGTAAAAATATTTGGAAAAAAGGGGGGTATTGGGCAGCGTTGAAGGCTTTTTCGTTAGCGAAATCCCTTTCTACGGGGAATTCAAAAAGTTTTTTTTTTACAACAAATAAATAA

>ccsA

ATGATCTTTTTAACTTTAGAGCATATATTAACGCATATAGCCTGTTCGGTCGTTTCAATTGGAATTACAATTTATTTAATAACCTTATTAGGCGATGAAATCAGAGGACTATATGATTCATCCGAAAAGGGTATGCTAGCTACCTCTTTCTGTCTAACAGGATTATTAATCACCCGTTGGATTTATTCGAGACATTTCCCATTAAGTGATTTATATGAATCATTAATCTTTCTTTCATGGAGTTTCTCCATTATTCATAGGATTTTTGATTTAAAAAAAAATGAAAATCAGTTAAGCGCTATAACGGCACCAAGTGCTATTTTTTCCCAAGGCTTTGCGACTTCGGGTTTTTTAACCAAAATGCATCAATCCGGAATATTAGTACCCGCTCTCCAAGTCCAGTGGTTAATGATGCACGTCAGTATGATGGTATTGGGCTACGCAGCTCTTTTATGCGGATCATTATTATCCACGGCTCTTCTAGTCATTACATTTCGAAAAGTGATAAGGATTTTTTCGAAACGAAACTATTGTTTAAATGGAAATGAGTCATTTTGTTTCGGTGAAATCCAATACATGGACGGAAAAGGGAAGGTTTTCCTAAATAGTTTTTCCACTAGAAATTTTTACAGGTATCAAGTGCTTCAACAATTGGATCGGTGGAGTTATCGTATTATTAGTTTAGGATTTATCTTTTTAACCATGGGTATTCTTTCGGGAGCAGTATGGGCTAATGAGGCGTGGGGGTCTTATTGGAATTGGGATCCAAAAGAAACTTGGGCATTTATTACTTGGATGATTTTCGGGATTTATTTACATACTCGAACAAATAAAAATTTGGAAGGTGTAAATTCCGCAATTGTGGCTTCTACGGGCTTTCTTATAATTTGGATATGTTATTTCGGAGTCAATCTATTAGGAATAGGGTTACATAGTTATGGTTCATTTAATTAA

>psaC

ATGTCACACTCAGTAAAGATTTATGATACATGTATAGGGTGTACTCAATGTGTCCGAGCTTGCCCCACAGATGTATTAGAAATGATACCTTGGGACGGATGTAAAGCAAAGCAAATAGCTTCTGCTCCAAGAACAGAGGACTGTGTCGGTTGTAAGCGATGTGAATCTGCCTGTCCAACGGATTTCTTGAGTGTTCGGGTTTATTTATGGCATGAAACAACTCGAAGCATGGGTCTAGCTTATTGA

>ndhE

ATGATACTCGAGCATGCCCTTGTTTTGAGTGCCTATTTATTTTCTATTGGTATCTATGGATTGATTACGAGTCGAAATATGGTTCGGGCTCTGATGTGTCTTGAACTTATACTAAATGCAGTTAATCTAAATTTCGTAACATTCTCTGATTTTTTTGATAGTCGACAATTAAAAGGAGAGATTTTCTCCATTTTTGTTATCGCTATTGCAGCCGCGGAAGCTGCTATTGGATTAGCTATTGTTTCGTCAATTTATCGTAACAGAAAATCGACTCGTATCAATCAATCGACTTTGTTGAATAAGTAG

>ndhG

ATGGATTTGCCTGGACCAATACATGATTTTCTTTTAGTTTTTCTGGGATTGGGTCTTATATTAGGGGGTCTGGGCGTGGTATTATTTACCAACCCAATTTTTTCTGCCTTTTCCTTGGGATTGGTTCTTGTTTGTATCTCTTTATTCTATATTCTATCAAATTCCCAGTTTGTAGCTGCGGCGCAGCTTCTTATTTACGTGGGAGCTGTAAATGTTTTAATCATATTTGCTGTAATGTTCATGAATGGTTCAGACCATTCCAAAGATTTTCAGTTGAATCTTTGGACGATTGGTGATGGACTTACTTCCCTCGTTTGTACAAGTATTTTTTTTTCACTAATCACGGCTATTCTAGATACGTCGTGGTACGGGATTATTTGGACTACACGATCCAACCAGATTATCGAACAAGATTTGATAAGTAATAGTCAACAAATTGGAATTCATTTATCAACGGACTTTTTTCTTCCATTTGAACTCGTTTCAATAATTCTTTTAGTTGCTTTGATAGGTGCAATTGCCGCGGCTCGTCAGTAA

>ndhI

ATGTTCCCTATGGTAACTGGTTTCATGAATTATGGTCAACAAACAATACGAGCTGCAAGGTATATTGGTCAAAGTTTCATGATTACTTTATCCCAAGCAAATCGTTTACCTGTAACTATTCAATATCCTTATGAAAAATTAATCACATCGGAGCGTTTTCGCGGTCGAATCCATTTTGAATTTGATAAATGTATTGCTTGTGAAGTATGTGTTCGCGTATGCCCCATAGATTTGCCTGTTGTTGATTGGAAATTTGAAACAGATATTCGAAAGAAACGGTTGCTTAATTATAGTATTGATTTTGGAATTTGTATTTTTTGTGGTAACTGCGTTGAGTATTGTCCAACAAATTGTTTATCAATGACTGAAGAATATGAACTTGCTACTTACGACCGTCACGAATTGAATTATAATCAAATTTCTTTAGGTCGTTTACCAGTGTCAGTAATTGACGATTTTACAATTCGAACAGTCTTGAATTCGACTCAACGAAAAAATGACTAA

>ndhA

ATGCTAATTGATATAACAGAAGTACAAGAAATCAATTCTTTTTCCAGATTGGAATCCCTACAAGAGGTCTATGGGATCATATGGGTCCTTGCCCCTATTTTGACTCTTGTATTGACAATCACAATAGGTGTCCTCGTAATTGTGTGGTTAGAAAGAGAAATATCTGCAGGAATACAACAACGTATTGGACCTGAATACGCCAGCCCTTTGGGAATTCTTCAAGCTTTAGCAGATGGAACAAAACTACTTTTCAAAGAAAACCTTCTTCCATCGCGAGGAAATAGTAGTTTATTCAGTATTGGACCCTCTATAGCAGTCATAGCAATTCTACTAAGTTATTCAGTAATTCCATTTAGTTATAACCTTGTTTTAGCTGACCTCAATATTGGTATTTTTTTATGGATTGCCATTTCAAGTATTGCCCCTATTGGACTTCTTATGTCGGGATATGGATCAAATAATAAATATTCCTTTTTAGGTGGTTTGCGAGCTGCTGCTCAATCGATTAGTTATGAAATACCATTAACTTTATGTGTTTTATCAATATCTCTATTATCTAACAGTTCAAGTACAGTTGATATAGTTGGGGCACAATCAAAATATGGTTTGTGGGGGTGGAATTTGTGGCGTCAACCTATAGGGTTTATCGTTTTTCTAATTTCTTCCCTAGCGGAATGCGAGAGATTACCTTTTGATTTACCAGAAGCAGAAGAAGAATTAGTAGCCGGTTATCAAACCGAATATTCCGGAATTAAATTTGGTTTATTTTACGTTGCTTCCTATCTAAATCTATTAGTTTCCTCATTATTTGTAACCGTTCTTTACTTAGGGGGTTGGAATCTTTCCATTTCATCCATATTTGTTCCTGAGCTATTTGAAATAAATAAAGCGGATGGAATCTTTGGAACGACAATTGGTATCTTTATTACATTAGCTAAAACTTATTTGTTCTTGTTCATTCCTATTACAACCAGATGGACTTTACCGAGACTCAGAATGGACCAACTATTAAATCTTGGTTGGAAATTTCTTTTACCTATTTCTCTCGGTAATTTATTATTAACAACCTCTTCCCAACTCCTTTCGTTATAA

>ndhH

ATGAGTAGACTAGCTACAGAAAAAGAATTTATGATAGTCAATATGGGACCTCACCACCCATCAATGCACGGTGTTCTTCGGCTCATCCTTACTCTAGACGGTGAAGATGTTATTGACTGTGAACCAATATTGGGTTATTTACACAGGGGGATGGAAAAAATTGCGGAAAACCGAACAATTATACAATATCTCCCTTATGTAACCCGTTGGGATTATTTAGCTACTATGTTCACAGAAGCAATAACTATAAATGGGCCAGAGCTGTTGGGAAATATTCAAGTACCTAAAAGGGCCAGCTATATCAGAGTAATTATGTTGGAGTTGAGTCGTATAGCTTCCCATCTGTTATGGCTTGGCCCTTTTATGGCGGATATTGGTGCACAGACTCCTTTCTTCTATATTTTCCGAGAAAGAGAATTAGTATATGATCTGTTCGAAGCTGCCACCGGTATGAGAATGATGCATAATTATTTTCGTATCGGAGGAATAGCGGCTGATTTACCTCATGGTTGGATAGATAAATGTTTGGATTTCTGTGATTATTTTTTAACGGTAATTGCTGAATATCAAAAACTGATTACACGAAACCCTATTTTTTTAGAACGCGTTGAGGGAGTAGGCATTATTGGTGGAGAAGAAGCAATAAATTGGGGTTTATCAGGACCGATGCTACGAGCGTCTGGAATCGAATGGGATCTTCGTAAAGTTGATCATTATGAGTGTTATGACGAATTTGACTGGGAAGTCCAGTGGCAAAAAGAAGGGGATTCCTTAGCCCGTTATTTAGTCCGAATCGGTGAACTGACGGAATCCATAAAAATTATTCAACAGGCTTTAGAAGGAATTCCGGGCGGCCCCTATGAAAATTTAGAAATACGATGCTTTGATAGAGAAAACGATCCAGAATGGAATGATTTTGAAGATCGATTCATTAGTAAAAAGCCTTCTCCTACCTTTGAATTGACGAAACAAGAACTTTATGTGAGAGTAGAAGCCCCAAAGGGAGAATTGGGAATTTTTTTGATAGGAGATCAAGGCGGGTTTCCTTGGAGATGGAAAATTCGCCCCCCCGGTTTTATCAATTTGCAAATTCTTCCTCAGTTAGTTAAAAGTATGAAATTGGCTGATATTATGACGATATTAGGTAGTATAGATATCATTATGGGGGAAGTTGATCGTTGA

>rps15

ATGGTAAAAAATTCATTCATCTCAGTTAGGGTTCAAGAAAAACAAGAAGAAAACAGTGGATCGGTTGAATTTCAAGTATTTCGGTTCACCAATAAGATACGGAGACTTACTTCACATTTAGAATTGCACAGAAAAGATTATTTATCTCAAAGGGGTCTACGGAAAGTTTTGGAAAAACGCCAACGTCTACTAGCTTATTTGTCAAAGAAAAATAGAGTACGTTATAAAGAATTAATTAGTAAGTTGAATATTCGGGAGTCAAAAAATCGTTAA

>ycf1

ATGATTTTTCAATCTTTTATACTAGGTAATCTAGTATCCTTATGCATGAAGATAATCAATTCGGTCGTTGTGGTCGGACTCTATTATGGATTTATGACCACATTCTCCATAGGGCCCTCTTATCTCTTCCTTCTCCGAGCTCGGGTTATGGAAGAAGGAGAAGAAGGAACCAAGAAGAAGGTATCAGCAACAACAGGGTTTATTGCGGGACAGCTCATGATGTTCATATCGATCTATTATGCGCCTCTGCATCTAGCATTGGGTAGACCTCATACAATAACTGTCCTAGCTCTACCGTATCTTTTGTTTCATTTCTTCTGGAACAATCCCAAACACTTTTTTGATTATGGATCTACTACCAGAAATTCAATGCGTAATCTTAGCATTCAATGTGTATTCCTGAATAATCTCATTTTTCAATTATTCAACCATTTCATTTTACCAAGTTCAATGTTAGCCAGATTAGTCAACATTTATATGTTTCGATGCAACAACAAGATGCTATTTGTAACAAGTAGTTTTGTTGGTTGGTTAATTGGTCACATTTTATTCATGAAATGGGTTGGATTGGTATTAGTTTGGATACAGCAAAAGAATTCTATTAGGTCTAATGTACTTATTCGATTTAATAAGTACCTTGTGTCAGAATTGAGAAATTCTATGGCTCGAATCTTTAAGATTCTCTTATTTATTACCTGTATCTACTATTTAGGCAGAATACCCTCACCCATTTTTACTAAAAAACTGAAAGTGAAAGAAACCTCAGAAACGGAAGAAAGAGATGTAGAAATAGAAACAACTTTCGAAAGGGGGGGGACTAAACAAGAACAAGAGGTATCCGCCGAAGAAGATCCTTCTCCTTCCCTTTTTTCGGAAGAAAAGGAGGATCCGGACAAAATCGAGGAAACGGAAGAGATCCGAGTGAATGGAAAGGAAAAAAAAAAAACAAAGCATGAATTTCACTTTCGCTTTAAAGAGACATGCGATAAAAATAGCCCTGTTTATGAAACTTCGTATCTGGATGGGAATCCAGAAAATTCGCAATTGGAAATATTGCAAAACGAAGAGAAATCCTTATTAAGGTTTGAAAAACCTCTTGTAACTCTTCTTTTCGACTATAAATACTGGACTCGACCACTTCGCTATAAAAAAAATAATAGATTTGAAAATGCTGTTCGAAATGAAATGACAGAGTATTTTTTTTATACATGTCCAAGTGATGGAAAAGAAAGGATCTTTTTTACGTATCCTGCGAGTTTGTCAAGCTTTTTGGAAATGATAAAAAAAAAAAAGAATTTTTTTACCATAGAAAAACGGTTCTCTGATGAATTTTCCACACATTGGAATTACACTAATGACAAAAAAAAAAAGAGCTTGATCCGGGAGTTTAGAAATCGAATTGAAATTTTAGATAAGGGATCTCTTATTCAGGATATACTCGAAAAAAGGACTCAATTGTGTAATGATAATACTAAAACAAAATACTTACCTAAAATATATGATCCTTTATTACATGGACCTTATCGTAGAAGAAGACAAAAACTTGTTTTACCAGCAATACTAAATAGAACTTATATCCAAAATAAGATAGAAGCGCTTTGGATAAATAAAATTCACAATATAATTGTGATTAATGATTATCACGAATTTGAACAGACAATAGCCCTATTTAATCGAAAATCATTTTCAAGTGAAGAAGTGAAGTCTTTATTTACAGAACACGACCCAGAACAAATCGAGTTACAAGACCAAATAAAAATTTTTGATTTTTTATTAGATGCAGTTATAAGCGATCCTGATGATCAAAAAAGTAGAAAAAAATCGATAAAAGAAATTAGTAAAAGAGTTCCCCGGTGGTCATACAAATTAATCGATAATGTAGACCAAGAACTGGGAGAATACGACGAAAATGTAAGATGGGAACATCCATTTCGTTCACGAAAAGCAAAGCGTTTAGTGGTTTTTGTTGATCACGAGACAAACGAGGATGTTACTTTGCCAGAGTATTTGGAACAATCTGATTTTCGTCGATATATAATTAAAGGTTGCACGCGCGCACAAAGACGTAAAACCATTATTTTGAATCCTGTTCAAGCAAATGCCCATTCCCCTCTTTTTTTAGACAGAATAGCCAAACCCTTTTCTTTCTCTTTTGATATTTCCAAGCTGATGGAAGTACTGTTTCGCAATTGGCTGGGGAAAAAGACCGACCAAAAACTGTCTGATTCCACAAGTGAAAGAGTACGAAAATTGGATACAAAAGAAAAAAAGAAGCCCAAAGAAGAACGATACAAAAGACAAGAAATGGGACGTCTAAAACAAGCGGAAGGCTGGGATAAGCGTTTACTTACTCGCGTACTCCGCAGTTCTATGTTAGTAATGCAAGCAATTCTTCGAAAATATATTCTATTGCCGTCATTGATACTAGCTAAAAATTTGGTTCGCATCCTATTACTCCAAGACCCCGAGTGGTCCGAGGATTTTAAGGATTGGAATCATGAAATTTATGTTAAATGCACTTATAGTGGCGTTAATTTATCTGAAACAGAATTTCCGAAAAACTGGTTAACAGAAGGTATTCAGATAAAGATCCTATTCCCTTTTCACCTCAAACCCTGGCACAGATCTAAGATAGAAACCCCTCATAAGGATCCTCAAAATGAACAAGAAACCGATTTTTGCTTTTTAACAGTTTTGGGACTGGAAACAGAAATACCATTTGGGTCTCCCCGAAAACGACATTCTTTTTTTCAACCAATTTTGAAAGAACTAAAAAAAAAAATTTTCAAATGGCAAACTAAGTCTTTTATAGTTTTACGAGTTTTTAAAGAAGAAAAAATAGAAGAACTTTCCAAAATAAACTTGAGAGAAATCGCTAAATTGGTCGAAACTCAAAAAAATTCTCTAATCAGCAAGCAAATCAATCACGAATCGTCTATTGCAATTCCATCTACGAATTGGACAAATTTCTCCCGGACCGAAAAAAAAATGCAAGATCTGAGTACTAGAACAAGCGGAATCAGAAATAAAATAGATAAAATTAAAAAAGAAAAGAAAAAAAGATCACTAACTCAAGAGACAAATATTAGTTCGAACAAAACTACTTATTCGGCTAAAATATTCGACCCATCAAAAAAAATTTGGCAGATATTAAAAAAAAGAAATACTCGATTAATCCGGAAATCCTATTTTTTTATAAAATTTGTCATTGAAAAGATCTACAGAAATCTTTTTCTATCTACCCTTACTATTCCAAGAATCAATCCAAAATCTTTTCGGGAATCAACAAAAAGCCAAATTATAGCGAAAAACATACACCATAATGAAGCAAATCCGGAAATCTTTTTAATTAATAAAACAAATAAAAAGCGAAGTCACTTTTTTTCGACTATCAAAAAAGTACTTTTTAAGATTCGAAATCCGAATTCAAAGATTTCTTGTAATTTATCGTCGTTTTCACAATCACAAGCATATGTATTTTACAAATTGTTACAAGCCCCAATTTTGAACTTTTATAATTTAAGACCGATTCTTCAATATCACGACACACCTCGATTTCTTAAGAATGAAATAAAAAAGGGTTTTGAAAAACATGAAATATTTAATTACCAATTATACCAATTAAGACGGAAACCCTTTTTTAATTTGGGAAGGAATCAATGGCAAAATTGGTTAAGCGGGCATTATCAATATGATTTCTCGGGAATTAAATGGGCTAGATTAGTACAACAAGAATGGCGAAATAGAGCCAATCAACACTGTATGGCTCAAAATAAAGATTTAACTAAAAGTGATTCATATGAAAAAACCGGATTAACTCATTGCGAAAAACAACATTTTGTTGAAGTCGACTCATTACGTAGTCAAAAATCGAATTTTAAAAAACACTATAGATATGATCTTTTATCATATAAATCAATTAATTATGAAGATAAGAACGACTCTGATATTGATCGATCACTAGTTCAAGTAAATAAAAAAGAAGAGTATTATTCTAATTGCAATAGAAAGAAAGGCAAATTATTTGCTATGCTGGGAGGTATCTCTATCAATAATTATGGAGGGGAAGATGATATTCGGGAGATGGAAAAATTCTTGTATAGAAAATATTTTGATTGGAGAATTCTTAATTTTTGTCTTCGAAATAAGGTCAATATTGAAGCCTGGGTCGATATGAATATGGGTACCAGCAGTAATCAAAATACTAGGATTGGCTTCACTAATTATCCAAAATTTCATGCAATTAATAAAGGGCTCCCTTTTTATCTTACAATTCATCAAGATGAAGAAATTAACCCATCCAATCAAAAAAAAAACTTTTTTGATTGGATGGGAATGAATGAAGAAATCCTAAGTTGTCCTATATCAAACCCGGAGTCTTGGGTCTTCCCCGAATTGGGGCTACTTTTTAATGCATATAGAATGAAACCATGGATCATACCAAGCAAATTACTTTTTTTCAATTTCAATGGAAATCATAAGAAAAATCTAACCATAAAAAACGAAGCAAATTGTTTTCGAGTATCGACTCAACAAGAATCTCGTGAATTATCGAAGCCGAGTAAAGAAGAAAAGGAACTCGCAGACCAAGGAAATCCTAAATCCGATGCACACAAGCAAGGAAGTCTTGAGTTAGCTATCTCAAACCAAGAAAAAGATGTTGACGAAAATTATACGAAATCGGGCCTGAAAAACCGTATAAAGAACAAGCAATACAAGAGAGAAACCGAAGCGCAGCTCGATTTCTTCCTAAAAAAATATTTGTGTTTGCAGTTGAGATGGAGAGGTGCTGTTTCTTTCAGGGAAAAAATACTCAATGAGATGAAAGTATATTGTCATCTGGTTCGACTGATAAATCCCAGCGATGTTGCTATAGCCTCTATTCAAGGGGGAGAAATTAGTCTGCCTATGTTGATCACTAAGAAGGATTTCGCTCTTACAGAAGTGACGAAAGGTGGAATGCTTATTATCGAACCCCGCCGTTTGTCTGTAAAAAATGATGGACAATTTTTTCTATATCAAATCGTAGGTATTTCATTAGTTCATAAGAATAGGCGCACAATTACTAAAAGATACCAAGAAAGGGGCTATGGTGATCAAAAAATTTTTGATGAATCCATTGCAAAACATCAAAAAATGACTGGAAATCGAAACAAAAATCATTATGATTTGCCTGTTCCTGAAACTATTTTATTTCCTAAACGTCGTAGAGAATTAAGAACTCTAATTTGTTTCAATTCGAAGAAGCGAAATGGTATGCAGCGAAATCCAGTATTTTTTAATAACGTAAAAAGCATCGGTCGCGTTTTGGATAAAACAAAAAATCTTTCTATAGAGAAAAATCAACTAATTAAATTAAAGTTTTTTATTTGGCCCAATTCTCGATTAGAAGATTTAATTTGTATGAATCGCTATTGGTTTAATACCAATAATGGGAGTCGTTTCAGTATGGTAAGGATATATATGTATCCACGAGTTCAAATTCGTTAA

>rps7

ATGTCACGTCGAGGTACTACAGAAGAAAAAACTGCAAAATCCGATCCAATTTATCGTAATCGATTAGTTAACATGTTGGTTAACCGTATTCTGAAACACGGAAAAAAATCATTGGCTTATCAAATTATCTATCGAGCCTTGAAAAAGATTCAACAAAAGACAGAAAAAAATCCACTATCTGTTTTACGTCAAGCAATACGTGGAGTAACTCCCGATATAGCAGTAAAAGCAAGACGTGTAGGCGGATCGACTCATCAAGTTCCCATTGAAATAGGATCCGCACAAGGAAAAGCACTTGCCGTTCGTTGGTTATTAGGGGCATCCCGAAAACGTCCGGGTCGAAATATGGCTTTCAAATTAAGTTCCGAATTAGTGGATGCTGCCAAAGGGAGTGGCGATGCCATACGCAAAAAGGAAGAGACTCATAGAATGGCAGAGGCAAATAGAGCTTTTGCACATTTTCGTTAA

>ndhB

ATGATCTGGCATGTACAGAATGAAAACTTCATTCTCGATTCTACGAGAATTTTTATGAAAGCCTTTCATTTGCTTCTCTTCGATGGAAGTTTTATTTTCCCAGAATGTATCCTAATTTTTGGCCTAATTCTTCTTCTGATGATCGATTCAACCTCTGATCAAAAAGATATACCTTGGTTATATTTCATCTCTTCAACAAGTTTAGTAATGAGCATAACGGCCCTATTGTTCCGATGGAGAGAAGAACCTATGATTAGCTTTTCGGGAAATTTCCAAACGAACAATTTCAACGAAATCTTTCAATTTCTTATTTTACTATGTTCAACTCTATGTATTCCTCTATCCGTGGAGTACATTGAATGTACAGAAATGGCTATAACAGAGTTTCTGTTATTCGTATTAACAGCTACTCTAGGAGGAATGTTTTTATGCGGTGCTAACGATTTAATAACTATCTTTGTAGCTCCAGAATGTTTCAGTTTATGCTCCTACCTATTATCTGGATATACCAAGAAAGACGTACGGTCTAATGAGGCTACTATGAAATATTTACTCATGGGTGGGGCAAGCTCTTCTATTCTGGTTCATGGTTTCTCTTGGCTATATGGTTCATCCGGGGGCGAGATCGAGCTTCAAGAAATAGTGAATGGTCTTATCAATACACAAATGTATAACTCCCCGGGAATTTCAATTGCGCTTATATTCATCACTGTAGGAATTGGGTTCAAGCTTTCCCTAGCCCCTTCTCATCAATGGACTCCTGACGTATACGAAGGATCTCCCACTCCAGTCGTTGCTTTTCTTTCTGTTACTTCGAAAGTAGCTGCTTCAGCTTCAGCCACTCGAATTTTCGATATTCCTTTTTATTTCTCATCAAACGAATGGCATCTTCTTCTGGAAATCCTAGCTATTCTTAGCATGATATTGGGGAATCTCATTGCTATTACTCAAACAAGCATGAAACGTATGCTTGCATATTCGTCCATAGGTCAAATCGGATATGTAATTATTGGAATAATTGTTGGAGACTCAAATGGTGGATATGCGAGCATGATAACTTATATGCTGTTCTATATCTCCATGAATCTAGGAACTTTTGCTTGCATTGTATTATTTGGTCTACGTACCGGAACTGATAACATTCGAGATTATGCAGGATTATACACAAAAGATCCTTTTTTGGCTCTCTCTTTAGCTCTATGTCTCTTATCCCTAGGAGGTCTTCCTCCACTAGCAGGTTTTTTCGGAAAACTCCATTTATTCTGGTGTGGATGGCAGGCAGGCCTATATTTCTTGGTTTCAATAGGACTCCTTACGAGCGTTGTTTCTATCTACTATTATCTAAAAATAATCAAGTTATTAATGACTGGACGAAAGCAAGAAATAACCCCTCACGTACGAAATTATAGAGGATCCCCTTTAAGATCAAACAATTCCATCGAATTGAGTATGATTGTATGTGTGATAGCATCTACTATACCAGGAATATCAATGAACCCGATTATTGCAATTGCTCAGGATACCCTTTTTTAG

>ycf15

ATGCTACTGCTGAAACATAGAAGAATTGAAATCTTAGATCAAAACACTATGTATGGCTGGTATGAACTGCTTAAACAAGAATTCTTGAACAGCGAACCACCAGAGCTATTACTAACTACATCAAAAAATTTCCATTAA

>ycf2

ATGAAAGGACATCAATTCAAATCCTGGATTTTCGAATTGAGAGAGATATTGAGAGAGATCAAGAATTCTCACTATTTCTTAGATTCATGGACCCAATTCAATTCAGTGGGATCTTTCATTCACATTTTTTTCCACCAAGAACGTTTTATAAAACTCTTGGACCCACGAATTTGGAGTATCCTACTTTCACGCAATTCACAGGGTTCAACAAGCAATCGATATTTCACGATCAAGGGTGTAGTACTATTTGTAGTAGCGATCCTTATATATCGTATTAACAATCGAAATATGGTCGAAAGAAAAAATCTCTATTTGACAGGGCTTCTTCCTATACCTATGAATTCCATTGGACCCAGAACTGATACATTGGAAGAATCTTTTGGGTCTTCCAATATCAATAGGTTGATTGTTTCGCTCCTGTATCTTCCAAAAGGAAAAAAGATCTCTGAGAGCTTTTTCCTGGATCCGAAAGAGAGTACTTGGGTTCTCCCAATAACTAAAAAGTGTATCATGCCTGAATCTAACTGGGGTTCGCGGTGGTGGAGGAACTGGCTCGGAAAAAAGAAGGATTCTAGTTGTAAGATATCTAATGAAACCGTCGCTGGAATTGAGATCTCATTCAAAGAGAAAGATATCAAATATCTGGAGTTTCTTTTTGTATATTATATGGATGATCCGATCCGCAAGGACCATGATTGGGAATTGTTTGATTGTCTTTCTCCGAGTAAGAGGCGAAACATAATCAACTTGAATTCGGGACATCTATTCGAAATCTTAGTGAAAGACTGGATTTGTTATCTCATGTTTGCTTTTCGTGAAAAAATACCAATTGAAGTGGAGGGTTTCTTCAAACAACAAGGAGCTGGGTCAACTATTCAATCAAATGATATTGAGCGTTTTTCCCATCTCTTCTTGAGAAACAAGTGGGCTATTTCTTTGCAAAATTGTGCTCAATTTCATATGTGGCAATTCCACCAAGATCTCTTCGTTAGTTGGGGGAAGAATCCCCACGAATCGGATTTTTTGAGGAAGATATCGAGAGAGAATTGGATTTGGTTAGACAATGGGTGGTTGGTAAACAAGGATCGATTTTTTAGCAAGGTACGGAATGTATCGTCAAATATTCAATATGATTCCACAAGATCTAGTTTCGTTCAAGTAACGGATTCTAGCCAATTGAAAGGATCTTCTTCTTCTGATCAATCCAGAGATCATTTCGATTCCATTAGTAATGAGGATTCGGAATATCCCACATTGATCAATCAAAGAGAGATTCAACAACTAAAAGAAAGATCGATTCTTTGGGATCCTTCCTTTCTTCAAACGGAACGAAGAGAGATAGAATCAGGCCGATTCCCTAAATGTCTTTCTGGATATTCCTCAATGTCCCGGCTATTCACGGAACGTGAAAGGCAGATGAATAAGCATCTGCTTCCGGAAGAAATCGAAGAATTTCTTGGGAATCCTGCAAGATCCATTCGTTCTTTTTTCTCTGACAGATGGTCAGAACTTCATCTGGGTTCGAATCCTACTGAGAGGTCCACTAGAGATCAGAAATTGTTGAAGAAAGAAGAGGATGTTTCTTTTGTCCCTTCCAGGCGATCGGAAAATAAAGAAATAGTTAATATATTCAAGATAATTACGTATTTACAAAATACCGTCTCAATTCATCCTATTTCATCAGATCCGGGATGTGATATGGTTCCGAAGGATGAACTGGATATGGGCAGTTCCAATAAGATTTCATTCTTAAACAAAAATCCATTTTTTGATTTCTTTCATCTATTCCATGACCGGAACAGGGGGGGATACACGTTACACCACGATTTTGAATCAGAAGAGAGATTTCAAGAAATGGCGGATCTATTCACTCTATCAATAACCGAGCCGGATCTGGTGTATCATAAGGGATTTTCCTTTTCTATTTTTTCCTACGGATTGGATCAAAAACAATTCTTGAATGAGGTATTCAACTCCGGGAATGAATCGAAAAAGAAATCTTTATTGGTTCTACCTCCTATTTTTTATGAAGAGAATGAATCTTTTTATCGAAGGATCAGAAAAAAATGGGTCCGGATCTCCTGCGGGAATGATTGGGAAGATCCAAAACCAAAAATAGTGGTATTTGCTAGCAACAACATAATGGAGGCAGTCAATCAATCTAGATTGATCCGAAATCGGATTAAAATCCAATATAGCGCCTATGGGTACATAAGAAAGGGATTGAATCGATTCTTTTTAATGAATAGATCCGATCGCAACTTCGAATATGGAATTCACAGGGATCAAATAGGAAATGATACTCTGAATCATAGAACTATAATGAAATATACGATCAACCAACATTTATCGAATTTGAAAAAGAGTCAGAAGAAATGGTTCGCTCCTCTAATTTTTCTTTCTCGAACCGAGAGATTCATGAATCGGGATCCTAATGCATATAGATACAAATGGTCCAATGGGAGCAAGAATTTCCAGGAGCATTTGAACCATTTCGTTTCTGAGCAGAAGAGCCGTTTTCAAGTAGTGTTCGATCGATTACGTATTAATCAATATTCGATTGATTGGTCTGAAGTTATCGACAAAAAAGATTTGTCTAAGTCACTTCCTTTCTTTTTGTCCAAGTTGCTTTTATTTTTGTCTAACTCACTTCCTTTTTTCTTTGTGAGTTTCGGGAATATCCCCATTCATAGGTCTGAGATCCACATCTATGAATTGAAAGGTCCGAATGATCAACTCTGCAATCAGTTGTTAGAATCAATAGGTCTTCAAATCGTTCATTTGAAAAAATTGAAACCCTTCTTATTGGATGATCATAATACTTCCCAAAAATCGAAATTCTTGATCAATGGAGGAAGAATATCACCATTTTTGTTCAATAAGATACCAAAGTGGATGATTGACTCATTCCATACTAGAAATAGTCGCAGGAAATCTTTTGATAACACGGATTCCTATTTCTCAATGATATCCCACGATCAAGACAATTGGCTGAATCCTGTGAAACCATTTCATAGAAGTTCATTGATATCTTCTTTTTATAAAGCAAATCGACTTCGATTCTTGAATAATCCACACCGCTTCCGCTTCTATTGTACCAAAAGATTCACTTTTTGTGTGGAAAAGGTCCGTATCAATAATTATGATTTTACGTATGGACAATTCCTCAATATCTTGTTCATTCGCAACAAAATATTTTCTTTATGCGGCGGTAAAAAAAAGCATGCTTTTTTGGAGAGAGATACTATTTCACCAATCGAGTCACAGGTATCTAACATATTCATACCTAACGATTTTCCACAAAGCGGTGACGAAAGGTATAACTTGTACAAATTTTTCCCTTTTCCAATTCGATCCGATCTATTAGTTCGTAGAGCTATTTACTCGATCGCAGCCATTTCTGGAACACCTCTATCAGAGGGACAAATAGTCAATTTTGAAAGAACTTATTGTCAACCTCTTTCAGATATGAATCGATCTGATTCAGACGAGAAGAACTTGCATCAGTATCTCAATTTCAATTCAAACATGGGTTTGATTCACACTCCATGTTCTGAGAAATATTTACCATCCGAAAAGAGGAAAAAACAGAGTCTTTTTTGTCTAAAGAAATGCGTTGAGAAAGGGCAGATGTCTAGAACCTTTCAACGAGACAGTGCTTTTTCAACTCTCTCAAAATGGAATCGATTCCAAACATATATGCCATGGTTCCTTACTTCGACAGGGTACAAATATCTAAATTTGATATTTTTAGATACTTTTTCAGACCTATTGCCGGTACTAAGTAGCAGTCAAAAATTTGTATCCATTTTTCATGATATTATGCATGGAGCAGATAGATCATGGCGAATTCTTCAGAAAAAATGGTGGCTTCCACAATGGAATCTGATAAGTGAGATTTCGAGTAAGTGTTTCCATAATCTTCTTCTGTCCGAAGAAATGATTCATCGAAATAATGAGTCACCATTGATATCGACACATCGGAGATCGCCAAATGCTCGGGAGTTCCTCTATTCAATCCTTTTCCTTCTTCTTGTTGCTGGATATCTCGTTCATACACATCTTCTCTTTGTTTCCCGAGCCTATAGTGAGTTAGAGACAGAGTTCGAAAGGGTCAAATCTTTGATGATTCCATCATACATGATTGAGTTGCGAAAACTTCTGGATAGGTATCCCACATCTGAACTGAACTCTTTCTGGTTAAAGAATCTCTTTCTAGTTGCTCTGGAACAATTAGGAGATTCTCTAGAAGAAATACGGGATTCTGCTTTTGGCGGCAACATGCTATGGGGTGGTGGTCCCGCGGATGGGGTTAAATCAATACGTTCTAAGACGAAAGATTTGAATATCAATCTCGTCGATATCATCGATCTCATACGTATCATACCAAATCCCATCAATCGAATCACTTTTTCGAGAAATACGAGACATCTAAGTAATACAAGTAAAGAGATCTATTCATTGATAAGAAAAAGAAAAAAGGGGAACGGTGATTGGATTGATGATAAAATAGAATCCTGGGTCGCGAACAGTGATTCGATTGATGATAAAGAAAGAGAATTCTTGGTTCAGTTCTCCACCTTAACCTTAACGACAGAAAAAAGGATTGATCAAATTCTATTGAGTCTGACTCATAGTGATCATTTATCAAAGAATGACTCTGGTTATCAAATGATTGAACAACCGGGAACAATTTACTTACGATACTTAGTCGACATTCATAAAAAGCATTTCATGAATTATGAGTTCAATACATACTGTTTAGCAGAAAGACGGATATTCCTTGCTCATTATCAGACAATCACTTATTCACAAACTTCGTGTGGGGCTAATAGTTTTCATTTCCCATCTCATGGAAAACCCTTTTCGCTCCGCCTAGCCCTATCCCCCTCGAGGGGTATTTTAGTGCTAGGTTCTATAGGAACCGGACGCTCCTATTTGGTCAAATACCTAGCGACAAACTCCTATGTTCCTTTCATTACAGTATTTCTGAACAAGTTCTTGGATAACAAGCGTAAAGGTTTTCTTATTGATGATATCGATGATGATAGTGACGATATTGATGCTAGTGACGATATCGATCGTGACCTTGATACGGAGCTGGAGCTTCTAACTATGATGAATGCGCTAACTATCGATATGATGTCGGAAATAGGCCGATTTTATATTACCCTTCAATTCGAATTAGCAAAAGCAATGTCTCCTTGCATAATATGGATTCCAAACATTCATGATCTGGATGTGAATGAGTCGAATTACTTATCCCTCGGTCTATTAGTGAACTATCTCTCCAGGGATTGTGAAAGATGTTCCACTAGAAATATTCTTGTTATTGCTTCGACTCATATTCCCCAAAAAGTGGATCCCGCTCTAATAGCCCCGAATAAATTAAATACATGCATTAAGATACGAAGGCTTCTTATTCCACAACAACGAAAGCACTTTTTCACTCTTTCATATACTAGGGGATTTCACTTGGAAAAGAAAATGTTCCATACTAATGGATTCGGATCCATAACCATGGGTTCCAATGCACGAGATCTTGTAGCACTTACCAATGAGGCCCTATCGATTAGTATTACACAGAAGAAATCAATTATAGACACTAATACAATTAGATCTGCTCTTCATAGACAAACTTGGGATTTGCGATCCCAGGTAAGATCGGTTCAGGATCATGGGATCCTTTTCTATCAAATAGGAAGGGCTGTTGCACAAAATGTACTTCTAAGTAATTGCCCCATAGATCCTATATCTATCTATATGAAGAAGAAATCATGTAACGAAGGGGATTCTTATTTGTACAAATGGTACTTCGAACTTGGAACGAGCATGAATAAATTAACGATACTTCTTTATCTTTTGAGTTGTTCTGCCGGATCGGTCGCTCAAGACCTTTGGTCTCTACCCGGACCCGATGAAAAAAACGAGATCACTTCTTCTGGACTCGTTGAGAATGATTCTGATCTAGTCCATGGCCTATTAGAAGTAGAAGGCGCTCTGGTGGGATCCTCGCGGACAGAAAAAGATTGCAGTCGGTTTGATAATGATCGGGTGACATTGCTTCTTCGGCCCGAACCAAGGAATCCCTTAGATATGATGCAAAAAGGATCTTGTTCTATTGTTGATCAGAGATTTATCTATGAAAAATACGAATCGGAGTTTGAAGAGGGGGAAGGAGTCCTCGACCCGCAACAGATAGAGGAGGATTTATTCAATCACATAGTCTGGGCTCCTAGAATATGGCGCCCCTGGGGCTTTCTATTTGATTGTATCGAAAGGCCCAATGAATTGGGATTTCCCTATCGGGCCGGGGCATTTCGGGGCAAGCGGATCATTTATGATGAAAAGGATGAGCTTCAAGAGAATGATTCGGAGTTCTTGCAGAGTAGAACCATGCAGTACCAGACACGAGATAGATCTTCCAACGAACAAGGCTTTTTTCGAATAAGCCAATTCATTTGGGAACCTGCGGATCCACTCTTTTTCCTATTCAAAGATCAGCCCCTTGTCTCTGTGTTTTCACACCGAGAATTCTTTGCAGATGAAGAGATGTCAAAGGGGCTTCTTACTTCCCAACCAGATCCTCCTACATCTATATATAAACGCTGGTTTATCAAGAATACGCAAGAAAAGCACTTCGAATTGTTGATTCATCGCCAGAGATGGCTTAGAACCAATAGTTCATTATCTAATGGATTTTTCCGTTCTAATACTCCATCCGAGAGTTATCAGTATTTATCAAATCTGTTCCTATCTAACGGAACGCTATTGGATCAAATGACAAAGACATTGTTGAGAAAAAGGTGGCTTTTTCCGGATGAAATGAAAATTGGATTCATGTAA

>rpl23

ATGGATGGAATCAAATATGCAGTATTTACAGACAAAAGTATTCGGTTATTGGGGAAAAATCAATATACTTCTAATGTCGAATCAGGATCAACTAGGACAGAAATAAAGCATTGGTTCGAACTCTTCTTTGGTGTCAAGGTAATAGCTATGAATAGTCATCAACTCCCCCGAAAGGGTAGAAGAATGGGACCTATTATGGCACATACAATGCATTACAGACGTATGATCATTACGCTTCAACCGGGTTATTCTATTCCACCTCTTAGAAAGAAAAGAACTTAA

>rpl2

ATGGCGATACATTTATACAAAACTTCTACCCCGAGCACACGCAATGGAGCCGTAGACAGTCGAGTGAAATCCAATCCACGAAATAATTTGATCTATGGACAGCATCGTTGTGGTAAAGGTCGTAATGCCAGAGGAATCATTACCGCAGGGCATAGAGGGGGAGGTCATAAGCGTCTATACCGTAAAATAGATTTTCGACGGAATGAAAAAGACATATATGGTAGAATCGTAACCATAGAATACGACCCTAATCGAAATGCATACATTTGTCTCATACACTATGGGGATGGTGAGAAGAGATATATTTTACATCCCAGAGGGGCTATAATTGGAGATACCATTGTTTCTGGTACAGAAGTTCCTATAAAAATGGGAAATGCCCTACCTTTGACCGATATGCCCTTAGGCACGGCCATACATAACATAGAAATAACACTTGGAAAGGGTGGACAATTAGCTAGAGCAGCGGGTGCTGTAGCGAAACTGATTGCAAAAGAGGGGAAATCGGCCACATTAAAATTACCTTCTGGGGAGGTCCGTTTGATATCCAAAAACTGCTCAGCAACAGTCGGACAAGTGGGGAATGTTGGGGTGAACCAGAAAAGTTTGGGTAGAGCCGGATCTAAATGTTGGCTAGGTAAGCGTCCTGTAGTAAGAGGAGTAGTTATGAACCCTGTAGACCACCCCCACGGGGGTGGTGAAGGAAGGGCTCCAATTGGTAGAAAAAGGCCCGCAACCCCTTGGGGTTATCCTGCACTTGGAAGAAGAAGTAGAAAAAGGAATAAATATAGTGATAATTTGATTCTTCGCCGCCGTACTAAATAG

***T. asiatica***

>psbA

ATGACTGCAATTTTAGAGAGACGCGAAAACGGACGCCTATGGGGTCGTTTCTGTAACTGGATAACCAGCACCGAAAACCGCCTTTACATTGGATGGTTTGGTGTTTTGATGATCCCTACTTTATTGACCGCAACTTCTGTATTTATTATTGCCTTCATTGCTGCTCCTCCAGTCGATATTGATGGTATTCGTGAACCTGTTTCTGGATCTCTACTTTACGGAAACAATATTATTTCTGGTGCGATTATTCCTACTTCTGCAGCTATAGGTTTGCACTTTTACCCGATATGGGAAGCGGCATCTGTTGATGAATGGTTATACAATGGCGGTCCTTATGAGCTAATTGTTCTACATTTCTTACTTGGTGTAGCTTGTTACATGGGTCGTGAGTGGGAACTTAGTTTCCGTCTGGGTATGCGCCCTTGGATTGCTGTTGCATATTCAGCTCCTGTTGCAGCAGCGACTGCTGTTTTCTTGATCTACCCAATTGGTCAAGGAAGTTTTTCTGATGGTATGCCTCTAGGAATCTCTGGTACTTTCAACTTCATGATTGTATTCCAGGCTGAGCACAACATCCTTATGCACCCATTCCACATGTTAGGCGTAGCTGGTGTATTCGGCGGCTCCCTATTCAGTGCTATGCATGGTTCCTTGGTAACCTCTAGTTTGATCAGGGAAACCACAGAAAATGAATCCGCTAATGAAGGTTACAGATTCGGTCAAGAGGAAGAAACTTATAATATCGTAGCTGCTCACGGTTATTTTGGCCGATTGATCTTCCAATATGCTAGTTTCAACAATTCTCGTTCTTTACATTTCTTCCTAGCTGCTTGGCCTGTAGTAGGTATCTGGTTTACTGCTTTAGGTATTAGCACTATGGCTTTCAATCTAAATGGTTTCAACTTCAACCAATCTGTAGTTGATAGTCAAGGTCGTGTAATTAATACCTGGGCTGATATTATTAATCGTGCTAACCTTGGTATGGAAGTTATGCATGAACGTAATGCTCATAACTTCCCTCTAGACCTAGCTGCTATTGAAGCTCCATCTACAAATGGATAA

>matK

ATGGAGGAATTTCAAGTATATTTAGAACTAGATAGATCTCAACAACACGACTTCCTATACCCACTTCTTTTTCGGGAGTATATTTATGCACTTGCTTATGATCATGGTTTAAATAGCTCGATGATTTCATTGGAAAGTGGGGGTTATGACAATAAATCTAGTTCACTAAGTGTGAAACGGTTAATTACTCGAATGTCTCAACAGATTAATTTGAGTATTGCTGCGAATGACTCTAACCAAAATCCAATTTTTGGGCACAACAATAAGTTGCATTCTCAAATTATATCAGAGGGATTTGCTGTCGTGGTGGAAATTCCATTTGCCCCACGGTTAGTAGCTTTTTTAGAAGGGAAAGAATTTGAAAACTCTCAAAGTTTCCAATCAATTCATTCAATATTTCCTTTTTTCGAGGACAAATTGTCACATTTAAATTATGTGTTAGATGTACTAATACCCCACCCCATTTGTCCCGAAATCTTGGTTCAACCCCTTCGCTACTGGGTAAAGGATGCCTCTTCTTTCCATTTATTACGGTTCTTTCTCCACGAGTATTTTAATTCGAATAGTCTTATTACTACAAAGAACTCGATTTCTGTTTTTTTAAAAAGGAATCCAAGATTGTTATTGTTTCTATATAATTCTCATGTATATGAATATGAATCCATCCTCTTTTTTCTCTGTAACCAATCGTCTCATTTACGATCAACATCCTCTCGAGTCCTCGTTGAACGAATGTATTTCTATGGAAAAGTCGAAGATCTTGTCGAAGTCTTTGCTAAAGATTTTCAGGACATCTTATGCTTGTTCAAGGATCCTTTCATGCATTATGTTAGATATCAAGGAAAATCCATTCTGGCTTCAAAGGATACGCCTCTTCTGATGAATAAATGGAAATATTACCTTGTCGGTTTATGGCAATGGCATTTTCACGTGTCGTCTCAACCAGGAAGGGTTCATCTAAACCACTTAGGCAAGTACTCTATCAACTTTCTGGGCTATCTTTCCGGTGTGCGACTCAATTCTTTGGTGGTACGGAGTCAAATGCTAGAAAATTCATTTCTAATAGGTAATTCTATGAAGAAGGTCGATACGACCGTTCCAATTATTTATCTGATTGGATCATTGACGAAGGCGCGGTTTTGTAACGCATTAGGGCATCCCATCAGTAAGGCGGCCTGGGCCGATTTCTCTGATTCTCATCTTATCGACCGATTTGTGCGTATATGCAGAAATCTTTCTCATTATCACAGCGGATCCGCAAAAAAAAAAAGTTTGTATCGAATAAAATATATACTTCGGCTTTCTTGTGTTAAAAGTTTGGTTCGTAAACATAAAAGTACTGTACGCACCTTTTTGAAAAGGTTAGGTTCGGAATTATTGGAAGAATTCCTTACGGAGGAAGAACACGTTCTTTCTTTAATCTTTCCAAGAGCTTCGTCTACTTCGCGTAGGTTTGATTTATATAGAGGGCGGATTTGGTATTTGGATATTATTTGTATCAATGATCTGGTTAATTATGAATGA

>chlB

ATGAAATTTCTTTTGTTCCGCGCCAAGAATTCAAACTTGGTTTTGTGTCAATTGAACAAGAATAAAATATTCTCAAAATTATCCATTGATACGACATGCTGTTTTTTCCATTCATTCCTTTCAGGATCAGTCGTGGTCTTACAAACTCTCCCGAAGATTTGGACGAATCCCTTGCTTCATAGAAATGTGTAA

>rps16

ATGGTAAAACTTCGTTTGAAACGATGTGGTAGAAAGCAACGAGCCGTTTATCGAATCGTTGCAATTGATGGTCGATCCCGAAGAGAAGGAAGAGATCTTCAGAAAGTGGGTTTTTATGATCCGATAAATAATCAAACCCATTTAAATGTTCCCGCTATTCTATATTTCCTTGTCAAGGGCGCCCAACCTACAGGAACCGTTCATGATATTTCAAAGAAAGCGGGGGTTTTTACAGAACTTAGTCTTAATCAAATTTAA

>psbK

ATGCTTAATATCTTTAGTTTAATGTATATCTGTCTTAATTCTGCCCTTTATTCGAGTAGTTTTTTATTCGCCAAATTGCCCGAGGCCTACGCTTTTTTGAATCCAATTGTAGATGTTATACCAGTAATACCTGTTCTATTTTTGCTCTTAGCCTTTGTTTGGCAAGCTGCTGTAAGTTTTCGATGA

>psbI

ATGCTTACTCTCAAACTCTTTGTTTACACCGTAGTGATATTCTTTGTTTCTCTCTTCATCTTCGGATTCCTGTCTAATGATCCAGGGCGTAATCCCGGACGCGAAGAATAA

>atpA

ATGGCAACAATTAAAGCCGACGAAATTAGTAATATTATCCGTGAACGTATTGAGCAATATAATAGAGAAGTAAAGATTGTAAATATTGGTACCGTACTTCAAGTAGGCGACGGCATCGCCCGTATTTATGGTCTTGATGAAGTAATGGCAGGGGAATTAGTAGAATTTGAAGAGGGTACAATAGGCATTGCTCTTAATTTGGAATCAAATAATGTTGGTGTTGTTTTAATGGGTGACGGTTTACTGATACAAGAGGGAAGTTCTGTAAAAGCAACCGGCAAAATTGCTCAGATACCAGTAAGCGAGGCTTATTTAGGTCGTGTTATAAATGCCCTGGCTAAACCTATTGATGGTCGAGGTGAAATTTCAGCTTCTGAATCTCGATTAATCGAATCTCCCGCTCCGGGTATTATTTCGAGACGTTCCGTATATGAGCCTCTTCAAACAGGACTTATTGCTATTGATTCGATGATCCCTATAGGACGCGGGCAGCGAGAATTAATTATTGGGGACCGACAGACCGGTAAAACTGCAGTAGCCACGGATACGATTCTCAACCAACAAGGGCAAAATGTAATATGCGTTTATGTAGCTATTGGGCAAAAAGCATCTTCTGTGGCTCAGGTAGTGGGTACTTTCCAGGAAAGGGGGGCAATGGAATACACTATTGTGGTAGCCGAAACGGCGGATTCCCCCGCTACGTTACAATACCTCGCTCCTTATACGGGCGCAGCTCTGGCTGAATATTTTATGTACCGTGAACGACACACTTTAATCATTTATGATGATCCCTCCAAACAAGCGCAGGCTTATCGACAAATGTCTCTTCTATTACGAAGACCACCCGGTCGCGAAGCTTATCCCGGAGATGTTTTTTATTTGCATTCACGGCTTTTGGAAAGAGCCGCGAAATTAGGTTCGCAGTTAGGTGAAGGAAGTATGACCGCTTTACCAATAGTGGAGACCCAGTCAGGAGATGTTTCGGCTTATATTCCTACTAATGTAATTTCCATTACAGACGGGCAAATATTCTTATCCGCTGATCTATTCAATGCTGGAATCAGACCCGCGATTAATGTGGGTATTTCTGTCTCTAGAGTAGGATCCGCAGCTCAAATTAAAGCTATGAAACAAGTAGCCGGCAAATTAAAATTGGAATTGGCCCAATTCGCGGAATTAGAAGCCTTTGCACAATTTGCTTCTGATCTCGATAAAGCTACTCAGAACCAATTGGCAAGGGGTCAACGCTTACGTGAGTTGCTCAAACAATCCCAATCAGCCCCTCTCACGGTCGAAGAACAGATAATGACTATTTATACAGGAACGAATGGTTATCTTGATTCATTAGAAATTGGCCAAGTAAGAAAATTTCTCGTTGAGTTACGTACTTACTTAAAAACGAATAAACCTCAGTTCCAAGAAATAATATCTTCTACCAAGATATTCACCGAGGAAGCAGAAGCTCTTTTGCAAGAAGCTATTCAGGAACAAATGGAACGCTTTCTACTTCAGGAACAATTATAA

>atpF

ATGAAAAATTTAACCGATTCTTTCGTTTCTTTGGTTCACTGGCCATTCGCCGGGAGTTTCGGGTTTAATACCGATATTTTAGCAACAAATCCAATAAATCTAAGTGTAGTGCTTGGTGTATTGATCTTTTTTGGAAAGGGAGTGTTAAGTGATTTATTAGATAATCGCAAACTGAGGATCTTGAATAGTATTCGAAATTCAGAAGAACTGCAGGGCGGGGCCGTTGAACGGCTGGAAAAAGCCCGGGCCCGGTTACGGAAAGTCGAAATAGAAGCAGATCAGTTTCGAGTGAACGGATACTCTGAGATAGAACGAGAAAAATTCAATTTGATTAATTCAACTTCTAAGACTTTGGACCAATTAGAAAATTACAAAAATGAAACCATTCATTTTGAACAACAAAGAGCAATTAATCAAGTACGACAACGGGTTTTCCAACAAGCTTTACAAGGAGCGCTCGGAACTCTGAATAGTTGTTTGAACATGAACAAGGAGTTACATTTACGTACCATTAGTGCCAATATTGGCATGTTTGGGGCGATGAAAGAAATAACTGATTAG

>atpH

ATGAATCCACTGATTTCTGCCGCTTCCGTTATTGCCGCTGGGTTGGCTGTTGGGCTTGCTTCTATTGGACCTGGAGTTGGTCAAGGTACTGCTGCGGGCCAAGCAGTAGAGGGGATTGCGAGACAACCCGAGGCGGAGGGAAAAATACGAGGTACTTTATTGCTTAGTCTGGCTTTTATGGAAGCTTTAACAATTTATGGACTGGTTGTAGCATTAGCGCTTTTATTTGCGAATCCTTTTGTTTAA

>atpI

ATGAATGTTCTATCATGTTCCATCAACACACTAAAGGGGTTATACGATATATCCGGTGTGGAAGTAGGCCAACATTTCTATTGGCAAATAGGCGGGTTCCAAGTCCATGCCCAAGTACTTATTACTTCTTGGGTTGTAATTGCTATCTTATTAGGTTCAGCCTTTATAGCCGTTCGGAATCCACAAACCGTTCCGACTGCCAGTCAAAATTTCTTCGAATATGTCCTTGAATTCATTCGAGACGTGAGCAAAACTCAGATTGGAGAAGAATACGGCCCATGGGTTCCCTTTATTGGAACTATGTTTCTTTTTATTTTTGTTTCGAATTGGTCTGGTGCTCTTTTACCTTGGAAAATCATAGAGTTACCTCATGGGGAGTTAGCCGCACCTACGAATGATATAAATACTACCGTTGCTTTAGCTTTGCTCACGTCAGTAGCATACTTCTATGCGGGTCTTTCCAAAAAGGGATTAGGTTATTTCAGTAAATACATTCAACCGACTCCAATTCTGTTACCCATTAACATTTTAGAAGATTTCACAAAACCTTTATCACTTAGTTTTCGACTTTTCGGCAATATATTAGCCGATGAATTAGTAGTTGTTGTTCTTGTTTCTTTAGTCCCTTTAGTGGTTCCTATACCTGTCATGTTCCTTGGATTATTTACAAGCGGTATTCAAGCTCTTATTTTTGCAACTTTAGCTGCGGCTTATATAGGCGAATCTATGGAGGGACATCATTGA

>rps2

ATGGCAAGAAGATATTGGAACATCCATTTGGAAGAAATGATGGAAGCAGGAATCCATTTTGGTCATGGTACTCGGAAATGGAATCCTAGAATGGCACCTTATATATCTGCAAAACACAAAGGTATTCATATTACAAATCTGACTCGAACTGCTCGTTTTTTATCAGAAGCTTGTGATTTAGTTTTTGATGCAGCAAGTAGGGGAAAACAATTCTTAATTGTTGGTACTAAAAATAAAGCAGCTGATTCAGTCGCGCGAGCTGCAATAAGGGCTCGGTGTCATTATGTTAATAAAAAATGGCTCGGTGGTATGTTAACGAATTGGTCCACTACAGAAACAAGACTTCACAAGTTCAGGGATTTGAGAACGGAACAAAAAAAGGGGAGACTCGACAGTCTTCCCAAAAGGGATGCCGCTATTTTGAAGAGACAATTATCACGCCTGCAAACGTATCTGGGCGGGATTAAATATATTACGAGGGTACCCGATATTGTAATCATCGTCGATCAGCACGAAGAATATACGGCTCTTCGAGAATGTATCACTTTGGGAATTCCAACAATTTGTTTAATCGATACAAATTGTGACCCCGATCTCGCAGATATTTCGATTCCAGCAAACGATGACGCTATAGCCTCAATCCGATTAATTCTTAACAAATTAGTATTCGCAATTTGTGAGGGTCGCTCTAGCTATATACGAAATCGTTGA

>rpoC2

ATGGCAGAACGGGCGAGTCTGGTCTTTCACAATAAAATGATAGATGGAACTGCCATTAAACGACTTATTAGCAGGTTAATAGATCACTTCGGAATGGCATATACATCACACATCCTGGATCAAGTAAAGACCCTGGGTTTCCAGCAAGCCACTGCTACATCTATTTCATTAGGCATTGATGATCTTTTAACGATACCTTCTAAGCGATGGCTAGTCCAAGATGCTGAACAACAAAGTTTTATTTTGGAAAAACACCATCATTATGGGAATGTACACGCGATAGAAAAACTACGTCAATCCATTGAGATATGGTATGCTACAAGTGAATATTTGCGACAAGAAATGAATCCTAATTTTAGGATGACCGATCCCTTTAATCCAGCCCATATAATGTCTTTTTCGGGAGCTAGAGGAAATGCATCTCAAGTACACCAATTGGTGGGTATGAGAGGATTAATGTCTGATCCCCAAGGTCAAATGATTGATTTACCCATTCAAAGCAATTTACGCGAAGGACTTTCTTTAACAGAATATATAATTTCTTGCTATGGAGCCCGCAAGGGAGTTGTAGATACCGCTGTACGAACATCAGATGCTGGATATCTTACGCGCAGACTTGTTGAAGTAGTTCAACACATTGTTGTACGTAGAACAGATTGTGGCACCATCCGAGGAATTTCTGTGAGTCCTCAAAATCAAAATAGGATGCTGTCGGAAAGGGTTTTTAGCCAAACATTAATTGGTCGTGTATTAGCAGACGATATATATATGGGTCCGCGATGCATCGCCATTAGAAATCAAGATATTGGGATTGGACTTGTCAATCGACTCATAACCTTTCGAGCACAAGCAATATCTATTCGAACCCCCTTTACTTGTAGGAGTACATCTTGGATCTGTCGATTATGCTATGGTCGGAGTCCGACTCATGGTGACCTGGTTGAATTGGGGGAAGCCGTAGGTATTATTTCGGGTCAATCTATTGGAGAACCGGGGACTCAACTAACATTAAGGACTTTTCATACCGGTGGCGTATTTACAGGGGGCACTGCAGAACATGTACGAGCCCCTTCTAATGGTAAAATAAAATTCAACGAGGATTTGGTTCATCCCACGCGCACACGTCACGGGCATCCTGCTTTTCTATGTTCGATAGATTTGGATATAATTATTGAGAGTGAAGATATTATGCATAATGTGACTATTCCACCAAAAAGTTTTCTTTTAGTTCAAAACGATCAATATGTCGAATCAGAACAAGTGATTGCTGAGATTCAGGCGGGAGCATACACTTTGAATTTTAAAGAGAGGGTTCGAAAACATATCTATTCTGATTCAGAGGGAGAAATGCACTGGAGTACTGATGTGTACCATGCACCCGAATTTACATATAGTAATGTACACCTCTTGCCAAAAACAAGTCATTTATGGATATTATCGGGGAGTTCATGCAGATCTAGTGTAGTTTCTTTTTCACTCTACAAGGATCAAGATCAAATGAATATTCATTCTCTTTCTGTCGAACGAAGAGAGATTTCTAGCCTCTCGCTCTCGGTGAATAATGATCAAGCGAGACACAAATTATTTAGTTCTGATTTTTCTGCTAAAAAAGAAGGTGGAATTCTTGAGATATCTGATTATTCGGGATTTAAGAGAATCATAGGTACTGGTCATTGTAATCTCATACATCCTGCAATTCTCCACGCGAATTCGGATTTATTGGCAAAAAGGCAAAGAAATCGATTTCTTATTCCATTCCACTCGATTCAAGAACAAGAGAAAGAGCTAATGCCCCATTCAGGGATCTCGATTGAAATACCCATAGGGGGTATTTTCCGTAGAAATAGTATTCTTGCTTATTTCGACGATCCTCGATACAGAAGAAAGAGTTCCGGAATTACTAAATATGGGACTCTGGGGGCGCATTCAATCGTCAAAAAAGAGGACTTGATTGAGTATCGAGGACTCAAAAAAATTAAGCCAAAATACCAAATGCAAATAGATCGCCTTTTTTTCATTCCCGAGGAAGTGCATATTTTTCCCGAATCTTCTTACCTAATGGTACGGAATAATAGTATCATTGGAGTAGATACACGAATCACTTTAAATATAAGAAGCCGAGTGGGCGGATTGGTGCGAATGGAGAGAAAAAAAGGGGGGATTGAACTAAAAATATTTTCGGGAGATATCCATTTTCCCGGAGAGATAGATAAGATATCCCGACACAGTGGCATCTTGATACCGCCAGAAAGGGAAAAAAAAAAACTTAAGGAATCCACTAAGGAATCAAAAAAATTGAAAAAATGGATCTATGTTCAACGGATCACACCTACCAAGAAAAAGTATTTTGTTTTGGTTCGACCCGTAGTCACATATGAAATAGCGGACGGTATAAATTTAGCAACACTCTTCCCCCAGGATCCGCTGCGGGAAAAGGATAATATGAAATTTCGAGTTGTCAATTATGTCCTTTATGGGAAGGGCAAAGCTGCTCGGGGAATTTCTGATACAAGTATTCAATTAGTTCGGACGTGTTTTGTGTTGAATTGGGACCAAGACAAAAAAAGTTCTTCCGTCGAAGAGGTTTGTGCTTCCTTTGTTGAAGTACGTACAAATGGTCTGATTCGCGATTTCTTAAGAATCAACTTAGTGAAATCCCAAATTTCATATATCAGAAAAAGGAATCATCCGTCAGGTTCAGGATTGATCTCTGATAATGGTTCCGTTCGCACCAATAGCAATCCGTTTTATTCCGTTTTTGGCAAGGCGGGGGTTGAACAATCACTTAGCCAAAATCAAGGAACTATTCGTACGTTGTTGAATAGAAATAAGGAATGCCAATCTTTGAGAATTTTGTCATCATCTAATTATTTTCGAATGGGTCCATTGAACGATGTAAAATATCACAATGTGATAAAACAATCAATTCCAACTCAAAAAGATTCTCTAACCCCAATTAGGAATTCGTTGGGACCTTTAGGAACAGTCCTTCAAATTGCGAATTTTTATTCATTTTACTATTTAATAACTCATAATCATCTCTCGGTAACTAAATATTTGAAACTTGACAATTTAAAACAGCCTTGTCAAATACTTAAATATTATTTAATGGATGAAAACGGGGAAATTTCGAATCCTGATACAGACAGTAAGATCATTTTGAATCCATTTAATTTGAATTGGTATTTTCTCCATCATAATTATTGTAAGGAAATGTCCCCGAGAATTAGTCTTGGGCAGTTTCTTTGTGAAAATGTATGTATAACCAAAAACGGACCACACCTAAAATCTGGTCAAGTTTTAATTGTTCAAGTCAACTCTGTAGTAATACGATCAGCTAAGCCTTATTTGGCTACTCCTGGAGCAACTGTTCATGGGCATTATGGAGAAATCCTTTACGAAGGGGATACATTAGTTACATTTATATATGAAAAATCGAGATCTGGTGATATAACGCAGGGTCTTCCAAAAGTAGAACAAGTGTTAGAAGTGCGTTCGCTTGATTCAATATCGATGAACCTAGAAAAGAGAGTTGAGGGTTGGAACGCGCGTATAACAAGAATTCTTGGGATTCCCTGGGGATTCTTGATTGGTGCTGAGCTAACTATAGTGCAAAGTCGTATCTCTTTGGTTAATAAGATCCAAAAGGTTTATCGATCGCAGGGGGTGCAGATCCATAATAGGCATCTAGAAATTATTGTACGTCAAATAACATCAAAAGTCTTGGTTTCAGAAGATGGAATGTCTAATATTTTTTTACCCGGCGAACTGATTGGATTGTTACGAGCGGAACGAACGGGGCGCGCTTTGGAAGAAGTGATCTGTTATCGAGCTATCTTATTGGGAATAACGAGAGCATCTCTGAATACTCAAAGTTTTATATCCGAAGCAAGTTTTCAAGAAACCACGCGAGTTTTAGCAAAAGCAGCTCTCCGAGGTCGTATCGATTGGTTGAAAGGCTTGAAGGAAAACGTTGTTCTGGGGGGGATAATACCCGTTGGTACCGGATTCAAAGGATTAGTGCACTGTTCAAGGCAGCATAACACCATTCTTTTGGAAAGACAAAAAGGGAATTTATTCGGGGGGGAAATGAGAGATATTTTCTTACACCACAGAGAATTATTTGACTCTTGCATTTCAACGACTTTCCATGATACATCAGAGCAATTGCTTAGAGGGTTTAATGAGTCCTAG

>rpoC1

ATGAATCAAAATTTTTCTTCTATGATTGATCGATATAAACATCAACAACTCCGAATTGGATCAGTTTCTCCTCAACAAATAAGCGCTTGGGCCAATAAAATCCTACCTAATGGAGAGATAGTTGGAGAAGTGACAAAACCCTATACTTTTCATTACAAAACCAATAAACCAGAAAAAGATGGATTATTTTGTGAAAGAATTTTTGGGCCTATTAAAAGTGGAATTTGCGCTTGTGGAAATTATCGAATAATTGGAGATGAAAAGGAAGACCCGCATTTTTGTGAACAATGTGGAGTTGAATTTGTTGATTCTCGGATACGAAGATATCAAATGGGATACATAAAACTGGGCTGCCCAGTAACCCACGTGTGGTATTTGAAACGTCTTCCTAGTTATATCGCGAATCTTTTAGATAAACCTCTTAAAGAATTAGAAGGCCTAGTATATTGCGATTTTTCTTTTGCTAGGCCTATAGGGAAAAAACCGACTTTTTTACGATTACGAGGTTCATTCGAATATGAAATACAATCCTGGAAATACAGCATCCCGCTTTTTTTTACTACCCAAGGTTTCGATAAATTTCGTAACAGAGAAATTTCTACGGGAGCAGTTGCTATCCGGGAACAATTAGCCGATCTAGATTTGCGAATTATTCTAGATTATTCCTTGTTAGAATGGAAAGAATTAGGGGAAGAAGGACCCGCGGGTAATGAATGGGAAGATCAAAAAGTTGGACGAAGAAGGGATTTTTTGGTTAGACGCATGGAATTAGCTAAGCATTTTCTTCGAACAAATATAGAACCTGAGTGGATGGTTTTATGTCTATTACCTGTTCTTCCTCCCGAGTTGAGACCGATCATTCAGATAGATGGAGGTAAACTAATGAGTTCAGATATTAATGAACTCTATAGAAGAGTTATCTATCGGAACAATACTCTTACCGATCTATTAACAACAAGTAGATCTACGCCAGGGGAATTAGTAATGTGTCAGGAGAAATTGGTACAAGAAGCCGTGGATACGCTTCTTGATAATGGAATCAGTGGACAGCCAATGAGGGATGGTCATAATAAGGTTTATAAGTCGTTTTCAGATGTAATTGAAGGCAAAGAAGGAAGATTTCGTGAGACTATGCTTGGCAAACGGGTTGATTATTCGGGGCGTTCTGTCATTGTTGTAGGCCCCTCACTTTCATTACATCGATGTGGATTGCCTCGCGAAATCGCAATAGAGCTTTTCCAGAGTTTTGTAATTTGTGGGCTAATTAGACAACATCTTGCTTCGAACATAGGAGTTGCTAAGAGTAAAATTCGGGAAAAAGGGCCGATTGTATGGGAAATACTGCAGGAAGTTATGCAGGGACATCCAGTATTGCTGAATAGAGCGCCTACTCTGCATAGATTGGGCATACAGGCATTCCAGCCCATTTTAGTGGAAGGGCGCGCTATTTGTTTACATCCATTAGTTTGTAAGGGATTCAATGCAGACTTTGATGGGGATCAAATGGCTGTTCATGTACCTTTATCTTTAGAGGCTCAAGCGGAGGCTCGTTTACTTATGTTTTCTCATATGAATCTCTTGTCTCCTACTATTGGAGATCCCATTTCCGTACCGACTCAAGATATGCTTATTGGACTCTATGTATTAACGAGCGGGAATCGTCGAGGTATTTGTGCAAATAGGTATCATCCATGTAATCGAAGAAATTATCAAGATGAAAGAATTGACGATAATAGCTATAAGTATACGAAAGAACCCTTTTTTTGTAATTCCTATGATGCAATTGGGGCTTATCGGCAGAAAAGAATCAATTTAGATAGTCCTTTGTGGCTCCGGTGGCGATTAGATCAACGCGTTATTGCTTCAAGGGAAGCTCCCATCGAAGTTCACTATGAATCTTTGGGTACCTATCATGAGATTTATGGACATTATCTAATAGTACGAAGTGTAAAAAAAGAAATTCTTTCTATATACATTCGAACCACCGTTGGCCATATTTCTCTTTATCGAGAAATCGAAGAAGCTATACAAGGGTTTTGTCGGGCCTGCTCATATGGTACCTAA

>rpoB

ATGCTCGGAGATGGAAATGCGGGAATGTCTACAATACCTGGGTCGAATCAGATACAATTTGAAGGCTTTTGTAGGTTCATTGATCAGGGCTTAACAGAAGAACTTTATAAGTTTCCAAAAATTGAAGATACGGATCAAGAAATTGAATTTCAATTATTTGTGGAAACATATCAATTGGTAGAACCCTTGCTAAAAGAAAGAGATGCTGTATATGAATCATTCACATATTCTTCTGAATTATATGTATCCGCAGGATTAATTTGGAAAAGCCGAGGGGATATGCAGGAACAAACAATTTTTATTGGAAACATTCCTCTAATGAATTCTTTGGGAACTTCTATAGTAAATGGAATATACCGAATTGTCATCAATCAAATATTGCAAAGTCCCGGTATCTATTATCGGTCAGAATTGGGCCATAATGGAATGTCGGTCTATACAGGCACCATAATATCCGATTGGGGAGGACGGTTAGAATTAGAGATTGATAGAAAAGCAAGGATATGGGCTCGTGTGAGTAGGAAACAGAAAGTATCTATTCTAGTTCTATCAGCAGCTATGGGTTCGAATCTACGAGAAATTCTAGAGAATATTTGCTACCCTGAAATTTTCTTGTCTTTCCTGACCAATAAGGAGAAAAAAAAAATTGGATCAAAAGAAAGTGCCATTTTGGAGTTTTATCAACAATTTGCTTGTGTAGGCGGAGATCCGGTATTTTCGGAATCCTTATGTAAGGAATTACAAAAGAAATTTTTTCACCAAAGATGTGAATTAGGAAAGATTGGTAGACGAAATATGAACCAGAGACTGAATCTTAATATACCTCAGAACAATACCTTTTTGTTACCACGAGATGTATTGGCAGCTGTCGATCATTTGATTGGACTGAAATTTGGAATGGGTACACTTGACGATATGAATCATTTGAAAAATAAGCGTATTCGGTCTGTAGCGAATCTTTTACAAGATCAATTCGGATTGGCCCTGGTTCGGTTAGAAAATGTGATTAGAGGAACTATATGCGGGGCAATTAGGCATAAATTGATGCCAACTCCTCAAAATTTGGTAGCTTCAACTCCATTAACAACCACTTATGATTCTTTTTTCGGATTACATCCATTATCTCAAGTTTTGGATCGAACTAATCCATTGACACAAATAGTTCATGGGAGAAAATTGAGTTATTTGGGCCCCGGAGGATTGACAGGACGGACTGCTAGTTTTCGGATACGAGATATCCATCCTAGTCACTATGGCCGCATTTGCCCAATTGACACGTCTGAAGGAATCAATGTTGGACTTATTGGATCCTTAGCAATTCATGCGAGAATTGGTTATTGGGGGTCTCTAGAAAGCCCATTTTATGAAATCTTTGAAAAATCAAAAAAAGTACGGATGCTTTATTTATCACCAAGTAGAGATGAATACTATATGGTAGCGACAGGAAATTCTTTGGCACTGAATCAGGGTAGTCAGGAAGAGCAGGTTGTTCCGACTCGATACCGTCAAGAATTCCTGACTATTGCGTGGGAACAGGTTCATCTTCGAAGTATTTTTCCCTTCCAATATTTTTCTATTGGGGCTTCCCTCATTCCTTTTATCGAGCATAATGATGCGAATCGGGCTTTAATGAGTTCGAATATGCAACGCCAAGCAGTTCCGCTCTTTCGGTCCGAGAAGTGCATTGTTGGAACTGGGTTGGAACGCCAAGTGGCTCTAGATTCCGGGGTTCCCGCTATAGCCGAACACGAGGGAAAGATCATTTATACCGATATTGACAAGATCATTTTATCAGGCAACGGGAATACTTATCGTATTCCGTTAGTTCTGTATCAACGTTCAAACAAAAATACTTGTATGCATCAAAAAACCCAGGTTGGGCGGGGTAAATGCATTAAAAAGGGCCAAGTTTTAGCGGATGGTACCGCTACAGTTGGTGGCGAACTCGCTTTAGGGAAAAACATATTAGTAGCTTATATGCCATGGGAAGGTTACAATTTTGAGGATGCGGTACTTATTAGCGAACGTCTGATATATGGAGATATTTATACTTCTTTTCACATACGGAAATATGAAATTCAGACTCATGTGACAAGCCAAGGTCCCGAAAGGATCACTAATGAAATACCACATCTAGAAGCCCGTTTACTCCGCAATTTAGACAAAAATGGAATTGTGATGCTGGGATCTTGGGTAGAGACCGGCGATATTTTAGTAGGTAAATTAACGCCTCAGGCAGCGAAAGAATCATCTTATGCTCCGGAAGATAGATTATTGCGGGCCATACTTGGCATTCAGGTCTCCACTTCAAAGGAAACTTGCCTAAAACTACCTATAGGTGGTAGGGGTCGAGTTATTGATGTGAGATGGGTCCAGAAAAAGGGGGGTTCTAGTTATAATCCAGAAACGATTTGTGTATATATTTCACAGAAACGTGAAATCAAAGTAGGTGATAAAGTAGCTGGAAGACATGGAAATAAGGGTATCATTTCCAAAATTTTGCCTAGACAGAATATGCCTTATTTGCAAGATGGAAGACCTGTTGATATGGTTTTCAACCCATTAGGAGTACCCTCGCGAATGAATGTAGGACAGATATTTGAATGCTCGCTCGGGTTAGCGGGGGGTCTGCTAAATCGCCATTATCGAATAGCACCTTTTGATGAGAGATATGAACAAGAGGCTTCGAGAAAACTCGTGTTTTCTGAATTATATGAAGCCAGTAAGCAAACAGCGAATCCATGGGTATTTGAGCCGGAATACCCGGGAAAAAGCAGAATATTTGATGGACGAACGGGAGATCCTTTTGAACAACCTGTTCTAATAGGAAAGCCTTATATCTTGAAATTAATTCATCAAGTTGATGATAAAGTACACGGACGTTCCAGTGGGCATTATGCACTTGTTACCCAACAACCCCTTAGAGGAAGGTCAAAACAGGGGGGGCAGCGCGTAGGAGAAATGGAGGTTTGGGCTCTAGAGGGCTTTGGTGTTGCTCATATTTTACAAGAGATGCTTACTTATAAATCTGATCATATTAGAGCTCGCCAAGAAGTACTTGGTACTACAATCATTGGAGAAACAATACCTAGCCCCGAAGATGCTCCAGAATCTTTTCGATTGCTCGTTCGAGAACTACGATCTTTGGCTTTGGAACTGAATCATTTTCTTGTATCTGAGAAAAACTTCCAGATTAATAAGAAGGAAGCTTAA

>psbD

ATGACTATAGCCCTTGGTAAATTTACCAAAGATGAAAAAGATTTATTTGATATTATGGATGACTGGTTACGGAGGGACCGATTCGTTTTTGTAGGTTGGTCCGGTCTATTGCTCTTTCCTTGTGCCTATTTCGCTTTAGGGGGTTGGTTCACAGGCACAACCTTTGTAACTTCATGGTATACCCATGGATTGGCCAGTTCCTATTTGGAAGGCTGCAACTTCTTAACCGCCGCAGTTTCGACTCCTGCTAATAGTTTAGCGCATTCTTTGTTGTTACTATGGGGTCCTGAAGCACAAGGGGATTTTACTCGTTGGTGTCAATTAGGCGGTCTTTGGACTTTTGTTGCTCTCCACGGTGCTTTCGGACTAATAGGTTTCATGTTACGTCAATTTGAACTTGCTCGCTCTGTGCAATTGCGACCTTATAATGCAATAGCATTCTCTGGTCCAATTGCTGTTTTTGTTTCTGTATTCCTGATTTATCCACTGGGCCAGTCTGGTTGGTTCTTTGCGCCTAGTTTTGGTGTAGCGGCGATATTTCGATTCATCCTTTTTTTCCAAGGGTTTCATAATTGGACATTGAACCCCTTTCATATGATGGGAGTTGCCGGTGTATTGGGCGCAGCTCTGCTATGTGCTATTCATGGCGCTACCGTAGAAAATACTTTATTTGAAGATGGTGATGGTGCAAATACATTCCGCGCTTTTAACCCAACGCAAGCCGAAGAAACTTATTCGATGGTCACCGCTAACCGCTTTTGGTCCCAAATCTTTGGGGTTGCTTTTTCCAATAAACGTTGGTTACATTTCTTTATGTTATTTGTACCAGTAACCGGTTTATGGATGAGTGCTCTTGGAGTAGTCGGCCTAGCCCTGAACCTACGTGCTTATGACTTCGTTTCCCAGGAAATCCGTGCAGCGGAAGATCCTGAATTTGAGACTTTCTACACAAAAAATATTCTTTTAAACGAGGGTATTCGTGCTTGGATGGCGGCTCAAGATCAGCCTCATGAAAACCTTATATTCCCTGAGGAGGTTCTACCCCGTGGAAACGCTCTTTAA

>psbC

ATGAAAACCTTATATTCCCTGAGGAGGTTCTACCCCGTGGAAACGCTCTTTAATGGAACTTTAGCTGTAGCAGGTCGTGACCAAGAAACCACCGGTTTCGCTTGGTGGGCCGGGAATGCCCGACTTATCAATTTATCCGGTAAACTGCTGGGCGCTCATGTAGCCCATGCTGGATTAATCGTATTCTGGGCCGGGGCAATGAACCTATTTGAAGTGGCTCATTTCGTACCGGAAAAGCCCATGTATGAACAAGGATTAATTTTACTTCCCCACCTAGCTACTCTAGGCTGGGGGGTAGGCCCTGGTGGGGAAGTTATAGACACCTTTCCATACTTTGTATCTGGAGTACTTCACTTAATTTCCTCTGCTGTATTGGGCTTTGGCGGTATTTATCATGCCCTTCTGGGACCTGAGACTCTTGAAGAATCTTTTCCATTCTTCGGTTATGTATGGAAAGATAGAAATAAAATGACCACAATTTTGGGTATTCACTTAATCTTGTTGGGTATAGGTGCTTTTCTTCTAGTATTCAAGGCTCTTTATTTTGGGGGTGTATATGATACCTGGGCTCCGGGGGGGGGGGATGTAAGAACAATTAACAACTTGACCCTTAGCCCAAGTGTTATTTTTGGTTATGTACTAAAATCCTTCTTTGGAGGAGAGGGGTGGATTGTTAGTGTGGATGATTTGGAAGATATAATTGGAGGGCATGTATGGATAGGTTCCATTTGTATATTTGGTGGAATCTGGCATATCTTAACCAAACCTTTTGCGTGGGCTCGCCGTGCACTTGTATGGTCTGGGGAGGCTTACTTGTCTTATAGTTTAGGTGCTTTATCTGTTTTTGGTTTCATTGCTTGTTGCTTTGTCTGGTTCAATAATACCGCTTATCCTAGTGAGTTTTATGGGCCCACTGGGCCAGAAGCTTCTCAAGCTCAAGCATTTACTTTTCTAGTTAGAGACCAACGTCTTGGGGCTAACGTGGGATCCGCTCAAGGGCCTACTGGTTTAGGTAAATATCTAATGCGTTCCCCCACTGGAGAAGTCATTTTTGGAGGAGAAACTATGCGTTTTTGGGATCTGCGTGCTCCATGGTTAGAACCTCTAAGGGGTCCCAACGGTTTAGACTTGAGTAGGTTGAAAAAAGACATACAACCTTGGCAAGAACGACGTTCTGCGGAATATATGACTCATGCTCCTTTAGGTTCTTTAAATTCTGTGGGTGGCGTAGCTACCGAGATCAATGCAGTTAATTATGTCTCTCCGAGAAGTTGGTTAGCTACTTCTCATTTTGTTCTAGGCTTCTTCCTATTCGTAGGTCATTTATGGCACGCGGGAAGAGCTCGTGCAGCTGCTGCAGGGTTTGAAAAAGGAATTGATCGTGATTTTGAACCTGTTCTTTCGATGACCCCTCTTAACTGA

>psbZ

ATGACTATTGCTTTCCAATTGGCTGTTTTTGCATTAATTGCTACTTCATCAATCTTACTGATTAGTGTACCCGTTGTATTTGCTTCTCCGGATGGTTGGTCGAGTAACAAAAATGTCGTATTTTCTGGTACATCATTGTGGATTGGATTAGTCTTTCTGGTGGGTATCCTTAATTCTCTCATCTCTTAA

>rps14

ATGGCAAGGAAAAGTTTGATTCATAGGGAGAAGAAAAGGCAAAAATTGGAACAAAAATATCATTTGATTCGCCGATCCTCAAAAAAAGAAATAAACAAAGTTCCGTCGTTGAGCGATAAATGGAAAATTCATGGAAAGCTACAATCCTCACCGCGTAATAGTGCACCTACACGTCTTCATCGACGTTGTTTTTCGACCGGAAGGCCGAGAGCTAACTATCGAGACTTTGGACTATCCGGGCACATACTTCGTGAAATGGTTCATGCATGTTTGTTGCCGGGGGCAACAAGATCAAGTTGGTAA

>psaB

ATGGCATTAAGATTTCCAAGGTTTAGCCAAGGCTTAGCTCAGGACCCCACTACTCGTCGTATTTGGTTTGGTATTGCTACCGCACATGACTTCGAGAGTCATGATGATATTACTGAGGAACGTCTTTATCAGAATATTTTTGCTTCTCACTTCGGGCAATTAGCAATAATTTTTCTGTGGACTTCCGGAAATCTGTTTCATGTAGCTTGGCAAGGAAATTTTGAGGCATGGGTACAGGACCCTTTACATGTAAGACCTATTGCTCATGCAATTTGGGATCCTCATTTTGGTCAACCGGCCGTGGAAGCTTTTTCTCGGGGAGGTGCTCTTGGCCCGGTGAATATCGCTTATTCTGGTGTTTATCAGTGGTGGTATACAATTGGTTTACGCACTAATGAGGATCTTTATACTGGAGCTCTTTTTCTATTATTTCTTTCTGCCATATCCTTAATAGCGGGTTGGTTACACCTACAACCGAAATGGAAACCGAGCGTTTCGTGGTTCAAAAATGCCGAATCTCGTCTCAATCATCATTTGTCAGGACTGTTCGGAGTAAGTTCCTTGGCTTGGACAGGACATTTAGTACATGTCGCTATTCCTGGATCCAGGGGGGAATATGTTCGATGGAATAATTTCTTAGATGTATTACCACATCCCCAAGGGTTAGGCCCACTTTTTACAGGTCAGTGGAATCTTTATGCTCAAAACCCCGATTCAAGTAGTCATTTATTTGGTACCTCCCAAGGATCAGGAACTGCCATTCTAACCCTTCTCGGGGGATTCCATCCACAAACGCAAAGTTTATGGCTGACCGATATTGCTCATCATCATTTAGCTATTGCAATTCTTTTCCTGATCGCGGGTCACATGTATAGAACAAATTTCGGGATTGGGCACAGTATAAAAGATCTTTTAGAAGCACACATTCCTCCGGGAGGACGATTGGGGCGCGGGCATAAGGGCCTTTATGACACAATCAACAATTCGCTTCATTTTCAATTAGGCCTTGCTCTAGCCTCTTTAGGGGTTATTACTTCCTTGGTAGCTCAACACATGTACTCTTTACCTGCTTATGCATTCATAGCGCAAGATTTTACTACTCAAGCTGCGTTATATACCCATCACCAATACATCGCAGGATTCATCATGACCGGAGCTTTTGCTCATGGAGCTATCTTTTTTATTAGAGATTACAATCCGGAACAGAATGAGGATAATGTATTGGCAAGAATGTTAGACCATAAGGAAGCTATTATATCCCATTTAAGTTGGGCCAGTCTGTTTCTGGGGTTCCATACTTTGGGACTTTATGTTCATAATGATGTCATGCTTGCTTTTGGTACTCCGGAGAAACAAATCTTGATCGAACCGATATTTGCCCAATGGATACAATCTGCTCATGGTAAAACTTCATATGGATTCGATGTACTTTTATCTTCAACGAATGGACCTGCATTCAATGCGGGTCGAAGCATATGGTTGCCTGGCTGGTTAAATGCTATTAATGAGAATAGTAATTCTCTATTCTTAACAATAGGCCCTGGAGACTTCTTGGTTCATCATGCTATTGCTCTAGGTTTACATACAACTACATTGATCTTAGTAAAGGGGGCTTTAGATGCACGCGGTTCCAAGTTAATGCCAGATAAAAAGGATTTCGGTTATAGTTTTCCTTGCGACGGTCCGGGACGAGGCGGTACTTGTGATATTTCGGCTTGGGACGCATTTTATTTGGCAGTTTTCTGGATGTTAAATACCATTGGGTGGGTTACTTTTTATTGGCATTGGAAACACATCACGTTATGGCAGGGTAACGTTTCACAGTTTAATGAATCTTCCACTTATTTGATGGGCTGGTTAAGAGATTATCTATGGTTAAACTCTTCCCAACTTATCAATGGGTATAACCCGTTTGGTATGAATAGTTTATCAGTCTGGGCGTGGATGTTCTTATTTGGACATCTTGTTTGGGCTACTGGATTTATGTTCTTAATTTCCTGGCGCGGGTATTGGCAAGAATTGATTGAAACTTTAGCATGGGCTCACGAACGCACACCCTTGGCTAATTTGATTCGATGGAGAGATAAACCAGTGGCTCTTTCCATTGTGCAAGCAAGATTGGTTGGATTAGCCCACTTCTCTGTAGGTTATATATTCACTTATGCGGCTTTCTTGATTGCCTCTACATCGGGCAAATTTGGTTAA

>psaA

ATGATTATTCGTTCGCCGGAACCAGAAGTAAAAATTTTGGTAGATAGGGATCCTATAAAAACTTCTTTCGAGGAATGGGCCAGACCGGGTCATTTCTCAAGAACCATAGCTAAGGGACCTGATACTACCACTTGGATCTGGAACCTACATGCTGATGCTCACGACTTCGATAGCCATACCAGTGATTTGGAGGAGATCTCTCGAAAAGTATTTAGTGCCCATTTCGGACAACTCTCCATCATTTTTCTTTGGCTGAGCGGCATGTATTTCCACGGTGCTCGTTTTTCCAATTATGAAGCCTGGCTAAGCGATCCTACTCACATTGGACCCAGTGCACAGGTGGTTTGGCCAATAGTGGGCCAAGAAATATTGAACGGTGATGTGGGCGGGGGTTTCCGAGGAATACAAATAACCTCCGGGTTTTTTCAGCTTTGGCGAGCATCTGGAATAACTAATGAATTACAACTCTATTGTACCGCAATTGGCGCATTGATTTTTGCAGCCTTAATGCTTTTTGCTGGTTGGTTCCATTATCACAAAGCTGCTCCAAAATTGGCTTGGTTTCAGGATGTAGAATCTATGTTGAATCACCATTTAGCGGGGCTGCTAGGGCTCGGGTCCCTTTCTTGGGCCGGGCATCAAGTACATGTATCTTTACCGATTAACCAATTTCTAAACGCTGGAGTAGACCCTAAAGAGATTCCACTTCCTCATGAATTTATCTTGAATCGGGATCTTTTGGCTCAACTTTATCCCAGTTTTGCCGAAGGAGCAACCCCATTTTTTACCTTGAATTGGTCAAAATATGCGGACTTTCTTACTTTTCGTGGCGGATTAGATCCAGTAACTGGGGGTCTATGGCTGACCGATATTGCACACCATCATTTAGCTATTGCAATTCTTTTCCTGATCGCGGGTCACATGTATAGGACCAACTGGGGAATTGGTCATGGTCTAAAAGATATTTTAGAGGCTCATAAAGGTCCATTTACAGGTCAAGGCCATAAAGGACTATATGAGATCCTAACAACATCATGGCATGCTCAATTATCGCTTAACCTAGCTATGTTAGGATCTTTAACCATTGTTGTAGCTCACCATATGTATTCCATGCCCCCTTATCCATATCTAGCTACTGACTATGGTACACAACTGTCATTGTTCACACATCACATGTGGATTGGTGGATTTCTCATCGTTGGCGCTGCTGCGCATGCAGCCATTTTTATGGTAAGAGACTATGATCCAACTACTCGATACAACGATCTCTTAGATCGTGTCCTTCGGCATCGCGATGCAATCATATCACATCTCAACTGGGTATGTATATTTCTAGGATTTCACAGTTTTGGTTTGTATATTCATAATGATACCATGAGTGCTTTAGGGCGTCCACAAGATATGTTTTCAGATACCGCGATACAATTACAACCCGTCTTTGCTCAATGGATACAAAACACCCACGCCTTAGCGCCTAGTGGAACGGCCCCTGGTGCAACAGCAAGCACCAGTTTGACTTGGGGGGGTGGTGATTTAGTGGCAGTGGGTGGAAAAGTTGCTTTGTTACCTATTCCATTAGGAACCGCGGATTTTTTGGTACATCACATTCACGCATTTACGATTCATGTGACGGTATTGATACTCCTGAAAGGAGTTCTATTTGCTCGTAGCTCGCGTTTGATACCGGATAAAGCAAATCTTGGGTTTCGTTTCCCTTGTGATGGGCCTGGAAGAGGGGGGACATGTCAAGTATCCGCTTGGGATCATGTCTTCTTAGGACTATTCTGGATGTACAATGCAATTTCCGTAGTAATATTCCATTTCAGTTGGAAAATGCAGTCAGATGTTTGGGGTAGTATAAGCGATCAGGGGGTGGTAACTCATATTACAGGAGGAAACTTTGCGCAGAGTTCCATTACGATTAATGGGTGGCTCCGCGATTTTTTATGGGCACAGGCATCCCAGGTAATTCAGTCTTATGGTTCTTCATTATCTGCGTATGGCCTTTTTTTCCTAGGTGCTCATTTTGTCTGGGCTTTTAGTTTAATGTTTCTATTCAGCGGGCGTGGTTACTGGCAAGAACTTATTGAATCCATCGTTTGGGCTCATAATAAATTAAAAGTTGCTCCTGCTACTCAGCCTAGAGCCTTGAGCATTGTACAAGGACGTGCTGTAGGAGTAACCCATTACCTTCTGGGTGGAATTGCCACAACATGGGCGTTCTTCTTAGCAAGAATTATTGCAGTAGGATAA

>ycf3

ATGCCTAGATCGCGGATAAATGGAAATTTTATTGATAAGACTTTTTCAATTGTAGCCAATATATTATTACGAATAATTCCGACAACTTCAGGAGAAAAAGAGGCATTTAGCTATTACAGAGATGGTATGTCAGCTCAATCCGAAGGCAATTATGCGGAAGCTTTACAGAATTATTATGAAGCTATGCGACTAGAAATCGATCCCTACGATCGAAGCTATATACTCTATAACATAGGCCTTATCCACACAAGTAACGGAGAACATACAAAAGCTTTAGAATATTATTTTCGGGCACTCGAACGAAATCCATTCTTACCACAAGCTTTGAATAATATGGCTGTGATCTGTCATTACCGGGGAGAACAGGCCGTTCGGCAGGGAGATTCTGAAATTGCGGAGTCTTGGTTCAATCAAGCCGCCGAGTATTGGAAACAAGCTATTGCGCTTACTCCTGGTAATTATATTCAAGCGCAGAATTGGTTGAAGATCACGGGGCGTTTCGAATAA

>rps4

ATGTCACGTTACCGAGGGCCTCGTTTCAAAAAAATACGCCGTCTGGGGGCTTTGCCGGGACTAACGAGTAAAAGGCCTAGAGCCGGAAGCGATTTTAGAAACCAATCGCGCTCCGGAAAAAAATCTCAATATCGAATTCGTTTAGAAGAAAAACAAAAATTGCGTTTTCATTATGGTCTTACAGAACGACAATTACTTAAATATGTTCGTATCGCCGGAAAAGCCAAAGGGTCAACCGGTCTGGTTTTACTACAATTACTTGAAATGCGTTTGGATAACATTCTTTTTCGATTGGGTATGGCTTCAACTATTCCTCAAGCCCGCCAATTAGTTAACCATCGACATATTTTAGTTAATGGGCGTATAGTCGATATACCAAGTTATCGTTGCAAACCCCGAGATATTATTACAGCAAGGGATGACCAAAAATCTAGATCTCTGATTCAAAATTATCTTGATTCATCCCACCATGAGGAATTGCCAAAGCATTTGACTCTTCACGCATTCCAATATAAAGGATTAGTCAATCAAATAATAGATAGTCAATGGGTCGGTTTGAAAATAAATGAATTGCTTGTCGTAGAATATTATTCTCGTCAGACTTAA

>ndhJ

ATGCAGGGTCGTTTGTCTGCTTGGCTAGTCAAGCATGGGCTAGTTCATAGATCTTTGGGCTTCGATTACCAAGGAATAGAAACTTTACAAATAAAGCCCGAGGATTGGCATTCCGTTGCTGTCATTTTGTATGTATATGGTTACAATTATCTACGTTCCCAATGTGCCTATGATGTAGCACCGGGTGGACTGTTAGCCAGTGTGTATCATCTTACGCGACTAGAGTATGGTGTAGATCAACCAGAAGAGGTATGCATAAAAGTATTTGCTCCAAGGAGGAATCCGAAAATTCCATCCGTTTTCTGGGTTTGGAAAAGTGCGAATTTTCAAGAACGGGAATCTTATGATATGTTGGGAATCCTTTATGATAATCATCCACGACTGAAACGTATCTTAATGCCGGAAAGTTGGATAGGGTGGCCTTTGCGTAAGGATTATATTGCCCCCAATTTTTATGAAATACAAGATGCATATTGA

>ndhC

ATGTTTCTGCTTTACAAATATGATATTTTCTGGGCATTTCTAATAATATCAAGCGTTATTCCTATTTTGGCATTTCTAATTTCCGCAGTTTTAGCCCCGATTAACAAAGGGCCAGAGAAACTTTCTAGTTATGAATCGGGTATAGAACCAATGGGCGATGCTTGGTTACAATTTCGAATCCGGTATTATATGTTTGCTCTAGTTTTTGTTGTTTTTGATGTTGAAACCGTTTTTCTTTATCCATGGGCAATGAGTTTTGATGTATTGGGGGTATCCGTATTTCTAGAAGCTTTCATTTTCATGCTTATCCTAATTGTTGGTTCAGTTTATGCGTGGCGAAAAGGAGCATTAGAGTGGTCTTAG

>atpE

ATGACCTTAAATCTTTGTATACTGACCCCGAATCGAATTGTTTGGGATTCAGAAGTGAAAGAAATCATTTTATCGACTAATAGTGGACAAATAGGCGTATTACCAAATCACGCGCCTATTGCGACAGCTGTAGATATAGGTATTTTAAGAATCCGCTTTAACGACCAATGGGTAACGATGGCTCTGATGGGTGGTTTTGCTAGAATAGGGAATAATGAGATCACTATTTTAGTAAATGATGCGGAGAAGAGTAGTGACATTGATCCCCAAGAAGCCCAGCAAACTCTTGAAATAGCGGAAGCTAATTTGAGGAAAGCTGAAAGCAAGAGACAAACAATTGAGGCAAATCTAGCTCTCAGACGAGCTAGGACACGAGTAGAGGTTATCAATGCGATTTGA

>atpB

ATGAAAATAAATCCTACTACTTCCGGCCCTGGGGTTTCCGCGCTTGCAAAAAAAAACCTGGGACATATCGCTCAAATCATTGGTCCGGTACTGGATGTAGCCTTTCCCCCCGGCAAGATGCCTAATATTTACAATGCTCTCGTAGTTAAGGGTCGAGATACTGTCGGTCAACCAATTAATGTGACTTGCGAGGTACAGCAATTATTAGGGAATAATCGGGTTAGAGCTGTAGCCATGAGTGCTACAGATGGTCTAACGAGAGGGATGGAAGTGATTGACACGGGAGCTCCTCTAAGTGTTCCAGTCGGCGGAGCGACTCTAGGACGAATTTTCAACGTGCTTGGAGAGCCTGTTGATAATTTAGGTCCTGTAGATACTCGCACAACATCCCCTATTCATAAATCCGCGCCTGCCTTTATACAGTTAGATACAAGATTATCTATTTTTGAAACAGGAATTAAAGTAGTAGATCTTTTAGCTCCTTATCGTCGGGGAGGAAAAATCGGACTATTCGGGGGAGCTGGAGTGGGTAAAACAGTACTCATTATGGAATTGATCAACAACATTGCCAAAGCTCATGGGGGTGTATCCGTATTTGGCGGAGTCGGTGAACGTACTCGTGAAGGAAATGATCTTTACATGGAAATGAAAGAGTCTGGAGTAATTAATGAACAAAATATTGCGGAATCTAAAGTGGCTCTAGTCTACGGTCAGATGAATGAACCACCAGGAGCTCGTATGAGAGTTGGTTTGACGGCCCTAACTATGGCAGAATATTTCCGAGATGTTAATGAACAAGACGTACTTCTATTTATCGACAATATCTTCCGTTTCGTCCAAGCGGGATCCGAGGTATCCGCCTTATTGGGTAGAATGCCTTCTGCTGTGGGTTATCAACCTACCCTTAGTACCGAAATGGGTTCTTTACAAGAAAGAATTACTTCTACCAAAGAGGGGTCCATAACTTCTATTCAAGCAGTTTATGTACCCGCGGATGATTTGACTGACCCCGCTCCTGCCACGACATTTGCCCATTTAGATGCTACTACCGTACTATCAAGAGGATTAGCTGCTAAAGGTATCTATCCAGCAGTTGATCCTTTAGACTCAACGTCAACTATGCTCCAACCTCGGATCGTTGGCGAGGAACATTATGAAACTGCGCAAAGAGTTAAGCAAACTTTACAACGTTACAAAGAACTTCAGGACATTATAGCTATCCTTGGGTTGGACGAATTGTCCGAAGAGGATCGTTTAACCGTAGCAAGAGCGCGAAAAATTGAGCGTTTCTTATCACAACCCTTTTTCGTAGCAGAAGTATTTACCGGTTCCCCGGGGAAATATGTTGGTCTAGCAGAAACTATTAGAGGGTTTAAATTGATCCTGTCCGGAGAATTAGACGGTCTTCCTGAACAGGCCTTTTATTTGGTAGGTAACATCGATGAAGTTACTGCGAAGGCTACAAACTTAGAAATGGAGAGTAATTTGAAGAAATGA

>rbcL

ATGTCACCACAAACAGAGACTAAAGCGAGTGTTGGATTCAAAGCCGGTGTTAAAGATTATAAATTGACTTATTATACTCCTGACTATGTAACCAAAGATACTGATATCTTGGCAGCATTCCGAGTAAGTCCTCAACCCGGAGTTCCACCCGAGGAAGCGGGGGCTGCGGTAGCTGCGGAATCTTCTACTGGTACATGGACAACTGTGTGGACCGATGGGCTTACCAGCCTTGATCGTTACAAAGGGCGATGCTACAACATTGAGCCCGTTGCTGGAGAAGAAAATCAATATATATGTTATGTAGCTTACCCGTTAGACCTTTTTGAAGAAGGTTCTGTTACTAACATGTTTACTTCCATTGTGGGTAATGTATTTGGTTTCAAAGCCCTGCGCGCTCTACGTCTAGAGGATCTACGAATCCCTACCGCGTATACTAAAACTTTCCAAGGCCCGCCTCACGGCATCCAAGTTGAGAGAGATAAATTGAACAAGTATGGACGTCCCCTGTTGGGATGTACTATTAAACCTAAATTGGGGTTATCCGCTAAGAATTACGGTAGGGCAGTTTATGAATGTCTACGCGGTGGACTTGACTTTACCAAAGATGATGAGAACGTGAACTCCCAACCATTTATGCGTTGGAGGGACCGTTTCTTATTTTGTGCGGAAGCACTTTATAAAGCGCAAGCTGAAACAGGTGAAATCAAAGGTCATTACTTGAATGCTACTGCAGGGACATGCGAAGAAATGCTAAAAAGGGCTGTCTTTGCCAGAGAGTTGGGAGTTCCTATCGTAATGCATGACTACTTAACAGGGGGGTTCACCGCAAATACTAGCTTGGCTCATTATTGCCGAGATAATGGTCTACTTCTTCACATCCACCGTGCAATGCATGCAGTTATTGATAGACAGAAGAATCATGGTATGCACTTTCGTGTACTAGCTAAAGCTTTACGTATGTCTGGTGGAGATCATATTCACGCTGGTACAGTAGTAGGTAAACTTGAAGGGGAAAGAGACATAACTTTGGGATTTGTTGATTTACTACGTGATGATTTTATTGAAAAAGATAGAAGCCGCGGTATTTATTTCACTCAAGATTGGGTCTCTTTACCAGGTGTTTTGCCCGTGGCTTCCGGAGGTATTCACGTTTGGCATATGCCTGCTTTGACCGAGATCTTTGGAGATGATTCCGTACTACAATTTGGTGGAGGAACTTTAGGACACCCTTGGGGAAATGCACCAGGCGCCGTAGCTAATCGAGTAGCTCTAGAAGCATGTGTACAAGCTCGTAATGAAGGACGCGATCTTGCTCGCGAAGGTAATGAAATTATCCGGGAGGCGAGCAAATGGAGTCCTGAACTGGCTGCTGCTTGTGAAGTATGGAAGGAGATCAAATTTGAATTCCCAGCAATGGATACTTTGTAA

>accD

ATGAAAAAAAGGTGGTTCAATTCGATTAAAGGTAAGGAGGAATTAGAATACAGGTGTGGGTTAAGTAAATCAATGGATAGTCTTGGTCCTATTCAAAATACCAGTGTAAGCGAGGACCCGATTCGAAATGATAAGGATAAAAACATTCATAGTTCGAGTGATAGTGACTGTTCGAGTTACAGCAATTTAGCTGGTGTCAGGGACATTCGTAATTTCATCTCGGATGACACCTTTTTTGTTAAGGATAGTAATAGGGACAGCTATTCCATATATTTTGATATTGAAAATCGAATTTTGGAAATAGACAACGATCATTCTTTTCTGAGTGAACCCGAAAGTTCTTTTTATAGCTTTCGTAATTCTAGTTCTAGGAATAATGGATCCAAAAGTGATGATCCCGACTATGATCGTTACATGTATGATACTCAATCGAGTTGGAATAATCACATTCATAATTGCCTCGACTATTATCTTCATTCTCAAATCTGTATTGATAGTCACATTTTAAGTAGTAGTGACTATTATAGTGCCAGTTACATTTATAATTTCATTTCTAGTGAAAGTGGAAATAGTAGTGAAAGCGAGAGTTCCAATATACAAAGTAGCACGAATGGTAGTGATTTAACTATAAGCGAAAGTTCTGATGATCTCGATGTAACTCAAAAATACAGGCATTTATGGGTTCAATGCGAAAATTGTTATGGATTAAATTATAAGAAATTTCTTAAGTCAAAAATGTATATTTGTGAACAATGTGGATTTCATTTGAAAATGAGTAGCTCAGATAGAATCGAACTTTCGGTTGATCCAGGTACTTGGGATCCGATGGATGACGACATGGTCTCTATAGATCCCATTGAATTTAATTCAGAAGAGGAACCTTATAAAAATCGTATTGATTCTTATCAAACAAAGACAGGATTAACGGAGGCTGTTCAAACAGGTACAGGGCAACTAAACGGGATTCCCATCGCAATTGGGGTTATGGATTTTCAGTTTATGGGGGGTAGTATGGGATCCGTAGTAGGCGAGAAAATCACCCGTTTGATCGAGTATGCTGCCAATCAAATTTTACCTCTTCTTCTAGTGTGTGCTTCCGGGGGAGCACGCATGCAAGAAGGAAGTTTGAGCTTGATGCAAATGGCTAAAATATCTTCCGCTTTATATGATTATCAATTAAATAAAAAGTTATTCTATGTATCAATTCTTACATCTCCTACTACTGGTGGAGTGACAGCTAGTTTTGGTATGTTGGGGGATATCATTATTGCTGAACCTAATGCCTATATTGCATTTGCGGGTAAACGAGTAATTGAACAAACATTGAATAAGACAGTACCTGAAGGTTCCCAAGCAGCTGAATATTTATTCCATAAGGGCTTATTCGATCCAATCGTACCACGTAATCCTTTAAAGGGTGTTCTGAGCGAGTTATTTCAGTTCCACGCCTTTTTTCCTTTGAATCAAAATTAA

>psaI

ATGACAATTCTCAACAGCTTTCCCTCTATTTTTGTGCCTTTAGTGGGCCTAGTATTTCCGGCAATGGCAATGGCTTCTTTATTTCTTTATCTTGAAAAAAATAAGATTTTTTAA

>ycf4

ATGCAACTGGAGCTAGTATGTATGAGTTGGCGATCAGAATATATATGGATAGAGTTTATAGCAGGCTCTCGCAAAACAAGCAATTTCTGCTGGGCCCTTATCATTTTTTTAGGTTCATTAGGATTCTTAGTGGTTGGAATTTCTAGTTATCTTGATAGGAATTTGCTATCTTTATTTCCGTCTCAGCAAATCAATTTTTTTCCACAAGGGATCGTGATGTCTTTCTACGGGATCGCGGGTCTCTTTATTAGTTCCTATTTGTGGTGCACAATTACATGGAATGTAGGTAGCGGTTATGATCGATTTGATACAAAAGAGGGAATAGTGTGTATTTTTCGTTGGGGATTTCCTGGAAAAAATCGCCGCATCTTTCTCCGATTCCTTATGAAAGATATTCAGTCCATCAGAATAGAAGTTAAAGAGGGTATTTATGCTCGTCGTGTCCTTTATATAGAAAGCAGAGACTTGGGGGCCATTCCCTTGAATCGTACTGATGAGAATTTGACCCCACGAGAAATTGAGCAAAAGGCTGCGGAATTGGCCTATTTCTTGCGTGTACCAATTGAAGGGTTTTGA

>cemA

ATGACAAAAAAGAGCGCATCCATTCCCCTTAGATATCTTTTATCTATAGTATTTGTAGTATTTTTGCCCTGGTGGATCCCTCTCTCATTTAATAAAAGTCTGGAATCCTGGGTTACTAATTGGTGGAATACTAGTCAACCCGAAACCTTTTTGAACGATATTCAGGAAAAGGCTATTCTAGAAAAATTCATAGAATTAGAGGAATTATTCCTCTTGGACGAAATGATAAAGGAATTTCCGGAAAGACATCTAGAAAAGCTTCGTATAGGGCTCCAGAAAGAAAGAGTCCAATTAATCAAGATGCACGATGAGGATCATATCCATACGATTTTTCACTTCTCGACAAATACAATCTGCTTTGTTATTCTAAGTGGTTATTCGATTCTGTGTAATGAAGAACTTTTTATTCTTAACTCTTGGGTTCAAGAATTCCTATATAATTTAAGCGACACAATAAAAGCCTTTTCGATTCTTTTCGTAACTGATTTATGTATCGGATTCCATTCGCCCCGCGGTTGGGAACTACTGATTGGCTATGCCTACAACGATTTTGGATTTGCTCATAATGATATTATTCTATCTGTTCTTGTTTCCACTTTTCCAGTCATTCTAGATACGTTTTTTAAATATTGGCTTTTTTCTTATTTAAATCGTGTATCTCCGTCACTTGTAGTGATTTATCATTCAATGACTGAGTGA

>petA

ATGCAAATTAGAAATACCTTTTCTTCATTAAAGGGAGAGATTACTCGATTCATTTCCGTATCCCTCATGATATATATAATAACTCGGGCATCGATTTCAAATGCATATCCCGTTTTTGCGCAGCAGGGTTTTGAAAATCCACGCGAGGCAACTGGTCGTATTGTATGCGCCAATTGTCATTTAGCTAATAAGCCCGTCGATATTGAGGTTCCACAGGCGGTACTCCCTGATACCGTATTTGAAGCAGTTCTTAGAATTCCGCATGATATGCAACTGAAACAAGTTCTTGCTAATGGTAAAAAGGGGTCTTTGAATGTGGGGGCCGTTCTTATTTTACCAGAGGGGTTTGAATTAGCCCCCTCCGACCGCATTTCGCCCGAGATGAAAGAAAAGATAGGCAAGCTGTCTTTTCAGACCTACCGACCCACTAAAAAAAATATTCTTGTGATAGGGCCAGTTCCTGGTCAGAAATATAGTGAAATCACTTTTCCTATTCTTTCCCCGAACCCTGCGACCAATAAAGATGCTCACTTCTTAAAATATCCAATATACGTAGGTGGGAACAGGGGTAGGGGTCAGATTTATCCCGACGGGAACAAAAGTAACAATACGGTTTATAATGCCACAGCTTCGGGTATAGTAAGCAAAATCATACGAAAAGAAAAAGGGGGATACGAAATAACCATAACGGATGCATCGAATGGGCGTGAAGTAGTTGATATTATCCCTCCAGGACCAGAACTTCGTGTTTCAGAGGGCCAATCTATCAAACTTGATCAACCATTAACAAGTAATCCTAATGTAGGTGGGTTTGGTCAGGCAGATGCAGAAATAGTACTTCAAGACCCATTACGTGTCCAAGGCCTTTTGTTCTTTTTGGCATCTGTTGTTTTGGCACAAATCTTTTTGGTTCTTAAAAAGAAACAGTTTGAGAAGGTCCAATTGTCCGAAATGGATTTCTAG

>psbJ

ATGGCCGATACTACTGGAAGGATTCCCCTTTGGATAATAGGTACTGTAACTGGTATTCCTGTGATCGGTTTAATAGGCATTTTCTTTTATGGTTCATATTCCGGATTAGGTTCGTCCTTGTAG

>psbL

ATGACACAATCAAACCCGAACGAACAAAATGTTGAATTGAACCGTACCAGTCTCTACTGGGGGTTATTACTCATTTTTGTACTTGCTGTTTTATTTTCCAATTACTTCTTCAATTAA

>psbF

ATGACCATAGATCGAACCTATCCAATTTTTACAGTGCGATGGTTGGCTGTGCACGGACTAGCTGTACCTACCGTTTCTTTTTTGGGGTCAATATCAGCAATGCAGTTCATCCAACGATAA

>psbE

ATGTCTGGAAGCACAGGAGAACGTTCTTTTGCTGATATTATTACCAGTATTCGATACTGGGTCATTCATAGCATTACTATACCTTCCCTATTCATTGCGGGTTGGTTATTCGTCAGCACGGGGTTAGCTTACGATGTGTTTGGAAGCCCTCGCCCAAACGAGTATTTTACAGAGAGCCGACAAGGAATTCCATTAATAACCGGCCGTTTTGATTCTTTGGAACAACTCAACGAATTTAGTAGATCTTTTTAG

>petG

ATGATTGAAGTCTTTCTATTTGGAATCGTCTTAGGTCTAATTCCTATTACTTTGGCTGGATTATTCGTAACCGCATATTTACAATACAGACGTGGTGATCAGTTGGACCTTTGA

>psaJ

ATGCGAGATCTAAAAACATATCTTTCCGTGGCACCGGTACTAAGTACTCTCTGGTTCGGGTCTTTAGCAGGGTTATTGATAGAAATCAACCGTTTATTCCCGGACGCATTGACATTTCCTTTTTTTTAA

>rpl33

ATGGCCAAGGGTAAAGATGTCCGAGTAAGGGTTATTTTGGAATGTACTAGTTGTGTTCGAAACGGTGTTAATAAGGAATCAAGGGGTATTTCCAGATATATTACTCAAAAGAATCGACACAATACACCTAGTCGATTGGAATTGAGAAAATTCTGTCCCTATTGTTACAAACATACACTTCATGGGGAGATAAAAAAATAG

>rps18

ATGGATAAAACCAAGCGACTCTTTCTTAAATCCAAGCGATCTTTTCGTAGGCGTTTGCCCCCGATCCAATCGGGGGATCGGATTGATTATAGAAACATGACTTTAATTAGTCGATTTCTTAGTGAACAAGGAAAAATATTATCTAGACGGGTGAATAGATTGACCTTAAAAGAACAACGATTAATTACTATTGCTATAAAACAAGCTCGTATTTTATCTTCGTTACCTTTTATTAATAATGAGAAACAATTTGAAAGAAGTGGGTCAACCACTAGAACTCCAGGTCTTCGAACCAGAAAAAAATAG

>rpl20

ATGACCAGAATTAGACGAGGATATATAGCTCGGAGACGTAGAACAAAAATGCGTTTATTTGCATCAAGCTTTCGCGGGGCTCATTCAAGACTTAGTCGAACAATTACTCAACAGAAAATAAGAGCTTTGGTTTCGGCTCATCGTGATAGAGATAGGAAAAAAAGGGATTTTCGTCGTTTGTGGATCACTCGAATAAATGCAGTAATTCGCGGAAACAGGGTATCCTATATTTATAGTAGATTAATAAGCAATCTGTATAAGGCGCAGTTGGTTCTTAATCGTAAGATACTTGCACAAATAGCTATATCAAATAGGAATTGTCTTTATATGATTTCCAATGAGATTTTAAAATAA

>rps12

ATGCCAACTATTAAACAACTTATTAGAAACCCAAGACAGCCAATCAGAAACCTTACAAAATCCCCCGCTCTTGGGGGATGCCCTCAGCGCCGAGGAACATGTACAAGGGTGTATACTATCACCCCCAAAAAACCAAACTCTGCCTTACGTAAAGTTGCCAGAGTACGATTAACCTCTGGGTTTGAAATCACTGCTTATATACCCGGTATTGGCCATAATTCACAAGAACATTCTGTAGTCTTAGTAAGAGGGGGGAGGGTTAAGGATTTACCCGGTGTGAGATATCACATTGTTCGAGGAACCCTAGATGCTGTCGGAGTAAAGGATCGTCAACAAGGGCGTTCTAAATATGGGGTCAAAAAGCCAAAATAA

>clpP

ATGCCTATTGGTGTTCCAAAAGTACCTTATCGAAGTCCCGGGGACAAGCATCCATCTTGGGTTGACATATACAACCGACTTTATCGAGAAAGATTACTTTTTTTAGGTCAAATGGTTGAGAGTGATATCTCGAATCAACTTATTGGTATTATGGTATATCTCAGTATAGAGAACGAGACCAAGGATTTGTATTTATTTATCAACTCTCCTGGCGGATGGGTAATACCCGGAATAGCAATTTATGATACTATGCAATTTGTGCGACCAGATGTACAGACAATATGCATGGGATTGGCCGCCTCCATGGGGTCTTTCCTCCTGGCCGCAGGAGCAAGTACCAAACGTCTAGCATTCCCTCACGCTAGGGTCATGATCCATCAACCTATTGGCGCTTTTTATGGGGCACAAACGGGAGAATTTATCCTGGATACGGAAGAACTACTGAAACTGCGCGAAATCCTTACAATGGTTTATGTACAAAGATCGGGCAAGCCCTTATGGGTTGTATCCGAAGACATGGAAAGGGATACTTTTATGTCAGCAACAGAAGCCCAAGCTCATGGAATTGTTGATCTTGTAGCGGTTGGATAA

>psbB

ATGGGTTTGCCTTGGTATCGTGTTCATACCGTCGTATTGAATGATCCCGGTCGTTTGATTGCTGTCCATATAATGCATACAGCCCTGGTTGCGGGTTGGGCCGGTTCAATGGCTCTATATGAATTAGCTGTTTTTGATCCCTCCGATCCAGTTCTTGATCCAATGTGGAGACAAGGCATGTTCGTTATACCCTTCATGACTCGTTTAGGAATAACCGATTCATGGGGCGGTTGGAGTATTACGGGGGGTACGGTAACGAATCCGGGTATTTGGAGTTACGAAGGTGTAGCCGGGGCACATATTGTGTTTTCGGGCTTGTGCTTCTTGGCAGCTATCTGGCATTGGGTGTATTGGGATCTAGCAATATTTGTCGATGACCGTACGGGAAAACGCTCTTTGGATTTGCCTAAAATCTTTGGAATTCATTTATTTCTCTCAGGAGTGGCTTGCTTTGGTTTTGGGACATTTCATGTAACAGGATTGTATGGTCCTGGAATATGGGTGTCCGACCCGTACGGACTAACTGGAAAGGTACAATCTGTAAATCCAGCATGGGGTGTGGAAGGTTTTGATCCTTTTGTTCCAGGAGGAATAGCCTCTCATCATATTGCAGCAGGGACATTGGGCATATTAGCAGGCTTATTCCATCTTAGTGTCCGCCCACCTCAACGCCTATACAAAGGATTACGTATGGGCAATATTGAAACCGTTCTTTCCAGCAGCATCGCTGCTGTCTTTTTTGCAGCGTTTGTTGTTGCTGGAACTATGTGGTATGGTTCAGCAACTACTCCCATCGAATTATTTGGTCCCACCCGTTATCAATGGGATCAGGGATACTTTCAGCAAGAAATATATCGAAGAGTCAGTGCTGGACTAGCCGAAAATCAAAGTTTATCAGAAGCTTGGTCTAAAATTCCTGAAAAATTAGCTTTTTATGATTACATCGGAAATAATCCTGCGAAAGGGGGATTATTCAGAGCGGGTTCAATGGATAACGGGGATGGAATAGCTGTCGGGTGGTTAGGACACCCTATCTTTAGAGATAAAGAAGGGCGTGAACTTTTTGTACGTCGTATGCCTACTTTTTTTGAAACATTTCCAGTTGTTTTGGTAGACGGAGATGGAATTGTTAGAGCCGACGTGCCTTTTCGAAGGGCAGAATCGAAGTATAGTGTCGAACAAGTAGGTGTAACTGTTGAGTTCTATGGTGGCGAACTGAATGGAGTGAGTTATAGTGATCCTGCTACTGTGAAAAAATACGCTAGACGTGCTCAATTGGGTGAAATTTTTGAATTAGATCGTGCTACTTTGAAATCCGATGGTGTTTTTCGTAGCAGTCCAAGGGGCTGGTTTACTTTTGGACACGCTTCATTTGCTCTGCTTTTCTTCTTCGGACACATTTGGCATGGTGCTAGAACCTTGTTCAGAGATGTTTTTGCTGGTATTGACCCGGATTTGGATGCTCAAGTGGAATTTGGAGTATTCCAAAAACTTGGAGATCCAACTACAAGAAGACAAGTAGTCTGA

>psbN

ATGGAAACAGCAACCCTAGTCGCCATCTCTATATCTGGGTTACTTGTAAGTTTTACTGGGTACGCCTTATATACTGCTTTTGGGCAACCCTCTCAACAACTAAGAGATCCATTCGAGGAACACGGAGACTAG

>psbH

ATGGCTCCACAAACAGTTGAGGGTAGTTCTAGAGCTCGTCCAAAAATGACTTCTGCAGGGGGGTTATTGAAACCTTTGAATTCGGAATATGGTAAAGTAGCTCCTGGATGGGGGACTACTCCTTTGATGGGTATCGCAATGGCTCTATTTGCGATATTCCTGTCTATTATTTTGGAGATTTATAATTCGTCCGTTTTACTGGACGGAATTTCAATGAATTAG

>petB

ATGAGTAAAGTCTACGATTGGTTCGAAGAACGTCTCGAGATTCAGGCGATTGCAGACGATATAACTAGTAAATACGTTCCTCCTCATGTCAACATATTTTATTGTCTAGGAGGAATTACGCTTACTTGTTTTTTAGTACAAGTAGCTACAGGGTTTGCTATGACTTTTTACTACCGTCCGACCGTTACTGAGGCTTTTGCTTCTGTTCAATACATAATGACGGAAGCTAACTTTGGCTGGTTAATCCGATCGGTTCATCGATGGTCGGCAAGTATGATGGTCCTAATGACAATCCTGCACGTATTTCGTGTGTATCTAACTGGCGGTTTTAAAAAACCTCGCGAATTGACTTGGGTTACAGGCGTGATTCTGGCTGTATTGACCGCATCCTTTGGTGTAACCGGTTATTCTTTACCTTGGGACCAAATTGGGTATTGGGCAGTCAAAATTGTAACAGGCGTGCCAGAAGCAATTCCGGTAATAGGATCGCCTTTGGTAGAGTTATTACGCGGAAGTGCTAGTGTGGGACAGTCCACTTTGACTCGTTTTTATAGTTTACACACTTTTGTATTACCTCTTCTTACTGCCGTATTTATGTTAATGCATTTCCTAATGATACGTAAACAAGGCATTTCTGGCCCTTTATAA

>petD

ATGGGAGTAACAAAAAAACCTGACTTGAATGATCCTGTATTAAGGGCTAAGTTGGCTAAAGGTATGGGTCATAATTATTATGGCGAACCCGCATGGCCCAACGATCTTTTATATATTTTTCCAGTAGTAATTCTAGGTACTATTGCATGTAACGTAGGCTTAGCGGTTCTAGAACCATCAATGATTGGTGAACCCGCGGATCCATTTGCAACTCCTTTGGAAATATTACCCGAATGGTATTTCTTTCCCGTATTTCAAATACTTCGTACAGTACCCAATAAGCTGTTGGGTGTTCTTTTAATGGTTTCAGTACCTACGGGATTATTAACAGTACCTTTTTTGGAAAATGTTAATAAATTCCAAAATCCATTTCGCCGCCCAGTAGCGACAACCGTCTTTTTGATTGGTACCGCAGTGGCCTTGGGCTTGGGTATTGGAGCAACATTACCTATTGAAAAATCCCTAACTTTAGGTCTTTTTTAA

>rpoA

ATGGTTCGAGAGAAAGTAAAAGTATCTACTCGGACACTACAGTGGAAGTGTGTTGAATCAAGAGCAGACAGTAAGCGTCTTTATTATGGACGCTTTATTTTGTCTCCACTTATGAAAGGTCAAGCCGACACAATAGGCATTGCGATGCGAAGAGTTTTGCTTGGAGAAATAGAAGGAACGTGTATTACACGCGCAAAATCTGAGAAAATCCCACATGAATATTCTACCATAGTGGGTATTCAAGAATCGGTACATGAAATTTTAATGAATTTGAAAGAAATTGTATTGAGAAGTAATCTTTATGGAACTTGTGACGCGCTTATTTTTGTCAAAGGTCCGGGAGATGTAACTGCTCAAGACATCCTCTTGCCACCTTCTGTGGAAATCGTTGATAAGACGCAGCACATAGCTAGCCTAACAGAACCAATTGATTTGTGTATTGGATTACAAATCGAGAGGAGTCGAGGATATAATATAAAAACGCCAAATAACTTTCAAGACGGAAATTGTTATCCTATAGATGCTGTATTCATGCCTGTTCGAAATGCGAATCATAGTATTCAGTCTTATGGGAATGGCAATGAAAAACAAGAGATCCTTTTTCTAGAAATATGGACAAACGGGAGTTTAACTCCTAAAGAAGCACTTCATCAAGCCTCCCGGAGTTTGATTGATTTATTTATTCCCTTTCTCCAGGCAGCAGACGAAAACTTACATTTAGAGAACAATCAATACAAGGTTACTTTCCCTTTTTTTACTTTTCATGATAGATTGGCTAAACTAACGAAAAAGAAAAAAGAAATCGCATTGAAATCGATTTTTATTGACCAATCAGAATTGTCTCCCAGGATCTATAATTGTCTCAAAAAGTCCAATATACATACATTATTCGACCTTTTGAATAAGAGTCAAGAAGACCTTATGAAAATTGAACACTTTCGCCTAGACGATGTAAAGCAGATAATGGGTATTCTAGAAAAGAAATAG

>rps11

ATGGCAAAATCTACACCAAGAAGTGGTTCACGTAGGACTGGACGGATTGGTTCGCGCAAAAGTGGACGTCGAATACCAAAGGGCGTTATTCATGTTCAAGCAAGTTTCAACAACACCATTGTGACTGTTACGGATGTACGGGGTCGGGTAATTTCTTGGTCCTCGGCCGGTACTTGTGGATTCAGGGGTACAAGAAGAGGTACGCCCTTTGCTGCTCAAACCGCAGCAGGAAGTGCTATTCGAGCAGTAGCGGATCAAGGTATGCAACGAGCAGAAGTCATGATAAAGGGTCCTGGTCTCGGAAGAGATGCAGCATTACGAGCTATTCGTAGAAGCGGTATCCTTTTAAATTTCGTACGGGATGTAACCCCTATGCCACATAATGGTTGCAGACCCCCTAAAAAAAGACGGGTGTAG

>rpl36

ATGAAAATAAGGGCTTCCGTTCGTAAAATTTGTGAAAAATGTCGACTGATCCGCAGGAGGGGACGGATTATAGTAATTTGTTCCAACCCGAGACATAAACAAAGACAAGGATAA

>infA

ATGTTCCGGGTTCTGCTATCTGTTAGATGGTTTAGACAACTAAGTAAGATTCTAGGTTATGTTTCAGGAAGGATCCAACCCGTTTTTATACATATACTACCGGTGGATATAGTTAAAATTGAAGGAAGTCATTATGATTCAAAGGGAGGGCGTATAATTTATAGGCTACACAAAAGGATTTGA

>rps8

ATGGGCAAAGACACTATTGCTGACATAATAACTTCTATACGAAATGCTGACATGAATCGAAAGGGAACAGTTCGAATAGCATCTACTAACATCACTGAAAACATTGTTAAAATACTTTTGCGAGAGGGTTTTATAGAAAACGTAAGGAAACTCTTGGAAAACCAAAAAGAGTTTTTGGTTTTAACCCTACGCCATAGAAGGAATAGGAAAGGACCGTATAGACCCATTTTAAATTTAAAACGAATCAGTCGACCCGGTCTACGAATCTATTTTAACTATCAACGAATTCCTAGAATTTTAGATGGGATGGGGATTGTGATTCTCTCTACATCTCGGGGTATAATGACAGACCGAGCGGCTCGACTAGAAAGAATCGGCGGAGAGATTTTGTGTTATATATGGTAA

>rpl14

ATGATTCAACCTCAAACCCATTTGAATGTAGCGGACAATAGCGGTGCCCGAGAATTGATGTGTATTCGAATCATAGGAGCCAGTAATCGTAGATATGCTCATATTGGTGACGTTATTGTTGCTGTGATCAAGGAAGCAGTACCAAATACGCCTCTAGAAAGATCAGAAGTGATCAGAGCTGTAATTGTACGTACTTGTAAAGAACTCAGACGTGATAACGGTATGATAATACGTTATGATGACAATGCTGCAGTTGTCATTGATCAAGAAGGAAATCCAAAGGGAACTCGAGTTTTTGGTGCGATCGCCCGAGAATTGAGACAGTTGAATTTTACTAAAATAGTTTCATTAGCACCTGAAGTATTATAA

>rpl16

ATGCTTAGTCCCAAAAGAACAAGATTCCGTAAACAACATAGAGGAAGAATGAAAGGAATATCTTATCGAGGTAATCATATTTGTTTCGGCAGATATGCTCTTCAAGCACTTGAACCCGCTTGGATCACATCTAGACAAATCGAAGCAGGGCGCCGAGCAATGACACGAAATGTACGGCGTGGCGGAAAAATATGGGTACGTATATTTCCAGACAAACCAGTTACAGTAAGACCCACGGAAACCCGTATGGGGTCCGGGAAAGGATCCCCCGAATATTGGGTAGCCGTCGTTAAACCGGGTAGAATACTTTATGAAATGAGTGGAGTCGCTGAAAATATCGCTCGAAAGGCTATTTCAATAGCGGCGTCAAAAATGCCTATAAGAACTCAATTCATTATTTCTGGATAG

>rps3

ATGGGACAAAAAATAAATCCACTTGGTTTCCGGCTTGGTACAACACAAAGTCATCATTCGCTTTGGTTTGCAAAACCAAAAAATTATTGCGAAGGTCTACAAGAGGATCAAAAAATACGAAACGTTATTAAGAATTATGTACAAAAAAATATGAGAATATCCTCCGGTGTTGAGGGAATTGCCCGGATAGAGATTCAAAAAAGAATTGATCTAATTCAAGTCATAATCTATATAGGATTCCCAAAATTATTACTAGAAAATAGACCGCGAAGAATTGAAGAATTACAGATGAATGTACAAAAAGAACTTAATTGTGTGAACCGAAAAATAAACATTGCTATTACACGAATTGCAAATCCTTATGGACACCCCAATATTCTTGCCGAATTTATAGCGGGCCAATTAAAAAACAGAGTTTCTTTTCGCAAAGCAATGAAAAAAGCTATTGAATTAACTGAACAGGCAGATACAAAAGGAATTCAAGTCCAAATAGCAGGGCGTCTTGACGGAAAAGAAATTGCGCGCGCCGAATGGATCAGAGAAGGTAGAGTTCCTCTACAAACCATTGGAGCTAAAATTGATTATTGTTCCTATACAGTTCGAACTATATACGGGGTATTAGGAATCAAAATTTGGATATTTGTAGACGAAGAAAAATAA

>rpl22

ATGATAAGGATAATAAAGAAGAAGGTAGAAGTCTCTGCTTTAGGTCAACATATATGTATGTCTGCTCACAAAGCCCGAAGGGTAATTGATCAGATTCGTGGACGTTCTTACGAGGAAACCCTTATGATACTCGAACTCATGCCTTATCGAGCATGTTATCCCATTTTAAAATTGGTTTATTCTGCAGCAGCAAATGGTATTCACAATCTGGGTTTCAACGAAGGGAGTTTATTCATTATTAAAGCCGAAGTAAACGAGGGTACTGCTGCGAAAAGATTTAAACCTCGAGCTCGAGGACGGAGTTATATGATCAAAAGACCCACTTGTCATATAACTATTGTTTTAAAAGATATCTCTGTAGATGAATATGAATATAGGGACTATCTCGACTGTTCAAAAAGGACTGGATTAAAAAAAAAAAAAAGAACAAAACTATGA

>ndhF

ATGGAACATACATATCAATATTCCTGGATCATACCTTTAGTTCCACTTCCAGTCCCTATGTTAATAGGGGTGGGACTTCTACTTTTTCCGACCGCAACAAAACATCTTCGTCGTATGTGGGCTTTTCTTAGTATTTTATTGTTAAGTATAGTTATGATTTTTTCGATCGATCTATCTATTGAGCAAATAGATCGAACTTGTATCTATCAATCCCTAAGGTCTTGGACCATCAATAATGATTTTTCTTTCGAGTTCGGATACTTTATTGATCCACTTACTTCTATTATGTCAATATTAATCACTACAGTTGGAATTCAGGTTCTTGTTTATAGTGACAATTATATGTCTCATGATCAAGGATATTTGAGATTTTTTGCTTACATGAGTTTTTTCAATGCTTCCATGTTAGGATTAGTTACAAGTTCGAATTTGATACAAATTTATATTTTTTGGGAATTGGTTGGAATGTGCTCTTATCTATTAATAGGATTTTGGTTCACACGACCTATTGCGGCAGGCGCTTGTCAAAAAGCCTTTGTAACTAATCGTGTAGGGGATTTTGGATTATTATTAGGAATCCTAGGTCTTTATTGGATAACGGGTAGTTTCGAATTTCGGGATTTGTTCGAAATATTGAATAACTTGATTTATAATAATCAGGTTAACCTTTTATTTGTTACTTTGTGTGCATTTCTATTATTTGCCGGCCCGGTTGCTAAATCCGCGCAATTCCCTCTTCATGTATGGTTACCCGATGCCATGGAAGGGCCTACTCCTATTTCGGCTCTTATCCATGCTGCTACTATGGTAGCGGCGGGAATTTTTCTTGTAGCTCGTCTTCTTCCGCTTTTCATAGTCATACCGTACATAATGAATCTAATATCTTTGATAGGTATAATAACAGTATTTTTAGGAGCTACTTTAGCTCTTGCTCAACAAGATATTAAGAGAGGTTTAGCTTATTCTACAATGTCTCAATTGGGTTATATGATGTTAGCTCTAGGGATGGGGTCTTATCGAGCCGCTTTATTTCATTTGATTACTCATGCTTATTCCAAAGCCTTGTTGTTTTTAGGATCCGGATCAATTATTCATTCAATGGAAGCCATTGTTGGATATTTTCCAGATAAAAGCCAGAATATGGTTCTTATGGGTGGGTTAAGAAAGCACGTGCCAATTACAAAAACCGCCTTTTTGTTGGGTACCCTTTCTCTTTGTGGTATTCCGCCTCTCGCTTGTTTTTGGTCCAAAGATGAAATTCTTAATGATAGTTGGTTGTATTCGCCGATTTTCGCAATAATAGCTTTTTTCACAGCCGGATTAACCGCATTTTATATGTTTCGAATTTATTTACTTACTTTTGAGGGACCTTTCAACTTTTGCTTTCAAAATTACAGTGGTAAAAAAAGCTATTCCTTTTATTCAATATCTCTATGGGGTAAAGAAGAACCAAAACCAATTAAAAAAAATTTTCATTTAGTTTCTTTATTAACAATGAATAATAATGAAAAGGCTTCTTTTTTTTCGCAGAAGGCTCATCGAATTGATAGGAATGTAACAAATACGCCTTTTCTTACTATTTTTCCTTTTGGTGCTACCAAGACTTTTTGTTATCCTCACGAATCAGACAATACTATGTTATTTGTTATGCTTGTATTAGTCCTATTTCCTTTGTTTGTTGGAACGATAGGAATTCCTTTGACTCAAGAAGGAATCGGTTCGGATATTTTATCAAAATTGTTAACTCCGTCTATAAATCTTTTACATCAAAATTCAACTCATTTTGTTGATTGGTATGAAGTTTTGAAAAATCCAACCCTTTCCGTCAGTATAACGTATTTCGGAATCCTTCTAGCCTACTTTTTCTATAAGCCCTTTTATTCATCTTTACACAATTGGAACATACTAAATTTATTTGCTAAAAGAGGACCTAAGAGAATTCTTTGGGACAAAATACTCAATTTTCTATATGATTGGTCATATAATCGTGCTTATATAGATGCCTTTTACACAAGATCCTTAACGGAAGGGATAAGAGGATTAGCGGAACTAACGCATTTGTTCGACAGACGAGTAATTGATGGAATTACGAATGGGGTCGGTATTACAAATTTTTTTGTAGGGGAAGGTATAAAATATTTAGGGGGAAGTCGCATCTCTTTTTATCTCTTATTATATTTATTTTCAGTCTTAGTCTTCTTAATCTTTTTATTAATCTTTTTTTAA

>rpl32

ATGGCAGTTCCAAAAAAACGTACTTCTATATTAAAAAAACGTATTCGTAAGAATATTTGGAAAAAAGGGGGGTATTGGGCAGCGTTGAAGGCTTTTTCGTTAGCGAAATCCCTTTCTACTGGGAATTCAAAAAGTTTTTTTGTACAACAAATAAATAAGAAAACATTGAAATAA

>ccsA

ATGATCTTTTCAACTTTAGAGCATATATTAACGCATATATCCTTTTCGGTCGTTTCAATTGGAATTACAATTTATTTACTAACCTTATTAGTCGATGAAATCAGAGGACTATATGATTCATCAGAAAAGGGTATGATAGCTACCGCTTTCTGTCTAACAGGATTATTAATCACCCGTTGGATTTACTCGAGACATTTCCCATTAAGTGATTTATATGAATCATTAATCTTTCTTTCATGGAGTTTCTCCATTATTCATAGGATTTTCGATTTAAAAAAAAATAAAAATCATTTAAGTGCTATAACGGCACCAAGTGCTATTTTTACCCAAGGTTTTGCTACTTCGGGTTTTTTAACCAAAACCCATCAATCCGGAATATTAGTACCCGCTCTCCAAGTCCAGTGGTTAATGATGCACGTAAGTATGATGGTATTGGGCTATGCAGCTCTTGTATGTGGATCCTTATTATCCACGGCTCTTCTAGTCATTACATTTCGAAAAGTGATAAGGGTTTTTTTGAAAAGAAAAAATTTTGTAAATGGAAATGAGTCGTTTTGCTTCGGTGAAATCCAATACATGAACGAAAAAAGGACTGTTTTACTAAATACTTTTTCCGCTAGAAATTATTACAGGTATCAAGTGATTCAACAATTGGATCGCTGGAGTTATCGTATTATTAGTTTAGGATTTATCTTTTTAACCATAGGGATTCTTTCGGGAGCAGTATGGGCTAATGAGGCGTGGGGGTCTTATTGGAATTGGGATCCAAAAGAAACTTGGGCATTTATTACTTGGACTATATTCGGGATTTATTTACATACTCGAACAAATACAAATTTGGAAGGTGTAAATTCCGCAATTGTCGCTTCTACGGGCTTTCTTATAATTTGGGTATGCTATTTCGGAGTCAATCTATTAGGAATAGGGTTACATAGTTATGGTTCATTTAATTAA

>psaC

ATGTCACATTCAGTAAAGATTTATGATACATGTATAGGGTGTACTCAATGTGTCCGAGCTTGCCCCACAGATGTATTAGAAATGATACCTTGGGACGGATGTAAAGCAAAGCAAATTGCTTCTGCTCCAAGAACAGAGGACTGTGTTGGTTGTAAGCGATGCGAATCCGCCTGTCCAACGGATTTCTTGAGTGTTCGGGTTTATTTATGGCATGAAACAACTCGAAGCATGGGTCTAGCTTATTGA

>ndhE

ATGCTCGAGCATGTACTTGTTTTGAGTGCCTATTTATTTTCTATTGGTATCTATGGATTGATTACGAGCCGAAATATGGTTCGGGCCCTGATGTGTCTTGAACTTATACTAAATGCAGTTAATATCAATTTCGTAACATTCTCTGATTTTTTTGATAGTCGACAATTAAAAGGAAATATTTTCTCAATTTTTGTTATAGCTATTGCAGCCGCCGAAGCAGCTATCGGATCAGCTATTGTTTCGTCAATTTATCGTAACAGAAAATCGACTCGTATCAATCAATCGACTTTATTGAATAAATAG

>ndhG

ATGGATTTGCCTGGACCAATACATGATTTTCTTTTAGTTTTTCTGGGATCAGGTCTTATATTAGGAGGTCTGGGAGTGGTATTATTTACCAACCCGATTTATTCTGCCTTTTCCTTGGGATTGGTTCTTGTTTGTATATCCTTATTCTATATTCTAGCAAATTCCCATTTTGTAGCTGCCGCGCAGCTCCTTATTTACGTGGGAGCTGTAAATGTTTTAATCATATTTGCTGTAATGTTCATGAATGGTTCAGACTATTCCAAAGATTTTCATTTTAATCTTTGGACTGTTGGTGATGGGCTTACTTCCCTGGTTTGTACAAGTATTTTTTTTTCGCTAATCGCTACTATTCTAGATACGTCGTGGTACGGGATTATTTGGACTACACGACCCAACCAGATTATCGAACAAGATTTGATAAGTAATAGTCAACAAATTGGAATTCATTTATCAACAGACTTTTTTCTTCCATTTGAACTCGTTTCAATAATTCTTTTAGTTGCTTTGATAGGTGCAATTGCCGTGGCTCGTCAGTAA

>ndhI

ATGTTCCCTATGGTAACTGGTTTCATGAATTATGGTCAACAAACAATACGAGCTGCAAGGTACATTGGTCAAAGTTTCATGATTACTTTATCCCAAGCAAATCGTTTACCTGTAACTATTCAATATCCTTATGAAAAATTAATAACATCGGAGCGTTTTCGCGGTCGAATCCATTTTGAATTTGATAAATGTATTGCTTGTGAAGTATGCGTTCGCGTATGTCCTATAGATCTGCCTGTTGTTGATTGGAAATTTGAAACAGATATTCGAAAGAAACGATTGCTTAATTACAGTATTGATTTTGGAATTTGTATTTTTTGTGGTAACTGCGTTGAGTATTGTCCAACAAATTGTTTATCAATGACTGAAGAATATGAACTTGCTACTTACGACCGTCACGAATTGAATTATAATCAAATTGCTTTAGGTCGTTTACCAATGTCAGTCATTGACGATTTTACAATTCGAACAGTGTTGAATTCGCCTCAAAGAAAAAATGGAAAAAATGACTAA

>ndhA

ATGATAATTGATACAACAGAAGTACAAGATATCAATTCTTTTTCCAGATTGGAATCCCCTCAAGAAGTCTATGGGATCGTGTGGGTGCTTGCCCCTATTTCGACTCCTGTAGTAGCAATCACAATAGGTGTCCTAGTAATTGTGTGGTTAGAAAGAGAAATATCTGCAGGAATACAACAACGTATTGGGCCTGAATACGCCAGTCCCTTGGGAATTCTTCAAGCTTTAGCAGATGGAACAAAACTACTTTTCAAAGAAAACCTTCTTCCATCTAGAGGAAATAGTAGTTTATTCAGTATTGGACCATCTATAGCAGTCATAGCAATTCTACTAAGTTATTCAGTAATTCCTTTTAGTTATAACTTTGTTTTAGCTGACCTCAATATCGGTATTTTTTTATGGATTGCCATTTCAAGTATTGCCCCCATTGGACTTCTTATGTCAGGATATGGATCAAATAATAAATATTCCTTTTTAGGTGGTCTGCGAGCTGCTGCTCAATCGATTAGTTATGAAATACCATTAACTTTATGTGTTTTATCAATATCTCTATTATCTAACAGTTCAAGTACAGTTGATATAGTTGGAGCACAATCAAAATATGGTTTTTGGGGGTGGAATTTGTGGCGTCAACCTATAGGGTTTATCGTTTTTCTAATTTCTTCCCTAGCGGAATGCGAGAGATTACCTTTTGATTTACCAGAAGCAGAAGAAGAACTAGTAGCAGGTTATCAAACCGAATATTCAGGAATCAAATTTGGTTTATTTTACGTTGCTTCCTATCTAAATCTATTAGTTTCCTCATTATTTCTAACAGTTCTTTACTTGGGGGGTTGGAATCTTTCCATTCCATACATATTTGTTCCTGAGCTATTTGAAATAAATAAAGCGGATGGAATCTTTGGAACGACAATTGGTATCTTTATTACATTAGCTAAAACTTATTTGTTCTTGTTCGTTCCGATTACAACAAGGTGGACTTTACCGAGACTAAGAATGGACCAACTATTAAATCTTGGCTGGAAATTTCTTTTACCTATTTCTCTCGGTAATCTATTATTAACAACTTCTTCCCAACTCCTTTCGCTATAA

>ndhH

ATGAGTATACTAGCTACAGAAAAAGAATTTATGATAGTCAATATGGGACCTCACCACCCATCAATGCACGGTGTTCTTCGTCTCATCGTTACTCTAGATGGTGAAGATGTTATTGACTGTGAACCAATATTGGGTTATTTACACCGAGGGATGGAAAAAATTGCGGAAAATCGAACAATTATACAATATCTGCCTTATGTAACCCGTTGGGATTATTTAGCTACTATGTTCACCGAAGCAATAACTGTAAATGGACCCGAACTGTTGGGAAATATTCAAGTACCCAAAAGGGCCAGCTATATCAGAGTCATTATGTTGGAGTTGAGTCGTATAGCTTCCCATCTGTTATGGCTTGGCCCTTTTATGGCAGATATTGGCGCACAGACTCCTTTCTTCTATATTTTCAGAGAAAGAGAATTAGTATATGATCTGTTCGAAGCTGCCACCGGTATGAGGATGATGCATAATTATTTTCGTATCGGAGGAATAGCAGCTGATTTACCTCATGGTTGGATAGATAAATGTTTGGATTTCTGCGATTATTTTTTAACGGGGGTTGCTGAATATCAAAAACTTATTACGCGAAACCCTATTTTTTTAGAACGAGTTGAAGGAGTAGGCATTCTTGGTGGAGAAGAAGCAATAAATTGGGGTTTATCCGGACCAATGCTACGAGCGTCTGGAATAGAATGGGATCTTCGTAAAGTTGATCATTATGAGTGTTATGACGAATTTGATTGGGAAGTCCAGTGGCAAAAAGAAGGAGATTCATTAGCTCGTTATTTAGTCCGAATCGGTGAAATGACGGAATCCGTAAAGATTCTTCAACAGGCTTTAGAAGGAATTCCGGGAGGACCCTATGAAAATTTAGAAATCCGATGTTTTGATAGAGAAAACGATCCAGAAGGGAATGATTTTGAAGATCGATTCATTAGTAAAAAGCCTTCTCCCACCTTTGAATTGACGAAACAAGAACTTTATGTGAGAGTAGAAGCCCCAAAAGGAGAATTGGGAATTTTTCTGATAGGAGATCAAAGTGGTTTTCCTTGGAGATGGAAAATTCGCCCACCGGGTTTTATCAATTTGCAAATTCTTCCTCAGTTAGTTAAAAGAATGAAATTGGCTGATATTATGACAATATTAGGTAGTATAGATATCATTATGGGGGAAGTTGATCGTTGA

>rps15

ATGGTAAAAAATTCATTCGTCTCAGTTATGGTTCAAGAAAAAAAAGAAGAAAACTGTGGATCGGTTGAATTTCAAGTATTCCGTTTCACTAATAAGATACGGAGACTTACTTCACATTTAGAATTGCACAGAAAAGACTATTTATCTCAAAGGGGTCTACGGAAAATTTTGGAAAAACGCCAACGTCTGCTAGCTTATTTGTCAAAGAAAAATAGAGTACGTTATAAAGAATTAATTAGTAAGTTGAATATTCGGGAGTCCAAAAATCGTTAA

>ycf1

ATGATTTTTCAATCTTTTATACTAGGTAATCTAGTATCCTTATGCATGAAGATAATCAATTCGGTCGTTGTGGTCGGACTCTATTATGGATTTATGACCACATTCTCCATAGGGCCCTCTTATCTCTTCCTTCTCCGAGCTCGGGTTATGGAAGAAGGAGAAGAAGGAACCGAGAAGAAGGTATCAGCAACAACTGGTTTTATTGCGGGACAGCTCATGATGTTCATATCGATCTATTATGCGCCTCTGCATCTAGCATTGGGTAGACCTCATACAATAACTGTCCTAGCTCTACCGTATCTTTTGTTTCATTTCTTCTGGAACAATCCCAAACACTTTTTTGATTATGGATCTACTACCAGAAATTCAATGCGTAATCTTAGCATTCAATGTGTATTCCTGAATAATCTCATTTTTCAATTATTCAACCATTTCATTTTACCAAGTTCAATGTTAGCCAGATTAGTCAACATTTATATGTTTCGATGCAACAACAAGATGTTATTTGTAACAAGTAGTTTTGTTGGTTGGTTAATTGGTCACATTTTATTCATGAAATGGGTTGGATTGGTATTAGTTTGGATACAGCAAAAGAATTCTATTAGGTCTAATGGACTTATTCGATTTAATAAGTACCTTGTGTCAGAATTGAGAAATTCTATGGCTCGAATCTTTAGTATTCTCTTATTTATTACCTGTATCTACTATTTAGGCAGAATACCCTCACCCATTTTTACTAAGAAACTGAAAGTGAAAGAAACCTCAGAAACGGAAGAAAGAGATGTAGAAATAGAAACAACTTTCGAAAGGAGGGGGACTAAACAGGAACAAGAGGTATCCGCCGAAGAAGATCCTTCTCCTTCCCTTTTTTCGGAAGAAAAGGAGGATCCGGACAAAATCGAGGAAACGGAAGAGATCCGAGTGAATGGAAAGGAAAAAAAAAAAACAAAGCATGAATTCCACTTTCGCTTTAAAGAGACATGCGAGAAAAATAGTCCAGTTTATGAAACTTCTTATCTGGATGGGAATCAAGAAAATTCGAAATTACAAATATTTAAAGAAAAAGAAGAGAAATATTTATTATGGTTTGAAAAACCTCTTGGGACTCTTCTTTTCGACTATAAAAGATGGACTCGACCAGTTCGATATAAAAAAAATAATCGATTTGAAAATGCTGTTCGAAATGAAATGTCACACTATTTTTTTTATACATGTCGAAGTGATGGAAAAGAAAAGATCTCTTTTACGTATCCAGCAAGTCTGTCAACTTTTTTGGAAATGATAACAAAAAAAATTTCTTTTTTCACAACAGAAAAACTATCCTCTGATGAATTTTATACACATTGGAATTACACAAATGACCAAAAAAGGAAGAACTTAACCAGGGAGTTTAGAAATAGAATCGAAGTTTTAGATAAAGGATCTCTTATTCTGGATATACTCGAAAAAAGGACTCAATTGTACAATGATAAGACTAAAAAAAAATACTTACCTAAAATATATGATCCTTTATTACATGGACCTTATCGTGGAAGAATCAAAAAATTATTTTCATCCGCAATCCTAAATAAAACTTATATCAAAAATAAGATAGGAACGCTTTGGATAAATAAGATTCACAATATCATTCTTATTAATGATTATGACGAATTTGAACAGACAATAGACCGAGTTAATCGAAAATCATTTTCAAGAGAAGAAGTAGGGTCTTTATTTACAGAACACGAACGAGAACAAATCGATTCAGAAGAACGAATCCAAATTTTAAAATTTTTATTCGATGCAGTTATAACCGATCCCAATGATCAAAAAATTCGCAAAAAATCGATAAAAGAAATTAGTAAAAGAGTTCCCCGGTGGTCATACAAATTAATCGATAACTTAGACCAAGAGCTGGGAGAATACGACGAAAATGTAAGAGGGGAGCATGCATTTCGTTCACGAAAAGCCAAACGTTTAGTGGTTTCTGTTGATCACCAGACAAAAGAGGATGTGACTTTGCCACGTTATTTAGAACAATCGGATTTTCGTCGATATATAATCAAAGGTTCCATGCGCGCACAAAGACGTAAAACCTTTATTTGTAAACCGGTTCAAGCAAATGCCCATTCCCCTCTTTTTTTGGACAGAATAGACAAACCCTTTTATTTGTCTTTTGATATTTCCGGGCTAATGAAAGTAATTTTTAGAAATTGGATGTGGAAAAATAACGACCAAAAACTTTCTGATTATACAAAGGAAAGGCCAAGAAAATTAGATAAAAAAACAAAAAAGAAGCCCAAAAAAGAAAGATACACAAGACAAGAAAAGGTACGTATAAAACAAGCAGAGGGCTGGGATAAGCGTTTCCTTACTCGAGTACTAAGAAGTTCTATGTTAGTAATCCAATCGATTCTTAGAAAATATATTCTATTACCTTCATTGATAATAGCTAAAAATTTGGTTCGCATACTATTATTTCAAGATCCCGAGTGGTCGGAGGATTTTAAGGATTGGAGTCGGGAAATTTATGTTAAATGCACTTATAGTGGCGTTAATTTATCTGAAACAGAATTTCCGAAAAACTGGTTAACAGAAGGTATTCAGATAAAGATACTATTCCCTTTTCGCCTGAAACCCTGGCACAGATCTAAGATACAATCCCCTCATAAAGATGCAAAAAGTGAAGCTGATTTTTGTTTTTTAACGGCTTTGGGATTGGAAACTGAAATGCCCTTTGGTTCTCCCCGAAAACGACGTTCGTTTTTTGAACCCATTTTGAAAGAACTAAAAAAAAAAATTATAAAATTGAAAACTAAGTCTTTTATAGTTTTAAGGGTTTTCAAAGAAGGAAAAATAAAAGAACTTTCAAAAAGAAACTTGAGAGAAATCGATGAATTGGGTGAAACTCAAAAAAATTCGATACTCAGTAATCAGAAGATTCACGAATCGTCTATTGAAATTCTATCTATGGATTGGCCAAATTTCTCCCGGACCGAAAAAAAAATGACAGATCTGACTAATAGAACAAGCAGAATACGAAATCAAATATATAAAATTACAAAAGAAAATAAAAAAGTATCGCTAACTCAAGAAACAAATATTAGTTCGAACAAAACAACTTATTCTGCTAAAATATTAGACTCATCAAAAAAGATTTGGCAGATAGTCAAAAAAAGAAATACTCGATTAACCCGTAAATCCTATTTTTATGTAAATTTTTTTATTGAAAAGCTATACAGAAATTTTTTTCTATCTACCCTTACTATTCCAAGAATCAATGCAAAACCTTTTAGTGAATCAACAAGAAAAAAAATGAAAATTATTGAGAAAAACATCCACAATAATGAAGAAAATCCGGAAATAATTAATAAAACAAATCAAAATCGAATTCACTTTATTTCGACTGTCAAAAAATCACTTTCGAAGATTAGTAATAAGAATTCAAAGATTTCTTGTAATTTATCGTCTTTTTCACAATCCCAAGCGTATGTATTTTACAAATTGTTACAAGCCCCGATTTTGAACTTTTCGAATTTAAGACCTGTTCTTCAATATCACGGAACATCTCTATTTCTTAAGAATGAAATAAAGGATTTTTTTGAAAAACACGGAATATTTAATTACCAATTAAGACATAAACCTTTTTGGAATTTTGGAAGGAATCCATGGAAAAACTGGTTAAGCGGTCATTCTCACTATGATTTCTCTCGGATTAAATGGGCTAGATTAGTACGACAAGAATGGCGAAATAAAGCCAATCAACACTGTATGGCTCAAAATAAAAATTTAACTAAAAGAGATTCATATGAAAAAAACGGATTAACTCATTGCGAAAAACAACATTTTTTTGAAACAGACTCATTACGTAATCAAAAATCGAATTTTCAAAAACACTATAGATATGATCTTTTATCATATAAATTGATTAATTATGAAGATAAGAACGACTCATATATTGATAGATTACTAGGCCAAGTAAATACTAAAGAAGAGTATTATTATAATTACAATATAAAGAAAGGAAAATTATTTGCTATGCTGGGAGGTATCCTTATCAATAATTATCTAGGAGAAGATGATATTATGGATATGGAAAAATTCTTGTATAGAAAATATTTTGATTGGAGAATTCTGACTTTTTGTCTTAGAAATAAGGTCAATATTGAAGCCTGGGTTGATATGGATACTGGTACCAGCAGTAAGCAAAATACTAAGATTGGGTCCGCTAATTATAAAAAAATTGATGCAATTAATAAAAGAGGCCCCTTTTATCTTACAATTCATCAAGATGAAGAAATTAACCCATCCAACCAAAAAAGAACACTTTTTGATTGGATGGGAATGAATGAAGAAGTACTAAGTTGTCCTATATCAAACCTGGAGCCTTGGTTCTTTCCAGAATTTGTGCTACTTTTTAATGCATATAGAAGGAAACCATGGATCATACCAATCAAATTACTTCTTTTCAATTTTCATGGAAATGGTAAGAAAATTCTAACCGGAAAGAACGAAGCGGATCTTTTTATATCATCCACTCAAAAAGAATATCTTGAATTATCGAATCAAAGTAAAGAAGAAAAAGAACTCGCAGACCAAGGAAATCCGGGATCGGATGCCCAAAAGCAAGTAAGTCTTGGATCAGTTCTCTCAAACCAAGAAAAAGATGTTGAAGAAAATTATACGAGATCGGACATGAAAAAAGGTATAAAGAAAAAGCAATACAAGAGAGAAACAGAAGCCCAGCTTGATTTCTTCCTAAAAAAATATTTGTGTTTGCAGTTGAGATGGAGAGGTACTGTTTCTTTCAGGGAAAAAATACTCAATGATATGAAAGTTTATTGTCACCTGGTTCGACTGATAAATCCTAGCGACGTTACTATAGCCTCTATTCAAGGAGGAGAAATTAGTTTGGCTATTTTGATCACTAAGAAGGATTTCGCTCTTAGAGAGTTGACGAAAGGGGGAATGCTTATTATTGAACCCCGTCGTTTGTCTGTAAAAAATGATGGCCAATTTTTTATATATCAAATCGTAGGTATTTCATTGGTTCATAAGAATAAGCGCAAAATTACTAAAAGATACCGCGAAAAAGGCTATGTTGATAAAAAAAATTTTGATGAATTCATTGCAAAACATCAAAAAATGACTGGAAATAGAAACAAAAATCCTTATGATTTGCTTGTTCCTGAAAATATTTTATTCCCTAAACGTCGTAGAGAATTAAGAACTCTAATTTGTTTCAATTCGAAGAATCAAAATGGTATGCAGAGAAATCCAGTATTTTGTACTAACAGAAAAATCGGGGGTCACATTTTGGATAAAAACAAAGATCTTTCTAGAGAGAAAAATCAACTAATTAAATTAAAGTTCTTTATTTGGCCCAATTCTCGATTAGAAGATTTAATTTGTATGAATCGCTATTGGTTTAGTACCAATAATGGGAGTCGTTTCAGTATGGTAAGGATACATATGTATCCACGATTGAAAATTCGTTAA

>rps7

ATGTCACGTCGAGGTACTACAGAAGAAAAAGCTGCAAAATCCGATCCAATTTATCGTAATCGCTTAGTTAACATGTTGGTTAACCGTATTCTGAAACACGGAAAAAAATCATTGGCTTATCAAATTATCTATCGAGCCTTGAAAAAGATTCAACAAAAGACAGAAAAAAATCCACTATCTGTTTTACGTCAAGCAATACGTGGAGTAACTCCCGATATAGCAGTAAAAGCAAGACGTGTAGGCGGATCGACTCATCAAGTTCCCATTGAAATAGGATCCGCACAAGGAAAAGCACTTGCCGTTCGTTGGTTATTAGGGGCATCCCGAAAACGTCCGGGTCGAAATATGGCTTTCAAATTAAGTTCCGAATTAGTGGATGCTGCCAAAGGGAGTGGCGATGCCATACGCAAAAAGGAAGAGACTCATAGAATGGCAGAGGCAAATAGAGCTTTTGCACATTTTCGTTAA

>ndhB

ATGAAAGCCTTTCATTTGCTTCTCTTCGATGGAAGTTTTATTTTCCCAGAATGTATCCTAATTTTTGGCCTAATTCTTCTTCTGATGATCGATTCAACCTCTGATCAAAAAGATATACCTTGGTTATATTTCATCTCTTCAACAAGTTTAGTAATGAGCATAGCGGCCCTATTGTTCCGATGGAGAGAAGAACCTATGATTAGCTTTTCGGGAAATTTCCAAACGAACAATTTCAACGAAATCTTTCAATTTCTTATTTTACTATGTTCAACTCTATGTATTCCTCTATCCGTGGAGTATATTGAATGTACAGAAATGGCTATAACAGAGTTTCTGTTATTCGTATTAACAGCTACTCTAGGAGGAATGTTTTTATGCGGTGCTAACGATTTAATAACTATCTTTGTAGCTCCAGAATGTTTCAGTTTATGCTCCTACCTATTATCTGGATATACCAAGAAAGACGTACGGTCTAATGAGGCTACTATGAAATATTTACTCATGGGTGGGGCAAGCTCTTCTATTCTGGTTCATGGTTTCTCTTGGCTATATGGTTCATCCGGGGGCGAGATCGAGCTTCAAGAAATAGTGAATGGTCTTATCAATACACAAATGTATAACTCCCCAGGAATTTCAATTGCGCTTATATTCATCACTGTAGGAATTGGGTTCAAGCTTTCCCTAGCCCCTTCTCATCAATGGACTCCTGACGTATACGAAGGATCTCCCACTCCAGTCGTTGCTTTTCTTTCTGTTACTTCGAAAGTAGCTGCTTCAGCTTCAGCCACTCGAATTTTCGATATTCCTTTTTATTTCTCATCAAACGAATGGCATCTTCTTCTGGAAATCCTAGCTATTCTTAGCATGATATTGGGGAATCTCATTGCTATTACTCAAACAAGCATGAAACGTATGCTTGCATATTCGTCCATAGGTCAAATCGGATATGTAATTATTGGAATAATTGTTGGAGACTCAAATGGTGGATATGCGAGCATGATAACTTATATGCTGTTCTATATCTCCATGAATCTAGGAACTTTTGCTTGCATTGTATTATTTGGTCTACGTACCGGAACTGATAACATTCGAGATTATGCAGGATTATACACAAAAGATCCTTTTTTGGCTCTCTCTTTAGCTCTATGTCTCTTATCCCTAGGAGGTCTTCCTCCACTAGCAGGTTTTTTCGGAAAACTCCATTTATTCTGGTGTGGATGGCAGGCAGGCCTATATTTCTTGGTTTCAATAGGACTCCTTACGAGCGTTGTTTCTATCTACTATTATCTAAAAATAATCAAGTTATTAATGACTGGACGAAAGCAAGAAATAACCCCTCACGTGCGAAATTATAGAGGATCCCCTTTAAGATCAAACAATTCCATCGAATTGAGTATGATTGTATGTGTGATAGCATCTACTATACCAGGAATATCAATGAACCCGATTATTGCAATTGCTCAGGATACCCTTTTTTAG

>ycf2

ATGAAAGGACATCAATTCAAATCCTGGATTTTCGAATTGAGAGAGATATTGAGAGAGATCAAGAATTCTCACTATTTCTTAGATTCATGGACCCAATTCAATTCAGTGGGATCTTTCATTCACATTTTTTTCCACCAAGAACGTTTTATAAAACTCTTGGACCCACGAATTTGGAGTATCCTACTTTCACGCAATTCACAGGGTTCAACAAGCAATCGATATTTCACGATCAAGGGTGTAGTACTATTTGTAGTAGCGATCCTTATATATCGTATTAACAATCGAAATATGGTCGAAAGAAAAAATCTCTATTTGACAGGGCTTCTTCCTATACCTATGAATTCCATTGGACCCAGAACTGATACATTGGAAGAATCTTTTGGGTCTTCCAATATCAATAGGTTGATTGTTTCGCTCCTCTATCTTCCAAAAGGAAAAAAGATCTCTGAGAGCTTTTTCCTGGATCCGAAAGAGAGTACTTGGGTTCTCCCAATAACTAAAAAGTGTATCATGCCTGAATCTAACTGGGGTTCGCGGTGGTGGAGGAACTGGCTCGGAAAAAAGAGGGATTCTAGTTGTAAGCTATCTAATGAAACCGTCGCTGGAATTGAGATCTCATTCAAAGAGAAAGATATCAAATATCTGGAGTTTCTTTTTGTATATTATATGGATGATCCGATCCGCAAGGACCATGATTGGGAATTGTTTGATCGTCTTTCTCCGAGTAAGAGGCGAAACATAATCAACTTGAATTCGGGACATCTATTCGAAATCTTAGTGAAAGACTGGATTTGTTATCTCATGTTTGCTTTTCGTGAAAAAATACCAATTGAAGTGGAGGGTTTCTTCAAACAACAAGGAGCTGGGTCAACTATTCAATCAAATGATATTGAGCGTTTTTCCCATCTCTTCTTGAGAAACAAGTGGGCTATTTCTTTGCAAAATTGTGCTCAATTTCATATGTGGCAATTCCGCCAAGATCTCTTCGTTAGTTGGGGGAAGAATCCGCACGAATCGGATTTTTTGAGGAACATATCGAGAGAGAATTGGATTTGGTTAGACAATGTGTGGTTGGTAAACAAGGATCGATTTTTTAGCAAGGTACGGAATGTATCGTCAAATATTCAATATGATTCCACAAGATCTAGTTTCGTTCAAGTAACGGATTCTAGCCAATTGAAAGGATCTTCTGATCAATCCAGAGATCATTTCGATTCCATTAGTAATGAGGATTCGGAATATCACACATTGATCAATCAAAGAGAGATTCAACAACTAAAAGAAAGATCGATTCTTTGGGATCCTTCCTTTCTTCAAACGGAACGAAGAGAGATAGAATCAGACCGATTCCCTAAATGTCTTTCTGGATATTCCTCAATGTCCCGGCTATTCACGGAACGTGAAAGGCAGATGAATAAGCATCTGCTTCCGGAAGAAATCGAAGAATTTCTTGGGAATCCTGCAAGATCCATTCGTTCTTTTTTCTCTGACAGATGGTCAGAACTTCATCTGGGTTCGAATCCTACTGAGAGGTCCACTAGAGATCAGAAATTGTTGAAGAAAGAAGAGGATGTTTCTTTTGTCCCTTCCAGGCGATCGGAAAATAAAGAAATAGTTAATATATTCAAGATAATTACGTATTTACAAAATACCGTCTCAATTCATCCTATTTCATCAGATCCGGGATGTGATATGGTTCCGAAGGATGAACTGGATATGGACAGTTCCAATAAGATTTCATTCTTAAACAAAAATCCATTTTTTGATTTATTTCATCTATTCCATGACCGGAACAGGGGGGGATACACGTTACACCACGATTTTGAATCAGAAGAGAGATTTCAAGAAATGGCAGATCTATTCACTCTATCAATAACCGAGCCGGATCTGGTGTATCATAAGGGATTTGCCTTTTCTATTTTTTCCTACGGATTGGATCAAAAACAATTCTTGAATGAGGTATTCAACTCCAGGAATGAATCGAAAAAGAAATCTTTATTGGTTCTACCTCCTATTTTTTATGAAGAGAATGAATCTTTTTATCGAAGGATCAGAAAAAAATGGGTCCGGATCTCCTGCGGGAATGATTTGGAAGATCCAAAACCAAAAAGAGTGGTTTTTGCTAGCAACAACATAATGGAGGCAGTCAATCAATCTAGATTGATCCGAAATCGGATTCAAATCCAATATAGCACCTATGGGTACATAAGAAATGTATTGAATCGATTCTTTTTAATGAATAGATCCGATCGCAACTTCGAATATGGAATTCACAGGGATCAAATAGGAAATGATACTCTGAATCATAGAACTATAATGAAATATACGATCAACCAACATTTATCGAATTTGAAAAAGAGTCAGAAGAAATGGTTCGCTCCTCTTATTTTTATTTCTCGAACCGAGAGATTCATGAATCGGGATCCTAATGCATATAGATACAAATGGTCCAATGGGAGCAAGAATTTCCAGGAGCATTTGAAACATTTCGTTTCTGAGCAGAAGAGCCGTTTTCAAGTAGTGTTCGATCGATTACGTATTAATCAATATTCGATTGATTGGTCTGAAGTTATCGACAAAAAAGATTTGTCTAAGTCACTTCCTTTCTTTTTGTCCAAGTTGCTTTTCTTTTTGTCTAACTCACTTCCTTTTTTCTTTGTGAGTTTCGGGAATATCCCCATTCATAGGTCTGAGATCCACATCTATGAATTGAAAGGTCCGAATGATCAACTCTGCAATCAGTTGTTAGAATCAATAGGTCTTCAAATCGTTCATTTGAAAAAATTGAAACCCTTCTTATTGGATGATCATAATACTTCCCAAAAATCGAAATTCTTGATCAATGGAGGAAGAATATCACCATTTTTGTTCAATAAGATACCAAAGTGGATGATTGACTCATTCCATACTAGAAATAATCGCAGGAAATCTTTTGATAACACGGATTCCTATTTCTCAACGATATCCCACGATCAAGACAATTGGCTGAATCCTGTGAAACCATTTCATAGAAGTTCATTGATATCTTCTTTTTATAAAGCAAATCGACTTCGATTCTTGAATAATCCACATCGCTTCCGCTTCTATTGTAACAAAAGATTCACTTTTGATGTGGAAAAGGTCCGTATCAATAATTATGATTTTACGTATGGACAATTCCTCAATATCTTGTTCATTCGCAACAAAATATTTTCTTTGTGCGGCGGTAAAAAAAAACATGCTTTTTTGGAGAGAGATACTATTTCACCAATCGAGTCACAGGTATCTAACATATTGATACCTAACGATTTTCCACAAAGCGGTGACGAAAGGTATAACTTGTACAAATTTTTCCCTTTTCCAATTCGATCCGATCTATTAGTTCATAGAGCTATTTACTCGATCGCAGCCATTTCTGGAACACCTCTAACAGAGGGACAAATAGTCAATTTTGAAAGAACTTATTGTCAACCTCTTTCAGATATGAATCTATCTGATTCAGACGAGAAGAACTTGCATCAGTATCTCAATTTCAATTCAAACATGGGTTTGATTCACACTCCATGTTCTGAGAAATATTTACCATCCGAAAAGAGGAAAAAACGGAGTCTTTGTCTAAAGAAATGCGTTGAGAAAGGGCAGATGTCTAGAACCTTTCAACGAGACAGTGCTTTTTCAACTCTCTCAAAATGGAATCGATTCCAAACATATATGCCATGGTTCCTTACTTCGACAGGGTACAAATATCTAAATTTTCTATTTTTAGATACTTTTTCAGACCTATTGCCGGTACTAAGTAGCAGTCAAAAATTTGTATCCATTTTTCATGATATTATGCATGGATCAGATATATCATGGCGAATTCTTCAGAAAAAATGGCGTCTTCCACAATGGAATCTGATAAGTGAGATTTCGAGTAAGTGTTTCCATAATCTTCTTCTGTCCGAAGAAATGATTCATCGAAATAATGAGTCACCATTGATATCGACACATCTGAGATCGCCAAATGCTCGGGAGTTCCTCTATTCAATCCTTTTCCTTCTTCTTGTTGCTGGATATCTCGTTCATACACATCTTATCTTTGTTTCCCGAGCCTATAGTGAGTTAGAGACAGAGTTCGAAAGGGTCAAATCTTTGATGATTCCATCATACATGATTGAGTTGCGAAAACTTCTGGATAGGTATCCCACATCTGAACTGAATTCTTTCTGGTTAAAGAATCTCCTTCTAGTTGCTCTGGAACAATTAGGAGATTCTCTAGAAGAAATACGGGGTTCTGCTTTTGGCGGCAACATGCTATGGGGTGGTGGTCCCGCGGATGGGGTTAAATCAATACGTTCTAAGACGAAAGATTTGAATATCAATCTCATCGATATCATCGATCTCATAAGTATCATACCAAATCCCATCAATCGAATCACTTTTTCGAGAAATACGAAACATCTAAGTCATACAAGTAAAGAGATCTATTCATTGATAAGAAAAAGAAAAAAGGGGAACGGTGATTGGATTGATGATAAAATAGAATCCTGGGTCGCGAACAGTGATTCGATTGATGATAAAGAAAGAGAATTCTTGGTTCAGTTCTCCACCTTAACGACAGAAAAAAGGATTGATCAAATTCTATTGAGTCTGACTCATAGTGATCATTTATCAAAGAATGACTCTGGTTATCAAATGATTGAACAACCGGGAACAATTTACTTACGATACTTAGTTGACATTCATAAAAAGCATTTCATGAATTATGAGTTCAATACATACTGTTTAGCAGAAAGACGGATATTCCTTGCTCATTATCAGACAATCACTTATTCACAAACTTCGTGTGGGGCTAATAGTTTTCATTTCCCATCTCATGGAAAACCCTTTTCGCTCCGCCTAGCCCTATCCCCCTCTAGGGGTATTTTAGTGATAGGTTCTATAGGAACCGGACGCTCCTATTTGGTCAAATACCTAGCGACAAACTCCTATGTTCCTTTCATTACAGTATTTCTGAACAAGTTCCTGGATAACAAGCCTAAAGGTTTTCTTATTGATGATATCGATGATGATAGTGACGATATTGATGCTAGTGACGATATTGATGCTAGTGACGATATCGATCGTGACCTTGATACGGAGCTGGAGCTTCTAACTATGATGAATGCGCTAACTATCGATATGATGTCGGAAATAGGCCGATTTTATATCACCCTTCAATTCGAATTAGCAAAAGCAATGTCTCCTTGCATAATATGGATTCCAAACATTCATGATCTGGATGTGAATGAGTCGAATTACTTATCCCTCGGTCTATTAGTGAACTATCTCTCCAGGGATTGTGAAAGATGTTCCACTAGAAATATTCTTGTTATTGCTTCGACTCATATTCCCCAAAAAGTGGATCCCGCTCTAATAGCCCCGAATAAATTAAATACATGCATTAAGATACGAAGGCTTCTTATTCCACAACAACGAAAGCACTTTTTCACTCTTTCATATACTAGGGGATTTCACTTGGAAAAGAAAATGTTCCATACTAATGGATTCGGGTCCATAACCATGGGTTCCAATGCACGAGATCTTGTAGCACTTACCAATGAGGCCCTATTGATTAGTATTACACAGAAGAAATCAATTATAGACACTAATACAATTAGATCTGCTCTTCATAGACAAACTTGGGATTTGCGATCCCAGGTAAGATCGGTTCAAGATCATGGGATCCTTTTCTATCAGATAGGAAGGGCTGTTGCACAAAATGTACTTCTAAGTAATTGCCCCATAGATCCTATATCTATCTATATGAAGAAGAAATCATGTAACGAAGGGGATTCTTATTTGTACAAATGGTACTTCGAACTTGGAACGAGCATGAAGAAATTAACGATACTTCTTTATCTTTTGAGTTGTTCTGCCGGATCGGTCGCTCAAGACCTTTGGTCTCTACCCGGACCCGATGAAAAAAACGGGATCACTTCTTCTGGACTCGTTGAGAATGATTCTGATCTAGTCCATGGCCTATTAGAAGTAGAAGGCGCTCTGGTGGGATCCTCGCGGACAGAAAAAGATTGCAGTCGGTTTGATAATGATCGAGTGACATTGCTTCTTCGGCCCGAACCAAGGAATCCCTTAGATATGATGCAAAAAGGATCTTGTTCTATCGTTAATCAGAGATTTCTCTATGAAAAATACGAATCGGAGTTTGAAGAAGGGGAAGGAGAAGGAGTCCTCGACCCGCAACAGATAGAGGAGGATTTATTCAATCACATAGTCTGGGCTCCTAGAATATGGCGCCCCTGGGGCTTTCTATTTGATTGTATCGAAAGGCCCAATGAATTGGGATTTCCCTATTGGGCCGGGTCATTTCGGGGCAAGCGGATCATTTATGATGAAAAGGATGAGCTTCAAGAGAATGATTCGGAGTTCTTGCAGAGTAGAACCATGCAGTACCAGACACGAGATAGATCTTCCAACGAACAAGGCTTTTTTCGAATAAGCCAATTCATTTGGGAACCTGCGGATCCACTCTTTTTCCTATTCAAAGATCAGCCCCTTGTCTCTGTGTTTTCACACCGAGAATTCTTTGCAGATGAAGAGATGTCAAAGGGGCTTCTTACTTCCCAACCAGATCCTCCTACATCTATATATAAACGCTGGTTTATCAAGAATACGCAAGAAAAGCACTTCGAATTGTTGATTCATCGCCAGAGATGGCTTAGAACCAATAGTTCATTATCTAATGGATTTTTCCGTTCGAATACTCCATCCGAGAGTTATCAGTATTTATCAAATCTCTTCCTATCTAACGGAACGCTATTGGATCAAATGACAAAGACATTGTTGAGAAAAAGATGGCTTTTTCCGGATGAAATGAAAATTGGATTCATGTAA

>rpl23

ATGGATGGAATCAAATATGCAGTATTTACAGACAAAAGTATTCGGTTATTGGGGAAAAATCAATATACTTCTAATGTCGAATCAGGATCAACTAGGACAGAAATAAAGCATTGGGTCGAACTCTTCTTTGGTGTCAAGGTAATAGCTATGAATAGTCATCAACTCCCCCGAAAGGGTAGAAGAATGGGACCTATTATGGCACATACAATGCATTACAGACGTATGATCATTACGCTTCAACCGGGTTATTCTATTCCACCTCTTAGAAAGAAAAGAACTTAA

>rpl2

ATGGCGATACATTTATACAAAACTTCTACCCCGAGCACACGCAATGGAGCCGTAGACAGTCAAGTGAAATCCAATCCACGAAATAATTTGATCTATGGACAGCATCGTTGTGGTAAAGGTCGTAATGCCAGAGGAATCATTACCGCAGGGCATAGAGGGGGAGGTCATAAGCGTCTATACCGTAAAATCGATTTTCGACGGAATGCAAAAGACATATATGGTAGAATCGTAACCATAGAATACGACCCTAATCGAAATGCATACATTTGTCTCATACACTATGGGGATGGTGAGAAGAGATATATTTTACATCCCAGAGGGGCTATAATTGGAGATACCATTGTTTCTGGTACAGAAGTTCCTATAAAAATGGGAAATGCCCTACCTTTGACCGATATGCCCTTAGGCACGGCCATACATAACATAGAAATCACACTTGGAAAGGGTGGACAATTAGCTAGAGCAGCGGGTGCTGTAGCGAAACTGATTGCAAAAGAGGGGAAATCGGCCACATTAAAATTACCTTCTGGGGAGGTCCGTTTGATATCCAAAAACTGCTCAGCAACAGTCGGACAAGTGGGGAATGTTGGGGTGAACCAGAAAAGTTTGGGTAGAGCCGGATCGAAATGTTGGCTAGGTAAGCGTCCTGTAGTAAGAGGAGTAGTTATGAACCCTGTAGACCACCCCCATGGGGGTGGTGAAGGAAGGGCTCCAATTGGTAGAAAAAGACCCGCAACCCCTTGGGGTTATCCTGCACTTGGAAGAAGAAGTAGAAAAAGGAATAAATATAGTGATAATTTGATTCTTCGCCGCCGTACTAAATAG

***T. ruticarpum***

>psbA

ATGACTGCAATTTTAGAGAGACGCGAAAGCGAACGCCTATGGGGTCGCTTCTGTAACTGGATAACCAGCACCGAAAACCGCCTTTACATTGGATGGTTTGGTGTTTTGATGATCCCTACTTTATTGACTGCAACTTCTGTATTTATTATCGCCTTCATTGCTGCCCCTCCAGTAGATATTGATGGTATTCGTGAACCTGTTTCTGGATCTCTACTTTATGGAAACAATATTATTTCTGGTGCGATTATTCCTACTTCTGCAGCTATAGGTTTGCACTTTTACCCGATATGGGAAGCGGCATCTGTTGATGAATGGTTATACAATGGTGGTCCTTATGAGCTAATTGTTCTACACTTCTTACTTGGTGTAGCTTGTTACATGGGTCGTGAGTGGGAACTTAGTTTCCGTCTGGGTATGCGCCCTTGGATTGCTGTTGCATATTCAGCTCCTGTTGCAGCAGCGACTGCTGTTTTCTTGATCTACCCAATCGGTCAAGGAAGTTTTTCTGATGGTATGCCTCTAGGAATCTCTGGTACTTTCAACTTCATGATTGTATTCCAAGCCGAGCACAACATCCTTATGCACCCATTCCACATGTTAGGCGTAGCTGGTGTATTCGGCGGCTCCCTATTCAGTGCTATGCATGGTTCCTTGGTAACCTCTAGTTTGATCAGGGAAACTACAGAAAATGAATCTGCTAATGAAGGTTACAGATTCGGTCAAGAGGAAGAAACTTATAATATCGTAGCTGCTCACGGTTATTTTGGCCGATTGATCTTCCAATATGCTAGTTTCAACAATTCTCGTTCTTTACATTTCTTCCTAGCTGCTTGGCCTGTAGTAGGTATCTGGTTTACCGCTTTAGGTATTAGCACTATGGCTTTCAATCTAAATGGTTTCAACTTCAATCAATCCGTAGTTGATAGTCAAGGTCGTGTAATTAATACCTGGGCTGATATTATTAATCGTGCTAATCTTGGTATGGAAGTTATGCATGAACGTAATGCTCATAACTTCCCTCTAGACCTAGCTGCTATTGAAGCTCCATCTACAAATGGATAA

>matK
[truncated: 261,596 more chars]
